# Tetramate Derivatives by Chemoselective Dieckmann Ring Closure of *threo*-Phenylserines and Their Antibacterial Activity

**DOI:** 10.1021/acs.joc.2c01382

**Published:** 2022-09-02

**Authors:** Liban Saney, Kirsten E. Christensen, Xiang Li, Miroslav Genov, Alexander Pretsch, Dagmar Pretsch, Mark G. Moloney

**Affiliations:** † The Department of Chemistry, Chemistry Research Laboratory, University of Oxford, 12 Mansfield Road, Oxford OX1 3TA, U.K.; ‡ Department of Pharmaceutical Engineering, China Pharmaceutical University, Nanjing 211198, P. R. China; § Oxford Suzhou Centre for Advanced Research, Suzhou Industrial Park, Building A, 388 Ruo Shui Road, Jiangsu 215123, P.R. China; ∥ Oxford Antibiotic Group, The Oxford Science Park, Magdalen Centre, Oxford OX4 4GA, U.K.

## Abstract

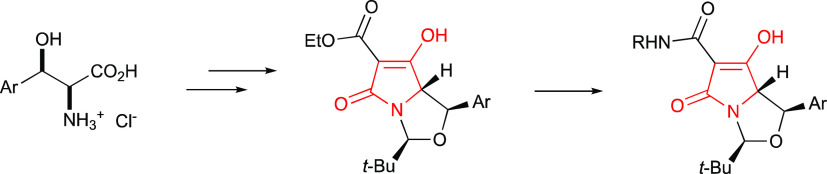

A general route, which provides direct access to substituted bicyclic tetramates, making use of Dieckmann cyclization of oxazolidines derived from *threo*-arylserines, is reported; the latter were found to be available by an efficient aldol-like reaction of glycine with some substituted benzaldehydes under alkaline conditions. The tetramates were found to release chelated metal cations acquired during chromatographic purification by mild acid wash. Some compounds in the library showed good antibacterial activity against Gram-positive bacteria. Cheminformatic analysis demonstrates that the most active compounds were Ro5-compliant and occupy a narrow region of chemical space, distinct from that occupied by other known antibiotics, with the most potent compounds having 399 < M_w_ < 530 Da; 3.5 < cLog*P* < 6.6; 594 < MSA <818 Å^2^; 9.6 < rel. PSA <13.3%. MIC values were shifted to higher concentrations when tested in the presence of HSA or blood, but was not completely abolished, consistent with a plasma protein binding (PPB) effect.

## Introduction

**Figure 1:**
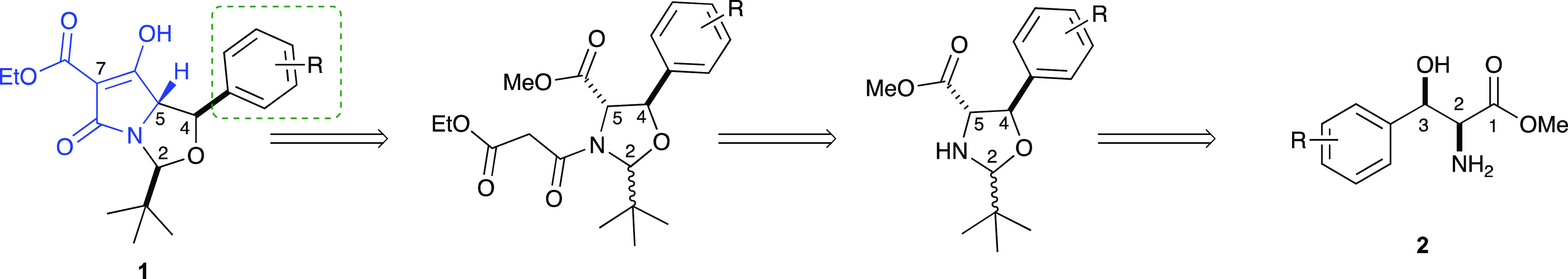
Retrosynthesis of tetramates leading to β-arylserines

The tetramate system occurs as a scaffold in natural products which exhibit a wide range of bioactivities,^1−3^ and we have previously established that bicyclic tetramates are readily available from malonyloxazolidines by highly chemoselective and stereoselective Dieckmann ring closures^4^ of oxazolidines and thiazolidines derived from serine,^4^ threonine, and cysteine; differently substituted threonines effectively gave cyclization,^5^ but thus far this approach has been limited to such readily available amino acids. Of interest was whether phenylserines could be used for similar cyclizations, especially so since such systems would also further expand the substrate scope by enabling greater functional group diversity around the bicyclic ring (Figure 1). In order to obtain aryl-substituted tetramates of type **1**, we required rapid access to diverse β-hydroxy-β-aryl-α-amino acids **2**. While the synthesis of the latter has been the focus of recent attention,^6^ we sought in the first instance a reliable and rapid diastereoselective synthesis, which would enable evaluation of the downstream Dieckmann cyclization in the presence of the additional aryl C-4 substituent on the oxazolidine ring. Thus, the first task devolved to finding an effective racemic but diastereoselective synthesis of β-arylserines which could be run conveniently and at scale, followed by their application to the construction of substituted tetramates.

## Results and Discussion

**Scheme 1:**
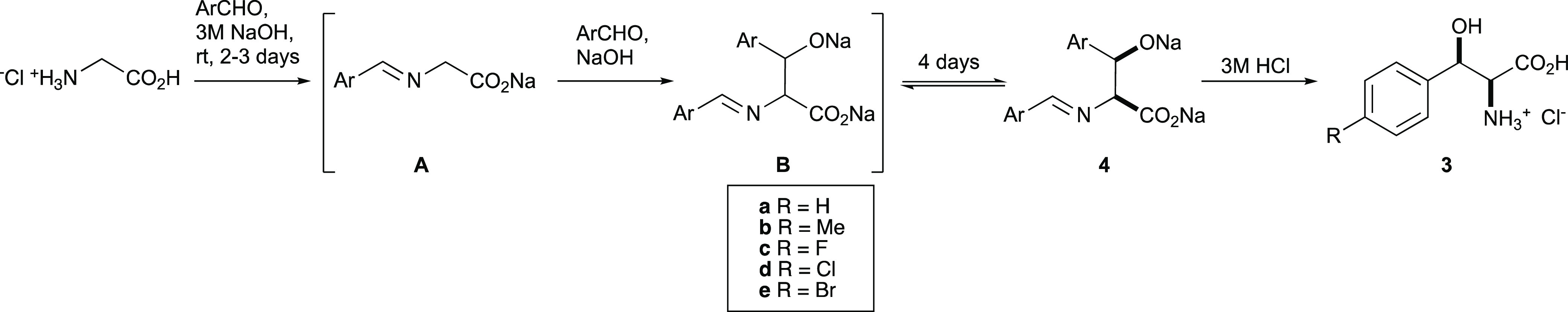
Synthesis of β-arylserines

We took inspiration from Erlenmeyer’s report^7^ that glycine could be reacted with two equivalents of benzaldehyde to form β-aryl serine **3** under basic conditions via an aldol reaction (Scheme 1).^8^ The course of this reaction is believed to proceed by the reaction of glycine with the first equivalent of benzaldehyde to form imine **A**, which in turn reacts with a second equivalent of benzaldehyde under the basic conditions via an aldol reaction to form the initial product **B** as its disodium salt. This salt, which precipitates out of solution as a condensation cake, is then subsequently protonated to form β-arylserine products **3**, along with regeneration of the starting benzaldehyde (Scheme 1). This sequence has the obvious appeal that variation of the aldehyde component would give direct access to substituted derivatives, but in the event, it was quickly found that the diastereoselectivity in the formation of the two contiguous stereocenters strongly depended on reaction time, solvent, and temperature, for different aldehydes, and considerable optimization was found to be necessary (Table 1). The formation of the condensation cake seemed to play a pivotal role toward the achievement of high diastereoselectivities, and it was found that leaving the condensation cake for a period of 3 days prior to acid treatment led to much higher diastereoselectivity (93:7 dr) of **3a**, while immediate protonation gave lower diastereoselectivity (58:42 dr) (compare entries 1 with 3, Table 1). It was believed that the higher levels of diastereoselectivity most likely arose due to equilibration of the initial adduct **B** toward the thermodynamically more stable *threo*-aldol product **4** (Scheme 1). Moreover, it was found that lowering the reaction temperature of the condensation cake by fridge storage prior to acid treatment also lowered the diastereoselectivity—presumably since the equilibration toward the *threo* product was slowed (entry 5, Table 1); however, increasing the reaction temperature to more than 50 °C led to unidentifiable by-products being formed along with the products **3a** being formed in high diastereoselectivity (98:2 dr) (entry 4, Table 1). Thus, the optimum temperature for this reaction was room temperature. The base concentration was found not to adversely affect diastereoselectivity (compare entries 1 with 2; Table 1), and so, 3M NaOH (aq) was used. Overall, the optimum procedure (entry 3, Table 1) found for the synthesis of **3a** was the addition of 1 eq. of glycine to 3M NaOH (1.5 eq.) (aq) solution and the solution left to stir for 10 min. Benzaldehyde (2.1 eq) was then added at room temperature to form the initial aldol product **B**, which led to the formation of a condensation cake that was left for 3 days at room temperature. The thus obtained aldol product **4a** was then subsequently treated with 3M HCl (aq) to form β-aryl serine **3a** along with regenerated benzaldehyde. The excess aldehyde could be removed from the desired β-aryl serine **3a** by addition of Et_2_O and water, and separation of the layers followed by evaporation of the aqueous layer to dryness in vacuo gave β-aryl serine **3a** in high yield and good diastereoselectivity, along with NaCl. Of greatest value to us was this high stereoselectivity, rather than yield and purity, and crude material was taken forward in the synthetic sequence.

**Table 1:** Optimization of the Formation of Amino Acids **3a,e**

entry	solvent	temperature (^o^C	base	reaction time after condensation cake formation before hydrolysis	dr for threo-erythro isomers^a^
1^b^	water	25	3M NaOH	immediate	58:42
2^b^	water	25	6M NaOH	immediate	70:30
3^b^	water	25	3M NaOH	72 h	93:7
4^b^	water	50	6M NaOH	24 h	98:2
5^b^	water	5–8	6M NaOH	24 h	70:30
6^c^	water	25	3M NaOH	72 h	66:34
7^c^	ethanol	25	3M KOH	72 h	94:6

a Determined from ^1^H NMR studies of crude material.

b Benzaldehyde.

c 4-Bromobenzaldehyde.

An examination was made of the scope of this approach by using substituted benzaldehydes. However, with 4-bromobenzaldehyde, the expected β-aryl serine **3e** did not form in significant quantities and mostly unreacted glycine was obtained (entry 6, Table 1). This was thought to be due to solubility differences between the various aldehydes in the water solvent medium, but when an ethanolic 3M KOH solution was used, β-aryl serines **3b–e** were routinely obtained in good yields and diastereoselectivities (compare entry 6 with 7, Tables 1 and 2), with the exception of *p*-anisaldehyde, which gave poorer diastereoselectivity (Table 2). In the latter case, no condensation cake was formed, even after 3 days of stirring. Acid hydrolysis of the resultant liquid reaction mixture led to a 3:2 mixture of diastereomers of **3f**, although this diastereomeric ratio was not reproducible from reaction to reaction; this outcome indicates the importance that the condensation cake has in driving diastereoselectivity toward the *threo*-β-aryl serine. Access to the small library of β-aryl serines **3a-e** was sufficient for our purposes, with the main focus being on the parent *threo*-phenylserine derivative, **3a**.

**Table 2:** Key Chemical Shifts (δ), Coupling Constants (*J*), Diastereomeric Ratio (dr), and Conversion of β-aryl Serines **3a-f** (Scheme 1)

compound	*R*	δ H2 (ppm)^a^	δ H3 (ppm)^a^	*J*_H2–H3_ (Hz)^a^	dr for *threo-erythro* isomers^b^	conversion (%)^c^
**3a**	H	4.07	5.37	4.1	93:7	Quant.
**3b**	Me	4.26	5.40	4.2	19:1	Quant.
**3c**	F	4.10	5.35	4.3	84:16	76
**3d**	Cl	4.22	5.39	4.0	9:1	68
**3e**	Br	4.28	5.38	3.9	94:6	quant.
**3f**	OMe	4.23	5.32	4.2	3:2	quant.

a D_2_O solvent, 400 MHz.

b Determined from ^1^H NMR studies of crude material.

c Unpurified product.

**Scheme 2:**
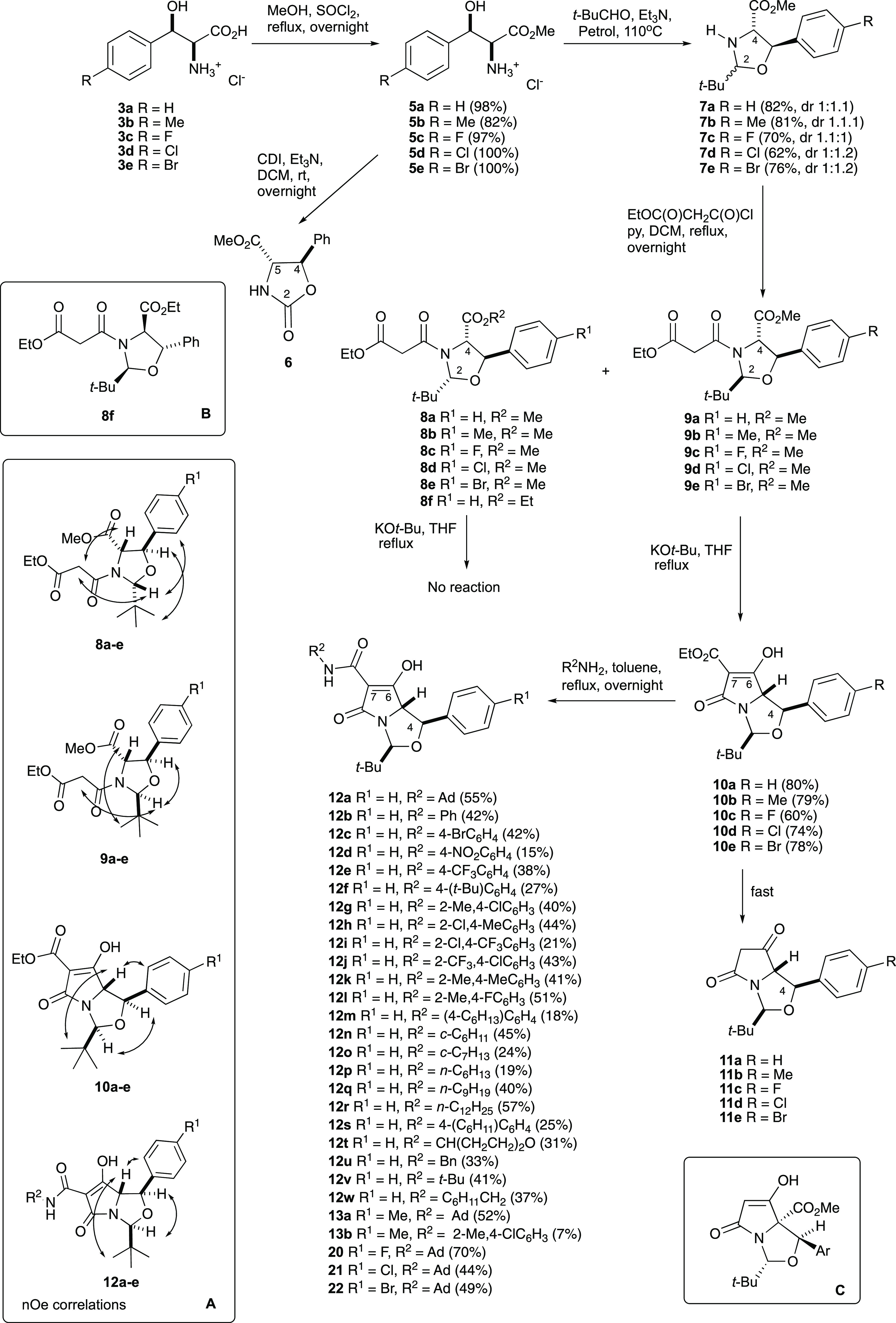
Synthesis of tetramates

With β-aryl serines **3a-e** in hand, esterification using thionyl chloride in MeOH was examined. It was found that stirring at 40 °C for 3 h led to an incomplete reaction—as determined from NMR analysis—however, refluxing overnight led to the complete conversion to methyl ester hydrochloride salts **5a-e** in excellent yields (Scheme 2).^9^ In order to confirm that the amino acids **3a-e** were indeed the *threo* diastereomers, amino ester **5a** was converted to oxazolidinone **6** with 1,1′-carbonyldiimdazole (CDI) and Et_3_N at rt overnight (Scheme 2). The ^1^H NMR data for *trans*-oxazolidinone **6** were consistent with literature NMR data (*threo*-derived oxazolidinones^10^ and *erythro*-derived oxazolidinones^11^), confirming that the amino acid **3a** formed was indeed the *threo* isomer (Table S1).

Using literature precedent,^4,12−14^ amino esters **5a–e** were reacted with pivaldehyde in the presence of Et_3_N and petroleum ether 40:60, at 100 °C for 16–24 h to form oxazolidines **7a–e** in good yields as roughly a 1:1 mixture of diastereomers at the C2 center, as determined from ^1^H NMR spectroscopic studies of the crude material (Scheme 2). That this mixture of diastereomers was observed arose from ring-chain tautomerization involving the heterocycle and the corresponding imine.^13^ nOe studies were used to distinguish between the 2,4-*cis* and 2,4-*trans*-oxazolidine diastereomers, and the key chemical shifts, coupling constants, and yields of the oxazolidine diastereomers **7a–e** confirmed their formation (Table S2, Scheme 2 and Figure S1). Importantly, the relative stereochemistry of oxazolidines **7a–e** at positions C4 and C5 was conserved from the starting amino esters **5a–e**.

An attempt to purify these oxazolidines by chromatographic purification gave low recovery of the product, and this is likely to arise from retro-aldol reactions for oxazolidines with C2 aromatic substituents, which have been reported previously.^15^ As the crude oxazolidines **7a–e** were relatively pure by NMR analysis, they were therefore used directly and *N*-acylated using ethyl hydrogen malonate under DCC/DMAP coupling conditions to furnish the malonamide products, although in poor yields. Application of more forcing conditions, by reaction with ethyl malonyl chloride under basic conditions, gave malonamides **8a–e** and **9a-e** in better yields—typically between 60 and 88%—as approximately a 3:2 mixture of diastereomers at the C2 center (Scheme 2 and Table S3). These were obtained as stable oils but were extremely difficult to separate by chromatography.

**Figure 2:**
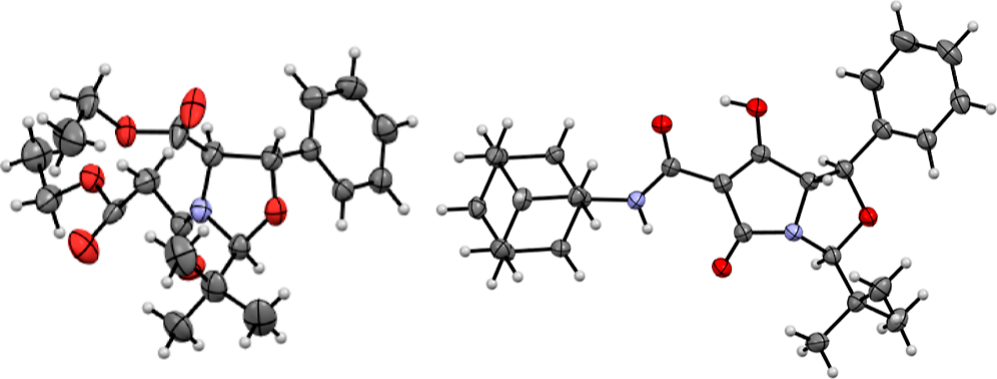
X-ray structures of ethyl ester derivative **8f** and carboxamidotetramate **12a**.

The relative stereochemistry of 2,4-*cis*-malonamides **8b–d** was assigned using nOe and NOESY spectroscopic studies (Scheme 2, inset A; and Figure S1), and confirmed by single crystal X-ray diffraction of the racemic ethyl ester analogue **8f** (Scheme 2, inset B; Figure 2) prepared by an analogous route which also showed NMR characteristics consistent with the methyl derivatives (Table S3).^16^ In the case of 2,4-*trans*-malonamides **9a–d**, low temperature VT ROESY experiments were used to determine their relative stereochemistry (Figure S1); this is further discussed below. Importantly, there was no evidence of any epimerization from the starting relative configuration at the C4 and C5 positions of malonamides **8a–e** and **9a–e** under the basic conditions of the *N*-acylation, and overall, the *N*-acylation of oxazolidines **7a–e** was not selective, unlike similar reactions of oxa(or thia)zolidines derived from serine, threonine, and cysteine,^5^ suggesting no substantial energy difference between malonamides **8a–e** and **9a–e**.

Of particular interest was the contrasting NMR behavior of malonamides **8a–e** with **9a–e**. Malonamides **8a–e** showed sharp NMR peaks at room temperature, whereas malonamides **9a–e** had very broad NMR signals (Figure S2), to the point where key signals disappeared, and this was attributed to fluxional ring conformational effects. For the former, all substituents may reside in pseudo-equatorial positions giving a clear-cut energy minimum, but for the latter, at least one must reside in a pseudo-axial position, giving several energetically low-lying structures which are each visible and averaged on the NMR time-scale. Low temperature VT-NMR experiments slightly sharpened the NMR peaks (Figure S3).

Initially, the diastereomeric mixtures of malonamides **8a–e** and **9a-e** were reacted directly with KO^*t*^Bu in THF overnight at reflux. However, poor yields were obtained for the product tetramates **10a–e**–typically between 30 and 40%—along with other unidentified side products. In order to understand why such poor yields of tetramates arose, a small amount of each malonamide diastereomer was separated by careful chromatographic purification and each reacted under Dieckmann conditions individually with KO^*t*^Bu in THF overnight at reflux. Of interest is that malonamides **8a–e** proved to be resistant to the Dieckmann cyclization (Scheme 2), with the starting material being mostly recovered—as determined from TLC and ^1^H NMR studies—along with other unidentified side-products. However, for malonamides **9a–e**, tetramates **10a–e** were readily obtained in good yields whose relative stereochemistry was determined from nOe studies (Scheme 2, inset A) (Table S4); these tetramates **10a–e** were found to be unstable to chromatographic purification, and this is similar to that observed by Andrews et al. for analogous C7-ethyl ester tetramates derived from l-serine.^4^ They were also unstable in CDCl_3_ solution, where they rapidly degraded to C7-decarboxylated tetramates **11a–e**—as determined from NMR spectroscopic and mass spectrometric studies (Scheme 2). Fortunately, the crude tetramates **10a–e** were relatively pure by NMR analysis and could be used directly. Of interest is that no products arising from the alternative mode of ring cyclization were observed (Scheme 2, inset C).

**Scheme 3:**
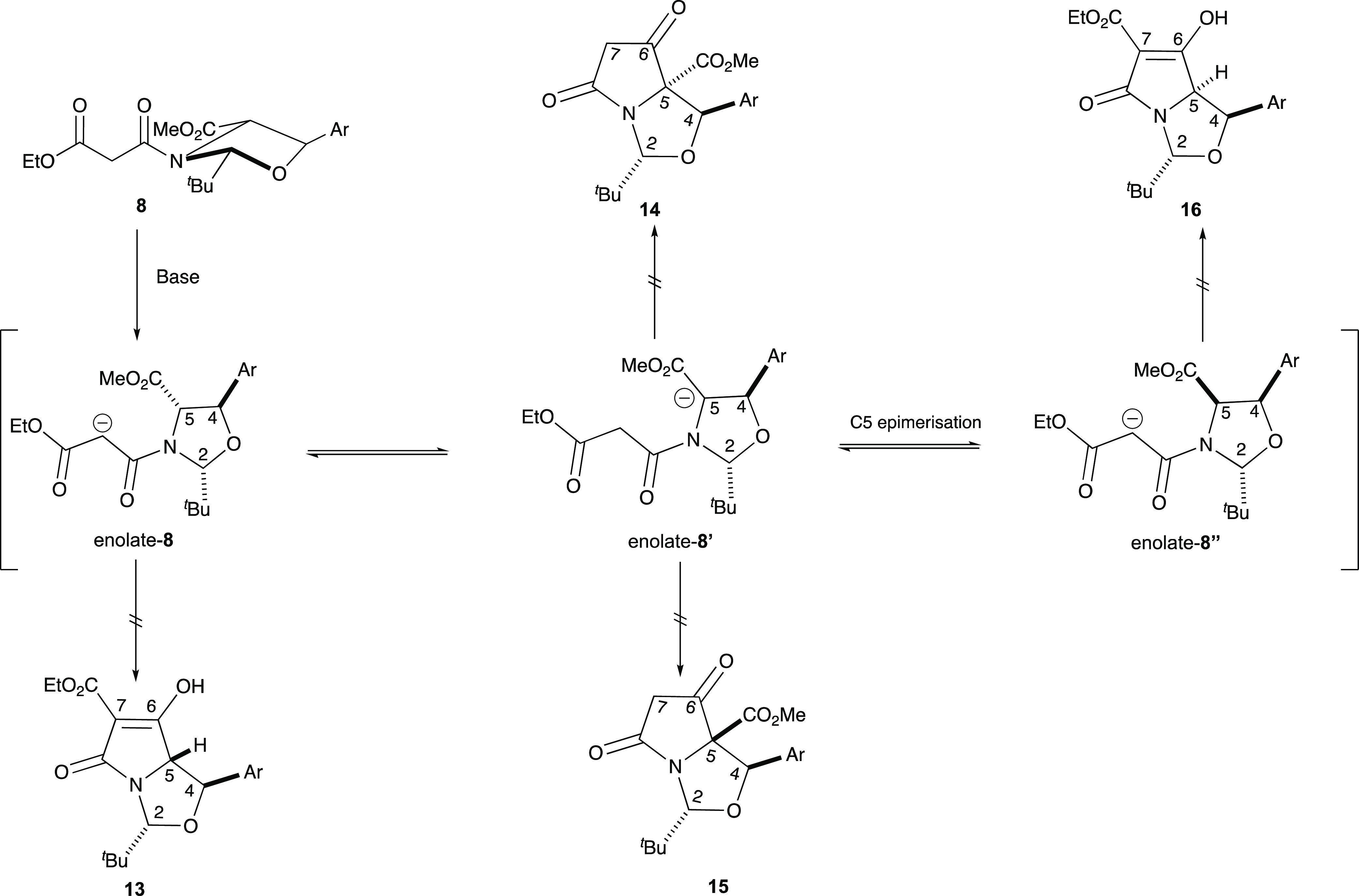
Selectivity for cyclization(*cis*-isomer)

**Scheme 4:**
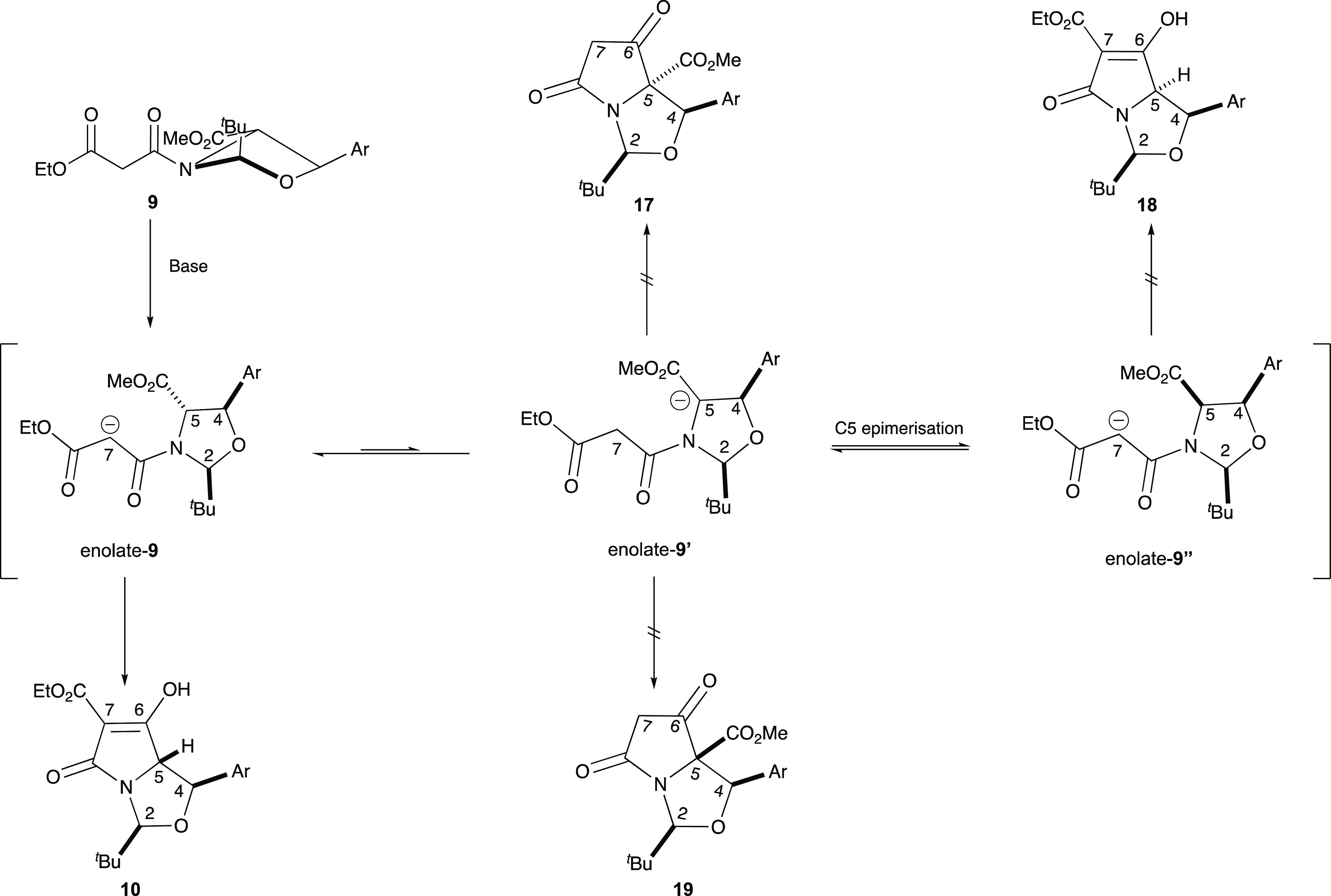
Selectivity for cyclization(*trans*-isomer)

The high levels of chemoselectivity observed for the formation of tetramates **10a–e** from malonamides **9a–e**, compared to the resistance of malonamides **8a–e** to Dieckmann cyclization, was attributed to steric constraints imposed by the bicyclic lactam structure and especially the bulky *t*-butyl substituent. Thus, in the case of 2,4-*cis*-malonamides **8**, ring closure of the potentially freely equilibrating enolate **8**, enolate **8**′, or enolate **8**″ to any of products **13-16** places one of the C-2*t*-Bu or C-4 aryl groups on the more hindered *endo*-face of the bicyclic product (Scheme 3), none of which are favourable. Similarly, for malonamides **9**, ring closure to tetramates **17** and **18** also places both C-2*t*-Bu and C-4 aryl groups on the more hindered *endo*-face (Scheme 4), and so, both these products are therefore not favored. However, both of **10** and **19** enjoy the location of C-2*t*-Bu and C-4 aryl groups on the less hindered *exo*-face, but of these, only **10** is formed via the more stable enolate; since the formation of **19** requires access to the thermodynamically more unstable enolate **9′**, it is therefore not observed.

In order to understand this process in more detail, DFT calculations were conducted invoking all the intermediates and transition states along with solvent (THF) and base (KO^*t*^Bu) (Figure S4). Of note is that malonamide **8a** was more stable than malonamide **9a** by approximately 4.1 kcal/mol (17.2 kJ/mol), accounting for its experimentally observed slight diastereomeric preference (Table S3 and Scheme 2). For the deprotonation of malonamides **8a** and **9a** using KO^*t*^Bu as a base, there was a significant energy difference between enolate **enolate-9a** and the next enolate highest in energy, **enolate-9a’**—about 20.3 kcal/mol (84.9 kJ/mol)—so that formation of the latter is strongly disfavored; this was in agreement with the experimentally observed chemoselectivity (Scheme 4). **Enolate 8a**, **enolate 8a′**, and **enolate-9a** were all of similar energy. The activation energy, E_a_, for the lowest energy transition state was calculated to be **enolate-9a**, which had an *E*_a_ = 11 kcal/mol (46 kJ/mol). The next highest energy transition states were **enolate-8a**, which had an *E*_a_ = 20.3 kcal/mol (84.9 kJ/mol), and **enolate-8a′**, which had an E_a_ = 19.5 kcal/mol (81.6 kJ/mol), suggesting that **enolate-9a** was the kinetically more favored reaction pathway leading toward the formation of tetramate **10a** (pathway highlighted in red in Figure S4), and consistent with experiment. Moreover, tetramate **10a** was considerably more stable than tetramates **15** and **13**, by 12.1 (50.6 kJ/mol) and 9.3 kcal/mol (38.9 kJ/mol), respectively. Overall, this suggested that tetramate **10a–e** was both the kinetic and thermodynamic product. This outcome likely arises from the steric effect of the *t*-butyl group, which while simultaneously protecting the O and N groups, also provides a significant steric bias to influence reaction chemoselectivity and therefore direction of ring closure.

**Figure 3:**
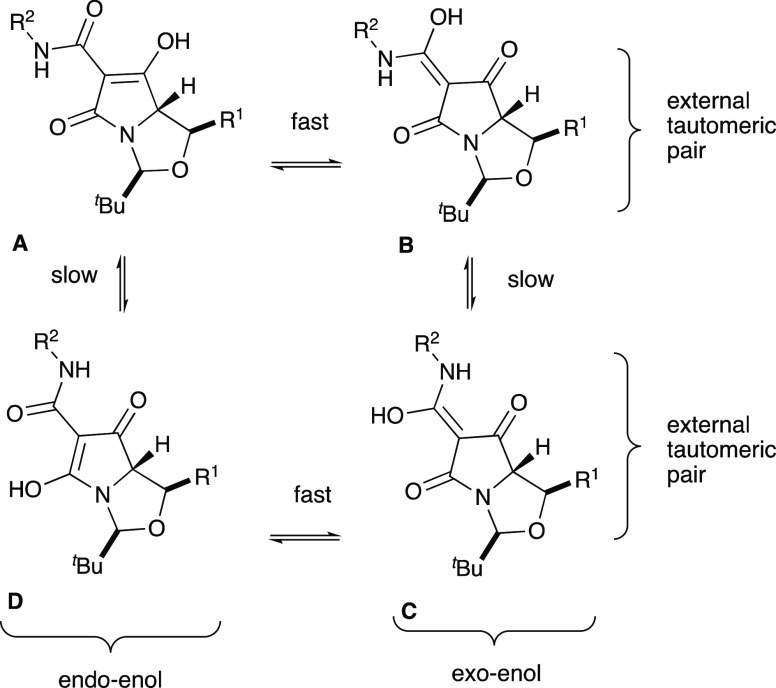
Tautomeric equilibria in tetramates.

The chemoselective Dieckmann cyclization of malonamides **9a–e** fortuitously established a C7-ethyl ester moiety in tetramates **10a–e**, which allowed for direct transamination using aliphatic or aromatic amines to form C7-carboxamides.^17^ Thus, 28 C7-carboxamides **12a–w, 13a–b, 20, 21,** and **22** were synthesized using the appropriate amine in yields ranging between 7 and 70% (Scheme 2 and Table S5). Interestingly, these tetramates were far more stable than their ethyl ester precursors **10a–e**, fully surviving chromatographic purification and storage in CDCl_3_ solution for many months. However, upon chromatography on silica gel, metal chelate formation was rapid, readily seen in the NMR spectrum, where broad peaks were observed; earlier work has shown that these are likely to be principally divalent metal cations, including Mg^2+^ and Ca^2+^, along with monovalent cations, such as Na^+^ and K^+^.^17^ For example, in the case of compound **12a** (Figure S5), the NMR peaks for H4 and H5 were extremely broad after chromatographic purification, consistent with their proximity to the metal chelation site, whereas the ^*t*^Bu, aromatic, and adamantyl groups were relatively sharper, being further away from the metal chelation site. However, when metal-chelated C7-carboxamides were washed with 10% citric acid solution, the NMR peaks significantly sharpened. The fact that only such a mild acid wash was needed, in comparison to the reported 2M HCl (aq) which is usually required, suggested that the C4-aromatic substituents exerted steric disruption of the metal chelation across the tricarbonyl core.^17^ Additionally, C7-carboxamides were found to exist as a mixture of tautomeric pairs AB/CD as observed by NMR spectroscopy (Figure 3). Although the internal tautomeric pairs (A and B; C and D) cannot be readily distinguished by NMR spectroscopy and are generally observed as an averaged signal, the external tautomeric pairs (AB/CD) have distinct ^13^C NMR chemical shift differences at C6, C8, and C9, enabling the different tautomeric forms to be distinguished readily by NMR studies; this is consistent with an earlier report by Panduwawala et al. in related systems.^17^ The major tautomeric form of C7-carboxamidotetramic acids **12a–w**, **13a–b**, **20**, **21**, and **22** was Type **A**, based on the similar ^13^C NMR chemical shift values of C6, C8, and C9 to that reported.^33.^ The ^13^C NMR chemical shift values for C6, C8, and C9 tended to be more downfield for the minor tautomeric pair, **CD**, than for the major tautomeric pair AB (Table S5).

nOe and NOESY studies were conducted to determine the relative stereochemistry of these C7-carboxamidotetramic acids, giving the typical correlations indicated (Scheme 2 and Figure S1), and it was clear that there appeared to be no epimerization after aminolysis at any of the stereogenic centers within the bicyclic molecule. Furthermore, a single crystal X-ray diffraction structure of compound **12a** confirmed the relative stereochemistry (Figure 2).^16^ Since the NMR-chemical shifts of **12a** were consistent with all of the C7-carboxamide series (Table S5), it was assumed that all C7-carboxamidotetramic acids had the same relative stereochemistry.

### Antibacterial Activity

With access to these derivatives routinely available, an assessment of their antibacterial activity was made by measuring minimum inhibitory concentration (MIC) values (Table S6) against two example organisms, methicillin-resistant *Staphylococcus aureus* (MRSA) and *E. coli*. C7-Ethyl ester tetramates derived from the methyl esters of l-serine, l-threonine, and l-cysteine or unfunctionalized/simple tetramic acids have been observed to have no intrinsic antibacterial activity against Gram (+) and Gram (−) bacteria, with MIC values in most cases exceeding 250 μg/mL.^5^ In keeping with these observations, tetramates **10a,b** showed no activity against MRSA and *E. coli*, but of interest was some modest activity for compounds **10c–e** against MRSA with MIC values ranging between 15.6 and 31.3 μg/mL. By contrast, upon conversion of the C7-ethyl ester to a C7-aromatic carboxamide, good to excellent antibacterial activity was observed against MRSA, with activity ranging between 0.49 and 15.6 μg/mL, although no activity was observed against *E. coli* (Table S6). Although **12b** exhibited only modest antibacterial activity (MIC = 7.80 μg/mL), additional substituents around the aromatic carboxamide gave much better activities, for example, *p*-Br **12c** and *p*-N O _2_**12d** had a MIC value of 3.90 μg/mL; *p*-CF_3_**12e** and *p*-cyclohexyl **12s** had MIC = 1.00 μg/mL; and *p*-hexyl **12m** had MIC = 0.49 μg/mL, suggesting that a hydrophobic tail was important. *Ortho*-and *para*-Substituted aromatic rings **12g–12l** were well-tolerated, with activity ranging between 0.49 and 3.90 μg/mL, and fluoro-substituted **12i** and **12l** had high activities. In the case of aliphatic carboxamides, more hydrophobic side chains at the C7-position gave better activity against MRSA, and dodecyl **12r** and nonyl **12q** were far more active (MIC = 0.49 μg/mL) than hexyl **12p** (MIC = 3.90 μg/mL). While *t*-Bu **12v** had comparable activity with the hexyl **12p**, benzyl **12u** only exhibited modest bioactivity (15.60 μg/mL). By contrast, a more polar substituent at the C7-position (pyran **12t)** compromised the antibacterial activity (125 μg/mL). This was consistent with reports by Panduwawala et al.^17^ and Jeong et al.^18^ that polar substituents at the C7-position significantly worsened antibacterial activity for bicyclic tetramates. An exception was **12d**, which had good bioactivity. Although cyclohexyl **12n** and cycloheptyl **12o** had a MIC value of 1.00 μg/mL, they were not as potent as the adamantyl substituent **12a** (MIC = 0.25 μg/mL), this being the most potent compound. Since carboxamide **12a** was the most active compound, the C4-aromatic substituents in the tetramate core were varied; for **13a** and **15**, both the *p*-Me substituent (MIC = 3.90 μg/mL) and *p*-Cl substituent (MIC = 7.80 μg/mL) had a detrimental impact on the antibacterial activity, although *p*-F **14**and *p*-Br **16** (1.00 μg/mL and 0.49 μg/mL, respectively) only had a slightly reduced bioactivity in comparison to **12a**. Compound **13b** had the same activity as **12g** at 1.00 μg/mL. Overall, increasing lipophilicity corresponded to higher potency against MRSA.

**Figure 4:**
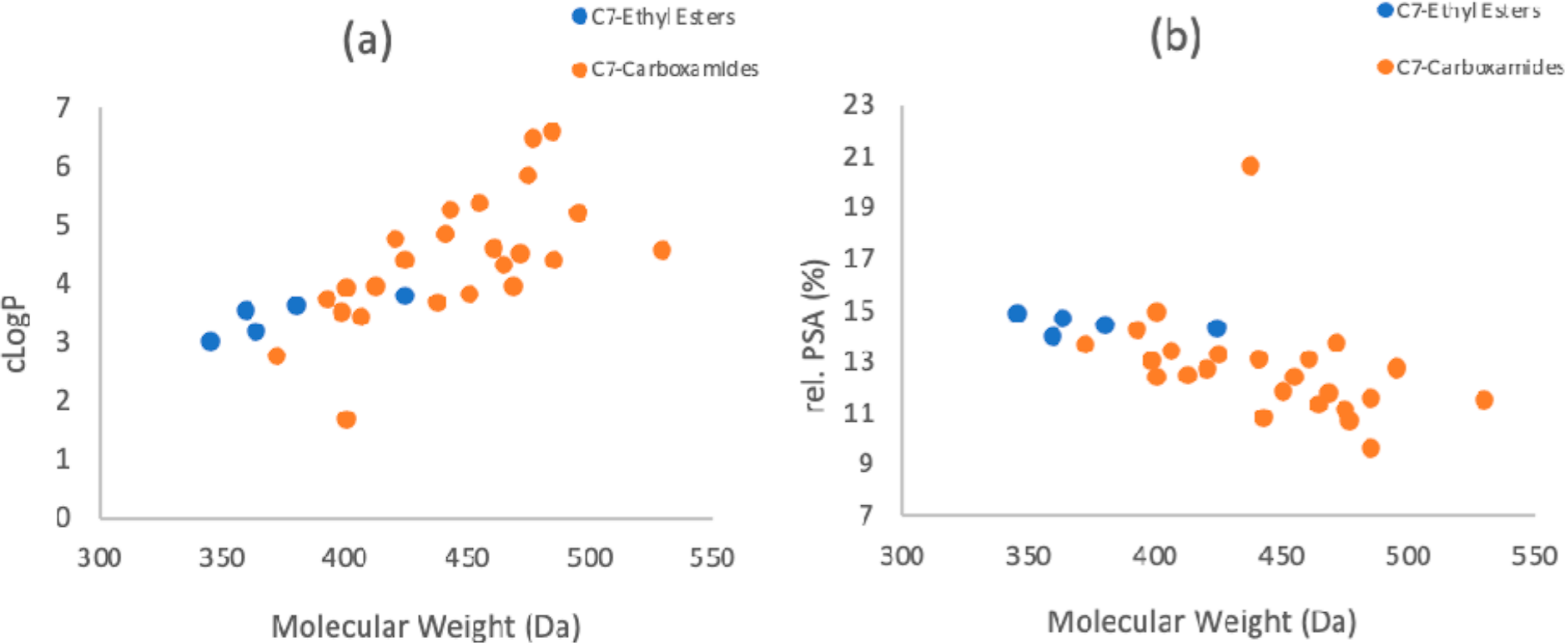
Physicochemical property space of tetramate esters **10** and amides **12–16**; **(a)** cLog*P* plotted against *M*_w_ and **(b)** rel. PSA plotted against *M*_w_ (cLog*P*, MSA, and PSA were calculated using Marvin (19.9.0), 2019, ChemAxon).

**Figure 5:**
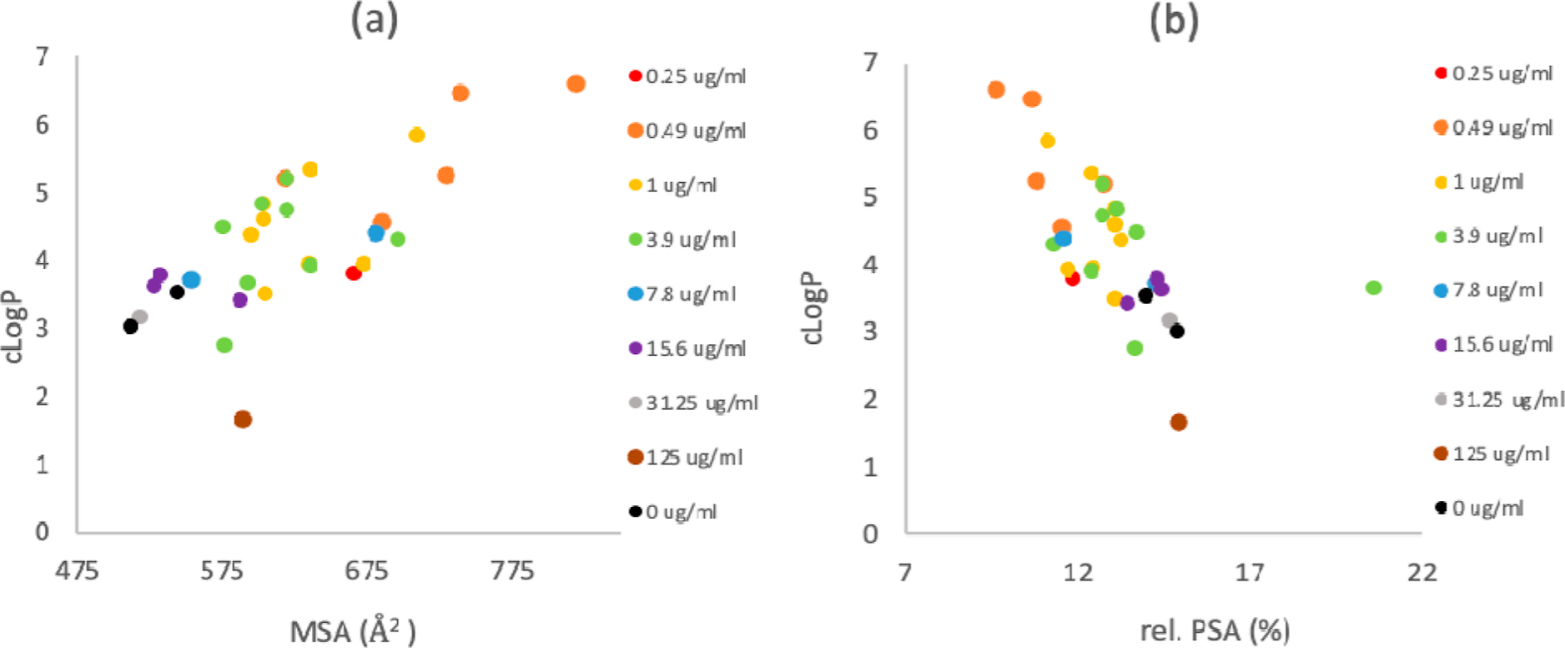
Correlation of potency of tetramates against MRSA with physicochemical properties; **(a)** cLog*P* against MSA and **(b)** cLog*P* plotted against rel. PSA (cLog*P*, MSA, and PSA were calculated using Marvin (19.9.0), 2019, ChemAxon).

A consideration of the physicochemical property space of the bicyclic tetramate esters **10** and amides **12–16** (Table S6) shows that the library is characterized by 345 < M_w_ < 529 Da 1.7 < cLog*P* < 6.6; 9.6 < rel-PSA < 20.6%, and many of the bioactive compounds are within the scope of Ro5 (Figure 4). However, the most potent compounds (1 μg/mL or less) occupied a much narrower region of chemical space, with cLog*P* values ranging between 3.5 < cLog*P* < 6.6 and MSA values ranging between 594 < MSA <818 Å^2^ with a cutoff point at which an increase in rel. PSA >13% led to a reduction in potency (MIC >1 μg/mL) (Figure 5).

The selectivity of these compounds for activity against prokaryotic cells over mammalian cells was analyzed by considering their cytotoxicity against Hela, heK-293, CaCo, and MDCK human cell lines, allowing estimation of the therapeutic ratio [EC_50__Hela cell line_/MIC_(_*S. aureus*_)_] (Table 3). While C7-ethyl ester tetramates **10c-e** had a therapeutic ratio of between 1 and 2, mainly due to their low potency against MRSA, carboxamide **12a** was 26-fold more selective for bacteria over the Hela cell line, showing an excellent compromise between high potency and low toxicity (Table S4), and better than the desired >10-fold higher antibacterial activity over cytotoxicity.^19^

**Table 3:** EC_50_ Values Against Various Cell Lines and the Therapeutic Ratio for Selectivity for Bacteria Over Human Cell Lines

	cell lines EC_50_ μg/mL)					
compound	Hela	heK-293	CaCo	MDCK	MIC(*S. aureus*)(μg/mL)	therapeutic ratio (using Hela cell lines)
**10c**	31.3	62.5	62.5	62.5	31.3	1
**10d**	31.3	31.3	31.3	62.5	15.6	2
**10e**	31.3	62.5	62.5	62.5	15.6	2
**12a**	7.8	31.3	15.6	7.8	0.3	26

However, when carboxamides **12–16** were tested in the presence of blood, the antibacterial activity for most of these compounds significantly reduced, with 15 out of 26 carboxamides only showing activity at concentrations >124 μg/mL. Nonetheless, 11 carboxamides were active at concentrations between 31.25 and 62.5μg/mL, an important outcome, as earlier findings had demonstrated that C3/C7-acyl/carboxamidotetramates in most cases suffered from either partial or complete loss of efficacy under similar conditions.^5^ As tetramic acids are well-known metal chelators,^1,20^ of interest was to investigate whether this loss of activity was due to metal chelation or plasma protein binding (PPB), specifically with human serum albumin (HSA).^17,18^ To answer this question, bicyclic tetramate **12a** was tested against MRSA using a broth dilution assay, under standard conditions and with the addition of similar metal cations (Fe, Ca, Mg, and Zn) with concentration as that found in blood, respectively. The activity was found to be slightly improved for the latter {0.49 vs 0.24 [MIC (μg/mL) against MRSA]}, suggesting that the weaker metal chelating properties of these aryl-substituted tetramates discussed above may be important for in vivo activity in therapeutic application. Bicyclic tetramate **12a** was also tested against MRSA using a broth dilution assay, under standard conditions but in the presence of one-third the serum albumin concentration as that found in human blood. The activity dropped 16-fold **{**7.81 [MIC (μg/mL) against MRSA]}, and this outcome is similar to the work by Panduwawala et al. and Jeong et al.,^17,18^ who had also earlier demonstrated that carboxamidotetramic acids lost antibacterial activity in the presence of HSA. A review has shown that PPB strongly correlates with increased hydrophobicity, and this might point to a problem with the tetramate class, which clearly depends upon hydrophobicity for their antibacterial activity, as noted above.^21^

## Conclusions

A general route which provides direct access to substituted bicyclic tetramates, making use of Dieckmann cyclization of oxazolidines derived from *threo*-arylserines, has been developed; this required the development of a diastereoselective route to β-hydroxy-β-aryl-α-amino acids, achieved by an aldol-like reaction of glycine with substituted benzaldehydes under alkaline conditions. The reactions of bicyclic lactams^22−24^ are governed by several phenomena including steric,^25,26^ electronic,^27^ and hydrogen bonding^28^ effects, and the work described here shows that ring substituents may critically influence reaction outcomes leading to such functionally dense bicyclic ring systems, likely to be of relevance to the recently identified clausenamide group of natural products.^29^ This work expands opportunities for scaffold hopping from tetramates to pyroglutamates, by providing a general route to highly substituted systems.^30,31^ For the synthesized *N,O*-bicyclic tetramates, it has been demonstrated that C7-ethyl ester tetramates **10a–e** showed either no antibacterial activity or only weak antibacterial activity against MRSA. However, C7-carboxamides **12–22** showed much improved antibacterial activity with large hydrophobic carboxamide pendants at the C7-position giving the most potent antibacterial activity. However, the use of a more polar substituent at the C7-position compromised bioactivity. All the compounds screened showed no antibacterial activity against *E. coli*. The C7-adamantylcarboxamidotetramate **12a** was very selective for bacteria over mammalian cells. Many of the compounds synthesized were Ro5-compliant and occupied a distinct chemical property space different from that occupied by other known antibiotics, with the most potent compounds having 399 < *M*_w_ < 530 Da; 3.5 < *c*Log*P* < 6.6; 594 < MSA <818 Å^2^ 9.6 < rel. PSA <13.3%. Unfortunately, MIC values were shifted to higher concentrations when tested in the presence of HSA or blood, consistent with a plasma protein binding (PPB) effect, even though metal chelation was reduced in the more densely functionalized tetramate system. Considering the physicochemical properties, potency, and toxicity of these compounds, the most promising candidates for further optimization are **12a** and **12i**, along with **22** even though it was of high molecular weight (M_w_ > 500 g/mol).

## Supporting Information Available

Experimental details; ^1^H and ^13^C NMR spectra; DFT analysis; X-ray crystallographic data; and bioassay data (PDF).
