# Supplementary material for: Tetramate Derivatives by Chemoselective Dieckmann Ring Closure of threo-Phenylserines and Their Antibacterial Activity
Source: J Org Chem. 2022 Sep 2;87(18):12240–9. doi: 10.1021/acs.joc.2c01382 (PMC9486951; doi:10.1021/acs.joc.2c01382)
Supplement: Supplementary file 1 — jo2c01382_si_001.pdf [file jo2c01382_si_001.pdf]

Supporting Information

Tetramate Derivatives by Chemoselective Dieckmann Ring Closure of *threo*-Phenylserines, and their Antibacterial Activity

Liban Saney,[†] Kirsten E. Christensen,[†] Xiang Li,[†,+] Miroslav Genov,[‡] Alexander Pretsch,[‡] Dagmar Pretsch,[‡] and Mark G. Moloney*[,†,#]

[†]The Department of Chemistry, Chemistry Research Laboratory, University of Oxford, 12 Mansfield Road, Oxford. OX1 3TA

[+]Department of Pharmaceutical Engineering, China Pharmaceutical University, Nanjing, 211198, P. R. China.

[#]Oxford Suzhou Centre for Advanced Research, Building A, 388 Ruo Shui Road, Suzhou Industrial Park, Jiangsu, 215123, P.R.China.

[‡]Oxford Antibiotic Group, The Oxford Science Park, Magdalen Centre, Oxford OX4 4GA, UK.

mark.moloney@chem.ox.ac.uk

Table of Contents

Figure S1 NOE and NOESY Correlations S-3,4

Figure S2 1H-NMR peaks of malonamides **8b** and **9b** S-5

Figure S3 ^{1}H-NMR peaks of malonamide **9b** S-6

Figure S4 DFT calculated energy profile for the synthesis of tetramate **10a** S-7

Figure S5 ^{1}H-NMR peaks of C7-carboxamide **12a** S-8

Table S1: Comparison of literature ^{1}H-NMR data with oxazolidinone **6**. S-9

Table S2. Key chemical shifts (δ), coupling constants (*J*), diastereomeric ratio (dr) and conversion of oxazolidines **7a-e**. S-9

Table S3. Key chemical shifts (δ), coupling constants (*J*), diastereomeric ratio (dr) and yields of malonamides **8a-f** and **9a-e**. S-9

Table S4. Key chemical shifts (δ), coupling constants (*J*) and yields of tetramates **10a-e**. S-10

Table S5: Key ^{1}H and ^{13}C-NMR chemical shifts (δ) and coupling constants (*J*) of the major and minor tautomeric pairs AB:CD of C7-carboxamidotetramic acids and MIC values S-10

Table S6: MIC values and Physicochemical properties of bicyclic tetramates (cLogP, MSA, PSA, H-donors and H-acceptors) S-13
DFT Calculation Data S-18
Experimental procedures and data S-41
X-ray Crystallographic Data S-76
References S-80
NMR Spectra S-81

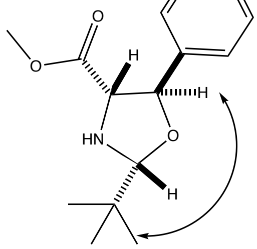

2,5-*cis*- 7a

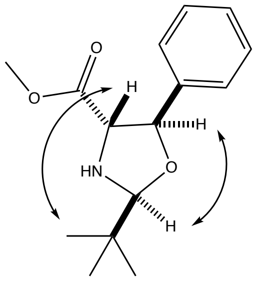

2,5-*trans*-7a

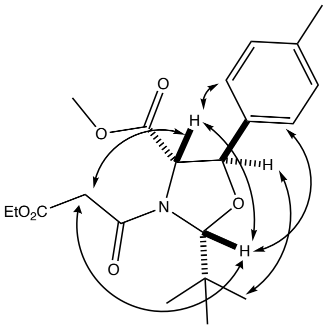

8b

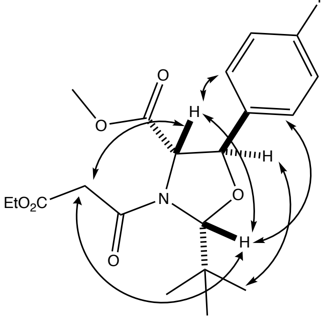

8c

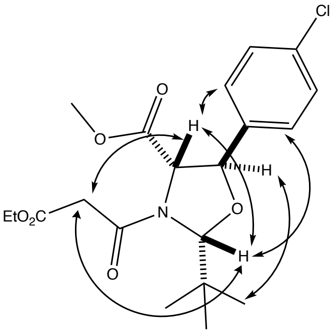

8d

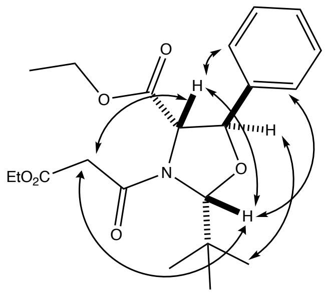

8f

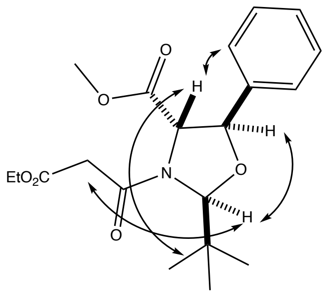

9a

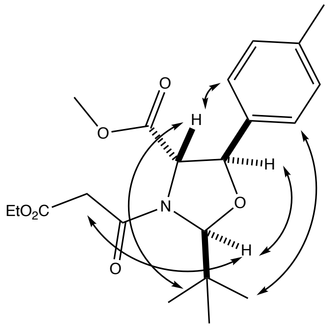

9b

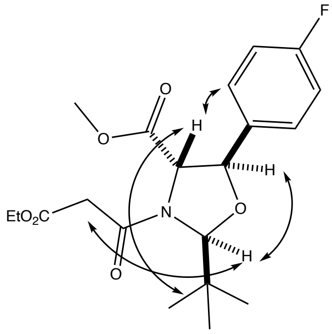

9c

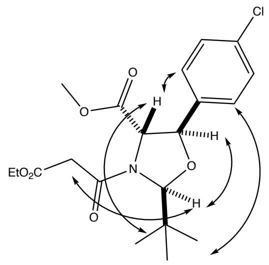
9d

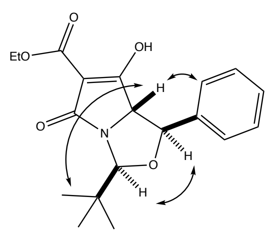
10a

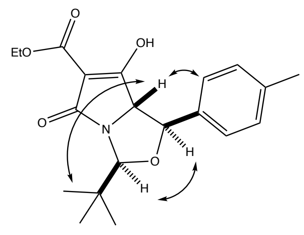
10b

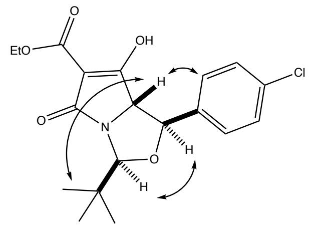
10d

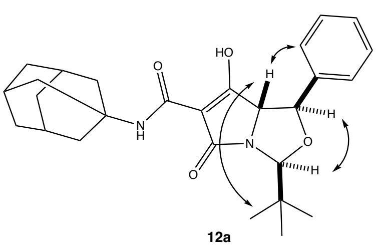
12a

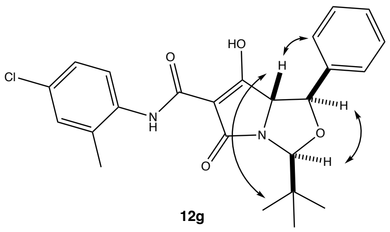
12g

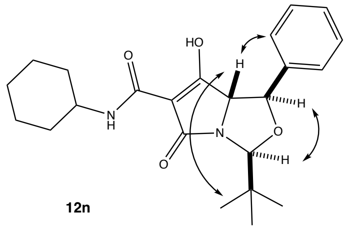
12n

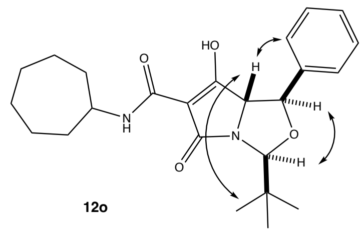
12o

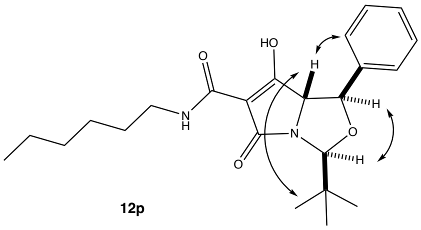
12p

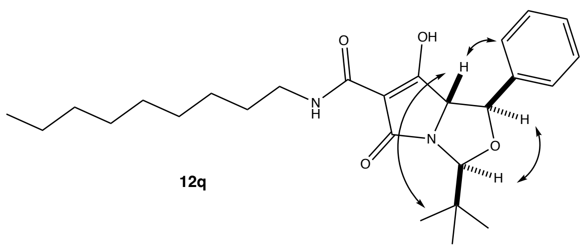
12q

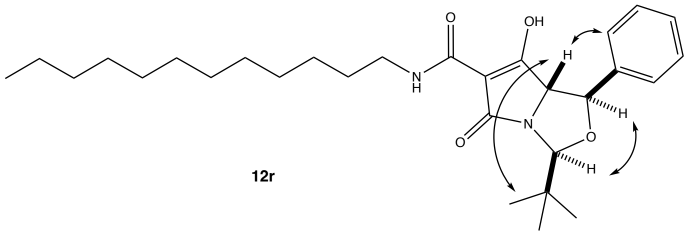
12r

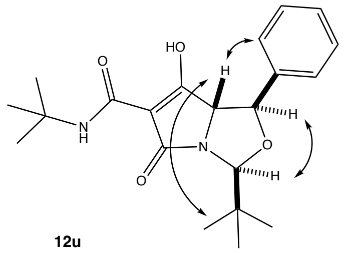
12u

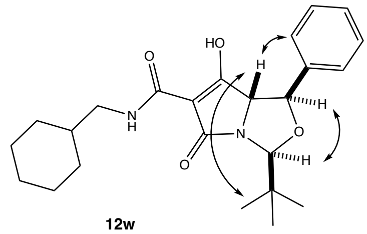
12w

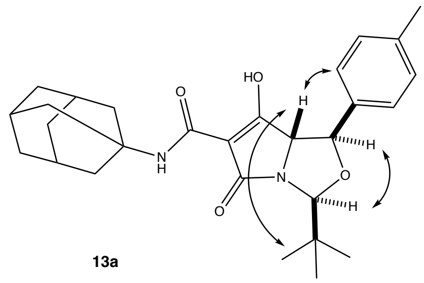
13a

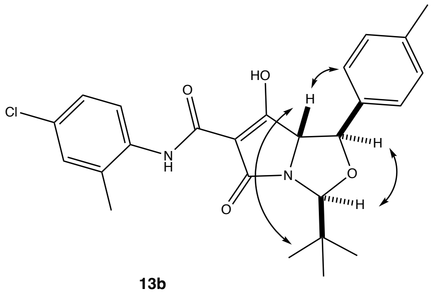
13b

Figure S1: nOe and NOESY interactions

8b [a]

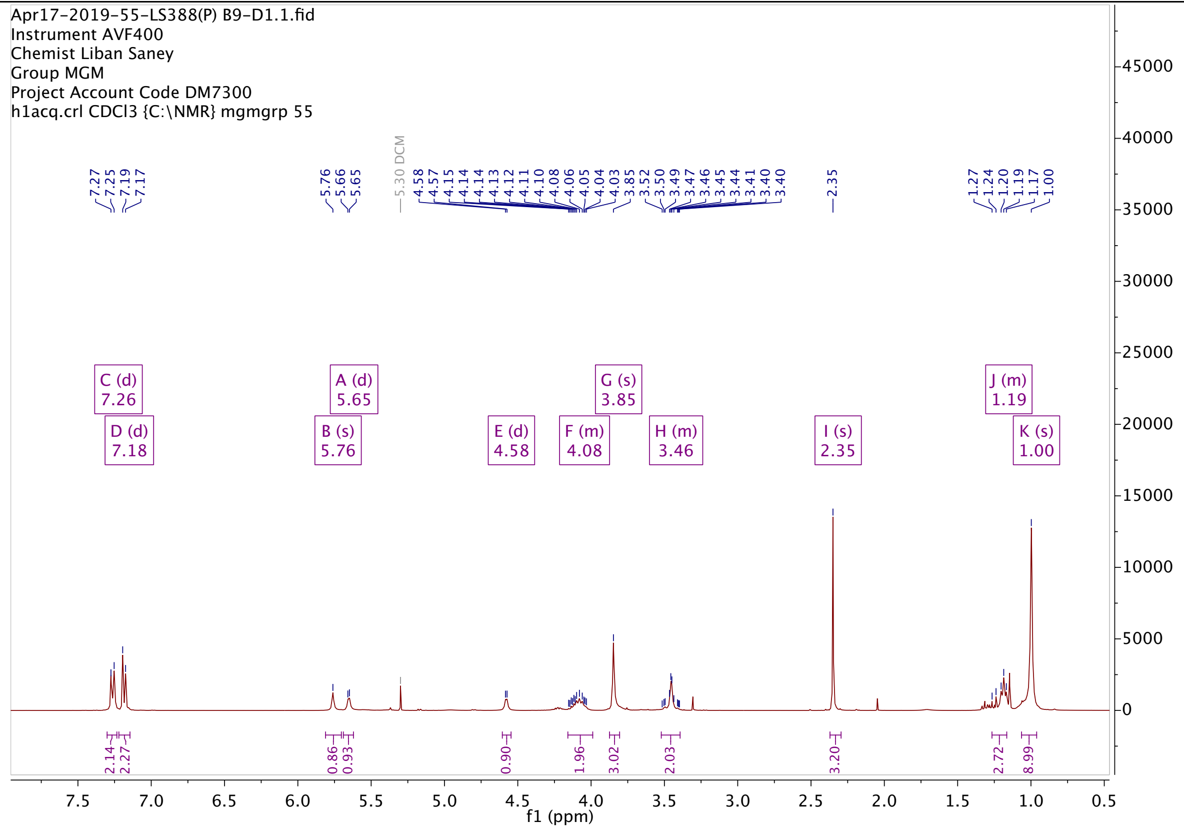

9b [b]

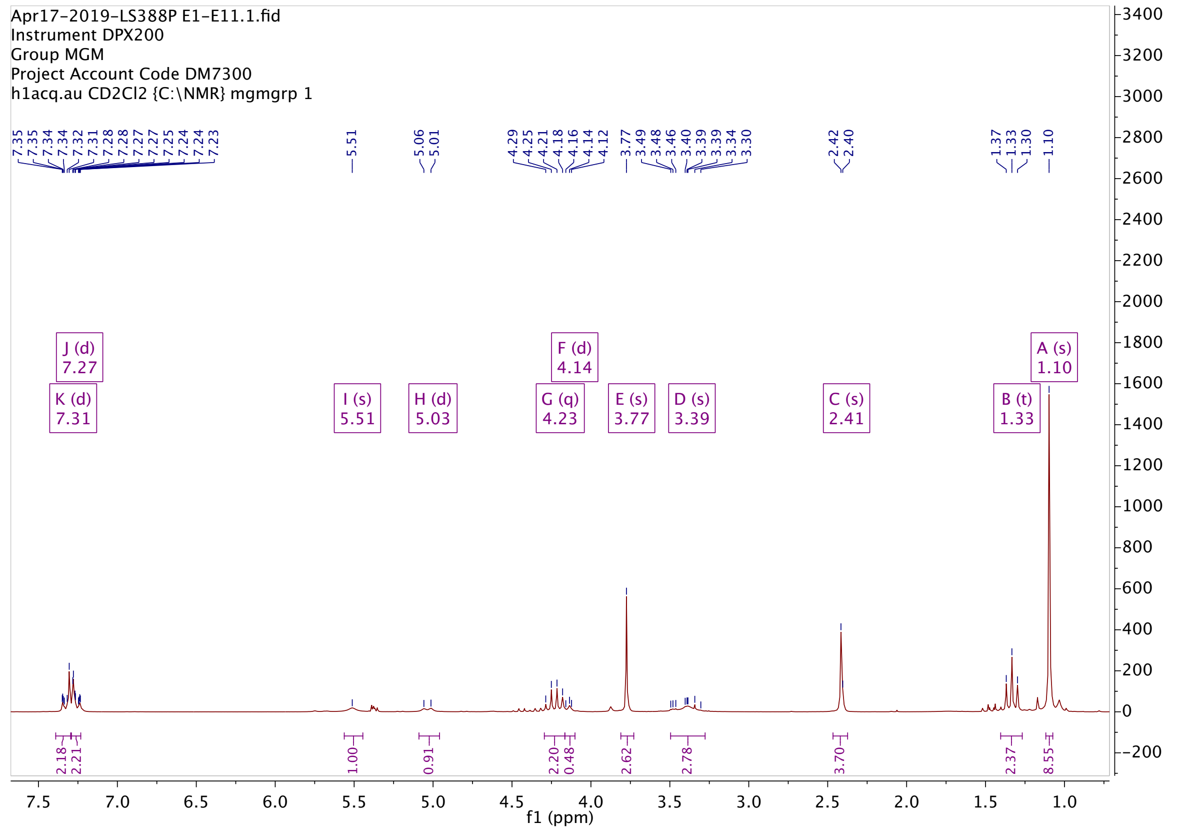

Figure S2. ^{1}H-NMR peaks of malonamides **8b** and **9b** at room temperature; [a] $CDCl_3$ solution, 400 MHz; [b] CD_2Cl_2 solution, 200 MHz.

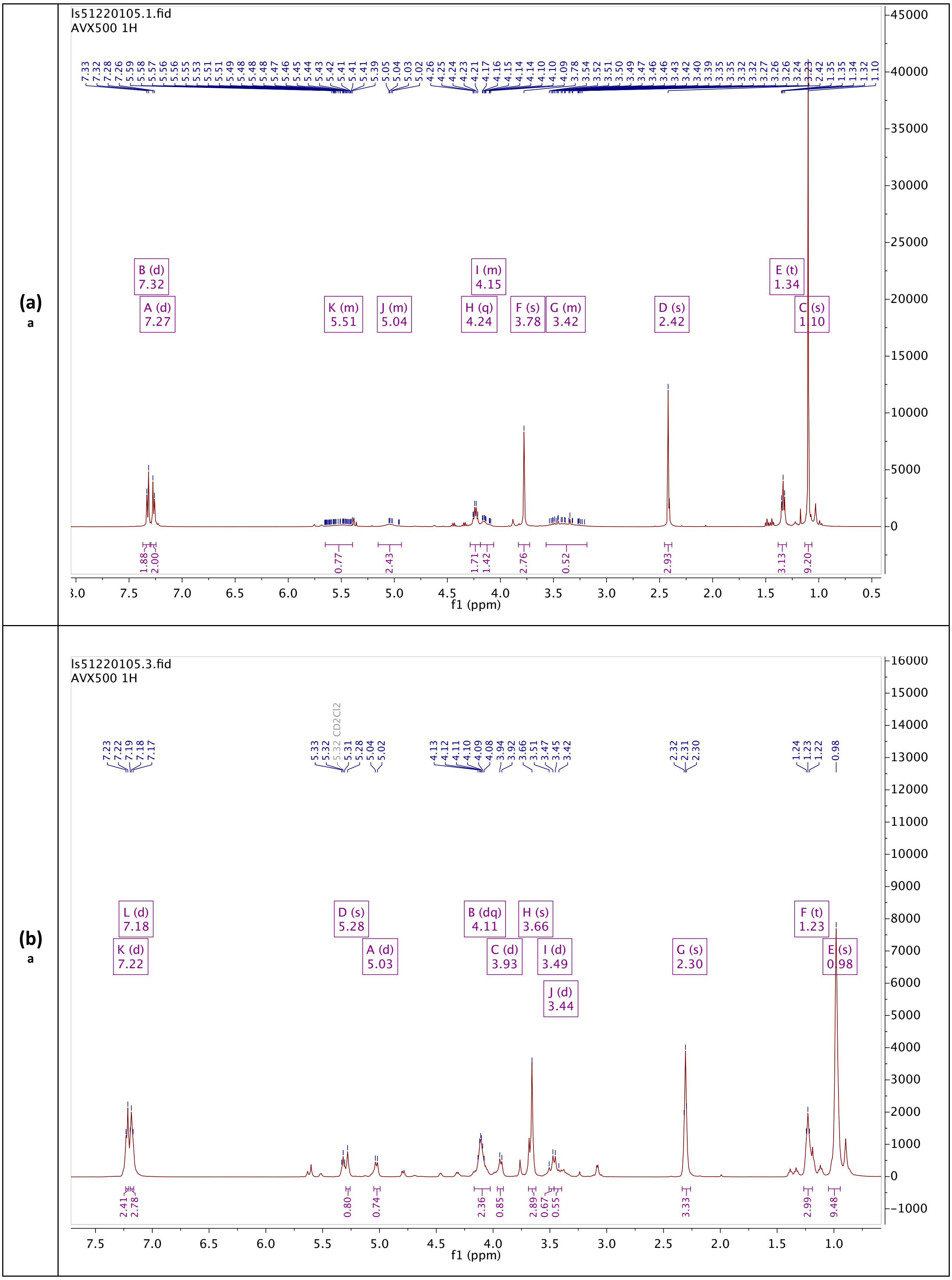

Figure S3. ^{1}H-NMR peaks of malonamide **9b** at (a) room temperature and at (b) 203 K; ^{a} CD_2Cl_2 solution, 500 MHz.

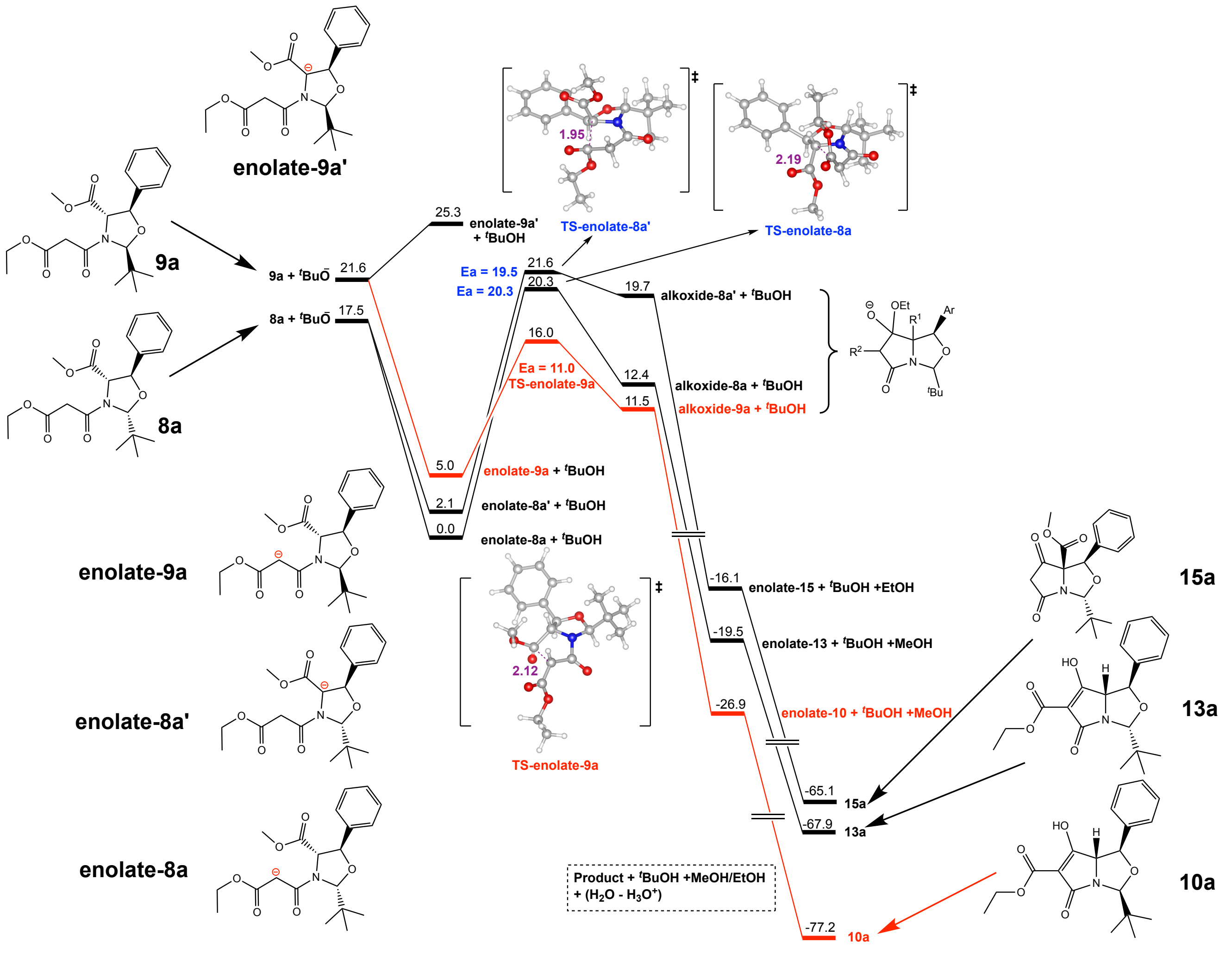

Figure S4. DFT calculated energy profile for the synthesis of tetramate **10a** from malonamides **8a** and **9a** using a CentOX Linux 7 operating system; where a Gaussian 16 (G16RevA.03) software was run in parallel over 32 CPU cores, using the B3LYP/6-311++g(d,p) level of theory with SMD solvation (THF). Relative electronic energy (kJ/mol) of all structures are shown.

(a)[a]

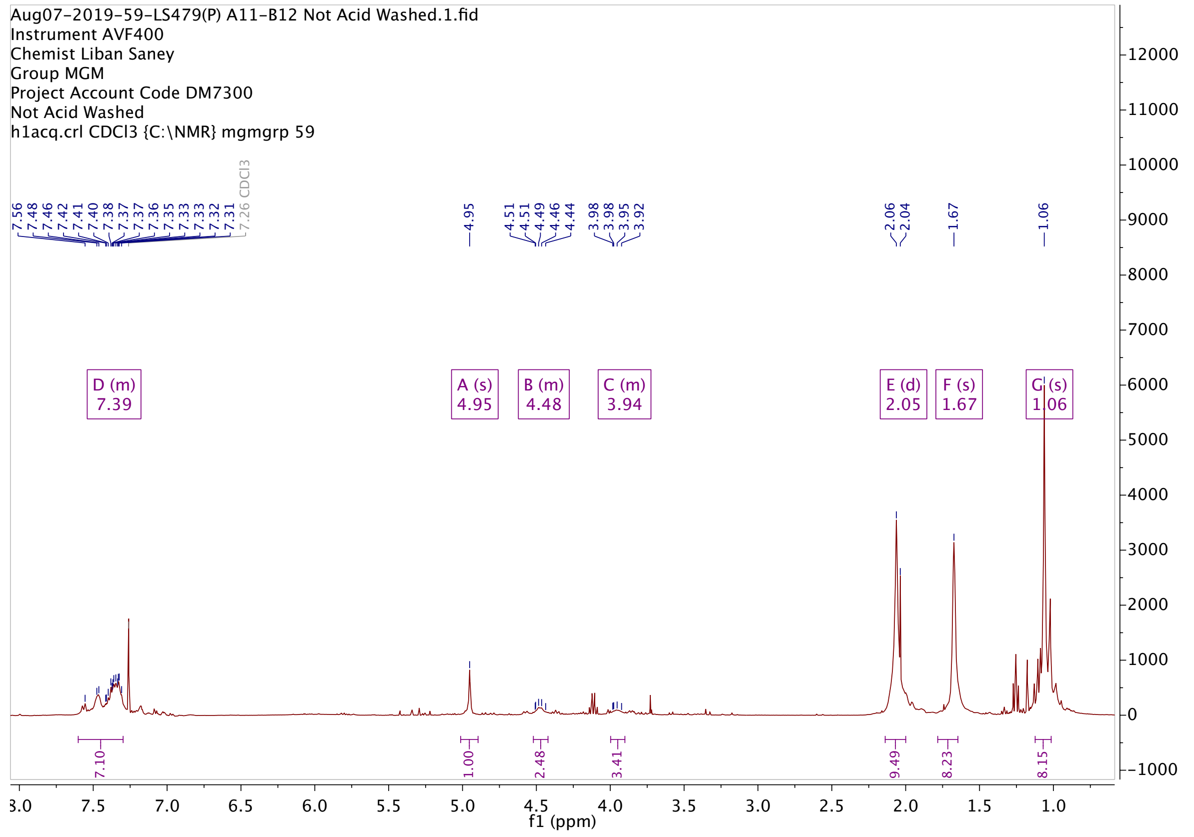

(b)[a]

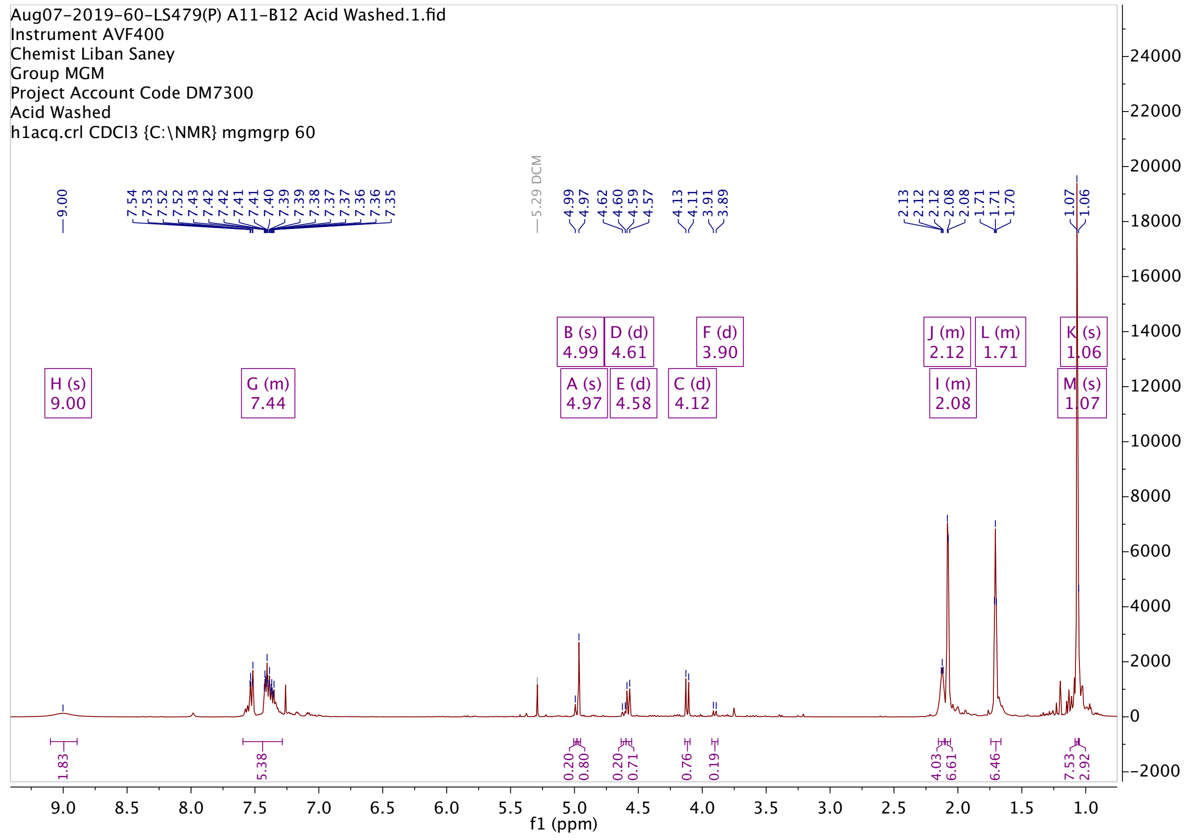

Figure S5. ^{1}H-NMR peaks of C7-carboxamide **12a** post-chromatographic purification, before (a) acid wash and (b) after 10% citric acid wash; [a] $CDCl_3$ solution, 400 MHz.

Table S1. Comparison of literature ^{1}H-NMR data with oxazolidinone **6**.

Compound	δ H4 (ppm)	δ H5 (ppm)	J_{H4-H5} (Hz)
**83a/6** [a]	5.63	4.31	5.0
Literature data (Hameršak, Z.; Šepac, D.; Žiher, D.; Šunjić, V., *Synthesis* **2003,** *3*, 375-382) for *threo*-derived oxazolidinone [b]	5.65	4.33	5.0
Literature data (Seashore-Ludlow, B.; Villo, P.; Hacker, C.; Somfai, P., *Org. Lett.* **2010,** *12*, 5274-5277) for *erythro*-derived oxazolidinone [c]	5.84	4.67	9.0

[a] $CDCl_3$ solvent, 400 MHz; [b] $CDCl_3$ solvent, 400 MHz; [c] $CDCl_3$ solvent, 500 MHz.

Table S2. Key chemical shifts (δ), coupling constants (*J*), diastereomeric ratio (dr) and conversion of oxazolidines **7a-e**.

	2,5-*trans* oxazolidine				2,5-*cis* oxazolidine					
Compound	δ H2 (ppm) [a]	δ H4 (ppm) [a]	δ H5 (ppm) [a]	J_{H4-H5} (Hz) [a]	δ H2 (ppm) [a]	δ H4 (ppm) [a]	δ H5 (ppm) [a]	J_{H4-H5} (Hz) [a]	dr at the C2 position [b]	Conversion (%) [c]
**7a**	4.42	4.74	3.63	6.6	4.42	4.67	3.77	7.0	1:1.1	82
**7b**	4.41	4.69	3.61	6.7	4.41	4.63	3.77	7.0	1:1.1	81
**7c**	4.41	4.70	3.58	6.7	4.40	4.62	3.70	7.0	1:1.1	70
**7d**	4.41	4.70	3.58	6.6	4.39	4.62	3.71	7.0	1:1.2	62
**7e**	4.41	4.69	3.58	6.6	4.38	4.60	3.71	6.9	1:1.2	69

[a] $CDCl_3$ solvent, 400 MHz; [b] Determined from ^{1}H-NMR studies of the crude material; [c] Yields include both diastereomers.

Table S3. Key chemical shifts (δ), coupling constants (*J*), diastereomeric ratio (dr) and yields of malonamides **8a-f** and **9a-e**.

Compound	δ H2 (ppm) [a]	δ H4 (ppm) [a]	δ H5 (ppm) [a]	J_{H4-H5} (Hz) [a]	Compound	δ H2 (ppm) [b]	δ H4 (ppm) [b]	δ H5 (ppm) [b]	J_{H4-H5} (Hz) [b]	dr **8** : **9** [c]	Yields (%) [d]
**8a**	5.77	5.69	4.59	4.2	**9a**	5.36	5.14	4.00	9.2	65:35	81
**8b**	5.76	5.65	4.58	4.3	**9b**	5.28	5.03	3.93	9.2	59:41	67
**8c**	5.77	5.66	4.57	4.4	**9c**	5.27	5.05	3.91	9.2	59:41	60
**8d**	5.75	5.68	4.62	4.0	**9d**	5.29	5.07	3.91	9.2	60:40	76
**8e**	5.76	5.66	4.58	4.2	**9e**	5.27	5.04	3.89	9.2	60:40	88
**8f**	5.78	5.67	4.55	4.4	-	-	-	-	-	-	34 [e]

[a] $CDCl_3$ solvent, 400 MHz at room temperature; [b] CD_2Cl_2 solvent, 500 MHz, low temperature VT ^{1}H-NMR; [c] Determined from ^{1}H-NMR studies of the crude material at room temperature; [d] Yields include both diastereomers; [e] Yield is for single diastereomer.

Table S4. Key chemical shifts (δ), coupling constants (*J*) and yields of tetramates **10a-e**.

Compound	δ H2 (ppm) [a]	δ H4 (ppm) [a]	δ H5 (ppm) [a]	J_{H4-H5} (Hz) [a]	Yield (%)
**10a**	4.99	4.53	4.17	8.4	80
**10b**	5.00	4.51	4.18	8.5	79
**10c**	5.00	4.54	4.16	8.4	60
**10d**	5.01	4.54	4.14	8.4	74
**10e**	5.00	4.52	4.13	8.4	78

[a] $CDCl_3$ solvent, 400 MHz.

Table S5: Key ^{1}H and ^{13}C-NMR chemical shifts (δ) and coupling constants (*J*) of the major and minor tautomeric pairs **AB:CD** of C7-carboxamidotetramic acids ($CDCl_3$ solvent, 400 MHz) (see Scheme 2) and MIC values against *S. aureus*; [a] Determined from the H2 signal from the ^{1}H-NMR spectrum.

Compound	R[1]	R[2]	δ (ppm)							Tautomeric ratio [a]	Yield (%)	MIC (μg/mL)
			H2	H4	H5	J_{H4-H5} (Hz)	C6	C8	C9			
**12a**		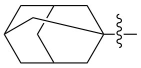	4.97<br>4.99	4.58<br>4.61	4.12<br>3.90	8.6<br>8.9	186.9<br>190.5	175.1<br>181.3	166.3<br>167.1	Major (AB) 80%<br>Minor (CD) 20%	55	0.25
**12b**		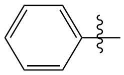	5.02	4.63	4.27	8.5	184.0	174.4	164.1	AB only	42	7.8
**12c**	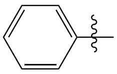	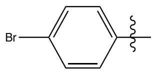	5.01	4.61	4.27	8.5	183.7	174.2	164.0	AB only	42	3.9
**12d**		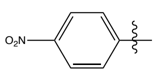	5.02	4.64	4.31	8.5	184.0	174.0	164.3	AB only	15	3.9
**12e**		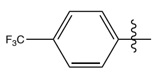	5.02	4.63	4.30	8.5	183.9	174.2	164.3	AB only	38	1.0

**12f**		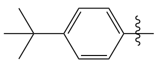	4.90	4.44	4.00	8.5	185.1	175.4	164.1	AB only	27	-
**12g**		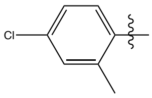	5.03	4.63	4.27	8.5	183.6	174.4	163.9	AB only	40	1.0
**12h**		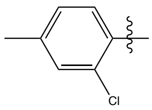	5.06	4.63	4.27	8.4	183.9	174.0	164.2	AB only	44	3.9
**12i**		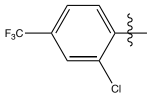	5.07	4.64	4.31	8.5	184.0	173.8	164.5	AB only	21	0.49
**12j**		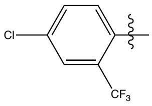	5.06	4.64	4.28	8.5	183.8	173.7	164.5	AB only	43	3.9
**12k**		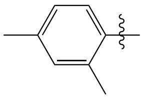	5.05	4.65	4.27	8.5	184.2	174.5	164.2	AB only	41	3.9
**12l**		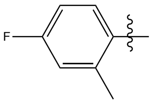	5.04	4.64	4.27	8.5	183.8	174.4	164.0	AB only	51	1.0
**12m**		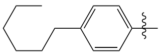	5.02	4.62	4.26	8.5	184.1	174.4	164.0	AB only	18	0.49
**12n**		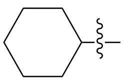	4.97<br>4.99	4.58<br>4.61	4.15<br>3.90	8.6<br>8.9	186.1<br>190.6	174.9<br>181.0	165.5<br>166.0	Major (AB) 82%<br>Minor (CD) 18%	45	1.0

**12o**		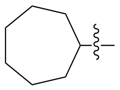	4.97<br>4.99	4.58<br>4.61	4.15<br>3.90	8.5<br>8.8	186.1<br>190.6	174.9<br>181.0	165.2<br>165.7	Major (AB) 83%<br>Minor (CD) 17%	24	1.0
**12p**		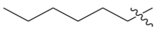	4.97<br>5.00	4.58<br>4.61	4.16<br>3.90	8.5<br>8.9	185.6<br>188.4	174.8<br>181.0	166.3<br>167.0	Major (AB) 82%<br>Minor (CD) 18%	19	3.9
**12q**		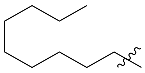	4.97	4.58	4.17	8.6	185.6	174.8	166.3	AB only	40	0.49
**12r**		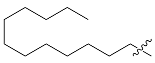	4.97<br>5.00	4.58<br>4.60	4.16<br>3.90	8.6<br>8.9	185.6<br>190.7	174.8<br>180.4	166.3<br>167.0	Major (AB) 84%<br>Minor (CD) 16%	57	0.49
**12s**		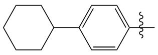	5.02	4.63	4.26	8.5	184.1	174.4	164.0	AB only	25	1.0
**12t**		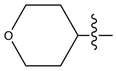	4.97<br>4.99	4.58<br>4.62	4.19<br>3.92	8.6<br>8.9	184.9<br>190.6	174.6<br>181.1	165.6<br>166.4	Major (AB) 88%<br>Minor (CD) 12%	31	125
**12u**		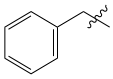	4.96	4.59	4.20	8.5	184.6	174.5	166.0	AB only	33	15.6
**12v**		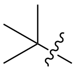	4.96<br>4.99	4.58<br>4.61	4.13<br>3.91	8.6<br>8.8	186.5<br>190.5	175.1<br>181.2	166.3<br>167.2	Major (AB) 82%<br>Minor (CD) 18%	41	3.9
**12w**		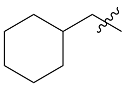	4.98	4.58	4.16	8.5	185.6	174.8	166.3	AB only	37	-

**13a**	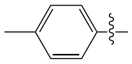	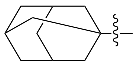	4.94<br>4.97	4.53<br>4.57	4.10<br>3.88	8.6<br>8.8	186.6<br>190.5	175.1<br>181.2	166.2<br>167.0	Major (AB) 80%<br>Minor (CD) 20%	52	3.9
**13b**		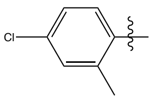	5.01	4.59	4.27	8.5	183.8	174.4	164.1	AB only	7	1.0
**20**	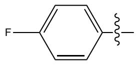	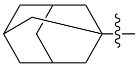	4.94<br>4.97	4.53<br>4.57	4.04<br>3.83	8.9<br>9.3	187.1<br>190.4	175.1<br>181.1	166.2<br>167.0	Major (AB) 78%<br>Minor (CD) 22%	70	1.0
**21**	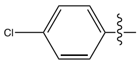	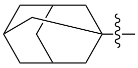	4.96<br>4.99	4.55<br>4.58	4.04<br>3.84	8.6<br>8.9	187.3<br>190.3	175.1<br>181.1	166.3<br>167.0	Major (AB) 76%<br>Minor (CD) 24%	44	7.8
**22**	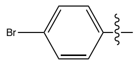	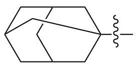	4.95<br>4.98	4.53<br>4.56	4.03<br>3.83	8.6<br>8.9	187.3<br>190.3	175.1<br>181.1	166.3<br>166.9	Major (AB) 77%<br>Minor (CD) 23%	49	0.49

Table S6: Physicochemical properties of bicyclic tetramates (cLogP, MSA, PSA, H-donors and H-acceptors were calculated using Marvin (19.9.0), 2019, ChemAxon).

Compound	Structure	MW (g/mol)	cLog*P*	PSA (Å²)	MSA (Å²)	rel. PSA (%)	H-donor	H-acceptor	Ro5	MIC (µg/ml)
**10a**	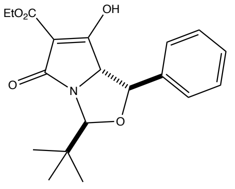	345.40	3.02	76.07	511.17	14.88	1	4	4/4	0.0
**10b**	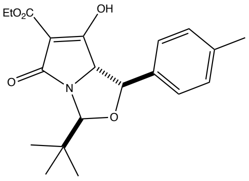	359.42	3.54	76.07	543.26	14.00	1	4	4/4	0.0
**10c**	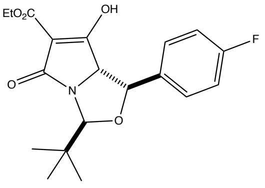	363.39	3.17	76.07	518.16	14.68	1	4	4/4	31.3

**10d**	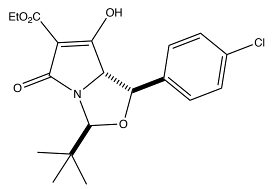	379.84	3.63	76.07	527.59	14.42	1	4	4/4	15.6
**10e**	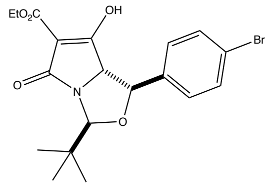	424.29	3.79	76.07	531.82	14.30	1	4	4/4	15.6
**12a**	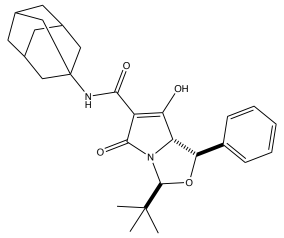	450.58	3.80	78.87	665.18	11.86	2	4	4/4	0.25
**12b**	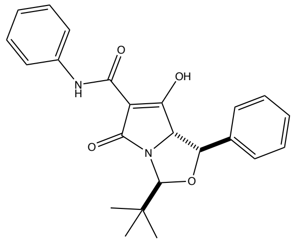	392.46	3.72	78.87	553.87	14.24	2	4	4/4	7.8
**12c**	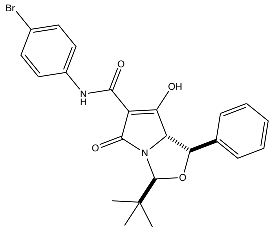	471.35	4.49	78.87	574.55	13.73	2	4	4/4	3.9
**12d**	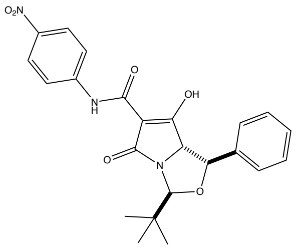	437.45	3.66	122.01	591.78	20.62	2	6	4/4	3.9
**12e**	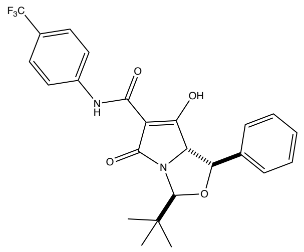	460.45	4.60	78.87	603.57	13.07	2	4	4/4	1.0

**12g**	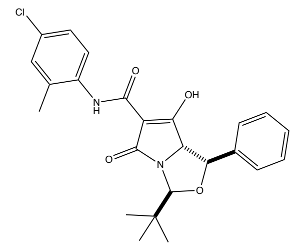	440.92	4.84	78.87	603.49	13.07	2	4	4/4	1.0
**12h**	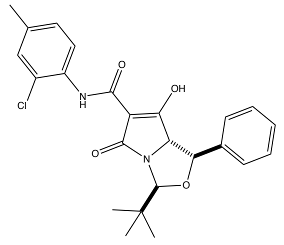	440.92	4.84	78.87	601.62	13.11	2	4	4/4	3.9
**12i**	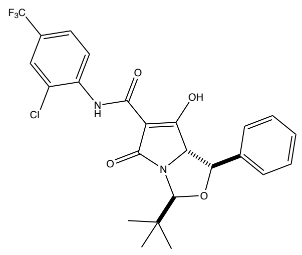	494.90	5.20	78.87	618.62	12.75	2	4	3/4	0.49
**12j**	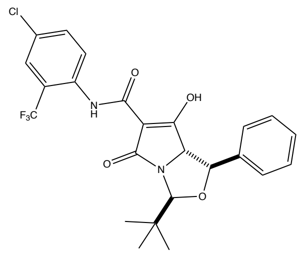	494.90	5.20	78.87	619.04	12.74	2	4	3/4	3.9
**12k**	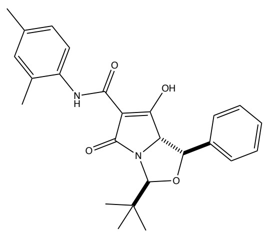	420.51	4.75	78.87	619.60	12.73	2	4	4/4	3.9
**12l**	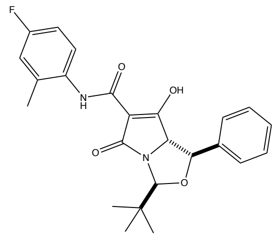	424.47	4.38	78.87	594.16	13.27	2	4	4/4	1.0
**12m**	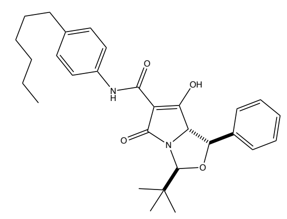	476.62	6.46	78.87	738.95	10.67	2	4	3/4	0.49

**12n**	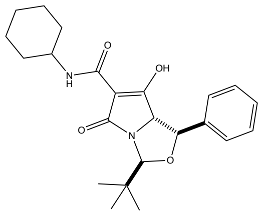	398.50	3.50	78.87	603.76	13.06	2	4	4/4	1.0
**12o**	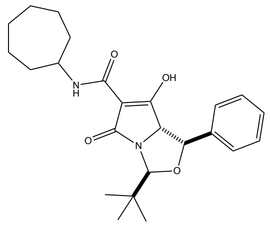	412.53	3.95	78.87	634.11	12.44	2	4	4/4	1.0
**12p**	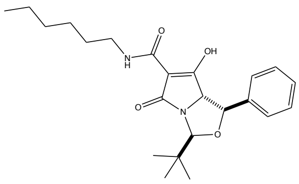	400.52	3.92	78.87	635.67	12.41	2	4	4/4	3.9
**12q**	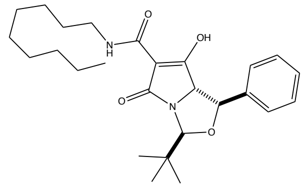	442.60	5.25	78.87	729.45	10.81	2	4	3/4	0.49
**12r**	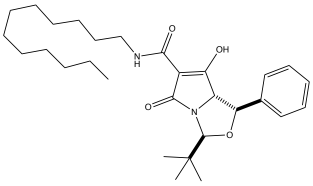	484.68	6.59	78.87	818.41	9.64	2	4	3/4	0.49
**12s**	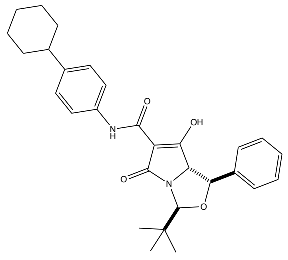	474.60	5.84	78.87	708.23	11.14	2	4	3/4	1.0
**12t**	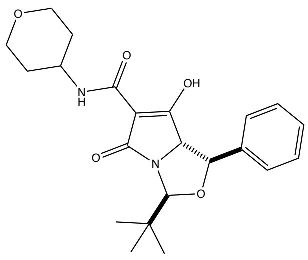	400.48	1.67	88.10	589.42	14.95	2	5	4/4	125
**12u**	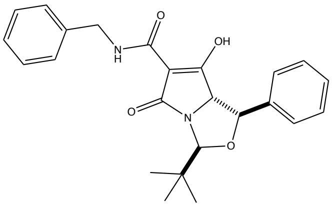	406.48	3.43	78.87	586.97	13.44	2	4	4/4	15.6

**12v**	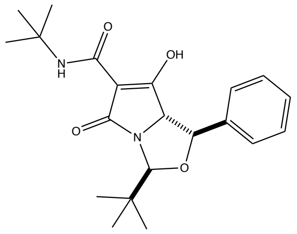	372.47	2.76	78.87	575.98	13.69	2	4	4/4	3.9
**13a**	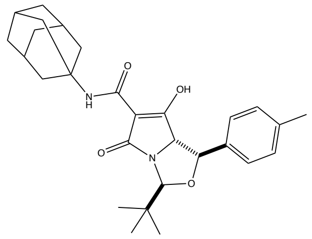	464.61	4.31	78.87	696.09	11.33	2	4	4/4	3.9
**13b**	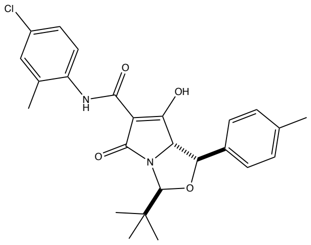	454.95	5.35	78.87	635.74	12.41	2	4	3/4	1.0
**20**	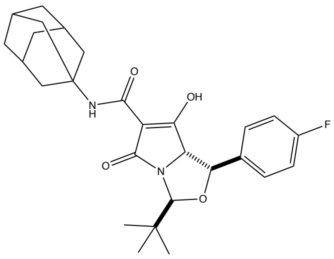	468.57	3.94	78.87	671.84	11.74	2	4	4/4	1.0
**21**	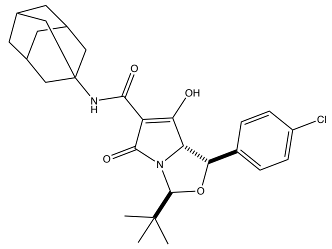	485.02	4.40	78.87	680.88	11.58	2	4	4/4	7.8
**22**	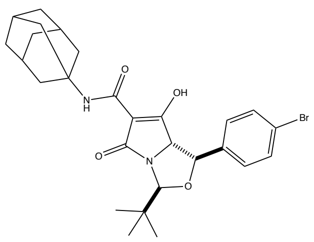	529.48	4.56	78.87	684.78	11.52	2	4	3/4	0.49

Computational Methods - DFT Calculation Data

Calculation level: b3lyp/6-31g(d)//b3lyp/6-311++g(d,p) scrf=(smd,solvent=THF). Calculations were carried out by means of the Gaussian 16 software package at the B3LYP/6-311++G(d,p) level of theory with SMD solvation (THF). All stationary points were characterized by the correct number of imaginary frequencies (1 for transition states, TS, and 0 for minima). Relative electronic energy with zero-point energy correction for the structures have been calculated (Kcal/mol).
The energies in this manuscript are provided as electronic energies. Gibbs free energies were also calculated using the "Thermal Correction to Free Energy" obtained by the frequency analysis instead of the "Zero-point Energy Correction", and the reactivity trends based on the electronic energies are in agreement with the Gibbs free energies, especially in the key condensation process.

9a

C	-0.61140300	-0.16635200	0.75406500
C	-1.25690500	-0.02382200	-0.64503600
C	0.21239700	1.77800200	-0.39079900
N	0.56000500	0.70611500	0.57283200
C	1.79852600	0.33055800	1.02647500
O	-1.02242400	1.35014300	-0.95671600
O	1.92603600	-0.53903200	1.87524900
C	3.05104100	0.96486300	0.42353400
C	3.79177000	-0.04758600	-0.44091900
O	4.95444100	-0.33468900	-0.31661800
C	-0.27281700	-1.62665800	1.05881600
O	0.22303600	-2.38778400	0.26403200
O	-0.71852900	-1.97351000	2.27138000
C	-0.42461900	-3.32153700	2.68569100
C	0.00375700	3.20287800	0.20838300
C	1.32929900	3.79024900	0.72386900
C	-0.52253700	4.10421700	-0.92729800
C	-1.00607200	3.17217100	1.36844600
C	-2.72395100	-0.36519000	-0.73121200
C	-3.71994800	0.58113400	-0.47547800
C	-5.06490500	0.22103300	-0.52951600
C	-5.43024400	-1.08895000	-0.83520100
C	-4.44234300	-2.03648300	-1.09691200
C	-3.09760500	-1.67472200	-1.05162900
O	2.97241600	-0.54209900	-1.38634600
C	3.38168300	-2.95180600	-1.59882500
C	3.49809200	-1.58658500	-2.25397900
H	-1.27386900	0.21364800	1.53389500
H	-0.68867300	-0.66445600	-1.32835400
H	0.97736400	1.81759300	-1.17500600
H	2.84795500	1.83900600	-0.19111300
H	3.71558500	1.25158600	1.23661400
H	-0.86570400	-3.42246300	3.67452400
H	-0.86369600	-4.03920200	1.99166200
H	0.65561300	-3.46470500	2.72848100
H	1.15192100	4.79938500	1.10527800
H	1.75161200	3.20466900	1.54400300

H	2.07782300	3.87412700	-0.06962700
H	-0.64493600	5.12822100	-0.56357500
H	-1.48418800	3.75304300	-1.30251500
H	0.17799200	4.13228700	-1.76839400
H	-0.64382600	2.55870000	2.19773100
H	-1.16129400	4.18489200	1.75045900
H	-1.97452200	2.78595800	1.04815900
H	-3.44049700	1.60337200	-0.25619600
H	-5.82862500	0.96628000	-0.33657200
H	-6.47728300	-1.36710900	-0.87738500
H	-4.71751700	-3.05507200	-1.34661200
H	-2.33178300	-2.41211100	-1.26802500
H	2.35360800	-3.14267100	-1.28551900
H	3.68279200	-3.72359000	-2.31359600
H	4.03369000	-3.02252700	-0.72652100
H	2.88034400	-1.51663700	-3.14941600
H	4.53097300	-1.34173600	-2.50492100

Imaginary Freq = 0
Electronic Energy (EE) = -1284.900176 Hartree
Zero-point Energy Correction = 0.448886 Hartree
EE + Zero-point Energy = -1284.451290 Hartree

8a

C	-0.84292000	-0.25549300	0.92099300
C	-2.22468400	-0.37310800	0.22397400
C	-0.60205800	-1.07138700	-1.33529200
N	0.07759200	-0.40118600	-0.19967900
C	1.11980100	0.46989900	-0.45219200
O	-1.96215500	-1.18852900	-0.90853400
O	1.60475600	0.59249000	-1.56083500
C	1.65255500	1.25000800	0.75354000
C	2.97183300	1.92151000	0.42320700
O	3.10648600	3.10150200	0.21598600
C	-0.56972800	-1.23869300	2.06808700
O	0.50228400	-1.73146200	2.30647700
O	-1.66247300	-1.40124000	2.83523200
C	-1.49791300	-2.22765200	4.00868000
C	-0.08811600	-2.47707300	-1.74697900
C	1.38134300	-2.39874800	-2.19686000
C	-0.95570500	-2.93357100	-2.93885000
C	-0.21711200	-3.48837600	-0.59603300
C	-2.82396600	0.98284900	-0.13241400
C	-3.05344500	1.35163300	-1.45758400
C	-3.61430700	2.59365300	-1.75768600
C	-3.95987000	3.47453400	-0.73660900
C	-3.74764500	3.10591800	0.59211300
C	-3.18528800	1.86800300	0.89060700
O	3.97441500	1.02880500	0.41648000
C	6.24216200	0.35587200	0.05859900

C	5.28256100	1.52735800	0.03665000
H	-0.74968500	0.73359200	1.37231400
H	-2.92811600	-0.91990400	0.85297100
H	-0.52371000	-0.40279200	-2.19924200
H	0.94712600	2.03911700	1.02447400
H	1.77953600	0.57434900	1.60237000
H	-2.47220400	-2.24116600	4.49111900
H	-1.19538800	-3.23427400	3.71992900
H	-0.74429200	-1.79825000	4.66953000
H	1.69632200	-3.37909500	-2.56680500
H	2.04181300	-2.12369600	-1.37230900
H	1.52409600	-1.66722300	-2.99439700
H	-0.61592200	-3.91078000	-3.29291700
H	-2.00750000	-3.01749900	-2.65858500
H	-0.87878400	-2.23283600	-3.77655800
H	-1.25061400	-3.56850900	-0.25038400
H	0.09569500	-4.47775500	-0.94198300
H	0.41989900	-3.21621400	0.24748700
H	-2.80354500	0.66011400	-2.25199100
H	-3.78143500	2.87001300	-2.79272100
H	-4.39478100	4.43932900	-0.97128800
H	-4.02297700	3.78100100	1.39470700
H	-3.03734900	1.58617100	1.92932500
H	7.24190700	0.69547200	-0.22539500
H	6.30044500	-0.08579400	1.05603400
H	5.92900000	-0.41788600	-0.64547100
H	5.20269000	1.97564400	-0.95586900
H	5.57313500	2.31182900	0.73925400

Imaginary Freq = 0
Electronic Energy (EE) = -1284.906081 Hartree
Zero-point Energy Correction = 0.448256 Hartree
EE + Zero-point Energy = -1284.457825 Hartree

enolate-9a

C	-0.78980200	0.52475900	-0.58586100
C	-1.24211500	-0.18832700	0.71123300
C	0.70746900	-1.24456700	-0.05845100
N	0.60116800	0.10814600	-0.60387100
C	1.54743100	0.92028500	-1.28531200
O	-0.54457300	-1.42645000	0.64786500
O	1.04413200	1.70281400	-2.12559300
C	2.94631800	0.84540900	-1.04929800
C	3.73218300	0.49141600	0.06838600
O	4.96586900	0.53558900	0.13482600
C	-0.96130700	2.03501100	-0.49754600
O	-0.52852100	2.73772700	0.38382100
O	-1.79611200	2.48354800	-1.46263300
C	-1.94317100	3.90527800	-1.52841100
C	0.85895800	-2.42455300	-1.07215200

C	2.27309700	-2.41763100	-1.68136500
C	0.67685000	-3.74191500	-0.29002000
C	-0.17795800	-2.34059400	-2.20450100
C	-2.72861000	-0.40919700	0.86307900
C	-3.34625200	-1.59760000	0.46368700
C	-4.72561300	-1.75743200	0.59018600
C	-5.50871600	-0.73137000	1.11667900
C	-4.89950500	0.45521500	1.52423600
C	-3.52045000	0.61089300	1.40354100
O	3.01405500	0.08911100	1.21303800
C	4.00698800	1.14090100	3.18618800
C	3.77938800	-0.14310100	2.39431600
H	-1.36918300	0.16918600	-1.44518800
H	-0.87402700	0.41006900	1.55384100
H	1.52926700	-1.28607600	0.65631400
H	3.52468400	1.32244800	-1.83058900
H	-2.63143200	4.09171600	-2.35182500
H	-2.34767300	4.30272700	-0.59458700
H	-0.97518500	4.36883700	-1.72768700
H	2.39318500	-3.28912700	-2.33577300
H	2.46474000	-1.51514100	-2.26138500
H	3.04220200	-2.46931400	-0.90552200
H	0.84518100	-4.59716700	-0.95358800
H	-0.32509500	-3.82299200	0.13509700
H	1.39721400	-3.81174200	0.53206000
H	-0.05706300	-1.41805400	-2.77769300
H	-0.05034500	-3.18352700	-2.89262100
H	-1.19983800	-2.37676300	-1.81915200
H	-2.73362100	-2.39763600	0.06904800
H	-5.18908200	-2.68799500	0.27869100
H	-6.58191500	-0.85689300	1.21487600
H	-5.49745500	1.25801600	1.94292200
H	-3.04901900	1.53153100	1.73229200
H	3.05411100	1.62137300	3.42470400
H	4.53120400	0.92118400	4.12405100
H	4.61389900	1.83684800	2.60408200
H	3.19082800	-0.85531000	2.98228000
H	4.73634200	-0.59921300	2.13018600

Imaginary Freq = 0
Electronic Energy (EE) = -1284.390751 Hartree
Zero-point Energy Correction = 0.433914 Hartree
EE + Zero-point Energy = -1283.956837 Hartree

enolate-9a’

C	0.28489600	0.90056000	-0.08684200
C	1.52904600	0.73057600	-0.87118100
C	0.32241200	-1.33459500	-0.93657900
N	-0.30064200	-0.40982600	0.04713000
C	-1.29955600	-0.67365800	0.94800300

O	1.32884000	-0.55236700	-1.56699000
O	-1.46800300	-0.04956500	1.98964400
C	-2.35151500	-1.74244400	0.57131900
C	-3.72842800	-1.11577500	0.46506700
O	-4.63760700	-1.29712500	1.24286300
C	-0.53014400	2.03194100	-0.20575800
O	-1.74126300	2.17378400	-0.00983000
O	0.24709300	3.17166700	-0.56583600
C	-0.49301800	4.36872500	-0.71700000
C	0.98746900	-2.66106200	-0.39184900
C	-0.03652600	-3.75018600	-0.01960200
C	1.84552000	-3.23213800	-1.54341800
C	1.87792200	-2.39309400	0.83159100
C	2.88348100	0.72804200	-0.15636600
C	2.99464000	1.14957400	1.16909100
C	4.23897500	1.19678500	1.79955000
C	5.39314500	0.83220700	1.10797500
C	5.29381900	0.42280400	-0.22396900
C	4.04972300	0.37751900	-0.84889700
O	-3.82737000	-0.36355700	-0.64526200
C	-4.98131700	1.68775500	0.05354400
C	-5.02143000	0.43640100	-0.80976400
H	1.59162500	1.49352700	-1.65185400
H	-0.40324800	-1.61797200	-1.71541000
H	-2.13500000	-2.22841100	-0.37868700
H	-2.40993300	-2.48696800	1.36455200
H	0.23763600	5.14614000	-0.95451000
H	-1.22879700	4.29767600	-1.52672100
H	-1.02896500	4.63143800	0.20037600
H	0.49544800	-4.69215300	0.15018600
H	-0.57864700	-3.51925200	0.89607200
H	-0.76190900	-3.92590300	-0.82064500
H	2.29826800	-4.17952900	-1.23153400
H	2.63650700	-2.54032500	-1.82857400
H	1.23398100	-3.42995700	-2.43137900
H	1.30850300	-1.93829000	1.64549500
H	2.29904300	-3.33724700	1.19528400
H	2.70572200	-1.72841400	0.58841300
H	2.08347900	1.42868400	1.68846700
H	4.30593900	1.51896900	2.83422300
H	6.36135200	0.86982700	1.59741400
H	6.18717100	0.13977400	-0.77285200
H	3.97359200	0.05353300	-1.88213600
H	-4.03031000	2.20579700	-0.08283600
H	-5.81012800	2.34798500	-0.22704300
H	-5.08417600	1.43231400	1.10945200
H	-5.01648900	0.68948600	-1.87135900
H	-5.89438400	-0.18425600	-0.59049600

Imaginary Freq = 0

Electronic Energy (EE) = -1284.357338 Hartree
Zero-point Energy Correction = 0.432915 Hartree
EE + Zero-point Energy = -1283.924423 Hartree

enolate-8a

C	-0.94426000	-0.12960200	0.87568600
C	-1.99954800	0.68510600	0.06978400
C	-1.03311300	-0.88740400	-1.37810000
N	-0.25563700	-0.90274900	-0.13657600
C	1.20520000	-0.72194800	-0.26428000
O	-2.21624600	-0.11725500	-1.08766200
O	1.64843900	-0.55699400	-1.41000300
C	1.88395800	-0.77960600	0.97210900
C	3.25896900	-0.52896100	1.23767700
O	3.81502300	-0.66596600	2.33226500
C	-1.58253600	-0.94415200	2.00292100
O	-1.58469700	-2.13770400	2.14989400
O	-2.15725700	-0.09512500	2.90385300
C	-2.74439200	-0.71410700	4.05560100
C	-1.46651600	-2.26773000	-1.94354700
C	-0.20553900	-3.11522600	-2.19101200
C	-2.18465800	-2.01702200	-3.28472000
C	-2.40613300	-3.01291600	-0.98337800
C	-1.57030500	2.10370700	-0.27021600
C	-0.35298300	2.38613400	-0.90716600
C	-0.00998000	3.70079500	-1.21374300
C	-0.86810000	4.75358400	-0.89281200
C	-2.07882800	4.48268500	-0.25984800
C	-2.42317300	3.16577000	0.04489800
O	3.99637000	-0.07756700	0.15412400
C	5.99350200	0.63925300	-0.90590200
C	5.37515900	0.17243900	0.40263700
H	-0.23928200	0.54134100	1.37377900
H	-2.95388900	0.73860000	0.59787200
H	-0.43595400	-0.38716100	-2.14735500
H	1.33980100	-1.12337600	1.84107100
H	-3.13209800	0.10241200	4.66305800
H	-3.55156300	-1.38927900	3.76338900
H	-1.99434800	-1.27979500	4.61155300
H	-0.48277400	-4.05970400	-2.67313600
H	0.30046000	-3.34432700	-1.25085200
H	0.51095900	-2.59140800	-2.82770500
H	-2.48120100	-2.96788300	-3.74088900
H	-3.08146400	-1.40844500	-3.14408700
H	-1.52870500	-1.49689300	-3.99095600
H	-3.32857700	-2.44948100	-0.81896100
H	-2.67502300	-3.98709900	-1.40772600
H	-1.92760800	-3.17745400	-0.01703300
H	0.33814500	1.58940700	-1.16071400
H	0.93819800	3.90070200	-1.70104400

H	-0.59262900	5.77546400	-1.13226100
H	-2.75511800	5.29199400	-0.00378200
H	-3.36901600	2.95814700	0.53651700
H	7.06202900	0.84107700	-0.77079500
H	5.87643800	-0.12247600	-1.68086000
H	5.50857700	1.55334000	-1.25871400
H	5.48748900	0.92937200	1.18737000
H	5.86320700	-0.73771400	0.76898700

Imaginary Freq = 0
Electronic Energy (EE) = -1284.398545 Hartree
Zero-point Energy Correction = 0.433791 Hartree
EE + Zero-point Energy = -1283.964754 Hartree

enolate-8a’

C	0.86373900	0.29751400	0.73325700
C	2.18657700	0.37975800	0.02951100
C	0.54635300	1.59784600	-1.19145000
N	-0.10598800	0.68364100	-0.25007700
C	-1.24861300	0.02641100	-0.59630300
O	1.94010100	1.27638500	-1.09371800
O	-1.93639100	0.33150600	-1.57236700
C	-1.63288300	-1.10239500	0.35969700
C	-2.90048000	-1.78266100	-0.07902000
O	-2.97597400	-2.83326500	-0.67459700
C	0.63128400	0.41265700	2.09603000
O	-0.43911000	0.62275800	2.69567300
O	1.81324800	0.20018900	2.85060500
C	1.63492900	0.22712300	4.25687100
C	0.30682600	3.11390100	-0.93578300
C	-1.19052200	3.42720000	-1.10587400
C	1.11253600	3.89083600	-1.99561500
C	0.76275600	3.53692500	0.47057700
C	2.72297000	-0.94926500	-0.48758100
C	2.37800700	-1.45946200	-1.74372400
C	2.83535700	-2.71076700	-2.15491400
C	3.65661200	-3.47133800	-1.32280500
C	4.01714000	-2.96490700	-0.07312000
C	3.55538000	-1.71658100	0.33741300
O	-3.99443100	-1.07736200	0.29693200
C	-6.32854800	-0.59779900	0.30246400
C	-5.26324200	-1.57279500	-0.16242800
H	2.94855000	0.82938100	0.67396300
H	0.19765000	1.36316200	-2.20213800
H	-0.81841400	-1.82955200	0.38617800
H	-1.73600600	-0.68237300	1.36577500
H	2.62291100	0.04672700	4.68755900
H	1.25225200	1.19290800	4.60204700
H	0.93925900	-0.54929200	4.59118600
H	-1.35709600	4.50616500	-1.00919600

H	-1.78865700	2.91902200	-0.34717500
H	-1.56018900	3.10522800	-2.08285000
H	0.96280100	4.96903200	-1.87074000
H	2.17970800	3.67530800	-1.90956300
H	0.79288600	3.62401900	-3.00928300
H	1.82390800	3.32489700	0.62229200
H	0.61184500	4.61572600	0.59652500
H	0.19518400	3.01945800	1.24625100
H	1.75627900	-0.86564400	-2.40240000
H	2.55020000	-3.09214500	-3.13052000
H	4.01321900	-4.44446700	-1.64454500
H	4.65906800	-3.54483100	0.58315000
H	3.82048400	-1.33204200	1.31701500
H	-7.31504100	-0.93587200	-0.02993800
H	-6.33844100	-0.52120500	1.39258800
H	-6.14534100	0.39727200	-0.10896500
H	-5.23592900	-1.65488400	-1.25209000
H	-5.42577700	-2.57637800	0.24198000

Imaginary Freq = 0
Electronic Energy (EE) = -1284.394202 Hartree
Zero-point Energy Correction = 0.432783 Hartree
EE + Zero-point Energy = -1283.961419 Hartree

TS-enolate-9a

C	-0.47827500	-0.24491800	0.37827500
N	0.10733500	1.09348900	0.28916200
C	-0.74745000	1.97503200	-0.50866600
O	-1.87799700	1.17164900	-0.85807900
C	-1.50335300	-0.20775600	-0.78191500
C	0.63509000	-1.32733900	0.14294500
C	2.07653900	0.01551700	0.93058100
C	1.51202600	1.13937600	0.17716400
O	2.07063200	2.02763600	-0.45606500
O	0.91249400	-1.64348800	-1.01263300
C	-1.25242100	3.25116400	0.21661000
C	-2.19046400	4.00675400	-0.74456500
C	-2.00697200	2.88348500	1.50508100
C	-0.05325500	4.15100900	0.56282400
C	-2.71512200	-1.08933700	-0.61291400
C	-2.55705800	-2.47989900	-0.69272000
C	-3.65078600	-3.32511100	-0.52560100
C	-4.92056800	-2.79768900	-0.28433300
C	-5.08449000	-1.41599600	-0.21133700
C	-3.98884600	-0.56681500	-0.37368400
C	3.40880500	-0.50907900	0.76277500
O	4.03161700	-1.14127300	1.61375900
O	3.91455200	-0.32675900	-0.49117100
C	5.50444900	-0.66546700	-2.22147500
C	5.16982900	-0.95087000	-0.76762500

H	-0.99033200	-0.40242800	1.33242300
O	0.57654100	-2.41579000	1.05107400
C	0.66180800	-2.23027800	2.45316500
H	-0.19424500	2.27480500	-1.40878400
H	-0.96007000	-0.49750100	-1.68864800
H	1.76527700	0.01015600	1.96831300
H	-2.54411900	4.93345600	-0.27991300
H	-1.66927100	4.27478800	-1.66976700
H	-3.05816600	3.39953800	-1.01071600
H	-2.34322200	3.79119500	2.01772700
H	-1.35700600	2.33012500	2.18787000
H	-2.88267700	2.26721400	1.29089300
H	0.61899500	3.66461000	1.27181300
H	0.53633900	4.39158600	-0.32473000
H	-0.41023900	5.08550200	1.01040600
H	-1.57030400	-2.89079900	-0.87810600
H	-3.51072500	-4.39952000	-0.58490200
H	-5.77229900	-3.45793800	-0.15668300
H	-6.06815700	-0.99450100	-0.02917400
H	-4.11330800	0.50725100	-0.32473700
H	6.46327300	-1.12466200	-2.48522600
H	4.73044400	-1.06944700	-2.87816300
H	5.57240500	0.41087900	-2.39848400
H	5.93581600	-0.55327400	-0.09310800
H	5.09387100	-2.02556300	-0.57656900
H	0.35649100	-3.17902000	2.90215200
H	1.68844700	-2.00263300	2.75452100
H	-0.00818300	-1.44472400	2.82486800

Imaginary Freq = 1
Electronic Energy (EE) = -1284.372338 Hartree
Zero-point Energy Correction = 0.433108 Hartree
EE + Zero-point Energy = -1283.939230 Hartree

TS-enolate-8a

C	0.10898600	-0.23402600	0.28952700
N	-0.95020200	0.60779400	-0.23466800
C	-0.42985000	1.80782000	-0.88528400
O	0.95301100	1.80144600	-0.46216300
C	1.25948800	0.76152100	0.48637900
C	-0.36789300	-0.93347500	1.55131600
C	-2.31319600	-1.16305900	0.56473400
C	-2.23016600	0.14968400	-0.10462700
O	-3.21166000	0.83549500	-0.42571500
O	0.12571400	-2.01749200	1.91099400
C	-1.05147900	3.19818300	-0.56961600
C	0.02084200	4.26836100	-0.87575400
C	-1.46871500	3.30839700	0.90361400
C	-2.25140700	3.47625700	-1.49693100
C	2.65115500	0.23136100	0.22306200

C	3.07092400	-0.96384500	0.80577500
C	4.36132300	-1.42903900	0.59669500
C	5.24894000	-0.71102500	-0.19228800
C	4.83427900	0.47693600	-0.77532300
C	3.54383000	0.94560900	-0.56934300
C	-2.16713300	-2.38174600	-0.15377500
O	-2.57914400	-3.49414400	0.18133100
O	-1.43931200	-2.26882700	-1.32224600
C	-0.35303400	-3.01833100	-3.29232400
C	-1.14706800	-3.45578100	-2.07875900
O	-0.77161500	-0.01676000	2.50353100
C	-1.22162700	-0.52645000	3.76268100
H	0.38566700	-1.00641200	-0.40960300
H	-0.41886700	1.67509700	-1.96125200
H	1.20723700	1.18684500	1.47915500
H	-3.12237500	-1.20851900	1.26988000
H	-0.42669100	5.25452000	-0.79679500
H	0.41053800	4.15802000	-1.88340700
H	0.85282200	4.20592200	-0.19084400
H	-1.78390000	4.32602000	1.11901800
H	-2.28849400	2.64237300	1.12629000
H	-0.64331300	3.06593200	1.56231100
H	-3.02954700	2.74511600	-1.35863900
H	-2.64853800	4.46542600	-1.28637500
H	-1.93778100	3.46583600	-2.53845100
H	2.38615600	-1.53748500	1.40165400
H	4.66732600	-2.35513600	1.04609900
H	6.24678800	-1.07488000	-0.35356100
H	5.51100300	1.03878100	-1.39236300
H	3.21651300	1.85740100	-1.02399800
H	-0.11099700	-3.87375000	-3.91604200
H	-0.92247700	-2.31058700	-3.88167800
H	0.57071100	-2.54056300	-2.99117200
H	-0.59084600	-4.14336600	-1.45824600
H	-2.07398500	-3.93672100	-2.35700300
H	-1.49097400	0.33742800	4.34855700
H	-2.08125300	-1.16499500	3.61798200
H	-0.43844700	-1.08870900	4.24674900

Imaginary Freq = 1
Electronic Energy (EE) = -1284.365991 Hartree
Zero-point Energy Correction = 0.433646 Hartree
EE + Zero-point Energy = -1283.932345 Hartree

TS-enolate-8a’

C	0.01129500	0.38553500	0.54106700
N	-1.39439300	0.06838100	0.37847700
C	-1.50661100	-1.37194700	0.11576600
O	-0.27480900	-1.67127400	-0.56258900
C	0.62809000	-0.52749600	-0.51201000

C	-0.06802100	2.28504400	0.10146600
C	-1.52224900	2.40734100	0.60114700
C	-2.27801800	1.10951000	0.40667600
O	-3.50000300	1.03251000	0.30885000
O	0.85392000	2.96672400	0.58607700
C	-2.69501800	-1.95232400	-0.69738300
C	-2.32230100	-3.41132400	-1.04861500
C	-2.93262100	-1.16728800	-1.99763900
C	-3.96756100	-1.98940400	0.17104300
C	2.06495200	-0.99177600	-0.39733900
C	3.09762600	-0.04664800	-0.33611900
C	4.42694600	-0.45297300	-0.31761700
C	4.75298800	-1.81088200	-0.35863600
C	3.73180000	-2.75475200	-0.41383500
C	2.39561200	-2.34705300	-0.43643900
C	0.52606200	0.31555700	1.92182700
O	1.68817800	0.22662500	2.26085200
O	-0.47230200	0.48606500	2.85717900
C	-0.02029100	0.63037800	4.20329900
O	-0.18218600	2.21480400	-1.35380000
C	0.44576800	3.08492500	-3.48235100
C	0.93106700	2.69619000	-2.09249200
H	-1.48395900	-1.89396300	1.08574000
H	0.51894600	-0.01339900	-1.47329900
H	-2.05083600	3.21209500	0.08666500
H	-1.48327400	2.63368300	1.66848900
H	-3.17278000	-3.89473100	-1.54082400
H	-2.08466000	-3.98881200	-0.14847500
H	-1.45960800	-3.45438200	-1.71416900
H	-3.67628300	-1.68613900	-2.61303000
H	-3.30139000	-0.16261900	-1.79136600
H	-2.00841800	-1.09280700	-2.57643400
H	-3.80083900	-2.58645600	1.07520000
H	-4.27693900	-0.98620600	0.46050800
H	-4.78052800	-2.46297900	-0.39065200
H	2.85290600	1.00661600	-0.25819600
H	5.21288700	0.29273800	-0.25401700
H	5.79165600	-2.12573000	-0.34020900
H	3.96996300	-3.81378700	-0.44087600
H	1.59897300	-3.07815500	-0.48254700
H	-0.91983300	0.77226400	4.80284600
H	0.51885900	-0.26058500	4.53561900
H	0.64172900	1.49411800	4.29954600
H	1.28310700	3.44338700	-4.09163800
H	-0.00922400	2.23106000	-3.99261800
H	-0.30319200	3.87934800	-3.41943900
H	1.37536700	3.54683400	-1.56836000
H	1.70865000	1.92264700	-2.17123700

Imaginary Freq = 1

Electronic Energy (EE) = -1284.362768 Hartree
Zero-point Energy Correction = 0.432487 Hartree
EE + Zero-point Energy = -1283.930281 Hartree

alkoxide-9a

C	-0.42840600	-0.16250500	0.53070000
N	0.14707400	1.17070300	0.33900000
C	-0.69123800	1.97799100	-0.55430000
O	-1.66232400	1.05057600	-1.05100000
C	-1.25560300	-0.30261800	-0.75710000
C	0.81650900	-1.15758700	0.66900000
C	2.00849300	-0.08866900	0.97610000
C	1.51927400	1.20092400	0.33760000
O	2.19496000	2.14013400	-0.06230000
O	1.01702100	-1.92048400	-0.34230000
C	-1.40545600	3.18728000	0.10160000
C	-2.30546600	3.84386600	-0.96160000
C	-2.25184900	2.74786700	1.30700000
C	-0.34737100	4.20459600	0.56160000
C	-2.45559000	-1.21653600	-0.67810000
C	-2.23716900	-2.59043300	-0.50150000
C	-3.31285600	-3.47164900	-0.41980000
C	-4.62306300	-2.99666900	-0.51770000
C	-4.84528300	-1.63287200	-0.69900000
C	-3.76809700	-0.74665600	-0.77880000
C	3.35040100	-0.59464900	0.53950000
O	4.13400900	-1.15013700	1.28910000
O	3.59509800	-0.40634500	-0.76260000
C	4.88710200	-0.63222500	-2.76350000
C	4.83050600	-0.95002600	-1.28440000
H	-1.07960500	-0.18681500	1.40690000
O	0.58242200	-2.00469000	1.89360000
C	0.50261300	-1.41009100	3.17100000
H	-0.06104300	2.34440000	-1.37440000
H	-0.57739800	-0.66470800	-1.53800000
H	2.07499100	0.09233200	2.05010000
H	-2.78407900	4.73855900	-0.55110000
H	-1.72387000	4.14977500	-1.83810000
H	-3.08985600	3.16265400	-1.29870000
H	-2.75806200	3.61445900	1.74410000
H	-1.62984300	2.29967600	2.08720000
H	-3.01573800	2.02245600	1.01840000
H	0.31453500	3.78200600	1.32120000
H	0.27512400	4.54130500	-0.27220000
H	-0.83978500	5.08268800	0.99140000
H	-1.21296400	-2.94311700	-0.42330000
H	-3.12894000	-4.53194600	-0.27930000
H	-5.46035200	-3.68398100	-0.45530000
H	-5.85888900	-1.25378700	-0.78100000
H	-3.94461300	0.31124200	-0.92480000

H	5.81250800	-1.03381100	-3.18700000
H	4.04400800	-1.08173800	-3.29450000
H	4.87208500	0.44727400	-2.93620000
H	5.67010000	-0.50411400	-0.74480000
H	4.84342300	-2.02742600	-1.10330000
H	0.03592400	-2.13929800	3.84180000
H	1.49121000	-1.16257700	3.58610000
H	-0.11260000	-0.49890100	3.19600000

Imaginary Freq = 0
Electronic Energy (EE) = -1284.380131 Hartree
Zero-point Energy Correction = 0.433629 Hartree
EE + Zero-point Energy = -1283.946502 Hartree

alkoxide-8a

C	0.14559900	-0.30152800	0.21693300
N	-0.60109100	0.91830800	-0.06156500
C	0.32196300	1.91106800	-0.60428900
O	1.56869400	1.57748200	0.02809800
C	1.53341000	0.23787700	0.57989000
C	-0.69455600	-1.08794600	1.29137200
C	-2.18422400	-0.51076900	0.90717600
C	-1.91459900	0.86968800	0.30635600
O	-2.74398200	1.76673900	0.20699800
O	-0.51749200	-2.34908000	1.35834600
C	0.04310700	3.42460400	-0.42362900
C	1.33018600	4.17111100	-0.84032900
C	-0.29127200	3.76356500	1.03824000
C	-1.09214600	3.86519100	-1.36768700
C	2.68496700	-0.61237700	0.08760200
C	2.64079300	-1.99739900	0.30366800
C	3.70547500	-2.80050500	-0.09987700
C	4.82187100	-2.23914700	-0.72308100
C	4.86490500	-0.86346000	-0.94218400
C	3.80225100	-0.05364600	-0.53722700
C	-2.89167600	-1.45130700	-0.01580700
O	-3.79965200	-2.18946700	0.29188100
O	-2.37252900	-1.42521800	-1.27621800
C	-2.16106800	-2.22207100	-3.51357900
C	-2.89410900	-2.39737600	-2.19648500
O	-0.39622100	-0.32426800	2.56960300
C	-0.79236800	-1.01845900	3.72944300
H	0.19449800	-0.93668800	-0.67306500
H	0.43282500	1.71856400	-1.68508800
H	1.59473500	0.33587500	1.66693000
H	-2.80382300	-0.40460100	1.79499300
H	1.14870800	5.25055200	-0.81586000
H	1.63207100	3.90591600	-1.85959400
H	2.15976500	3.93949000	-0.17130500
H	-0.34341900	4.85084900	1.16328700

H	-1.25188700	3.33967100	1.33125600
H	0.48139700	3.38008400	1.70884200
H	-2.02716100	3.36210300	-1.12281400
H	-1.24229300	4.94678600	-1.27991000
H	-0.83414000	3.65217000	-2.41163500
H	1.75810400	-2.43330000	0.77172300
H	3.65876300	-3.87202100	0.06706300
H	5.64742200	-2.86935900	-1.03867700
H	5.72616900	-0.41725800	-1.43008800
H	3.82744600	1.01565600	-0.70850600
H	-2.53191700	-2.94610300	-4.24596400
H	-2.31392000	-1.21679800	-3.91457700
H	-1.08802700	-2.38136300	-3.38431000
H	-2.74236800	-3.39556300	-1.77759900
H	-3.97147400	-2.24434500	-2.30910700
H	-0.33109100	-0.51229500	4.58365200
H	-1.88626600	-1.00495600	3.87025300
H	-0.46805000	-2.06342400	3.69236000

Imaginary Freq = 0
Electronic Energy (EE) = -1284.379311 Hartree
Zero-point Energy Correction = 0.434258 Hartree
EE + Zero-point Energy = -1283.945053 Hartree

alkoxide-8a’

C	-0.01280200	0.39667800	0.53662100
N	-1.43069600	0.09232400	0.36288000
C	-1.59297000	-1.34975800	0.14544700
O	-0.29576800	-1.75508800	-0.32916800
C	0.57874300	-0.60488700	-0.46352500
C	0.07943400	2.02088300	0.21018000
C	-1.39783700	2.41570400	0.55331500
C	-2.25784300	1.18663800	0.34964100
O	-3.47510400	1.17433200	0.20025200
O	1.03775000	2.66138800	0.74589700
C	-2.70591300	-1.87117100	-0.80806400
C	-2.37950900	-3.35124700	-1.10929700
C	-2.73820400	-1.08060800	-2.12575100
C	-4.07526600	-1.82537100	-0.10220000
C	2.02970800	-1.01354500	-0.38918500
C	3.01737900	-0.01953300	-0.38701900
C	4.36255800	-0.37102400	-0.41242700
C	4.74530100	-1.71444800	-0.44369900
C	3.76555100	-2.70354700	-0.44329300
C	2.41324200	-2.35478400	-0.41879500
C	0.45574000	0.20471800	1.97411300
O	1.55039500	-0.14954200	2.33180100
O	-0.52521100	0.53213800	2.86167300
C	-0.12306200	0.55143800	4.23442200
O	0.10549700	1.99040300	-1.32715000

C	0.81887700	2.99999500	-3.36867700
C	0.48884300	3.22130800	-1.89720800
H	-1.73698200	-1.84292900	1.11938400
H	0.41314500	-0.15838600	-1.44700000
H	-1.77054800	3.24067100	-0.05502900
H	-1.44224500	2.70887600	1.60498800
H	-3.19103800	-3.78907700	-1.69974100
H	-2.28592700	-3.93378500	-0.18619400
H	-1.44651000	-3.45045700	-1.66472700
H	-3.44102800	-1.55061600	-2.82265600
H	-3.05735000	-0.05090200	-1.96351800
H	-1.75284400	-1.07061600	-2.59874100
H	-4.05818100	-2.42814300	0.81339800
H	-4.35543600	-0.80415500	0.15256400
H	-4.84098500	-2.25017100	-0.76073600
H	2.72075800	1.02288900	-0.31883100
H	5.11800500	0.40809100	-0.39395100
H	5.79669600	-1.98428000	-0.46028000
H	4.04960900	-3.75145400	-0.46074200
H	1.64890200	-3.12144800	-0.41484300
H	-1.01115300	0.83754100	4.79692000
H	0.22882300	-0.43294000	4.55163500
H	0.67708100	1.27900900	4.38529400
H	1.10741700	3.94477100	-3.84437800
H	1.64735900	2.29346300	-3.47278400
H	-0.04300000	2.59353700	-3.90682400
H	-0.32392400	3.96519700	-1.81140800
H	1.35194700	3.62538300	-1.35597900

Imaginary Freq = 0
Electronic Energy (EE) = -1284.366415 Hartree
Zero-point Energy Correction = 0.432977 Hartree
EE + Zero-point Energy = -1283.933438 Hartree

enolate-10

C	0.22717400	0.10552200	0.54917500
N	0.14599400	-1.33020100	0.31620600
C	1.28507200	-1.77103700	-0.47774600
O	1.97584100	-0.56584400	-0.86616400
C	1.14424100	0.57372500	-0.60674100
C	-1.24003700	0.59976300	0.49569900
C	-2.03058000	-0.51680300	0.09274200
C	-1.17519400	-1.66836900	-0.13605600
O	-1.39980200	-2.76029600	-0.63855000
O	-1.51963000	1.76793800	0.78553000
C	2.27281700	-2.71441700	0.25535000
C	3.44959700	-3.02126200	-0.68968900
C	2.79387300	-2.06812600	1.54906900
C	1.52953800	-4.01935700	0.59003700
H	3.09588600	-3.47083600	-1.62383400

H	4.14175100	-3.72852600	-0.21998900
H	4.00196200	-2.11297000	-0.94020800
H	1.96816700	-1.85313100	2.23206000
H	3.32212000	-1.13478700	1.34074200
H	3.48650500	-2.74694300	2.05849500
H	0.67896600	-3.82748400	1.24678900
H	2.20827000	-4.72094000	1.08775800
H	1.13795200	-4.49758600	-0.31182300
C	1.99873700	1.79679500	-0.34609200
C	3.36246700	1.80592400	-0.65368200
C	4.12998400	2.95114900	-0.43761800
C	3.54343600	4.10135600	0.08661500
C	2.18256300	4.09597000	0.39526400
C	1.41198900	2.95455300	0.18189900
H	3.81530600	0.90961500	-1.05747700
H	5.18821400	2.94193300	-0.67960100
H	4.14029200	4.99190700	0.25597200
H	1.71632500	4.98380500	0.81033300
H	0.35526500	2.94261400	0.43649400
C	-3.46530400	-0.56656500	-0.08934400
O	-4.15511200	-1.55685700	-0.26353300
O	-4.04057900	0.68528700	-0.04031100
C	-5.88896100	2.17623300	-0.05628100
C	-5.46258200	0.72153300	-0.16038400
H	-6.97820000	2.25746200	-0.13845500
H	-5.43456200	2.77080100	-0.85282600
H	-5.57708000	2.60222200	0.90035300
H	-5.91700600	0.11382900	0.62936800
H	-5.76684500	0.28188600	-1.11624200
H	0.67181400	0.35512000	1.51769800
H	0.90797700	-2.29092300	-1.36736000
H	0.51624200	0.76298800	-1.49078300

Imaginary Freq = 0
Electronic Energy (EE) = -1168.650852 Hartree
Zero-point Energy Correction = 0.380266 Hartree
EE + Zero-point Energy = -1283.989382 Hartree

enolate-13

C	0.36917200	-0.42041400	-0.73500700
N	0.05260900	0.99460600	-0.83216300
C	1.22596900	1.68656600	-0.32208500
O	1.71563500	0.84466200	0.75656900
C	1.33245200	-0.50888300	0.49049800
C	-0.97902900	-1.14108600	-0.54351500
C	-1.96750000	-0.11830600	-0.45954600
C	-1.34316200	1.19422300	-0.55213100
O	-1.84108400	2.30702100	-0.46056400
O	-1.02101900	-2.37499000	-0.49785800
C	1.20009500	3.15850400	0.14983800

C	0.35950900	3.36919000	1.42203900
C	2.66664500	3.53667100	0.46417000
C	0.69724500	4.05324000	-0.99829300
H	0.69369300	2.69856500	2.21719500
H	-0.69762100	3.19539600	1.22834200
H	0.48308700	4.40063400	1.77167300
H	3.31113000	3.40040300	-0.41137300
H	3.06621800	2.93015600	1.27967700
H	2.72083000	4.58959200	0.75846800
H	0.79466800	5.10693700	-0.71371700
H	-0.34856300	3.84208400	-1.21993000
H	1.29273000	3.89859200	-1.90560000
C	2.52386300	-1.42659000	0.28174500
C	3.83573300	-0.97908100	0.44844200
C	4.91029100	-1.85145700	0.26371900
C	4.68380100	-3.18118000	-0.08481600
C	3.37286600	-3.63343200	-0.24977600
C	2.29902200	-2.76517200	-0.06938200
H	4.00327400	0.05459500	0.72445900
H	5.92533600	-1.48929000	0.39442600
H	5.51878800	-3.85951800	-0.22795500
H	3.18585800	-4.66659500	-0.52475400
H	1.27649500	-3.10712900	-0.20944800
C	-3.39768300	-0.28295100	-0.31808200
O	-4.25746500	0.57626700	-0.41570200
O	-3.73894800	-1.59426600	-0.04790100
C	-5.63175600	-1.65003500	1.50411500
C	-5.13344000	-1.87702100	0.08072200
H	-5.04600100	-2.23990000	2.21462300
H	-6.68292200	-1.94892600	1.58980700
H	-5.54925500	-0.59484000	1.77089300
H	-5.23064100	-2.92963500	-0.19884600
H	-5.70091900	-1.26368500	-0.62330600
H	0.86595100	-0.79446800	-1.63670800
H	1.97567300	1.63255800	-1.12812200
H	0.77241700	-0.86404000	1.36305300

Imaginary Freq = 0
Electronic Energy (EE) = -1168.637951 Hartree
Zero-point Energy Correction = 0.380689 Hartree
EE + Zero-point Energy = -1283.976058 Hartree

enolate-15

C	-0.02473400	1.03593300	-0.04090900
N	-1.41634300	0.67486900	0.25744000
C	-1.47154400	-0.78381200	0.27181000
O	-0.45468800	-1.22114900	-0.67318600
C	0.46457100	-0.15829800	-0.92569300
C	-0.11016800	2.35824500	-0.86992800
C	-1.47206300	2.63600700	-1.00220400

C	-2.28714700	1.63815800	-0.40300700
O	-3.51622100	1.54295300	-0.36062200
O	0.91616200	2.90994500	-1.30128800
C	-2.77945200	-1.56867200	0.00448900
C	-2.42379600	-3.06378300	0.18313300
C	-3.32604900	-1.36579400	-1.41960200
C	-3.83229000	-1.18995400	1.06300800
H	-2.01280600	-3.25942400	1.17974600
H	-1.68973700	-3.38832800	-0.55697700
H	-3.32643300	-3.67256800	0.06737700
H	-3.68546000	-0.34799000	-1.56274300
H	-4.16129000	-2.05472700	-1.59149100
H	-2.55158300	-1.58237400	-2.15979100
H	-4.13275200	-0.14864200	0.95018000
H	-3.43916200	-1.34159400	2.07511400
H	-4.71424500	-1.83118500	0.95112900
C	1.91179300	-0.59411300	-0.78523000
C	2.25250300	-1.91611900	-0.49331200
C	3.59166200	-2.29812900	-0.40635700
C	4.60474000	-1.36262900	-0.61148100
C	4.26682600	-0.04099500	-0.90771800
C	2.93065000	0.34368200	-1.00008300
H	1.45897500	-2.63451900	-0.33107000
H	3.84289600	-3.32943400	-0.17760500
H	5.64655900	-1.65949700	-0.54259100
H	5.04721500	0.69631900	-1.06750000
H	2.65846200	1.37278800	-1.22382100
C	0.70769000	1.24890200	1.28698600
O	1.00211700	2.30870500	1.77613500
O	0.93735300	0.06724900	1.92468600
C	1.56588100	0.16668900	3.20750700
H	2.53484300	0.66245700	3.12297400
H	1.69274200	-0.85854500	3.55254300
H	0.93907100	0.73023600	3.90226000
H	-1.11774100	-1.09825200	1.26133200
H	0.32173600	0.16101700	-1.96509100
H	-1.88912500	3.48666100	-1.52031000

Imaginary Freq = 0
Electronic Energy (EE) = -1129.288919 Hartree
Zero-point Energy Correction = 0.35164 Hartree
EE + Zero-point Energy = -1283.959087 Hartree

10a

C	0.25401000	0.05782900	0.49276000
N	0.31090000	-1.37547700	0.22067100
C	1.54835000	-1.68461300	-0.52179800
O	2.16170700	-0.40241600	-0.72522500
C	1.16577300	0.61850100	-0.63851900
C	-1.21073700	0.35524000	0.42302300

C	-1.91287800	-0.74273200	0.02940400
C	-0.93821200	-1.83623200	-0.24345900
O	-1.11624900	-2.90240700	-0.78529700
O	-1.63792900	1.56393000	0.75159500
C	2.53215800	-2.63568300	0.19302600
C	3.78395700	-2.78031100	-0.69343600
C	2.92494200	-2.08985500	1.57557000
C	1.85157000	-4.00738900	0.35089900
H	3.52380500	-3.16685600	-1.68400800
H	4.48699800	-3.48322200	-0.23767000
H	4.29174100	-1.82296300	-0.82593200
H	2.04597900	-1.96036000	2.21292000
H	3.44395100	-1.13243500	1.49403400
H	3.59695100	-2.79241200	2.07626800
H	0.96189300	-3.94388400	0.98084400
H	2.54819300	-4.71360400	0.81164600
H	1.54125900	-4.41566700	-0.61425400
C	1.78994800	1.96555600	-0.38806300
C	2.83513400	2.11890900	0.52870500
C	3.37782600	3.37733000	0.77323600
C	2.88060600	4.49812900	0.10795800
C	1.84206900	4.35227900	-0.80868200
C	1.30361600	3.09089300	-1.05731000
H	3.23464700	1.24827800	1.03413800
H	4.19231100	3.48370000	1.48103500
H	3.30526000	5.47724300	0.29877800
H	1.45575800	5.21696000	-1.33639000
H	0.50109000	2.98129700	-1.77942300
C	-3.36453200	-0.83241100	-0.13519300
O	-4.01284600	-1.79066300	-0.45569500
O	-3.94957700	0.39199000	0.15254100
C	-5.83164100	1.85918700	0.41106200
C	-5.39838800	0.45302400	0.05142900
H	-6.91988300	1.93336000	0.34143000
H	-5.39933300	2.59462700	-0.27189800
H	-5.54141100	2.11497500	1.43312700
H	-5.82070100	-0.29296400	0.72712900
H	-5.68173800	0.18338500	-0.96758700
H	0.66380900	0.32487900	1.47175200
H	1.26287400	-2.12592200	-1.48533100
H	0.58014600	0.64967300	-1.56818400
H	-2.61216500	1.56086700	0.65584600

Imaginary Freq = 0
Electronic Energy (EE) = -1169.122932 Hartree
Zero-point Energy Correction = 0.394475 Hartree
EE + Zero-point Energy = -1284.087727 Hartree

13

C	0.28530900	-0.44937800	-0.63191600

N	0.10529700	0.99195400	-0.76767100
C	1.34904600	1.58399100	-0.25171200
O	1.67870200	0.73820100	0.86800000
C	1.27692400	-0.59926600	0.58179900
C	-1.10844000	-0.95667100	-0.44323000
C	-1.99977400	0.06704800	-0.43863400
C	-1.24460500	1.34908700	-0.56277900
O	-1.69164000	2.47087900	-0.52848800
O	-1.30907800	-2.26225000	-0.33901100
C	1.45422800	3.06837500	0.15279400
C	0.60123700	3.42137000	1.38483300
C	2.94250500	3.30610500	0.50142400
C	1.08511000	3.95185000	-1.05402700
H	0.83405500	2.75834700	2.22085400
H	-0.46454400	3.36557600	1.17095000
H	0.82776500	4.44573000	1.69529300
H	3.59678500	3.06273800	-0.34214100
H	3.25180700	2.70873900	1.36127100
H	3.09546700	4.36036400	0.74589900
H	1.28368300	4.99981200	-0.81164900
H	0.03146900	3.85431600	-1.31291100
H	1.69068100	3.69694100	-1.93042600
C	2.44424100	-1.53091400	0.29859600
C	3.76354500	-1.08113000	0.36014700
C	4.81922900	-1.95746200	0.10514400
C	4.56694400	-3.29077100	-0.20698000
C	3.24984300	-3.74751800	-0.26178200
C	2.19526300	-2.87380200	-0.01026000
H	3.95776700	-0.04797400	0.61781600
H	5.84021500	-1.59547100	0.15559300
H	5.38816000	-3.97093400	-0.40274900
H	3.04375800	-4.78527100	-0.49942900
H	1.17409500	-3.23975500	-0.05300000
C	-3.45366600	-0.05203700	-0.31591700
O	-4.27184200	0.82511000	-0.34936200
O	-3.80156800	-1.38844500	-0.15586600
C	-5.66610200	-1.58332900	1.43594100
C	-5.21875200	-1.69015900	-0.01119800
H	-5.08090600	-2.24536900	2.07884800
H	-6.71749300	-1.87480800	1.51375700
H	-5.56996200	-0.55888100	1.79888300
H	-5.31688200	-2.70786400	-0.39066500
H	-5.77763800	-1.01034100	-0.65391800
H	0.72740400	-0.89194000	-1.53118600
H	2.09739800	1.40944100	-1.03777600
H	0.73553600	-0.96341800	1.46088200
H	-2.27480000	-2.39396000	-0.23616200

Imaginary Freq = 0
Electronic Energy (EE) = -1169.108800 Hartree

Zero-point Energy Correction = 0.395036 Hartree
EE + Zero-point Energy = -1284.073034 Hartree

15

C	-0.01197000	0.95885400	-0.16403200
N	-1.39820300	0.57768700	0.12547000
C	-1.45410800	-0.89083000	0.27931100
O	-0.38328600	-1.33442100	-0.57419100
C	0.51009000	-0.28441100	-0.94524500
C	-0.19167700	2.25036000	-0.99549800
C	-1.53678200	2.81061900	-0.59730000
C	-2.31464400	1.58972600	-0.09831400
O	-3.51594600	1.54477500	0.04634500
O	0.55897900	2.64050300	-1.84609400
C	-2.74724900	-1.66876200	-0.05646900
C	-2.36886900	-3.16778500	-0.00497400
C	-3.28013200	-1.33358200	-1.45940400
C	-3.81116300	-1.40979900	1.02818600
H	-1.93382100	-3.43755300	0.96278900
H	-1.65417900	-3.43042100	-0.78544800
H	-3.27049600	-3.77032300	-0.14223400
H	-3.67493800	-0.31889300	-1.51284400
H	-4.09406100	-2.01799300	-1.71467400
H	-2.49732700	-1.45613300	-2.21247800
H	-4.13126000	-0.36949100	1.04621900
H	-3.43204200	-1.68229600	2.01892100
H	-4.68726900	-2.03428000	0.83150500
C	1.96517200	-0.66119400	-0.73693800
C	2.32725400	-1.87239100	-0.14584100
C	3.67346500	-2.19894600	0.01502400
C	4.66657400	-1.31985100	-0.41215900
C	4.30762400	-0.11133800	-1.00834700
C	2.96400300	0.21547600	-1.17617100
H	1.55246900	-2.55508000	0.17741400
H	3.94502800	-3.14483700	0.47083400
H	5.71268500	-1.57620400	-0.28738300
H	5.07327500	0.57691200	-1.34832200
H	2.68912300	1.15394000	-1.64672500
C	0.76971800	1.30993600	1.11703800
O	1.27369600	2.38762000	1.31433000
O	0.77948500	0.29450400	1.98668200
C	1.49451600	0.52113100	3.22313100
H	2.54166700	0.73719300	3.01235700
H	1.39699000	-0.40459800	3.78444800
H	1.04973400	1.35432000	3.76759800
H	-1.17172200	-1.12220800	1.31169700
H	0.36086300	-0.07377200	-2.01088600
H	-1.40155100	3.52707000	0.22046700
H	-2.06010500	3.31370200	-1.41010800

Imaginary Freq = 0
Electronic Energy (EE) = -1129.770846 Hartree
Zero-point Energy Correction = 0.364682 Hartree
EE + Zero-point Energy = -1284.068446 Hartree

***t*BuO$^-$**

O	-0.00012800	0.00001200	1.48838200
C	-0.00002400	-0.00000600	0.14774300
C	-1.38377700	-0.46347100	-0.43478500
C	0.29056300	1.43014100	-0.43472200
C	1.09332700	-0.96667200	-0.43462100
H	-1.42669200	-0.47757700	-1.53624400
H	-1.60159400	-1.46987100	-0.06033200
H	-2.16396800	0.20830700	-0.05991200
H	0.29955600	1.47440400	-1.53618300
H	1.26258700	1.76971500	-0.06007300
H	-0.47188300	2.12211400	-0.06007700
H	2.07375300	-0.65219900	-0.05995800
H	0.90150600	-1.97825000	-0.05988700
H	1.12722300	-0.99669500	-1.53608000

Imaginary Freq = 0
Electronic Energy (EE) = -233.223449 Hartree
Zero-point Energy Correction = 0.119416 Hartree
EE + Zero-point Energy = -233.104033 Hartree

***t*BuOH**

O	-0.02038700	-0.00041400	1.45477300
C	0.00570800	0.00000000	0.01158300
C	1.49054600	-0.00315600	-0.35263000
C	-0.69138900	-1.26231000	-0.51359600
C	-0.68577400	1.26577500	-0.51271400
H	-0.93919300	0.00020500	1.74440400
H	1.62403900	-0.00281700	-1.43739100
H	1.98243900	0.88055100	0.06022100
H	1.97848000	-0.88952400	0.05921200
H	-0.64953100	-1.31192600	-1.60549300
H	-1.74674200	-1.27483500	-0.22038200
H	-0.21289700	-2.15587100	-0.10607000
H	-1.74103400	1.28280600	-0.21942100
H	-0.20328800	2.15688800	-0.10454200
H	-0.64371600	1.31598000	-1.60457900

Imaginary Freq = 0
Electronic Energy (EE) = -233.759786 Hartree
Zero-point Energy Correction = 0.134854 Hartree
EE + Zero-point Energy = -233.624932 Hartree

MeOH

C	0.66767200	-0.02030600	0.00000000

H	1.08394000	0.98743200	-0.00012700
H	1.02857700	-0.54486400	0.89330400
H	1.02855800	-0.54508000	-0.89318400
O	-0.74978400	0.12211700	0.00000000
H	-1.14883400	-0.75259200	0.00000200

Imaginary Freq = 0
Electronic Energy (EE) = -115.769821 Hartree
Zero-point Energy Correction = 0.051025 Hartree
EE + Zero-point Energy = -115.718796 Hartree

EtOH

C	-0.08148700	0.54974100	-0.00000600
H	-0.13973900	1.19438000	-0.88778200
H	-0.13975200	1.19438500	0.88776500
O	-1.15510700	-0.39628600	-0.00001300
H	-1.99063200	0.07995500	0.00009300
C	1.22361100	-0.22348100	0.00000600
H	1.29180900	-0.85983500	-0.88566600
H	2.07461800	0.46365900	-0.00000500
H	1.29181000	-0.85981200	0.88569400

Imaginary Freq = 0
Electronic Energy (EE) = -155.101337 Hartree
Zero-point Energy Correction = 0.079529 Hartree
EE + Zero-point Energy = -155.021808 Hartree

H_2O

O	0.00000000	0.00000000	0.11703500
H	0.00000000	0.76349200	-0.46814000
H	0.00000000	-0.76349200	-0.46814000

Imaginary Freq = 0
Electronic Energy (EE) = -76.465603 Hartree
Zero-point Energy Correction = 0.021290 Hartree
EE + Zero-point Energy = -76.444313 Hartree

H_3O^+

H	0.00000000	0.94705200	-0.18405900
H	0.82017100	-0.47352600	-0.18405900
H	-0.82017100	-0.47352600	-0.18405900
O	0.00000000	0.00000000	0.06902200

Imaginary Freq = 0
Electronic Energy (EE) = -76.838116 Hartree
Zero-point Energy Correction = 0.034277 Hartree
EE + Zero-point Energy = -76.803839 Hartree

Experimental

General Methods

Overnight reaction refers to reactions occurring between 15-24 h. All reagents were obtained from commercial sources and was used without further purification. Anhydrous solvents were dried by pre-storing them over activated 3 Å molecular sieves before being passed through an activated alumina column on a solvent tower under N_2 pressure. Solvents were evaporated at 40°C under reduced pressure on a Büchi R-114 rotatory evaporator attached to a Vacuubrand CVC2 pump and a pressure control system, with the exception of water, which was evaporated between 70-80°C. Analytical thin-layer chromatography (TLC) was carried out on Merck aluminium foil backed sheets precoated with 0.2 mm Kielselgel 60 F_{254}. The eluent used is specified in each case. The spots were visualised by UV irradiation (λ=254 nm) and by staining with $KMnO_4$ solution followed by heating. Retention factors (R_f) are quoted to the nearest 0.01. Flash column chromatography was performed on Kielselgel 60 silica gel (230-400 mesh particle size). Melting points were measured using a Stuart Scientific SMP1 melting point instrument and are uncorrected. Infrared spectra were recorded on a Bruker Tensor 27 FT-IR spectrometer equipped with an attached Pike Miracle attenuated total reflectance (ATR) module. Absorption maxima (v_{max}) are reported in wavenumbers (cm^{-1}) and only selected peaks are reported. ^{1}H-NMR spectra were recorded at 200 MHz, 400 MHz and 500 MHz, ^{13}C-NMR spectra were recorded at 101 MHz or 126 MHz and ^{19}F-NMR spectra were recorded at 377 MHz or 471 MHz using either a Bruker DPX200, AVIIIHD 400 and AVIIIHD 500. Chemical shifts (δ_H, δ_F and δ_C) are reported in parts per million (ppm) downfield from TMS and are referenced to the residual solvent peak (in some cases, it was not possible to reference the chloroform peak in the ^{1}H-NMR spectra due to it being obscured by aromatic protons). Coupling constants (*J*) are quoted in Hertz (Hz). Data is reported in this format: chemical shift, multiplicity (s = singlet, d = doublet, t = triplet, q = quartet, qn = quintet, dd = double doublet, dt = double triplet, dq = double quartet, m = multiplet, br = broad and app = apparent), integration, coupling constant, and assignment. Two-dimensional COSY, HSQC, HMBC and NOESY experiments were recorded at 400 MHz and 500 MHz and nOe experiments were recorded at 500 MHz. Assignments of the spectra were made with ^{1}H, COSY, HSQC, HMBC, ^{13}C and DEPT-135 experiments and stereochemistry were assigned on the basis of nOe, NOESY or X-ray crystallography. Low resolution mass spectra (*m/z*) were recorded on an Agilent 6120 spectrometer or a Waters LCT Premier XE spectrometer using

electrospray ionisation (ESI). Selected peaks are reported in Daltons and their intensities given as percentages of the base peak. High resolution mass spectra (HRMS) were recorded on a Bruker microTOF (ESI) or on an Agilent 7200 Q-TOF (EI or CI). Crystals for X-ray crystallography were grown from slow vapour diffusion of petroleum ether 40:60 into a solution of compound dissolved in minimal EtOAc at room temperature. Low temperature[1] single-crystal X-ray diffraction data were collected using a (Rigaku) Oxford Diffraction SuperNova diffractometer. Raw frame data were collected and reduced using CrysAlisPro and the structures were solved using 'Superflip'[2] before refinement with CRYSTALS[3]. Heating of reactions was achieved using an oil bath and electric immersion heater.

Antibacterial Assay

Screening of compounds were performed by Oxford Antibiotic Group, Austria. For MIC determination by broth dilution assay, the samples were tested in a primary 96 well plates screening assay, in duplicate. The compounds were diluted in Mueller Hinton Broth (MHB) for bacterial screening to a stock solution of 1000 µg/mL, serial diluted and overlaid with a microbe solution in a concentration of 10^4 CFU/ml. The plates were incubated for 24 h at 35 °C, after which MIC values were read from the plates.

Cytotoxicity Assay

For cytotoxicity testing, the synthesized compounds were tested against four different cell lines: HeLa, HEK 293, MDCK and CaCo. The cells were seeded in a 96 well plate and incubated until a confluence of 80% was achieved (under physiological conditions - 37°C, 5% CO_2 and 95% humidity). The samples were tested by serial dilution in triplicates with starting concentration of 250 µg/mL. After 24 and 48 hours the survival of cells was evaluated by microscope and measured with Alamar blue. IC_{50} values were obtained from the calibration curves.

General procedure for the synthesis of *threo*-phenylserines 3a-e:[4]

Method A: To a stirring solution of 3 M NaOH (aq.) (1.5 eq.) at rt was added glycine (0.02-0.05 mol, 1.0 eq.) and the solution was stirred for 10 min. Benzaldehyde (2.1 eq.) was added and the solution was stirred until a solid condensation cake formed. The solid was broken apart after 48-72 h at rt and 3 M HCl (aq.) was added to acidify the solution to pH 1 and the mixture was stirred until the solid was consumed to give a clear solution. The solution was separated between Et_2O and water and the aqueous layer was reduced to dryness *in-vacuo* to give β-arylserine **1a** as a HCl salt that was contaminated with NaCl and was used without further purification.

Method B: To a stirring solution of 3 M KOH (ethanol) (1.5 eq.) at rt was added glycine (0.02-0.05 mol, 1.0 eq.) and the solution was stirred for 10 min (glycine tended to be sparingly soluble in ethanolic solution). Aldehyde (2.1 eq.) was then added, and the solution was stirred until a condensation cake formed. The solid was broken apart after 48-72 h at rt and 3 M HCl (aq.) was added to acidify the solution to pH 1 and the mixture was stirred until the solid was consumed to give a clear solution. The solution was separated between Et_2O and water and the aqueous layer was reduced to dryness *in-vacuo* to give β-arylserines **1a-e** as HCl salts that were contaminated with KCl and was used without further purification.

(2*S,3*R**)-2-Amino-3-hydroxy-3-phenylpropanoic acid hydrochloride salt 3a**

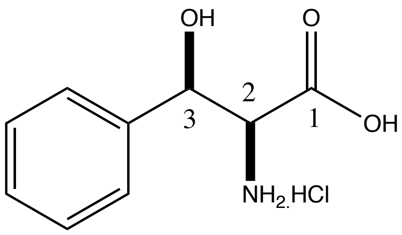

Prepared according to General Method A: glycine (3.75 g, 0.05 mol), benzaldehyde (11.7 g, 0.11 mol), aq NaOH (25 mL, 0.075 mol), Yield (9.12 g, Quantitative); Off-white solid; m.p. 159-163°C (lit.[4] 150-152°C); ν_{max}/cm^{-1} 3033 (O-H/N-H), 1725 (C=O); Diastereomeric ratio between *threo:erythro* 93:7; $\delta_{H\,(Threo)}$ (400 MHz, D_2O) 7.51-7.28 (m, 5H, Ar-H), 5.37 (d, 1H, *J*=4.0 Hz, H3), 4.07 (d, 1H, *J*=4.1 Hz, H2); $^{13}C\{^1H\}$ $\delta_{C\,(Threo)}$ (101 MHz, D_2O) 171.4 (C1), 138.6 (Ar-C), 129.0 (Ar-C), 128.7 (Ar-C), 125.9 (Ar-C), 71.0 (C3), 60.2 (C2); LRMS (ESI$^-$) *m/z*: 180.1 ([M – H]$^-$ 96%); HRMS (ESI$^-$) *m/z*: [M – H]$^-$ calcd. for $C_9H_{10}NO_3$, 180.0666, found 180.0659.

(2*S,3*R**)-2-Amino-3-hydroxy-3-(4-methylphenyl)propanoic acid hydrochloride salt 3b**

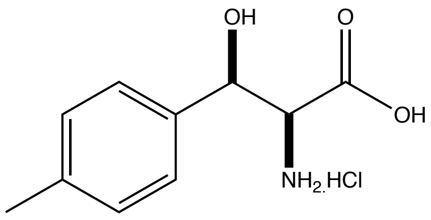

Prepared according to the General Method B: glycine (3.75 g, 0.05 mol), benzaldehyde (13.2 g, 0.11 mol), aq NaOH (25 mL, 0.075 mol), Yield (10.55 g, Quantitative); Off-white solid; m.p. 165-169°C; ν_{max}/cm^{-1} 3331 (O-H), 3038 (N-H), 1734 (C=O); Diastereomeric ratio between *threo:erythro* 19:1; $\delta_{H\,(Threo)}$ (400 MHz, D_2O) 7.40 (d, 2H, *J*=8.1 Hz, Ar-H), 7.34 (d, 2H, *J*=8.2 Hz, Ar-H), 5.40 (d, 1H, *J*=4.2 Hz, H3), 4.26 (d, 1H, *J*=4.2 Hz, H2), 2.37 (s, 3H, CH_3); $^{13}C\{^1H\}$ $\delta_{C\,(Threo)}$ (101 MHz, D_2O) 170.3 (C1), 139.2 (Ar-C), 135.1 (Ar-C), 129.6 (Ar-C), 125.9 (Ar-C), 70.6 (C3), 59.4 (C2), 20.3 (CH_3); LRMS (ESI$^+$) *m/z*: 234.1 ([M + K]$^+$ 55%), 391.2 ([2M + H]$^+$ 21%), 429.2 ([2M + K]$^+$ 100%); LRMS (ESI$^-$) *m/z*: 194.1 ([M – H]$^-$ 97%); HRMS (ESI$^+$) *m/z*: [M + H]$^+$ calcd. for $C_{10}H_{14}NO_3$, 196.0968, found 196.0970.

(2*S,3*R**)-2-Amino-3-hydroxy-3-(4-fluorophenyl)propanoic acid hydrochloride salt 3c**

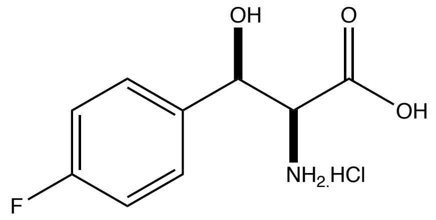

Prepared according to the General Method B: glycine (3.0 g, 0.04 mol), benzaldehyde (9.9 g, 0.08 mol), aq NaOH (20 mL, 0.06 mol), Yield (3.61 g, 76%); Off-white solid; m.p. 167-170°C (lit.[5] 162-164°C); ν_{max}/cm^{-1} 3324 (O-H), 2892 (N-H), 1735 (C=O); Diastereomeric ratio between *threo:erythro* 84:16; $\delta_{H\ (Threo)}$ (400 MHz, D_2O) 7.43 (m, 2H, Ar-H), 7.17 (m, 2H, Ar-H), 5.35 (d, 1H, *J*=4.2 Hz, H3), 4.10 (d, 1H, *J*=4.3 Hz, H2),; $^{13}C\{^1H\}$ $\delta_{C\ (Threo)}$ (101 MHz, D_2O) 170.8 (C1), 162.5 (d, *J*=244.3 Hz, Ar-C), 134.4 (d, *J*=2.9 Hz, Ar-C), 127.8 (d, *J*=8.6 Hz, Ar-C), 115.7 (d, *J*=21.9 Hz, Ar-C), 70.4 (C3), 59.8 (C2); $\delta_{F\ (Threo)}$ (377 MHz, D_2O) -114.19; LRMS (ESI$^+$) *m/z*: 238.1 ([M + K]$^+$ 58%), 437.1 ([2M + K]$^+$ 95%); LRMS (ESI$^-$) *m/z*: 198.0 ([M – H]$^-$ 95%); HRMS (ESI$^+$) *m/z*: [M + H]$^+$ calcd. for $C_9H_{11}NO_3F$, 200.0717, found 200.0720.

(2*S,3*R**)-2-Amino-3-hydroxy-3-(4-chlorophenyl)propanoic acid hydrochloride salt 3d**

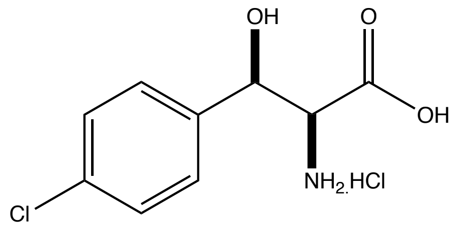

Prepared according to the General Method B: glycine (2.25 g, 0.03 mol), benzaldehyde (8.4 g, 0.06 mol), aq NaOH (15 mL, 0.045 mol), Yield (6.98 g, 68%); Off-white solid; m.p. 171-176°C; ν_{max}/cm^{-1} 3316 (O-H), 3064 (N-H), 1733 (C=O); Diastereomeric ratio between *threo:erythro* 9:1; $\delta_{H\ (Threo)}$ (400 MHz, D_2O) 7.46 (d, 2H, *J*=8.7 Hz, Ar-H), 7.42 (d, 2H, *J*=8.7 Hz, Ar-H), 5.39 (d, 1H, *J*=4.0 Hz, H3), 4.22 (d, 1H, *J*=4.0 Hz, H2); $^{13}C\{^1H\}$ $\delta_{C\ (Threo)}$ (101 MHz, D_2O) 170.2 (C1), 136.9 (Ar-C), 133.9 (Ar-C), 129.0 (Ar-C), 127.5 (Ar-C), 70.1 (C3), 59.2 (C2); LRMS (ESI$^-$) *m/z*: 214.0 ([M^{35} – H]$^-$ 97%), 216.0 ([M^{37} – H]$^-$ 32%); HRMS (ESI$^-$) *m/z*: [M^{35} – H]$^-$ calcd. for $C_9H_9NO_3Cl$, 214.0276, found 214.0273; [M^{37} – H]$^-$ calcd. for $C_9H_9NO_3Cl$, 216.0247, found 216.0243.

(2*S,3*R**)-2-Amino-3-hydroxy-3-(4-bromophenyl)propanoic acid hydrochloride salt 3e**

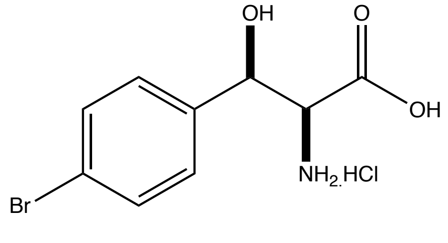

Prepared according to the General Method B: glycine (1.5 g, 0.02 mol), benzaldehyde (7.4 g, 0.04 mol), aq NaOH (10 mL, 0.03 mol), Yield (6.33 g, Quantitative); Off-white solid; m.p. 190-193°C; ν_{max}/cm^{-1} 3324 (O-H), 3031 (N-H), 1740 (C=O); Diastereomeric ratio between *threo:erythro* 94:6; $\delta_{H\ (Threo)}$ (400 MHz, D_2O) 7.58 (d, 2H, *J*=8.5 Hz, Ar-H), 7.34 (d, 2H, *J*=8.6 Hz, Ar-H), 5.38 (d, 1H, *J*=3.8 Hz, H3), 4.28 (d, 1H, *J*=3.9 Hz, H2); $^{13}C\{^1H\}$ $\delta_{C\ (Threo)}$ (101 MHz, D_2O) 169.7 (C1), 137.1 (Ar-C), 132.0 (Ar-C), 127.8 (Ar-C),

122.2 (Ar-C), 69.9 (C3), 58.8 (C2); LRMS (ESI$^+$) *m/z*: 260.0 ([M^{79} + H]$^+$ 42%), 262.0 ([M^{81} + H]$^+$ 41%); LRMS (ESI$^-$) *m/z*: 258.0 ([M^{79} – H]$^-$ 97%), 260.0 ([M^{81} – H]$^-$ 97%); HRMS (ESI$^+$) *m/z*: [M^{79} + H]$^+$ calcd. for $C_9H_{11}NO_3Br$, 259.9917, found 259.9919; [M^{81} + H]$^+$ calcd. for $C_9H_{11}NO_3Br$, 261.9896, found 261.9898.

General procedure for the synthesis of amino ester hydrochlorides 5a-f:[6]

$SOCl_2$ (1.2 eq.) was added dropwise to a stirring solution of amino acid **3a-e** (0.03-0.05 mol, 1.0 eq.) in MeOH (30 – 50 mL) at 0°C and left to stir for 15 mins at this temperature. The mixture was then refluxed overnight. The reaction was then stopped and left to cool to rt and then the solvent was evaporated under reduced pressure to give the methyl/ethyl ester hydrochloride salt **5a-f** and was used without further purification.

Methyl (2*S,3*R**)-2-amino-3-hydroxy-3-phenylpropanoate hydrochloride salt 5a**

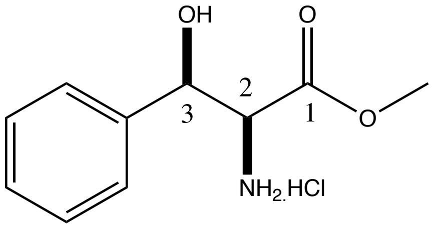

Prepared according to the General Method : amino acid (9.0 g, 0.05 mol), thionyl chloride (7.1 g, 0.06 mol), methanol (50 mL), Yield (9.31 g, 96%); Off-white/cream solid; m.p. 168-171°C (lit.[7] 179-180°C); ν_{max}/cm^{-1} 3321 (O-H), 2997 (N-H), 1744 (C=O); $\delta_{H\ (Threo)}$ (400 MHz, D_2O) 7.46-7.22 (m, 5H, Ar-H), 5.40 (d, 1H, *J*=4.0 Hz, H3), 4.37 (d, 1H, *J*=4.0 Hz, H2), 3.79 (s, 3H, OCH_3); $^{13}C\{^1H\}$ $\delta_{C\ (Threo)}$ (101 MHz, D_2O) 168.5 (C1), 137.5 (Ar-C), 129.1 (Ar-C), 129.0 (Ar-C), 125.8 (Ar-C), 70.5 (C3), 59.0 (C2), 53.7 (OCH_3); LRMS (ESI$^+$) *m/z*: 196.1 ([M + H]$^+$ 18%), 218.1 ([M + Na]$^+$ 9%), 391.2 ([2M + H]$^+$ 100%); HRMS (ESI$^+$) *m/z*: [M + H]$^+$ calcd. for $C_{10}H_{14}NO_3$, 196.0968, found 196.0971.

Methyl (2*S,3*R**)-2-amino-3-hydroxy-3-(4-methylphenyl)propanoate hydrochloride salt 5b**

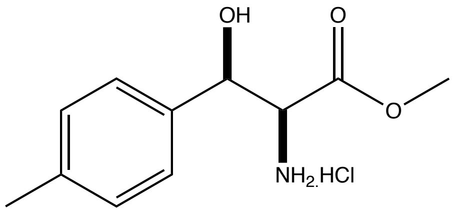

Prepared according to the General Method: amino acid (8.0 g, 0.04 mol), thionyl chloride (5.9 g, 0.05 mol), methanol (40 mL), Yield (10.86 g, 98%); Off-white/cream solid; m.p. 180-183°C; ν_{max}/cm^{-1} 3347 (O-H), 3014 (N-H), 2913 (C-H), 1758 (C=O); $\delta_{H\ (Threo)}$ (400 MHz, D_2O) 7.42 (d, 2H, *J*=8.3 Hz, Ar-H), 7.38 (d, 2H, *J*=8.1 Hz, Ar-H), 5.44 (d, 1H, *J*=4.5 Hz, H3), 4.46 (d, 1H, *J*=4.5 Hz, H2), 3.90 (s, 3H, OCH_3), 2.41 (s, 3H, CH_3); $^{13}C\{^1H\}$ $\delta_{C\ (Threo)}$ (101 MHz, D_2O) 168.6 (C1), 139.4 (Ar-C), 134.6 (Ar-C), 129.7 (Ar-C), 126.0

(Ar-C), 70.6 (C3), 59.1 (C2), 53.8 (OCH_3), 20.4 (CH_3); LRMS (ESI$^+$) *m/z*: 248.1 ([M + K]$^+$ 39%), 419.2 ([2M + H]$^+$ 100%); HRMS (ESI$^+$) *m/z*: [M + Na]$^+$ calcd. for $C_{11}H_{15}NO_3Na$, 232.0944, found 232.0947.

Methyl (2*S,3*R**)-2-amino-3-hydroxy-3-(4-fluorophenyl)propanoate hydrochloride salt 5c**

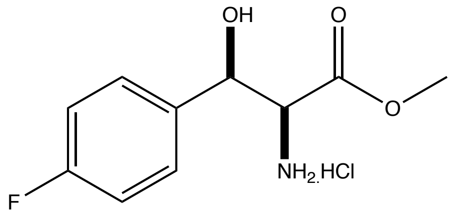

Prepared according to the General Method : amino acid (6.0 g, 0.03 mol), thionyl chloride (4.2 g, 0.036 mol), methanol (30 mL), Yield (1.16 g, 97%); Off-white/cream solid; m.p. 167-169°C; ν_{max}/cm^{-1} 3313 (O-H), 3002 (N-H), 2910 (C-H), 1744 (C=O); $\delta_{H\ (Threo)}$ (400 MHz, D_2O) 7.49 (dd, 2H, *J*=8.6 Hz, 5.4 Hz, Ar-H), 7.21 (app. t, 2H, *J*=8.9 Hz, Ar-H), 5.45 (d, 1H, *J*=4.1 Hz, H3), 4.43 (d, 1H, *J*=4.1 Hz, H2), 3.85 (s, 3H, OCH_3); $^{13}C\{^1H\}$ $\delta_{C\ (Threo)}$ (101 MHz, D_2O) 168.5 (C1), 162.6 (d, *J*=244.7 Hz, Ar-C), 133.5 (d, *J*=3.0 Hz, Ar-C), 127.9 (d, *J*=8.5 Hz, Ar-C), 115.9 (d, *J*=21.8 Hz, Ar-C), 70.0 (C3), 59.0 (C2), 53.8 (OCH_3); $\delta_{F\ (Threo)}$ (377 MHz, D_2O) -113.73; LRMS (ESI$^+$) *m/z*: 252.1 ([M + K]$^+$ 41%), 427.2 ([2M + H]$^+$ 100%); HRMS (ESI$^+$) *m/z*: [M + H]$^+$ calcd. for $C_{10}H_{13}NO_3F$, 214.0874, found 214.0885.

Methyl (2*S,3*R**)-2-amino-3-hydroxy-3-(4-chlorophenyl)propanoate hydrochloride salt 5d**

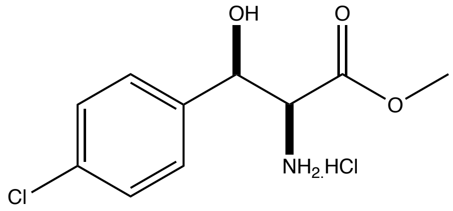

Prepared according to the General Method : amino acid (8.6 g, 0.04 mol), thionyl chloride (5.9 g, 0.05 mol), methanol (40 mL), Yield (7.08 g, Quantitative); Off-white/cream solid; m.p. 184-186°C; ν_{max}/cm^{-1} 3319 (O-H), 2998 (N-H), 2912 (C-H), 1744 (C=O); $\delta_{H\ (Threo)}$ (400 MHz, D_2O) 7.51 (d, 2H, *J*=8.9 Hz, Ar-H), 7.47 (d, 2H, *J*=8.7 Hz, Ar-H), 5.46 (d, 1H, *J*=4.2 Hz, H3), 4.46 (d, 1H, *J*=4.2 Hz, H2), 3.87 (s, 3H, OCH_3); $^{13}C\{^1H\}$ $\delta_{C\ (Threo)}$ (101 MHz, D_2O) 168.5 (C1), 136.4 (Ar-C), 134.1 (Ar-C), 129.1 (Ar-C), 127.6 (Ar-C), 70.0 (C3), 58.9 (C2), 53.9 (OCH_3); LRMS (ESI$^+$) *m/z*: 268.0 ([M^{35} + K]$^+$ 41%), 270.0 ([M^{37} + K]$^+$ 15%); HRMS (ESI$^+$) *m/z*: [M^{35} + H]$^+$ calcd. for $C_{10}H_{13}NO_3Cl$, 230.0578, found 230.0579; [M^{37} + H]$^+$ calcd. for $C_{10}H_{13}NO_3Cl$, 232.0549, found 232.0550.

Methyl (2*S,3*R**)-2-amino-3-hydroxy-3-(4-bromophenyl)propanoate hydrochloride salt 5e**

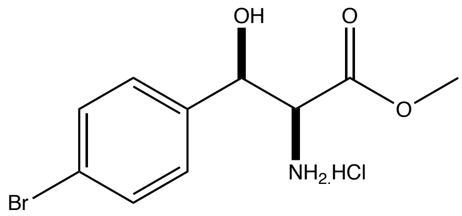

Prepared according to the General Method : amino acid (10.4 g, 0.04 mol), thionyl chloride (5.9 g, 0.05 mol), methanol (40 mL), Yield (12.02 g, Quantitative); Off-white/cream solid; m.p. 175-177°C; ν_{max}/cm^{-1} 3316 (O-H), 2997 (N-H), 2908 (C-H), 1743 (C=O); $\delta_{H\ (Threo)}$ (400 MHz, D_2O) 7.62 (d, 2H, *J*=8.5 Hz, Ar-H), 7.37 (d, 2H, *J*=8.4 Hz, Ar-H), 5.40 (d, 1H, *J*=4.1 Hz, H3), 4.41 (d, 1H, *J*=4.1 Hz, H2), 3.83 (s, 3H, OCH_3); $^{13}C\{^1H\}$ $\delta_{C\ (Threo)}$ (101 MHz, D_2O) 168.4 (C1), 136.8 (Ar-C), 132.0 (Ar-C), 127.9 (Ar-C), 122.3 (Ar-C), 70.0 (C3), 58.8 (C2), 53.8 (OCH_3); LRMS (ESI$^+$) *m/z*: 274.0 ([M^{79} + H]$^+$ 42%), 276.0 ([M^{81} + H]$^+$ 43%), 547.0 ([2M^{79} + H]$^+$ 82%), 549.0 ([2M^{79+81} + H]$^+$ 100%), 551.0 ([2M^{81} + H]$^+$ 66%); HRMS (ESI$^+$) *m/z*: [M^{79} + H]$^+$ calcd. for $C_{10}H_{13}NO_3Br$, 274.0073, found 274.0073; [M^{81} + H]$^+$ calcd. for $C_{10}H_{13}NO_3Br$, 276.0053, found 276.0053.

Methyl (4*S,5*R**)-2-oxo-5-phenyloxazolidine-4-carboxylate 6**[9]

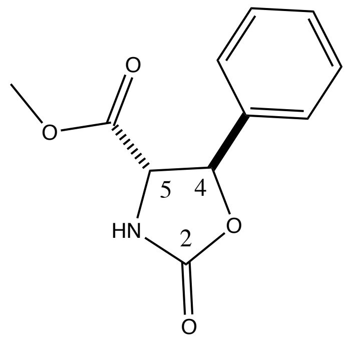

Methyl ester hydrochloride salt **(±) 5a** (46.5 mg, 0.24 mmol) was suspended in THF (20 mL) and triethylamine (1.2 eq.) was added, and the solution was stirred at rt for 5 mins. CDI (1.8 eq.) was then added, and the solution was stirred overnight at rt. The mixture was then quenched with sat. NH_4Cl (aq.), extracted with EtOAc, washed with brine, dried over Na_2SO_4, filtered, reduced *in-vacuo* and then purified by flash column chromatography to furnish oxazolidinone **(±) 6**.Yield (58 mg, 30%); Colourless oil; R_f (50% EtOAc in Pet. Ether 40:60) 0.33; ν_{max}/cm^{-1} 3274 (N-H), 1751 (C=O); δ_H (400 MHz, $CDCl_3$) 7.43-7.36 (m, 5H, Ar-H), 6.88 (br s, 1H, NH), 5.63 (d, 1H, *J*=5.0 Hz, H4), 4.31 (dd, 1H, *J*=5.0 Hz, 0.7 Hz, H5), 3.84 (s, 3H, OCH_3); $^{13}C\{^1H\}$ δ_C (101 MHz, $CDCl_3$) 170.3 (C=O), 158.6 (C2), 138.1 (Ar-C), 129.2 (Ar-C), 129.1 (Ar-C), 125.5 (Ar-C), 79.5 (C4), 61.5 (C5), 53.3 (OCH_3); LRMS (ESI$^+$) *m/z*: 244.0 ([M + Na]$^+$ 97%), 465.2 ([2M + Na]$^+$ 28%); HRMS (ESI$^+$) *m/z*: [M + Na]$^+$ calcd. for $C_{11}H_{11}NO_4Na$, 244.0580, found 244.0582.

General procedure for the synthesis of oxazolidines 7a-e:[10-11]

Aminoester hydrochloride salt **(±) 5a-e** (10.0 - 20.0 mmol, 1.0 eq.) was suspended in petroleum ether 40:60 (10 – 50 mL) and cooled to 0°C. Triethylamine (1.2 eq.) and trimethylacetaldehyde (1.1 eq.) was added to this reaction mixture and was heated to more than 100°C with continuous removal of water using a Dean-Stark apparatus overnight. The precipitate was then filtered and washed with Et_2O. The combined filtrates were

concentrated under reduced pressure to yield the desired products **7a-f** and was used without further purification.

Methyl (4*S,5*R**)-2-(*tert*-butyl)-5-phenyloxazolidine-4-carboxylate 7a**

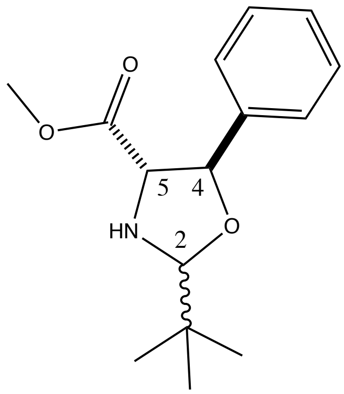

Prepared according to the General Method : amino ester (352 mg, 1.8 mmol), trimethylacetaldehyde (170 mg, 2.0 mmol), triethylamine (216 mg), petroleum ether (20 mL), Yield (472 mg, 82%); Yellow oil; ν_{max}/cm^{-1} 3337 (N-H), 2956 (C-H), 2907 (C-H), 2871 (C-H), 1739 (C=O); 1.1:1 mixture of diastereomers at the C2-position; $\delta_{H\ (2,5\text{-}trans\ \text{diastereomer})}$ (400 MHz, $CDCl_3$) 7.34-7.16 (m, 5H, Ar-H), 4.74 (d, 1H, *J*=6.6 Hz, H4), 4.42 (s, 1H, H2), 3.68 (s, 3H, OCH_3), 3.63 (d, 1H, *J*=6.6 Hz, H5), 0.98 (s, 9H, $C(CH_3)_3$); $\delta_{H\ (2,5\text{-}cis\ \text{diastereomer})}$ (400 MHz, $CDCl_3$) 7.34-7.16 (m, 5H, Ar-H), 4.67 (d, 1H, *J*=7.0 Hz, H4), 4.42 (s, 1H, H2), 3.77 (d, 1H, *J*=7.0 Hz, H5), 3.73 (s, 3H, OCH_3), 1.00 (s, 9H, $C(CH_3)_3$),; $^{13}C\{^1H\}$ δ_C (101 MHz, $CDCl_3$) 172.8 and 172.0 (C=O), 141.3 and 139.8 (Ar-C), 128.7 and 128.6 (Ar-C), 128.2 and 127.9 (Ar-C), 126.2 and 125.8 (Ar-C), 100.3 and 99.4 (C2), 82.4 and 81.4 (C4), 68.7 and 67.4 (C5), 52.7 and 52.5 (OCH_3), 34.5 and 33.7 ($C(CH_3)_3$), 25.4 and 25.3 ($(CH_3)_3$); LRMS (ESI$^+$) *m/z*: 264.1 ([M + H]$^+$ 55%), 286.1 ([M + Na]$^+$ 60%); HRMS (ESI$^+$) *m/z*: [M + H]$^+$ calcd. for $C_{15}H_{22}NO_3$, 264.1594, found 264.1595.

Methyl (4*S,5*R**)-2-(*tert*-butyl)-5-(4-methylphenyl)oxazolidine-4-carboxylate 7b**

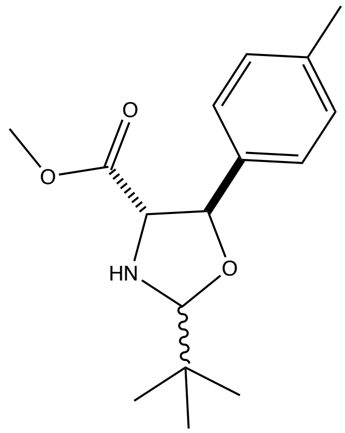

Prepared according to the General Method : amino ester (383 mg, 1.65 mmol), trimethylacetaldehyde (150 mg, 1.8 mmol), triethylamine (200 mg), petroleum ether (15 mL), Yield (461 mg, 81%); Yellow oil; ν_{max}/cm^{-1} 3338 (N-H), 2956 (C-H), 2908 (C-H), 2870 (C-H), 1740 (C=O); 1.1:1 mixture of diastereomers at the C2-position; $\delta_{H\ (2,5\text{-}trans\ \text{diastereomer})}$ (400 MHz, $CDCl_3$) 7.21-7.07 (m, 4H, Ar-H), 4.69 (d, 1H, *J*=6.7 Hz, H4), 4.41 (s, 1H, H2), 3.67 (s, 3H, OCH_3), 3.61 (d, 1H, *J*=6.7 Hz, H5), 2.27 (s, 3H, CH_3), 0.96 (s, 9H, $C(CH_3)_3$); $\delta_{H\ (2,5\text{-}cis\ \text{diastereomer})}$ (400 MHz, $CDCl_3$) 7.21-7.07 (m, 4H, Ar-H), 4.63 (d, 1H, *J*=7.0 Hz, H4), 4.41 (s, 1H, H2), 3.77 (d, 1H, *J*=7.0 Hz, H5), 3.71 (s, 3H, OCH_3), 2.27 (s, 3H, CH_3), 0.99 (s, 9H, $C(CH_3)_3$); $^{13}C\{^1H\}$ δ_C (101 MHz, $CDCl_3$) 172.9 and 172.0 (C=O), 138.3 and 137.9 (Ar-C),

137.6 and 136.8 (Ar-C), 129.4 and 129.3 (Ar-C), 126.2 and 125.8 (Ar-C), 100.1 and 99.3 (C2), 82.5 and 81.5 (C4), 68.7 and 67.4 (C5), 52.6 and 52.4 (OCH_3), 34.6 and 33.8 ($C(CH_3)_3$), 25.4 and 25.3 ($(CH_3)_3$), 21.3 and 21.3 (CH_3); LRMS (ESI$^+$) *m/z*: 278.2 ($[M + H]^+$ 12%), 300.1 ($[M + Na]^+$ 8%); HRMS (ESI$^+$) *m/z*: $[M + H]^+$ calcd. for $C_{16}H_{24}NO_3$, 278.1751, found 278.1751.

Methyl (4*S,5*R**)-2-(*tert*-butyl)-5-(4-fluorophenyl)oxazolidine-4-carboxylate 7c**

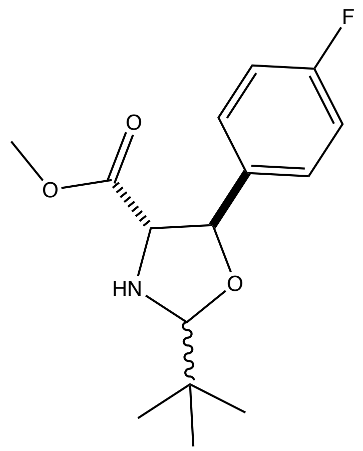

Prepared according to the General Method: amino ester (3.4 g, 16.7 mmol), trimethylacetaldehyde (1.6 g, 18.4 mmol), triethylamine (2.0 g), petroleum ether (50 mL), Yield (4.72 g, 70%); Yellow oil; ν_{max}/cm^{-1} 3335 (N-H), 2957 (C-H), 2907 (C-H), 2872 (C-H), 1740 (C=O); 1.1:1 mixture of diastereomers at the C2-position; $\delta_{H\ (2,5\text{-}trans\ \text{diastereomer})}$ (400 MHz, $CDCl_3$) 7.31-7.15 (m, 2H, Ar-H), 7.00-6.89 (m, 2H, Ar-H), 4.70 (d, 1H, *J*=6.7 Hz, H4), 4.41 (s, 1H, H2), 3.68 (s, 3H, OCH_3), 3.58 (d, 1H, *J*=6.7 Hz, H5), 0.96 (s, 9H, $C(CH_3)_3$); $\delta_{H\ (2,5\text{-}cis\ \text{diastereomer})}$ (400 MHz, $CDCl_3$) 7.31-7.15 (m, 2H, Ar-H), 7.00-6.89 (m, 2H, Ar-H), 4.62 (d, 1H, *J*=7.0 Hz, H4), 4.40 (br s, 1H, H2), 3.72 (s, 3H, OCH_3), 3.70 (m, 1H, H5), 0.99 (s, 9H, $C(CH_3)_3$); $^{13}C\{^1H\}$ δ_C (101 MHz, $CDCl_3$) 172.6 and 171.7 (C=O), 162.6 (d, *J*=245.9 Hz, Ar-C) and 162.4 (d, *J*=245.6 Hz, Ar-C), 137.0 (d, *J*=3.1 Hz, Ar-C) and 135.5 (d, *J*=3.1 Hz, Ar-C), 127.9 (d, *J*=8.4 Hz, Ar-C) and 127.5 (d, *J*=8.2 Hz, Ar-C), 115.5 (d, *J*=21.8 Hz, Ar-C) and 115.5 (d, *J*=21.5 Hz, Ar-C), 100.2 and 99.3 (C2), 81.9 and 80.8 (C4), 68.8 and 67.3 (C5), 52.7 and 52.5 (OCH_3), 34.5 and 33.7 ($C(CH_3)_3$), 25.3 and 25.2 ($(CH_3)_3$); δ_F (377 MHz, $CDCl_3$) -114.16, -114.78; LRMS (ESI$^+$) *m/z*: 282.1 ($[M + H]^+$ 22%), 304.2 ($[M + Na]^+$ 6%); HRMS (ESI$^+$) *m/z*: $[M + H]^+$ calcd. for $C_{15}H_{21}NO_3F$, 282.1500, found 282.1501.

Methyl (4*S,5*R**)-2-(*tert*-butyl)-5-(4-chlorophenyl)oxazolidine-4-carboxylate 7d**

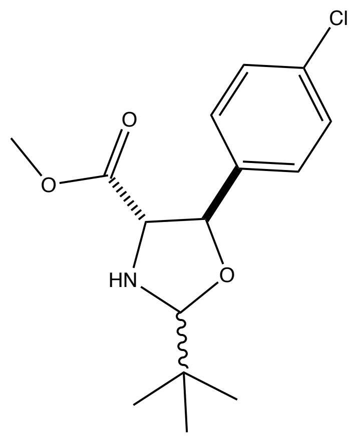

Prepared according to the General Method : amino ester (278 mg, 1.2 mmol), trimethylacetaldehyde (110 mg, 1.3 mmol), triethylamine (144 mg), petroleum ether (10 mL), Yield (354 mg, 62%); Yellow oil; ν_{max}/cm^{-1} 3335 (N-H), 2956 (C-H), 2908 (C-H), 2871 (C-H), 1741 (C=O); 1.2:1 mixture of diastereomers at the C2-position; $\delta_{H\ (2,5\text{-}trans\ \text{diastereomer})}$ (400 MHz, $CDCl_3$) 7.29-7.16 (m, 4H, Ar-H), 4.70 (d, 1H, *J*=6.6 Hz, H4), 4.41 (s, 1H, H2), 3.69 (s, 3H, OCH_3), 3.58 (d, 1H, *J*=6.6 Hz, H5), 0.96 (s, 9H, $C(CH_3)_3$); $\delta_{H\ (2,5\text{-}cis\ \text{diastereomer})}$ (400 MHz, $CDCl_3$) 7.29-7.16 (m, 4H, Ar-H), 4.62 (d, 1H, *J*=7.0 Hz, H4), 4.39 (br s, 1H, H2), 3.73 (s, 3H, OCH_3), 3.71 (d, 1H, *J*=7.2 Hz, H5), 0.99 (s, 9H, $C(CH_3)_3$); $^{13}C\{^1H\}$ δ_C (101 MHz, $CDCl_3$) 172.5 and 171.6 (C=O), 139.8 and 138.3 (Ar-C), 133.9 and 133.6 (Ar-C), 128.8 and 128.7 (Ar-C), 127.6 and 127.2 (Ar-C), 100.3 and 99.4 (C2), 81.7 and 80.7 (C4), 68.7 and 67.3 (C5), 52.8 and 52.6 (OCH_3), 34.5 and 33.7 ($C(CH_3)_3$), 25.3 and 25.3 ($(CH_3)_3$); LRMS (ESI^+) *m/z*: 298.0 ($[M^{35} + H]^+$ 28%), 300.1 ($[M^{37} + H]^+$ 9%); HRMS (ESI^+) *m/z*: $[M^{35} + H]^+$ calcd. for $C_{15}H_{21}NO_3Cl$, 298.1204, found 298.1205; $[M^{37} + H]^+$ calcd. for $C_{15}H_{21}NO_3Cl$, 300.1175, found 300.1175.

Methyl (4*S,5*R**)-2-(*tert*-butyl)-5-(4-bromophenyl)oxazolidine-4-carboxylate 7e**

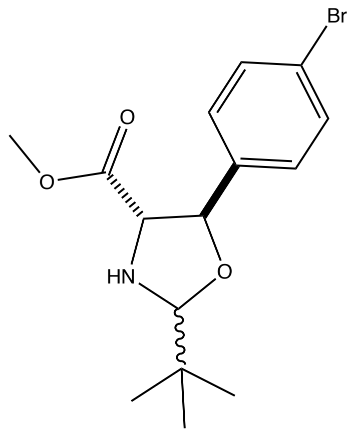

Prepared according to the General Method : amino ester (304 mg, 1.1 mmol), trimethylacetaldehyde (100 mg, 1.2 mmol), triethylamine (130 mg), petroleum ether (10 mL), Yield (385 mg, 69%); Yellow oil; ν_{max}/cm^{-1} 3332 (N-H), 2956 (C-H), 2907 (C-H), 2871 (C-H), 1740 (C=O); 1.2:1 mixture of diastereomers at the C2-position; $\delta_{H\ (2,5\text{-}trans\ \text{diastereomer})}$ (400 MHz, $CDCl_3$) 7.42-7.34 (m, 2H, Ar-H), 7.21-7.14 (m, 2H, Ar-H), 4.69 (d, 1H, *J*=6.6 Hz, H4), 4.41 (s, 1H, H2), 3.69 (s, 3H, OCH_3), 3.58 (d, 1H, *J*=6.6 Hz, H5), 0.96 (s, 9H, $C(CH_3)_3$),; $\delta_{H\ (2,5\text{-}cis\ \text{diastereomer})}$ (400 MHz, $CDCl_3$) 7.42-7.34 (m, 2H, Ar-H), 7.21-7.14 (m, 2H, Ar-H), 4.60 (d, 1H, *J*=6.9 Hz, H4), 4.38 (br s, 1H, H2), 3.73 (s, 3H, OCH_3), 3.71 (m, 1H, H5),0.99 (s, 9H, $C(CH_3)_3$); $^{13}C\{^1H\}$ δ_C (101 MHz, $CDCl_3$) 172.5 and 171.6 (C=O), 140.4 and 138.9 (Ar-C), 131.8 and 131.7 (Ar-C), 127.9 and 127.5 (Ar-C), 122.0 and 121.7 (Ar-C), 100.4 and 99.5 (C2), 81.7 and 80.7 (C4), 68.7 and 67.3 (C5), 52.8 and 52.6 (OCH_3), 34.5 and 33.7 ($C(CH_3)_3$), 25.3 and 25.3 ($(CH_3)_3$); LRMS (ESI^+) *m/z*:

342.0 ([M^{79} + H]$^+$ 19%), 344.0 ([M^{81} + H]$^+$ 19%); HRMS (ESI$^+$) *m/z*: [M^{79} + H]$^+$ calcd. for $C_{15}H_{21}NO_3Br$, 342.0699, found 342.0699; [M^{81} + H]$^+$ calcd. for $C_{15}H_{21}NO_3Br$, 344.0679, found 344.0679.

General procedure for the synthesis of *N*-acylated oxazolidines 8a-f/9a-e:[10]

To a stirring solution of oxazolidine **(±) 7a-e** (10 mmol, 1.0 eq.) in anhydrous DCM (50 mL) at 0°C was added ethyl malonyl chloride (1.1 eq.) and pyridine (1.2 eq.) dropwise. The mixture was stirred at 0°C for 10-20 min and then refluxed overnight. The mixture was then washed with sat. NH_4Cl (aq.), sat. $NaHCO_3$ (aq.), brine, dried over Na_2SO_4, filtered, concentrated under reduced pressure and purified by flash column chromatography to give *N*-acylated oxazolidines **8a-f/9a-e**.

Methyl (4*S,5*R**)-2-(*tert*-butyl)-3-(3-ethoxy-3-oxopropanoyl)-5-phenyloxazolidine-4-carboxylate 8a and 9a**

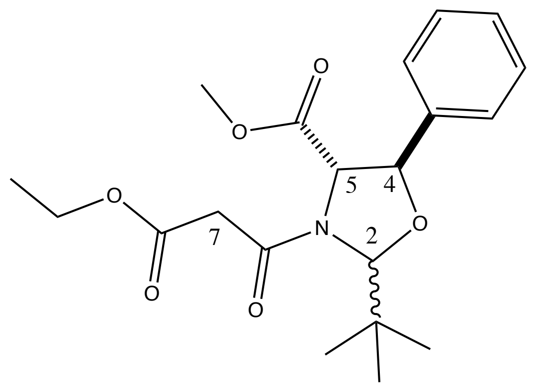

Prepared according to the General Method : oxazolidine (2.64 g, 10 mmol), ethyl malonyl chloride (1.65 g, 11 mmol), pyridine (1 mL), DCM (50 mL), Yield (3.74 g, 81%); Pale yellow oil; R_f (30% EtOAc in Pet. Ether 40:60) 0.48 $_{(8a)}$ and 0.25 $_{(9a)}$; ν_{max}/cm^{-1} $_{(8a)}$ 2958 (C-H), 1742 (C=O), 1675 (C=O); 65:35 diastereomeric ratio between **(±) 8a** and **(±) 9a**; $\delta_{H\,(8a)}$ (400 MHz, $CDCl_3$) 7.37-7.26 (m, 5H, Ar-H), 5.77 (s, 1H, H2), 5.69 (d, 1H, *J*=4.1 Hz, H4), 4.59 (d, 1H, *J*=4.2 Hz, H5), 4.05 (m, 2H, OCH_2CH_3), 3.84 (s, 3H, OCH_3), 3.47 (d, 1H, *J*=15.4 Hz, H7), 3.41 (d, 1H, *J*=15.4 Hz, H7), 1.15 (br t, 3H, *J*=6.4 Hz, OCH_2CH_3), 0.98 (s, 9H, $C(CH_3)_3$); $^{13}C\{^1H\}$ $\delta_{C\,(8a)}$ (101 MHz, $CDCl_3$) 170.1 (C=O), 167.0 (C=O), 166.8 (C=O), 140.3 (Ar-C), 128.9 (Ar-C), 128.6 (Ar-C), 125.8 (Ar-C), 98.0 (C2), 81.7 (C4), 66.9 (C5), 61.7 (OCH_2CH_3), 53.2 (OCH_3), 42.8 (C7), 38.4 ($C(CH_3)_3$), 25.9 ($(CH_3)_3$), 14.1 (OCH_2CH_3); $\delta_{H\,(9a)\,(203K,\ Major\ conformer)}$ (500 MHz, CD_2Cl_2) 7.43-7.38 (m, 5H, Ar-H), 5.36 (s, 1H, H2), 5.14 (d, 1H, *J*=9.2 Hz, H4), 4.17 (m, 2H, OCH_2CH_3), 4.00 (d, 1H, *J*=9.0 Hz, H5), 3.73 (s, 3H, OCH_3), 3.50 (m, 2H, H7), 1.27 (br m, 3H, OCH_2CH_3), 1.04 (s, 9H, $C(CH_3)_3$); $^{13}C\{^1H\}$ $\delta_{C\,(9a)\,(203K,\ Major\ conformer)}$ (126 MHz, CD_2Cl_2) 167.7 (C=O), 166.8 (C=O), 163.9 (C=O), 135.4 (Ar-C), 128.7 (Ar-C), 128.7 (Ar-C), 125.6 (Ar-C), 96.1 (C2), 82.2 (C4), 64.9 (C5), 61.7 (OCH_2CH_3), 53.1 (OCH_3), 41.7 (C7), 39.1 ($C(CH_3)_3$), 25.2 ($(CH_3)_3$), 13.8 (OCH_2CH_3); LRMS $_{(8a)}$ (ESI$^+$) *m/z*: 400.2 ([M + Na]$^+$ 97%); HRMS (ESI$^+$) *m/z*: [M + H]$^+$ calcd. for $C_{20}H_{28}NO_6$, 378.1911, found 378.1904.

Methyl (4*S,5*R**)-2-(*tert*-butyl)-3-(3-ethoxy-3-oxopropanoyl)-5-(4-methylphenyl)oxazolidine-4-carboxylate 8b and 9b**

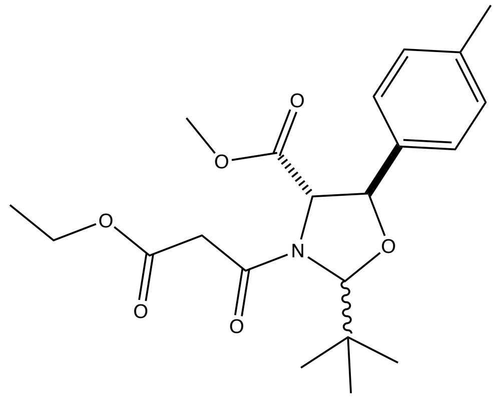

Prepared according to the General Method : oxazolidine (2.2 g, 8.0 mmol), ethyl malonyl chloride (1.65 g, 11 mmol), pyridine (1 mL), DCM (50 mL), Yield (3.45 g, 67%); Pale yellow oil; R_f (30% EtOAc in Pet. Ether 40:60) 0.53 $_{\mathbf{(8b)}}$ and 0.45 $_{\mathbf{(9b)}}$; ν_{max}/cm^{-1} $_{\mathbf{(8b)}}$ 2958 (C-H), 1740 (C=O), 1675 (C=O); 59:41 diastereomeric ratio between **(±) 8b** and **(±) 9b**; $\delta_{H\ \mathbf{(8b)}}$ (400 MHz, $CDCl_3$) 7.26 (d, 2H, *J*=8.1 Hz, Ar-H), 7.18 (d, 2H, *J*=7.9 Hz, Ar-H), 5.76 (s, 1H, H2), 5.65 (d, 1H, *J*=4.3 Hz, H4), 4.58 (d, 1H, *J*=4.3 Hz, H5), 4.09 (m, 2H, OCH_2CH_3), 3.85 (s, 3H, OCH_3), 3.46 (m, 2H, H7), 2.35 (s, 3H, CH_3), 1.19 (br t, 3H, *J*=7.1 Hz, OCH_2CH_3), 1.00 (s, 9H, $C(CH_3)_3$); $^{13}C\{^1H\}$ $\delta_{C\ \mathbf{(8b)}}$ (101 MHz, $CDCl_3$) 170.1 (C=O), 167.0 (C=O), 166.9 (C=O), 138.4 (Ar-C), 137.2 (Ar-C), 129.6 (Ar-C), 125.8 (Ar-C), 97.9 (C2), 81.7 (C4), 67.0 (C5), 61.7 (OCH_2CH_3), 53.1 (OCH_3), 42.8 (C7), 38.4 ($C(CH_3)_3$),25.9 ($(CH_3)_3$), 21.3 (CH_3), 14.1 (OCH_2CH_3); $\delta_{H\ \mathbf{(9b)}\ (203K,\ Major\ conformer)}$ (500 MHz, CD_2Cl_2) 7.22 (d, 2H, *J*=8.1 Hz, Ar-H), 7.18 (d, 2H, *J*=7.9 Hz, Ar-H), 5.28 (s, 1H, H2), 5.03 (d, 1H, *J*=9.2 Hz, H4), 4.11 (m, 2H, OCH_2CH_3), 3.93 (d, 1H, *J*=9.2 Hz, H5), 3.66 (s, 3H, OCH_3), 3.43 (m, 2H, H7), 2.31 (s, 3H, CH_3), 1.23 (br t, 3H, *J*=7.0 Hz, OCH_2CH_3), 0.98 (s, 9H, $C(CH_3)_3$); $^{13}C\{^1H\}$ $\delta_{C\ \mathbf{(9b)}\ (203K,\ Major\ conformer)}$ (126 MHz, CD_2Cl_2) 167.6 (C=O), 166.8 (C=O), 163.9 (C=O), 138.9 (Ar-C), 132.2 (Ar-C), 129.3 (Ar-C), 125.7 (Ar-C), 96.0 (C2), 82.3 (C4), 65.0 (C5), 61.7 (OCH_2CH_3), 53.1 (OCH_3), 41.7 (C7), 39.1 ($C(CH_3)_3$), 25.2 ($(CH_3)_3$), 21.0 (CH_3), 13.8 (OCH_2CH_3); LRMS $_{\mathbf{(8b)}}$ (ESI^+) *m/z*: 414.2 ($[M + Na]^+$ 95%); LRMS $_{\mathbf{(9b)}}$ (ESI^+) *m/z*: 414.2 ($[M + Na]^+$ 96%); HRMS (ESI^+) *m/z*: $[M + Na]^+$ calcd. for $C_{21}H_{29}NO_6Na$, 414.1887, found 414.1886.

Methyl (4*S,5*R**)-2-(*tert*-butyl)-3-(3-ethoxy-3-oxopropanoyl)-5-(4-fluorophenyl)oxazolidine-4-carboxylate 8c and 9c**

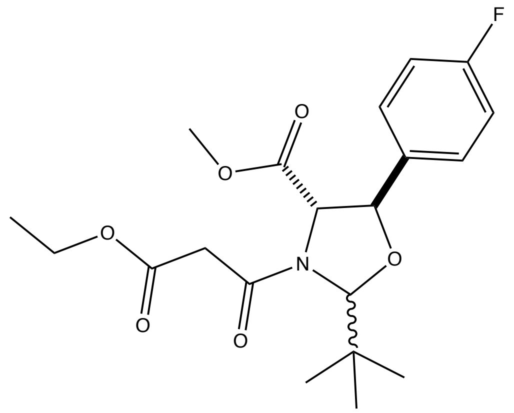

Prepared according to the General Method : oxazolidine (2.8 g, 10 mmol), ethyl malonyl chloride (1.65 g, 11 mmol), pyridine (1 mL), DCM (50 mL), Yield (3.90 g, 60%); Pale yellow oil; R_f (30% EtOAc in Pet. Ether 40:60) 0.55 $_{(8c)}$ and 0.38 $_{(9c)}$; ν_{max}/cm^{-1} $_{(8c)}$ 2959 (C-H), 1743 (C=O), 1675 (C=O); 59:41 diastereomeric ratio between **(±) 8c** and **(±) 9c**; $\delta_{H\,(8c)}$ (400 MHz, $CDCl_3$) 7.37 (dd, 2H, *J*=8.5 Hz, 5.3 Hz, Ar-H), 7.06 (app. t, 2H, *J*=8.6 Hz, Ar-H), 5.77 (s, 1H, H2),5.66 (d, 1H, *J*=4.4 Hz, H4), 4.57 (d, 1H, *J*=4.4 Hz, H5), 4.07 (m, 2H, OCH_2CH_3), 3.85 (s, 3H, OCH_3), 3.49 (d, 1H, *J*=15.4 Hz, H7), 3.42 (d, 1H, *J*=15.5 Hz, H7), 1.18 (br t, 3H, *J*=7.2 Hz, OCH_2CH_3), 0.98 (s, 9H, $C(CH_3)_3$); $^{13}C\{^1H\}$ $\delta_{C\,(8c)}$ (126 MHz, $CDCl_3$) 170.0 (C=O), 167.0 (C=O), 166.9 (C=O), 162.8 (d, *J*=247.1 Hz, Ar-C), 136.1 (d, *J*=3.2 Hz, Ar-C), 127.8 (d, *J*=8.4 Hz, Ar-C), 115.8 (d, *J*=21.6 Hz, Ar-C), 98.0 (C2), 81.3 (C4), 66.8 (C5), 61.8 (OCH_2CH_3), 53.3 (OCH_3), 42.7 (C7), 38.4 ($C(CH_3)_3$), 25.9 ($(CH_3)_3$), 14.1 (OCH_2CH_3); $\delta_{F\,(8c)}$ (471 MHz, $CDCl_3$) -113.43; $\delta_{H\,(9c)\,(203K,\ Major\ conformer)}$ (500 MHz, CD_2Cl_2) 7.32 (m, 2H, Ar-H), 7.06 (app. t, 2H, *J*=8.5 Hz, Ar-H), 5.27 (s, 1H, H2), 5.05 (d, 1H, *J*=9.2 Hz, H4), 4.09 (m, 2H, OCH_2CH_3), 3.91 (d, 1H, *J*=9.2 Hz, H5), 3.65 (s, 3H, OCH_3), 3.49 (d, 1H, *J*=15.9 Hz, H7), 3.36 (d, 1H, *J*=15.9 Hz, H7), 1.21 (br t, 3H, *J*=7.0 Hz, OCH_2CH_3), 0.96 (s, 9H, $C(CH_3)_3$); $^{13}C\{^1H\}$ $\delta_{C\,(9c)\,(213K,\ Major\ conformer)}$ (126 MHz, CD_2Cl_2) 167.6 (C=O), 166.8 (C=O), 164.1 (C=O), 162.5 (d, *J*=246.2 Hz, Ar-C), 131.3 (Ar-C), 127.8 (d, *J*=8.3 Hz, Ar-C), 115.7 (d, *J*=21.5 Hz, Ar-C), 96.1 (C2),81.7 (C4), 65.0 (C5), 61.8 (OCH_2CH_3), 53.2 (OCH_3), 41.7 (C7), 39.1 ($C(CH_3)_3$), 25.2 ($(CH_3)_3$), 13.8 (OCH_2CH_3); $\delta_{F\,(9c)\,(213K,\ Major\ conformer)}$ (471 MHz, CD_2Cl_2) -113.38; LRMS $_{(8c)}$ (ESI$^+$) *m/z*: 418.2 ([M + Na]$^+$ 96%); LRMS $_{(9c)}$ (ESI$^+$) *m/z*: 418.2 ([M + Na]$^+$ 97%); HRMS (ESI$^+$) *m/z*: [M + Na]$^+$ calcd. for $C_{20}H_{26}NO_6FNa$, 418.1636, found 418.1635.

Methyl (4*S,5*R**)-2-(*tert*-butyl)-5-(4-chlorophenyl)-3-(3-ethoxy-3-oxopropanoyl)oxazolidine-4-carboxylate 8d and 9d**

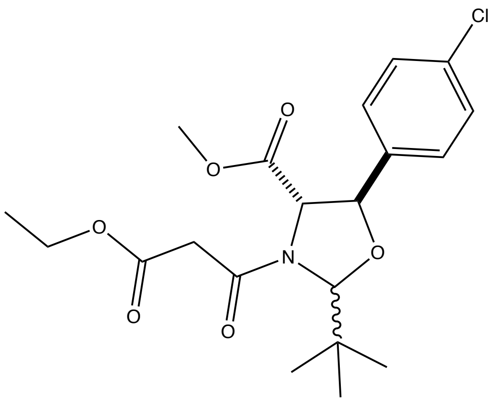

Prepared according to the General Method : oxazolidine (6.0 g, 20 mmol), ethyl malonyl chloride (1.65 g, 11 mmol), pyridine (1 mL), DCM (50 mL) Yield (4.66 g, 76%); Pale yellow oil; R_f (30% EtOAc in Pet. Ether 40:60) 0.50 $_{(\mathbf{8d})}$ and 0.38 $_{(\mathbf{9d})}$; ν_{max}/cm^{-1} $_{(\mathbf{8d})}$ 2959 (C-H), 1744 (C=O), 1671 (C=O); 60:40 diastereomeric ratio between **(±) 8d** and **(±) 9d**; $\delta_{H\ (\mathbf{8d})}$ (400 MHz, $CDCl_3$) 7.34 (s, 4H, Ar-H), 5.75 (s, 1H, H2), 5.68 (d, 1H, *J*=3.7 Hz, H4), 4.62 (d, 1H, *J*=4.0 Hz, H5), 4.07 (m, 2H, OCH_2CH_3), 3.85 (s, 3H, OCH_3), 3.50 (d, 1H, *J*=15.2 Hz, H7), 3.42 (br d, 1H, *J*=15.6 Hz, H7), 1.17 (br t, 3H, *J*=6.5 Hz, OCH_2CH_3), 0.99 (s, 9H, $C(CH_3)_3$); $^{13}C\{^1H\}$ δ_C $_{(\mathbf{8d})}$ (101 MHz, $CDCl_3$) 169.7 (C=O), 166.8 (C=O), 166.7 (C=O), 138.8 (Ar-C), 134.1 (Ar-C), 128.8 (Ar-C), 127.1 (Ar-C), 97.7 (C2), 80.9 (C4), 66.5 (C5), 61.5 (OCH_2CH_3), 53.0 (OCH_3), 42.5 (C7), 38.1 ($C(CH_3)_3$), 25.7 ($(CH_3)_3$), 13.9 (OCH_2CH_3); $\delta_{H\ (\mathbf{9d})\ (203K,\ Major\ conformer)}$ (500 MHz, CD_2Cl_2) 7.34 (m, 2H, Ar-H), 7.28 (d, 2H, *J*=8.5 Hz, Ar-H), 5.29 (s, 1H, H2), 5.07 (d, 1H, *J*=9.2 Hz, H4), 4.10 (m, 2H, OCH_2CH_3), 3.91 (d, 1H, *J*=9.2 Hz, H5), 3.68 (s, 3H, OCH_3), 3.50 (d, 1H, *J*=15.8 Hz, H7), 3.44 (d, 1H, *J*=15.6 Hz, H7), 1.21 (br m, 3H, OCH_2CH_3), 0.97 (s, 9H, $C(CH_3)_3$); $^{13}C\{^1H\}$ $\delta_{C\ (\mathbf{9d})\ (203K,\ Major\ conformer)}$ (126 MHz, CD_2Cl_2) 167.5 (C=O), 166.8 (C=O), 163.9 (C=O), 134.0 (Ar-C), 133.9 (Ar-C), 128.7 (Ar-C), 127.1 (Ar-C), 96.1 (C2),81.5 (C4), 64.8 (C5), 61.8 (OCH_2CH_3), 53.2 (OCH_3), 41.6 (C7), 39.0 ($C(CH_3)_3$), 25.1 ($(CH_3)_3$), 13.7 (OCH_2CH_3); LRMS $_{(\mathbf{8d})}$ (ESI^+) *m/z*: 412.1 ($[M^{35} + H]^+$ 79%), 414.2 ($[M^{37} + H]^+$ 67%); LRMS $_{(\mathbf{9d})}$ (ESI^+) *m/z*: 434.2 ($[M^{35} + Na]^+$ 98%), 436.2 ($[M^{37} + Na]^+$ 69%); HRMS (ESI^+) *m/z*: $[M^{35} + H]^+$ calcd. for $C_{20}H_{27}NO_6Cl$, 412.1521, found 412.1521; $[M^{37} + H]^+$ calcd. for $C_{20}H_{27}NO_6Cl$, 414.1492, found 414.1492.

Methyl (4*S,5*R**)-2-(*tert*-butyl)-5-(4-bromophenyl)-3-(3-ethoxy-3-oxopropanoyl)oxazolidine-4-carboxylate 8e and 9e**

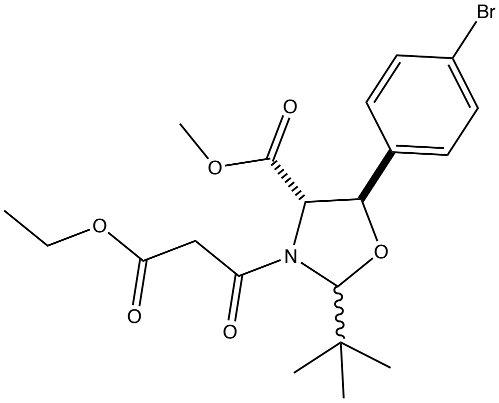

Prepared according to the General Method : oxazolidine (6.8 g, 20 mmol), ethyl malonyl chloride (1.65 g, 11 mmol), pyridine (1 mL), DCM (50 mL) Yield (8.53 g, 88%); Pale yellow oil; R_f (30% EtOAc in Pet. Ether 40:60) 0.50 $_{(\mathbf{8e})}$ and 0.40 $_{(\mathbf{9e})}$; ν_{max}/cm^{-1} $_{(\mathbf{8e})}$ 2958 (C-H), 1743 (C=O), 1675 (C=O); 60:40 diastereomeric ratio between **(±) 8e** and **(±) 9e**; $\delta_{H\ (\mathbf{8e})}$ (400 MHz, $CDCl_3$) 7.50 (d, 2H, *J*=8.4 Hz, Ar-H),

7.27 (d, 2H, *J*=8.4 Hz, Ar-H), 5.76 (s, 1H, H2), 5.66 (d, 1H, *J*=4.1 Hz, H4), 4.58 (d, 1H, *J*=4.2 Hz, H5), 4.07 (m, 2H, OCH_2CH_3), 3.86 (s, 3H, OCH_3), 3.49 (d, 1H, *J*=15.3 Hz, H7), 3.42 (d, 1H, *J*=15.4 Hz, H7), 1.17 (br t, 3H, *J*=7.2 Hz, OCH_2CH_3), 0.98 (s, 9H, $C(CH_3)_3$); $^{13}C\{^1H\}$ $\delta_{C\ (\mathbf{8e})}$ (101 MHz, $CDCl_3$) 169.9 (C=O), 166.9 (C=O), 166.9 (C=O), 139.4 (Ar-C), 132.0 (Ar-C), 127.6 (Ar-C), 122.6 (Ar-C), 98.0 (C2), 81.2 (C4), 66.7 (C5), 61.8 (OCH_2CH_3), 53.3 (OCH_3), 42.8 (C7), 38.4 ($C(CH_3)_3$), 25.9 ($(CH_3)_3$), 14.1 (OCH_2CH_3); $\delta_{H\ (\mathbf{9e})\ (193K,\ Major\ conformer)}$ (500 MHz, CD_2Cl_2) 7.48 (d, 2H, *J*=8.3 Hz, Ar-H), 7.21 (d, 2H, *J*=8.1 Hz, Ar-H), 5.27 (s, 1H, H2), 5.04 (d, 1H, *J*=9.1 Hz, H4), 4.07 (m, 2H, OCH_2CH_3), 3.89 (d, 1H, *J*=9.2 Hz, H5), 3.66 (s, 3H, OCH_3), 3.48 (d, 1H, *J*=16.5 Hz, H7), 3.43 (d, 1H, *J*=15.1 Hz, H7), 1.19 (m, 3H, OCH_2CH_3), 0.93 (s, 9H, $C(CH_3)_3$); $^{13}C\{^1H\}$ $\delta_{C\ (\mathbf{9e})\ (193K,\ Major\ conformer)}$ (126 MHz, CD_2Cl_2) 167.4 (C=O), 166.8 (C=O), 163.9 (C=O), 134.4 (Ar-C), 131.5 (Ar-C), 127.3 (Ar-C), 122.2 (Ar-C), 96.0 (C2), 81.4 (C4), 64.6 (C5), 61.7 (OCH_2CH_3), 53.2 (OCH_3), 41.4 (C7), 38.9 ($C(CH_3)_3$), 25.0 ($(CH_3)_3$), 13.6 (OCH_2CH_3); LRMS $_{(\mathbf{8e})}$ (ESI^+) *m/z*: 478.0 ($[M^{79} + Na]^+$ 96%), 480.0 ($[M^{81} + Na]^+$ 89%); LRMS $_{(\mathbf{9e})}$ (ESI^+) *m/z*: 478.0 ($[M^{79} + Na]^+$ 94%), 480.0 ($[M^{81} + Na]^+$ 96%); HRMS (ESI^+) *m/z*: $[M^{79} + H]^+$ calcd. for $C_{20}H_{27}NO_6Br$, 456.1016, found 456.1016; $[M^{81} + H]^+$ calcd. for $C_{20}H_{27}NO_6Br$, 458.0996, found 458.0993.

Ethyl (2*R,4*S**,5*R**)-2-(*tert*-butyl)-3-(3-ethoxy-3-oxopropanoyl)-5-phenyloxazolidine-4-carboxylate 8f**

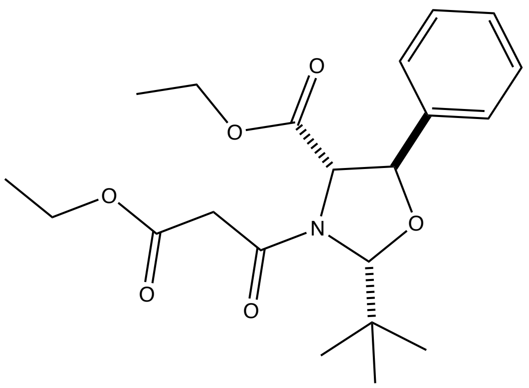

Prepared according to the General Method : oxazolidine (0.55 g, 2.0 mmol), ethyl malonyl chloride (1.65 g, 11 mmol), pyridine (1 mL), DCM (20 mL) Yield 34% (771 mg); Pale yellow oil; R_f (20% EtOAc in Pet. Ether 40:60) 0.43; ν_{max}/cm^{-1} 2959 (C-H), 1741 (C=O), 1675 (C=O); δ_H (400 MHz, $CDCl_3$) 7.38-7.26 (m, 5H, Ar-H),5.78 (s, 1H, H2), 5.67 (d, 1H, *J*=4.4 Hz, H4), 4.55 (d, 1H, *J*=4.3 Hz, H5), 4.30 (m, 2H, OCH_2CH_3), 4.04 (m, 2H, OCH_2CH_3), 3.47 (d, 1H, *J*=15.5 Hz, H7), 3.42 (d, 1H, *J*=15.5 Hz, H7), 1.34 (t, 3H, *J*=7.1 Hz, OCH_2CH_3), 1.15 (t, 3H, *J*=7.2 Hz, OCH_2CH_3), 0.99 (s, 9H, $C(CH_3)_3$); $^{13}C\{^1H\}$ δ_C (101 MHz, $CDCl_3$) 169.6 (C=O), 167.0 (C=O), 166.9 (C=O), 140.4 (Ar-C), 128.9 (Ar-C), 128.6 (Ar-C), 125.8 (Ar-C), 98.0 (C2), 81.9 (C4), 67.0 (C5), 62.5 (OCH_2CH_3), 61.7 (OCH_2CH_3), 42.7 (C7), 38.4 ($C(CH_3)_3$), 25.9 ($(CH_3)_3$), 14.2 (OCH_2CH_3), 14.1 (OCH_2CH_3); LRMS (ESI^+) *m/z*: 414.2 ($[M + Na]^+$ 96%); HRMS (ESI^+) *m/z*: $[M + H]^+$ calcd. for $C_{21}H_{30}NO_6$, 392.2068, found 392.2064.

General procedure for the synthesis of tetramates 10a-e:[10-11]

To a solution of malonamide **(±) 9a-e** (0.2-1.1 mmol, 1.0 eq.) in anhydrous THF (10 – 50 mL) was added potassium *tert*-butoxide (1.1 eq.). The mixture was heated at reflux for 3 h or refluxed overnight. Then the reaction mixture was cooled, separated between Et_2O and water and the aqueous layer was acidified to pH 1 using 3 M HCl (aq.) and extracted with EtOAc. The organic layer was washed with brine, dried over Na_2SO_4, filtered and concentrated under reduced pressure to obtain ethyl ester tetramates **10a-e** and was used without further purification.

Ethyl (1*R,3*S**,7a*S**)-3-(*tert*-butyl)-7-hydroxy-5-oxo-1-phenyl-5,7a-dihydro-1*H*,3*H*-pyrrolo[1,2-*c*]oxazole-6-carboxylate 10a**

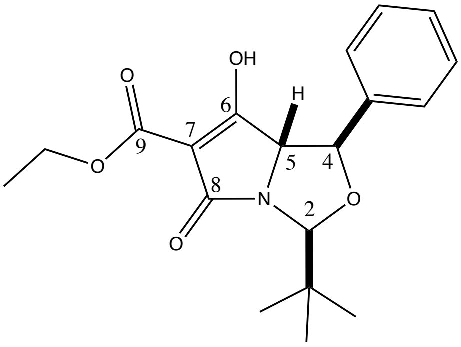

Prepared according to the General Method : malonamide (90 mg, 0.24 mmol), potassium *tert*-butoxide (25 mg, 0.22 mmol), THF (10 mL), Yield (88 mg, 80%); Yellow solid; m.p. 85-89°C; ν_{max}/cm^{-1} 2961 (C-H), 1711 (C=O), 1656 (C=O), 1616 (C=C); δ_H (400 MHz, $CDCl_3$) 7.49-7.32 (m, 5H, Ar-H), 4.99 (s, 1H, H2), 4.53 (d, 1H, *J*=8.4 Hz, H4), 4.36 (q, 2H, *J*=7.1 Hz, OCH_2CH_3), 4.17 (d, 1H, *J*=8.5 Hz, H5), 1.36 (t, 3H, *J*=7.1 Hz, OCH_2CH_3), 1.05 (s, 9H, $C(CH_3)_3$); $^{13}C\{^1H\}$ δ_C (101 MHz, $CDCl_3$) 185.3 (C6), 171.4 (C8), 167.1 (C9), 135.8 (Ar-C), 128.8 (Ar-C), 128.7 (Ar-C), 126.2 (Ar-C), 99.8 (C7), 94.9 (C2), 80.9 (C4), 67.9 (C5), 61.7 (OCH_2CH_3), 36.0 ($C(CH_3)_3$), 24.9 ($(CH_3)_3$), 14.1 (OCH_2CH_3); LRMS (ESI$^+$) *m/z*: 368.2 ([M + Na]$^+$ 95%); LRMS (ESI$^-$) *m/z*: 344.1 ([M – H]$^-$ 95%); HRMS (ESI$^+$) *m/z*: [M + Na]$^+$ calcd. for $C_{19}H_{23}NO_5Na$, 368.1468, found 368.1469.

Ethyl (1*R,3*S**,7a*S**)-3-(*tert*-butyl)-7-hydroxy-5-oxo-1-(4-methylphenyl)-5,7a-dihydro-1*H*,3*H*-pyrrolo[1,2-*c*]oxazole-6-carboxylate 10b**

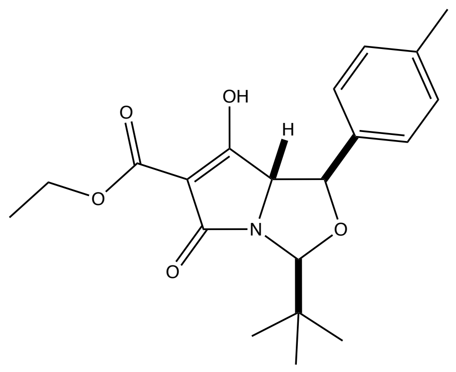

Prepared according to the General Method : malonamide (497 mg, 1.2 mmol), potassium *tert*-butoxide (146 mg, 1.3 mmol), THF (50 mL), Yield (401 mg, 79%); Orange solid; m.p. 91-95°C; ν_{max}/cm^{-1} 2969 (C-H), 1716 (C=O), 1658 (C=O), 1617 (C=C); δ_H (400 MHz, $CDCl_3$) 7.38 (d, 2H, *J*=8.1 Hz, Ar-H), 7.21 (d, 2H, *J*=7.9 Hz, Ar-H), 5.00 (s, 1H, H2), 4.51 (d, 1H, *J*=8.5 Hz, H4), 4.40 (q, 2H, *J*=7.1 Hz, OCH_2CH_3), 4.17 (d, 1H, *J*=8.5 Hz, H5), 2.37 (s, 3H, CH_3), 1.40 (t, 3H, *J*=7.1 Hz, OCH_2CH_3), 1.06 (s, 9H, $C(CH_3)_3$); $^{13}C\{^1H\}$ δ_C (101 MHz, $CDCl_3$) 185.7 (C6), 171.6 (C8), 167.5 (C9), 138.9 (Ar-C), 133.0 (Ar-C),129.5 (Ar-C), 126.4 (Ar-C), 99.9 (C7), 95.2 (C2), 81.2 (C4), 68.1 (C5), 61.8 (OCH_2CH_3), 36.2 ($C(CH_3)_3$), 25.1 ($(CH_3)_3$), 21.4 (CH_3), 14.3 (OCH_2CH_3); LRMS (ESI$^+$) *m/z*: 382.2 ([M + Na]$^+$ 96%); LRMS (ESI$^-$) *m/z*: 358.1 ([M – H]$^-$ 96%); HRMS (ESI$^-$) *m/z*: [M – H]$^-$ calcd. for $C_{20}H_{24}NO_5$, 358.1660, found 358.1657.

Ethyl (1*R,3*S**,7a*S**)-3-(*tert*-butyl)-1-(4-fluorophenyl)-7-hydroxy-5-oxo-5,7a-dihydro-1*H*,3*H*-pyrrolo[1,2-*c*]oxazole-6-carboxylate 10c**

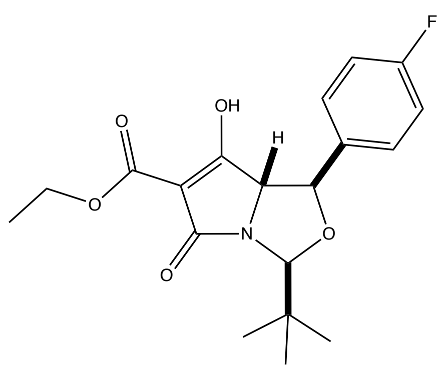

Prepared according to the General Method : malonamide (263 mg, 0.63 mmol), potassium *tert*-butoxide (78 mg, 0.70 mmol), THF (25 mL), Yield (228 mg, 60%); Red solid; m.p. 97-100°C; ν_{max}/cm^{-1} 2969 (C-H), 1715 (C=O), 1658 (C=O), 1617 (C=C); δ_H (400 MHz, $CDCl_3$) 7.48 (dd, 2H, *J*=8.7 Hz, 5.3 Hz, Ar-H),7.10 (app. t, 2H, *J*=8.6 Hz, Ar-H), 5.01 (s, 1H, H2), 4.54 (d, 1H, *J*=8.4 Hz, H4), 4.40 (q, 2H, *J*=7.1 Hz, OCH_2CH_3), 4.15 (d, 1H, *J*=8.4 Hz, H5), 1.40 (t, 3H, *J*=7.1 Hz, OCH_2CH_3), 1.06 (s, 9H, $C(CH_3)_3$); $^{13}C\{^1H\}$ δ_C (101 MHz, $CDCl_3$) 185.4 (C6), 170.0 (C8), 167.4 (C9), 163.1 (d, *J*=247.8 Hz, Ar-C), 131.8 (d, *J*=3.2 Hz, Ar-C), 128.2 (d, *J*=8.7 Hz, Ar-C),115.9 (d, *J*=22.1 Hz, Ar-C), 95.3 (C2), 80.5 (C4), 68.0 (C5), 62.0 (OCH_2CH_3), 36.2 ($C(CH_3)_3$), 25.0 ($(CH_3)_3$), 14.3 (OCH_2CH_3); δ_F (377 MHz, $CDCl_3$) -112.73; LRMS (ESI$^+$) *m/z*: 386.2 ([M + Na]$^+$ 96%); LRMS (ESI$^-$) *m/z*: 362.1 ([M – H]$^-$ 98%); HRMS (ESI$^-$) *m/z*: [M – H]$^-$ calcd. for $C_{19}H_{21}NO_5F$, 362.1409, found 362.1408.

Ethyl (1*R,3*S**,7a*S**)-3-(*tert*-butyl)-1-(4-chlorophenyl)-7-hydroxy-5-oxo-5,7a-dihydro-1*H*,3*H*-pyrrolo[1,2-*c*]oxazole-6-carboxylate 10d**

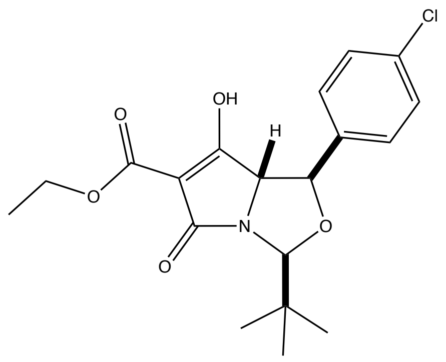

Prepared according to the General Method : malonamide (260 mg, 0.6 mmol), potassium *tert*-butoxide (74 mg, 0.66 mmol), THF (25 mL), Yield (236 mg, 74%); Red foamy solid; m.p. 99-105°C; ν_{max}/cm^{-1} 2969 (C-H), 1719 (C=O), 1662 (C=O), 1619 (C=C); δ_H (400 MHz, $CDCl_3$) 7.44 (d, 2H, *J*=8.5 Hz, Ar-H), 7.38 (d, 2H, *J*=8.6 Hz, Ar-H), 5.01 (s, 1H, H2), 4.54 (d, 1H, *J*=8.4 Hz, H4), 4.40 (q, 2H, *J*=7.1 Hz, OCH_2CH_3), 4.14 (d, 1H, *J*=8.4 Hz, H5), 1.40 (t, 3H, *J*=7.1 Hz, OCH_2CH_3), 1.05 (s, 9H, $C(CH_3)_3$); $^{13}C\{^1H\}$ δ_C (101 MHz, $CDCl_3$) 185.3 (C6), 172.4 (C8), 167.4 (C9), 134.8 (Ar-C), 134.5 (Ar-C), 129.1 (Ar-C), 127.7 (Ar-C), 95.3 (C2), 80.4 (C4), 67.9 (C5), 62.0 (OCH_2CH_3), 36.2 ($C(CH_3)_3$), 25.0 ($(CH_3)_3$), 14.3 (OCH_2CH_3); LRMS (ESI$^+$) *m/z*: 402.2 ([M^{35} + Na]$^+$ 91%), 404.2 ([M^{37} + Na]$^+$ 46%); LRMS (ESI$^-$) *m/z*: 378.0 ([M^{35} – H]$^-$ 97%), 380.0 [M^{37} – H]$^-$ 34%); HRMS (ESI$^-$) *m/z*: [M^{35} – H]$^-$ calcd. for $C_{19}H_{21}NO_5Cl$, 378.1114, found 378.1113; [M^{37} – H]$^-$ calcd. for $C_{19}H_{21}NO_5Cl$, 380.1085, found 380.1084.

Ethyl (1*R,3*S**,7a*S**)-3-(*tert*-butyl)-1-(4-bromophenyl)-7-hydroxy-5-oxo-5,7a-dihydro-1*H*,3*H*-pyrrolo[1,2-*c*]oxazole-6-carboxylate 10e**

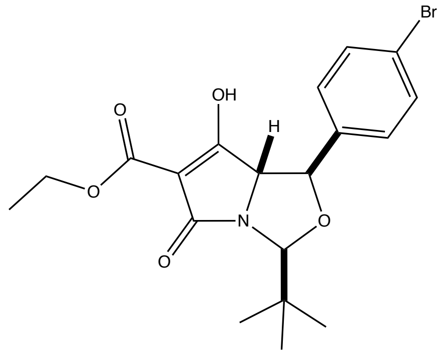

Prepared according to the General Method : malonamide (925 mg, 2.0 mmol), potassium *tert*-butoxide (246 mg, 2.2 mmol), THF (50 mL), Yield (808 mg, 78%); Red foamy solid; m.p. 95-97°C; ν_{max}/cm^{-1} 2968 (C-H), 1716 (C=O), 1618 (C=C); δ_H (400 MHz, $CDCl_3$) 7.53 (d, 2H, *J*=8.5 Hz, Ar-H), 7.38 (d, 2H, *J*=8.4 Hz, Ar-H), 5.00 (s, 1H, H2), 4.52 (d, 1H, *J*=8.4 Hz, H4), 4.39 (q, 2H, *J*=7.1 Hz, OCH_2CH_3), 4.13 (d, 1H, *J*=8.4 Hz, H5), 1.39 (t, 3H, *J*=7.1 Hz, OCH_2CH_3), 1.04 (s, 9H, $C(CH_3)_3$); $^{13}C\{^1H\}$ δ_C (101 MHz, $CDCl_3$) 185.2 (C6), 171.1 (C8), 167.4 (C9), 135.1 (Ar-C), 132.0 (Ar-C), 128.0 (Ar-C), 123.0 (Ar-C), 100.3 (C7), 95.2 (C2), 80.4 (C4), 67.7 (C5), 62.0 (OCH_2CH_3), 36.2 ($C(CH_3)_3$), 25.0 ($(CH_3)_3$), 14.3 (OCH_2CH_3); LRMS (ESI$^-$) *m/z*: 422.0 ([M^{79} – H]$^-$ 94%), 424.0 [M^{81} – H]$^-$ 97%); HRMS (ESI$^-$) *m/z*: [M^{79} – H]$^-$ calcd. for $C_{19}H_{21}NO_5Br$, 422.0609, found 422.0613; [M^{81} – H]$^-$ calcd. for $C_{19}H_{21}NO_5Br$, 424.0588, found 424.0591.

General procedure for the synthesis of C7-carboxamidotetramic acids:[12,13]

To a solution of ethyl ester tetramic acids **(±) 10a-e** (0.1 - 10.0 mmol, 1.0 eq.) in anhydrous toluene (5 – 50 mL) was added an aliphatic or aromatic amine (1.5 eq.). The mixture was heated at reflux overnight. Then the reaction mixture was evaporated *in-vacuo* and purified by flash column chromatography to yield tetramates that were chelated with metals. The tetramates were then redissolved in DCM, washed with 10% citric acid solution (aq.), dried over Na_2SO_4, filtered and concentrated under reduced pressure to yield the desired C7-carboxamides.

(1*R,3*S**,7a*S**)-*N*-(Adamantan-1-yl)-3-(*tert*-butyl)-7-hydroxy-5-oxo-1-phenyl-5,7a-dihydro-1*H*,3*H*-pyrrolo[1,2-*c*]oxazole-6-carboxamide 12a**

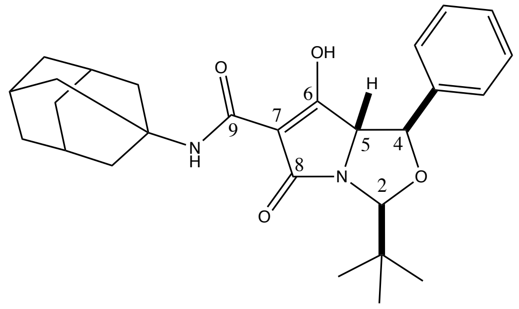

Prepared according to the General Method : tetramate (3.68 g, 0.01 mol), amine (2.27 g, 0.015 mmol), toluene (50 mL), Yield (3.32 g, 55%); Brown foamy solid; m.p. 130-135°C; R_f (30% EtOAc in Pet. Ether 40:60) 0.33; ν_{max}/cm^{-1} 2909 (C-H), 2852 (C-H), 1692 (C=O), 1645 (C=O); AB:CD=80:20; δ_H (400 MHz, $CDCl_3$) 9.00 (br s, 1H, NH/OH), 7.54-7.35 (m, 5H, Ar-H), 4.99 (s, 1H, H2, CD), 4.97 (s, 1H, H2, AB), 4.61 (d, 1H, *J*=8.9 Hz, H4, CD), 4.58 (d, 1H, *J*=8.6 Hz, H4, AB), 4.12 (d, 1H, *J*=8.7 Hz, H5, AB), 3.90 (d, 1H, *J*=8.8 Hz, H5, CD), 2.12 (br m, 3H, Adamantyl-CH), 2.08 (br m, 6H, Adamantyl-CH$_2$), 1.71 (br m, 6H, Adamantyl-CH$_2$), 1.07 (s, 9H, $C(CH_3)_3$, AB), 1.06 (s, 9H, $C(CH_3)_3$, CD); $^{13}C\{^1H\}$ δ_C (101 MHz, $CDCl_3$) 190.5 (C6, CD), 186.9 (C6, AB), 181.3 (C8, CD), 175.1 (C8, AB), 167.1 (C9, CD), 166.3 (C9, AB), 136.4 (Ar-C), 128.8 (Ar-C, AB), 128.8 (Ar-C, AB), 128.7 (Ar-C, CD), 126.5 (Ar-C, CD), 126.4 (Ar-C, AB), 97.0 (C7), 94.9 (C2, CD), 94.9 (C2, AB), 80.7 (C4, AB), 79.5 (C4, CD), 72.5 (C5, CD), 69.4 (C5, AB), 54.6 (Adamantyl-C, CD), 53.1 (Adamantyl-C, AB), 41.8 (Adamantyl-C),36.3 (Adamantyl-C, AB), 36.2 ($C(CH_3)_3$), 36.0 (Adamantyl-C, CD), 29.5 (Adamantyl-C), 25.1 ($(CH_3)_3$); LRMS (ESI$^-$) *m/z*: 449.2 ([M – H]$^-$ 100%); HRMS (ESI$^-$) *m/z*: [M – H]$^-$ calcd. for $C_{27}H_{33}N_2O_4$, 449.2446, found 449.2443.

(1*R,3*S**,7a*S**)-3-(*tert*-Butyl)-7-hydroxy-5-oxo-*N*,1-diphenyl-5,7a-dihydro-1*H*,3*H*-pyrrolo[1,2-*c*]oxazole-6-carboxamide 12b**

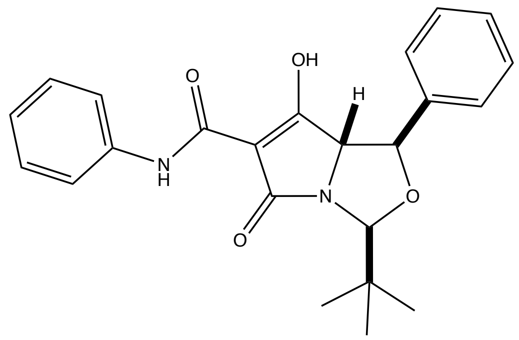

Prepared according to the General Method : tetramate (103 mg, 0.28 mmol), amine (39 mg, 0.42 mmol), toluene (10 mL) mmol scale, Yield (109 mg, 42%); Brown foamy solid; m.p. 75-79°C; R_f (50% EtOAc in Pet. Ether 40:60) 0.30; ν_{max}/cm^{-1} 3281 (O-H), 2960 (C-H), 1695 (C=O), 1650 (C=O), 1599 (C=C); AB:CD=99:1; δ_H (400 MHz, $CDCl_3$) 9.41 (br s, 1H, NH/OH), 9.16 (br s, 1H, NH/OH), 7.61-7.07 (m, 10H, Ar-H), 5.02 (s, 1H, H2), 4.63 (d, 1H, *J*=8.5 Hz, H4), 4.27 (d, 1H, *J*=8.5 Hz, H5), 1.11 (s, 9H, $C(CH_3)_3$); $^{13}C\{^1H\}$ δ_C (101 MHz, $CDCl_3$) 184.0 (C6), 174.4 (C8), 164.1 (C9), 136.6 (Ar-C), 135.8 (Ar-C), 129.3 (Ar-C), 129.1 (Ar-C), 128.9 (Ar-C), 126.4 (Ar-C), 125.2 (Ar-C), 120.5 (Ar-C), 100.0 (C7), 94.9 (C2), 81.1 (C4), 68.7 (C5), 36.2 ($C(CH_3)_3$), 25.0 ($(CH_3)_3$); LRMS (ESI$^-$) *m/z*: 391.1 ([M – H]$^-$ 97%); HRMS (ESI$^-$) *m/z*: [M – H]$^-$ calcd. for $C_{23}H_{23}N_2O_4$, 391.1663, found 391.1662.

(1*R,3*S**,7a*S**)-3-(*tert*-Butyl)-*N*-(4-bromophenyl)-7-hydroxy-5-oxo-1-phenyl-5,7a-dihydro-1*H*,3*H*-pyrrolo[1,2-*c*]oxazole-6-carboxamide 12c**

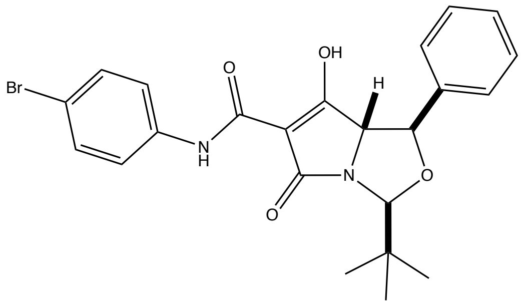

Prepared according to the General Method : tetramate (92 mg, 0.25 mmol), amine (65 mg, 0.38 mmol), toluene (10 mL), Yield (122 mg, 42%); Orange foamy solid; m.p. 91-94°C; R_f (50% EtOAc in Pet. Ether 40:60) 0.13; ν_{max}/cm^{-1} 3271 (O-H), 2962 (C-H), 1692 (C=O), 1633 (C=O), 1593 (C=C); AB:CD=99:1; δ_H (400 MHz, $CDCl_3$) 10.27 (br s, 1H, NH/OH), 9.44 (br s, 1H, NH/OH), 7.53-7.38 (m, 9H, Ar-H), 5.01 (s, 1H, H2), 4.61 (d, 1H, *J*=8.5 Hz, H4), 4.27 (d, 1H, *J*=8.5 Hz, H5), 1.11 (s, 9H, $C(CH_3)_3$); $^{13}C\{^1H\}$ δ_C (101 MHz, $CDCl_3$) 183.7 (C6), 174.2 (C8), 164.0 (C9), 135.8 (Ar-C), 135.7 (Ar-C), 132.3 (Ar-C), 129.1 (Ar-C), 128.9 (Ar-C), 126.3 (Ar-C), 121.8 (Ar-C), 117.8 (Ar-C), 100.1 (C7), 94.9 (C2), 81.1 (C4), 68.7 (C5), 36.2 ($C(CH_3)_3$), 25.0 ($(CH_3)_3$); LRMS (ESI$^-$) *m/z*: 469.0 ([M^{79} – H]$^-$ 11%), 471.0 ([M^{81} – H]$^-$ 11%); HRMS (ESI$^-$) *m/z*: [M^{79} – H]$^-$ calcd. for $C_{23}H_{22}N_2O_4Br$, 469.0768, found 469.0770; [M^{81} – H]$^-$ calcd. for $C_{23}H_{22}N_2O_4Br$, 471.0748, found 471.0749.

(1*R,3*S**,7a*S**)-3-(*tert*-Butyl)-7-hydroxy-*N*-(4-nitrophenyl)-5-oxo-1-phenyl-5,7a-dihydro-1*H*,3*H*-pyrrolo[1,2-*c*]oxazole-6-carboxamide 12d**

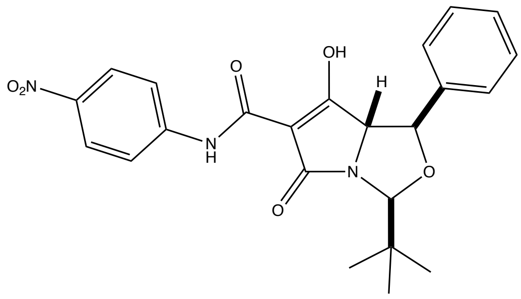

Prepared according to the General Method : tetramate (368 mg, 0.1 mmol), amine (21 mg, 0.15 mmol), toluene (25 mL), Yield (41 mg, 15%); Dark brown foam; m.p. 175-179°C; R_f (50% EtOAc in Pet. Ether 40:60) 0.13; v_{max}/cm^{-1} 2963 (C-H), 1694 (C=O), 1632 (C=O), 1595 (C=C), 1511 (N-O); AB:CD=99:1; δ_H (400 MHz, $CDCl_3$) 9.82 (br s, 1H, NH/OH), 8.23 (d, 2H, *J*=8.9 Hz, Ar-H), 7.78 (d, 2H, *J*=8.8 Hz, Ar-H), 7.54-7.38 (m, 5H, Ar-H), 5.02 (s, 1H, H2), 4.64 (d, 1H, *J*=8.5 Hz, H4), 4.31 (d, 1H, *J*=8.5 Hz, H5), 1.11 (s, 9H, $C(CH_3)_3$); $^{13}C\{^1H\}$ δ_C (101 MHz, $CDCl_3$) 184.0 (C6), 174.0 (C8), 164.3 (C9), 144.1 (Ar-C), 142.6 (Ar-C), 135.5 (Ar-C), 129.2 (Ar-C), 129.0 (Ar-C), 126.4 (Ar-C), 125.3 (Ar-C), 119.8 (Ar-C), 100.3 (C7), 94.9 (C2), 81.1 (C4), 68.7 (C5), 36.2 ($C(CH_3)_3$), 25.0 ($(CH_3)_3$); LRMS (ESI$^-$) *m/z*: 436.0 ([M – H]$^-$ 97%); HRMS (ESI$^-$) *m/z*: [M – H]$^-$ calcd. for $C_{23}H_{22}N_3O_6$, 436.1514, found 436.1512.

(1*R,3*S**,7a*S**)-3-(*tert*-Butyl)-7-hydroxy-5-oxo-1-phenyl-*N*-(4-(trifluoromethyl)phenyl)-5,7a-dihydro-1*H*,3*H*-pyrrolo[1,2-*c*]oxazole-6-carboxamide 12e**

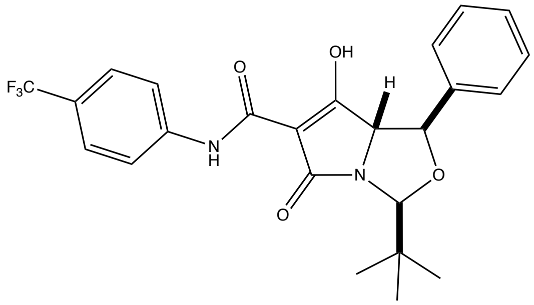

Prepared according to the General Method : tetramate (88 mg, 0.24 mmol), amine (58 mg, 0.36 mmol), toluene (10 mL), Yield (109 mg, 38%); Red oil; R_f (50% EtOAc in Pet. Ether 40:60) 0.15; v_{max}/cm^{-1} 3270 (O-H), 2963 (C-H), 1698 (C=O), 1655 (C=O), 1636 (C=O), 1605 (C=C); AB:CD=99:1; δ_H (400 MHz, $CDCl_3$) 9.61 (br s, 1H, NH/OH), 7.74 (d, 2H, *J*=8.4 Hz, Ar-H), 7.61 (d, 2H, *J*=8.6 Hz, Ar-H), 7.54-7.33 (m, 5H, Ar-H), 5.02 (s, 1H, H2), 4.63 (d, 1H, *J*=8.5 Hz, H4), 4.30 (d, 1H, *J*=8.6 Hz, H5), 1.11 (s, 9H, $C(CH_3)_3$); $^{13}C\{^1H\}$ δ_C (101 MHz, $CDCl_3$) 183.9 (C6), 174.2 (C8), 164.3 (C9), 139.8 (Ar-C), 135.6 (Ar-C), 129.2 (Ar-C), 129.0 (Ar-C), 126.6 (q, *J*=3.7 Hz, Ar-C), 126.4 (Ar-C), 120.0 (Ar-C), 100.2 (C7), 94.9 (C2), 81.1 (C4), 68.7 (C5), 36.2 ($C(CH_3)_3$), 25.0 ($(CH_3)_3$); δ_F (377 MHz, $CDCl_3$) -62.18; LRMS (ESI$^-$) *m/z*: 459.0 ([M – H]$^-$ 100%); HRMS (ESI$^-$) *m/z*: [M – H]$^-$ calcd. for $C_{24}H_{22}N_2O_4F_3$, 459.1537, found 459.1540.

(1*R,3*S**,7a*S**)-3-(*tert*-Butyl)-*N*-(4-(*tert*-butyl)phenyl)-7-hydroxy-5-oxo-1-phenyl-5,7a-dihydro-1*H*,3*H*-pyrrolo[1,2-*c*]oxazole-6-carboxamide 12f**

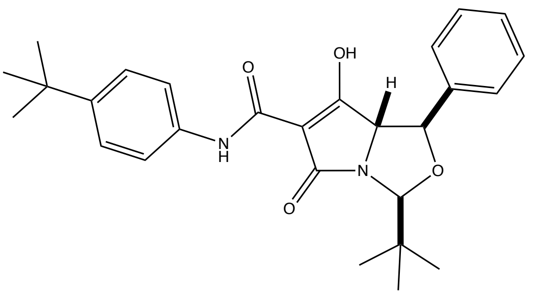

Prepared according to the General Method : tetramate (81 mg, 0.22 mmol), amine (49 mg, 0.33 mmol), toluene (10 mL), Yield (96 mg, 27%); Red/brown solid; m.p. 89-94°C; R_f (50% EtOAc in Pet. Ether 40:60) 0.75; ν_{max}/cm^{-1} 2962 (C-H), 1694 (C=O), 1646 (C=O), 1601 (C=C); AB:CD=99:1; δ_H (400 MHz, $CDCl_3$) 9.93 (br s, 1H, NH/OH), 9.50 (br s, 1H, NH/OH), 7.46-7.12 (m, 9H, Ar-H), 4.90 (s, 1H, H2), 4.44 (d, 1H, *J*=8.5 Hz, H4), 4.00 (br m, 1H, H5), 1.18 (s, 9H, Ar-C(CH_3)$_3$), 0.98 (s, 9H, C(CH_3)$_3$); ^{13}C{^{1}H} δ_C (101 MHz, $CDCl_3$) 185.1 (C6),175.4 (C8), 164.1 (C9), 147.8 (Ar-C), 136.1 (Ar-C),134.1 (Ar-C), 128.7 (Ar-C), 126.4 (Ar-C), 126.2 (Ar-C), 126.0 (Ar-C), 120.3 (Ar-C), 98.7 (C7), 94.8 (C2), 80.9 (C4), 69.1 (C5), 36.1 (C(CH_3)$_3$), 34.4 (Ar-C(CH_3)$_3$), 31.4 ((Ar-(CH_3)$_3$), 25.0 ((CH_3)$_3$); LRMS (ESI$^-$) *m/z*: 447.2 ([M – H]$^-$ 100%); HRMS (ESI$^-$) *m/z*: [M – H]$^-$ calcd. for $C_{27}H_{31}N_2O_4$, 447.2289, found 447.2289.

(1*R,3*S**,7a*S**)-3-(*tert*-Butyl)-*N*-(4-chloro-2-methylphenyl)-7-hydroxy-5-oxo-1-phenyl-5,7a-dihydro-1*H*,3*H*-pyrrolo[1,2-*c*]oxazole-6-carboxamide 12g**

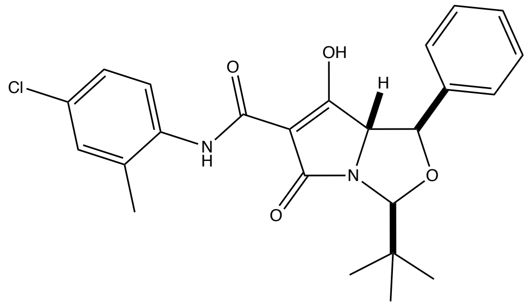

Prepared according to the General Method : tetramate (92 mg, 0.25 mmol), amine (54 mg, 0.38 mmol), toluene (10 mL), Yield (109 mg, 40%); Brown foamy solid; m.p. 101-105°C; R_f (50% EtOAc in Pet. Ether 40:60) 0.18; ν_{max}/cm^{-1} 3270 (O-H), 2961 (C-H), 1694 (C=O), 1634 (C=O), 1587 (C=C); AB:CD=99:1; δ_H (400 MHz, $CDCl_3$) 9.42 (br s, 1H, NH/OH), 9.18 (br s, 1H, NH/OH), 8.02 (d, 1H, *J*=8.4 Hz, Ar-H), 7.54-7.37 (m, 5H, Ar-H), 7.19-7.17 (m, 2H, Ar-H), 5.03 (s, 1H, H2), 4.63 (d, 1H, *J*=8.4 Hz, H4), 4.27 (d, 1H, *J*=8.5 Hz, H5),2.36 (s, 3H, CH_3), 1.11 (s, 9H, C(CH_3)$_3$); ^{13}C{^{1}H} δ_C (101 MHz, $CDCl_3$) 183.6 (C6), 174.4 (C8), 163.9 (C9), 135.8 (Ar-C), 133.5 (Ar-C), 130.5 (Ar-C), 130.3 (Ar-C), 130.2 (Ar-C),129.0 (Ar-C), 128.9 (Ar-C), 126.8 (Ar-C), 126.3 (Ar-C), 122.9 (Ar-C), 100.2 (C7), 94.7 (C2), 81.0 (C4), 68.7 (C5), 36.2 (C(CH_3)$_3$), 25.0 ((CH_3)$_3$), 17.8 (CH_3); LRMS (ESI$^-$) *m/z*: 439.1 ([M^{35} – H]$^-$ 36%),

441.1 ([M^{37} – H]$^-$ 15%); HRMS (ESI$^-$) *m/z*: [M^{35} – H]$^-$ calcd. for $C_{24}H_{24}N_2O_4Cl$, 439.1430, found 439.1427; [M^{37} – H]$^-$ calcd. for $C_{24}H_{24}N_2O_4Cl$, 441.1401, found 441.1393.

(1*R,3*S**,7a*S**)-3-(*tert*-Butyl)-*N*-(2-chloro-4-methylphenyl)-7-hydroxy-5-oxo-1-phenyl-5,7a-dihydro-1*H*,3*H*-pyrrolo[1,2-*c*]oxazole-6-carboxamide 12h**

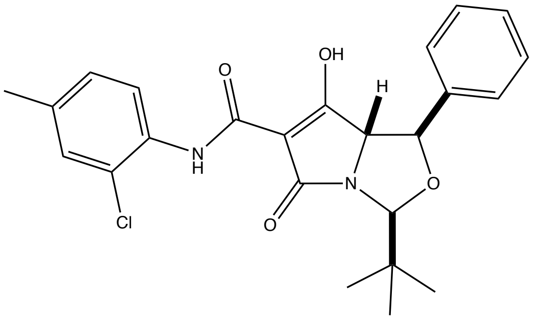

Prepared according to the General Method : tetramate (118 mg, 0.32 mmol), amine (71 mg, 0.50 mmol), toluene (10 mL), Yield (141 mg, 44%); Brown foam; m.p. 104-107°C; R_f (50% EtOAc in Pet. Ether 40:60) 0.25; ν_{max}/cm^{-1} 2959 (C-H), 1699 (C=O), 1649 (C=O), 1631 (C=O), 1609 (C=O), 1593 (C=C); AB:CD=99:1; δ_H (400 MHz, $CDCl_3$) 9.85 (br s, 1H, NH/OH), 8.20 (d, 1H, *J*=8.3 Hz, Ar-H), 7.54-7.24 (m, 6H, Ar-H), 7.08 (d, 1H, *J*=8.3 Hz, Ar-H), 5.06 (s, 1H, H2), 4.63 (d, 1H, *J*=8.4 Hz, H4), 4.27 (d, 1H, *J*=8.4 Hz, H5), 2.32 (s, 3H, CH_3), 1.11 (s, 9H, $C(CH_3)_3$); $^{13}C\{^1H\}$ δ_C (101 MHz, $CDCl_3$) 183.9 (C6), 174.0 (C8), 164.2 (C9), 135.9 (Ar-C), 135.8 (Ar-C), 131.2 (Ar-C), 129.9 (Ar-C), 129.0 (Ar-C), 128.9 (Ar-C), 128.3 (Ar-C), 126.4 (Ar-C), 124.0 (Ar-C), 122.1 (Ar-C), 100.4 (C7), 94.8 (C2), 81.1 (C4), 68.7 (C5), 36.3 ($C(CH_3)_3$), 25.1 ($(CH_3)_3$), 20.8 (CH_3); LRMS (ESI$^-$) *m/z*: 439.0 ([M^{35} – H]$^-$ 97%), 441.1 ([M^{37} – H]$^-$ 28%); HRMS (ESI$^-$) *m/z*: [M^{35} – H]$^-$ calcd. for $C_{24}H_{24}N_2O_4Cl$, 439.1430, found 439.1434; [M^{37} – H]$^-$ calcd. for $C_{24}H_{24}N_2O_4Cl$, 441.1401, found 441.1404.

(1*R,3*S**,7a*S**)-3-(*tert*-Butyl)-*N*-(2-chloro-4-(trifluoromethyl)phenyl)-7-hydroxy-5-oxo-1-phenyl-5,7a-dihydro-1*H*,3*H*-pyrrolo[1,2-*c*]oxazole-6-carboxamide 12i**

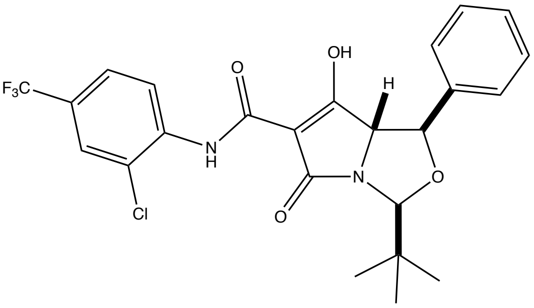

Prepared according to the General Method : tetramate (55 mg, 0.15 mmol), amine (43 mg, 0.22 mmol), toluene (5 mL), Yield (77 mg, 21%); Red foam; m.p. 93-97°C; R_f (50% EtOAc in Pet. Ether 40:60) 0.15; ν_{max}/cm^{-1} 2963 (C-H), 1701 (C=O), 1658 (C=O), 1633 (C=O), 1594 (C=C); AB:CD=99:1; δ_H (400 MHz, $CDCl_3$) 10.19 (br s, 1H, NH/OH), 8.59 (d, 1H, *J*=8.7 Hz, Ar-H), 7.70-7.34 (m, 7H, Ar-H), 5.07 (s, 1H, H2), 4.64 (d, 1H, *J*=8.5 Hz, H4), 4.31 (d, 1H, *J*=8.5 Hz, H5), 1.11 (s, 9H, $C(CH_3)_3$); $^{13}C\{^1H\}$ δ_C (101 MHz, $CDCl_3$) 184.0 (C6), 173.8 (C8), 164.5 (C9),

137.1 (Ar-C), 135.7 (Ar-C), 129.6 (Ar-C), 129.2 (Ar-C), 129.0 (Ar-C), 126.7 (q, *J*=3.7 Hz, Ar-C), 126.4 (Ar-C), 124.9 (q, *J*=3.7 Hz, Ar-C), 123.8 (Ar-C), 121.6 (Ar-C), 100.6 (C7), 94.9 (C2),81.2 (C4), 68.7 (C5), 36.3 ($C(CH_3)_3$), 25.1 ($(CH_3)_3$); δ_F (377 MHz, $CDCl_3$) -62.35; LRMS (ESI^-) *m/z*: 493.0 ($[M^{35} – H]^-$ 100%), 495.0 ($[M^{37} – H]^-$ 39%); HRMS (ESI^-) *m/z*: $[M^{35} – H]^-$ calcd. for $C_{24}H_{21}N_2O_4F_3Cl$, 493.1147, found 493.1152; $[M^{37} – H]^-$ calcd. for $C_{24}H_{21}N_2O_4F_3Cl$, 495.1118, found 495.1122.

(1*R,3*S**,7a*S**)-3-(*tert*-Butyl)-*N*-(4-chloro-2-(trifluoromethyl)phenyl)-7-hydroxy-5-oxo-1-phenyl-5,7a-dihydro-1*H*,3*H*-pyrrolo[1,2-*c*]oxazole-6-carboxamide 12j**

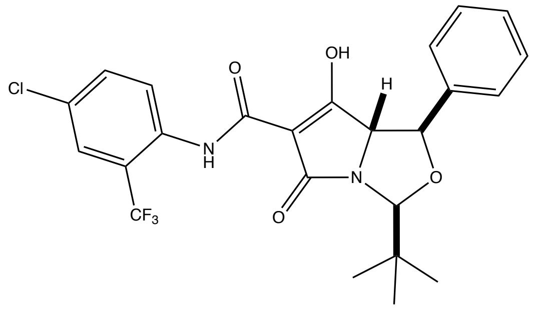

Prepared according to the General Method : tetramate (103 mg, 0.28 mmol), amine (82 mg, 0.42 mmol), toluene (10 mL), Yield (139 mg, 43%); Orange foam; m.p. 107-111°C; R_f (50% EtOAc in Pet. Ether 40:60) 0.15; ν_{max}/cm^{-1} 3270 (O-H), 2964 (C-H), 1701 (C=O), 1633 (C=O), 1590 (C=C); AB:CD=99:1; δ_H (400 MHz, $CDCl_3$) 9.83 (br s, 1H, NH/OH), 9.16 (br s, 1H, NH/OH), 8.20 (d, 1H, *J*=8.9 Hz, Ar-H), 7.65-7.37 (m, 7H, Ar-H), 5.06 (s, 1H, H2), 4.64 (d, 1H, *J*=8.4 Hz, H4), 4.28 (d, 1H, *J*=8.5 Hz, H5), 1.10 (s, 9H, $C(CH_3)_3$); $^{13}C\{^1H\}$ δ_C (101 MHz, $CDCl_3$) 183.8 (C6), 173.7 (C8), 164.5 (C9), 135.8 (Ar-C), 132.8 (Ar-C), 132.6 (Ar-C), 129.6-129.5 (m, Ar-C), 129.1 (Ar-C), 128.9 (Ar-C), 126.7-126.6 (m, Ar-C), 126.4 (Ar-C), 126.1 (Ar-C), 125.8 (Ar-C), 100.2 (C7), 94.8 (C2), 81.1 (C4), 68.6 (C5), 36.3 ($C(CH_3)_3$), 25.1 ($(CH_3)_3$); δ_F (377 MHz, $CDCl_3$) -61.31; LRMS (ESI^-) *m/z*: 493.0 ($[M^{35} – H]^-$ 97%), 495.0 ($[M^{37} – H]^-$ 34%); HRMS (ESI^-) *m/z*: $[M^{35} – H]^-$ calcd. for $C_{24}H_{21}N_2O_4F_3Cl$, 493.1147, found 493.1149; $[M^{37} – H]^-$ calcd. for $C_{24}H_{21}N_2O_4F_3Cl$, 495.1118, found 495.1119.

(1*R,3*S**,7a*S**)-3-(*tert*-Butyl)-*N*-(2,4-dimethylphenyl)-7-hydroxy-5-oxo-1-phenyl-5,7a-dihydro-1*H*,3*H*-pyrrolo[1,2-*c*]oxazole-6-carboxamide 12k**

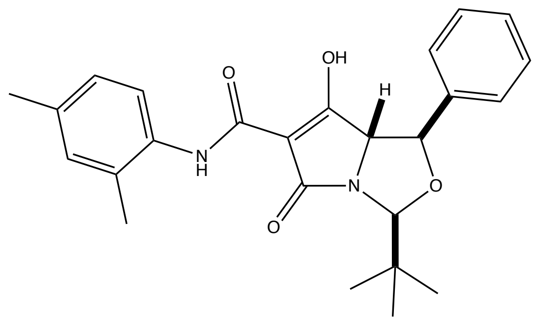

Prepared according to the General Method: tetramate (88 mg, 0.24 mmol), amine (44 mg, 0. 36 mmol), toluene (10 mL), Yield (102 mg, 41%); Yellow foam; m.p. 77-81°C; R_f (50% EtOAc in Pet. Ether 40:60) 0.45; ν_{max}/cm^{-1} 2968 (C-H), 1694 (C=O), 1648 (C=O),

1633 (C=O), 1603 (C=C); AB:CD=99:1; δ_H (400 MHz, $CDCl_3$) 9.29 (br s, 1H, NH/OH), 8.14 (br s, 1H, NH/OH), 7.85 (d, 1H, *J*=8.7 Hz, Ar-H), 7.56-7.53 (m, 2H, Ar-H), 7.46-7.37 (m, 3H, Ar-H), 7.04 (m, 2H, Ar-H), 5.05 (s, 1H, H2), 4.65 (d, 1H, *J*=8.5 Hz, H4), 4.27 (d, 1H, *J*=8.5 Hz, H5), 2.36 (s, 3H, CH_3), 2.32 (s, 3H, CH_3), 1.11 (s, 9H, $C(CH_3)_3$); $^{13}C\{^1H\}$ δ_C (101 MHz, $CDCl_3$) 184.2 (C6), 174.5 (C8),164.2 (C9), 136.0 (Ar-C), 135.3 (Ar-C),132.1 (Ar-C), 131.4 (Ar-C), 129.0 (Ar-C), 129.0 (Ar-C), 128.9 (Ar-C), 127.4 (Ar-C), 126.4 (Ar-C), 122.3 (Ar-C), 99.9 (C7), 94.8 (C2), 81.0 (C4), 68.8 (C5), 36.3 ($C(CH_3)_3$), 25.1 ($(CH_3)_3$), 21.1 (CH_3), 17.9 (CH_3); LRMS (ESI$^-$) *m/z*: 419.1 ($[M-H]^-$ 95%); HRMS (ESI$^+$) *m/z*: $[M+H]^+$ calcd. for $C_{25}H_{29}N_2O_4$, 421.2122, found 421.2125.

(1*R,3*S**,7a*S**)-3-(*tert*-Butyl)-*N*-(4-fluoro-2-methylphenyl)-7-hydroxy-5-oxo-1-phenyl-5,7a-dihydro-1*H*,3*H*-pyrrolo[1,2-*c*]oxazole-6-carboxamide 12l**

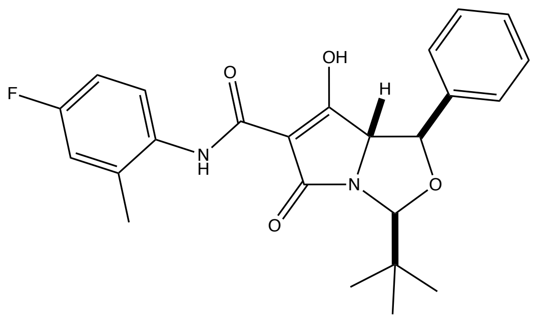

Prepared according to the General Method : tetramate (114 mg, 0.31 mmol), amine (58 mg, 0.46 mmol), toluene (10 mL), Yield (131 mg, 51%); Red/brown foam; m.p. 65-69°C; R_f (50% EtOAc in Pet. Ether 40:60) 0.45; v_{max}/cm^{-1} 3292 (O-H), 2961 (C-H), 1693 (C=O), 1632 (C=O), 1558 (C=C); AB:CD=99:1; δ_H (400 MHz, $CDCl_3$) 9.33 (br s, 1H, NH/OH), 9.17 (br s, 1H, NH/OH), 7.95 (dd, 1H, *J*=8.6 Hz, 5.3 Hz, Ar-H), 7.56-7.37 (m, 5H, Ar-H), 6.95-6.90 (m, 2H, Ar-H), 5.04 (s, 1H, H2), 4.64 (d, 1H, *J*=8.5 Hz, H4), 4.27 (d, 1H, *J*=8.5 Hz, H5), 2.37 (s, 3H, CH_3), 1.12 (s, 9H, $C(CH_3)_3$); $^{13}C\{^1H\}$ δ_C (101 MHz, $CDCl_3$) 183.8 (C6), 174.4 (C8), 164.0 (C9), 159.9 (d, *J*=244.7 Hz, Ar-C), 135.8 (Ar-C), 131.5 (d, *J*=8.0 Hz, Ar-C),130.7 (d, *J*=3.0 Hz, Ar-C), 129.0 (Ar-C), 128.9 (Ar-C), 126.3 (Ar-C), 123.8 (d, *J*=8.3 Hz, Ar-C), 117.3 (d, *J*=22.4 Hz, Ar-C), 113.3 (d, *J*=22.2 Hz, Ar-C), 100.0 (C7), 94.7 (C2), 81.0 (C4), 68.7 (C5), 36.2 ($C(CH_3)_3$), 25.0 ($(CH_3)_3$), 18.1 (CH_3); δ_F (377 MHz, $CDCl_3$) -116.90; LRMS (ESI$^-$) *m/z*: 423.1 ($[M-H]^-$ 95%); HRMS (ESI$^-$) *m/z*: $[M-H]^-$ calcd. for $C_{24}H_{24}N_2O_4F$, 423.1726, found 423.1726.

(1*R,3*S**,7a*S**)-3-(*tert*-Butyl)-*N*-(4-hexylphenyl)-7-hydroxy-5-oxo-1-phenyl-5,7a-dihydro-1*H*,3*H*-pyrrolo[1,2-*c*]oxazole-6-carboxamide 12m**

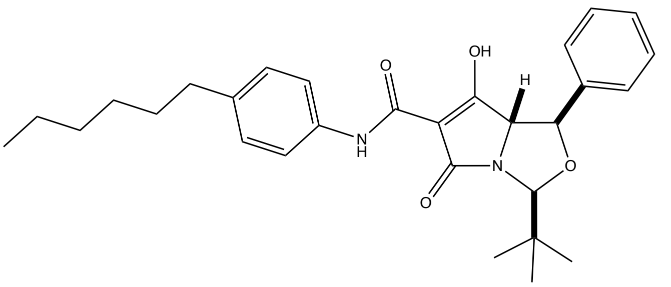

Prepared according to the General Method : tetramate (52 mg, 0.14 mmol), amine (35 mg, 0.2 mmol), toluene (5 mL), Yield (66 mg, 18%); Orange oil; R_f (50% EtOAc in Pet. Ether 40:60) 0.65; ν_{max}/cm^{-1} 3280 (O-H), 2957 (C-H), 2928 (C-H), 2857 (C-H), 1697 (C=O), 1649 (C=O), 1605 (C=C); AB:CD=99:1; δ_H (400 MHz, $CDCl_3$) 9.34 (br s, 1H, NH/OH), 8.54 (br s, 1H, NH/OH), 7.53 (d, 2H, *J*=8.3 Hz, Ar-H), 7.49 (d, 2H, *J*=8.1 Hz, Ar-H), 7.45-7.37 (m, 3H, Ar-H), 7.17 (d, 2H, *J*=8.1 Hz, Ar-H), 5.02 (s, 1H, H2), 4.62 (d, 1H, *J*=8.5 Hz, H4), 4.26 (d, 1H, *J*=8.5 Hz, H5), 2.58 (t, 2H, *J*=7.7 Hz, Alkyl-CH_2), 1.59 (m, 2H, Alkyl-CH_2), 1.33-1.26 (br s, 6H, Alkyl-CH_2), 1.11 (s, 9H, $C(CH_3)_3$), 0.88 (t, 3H, *J*=6.6 Hz, Alkyl-CH_3); $^{13}C\{^1H\}$ δ_C (101 MHz, $CDCl_3$) 184.1 (C6), 174.4 (C8), 164.0 (C9), 140.1 (Ar-C), 135.9 (Ar-C), 134.1 (Ar-C), 129.2 (Ar-C), 129.0 (Ar-C), 128.9 (Ar-C), 126.4 (Ar-C), 120.5 (Ar-C), 99.8 (C7), 94.9 (C2), 81.1 (C4),68.8 (C5), 36.2 ($C(CH_3)_3$), 35.6 (Alkyl-C), 31.9 (Alkyl-C), 31.6 (Alkyl-C), 29.1 (Alkyl-C), 25.1 ($(CH_3)_3$), 22.8 (Alkyl-C), 14.3 (Alkyl-C); LRMS (ESI$^-$) *m/z*: 475.1 ([M – H]$^-$ 96%); HRMS (ESI$^-$) *m/z*: [M – H]$^-$ calcd. for $C_{29}H_{35}N_2O_4$, 475.2602, found 475.2605.

(1*R,3*S**,7a*S**)-3-(*tert*-Butyl)-*N*-cyclohexyl-7-hydroxy-5-oxo-1-phenyl-5,7a-dihydro-1*H*,3*H*-pyrrolo[1,2-*c*]oxazole-6-carboxamide 12n**

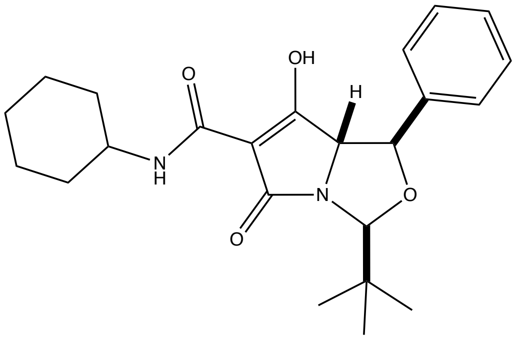

Prepared according to the General Method : tetramate (147 mg, 0.40 mmol), amine (59 mg, 0.60 mmol), toluene (10 mL), Yield (159 mg, 45%); Red/purple foam; m.p. 94-97°C; R_f (50% EtOAc in Pet. Ether 40:60) 0.20; ν_{max}/cm^{-1} 2932 (C-H), 2856 (C-H), 1697 (C=O), 1648 (C=O); AB:CD=82:18; δ_H (400 MHz, $CDCl_3$) 10.51 (br s, 1H, NH/OH), 7.56-7.34 (m, 5H, Ar-H), 4.99 (s, 1H, H2, CD), 4.97 (s, 1H, H2, AB), 4.61 (d, 1H, *J*=9.0 Hz, H4, CD), 4.58 (d, 1H, *J*=8.6 Hz, H4, AB), 4.15 (d, 1H, *J*=8.6 Hz, H5, AB), 3.90 (d, 1H, *J*=8.9 Hz, H5, CD), 3.89-3.80 (m, 1H, Cyclohexyl-CH), 1.96-1.14 (m, 10H, Cyclohexyl-CH_2), 1.07 (s, 9H, $C(CH_3)_3$, AB), 1.05 (s, 9H, $C(CH_3)_3$, CD); $^{13}C\{^1H\}$ δ_C (101 MHz, $CDCl_3$) 190.6 (C6, CD),186.1 (C6, AB), 181.0 (C8, CD), 174.9 (C8, AB), 166.0 (C9, CD),165.5 (C9, AB), 137.1 (Ar-C, CD), 136.2 (Ar-C, AB), 128.8 (Ar-C, AB), 128.7 (Ar-C, CD), 128.4 (Ar-C, CD), 126.4 (Ar-C, CD), 126.3 (Ar-C, AB), 97.2 (C7), 94.9 (C2, CD), 94.8 (C2, AB), 80.7 (C4, AB), 79.5

(C4, CD), 72.5 (C5, CD), 69.2 (C5, AB), 49.6 (Cyclohexyl-C, CD), 48.0 (Cyclohexyl-C, AB), 36.2 ($C(CH_3)_3$), AB), 36.1 ($C(CH_3)_3$), CD), 32.9 (Cyclohexyl-C), 32.8 (Cyclohexyl-C), 25.4 (Cyclohexyl-C), 25.0 ($(CH_3)_3$), 24.7 (Cyclohexyl-C); LRMS (ESI$^-$) *m/z*: 397.2 ([M – H]$^-$ 95%); HRMS (ESI$^-$) *m/z*: calcd. [M – H]$^-$ for $C_{23}H_{29}N_2O_4$, 397.2133, found 397.2127.

(1*R,3*S**,7a*S**)-3-(*tert*-Butyl)-*N*-cycloheptyl-7-hydroxy-5-oxo-1-phenyl-5,7a-dihydro-1*H*,3*H*-pyrrolo[1,2-*c*]oxazole-6-carboxamide 12o**

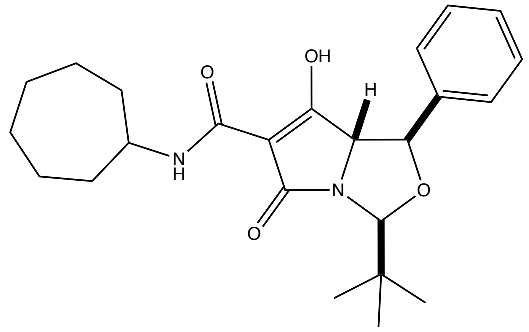

Prepared according to the General Method : tetramate (121 mg, 0.33 mmol), amine (55 mg, 0.50 mmol), toluene (10 mL), Yield (140 mg, 24%); Brown foam; m.p. 59-64°C; R_f (30% EtOAc in Pet. Ether 40:60) 0.20; ν_{max}/cm^{-1} 2929 (C-H), 2858 (C-H), 1695 (C=O), 1645 (C=O); AB:CD=83:17; δ_H (400 MHz, $CDCl_3$) 10.73 (br s, 1H, NH/OH), 7.57-7.33 (m, 5H, Ar-H), 4.99 (s, 1H, H2, CD), 4.97 (s, 1H, H2, AB), 4.61 (d, 1H, *J*=9.0 Hz, H4, CD),4.58 (d, 1H, *J*=8.6 Hz, H4, AB), 4.15 (d, 1H, *J*=8.5 Hz, H5, AB),4.06 (m, 1H, Cycloheptyl-CH), 3.90 (d, 1H, *J*=8.8 Hz, H5, CD), 2.05-1.48 (m, 12H, Cycloheptyl-CH_2), 1.07 (s, 9H, $C(CH_3)_3$, AB), 1.06 (s, 9H, $C(CH_3)_3$, CD); $^{13}C\{^1H\}$ δ_C (101 MHz, $CDCl_3$) 190.6 (C6, CD), 186.1 (C6, AB), 181.0 (C8, CD), 174.9 (C8, AB), 165.7 (C9, CD), 165.2 (C9, AB), 137.1 (Ar-C, CD), 136.2 (Ar-C, AB), 128.8 (Ar-C, AB), 128.7 (Ar-C, CD), 128.4 (Ar-C, CD), 126.4 (Ar-C, CD), 126.3 (Ar-C, AB), 97.2 (C7), 94.9 (C2, CD), 94.8 (C2, AB), 80.7 (C4, AB), 79.5 (C4, CD), 72.5 (C5, CD), 69.2 (C5, AB), 50.8 (Cycloheptyl-C, CD), 50.2 (Cycloheptyl-C, AB), 36.2 ($C(CH_3)_3$), AB), 36.1 ($C(CH_3)_3$), CD), 35.0 (Cycloheptyl-C), 34.9 (Cycloheptyl-C), 28.1 (Cycloheptyl-C, AB), 27.9 (Cycloheptyl-C, CD), 25.0 ($(CH_3)_3$),24.1 (Cycloheptyl-C, CD), 24.0 (Cycloheptyl-C, AB); LRMS (ESI$^-$) *m/z*: 411.1 ([M – H]$^-$ 100%); HRMS (ESI$^+$) *m/z*: [M + H]$^+$ calcd. for $C_{24}H_{33}N_2O_4$, 413.2435, found 413.2432.

(1*R,3*S**,7a*S**)-3-(*tert*-Butyl)-*N*-hexyl-7-hydroxy-5-oxo-1-phenyl-5,7a-dihydro-1*H*,3*H*-pyrrolo[1,2-*c*]oxazole-6-carboxamide 12p**

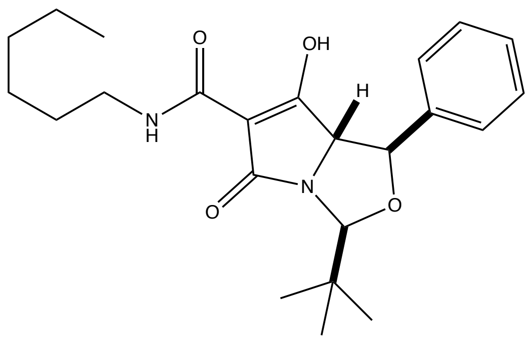

Prepared according to the General Method : tetramate (59 mg, 0.16 mmol), amine (25 mg, 0.24 mmol), toluene (5 mL), Yield (65 mg, 19%); Orange oil; R_f (50% EtOAc in Pet. Ether 40:60) 0.28; ν_{max}/cm^{-1} 3339 (O-H), 2957 (C-H), 2930 (C-H), 2860 (C-H), 1697 (C=O), 1649 (C=O); AB:CD=82:18; δ_H (400 MHz, $CDCl_3$) 10.00 (br s, 1H, NH/OH), 7.55-7.34 (m, 5H, Ar-H), 5.00 (s, 1H, H2, CD), 4.97 (s, 1H, H2, AB), 4.61 (m, 1H, H4, CD), 4.58 (d, 1H, *J*=8.4 Hz, H4, AB), 4.16 (d, 1H, *J*=8.5 Hz, H5, AB), 3.90 (d, 1H, *J*=8.9 Hz, H5, CD), 3.36 (q, 2H, *J*=6.8 Hz, Alkyl-CH_2), 1.68-1.52 (m, 2H, Alkyl-CH_2), 1.40-1.21 (m, 6H, Alkyl-CH_2),1.07 (s, 9H, $C(CH_3)_3$), 0.90 (t, 3H, *J*=6.7 Hz, Alkyl-CH_3); $^{13}C\{^1H\}$ δ_C (101 MHz, $CDCl_3$) 188.4 (C6, CD), 185.6 (C6, AB),181.0 (C8, CD), 174.8 (C8, AB), 167.0 (C9, CD), 166.3 (C9, AB), 137.0 (Ar-C, CD), 136.2 (Ar-C, AB), 129.2 (Ar-C, CD), 128.9 (Ar-C, AB), 128.8 (Ar-C, AB), 128.7 (Ar-C, CD), 126.4 (Ar-C, CD), 126.3 (Ar-C, AB), 97.7 (C7), 95.0 (C2, CD), 94.8 (C2, AB), 80.8 (C4, AB), 79.6 (C4, CD), 72.5 (C5, CD), 69.2 (C5, AB), 39.0 (Alkyl-C), 36.2 ($C(CH_3)_3$), 31.5 (Alkyl-C), 29.5 (Alkyl-C), 26.6 (Alkyl-C), 25.0 ($(CH_3)_3$), 22.6 (Alkyl-C), 14.2 (Alkyl-C); LRMS (ESI$^-$) *m/z*: 399.2 ([M – H]$^-$ 97%); HRMS (ESI$^-$) *m/z*: [M – H]$^-$ calcd. for $C_{23}H_{31}N_2O_4$, 399.2289, found 399.2282.

(1*R,3*S**,7a*S**)-3-(*tert*-Butyl)-7-hydroxy-*N*-nonyl-5-oxo-1-phenyl-5,7a-dihydro-1*H*,3*H*-pyrrolo[1,2-*c*]oxazole-6-carboxamide 12q**

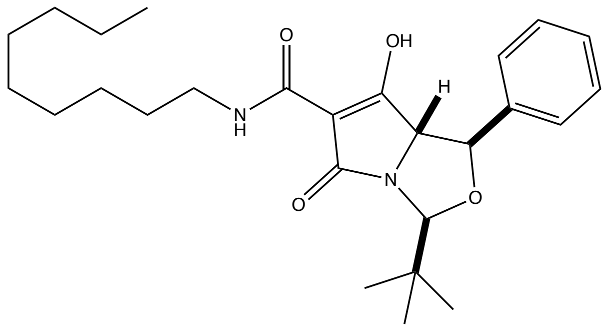

Prepared according to the General Method : tetramate (88 mg, 0.24 mmol), amine (52 mg, 0.36 mmol), toluene (5 mL), Yield (104 mg, 40%); Orange oil; R_f (30% EtOAc in Pet. Ether 40:60) 0.43; ν_{max}/cm^{-1} 2956 (C-H), 2925 (C-H), 2855 (C-H), 1696 (C=O), 1648 (C=O); AB:CD=99:1; δ_H (400 MHz, $CDCl_3$) 10.58 (br s, 1H, NH/OH), 7.53-7.33 (m, 5H, Ar-H), 4.97 (s, 1H, H2), 4.58 (d, 1H, *J*=8.6 Hz, H4), 4.17 (m, 1H, H5), 3.37 (m, 2H, Alkyl-CH_2), 1.58 (m, 2H, Alkyl-CH_2), 1.37-1.19 (m, 12H, Alkyl-CH_2), 1.07 (s, 9H, $C(CH_3)_3$), 0.88 (t, 3H, *J*=7.0 Hz, Alkyl-CH_3); $^{13}C\{^1H\}$ δ_C (101 MHz, $CDCl_3$) 185.6 (C6), 174.8 (C8), 166.3 (C9), 136.2 (Ar-C), 128.8 (Ar-C), 126.3 (Ar-C),97.6 (C7), 94.8 (C2), 80.8 (C4), 69.2 (C5),39.0 (Alkyl-C), 36.2 ($C(CH_3)_3$), 31.9 (Alkyl-C), 29.5-29.3

(Alkyl-C), 26.9 (Alkyl-C), 25.0 (($(CH_3)_3$), 22.8 (Alkyl-C), 14.2 (Alkyl-C); LRMS (ESI$^-$) *m/z*: 441.2 ([M – H]$^-$ 97%); HRMS (ESI$^+$) *m/z*: [M + H]$^+$ calcd. for $C_{26}H_{39}N_2O_4$, 443.2904, found 443.2901.

(1*R,3*S**,7a*S**)-3-(*tert*-Butyl)-*N*-dodecyl-7-hydroxy-5-oxo-1-phenyl-5,7a-dihydro-1*H*,3*H*-pyrrolo[1,2-*c*]oxazole-6-carboxamide 12r**

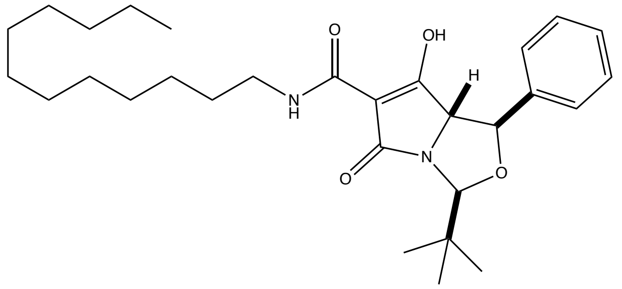

Prepared according to the General Method : tetramate (125 mg, 0.34 mmol), amine (94 mg, 0.51 mmol), toluene (10 mL), Yield (163 mg, 57%); Orange oil; R_f (30% EtOAc in Pet. Ether 40:60) 0.28; ν_{max}/cm^{-1} 2924 (C-H), 2854 (C-H), 1697 (C=O), 1648 (C=O); AB:CD=84:16; δ_H (400 MHz, $CDCl_3$) 10.51 (br s, 1H, NH/OH), 7.56-7.32 (m, 5H, Ar-H), 5.00 (s, 1H, H2, CD), 4.97 (s, 1H, H2, AB), 4.60 (m, 1H, H4, CD), 4.58 (d, 1H, *J*=8.5 Hz, H4, AB), 4.16 (d, 1H, *J*=8.6 Hz, H5, AB), 3.90 (d, 1H, *J*=8.9 Hz, H5, CD), 3.36 (m, 2H, Alkyl-CH_2), 1.64-1.54 (m, 2H, Alkyl-CH_2), 1.37-1.21 (br m, 18H, Alkyl-CH_2), 1.07 (s, 9H, $C(CH_3)_3$, AB), 1.06 (s, 9H, $C(CH_3)_3$, CD), 0.88 (t, 3H, *J*=6.7 Hz, Alkyl-CH_3); $^{13}C\{^1H\}$ δ_C (101 MHz, $CDCl_3$) 190.7 (C6, CD), 185.6 (C6, AB), 180.4 (C8, CD), 174.8 (C8, AB), 167.0 (C9, CD), 166.3 (C9, AB), 137.0 (Ar-C, CD), 136.2 (Ar-C, AB), 129.4 (Ar-C, CD), 128.8 (Ar-C, AB), 128.7 (Ar-C, CD), 126.5 (Ar-C, CD), 126.3 (Ar-C, AB), 97.7 (C7), 95.0 (C2, CD), 94.8 (C2, AB), 80.8 (C4, AB), 79.6 (C4, CD), 72.5 (C5, CD), 69.2 (C5, AB), 39.0 (Alkyl-C, AB), 38.9 (Alkyl-C, CD), 36.2 ($C(CH_3)_3$, AB), 36.1 ($C(CH_3)_3$, CD), 32.0 (Alkyl-C), 29.8-29.4 (Alkyl-C), 27.0 (Alkyl-C, CD), 27.0 (Alkyl-C, AB), 25.1 (($(CH_3)_3$), CD), 25.0 (($(CH_3)_3$), AB), 22.8 (Alkyl-C), 14.3 (Alkyl-C); LRMS (ESI$^-$) *m/z*: 483.3 ([M – H]$^-$ 97%); HRMS (ESI$^-$) *m/z*: [M – H]$^-$ calcd. for $C_{29}H_{43}N_2O_4$, 483.3228, found 483.3234.

(1*R,3*S**,7a*S**)-3-(*tert*-Butyl)-*N*-(4-cyclohexylphenyl)-7-hydroxy-5-oxo-1-phenyl-5,7a-dihydro-1*H*,3*H*-pyrrolo[1,2-*c*]oxazole-6-carboxamide 12s**

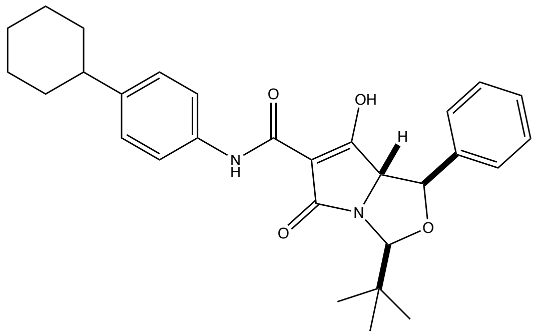

Prepared according to the General Method : tetramate (85 mg, 0.23 mmol), amine (61 mg, 0.35 mmol), toluene (5 mL), Yield (113 mg, 25%); Brown foamy solid; m.p. 97-101°C; R_f (50% EtOAc in Pet. Ether 40:60) 0.50; v_{max}/cm^{-1} 3272 (O-H), 2925 (C-H), 2852 (C-H), 1697 (C=O), 1649 (C=O), 1604 (C=C); AB:CD=99:1; δ_H (400 MHz, $CDCl_3$) 9.32 (br s, 1H, NH/OH), 7.54-7.19 (m, 9H, Ar-H), 5.02 (s, 1H, H2), 4.63 (d, 1H, *J*=8.5 Hz, H4), 4.26 (d, 1H, *J*=8.5 Hz, H5), 2.48 (m, 1H, Cyclohexyl-CH), 1.88-1.73 (m, 6H, Cyclohexyl-CH_2), 1.42-1.37 (m, 4H, Cyclohexyl-CH_2), 1.11 (s, 9H, $C(CH_3)_3$); $^{13}C\{^1H\}$ δ_C (101 MHz, $CDCl_3$) 184.1 (C6), 174.4 (C8), 164.0 (C9), 145.3 (Ar-C), 135.9 (Ar-C), 134.2 (Ar-C), 129.1 (Ar-C), 128.9 (Ar-C),127.6 (Ar-C), 126.4 (Ar-C), 120.6 (Ar-C), 99.8 (C7), 94.9 (C2), 81.0 (C4), 68.8 (C5), 44.2 (Cyclohexyl-C), 36.2 ($C(CH_3)_3$), 34.6 (Cyclohexyl-C), 27.0 (Cyclohexyl-C), 26.3 (Cyclohexyl-C), 25.1 ($(CH_3)_3$); LRMS (ESI^-) *m/z*: 473.1 ($[M - H]^-$ 100%); HRMS (ESI^-) *m/z*: $[M - H]^-$ calcd. for $C_{29}H_{33}N_2O_4$, 473.2446, found 473.2449.

(1*R,3*S**,7a*S**)-3-(*tert*-Butyl)-7-hydroxy-5-oxo-1-phenyl-*N*-(tetrahydro-2*H*-pyran-4-yl)-5,7a-dihydro-1*H*,3*H*-pyrrolo[1,2-*c*]oxazole-6-carboxamide 12t**

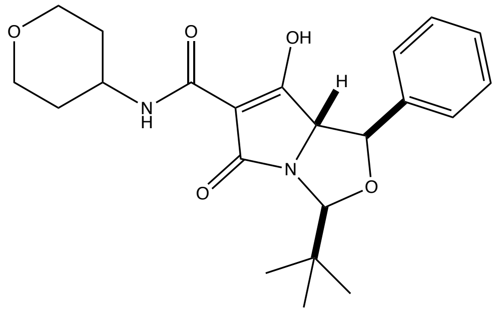

Prepared according to the General Method : tetramate (88 mg, 0.24 mmol), amine (36 mg, 0.36 mmol), toluene (10 mL), Yield (96 mg, 31%); Brown/orange foamy solid; m.p. 100-103°C; R_f (100% EtOAc) 0.10; v_{max}/cm^{-1} 3324 (O-H), 2960 (C-H), 2849 (C-H), 1692 (C=O), 1645 (C=O); AB:CD=88:12; δ_H (400 MHz, $CDCl_3$) 10.51 (br s, 1H, NH/OH), 7.56-7.33 (m, 5H, Ar-H), 4.99 (s, 1H, H2, CD), 4.97 (s, 1H, H2, AB), 4.62 (d, 1H, *J*=9.0 Hz, H4, CD),4.58 (d, 1H, *J*=8.6 Hz, H4, AB), 4.19 (d, 1H, *J*=8.6 Hz, H5, AB), 4.08 (m, 1H, Pyran-CH), 3.98 (m, 2H, Pyran-CH_2), 3.92 (d, 1H, *J*=8.9 Hz, H5, CD), 3.50 (m, 2H, Pyran-CH_2), 1.97-1.87 (m, 2H, Pyran-CH_2), 1.68-1.55 (m, 2H, Pyran-CH_2), 1.07 (s, 9H, $C(CH_3)_3$, AB), 1.06 (s, 9H, $C(CH_3)_3$, CD); $^{13}C\{^1H\}$ δ_C (101 MHz, $CDCl_3$) 190.6 (C6, CD),184.9 (C6, AB), 181.1 (C8, CD), 174.6 (C8, AB), 166.4 (C9, CD), 165.6 (C9, AB), 136.9 (Ar-C, CD), 136.1 (Ar-C, AB), 128.9 (Ar-C, AB), 128.9 (Ar-C, AB), 128.7 (Ar-C, CD), 126.4 (Ar-C, CD), 126.3 (Ar-C, AB),98.5 (C7), 95.0 (C2, CD),94.9 (C2, AB), 80.9 (C4, AB), 79.5 (C4, CD), 72.6 (C5, CD), 69.0 (C5, AB), 66.6 (Pyran-C, AB), 66.4 (Pyran-C, CD), 47.0 (Pyran-C, CD), 45.3 (Pyran-C,

AB), 36.2 ($C(CH_3)_3$, AB), 36.1 ($C(CH_3)_3$, CD), 32.9 (Pyran-C, AB), 32.8 (Pyran-C, AB), 32.7 (Pyran-C, CD), 25.1 ($(CH_3)_3$, CD), 25.0 ($(CH_3)_3$, AB); LRMS (ESI$^-$) *m/z*: 399.1 ($[M - H]^-$ 96%); HRMS (ESI$^-$) *m/z*: $[M - H]^-$ calcd. for $C_{22}H_{27}N_2O_5$, 399.1926, found 399.1925.

(1*R,3*S**,7a*S**)-*N*-Benzyl-3-(*tert*-butyl)-7-hydroxy-5-oxo-1-phenyl-5,7a-dihydro-1*H*,3*H*-pyrrolo[1,2-*c*]oxazole-6-carboxamide 12u**

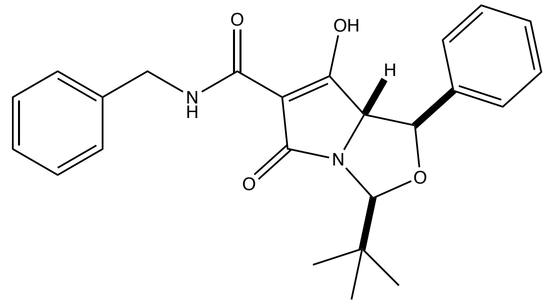

Prepared according to the General Method : tetramate (88 mg, 0.24 mmol), amine (39 mg, 0.36 mmol), toluene (10 mL), Yield (98 mg, 33%); Orange oil; R_f (50% EtOAc in Pet. Ether 40:60) 0.20; ν_{max}/cm^{-1} 3326 (O-H), 2967 (C-H), 1695 (C=O), 1647 (C=O); AB:CD=99:1; δ_H (400 MHz, $CDCl_3$) 7.87 (br m, 1H, NH), 7.53-7.30 (m, 10H, Ar-H), 4.96 (s, 1H, H2), 4.59 (d, 1H, *J*=8.6 Hz, H4), 4.56 (dd, 2H, *J*=6.2 Hz, 1.7 Hz, $NHCH_2$), 4.20 (d, 1H, *J*=8.5 Hz, H5), 1.07 (s, 9H, $C(CH_3)_3$); $^{13}C\{^1H\}$ δ_C (101 MHz, $CDCl_3$) 184.6 (C6), 174.5 (C8), 166.0 (C9), 137.1 (Ar-C), 136.0 (Ar-C), 129.0 (Ar-C),128.9 (Ar-C), 127.9 (Ar-C), 126.3 (Ar-C), 98.8 (C7), 94.8 (C2), 80.9 (C4), 68.9 (C5), 42.8 ($NHCH_2$), 36.2 ($C(CH_3)_3$), 25.0 ($(CH_3)_3$); LRMS (ESI$^-$) *m/z*: 405.1 ($[M - H]^-$ 100%); HRMS (ESI$^+$) *m/z*: $[M + H]^+$ calcd. for $C_{24}H_{27}N_2O_4$, 407.1965, found 407.1967.

(1*R,3*S**,7a*S**)-*N*,3-Di-*tert*-butyl-7-hydroxy-5-oxo-1-phenyl-5,7a-dihydro-1*H*,3*H*-pyrrolo[1,2-*c*]oxazole-6-carboxamide 12v**

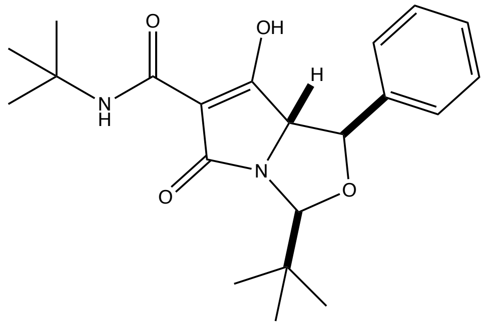

Prepared according to the General Method : tetramate (92 mg, 0.25 mmol), amine (28 mg, 0.38 mmol), toluene (10 mL), Yield (92 mg, 41%); Orange oil; R_f (50% EtOAc in Pet. Ether 40:60) 0.63; ν_{max}/cm^{-1} 3323 (O-H), 2968 (C-H), 1697 (C=O), 1649 (C=O), 1623 (C=C); AB:CD=82:18; δ_H (400 MHz, $CDCl_3$) 9.40 (br s, 1H, NH/OH), 7.57-7.36 (m, 5H, Ar-H), 4.99 (s, 1H, H2, CD), 4.96 (s, 1H, H2, AB), 4.61 (d, 1H, *J*=8.9 Hz, H4, CD), 4.58 (d, 1H, *J*=8.6 Hz, H4, AB), 4.13 (d, 1H, *J*=8.6 Hz, H5, AB), 3.91 (d, 1H, *J*=8.8 Hz, H5, CD), 1.47 (s, 9H, $NHC(CH_3)_3$, CD), 1.44 (s, 9H, $NHC(CH_3)_3$, AB), 1.07 (s, 9H, $C(CH_3)_3$, AB), 1.06 (s, 9H, $C(CH_3)_3$, CD); $^{13}C\{^1H\}$ δ_C (101 MHz, $CDCl_3$) 190.5 (C6, CD),

186.5 (C6, AB), 181.2 (C8, CD), 175.1 (C8, AB),167.2 (C9, CD), 166.3 (C9, AB), 137.1 (Ar-C, CD), 136.3 (Ar-C, AB), 128.8 (Ar-C, AB), 128.7 (Ar-C, CD), 126.4 (Ar-C, CD), 126.3 (Ar-C, AB), 97.3 (C7), 94.9 (C2, CD), 94.8 (C2, AB), 80.7 (C4, AB), 79.5 (C4, CD), 72.5 (C5, CD), 69.3 (C5, AB), 53.8 ($NHC(CH_3)_3$, CD), 52.3 ($NHC(CH_3)_3$, AB), 36.2 ($C(CH_3)_3$, AB), 36.2 ($C(CH_3)_3$, CD), 29.3 ($NC(CH_3)_3$, CD), 29.0 ($NC(CH_3)_3$, AB), 25.1 ($(CH_3)_3$); LRMS (ESI$^-$) *m/z*: 371.1 ([M – H]$^-$ 96%); HRMS (ESI$^+$) *m/z*: [M + H]$^+$ calcd. for $C_{21}H_{29}N_2O_4$, 373.2122, found 373.2124.

(1*R,3*S**,7a*S**)-3-(*tert*-Butyl)-*N*-(cyclohexylmethyl)-7-hydroxy-5-oxo-1-phenyl-5,7a-dihydro-1*H*,3*H*-pyrrolo[1,2-*c*]oxazole-6-carboxamide 12w**

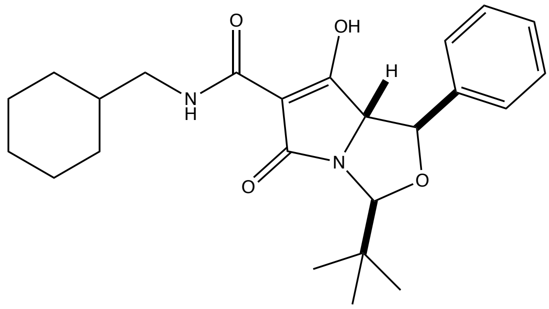

Prepared according to the General Method : tetramate (74 mg, 0.20 mmol), amine (34 mg, 0.30 mmol), toluene (10 mL), Yield (96 mg, 37%); Red/orange oil; R_f (50% EtOAc in Pet. Ether 40:60) 0.38; v_{max}/cm^{-1} 3337 (O-H), 2925 (C-H), 2853 (C-H), 1696 (C=O), 1648 (C=O); AB:CD=99:1; δ_H (400 MHz, $CDCl_3$) 10.61 (br s, 1H, OH), 7.59 (br t, 1H, *J*=6.4 Hz, NH), 7.53-7.33 (m, 5H, Ar-H), 4.98 (s, 1H, H2), 4.58 (d, 1H, *J*=8.5 Hz, H4), 4.16 (d, 1H, *J*=8.5 Hz, H5), 3.28-3.10 (m, 2H, $NHCH_2$), 1.78-1.65 (m, 6H, Cyclohexyl-CH_2), 1.58-1.46 (m, 1H, Cyclohexyl-CH), 1.30-1.13 (m, 4H, Cyclohexyl-CH_2), 1.07 (s, 9H, $C(CH_3)_3$); $^{13}C\{^1H\}$ δ_C (101 MHz, $CDCl_3$) 185.6 (C6), 174.8 (C8), 166.3 (C9), 136.2 (Ar-C), 128.8 (Ar-C), 126.3 (Ar-C), 97.6 (C7), 94.8 (C2), 80.7 (C4), 69.2 (C5), 45.0 (NCH_2), 37.9 (Cyclohexyl-C), 36.2 ($C(CH_3)_3$), 30.8 (Cyclohexyl-C), 26.3 (Cyclohexyl-C), 25.8 (Cyclohexyl-C), 25.0 ($(CH_3)_3$); LRMS (ESI$^-$) *m/z*: 411.1 ([M – H]$^-$ 96%); HRMS (ESI$^+$) *m/z*: [M + H]$^+$ calcd. for $C_{24}H_{33}N_2O_4$, 413.2435, found 413.2433.

(1*R,3*S**,7a*S**)-*N*-(Adamantan-1-yl)-3-(*tert*-butyl)-7-hydroxy-5-oxo-1-(*p*-tolyl)-5,7a-dihydro-1*H*,3*H*-pyrrolo[1,2-*c*]oxazole-6-carboxamide 13a**

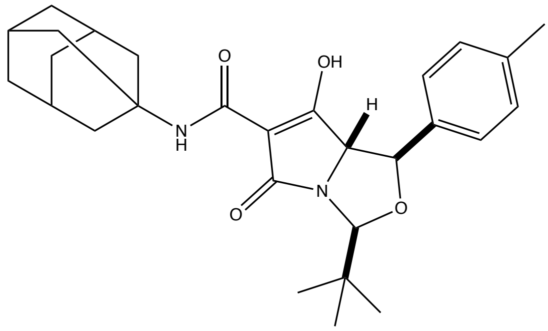

Prepared according to the General Method : tetramate (58 mg, 0.15 mmol), amine (35 mg, 0.23 mmol), toluene (5 mL), Yield (70 mg, 52%); Brown foamy solid; m.p. 124-127°C; R_f (50% EtOAc in Pet. Ether 40:60) 0.83; v_{max}/cm^{-1} 3312 (O-H), 2909 (C-H), 2852 (C-H), 1695 (C=O), 1647 (C=O); AB:CD=80:20; δ_H (400

MHz, $CDCl_3$) 9.92 (br s, 1H, NH/OH), 7.40 (d, 2H, *J*=8.0 Hz, Ar-H), 7.21 (d, 2H, *J*=7.9 Hz, Ar-H), 4.97 (s, 1H, H2, CD), 4.94 (s, 1H, H2, AB), 4.57 (d, 1H, *J*=8.9 Hz, H4, CD), 4.53 (d, 1H, *J*=8.6 Hz, H4, AB), 4.10 (d, 1H, *J*=8.6 Hz, H5, AB), 3.88 (d, 1H, *J*=8.8 Hz, H5, CD), 2.36 (s, 3H, CH_3, AB), 2.35 (s, 3H, CH_3, CD), 2.12 (br s, 3H, Adamantyl-CH), 2.07 (m, 6H, Adamantyl-CH_2), 1.70 (m, 6H, Adamantyl-CH_2), 1.06 (s, 9H, $C(CH_3)_3$, AB), 1.05 (s, 9H, $C(CH_3)_3$, CD); $^{13}C\{^1H\}$ δ_C (101 MHz, $CDCl_3$) 190.5 (C6, CD), 186.6 (C6, AB), 181.2 (C8, CD), 175.1 (C8, AB), 167.0 (C9, CD), 166.2 (C9, AB), 138.6 (Ar-C, AB), 138.1 (Ar-C, CD), 134.1 (Ar-C, CD), 133.3 (Ar-C, AB), 129.5 (Ar-C, AB), 129.4 (Ar-C, CD),126.4 (Ar-C, CD), 126.3 (Ar-C, AB), 97.2 (C7), 94.8 (C2, CD), 94.8 (C2, AB), 80.7 (C4, AB), 79.5 (C4, CD), 72.5 (C5, CD), 69.3 (C5, AB), 54.5 (Adamantyl-C, CD), 53.0 (Adamantyl-C, AB), 42.7 (Adamantyl-C, CD), 41.7 (Adamantyl-C, AB), 36.2 (Adamantyl-C, AB), 36.2 ($C(CH_3)_3$), 36.0 (Adamantyl-C, CD), 29.5 (Adamantyl-C, AB), 29.4 (Adamantyl-C, CD), 25.1 ($(CH_3)_3$), 21.4 (CH_3); LRMS (ESI$^-$) *m/z*: 463.2 ($[M - H]^-$ 97%); HRMS (ESI$^+$) *m/z*: $[M + H]^+$ calcd. for $C_{28}H_{37}N_2O_4$, 465.2748, found 465.2748.

(1*R,3*S**,7a*S**)-3-(*tert*-Butyl)-*N*-(4-chloro-2-methylphenyl)-7-hydroxy-5-oxo-1-(*p*-tolyl)-5,7a-dihydro-1*H*,3*H*-pyrrolo[1,2-*c*]oxazole-6-carboxamide 13b**

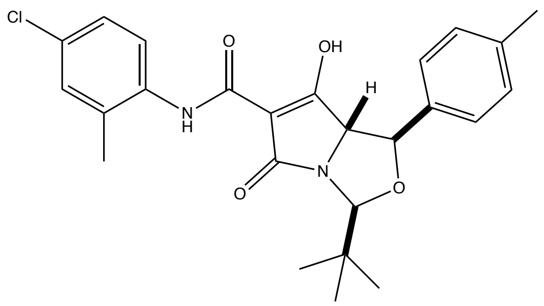

Prepared according to the General Method : tetramate (19 mg, 0.05 mmol), amine (mg, 0.075 mmol), toluene (5 mL), Yield (24 mg, 7%); Red oil; R_f (50% EtOAc in Pet. Ether 40:60) 0.25; ν_{max}/cm^{-1} 3272 (O-H), 2960 (C-H), 1694 (C=O), 1635 (C=O); AB:CD=99:1; δ_H (400 MHz, $CDCl_3$) 9.40 (br s, 1H, NH/OH), 8.01 (d, 1H, *J*=8.3 Hz, Ar-H), 7.41 (d, 2H, *J*=7.9 Hz, Ar-H), 7.23 (d, 2H, *J*=7.9 Hz, Ar-H), 7.19 (m, 2H, Ar-H), 5.01 (s, 1H, H2), 4.59 (d, 1H, *J*=8.5 Hz, H4), 4.27 (d, 1H, *J*=8.5 Hz, H5), 2.38 (s, 3H, CH_3), 2.37 (s, 3H, CH_3), 1.10 (s, 9H, $C(CH_3)_3$); $^{13}C\{^1H\}$ δ_C (101 MHz, $CDCl_3$) 183.8 (C6), 174.4 (C8), 164.1 (C9), 139.0 (Ar-C), 133.6 (Ar-C), 132.8 (Ar-C), 130.5 (Ar-C), 130.4 (Ar-C), 130.3 (Ar-C),129.6 (Ar-C), 126.8 (Ar-C), 126.4 (Ar-C), 123.0 (Ar-C), 100.3 (C7), 94.8 (C2), 81.2 (C4), 68.8 (C5), 36.2 ($C(CH_3)_3$), 25.1 ($(CH_3)_3$), 21.4 (CH_3), 17.9 (CH_3); LRMS (ESI$^-$) *m/z*: 453.1 ($[M^{35} - H]^-$ 96%), 455.1 ($[M^{37} - H]^-$ 34%); HRMS (ESI$^-$) *m/z*: $[M^{35} - H]^-$ calcd. for $C_{25}H_{26}N_2O_4Cl$, 453.1587, found 453.1587; $[M^{37} - H]^-$ calcd. for $C_{25}H_{26}N_2O_4Cl$, 455.1557, found 455.1549.

(1*R,3*S**,7a*S**)-*N*-(Adamantan-1-yl)-3-(*tert*-butyl)-1-(4-fluorophenyl)-7-hydroxy-5-oxo-5,7a-dihydro-1*H*,3*H*-pyrrolo[1,2-*c*]oxazole-6-carboxamide 20**

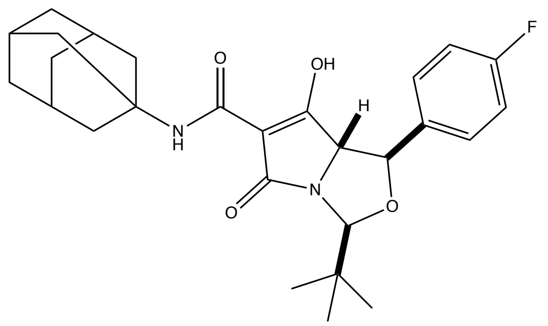

Prepared according to the General Method : tetramate (155 mg, 0.4 mmol), amine (91 mg, 0.6 mmol), toluene (10 mL), Yield (182 mg, 70%); Brown foam; m.p. 126-128°C; R_f (50% EtOAc in Pet. Ether 40:60) 0.50; ν_{max}/cm^{-1} 2909 (C-H), 2852 (C-H), 1693 (C=O), 1646 (C=O), 1625 (C=C); AB:CD=78:22; δ_H (400 MHz, $CDCl_3$) 12.22 (br s, 1H, NH/OH), 7.55-7.47 (m, 2H, Ar-H), 7.42 (br s, 1H, NH/OH), 7.09-7.03 (m, 2H, Ar-H), 4.97 (s, 1H, H2, CD), 4.94 (s, 1H, H2, AB), 4.57 (d, 1H, *J*=9.3 Hz, H4, CD), 4.53 (d, 1H, *J*=8.9 Hz, H4, AB), 4.04 (d, 1H, *J*=8.8 Hz, H5, AB), 3.83 (d, 1H, *J*=8.7 Hz, H5, CD), 2.10 (m, 3H, Adamantyl-CH), 2.06 (6H, br m, Adamantyl-CH_2), 1.69 (6H, br m, Adamantyl-CH_2), 1.04 (s, 9H, $C(CH_3)_3$, AB), 1.03 (s, 9H, $C(CH_3)_3$, CD); $^{13}C\{^1H\}$ δ_C (101 MHz, $CDCl_3$) 190.4 (C6, CD), 187.1 (C6, AB), 181.1 (C8, CD), 175.1 (C8, AB), 167.0 (C9, CD), 166.2 (C9, AB), 162.9 (d, *J*=247.2 Hz, Ar-C, AB), 162.7 (d, *J*=246.3 Hz, Ar-C, CD), 133.0 (Ar-C, CD), 132.2 (Ar-C, AB), 128.2 (obscured by Ar-C/AB, Ar-C, CD), 128.1 (d, *J*=8.4 Hz, Ar-C, AB), 115.7 (d, *J*=21.3 Hz, Ar-C, AB), 115.5 (d, *J*=21.5 Hz, Ar-C, CD), 96.5 (C7), 94.9 (C2, CD), 94.9 (C2, AB), 80.0 (C4, AB), 78.9 (C4, CD), 72.3 (C5, CD), 69.4 (C5, AB), 54.6 (Adamantyl-C, CD), 53.1 (Adamantyl-C, AB), 41.7 (Adamantyl-C, AB), 41.4 (Adamantyl-C, CD), 36.2 (Adamantyl-C, AB), 36.1 ($C(CH_3)_3$), 36.0 (Adamantyl-C, CD), 29.4 (Adamantyl-C), 25.0 ($(CH_3)_3$); δ_F (377 MHz, $CDCl_3$) -113.18 (AB), -113.97 (CD); LRMS (ESI^-) *m/z*: 467.1 ($[M – H]^-$ 94%); HRMS (ESI^-) *m/z*: $[M – H]^-$ calcd. for $C_{27}H_{32}N_2O_4F$, 467.2352, found 467.2352.

(1*R,3*S**,7a*S**)-*N*-(Adamantan-1-yl)-3-(*tert*-butyl)-1-(4-chlorophenyl)-7-hydroxy-5-oxo-5,7a-dihydro-1*H*,3*H*-pyrrolo[1,2-*c*]oxazole-6-carboxamide 21**

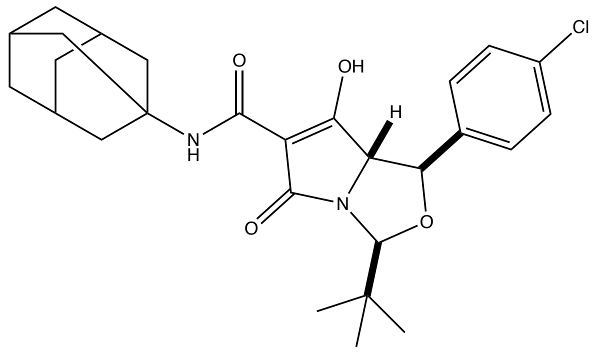

Prepared according to the General Method : tetramate (118 mg, 0.31 mmol), amine (71 mg, 0.47 mmol), toluene (10 mL), Yield (155 mg, 44%); Brown/red foamy solid; m.p. 107-110°C; R_f (50% EtOAc in Pet. Ether 40:60) 0.48; ν_{max}/cm^{-1} 2909 (C-H), 2852 (C-H), 1693 (C=O), 1647 (C=O); AB:CD=76:24; δ_H (400 MHz, $CDCl_3$) 10.29 (br s, 1H, NH/OH),

7.47 (d, 2H, *J*=8.5 Hz, Ar-H), 7.42 (br s, 1H, NH/OH), 7.37 (d, 2H, *J*=8.6 Hz, Ar-H), 4.99 (s, 1H, H2, CD), 4.96 (s, 1H, H2, AB), 4.58 (d, 1H, *J*=8.9 Hz, H4, CD), 4.55 (d, 1H, *J*=8.6 Hz, H4, AB), 4.04 (d, 1H, *J*=8.6 Hz, H5, AB), 3.84 (d, 1H, *J*=8.9 Hz, H5, CD), 2.13 (m, 3H, Adamantyl-CH), 2.07 (m, 6H, Adamantyl-CH_2), 1.70 (m, 6H, Adamantyl-CH_2), 1.05 (s, 9H, $C(CH_3)_3$, AB), 1.03 (9H, s, $C(CH_3)_3$, CD); $^{13}C\{^1H\}$ δ_C (101 MHz, $CDCl_3$) 190.3 (C6, CD), 187.3 (C6, AB), 181.1 (C8, CD), 175.1 (C8, AB), 167.0 (C9, CD), 166.3 (C9, AB), 135.8 (Ar-C, CD), 135.0 (Ar-C, AB), 134.6 (Ar-C, AB), 134.1 (Ar-C, CD), 129.0 (Ar-C, AB), 128.9 (Ar-C, CD), 127.8 (Ar-C, CD), 127.7 (Ar-C, AB), 96.3 (C7), 95.0 (C2, CD), 94.9 (C2, AB), 79.9 (C4, AB), 78.7 (C4, CD), 72.3 (C5, CD), 69.4 (C5, AB),54.6 (Adamantyl-C, CD),53.2 (Adamantyl-C, AB), 41.7 (Adamantyl-C), 36.2 (Adamantyl-C, AB), 36.2 ($C(CH_3)_3$), 36.0 (Adamantyl-C, CD), 29.5 (Adamantyl-C, AB), 29.4 (Adamantyl-C, CD), 25.0 ($(CH_3)_3$); LRMS (ESI$^-$) *m/z*: 483.1 ([M^{35} – H]$^-$ 100%), 485.1 ([M^{37} – H]$^-$ 41%); HRMS (ESI$^+$) *m/z*: [M^{35} + H]$^+$ calcd. for $C_{27}H_{34}N_2O_4Cl$, 485.2207, found 485.2203; [M^{37} + H]$^+$ calcd. for $C_{27}H_{34}N_2O_4Cl$, 487.2178, found 487.2171.

(1*R,3*S**,7a*S**)-*N*-(Adamantan-1-yl)-1-(4-bromophenyl)-3-(*tert*-butyl)-7-hydroxy-5-oxo-5,7a-dihydro-1*H*,3*H*-pyrrolo[1,2-*c*]oxazole-6-carboxamide 22**

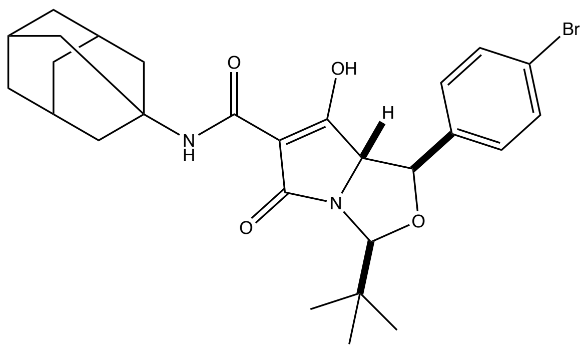

Prepared according to the General Method : tetramate (127 mg, 0.3 mmol), amine (68 mg, 0.45 mmol), toluene (10 mL), Yield (156 mg, 49%); Brown foamy solid; m.p. 107-108°C; R_f (50% EtOAc in Pet. Ether 40:60) 0.50; ν_{max}/cm^{-1} 3311 (O-H), 2909 (C-H), 2852 (C-H), 1697 (C=O), 1649 (C=O), 1626 (C=C); AB:CD=77:23; δ_H (400 MHz, $CDCl_3$) 10.17 (br s, 1H, NH/OH), 7.53-7.39 (m, 4H, Ar-H), 4.98 (s, 1H, H2, CD), 4.95 (s, 1H, H2, AB), 4.56 (d, 1H, *J*=9.2 Hz, H4, CD), 4.53 (d, 1H, *J*=8.7 Hz, H4, AB), 4.03 (d, 1H, *J*=8.6 Hz, H5, AB), 3.83 (d, 1H, *J*=8.9 Hz, H5, CD), 2.12 (m, 3H, Adamantyl-CH), 2.06 (m, 6H, Adamantyl-CH_2), 1.69 (m, 6H, Adamantyl-CH_2), 1.04 (s, 9H, $C(CH_3)_3$, AB), 1.02 (s, 9H, $C(CH_3)_3$, CD); $^{13}C\{^1H\}$ δ_C (101 MHz, $CDCl_3$) 190.3 (C6, CD), 187.3 (C6, AB), 181.1 (C8, CD), 175.1 (C8, AB), 166.9 (C9, CD), 166.3 (C9, AB), 136.3 (Ar-C, CD), 135.5 (Ar-C, AB), 132.0 (Ar-C, AB), 131.8 (Ar-C, CD), 128.1 (Ar-C, CD), 128.0 (Ar-C, AB), 122.7 (Ar-C, AB), 122.3 (Ar-C, CD), 96.3 (C7), 95.0 (C2, CD), 94.9 (C2, AB), 79.8 (C4, AB), 78.7 (C4, CD), 72.2 (C5, CD), 69.3 (C5, AB), 54.6 (Adamantyl-C, CD), 53.2 (Adamantyl-C, AB), 41.7 (Adamantyl-C, AB), 41.5 (Adamantyl-C, CD),

36.2 (Adamantyl-C, AB), 36.2 ($C(CH_3)_3$), 36.0 (Adamantyl-C, CD), 29.5 (Adamantyl-C, CD), 29.4 (Adamantyl-C, AB), 25.0 ($(CH_3)_3$); LRMS (ESI$^-$) *m/z*: 527.0 ([M^{79} – H]$^-$ 96%), 529.0 ([M^{81} – H]$^-$ 92%); HRMS (ESI$^-$) *m/z*: [M^{79} – H]$^-$ calcd. for $C_{27}H_{32}N_2O_4Br$, 527.1551, found 527.1555; [M^{81} – H]$^-$ $C_{27}H_{32}N_2O_4Br$, 529.1531, found 529.1537.

X-ray Crystallographic Data

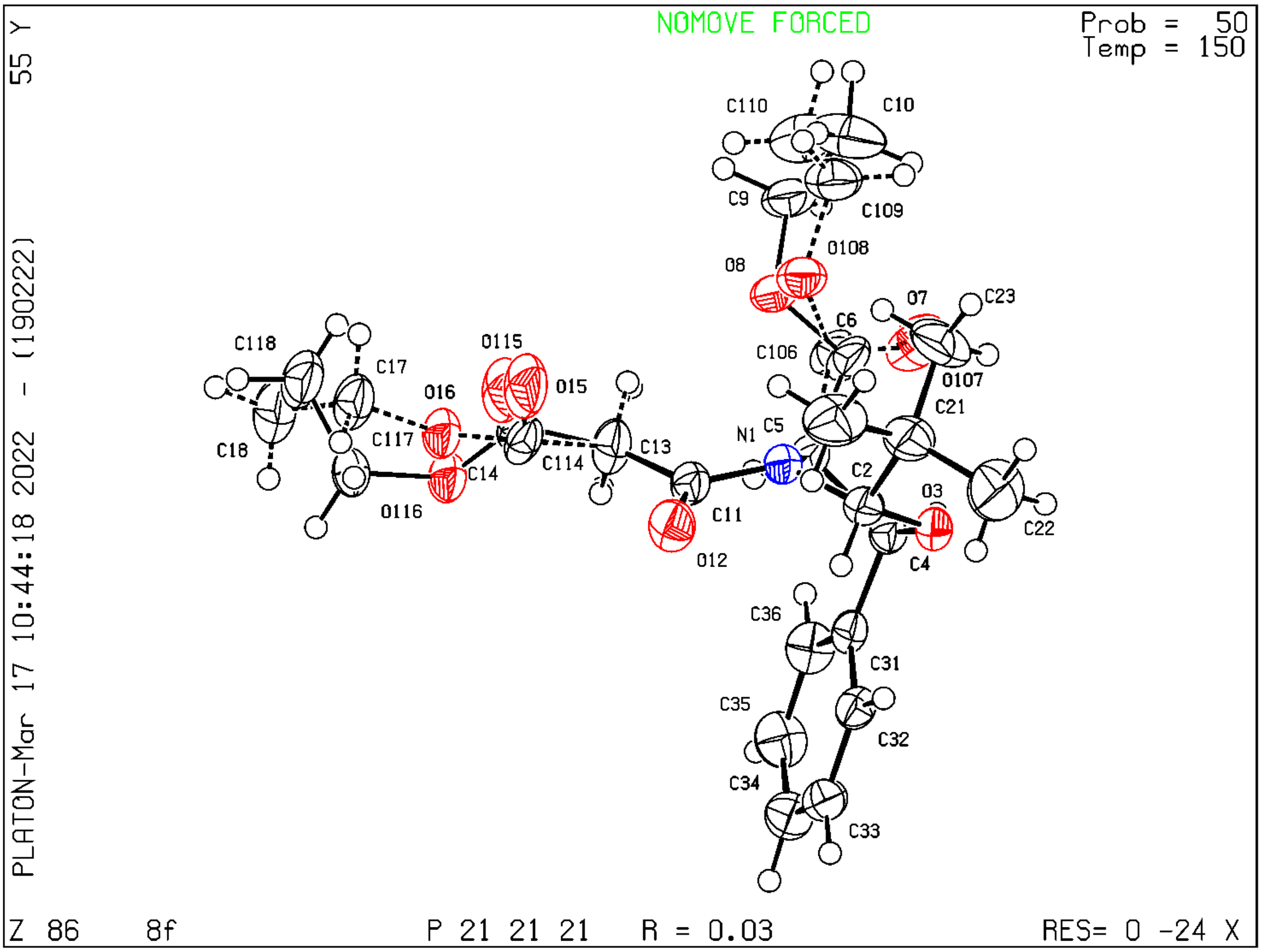

Thermal ellipsoid plot for structure **8f** with ellipsoid contour at 50% probability level.

Bond precision: C-C = 0.0023 A Wavelength=1.54184

Cell:	a=10.0615(1)	b=12.3970(2)	c=16.9654(3)
	alpha=90	beta=90	gamma=90
Temperature:	150 K		

	Calculated	Reported
Volume	2116.14(5)	2116.14(5)
Space group	P 21 21 21	P 21 21 21
Hall group	P 2ac 2ab	P 2ac 2ab
Moiety formula	C21 H29 N O6	C21 H29 N O6
Sum formula	C21 H29 N O6	C21 H29 N O6
Mr	391.45	391.46
Dx,g cm-3	1.229	1.229
Z	4	4
Mu (mm-1)	0.738	0.738
F000	840.0	840.0
F000'	842.72	
h,k,lmax	12,15,21	12,15,21
Nref	4462[2535]	4421
Tmin,Tmax	0.915,0.950	0.840,0.950
Tmin'	0.807	

Correction method= # Reported T Limits: Tmin=0.840 Tmax=0.950
AbsCorr = MULTI-SCAN

Data completeness= 1.74/0.99 Theta(max)= 76.599

R(reflections)= 0.0334(4175) wR2(reflections)= 0.0867(4421)

S = 1.006 Npar= 317

Crystal parameters for structure **8f**

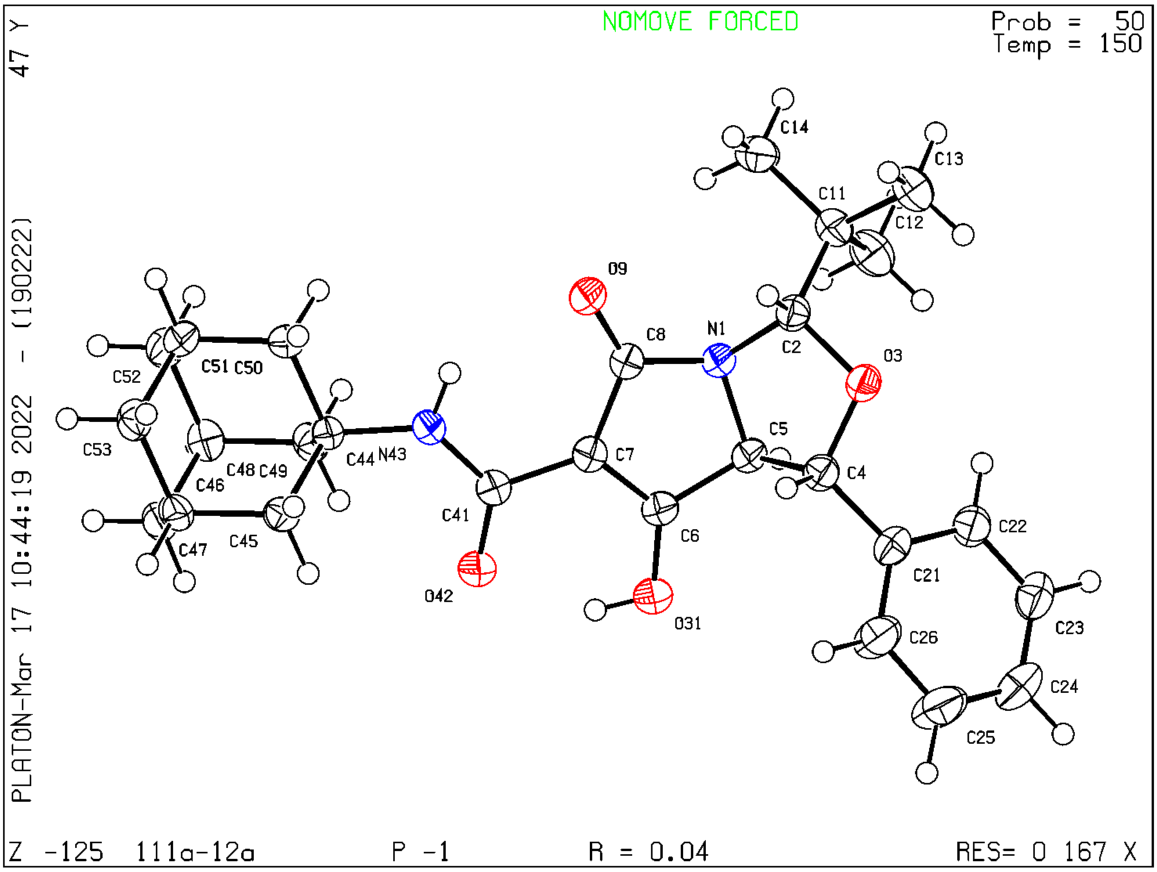

Thermal ellipsoid plot for structure **12a** with ellipsoid contour at 50% probability level.

Bond precision:	C-C = 0.0019 A		Wavelength=1.54184
Cell:	a=6.3963(2)	b=11.9497(4)	c=15.5243(5)
	alpha=88.428(3)	beta=81.238(2)	gamma=83.205(2)
Temperature:	150 K		

	Calculated	Reported
Volume	1164.44(7)	1164.44(7)
Space group	P -1	P -1
Hall group	-P 1	-P 1
Moiety formula	C27 H34 N2 O4	C27 H34 N2 O4
Sum formula	C27 H34 N2 O4	C27 H34 N2 O4
Mr	450.56	450.58
Dx,g cm-3	1.285	1.285
Z	2	2
Mu (mm-1)	0.689	0.689
F000	484.0	484.0
F000'	485.42	
h,k,lmax	8,15,19	8,14,19
Nref	4846	4807
Tmin,Tmax	0.855,0.902	0.780,0.900
Tmin'	0.825	

Correction method= # Reported T Limits: Tmin=0.780 Tmax=0.900
AbsCorr = MULTI-SCAN

Data completeness= 0.992 Theta(max)= 76.135

R(reflections)= 0.0389(4324) wR2(reflections)= 0.1058(4807)

S = 0.997 Npar= 298

Crystal parameters for structure **12a**

References

1) J. Cosier and A.M. Glazer, *J. Appl. Crystallogr.*, 1986, **19**, 105-107.
2) L. Palatinus and G. Chapuis, *J. Appl. Crystallogr.*, 2007, **40**, 786-790.
3) P. W. Betteridge, J.R. Carruthers, R.I. Cooper, K. Prout and D.J. Watkins, *J. Appl. Crystallogr.*, 2003, **36**, 1487-1487.
4) T. Shiraiwa, R. Saijoh, M. Suzuki, K. Yoshida, S. Nishimura and H. Nagasawa, *Chem. Pharm. Bull.*, 2003, **51**, 1363-1367.
5) Y. Singjunla, J. Baudoux and J. Rouden, *Org. Lett.*, 2013, **15**, 5770-5773.
6) P. Chaudhari and S. Bari, *Synth. Commun.*, 2015, **45**, 391-402.
7) C. Sparr and R. Gilmour, *Angew. Chem. Int. Ed.*, 2010, **49**, 6520-6523.
8) H. Hönig, P. Seufer-Wasserthal and H. Weber, *Tetrahedron*, 1990, **46**, 3841-3850.
9) S.G. Davies, A.M. Fletcher, A.B. Frost, J.A. Lee, P.M. Roberts and J.E. Thomson, *Tetrahedron*, 2013, **69**, 8885-8898.
10) M.D. Andrews, A.G. Brewster, K.M. Crapnell, A.J. Ibbett, T. Jones, M.G. Moloney, K. Prout and D. Watkin, *J. Chem. Soc. Perkin Trans. 1*, 1998, 223-235.
11) H. Bagum, K.E. Christensen, M. Genov, A. Pretsch, D. Pretsch and M.G. Moloney, *J. Org. Chem.*, 2019, **84**, 10257-10279.
12) T.D. Panduwawala, S. Iqbal, A.L. Thompson, M. Genov, A. Pretsch, D. Pretsch, S. Liu, R.H. Ebright, A. Howells, A. Maxwell and M.G. Moloney, *Org. Biomol. Chem.*, 2019, **17**, 5615-5632.
13) Y-C. Jeong, M. Anwar, Z. Bikadi, E. Hazai and M. G. Moloney, *Chem. Sci.*, 2013, **4**, 1008-1015.

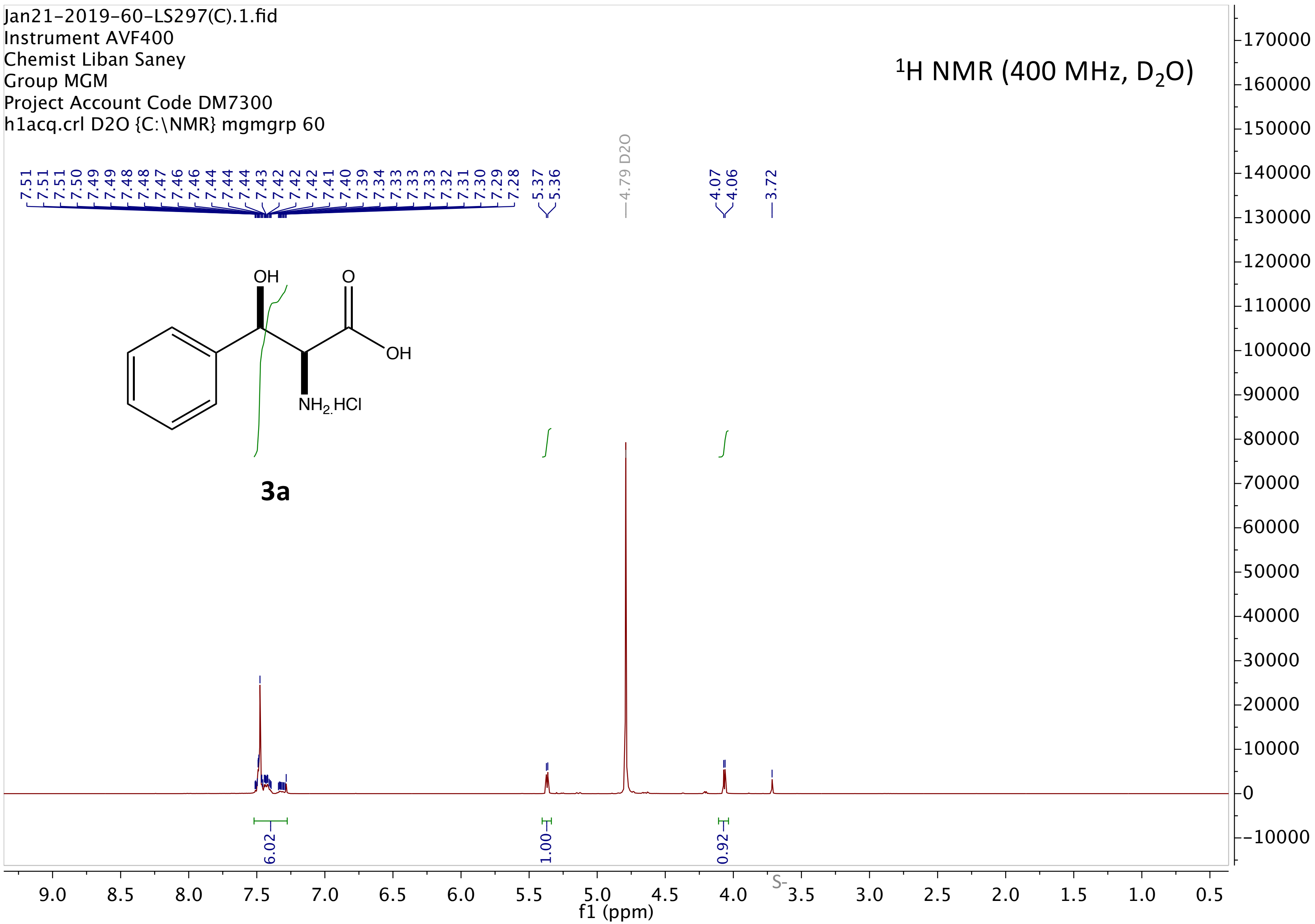
Jan21-2019-60-LS297(C).1.fid
Instrument AVF400
Chemist Liban Saney
Group MGM
Project Account Code DM7300
h1acq.crl D2O {C:\NMR} mgmgrp 60
¹H NMR (400 MHz, D₂O)
7.51 7.51 7.51 7.50 7.49 7.49 7.48 7.48 7.47 7.46 7.46 7.44 7.44 7.44 7.44 7.43 7.42 7.42 7.42 7.41 7.40 7.39 7.34 7.33 7.33 7.33 7.32 7.31 7.30 7.29 7.28
5.37 5.36
4.79 D2O
4.07 4.06
3.72
OH
O
OH
NH2.HCl
3a
6.02
1.00
0.92
f1 (ppm)

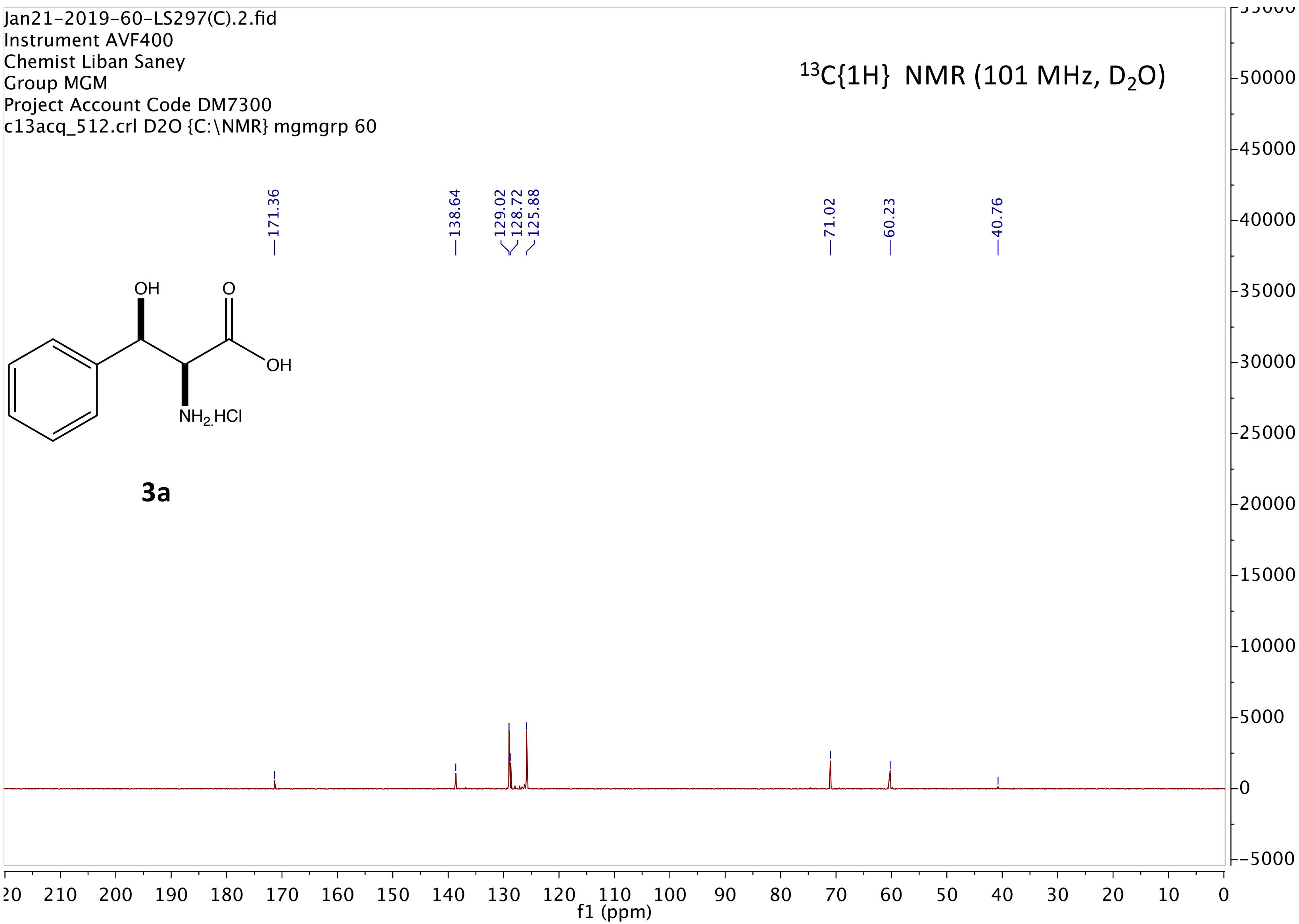
Jan21-2019-60-LS297(C).2.fid
Instrument AVF400
Chemist Liban Saney
Group MGM
Project Account Code DM7300
c13acq_512.crl D2O {C:\NMR} mgmgrp 60
^{13}C{1H} NMR (101 MHz, D_2O)
171.36
138.64
129.02
128.72
125.88
71.02
60.23
40.76
OH
O
OH
NH2.HCl
3a
f1 (ppm)

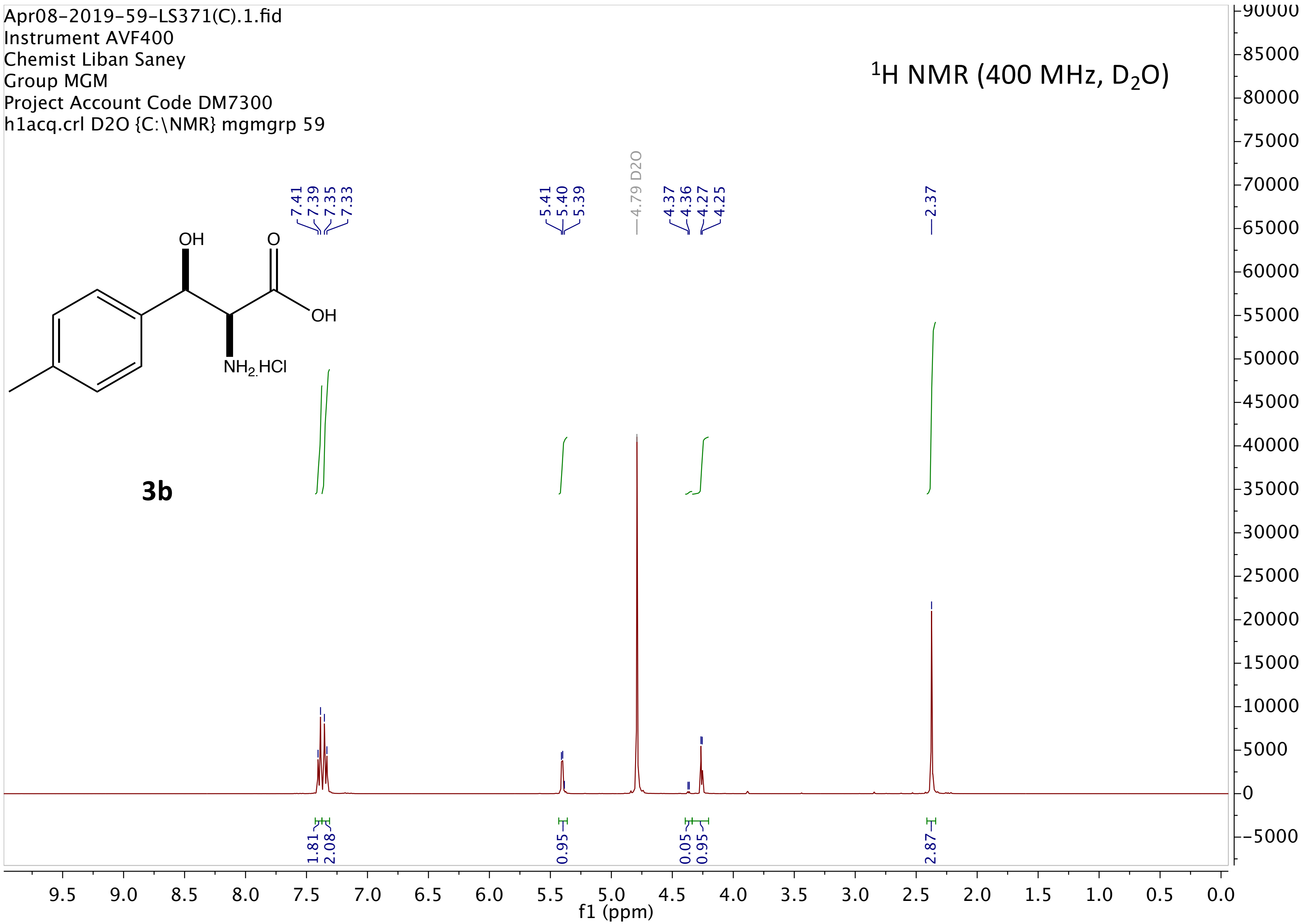

Apr08-2019-59-LS371(C).1.fid
Instrument AVF400
Chemist Liban Saney
Group MGM
Project Account Code DM7300
h1acq.crl D2O {C:\NMR} mgmgrp 59
1H NMR (400 MHz, D2O)
OH
O
OH
NH2.HCl
3b
7.41
7.39
7.35
7.33
5.41
5.40
5.39
4.79 D2O
4.37
4.36
4.27
4.25
2.37
1.81
2.08
0.95
0.05
0.95
2.87
f1 (ppm)

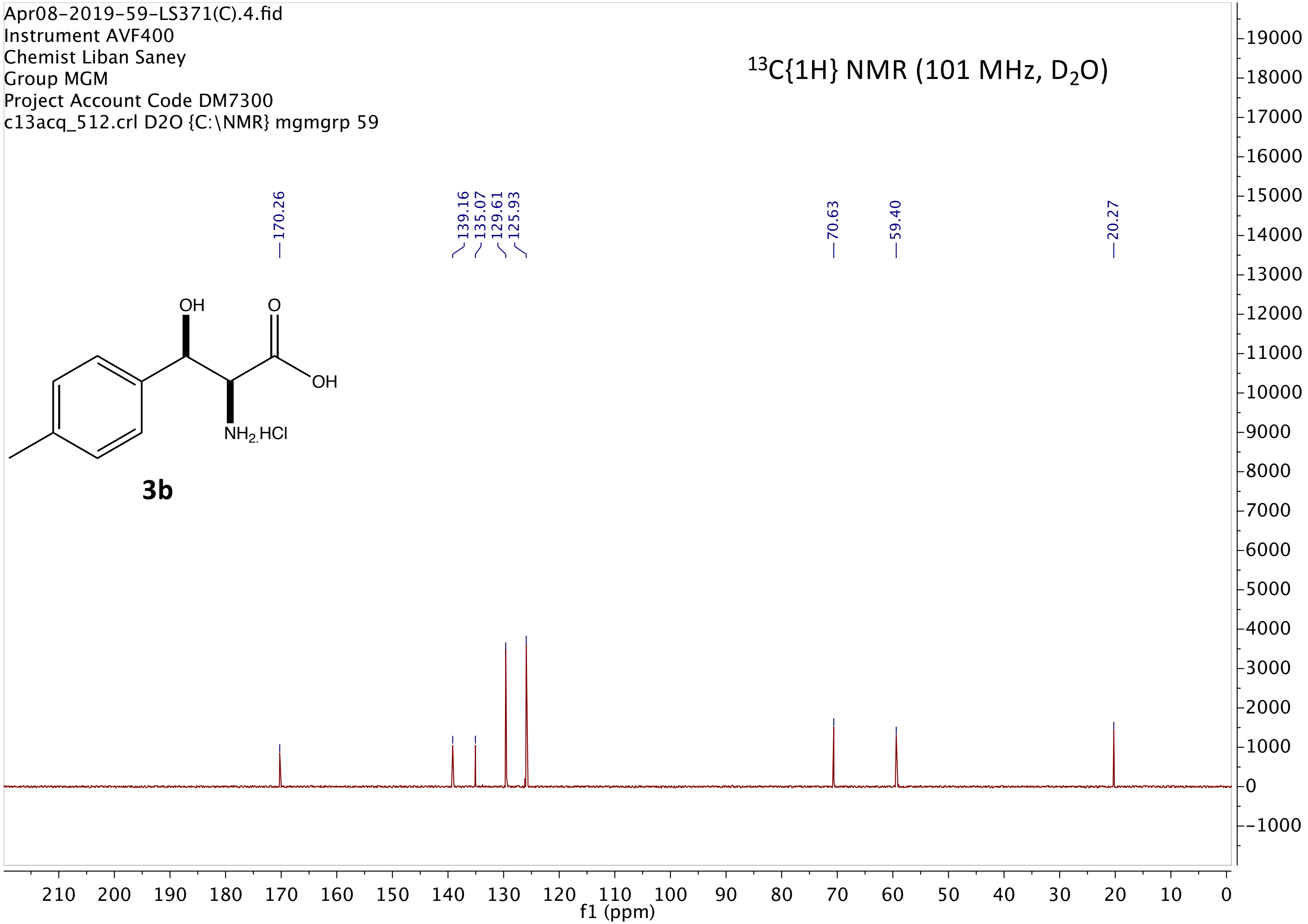
Apr08-2019-59-LS371(C).4.fid
Instrument AVF400
Chemist Liban Saney
Group MGM
Project Account Code DM7300
c13acq_512.crl D2O {C:\NMR} mgmgrp 59
$^{13}C\{1H\}$ NMR (101 MHz, D_2O)
170.26
139.16
135.07
129.61
125.93
70.63
59.40
20.27
OH
O
OH
NH2.HCl
3b
f1 (ppm)

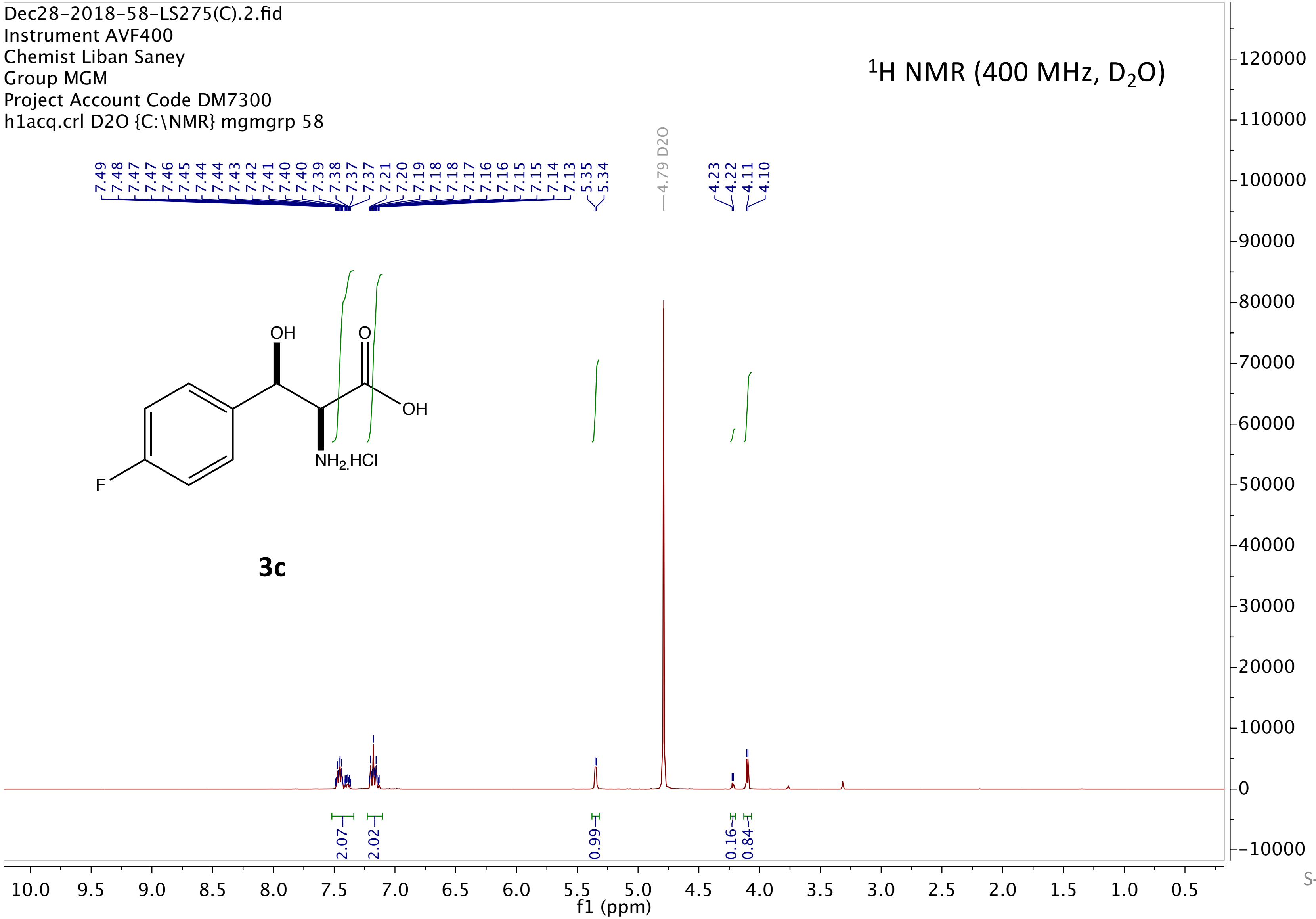

Dec28-2018-58-LS275(C).2.fid
Instrument AVF400
Chemist Liban Saney
Group MGM
Project Account Code DM7300
h1acq.crl D2O {C:\NMR} mgmgrp 58
^{1}H NMR (400 MHz, D_2O)
7.49 7.48 7.47 7.47 7.46 7.45 7.44 7.44 7.43 7.42 7.41 7.40 7.40 7.39 7.38 7.37 7.37 7.21 7.20 7.19 7.18 7.18 7.17 7.16 7.16 7.15 7.15 7.14 7.13 5.35 5.34
4.79 D2O
4.23 4.22 4.11 4.10
OH
O
OH
NH_2.HCl
F
2.07
2.02
0.99
0.16
0.84
f1 (ppm)

3c

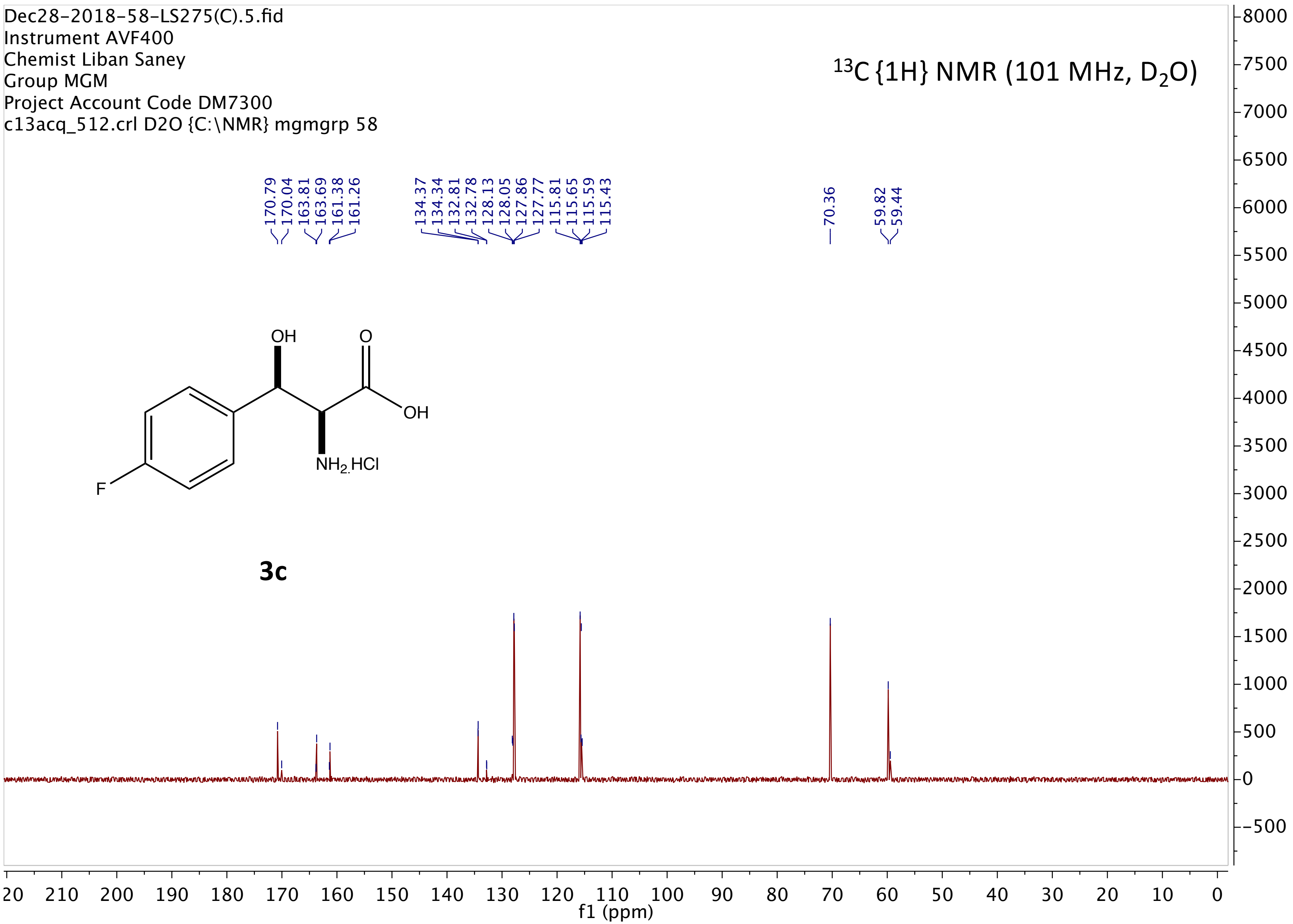
Dec28-2018-58-LS275(C).5.fid
Instrument AVF400
Chemist Liban Saney
Group MGM
Project Account Code DM7300
c13acq_512.crl D2O {C:\NMR} mgmgrp 58
$^{13}C\{1H\}$ NMR (101 MHz, D_2O)
170.79
170.04
163.81
163.69
161.38
161.26
134.37
134.34
132.81
132.78
128.13
128.05
127.86
127.77
115.81
115.65
115.59
115.43
70.36
59.82
59.44
OH
O
OH
F
NH2.HCl
3c
f1 (ppm)

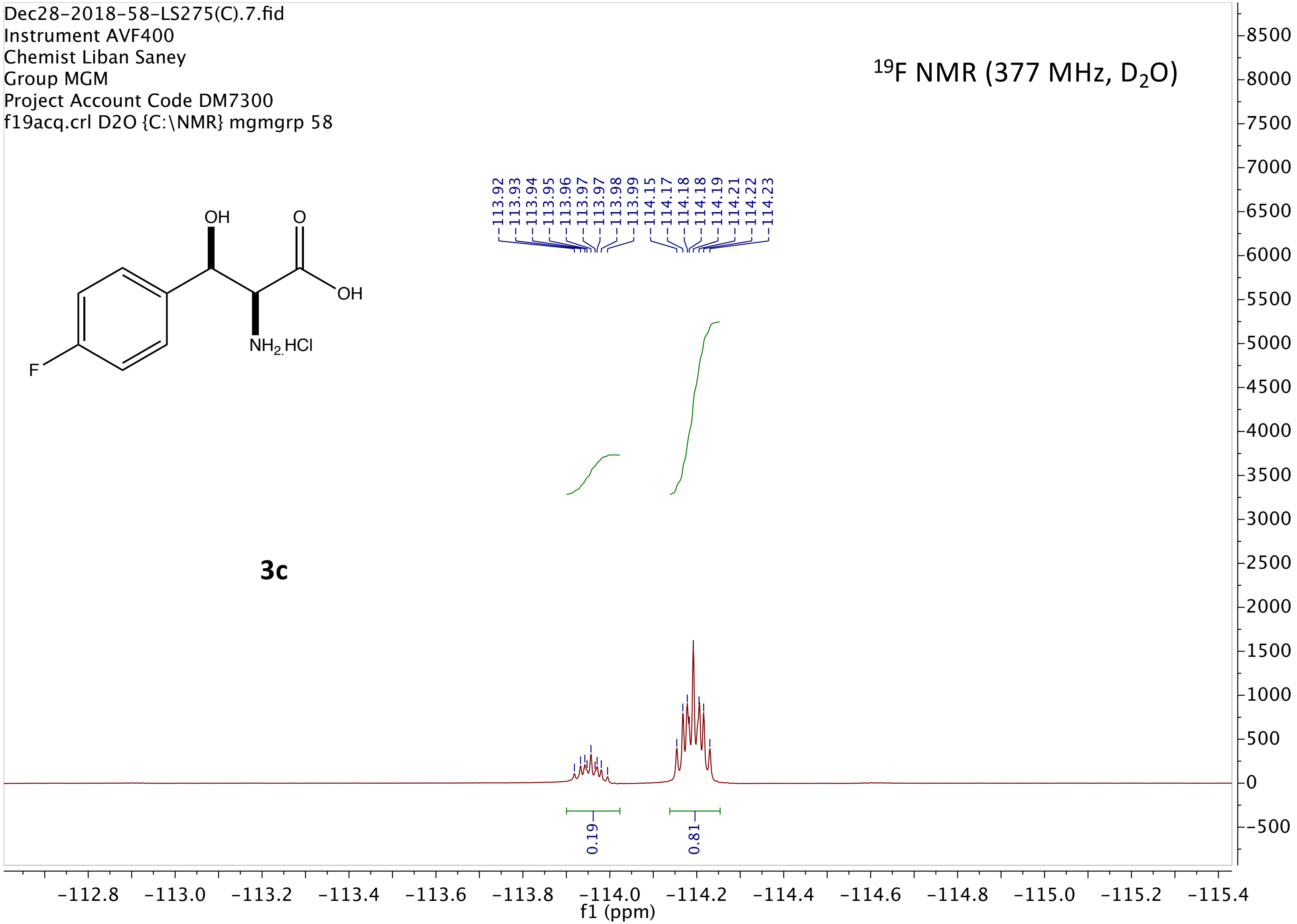
Dec28-2018-58-LS275(C).7.fid
Instrument AVF400
Chemist Liban Saney
Group MGM
Project Account Code DM7300
f19acq.crl D2O {C:\NMR} mgmgrp 58
19F NMR (377 MHz, D2O)
OH
O
OH
F
NH2.HCl
3c
-113.92
-113.93
-113.94
-113.95
-113.96
-113.97
-113.97
-113.98
-113.99
-114.15
-114.17
-114.18
-114.18
-114.19
-114.21
-114.22
-114.23
0.19
0.81
f1 (ppm)

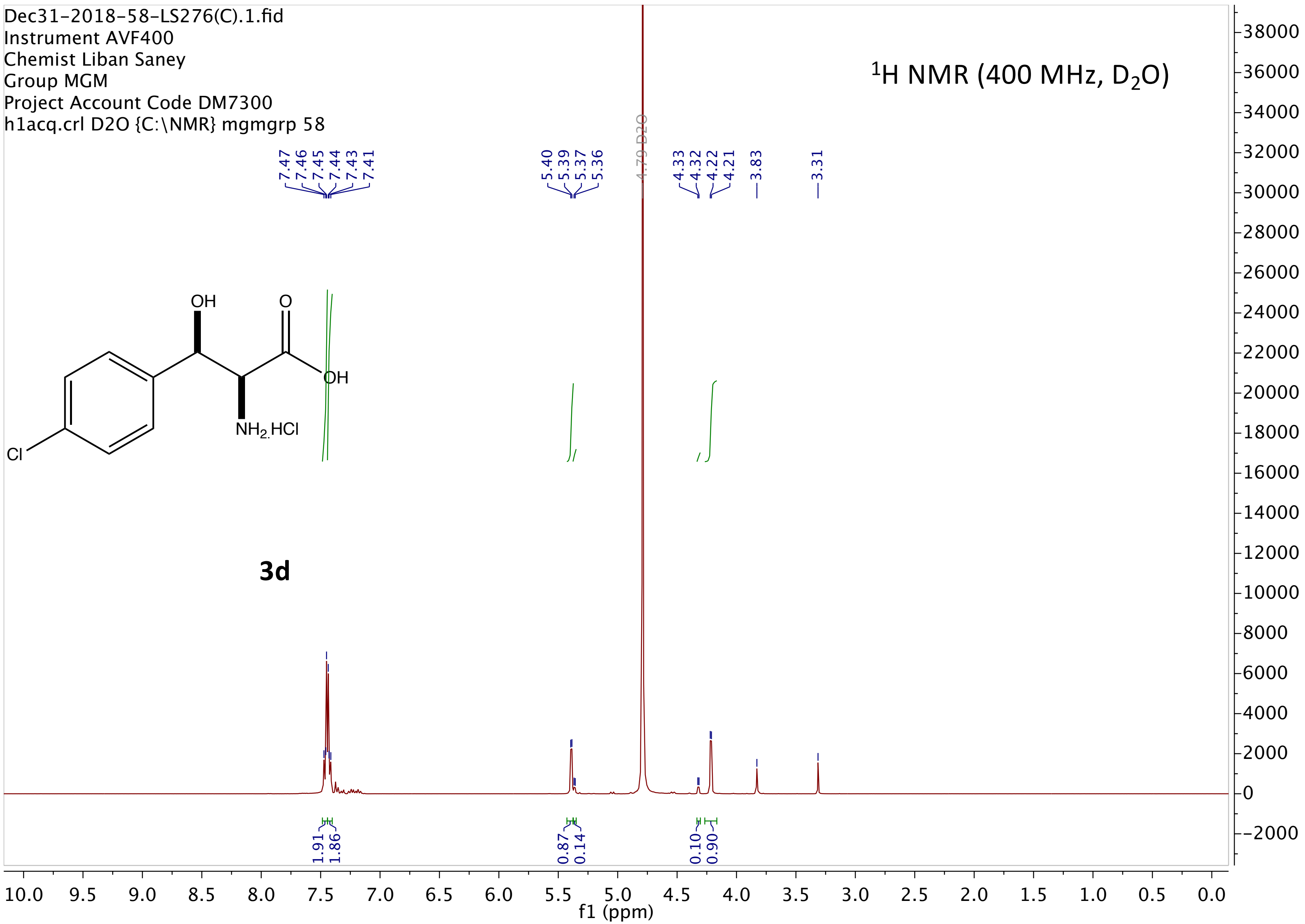

Dec31-2018-58-LS276(C).1.fid
Instrument AVF400
Chemist Liban Saney
Group MGM
Project Account Code DM7300
h1acq.crl D2O {C:\NMR} mgmgrp 58
¹H NMR (400 MHz, D₂O)
7.47 7.46 7.45 7.44 7.43 7.41
5.40 5.39 5.37 5.36
4.79 D2O
4.33 4.32 4.22 4.21
3.83
3.31
OH
O
OH
NH2.HCl
Cl
3d
1.91 1.86
0.87 0.14
0.10 0.90
f1 (ppm)

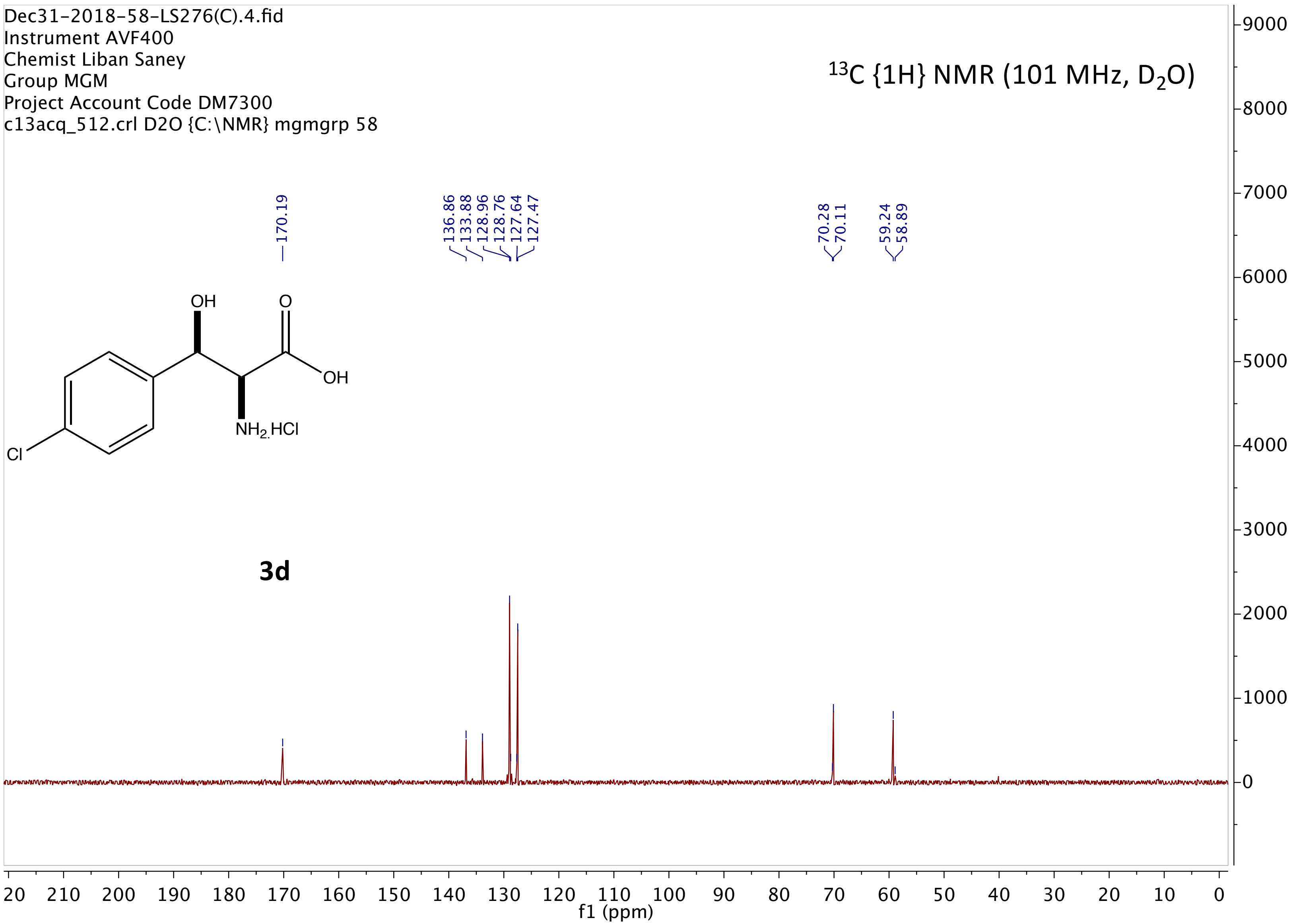
Dec31-2018-58-LS276(C).4.fid
Instrument AVF400
Chemist Liban Saney
Group MGM
Project Account Code DM7300
c13acq_512.crl D2O {C:\NMR} mgmgrp 58
^{13}C {1H} NMR (101 MHz, D_2O)
170.19
136.86
133.88
128.96
128.76
127.64
127.47
70.28
70.11
59.24
58.89
OH
O
OH
NH_2.HCl
Cl
3d
f1 (ppm)

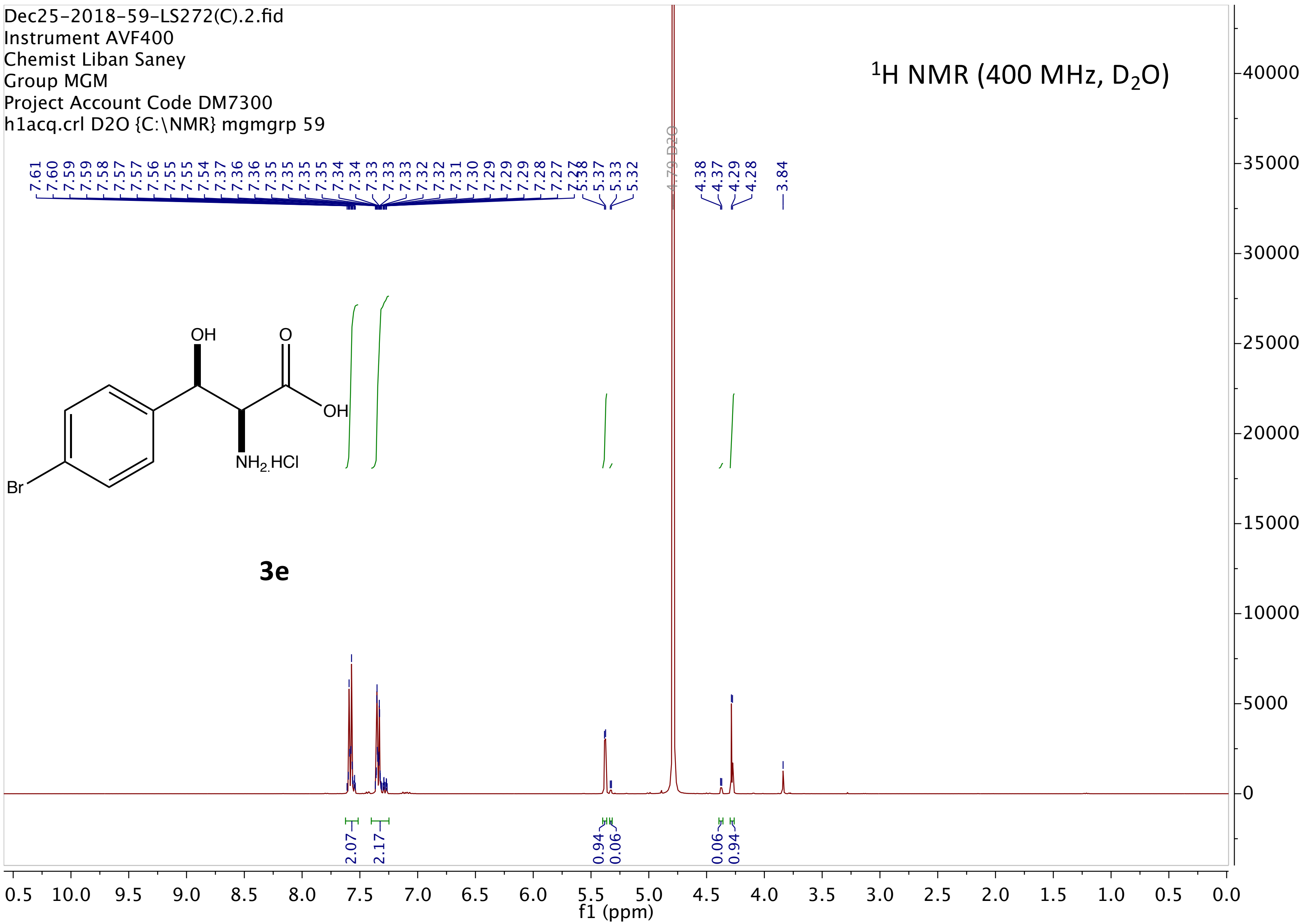
Dec25-2018-59-LS272(C).2.fid
Instrument AVF400
Chemist Liban Saney
Group MGM
Project Account Code DM7300
h1acq.crl D2O {C:\NMR} mgmgrp 59
1H NMR (400 MHz, D2O)
7.61 7.60 7.59 7.59 7.58 7.57 7.57 7.56 7.55 7.55 7.54 7.37 7.36 7.36 7.35 7.35 7.35 7.35 7.34 7.34 7.33 7.33 7.33 7.32 7.32 7.31 7.30 7.29 7.29 7.29 7.28 7.27 7.27
5.38 5.37 5.33 5.32
4.79 D2O
4.38 4.37 4.29 4.28
3.84
OH
O
OH
NH2.HCl
Br
3e
2.07
2.17
0.94
0.06
0.06
0.94
f1 (ppm)

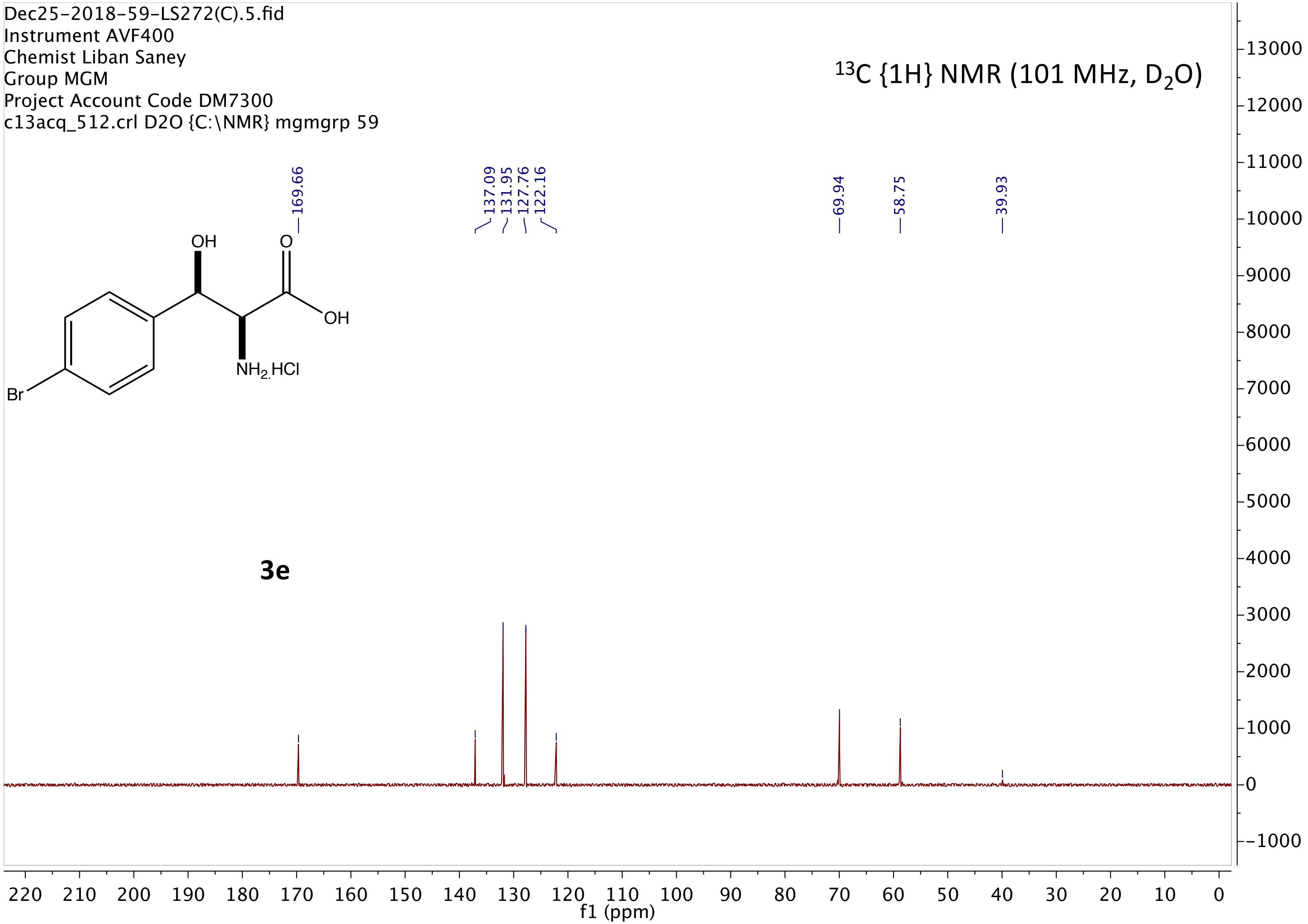
Dec25-2018-59-LS272(C).5.fid
Instrument AVF400
Chemist Liban Saney
Group MGM
Project Account Code DM7300
c13acq_512.crl D2O {C:\NMR} mgmgrp 59
13C {1H} NMR (101 MHz, D2O)
169.66
137.09
131.95
127.76
122.16
69.94
58.75
39.93
OH
O
OH
NH2.HCl
Br
3e
f1 (ppm)

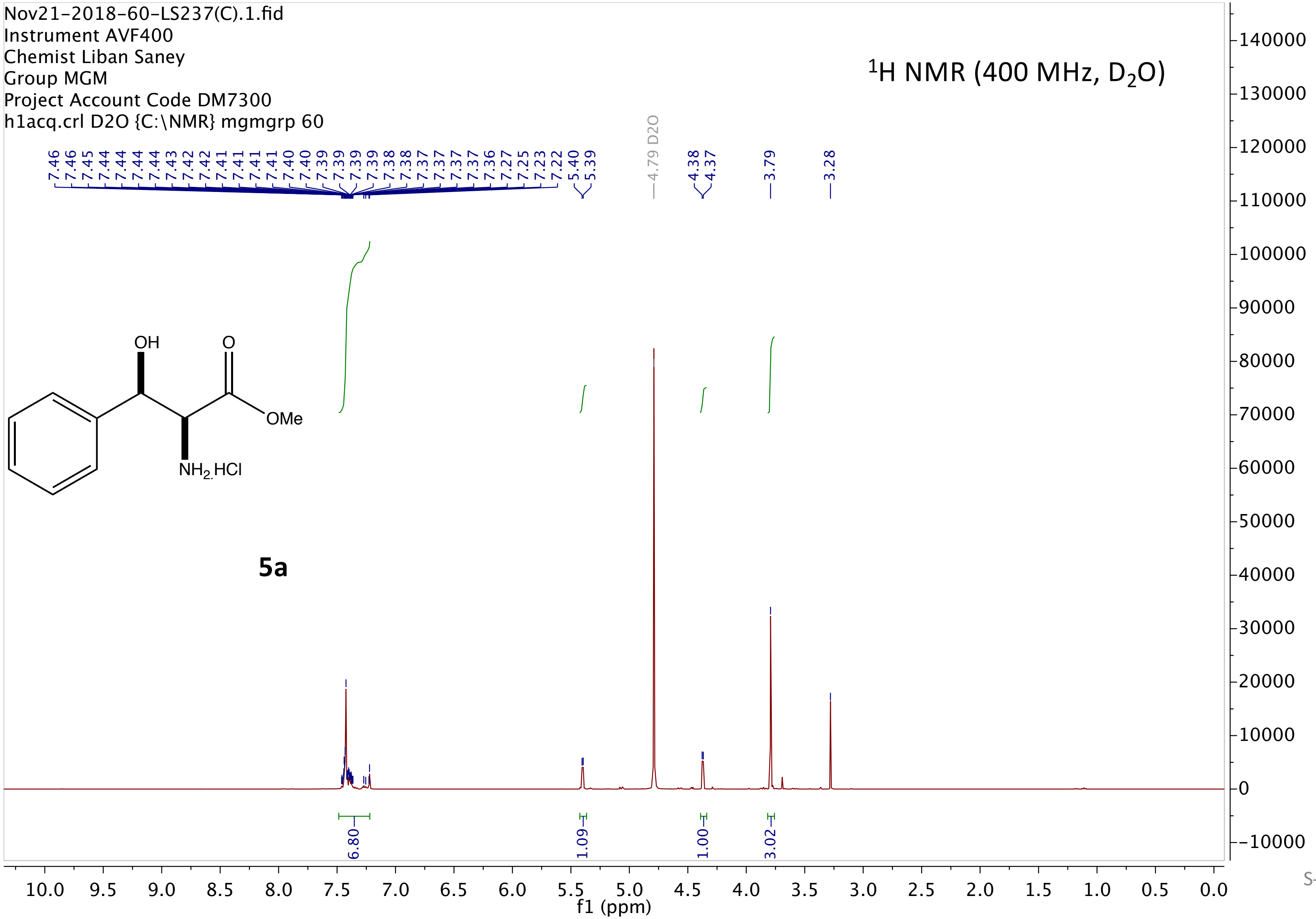

Nov21-2018-60-LS237(C).1.fid
Instrument AVF400
Chemist Liban Saney
Group MGM
Project Account Code DM7300
h1acq.crl D2O {C:\NMR} mgmgrp 60
^{1}H NMR (400 MHz, D_2O)
7.46 7.46 7.45 7.44 7.44 7.44 7.44 7.43 7.42 7.42 7.41 7.41 7.41 7.41 7.40 7.40 7.39 7.39 7.39 7.39 7.38 7.38 7.37 7.37 7.37 7.37 7.36 7.27 7.25 7.23 7.22
5.40 5.39
4.79 D2O
4.38 4.37
3.79
3.28
OH
O
OMe
NH2.HCl
5a
6.80
1.09
1.00
3.02
f1 (ppm)

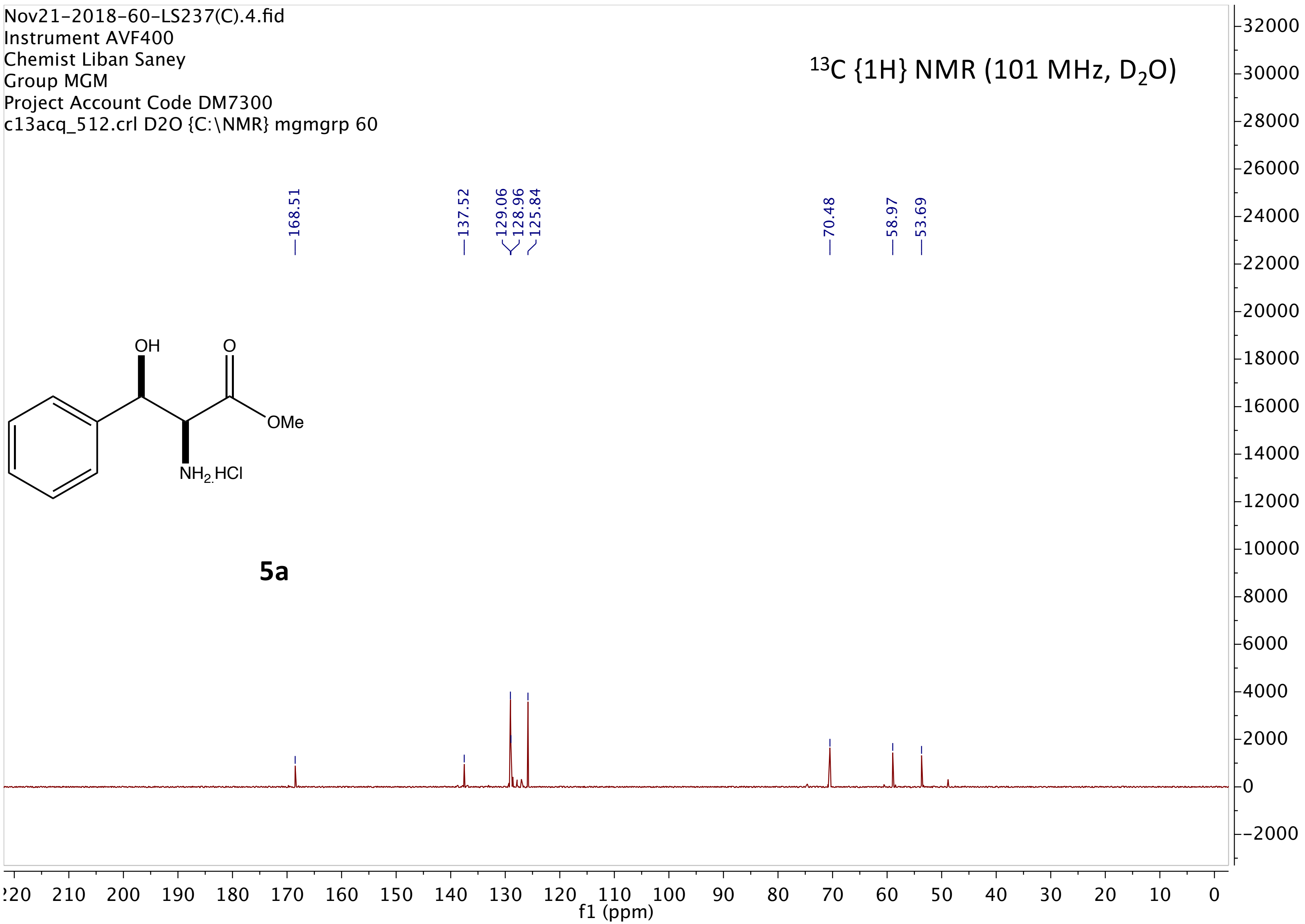
Nov21-2018-60-LS237(C).4.fid
Instrument AVF400
Chemist Liban Saney
Group MGM
Project Account Code DM7300
c13acq_512.crl D2O {C:\NMR} mgmgrp 60
13C {1H} NMR (101 MHz, D2O)
168.51
137.52
129.06
128.96
125.84
70.48
58.97
53.69
OH
O
OMe
NH2.HCl
5a
f1 (ppm)

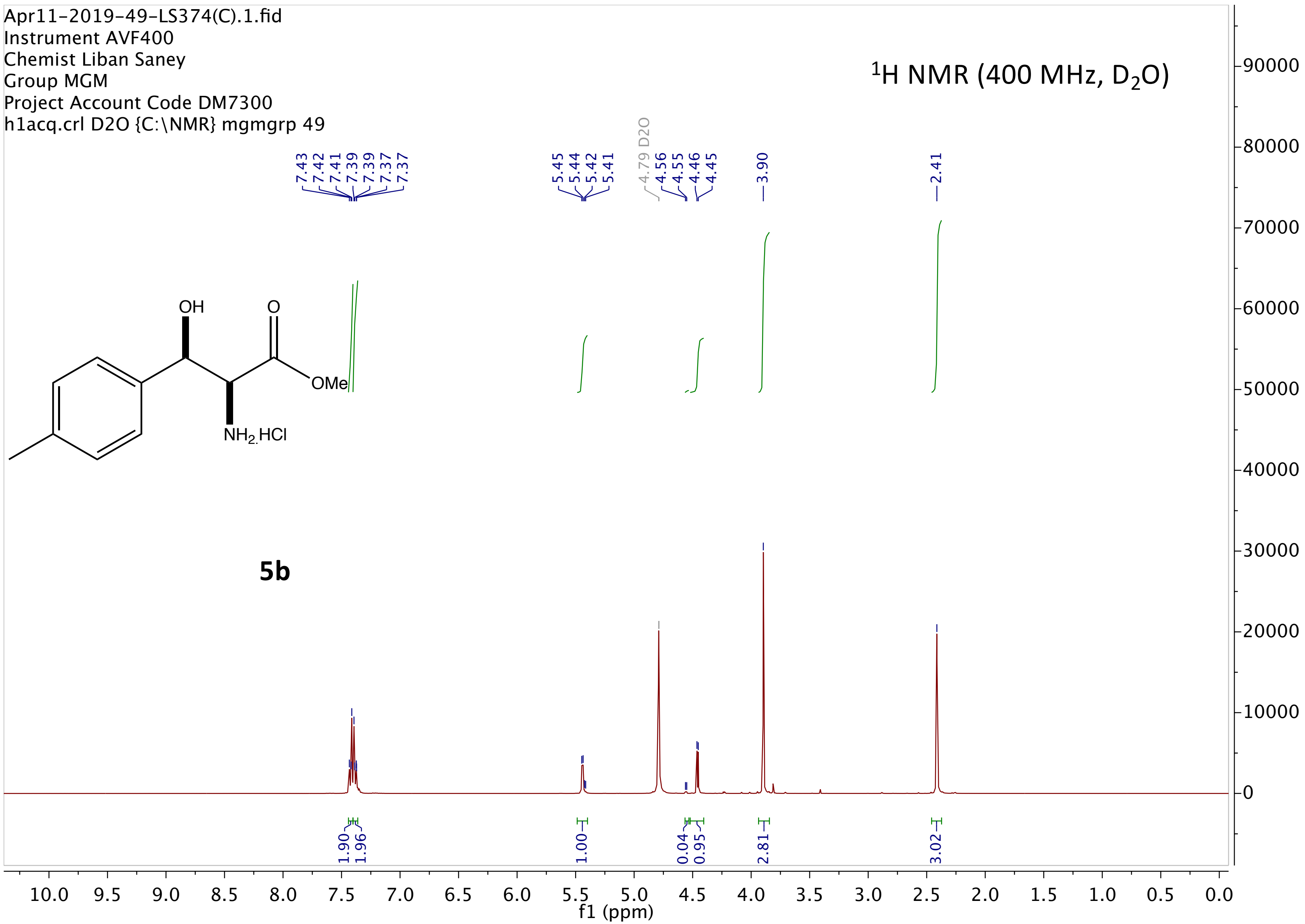
Apr11-2019-49-LS374(C).1.fid
Instrument AVF400
Chemist Liban Saney
Group MGM
Project Account Code DM7300
h1acq.crl D2O {C:\NMR} mgmgrp 49
1H NMR (400 MHz, D2O)
OH
O
OMe
NH2.HCl
5b
7.43
7.42
7.41
7.39
7.39
7.37
7.37
5.45
5.44
5.42
5.41
4.79 D2O
4.56
4.55
4.46
4.45
3.90
2.41
1.90
1.96
1.00
0.04
0.95
2.81
3.02
f1 (ppm)

Apr11-2019-49-LS374(C).4.fid
Instrument AVF400
Chemist Liban Saney
Group MGM
Project Account Code DM7300
c13acq_512.crl D2O {C:\NMR} mgmgrp 49

^{13}C {1H} NMR (101 MHz, D_2O)

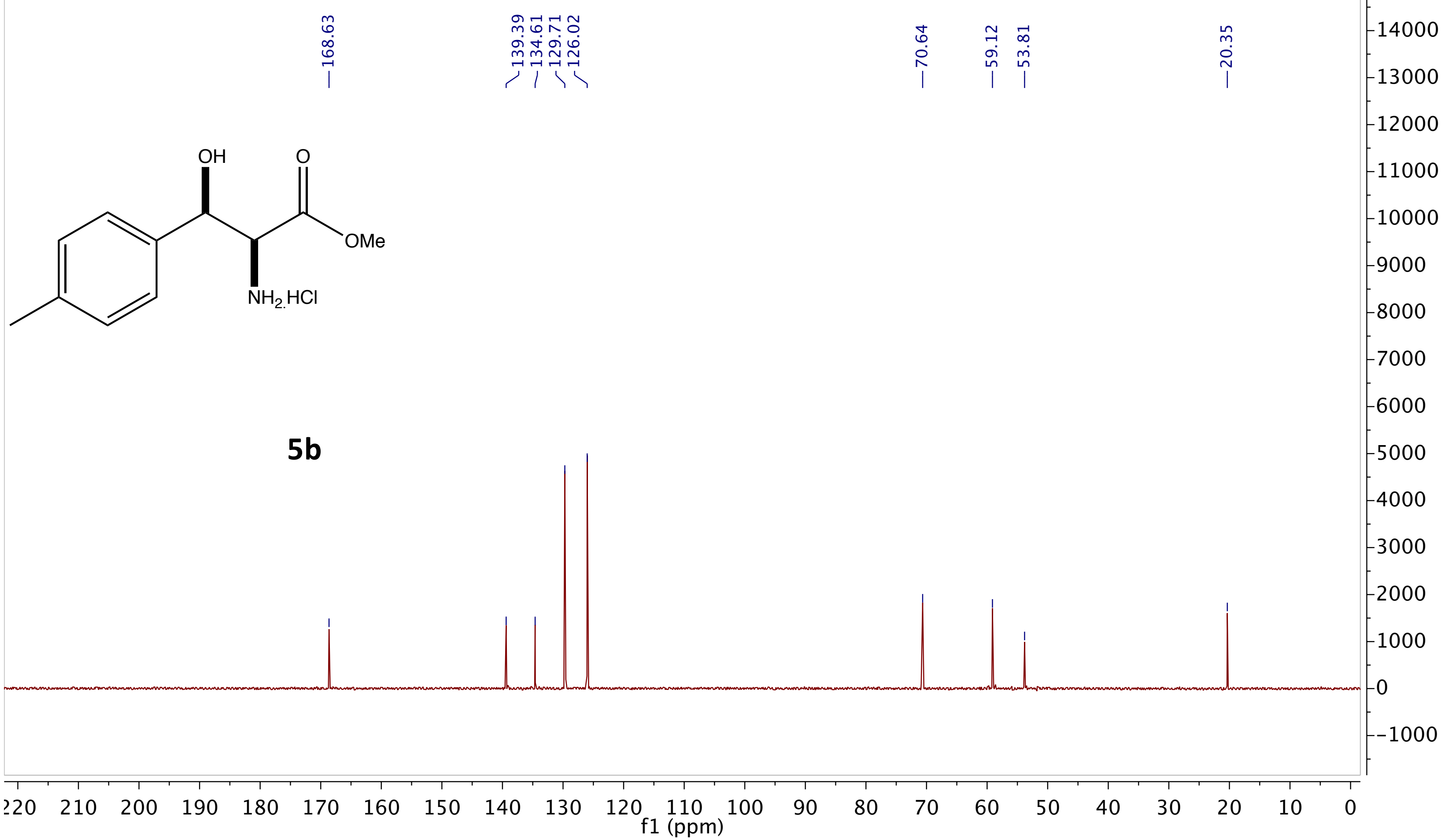

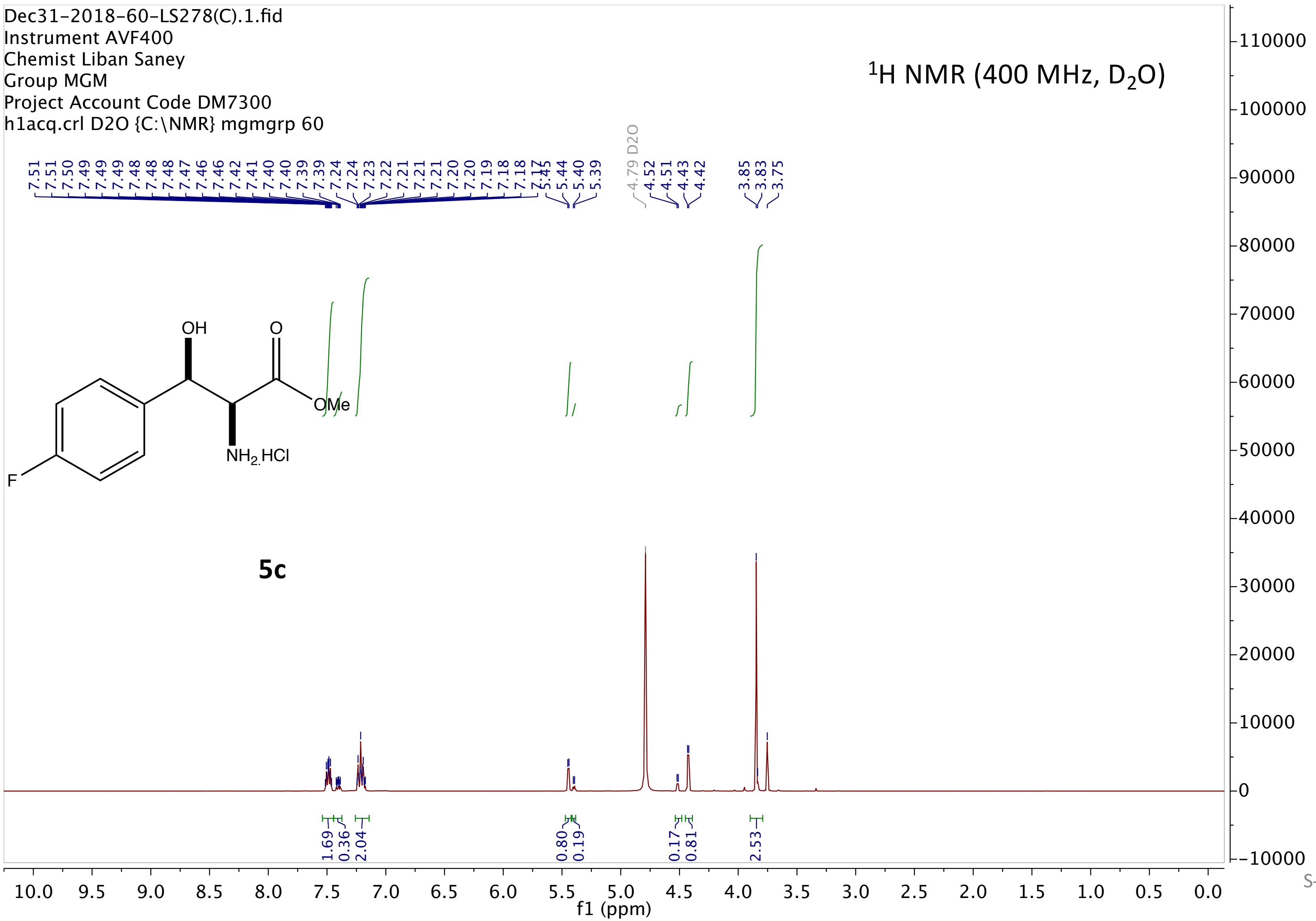
Dec31-2018-60-LS278(C).1.fid
Instrument AVF400
Chemist Liban Saney
Group MGM
Project Account Code DM7300
h1acq.crl D2O {C:\NMR} mgmgrp 60
^{1}H NMR (400 MHz, D_2O)
7.51 7.51 7.50 7.49 7.49 7.49 7.48 7.48 7.48 7.47 7.46 7.46 7.42 7.41 7.40 7.40 7.39 7.39 7.24 7.24 7.23 7.22 7.21 7.21 7.21 7.20 7.20 7.19 7.18 7.18 7.17 5.45 5.44 5.40 5.39
4.79 D2O
4.52 4.51 4.43 4.42
3.85 3.83 3.75
OH
O
OMe
F
NH2.HCl
5c
1.69 0.36 2.04 0.80 0.19 0.17 0.81 2.53
f1 (ppm)

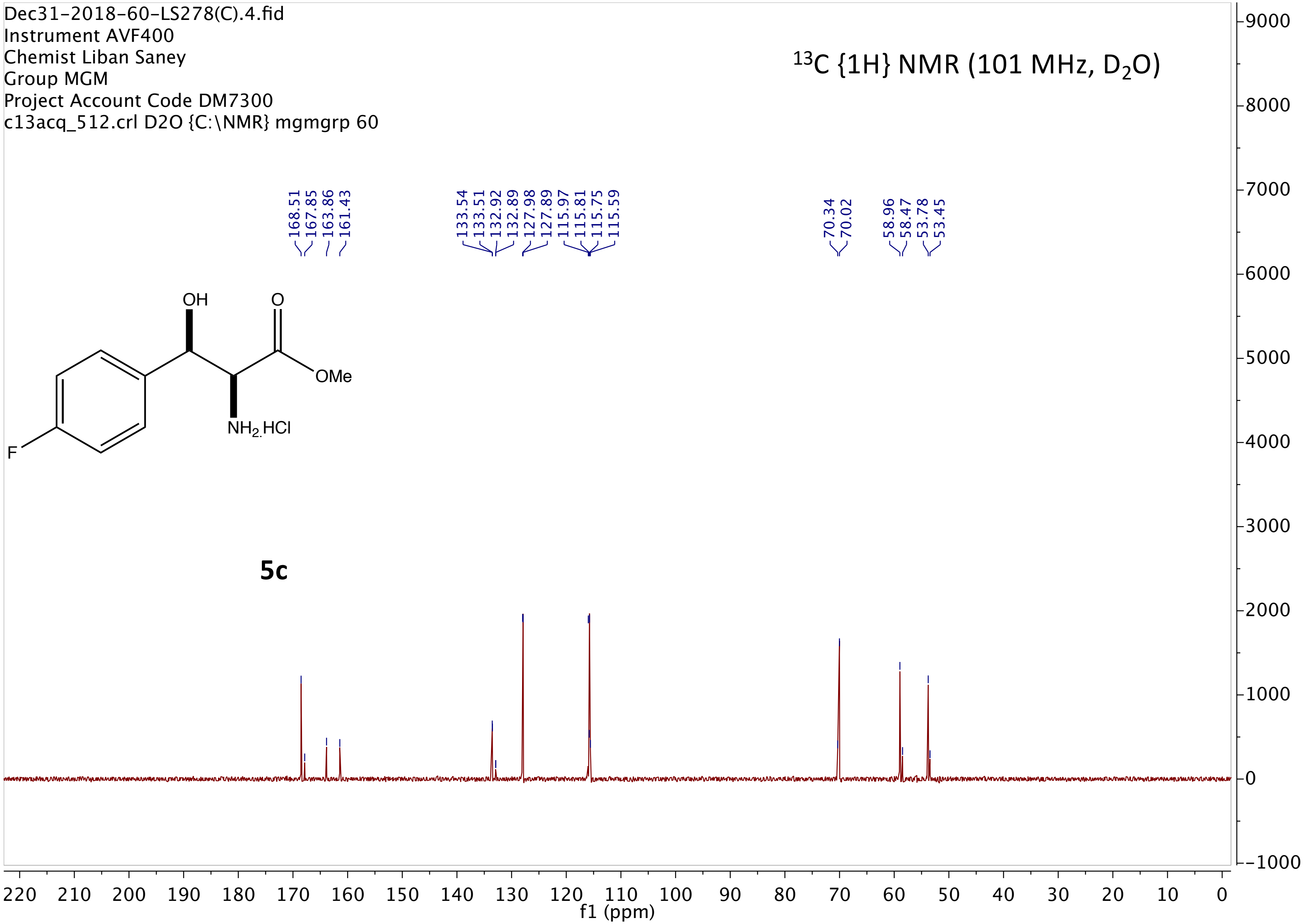
Dec31-2018-60-LS278(C).4.fid
Instrument AVF400
Chemist Liban Saney
Group MGM
Project Account Code DM7300
c13acq_512.crl D2O {C:\NMR} mgmgrp 60
^{13}C {1H} NMR (101 MHz, D_2O)
168.51
167.85
163.86
161.43
133.54
133.51
132.92
132.89
127.98
127.89
115.97
115.81
115.75
115.59
70.34
70.02
58.96
58.47
53.78
53.45
OH
O
OMe
NH2.HCl
F
5c
f1 (ppm)

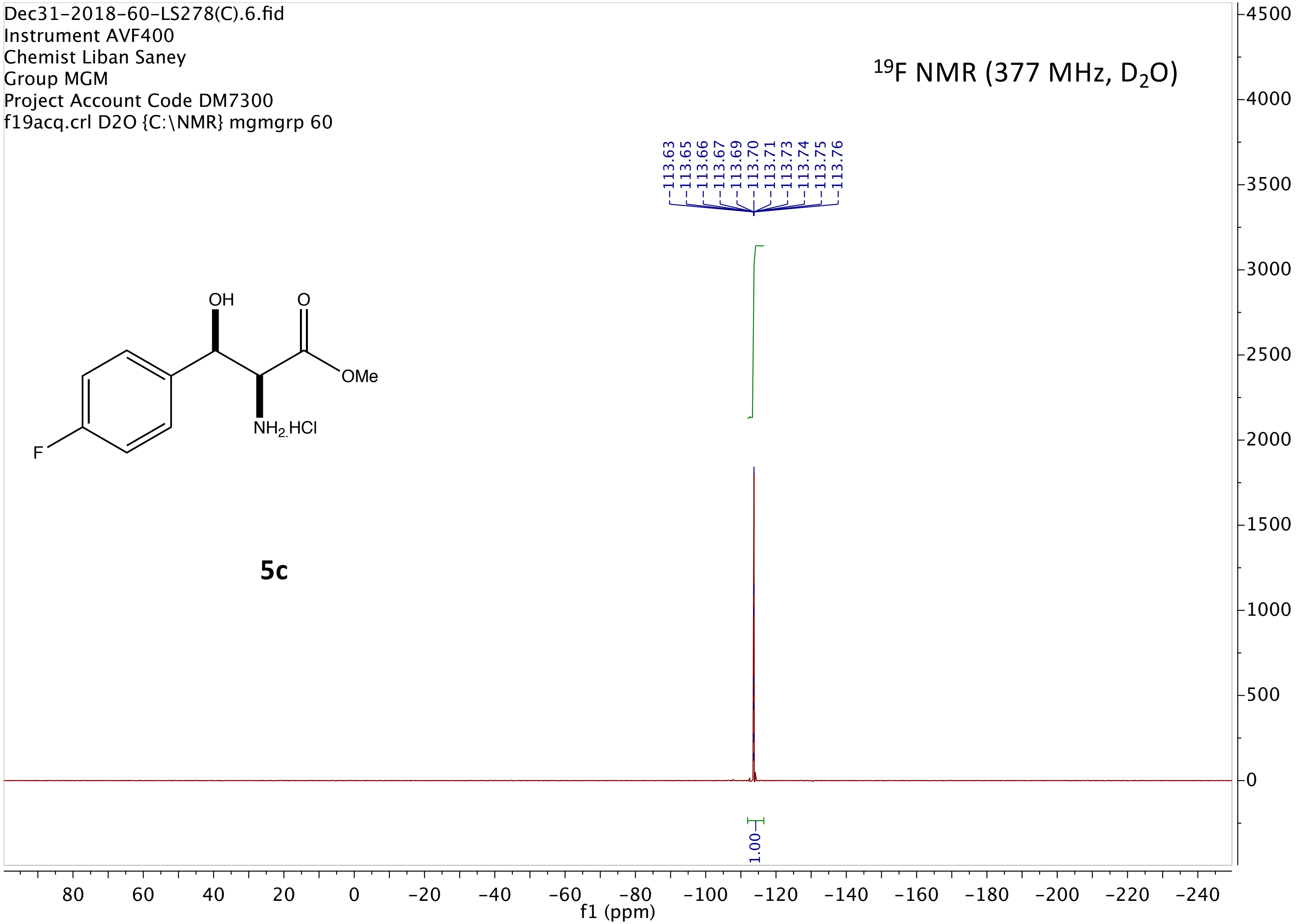
Dec31-2018-60-LS278(C).6.fid
Instrument AVF400
Chemist Liban Saney
Group MGM
Project Account Code DM7300
f19acq.crl D2O {C:\NMR} mgmgrp 60
^{19}F NMR (377 MHz, D_2O)
−113.63
−113.65
−113.66
−113.67
−113.69
−113.70
−113.71
−113.73
−113.74
−113.75
−113.76
OH
O
OMe
F
NH2.HCl
5c
1.00
4500
4000
3500
3000
2500
2000
1500
1000
500
0
80 60 40 20 0 −20 −40 −60 −80 −100 −120 −140 −160 −180 −200 −220 −240
f1 (ppm)

Dec31-2018-59-LS279(C).1.fid
Instrument AVF400
Chemist Liban Saney
Group MGM
Project Account Code DM7300
h1acq.crl D2O {C:\NMR} mgmgrp 59

^1H NMR (400 MHz, D_2O)

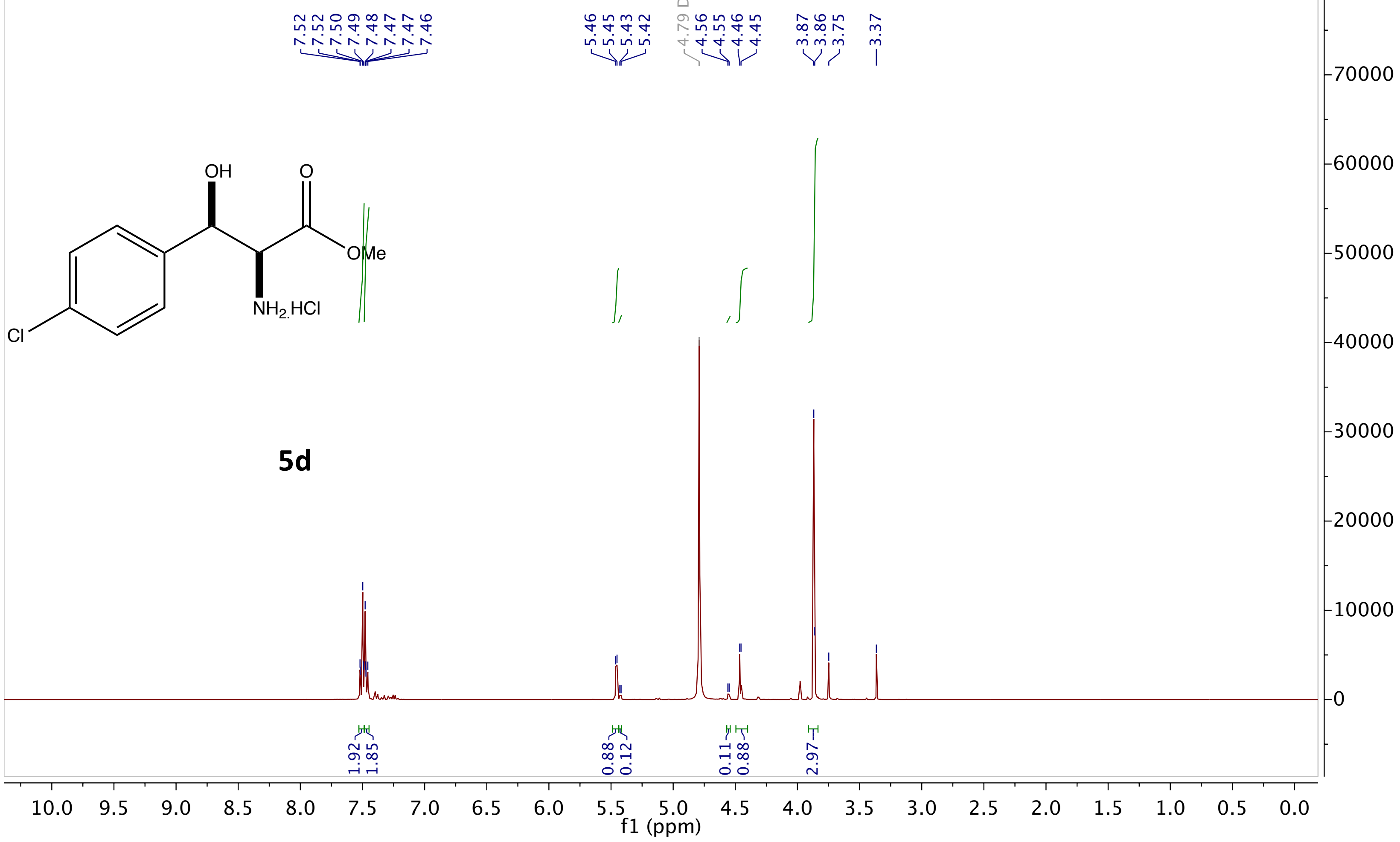

5d

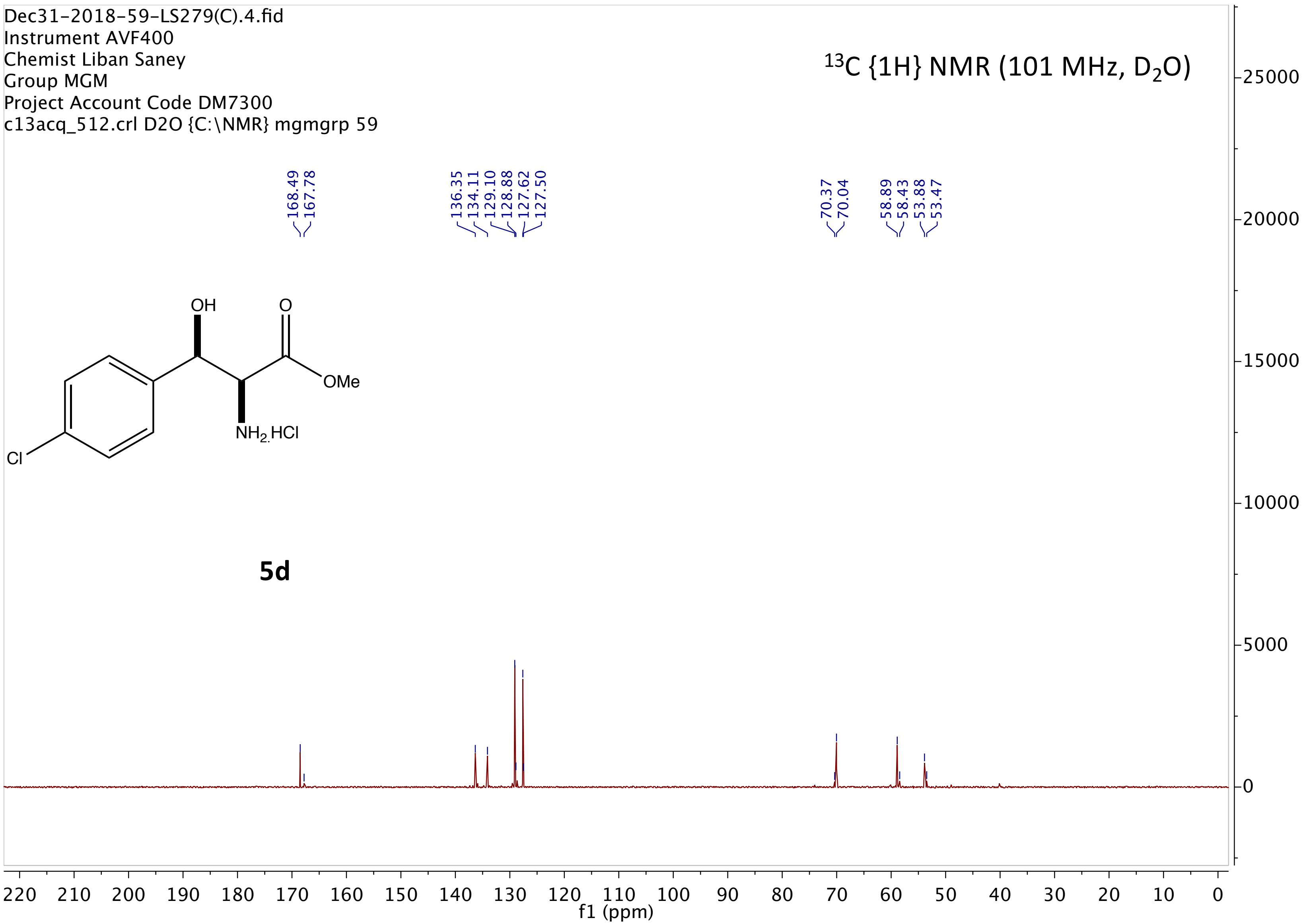
Dec31-2018-59-LS279(C).4.fid
Instrument AVF400
Chemist Liban Saney
Group MGM
Project Account Code DM7300
c13acq_512.crl D2O {C:\NMR} mgmgrp 59
13C {1H} NMR (101 MHz, D2O)
168.49
167.78
136.35
134.11
129.10
128.88
127.62
127.50
70.37
70.04
58.89
58.43
53.88
53.47
OH
O
OMe
NH2.HCl
Cl
5d
25000
20000
15000
10000
5000
0
220 210 200 190 180 170 160 150 140 130 120 110 100 90 80 70 60 50 40 30 20 10 0
f1 (ppm)

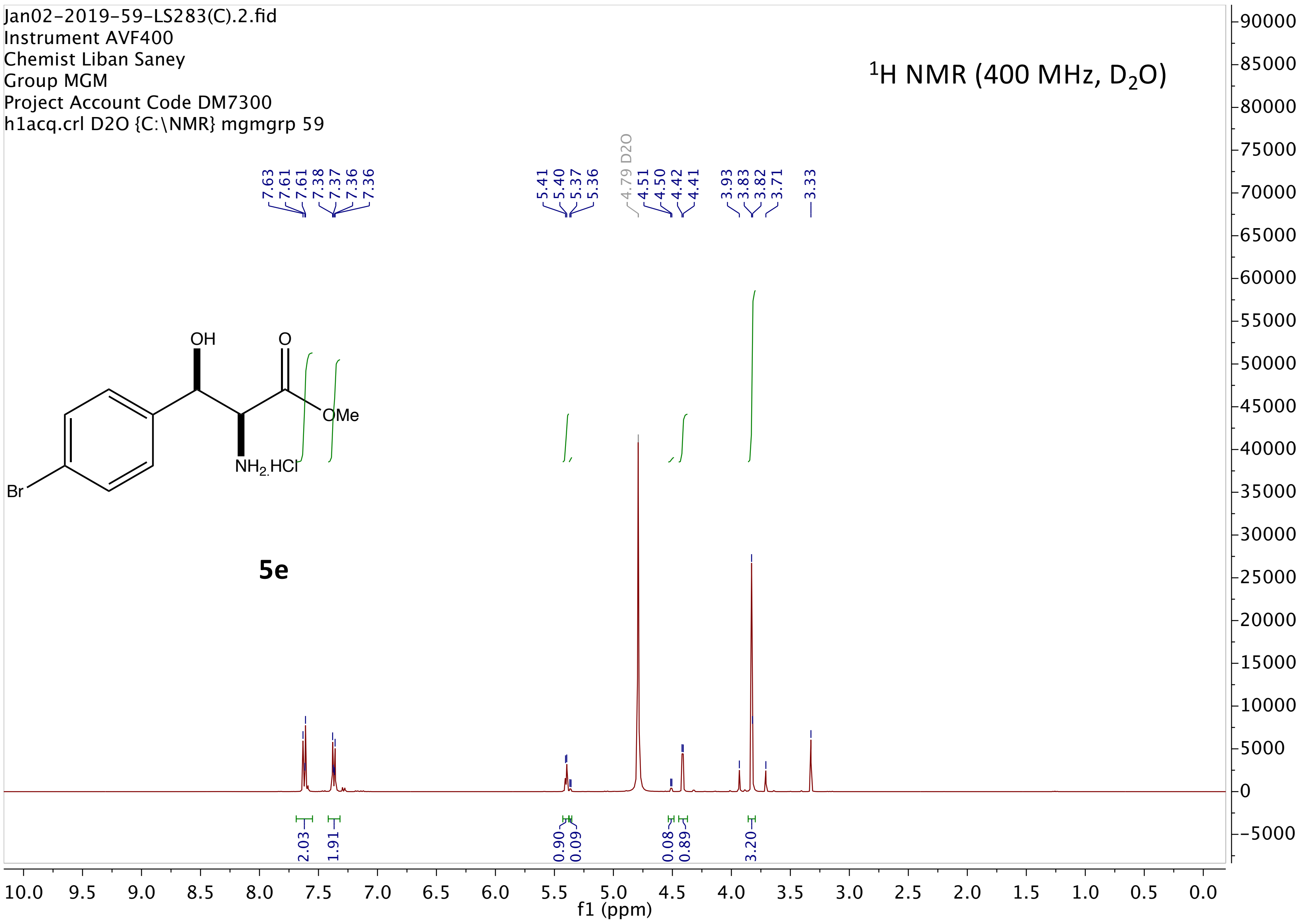

Jan02-2019-59-LS283(C).2.fid
Instrument AVF400
Chemist Liban Saney
Group MGM
Project Account Code DM7300
h1acq.crl D2O {C:\NMR} mgmgrp 59
1H NMR (400 MHz, D2O)
7.63 7.61 7.61 7.38 7.37 7.36 7.36
5.41 5.40 5.37 5.36
4.79 D2O
4.51 4.50 4.42 4.41
3.93 3.83 3.82 3.71
3.33
OH
O
OMe
Br
NH2.HCl
5e
2.03 1.91 0.90 0.09 0.08 0.89 3.20
f1 (ppm)

Jan02-2019-59-LS283(C).5.fid
Instrument AVF400
Chemist Liban Saney
Group MGM
Project Account Code DM7300
c13acq_512.crl D2O {C:\NMR} mgmgrp 59

^{13}C {1H} NMR (101 MHz, D_2O)

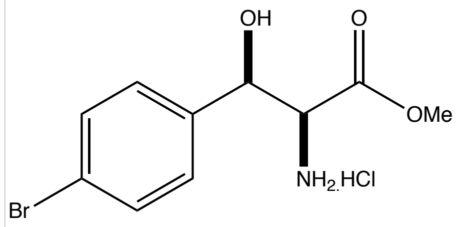

5e

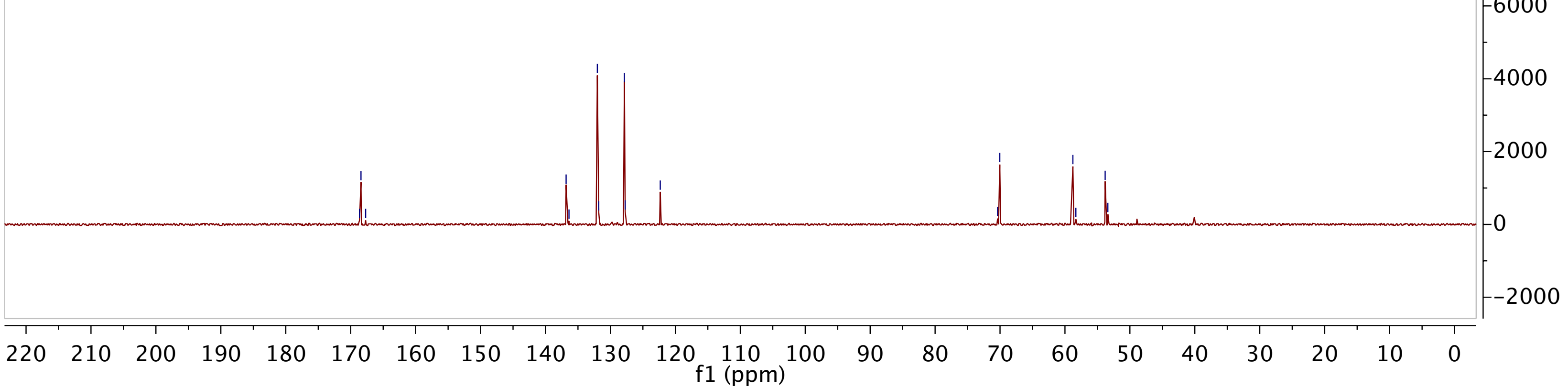

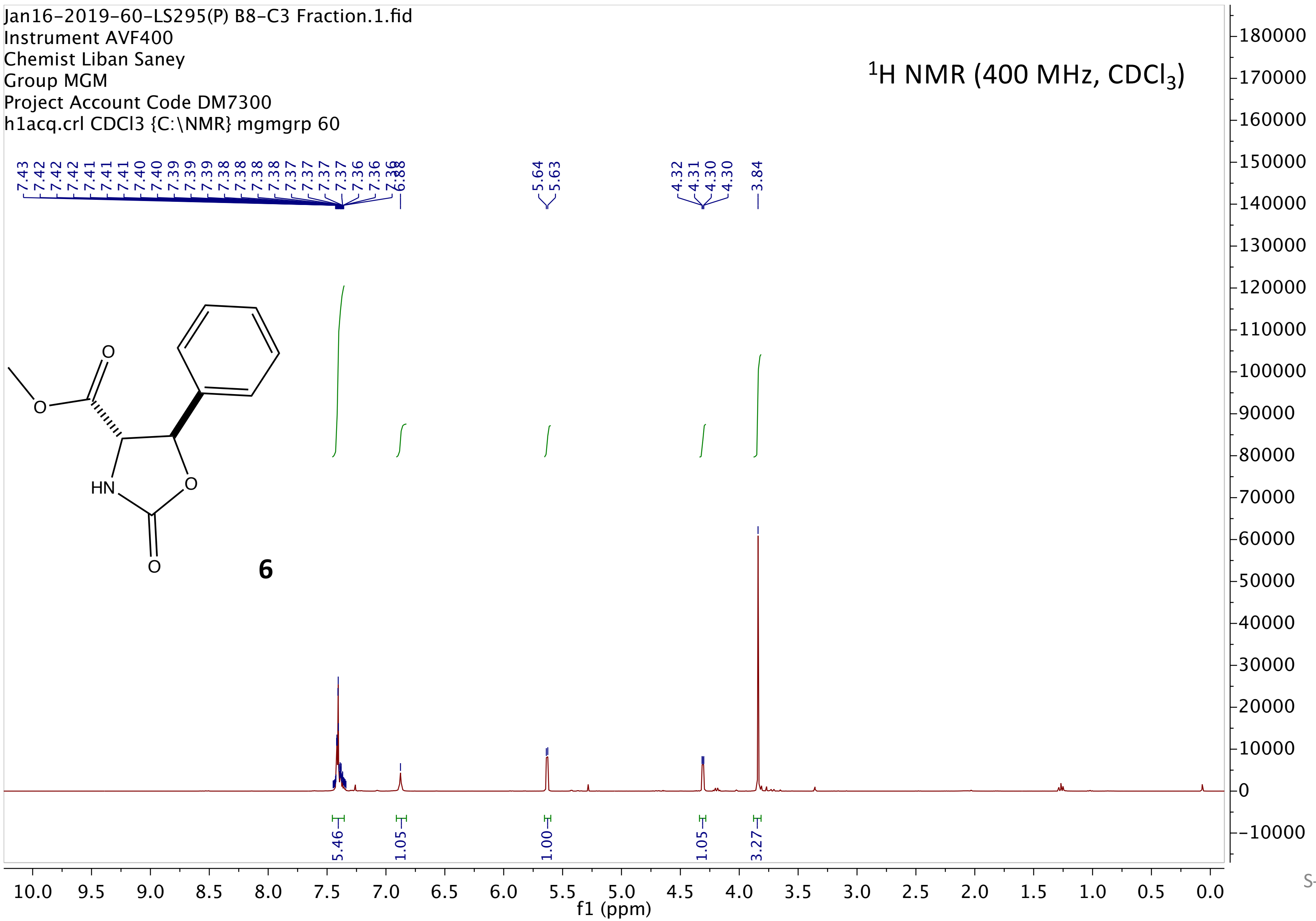
Jan16-2019-60-LS295(P) B8-C3 Fraction.1.fid
Instrument AVF400
Chemist Liban Saney
Group MGM
Project Account Code DM7300
h1acq.crl CDCl3 {C:\NMR} mgmgrp 60
1H NMR (400 MHz, CDCl3)
7.43 7.42 7.42 7.42 7.41 7.41 7.41 7.40 7.40 7.39 7.39 7.39 7.38 7.38 7.38 7.38 7.37 7.37 7.37 7.37 7.36 7.36 7.36 6.88
5.64 5.63
4.32 4.31 4.30 4.30
3.84
HN
O
6
5.46
1.05
1.00
1.05
3.27
f1 (ppm)

Jan16-2019-60-LS295(P) B8-C3 Fraction.4.fid
Instrument AVF400
Chemist Liban Saney
Group MGM
Project Account Code DM7300
c13acq_512.crl CDCl3 {C:\NMR} mgmgrp 60

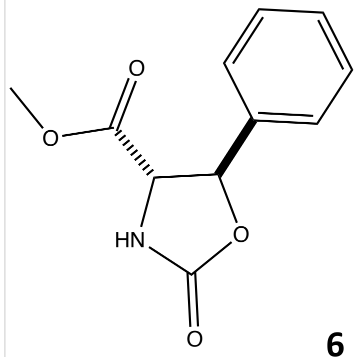

6

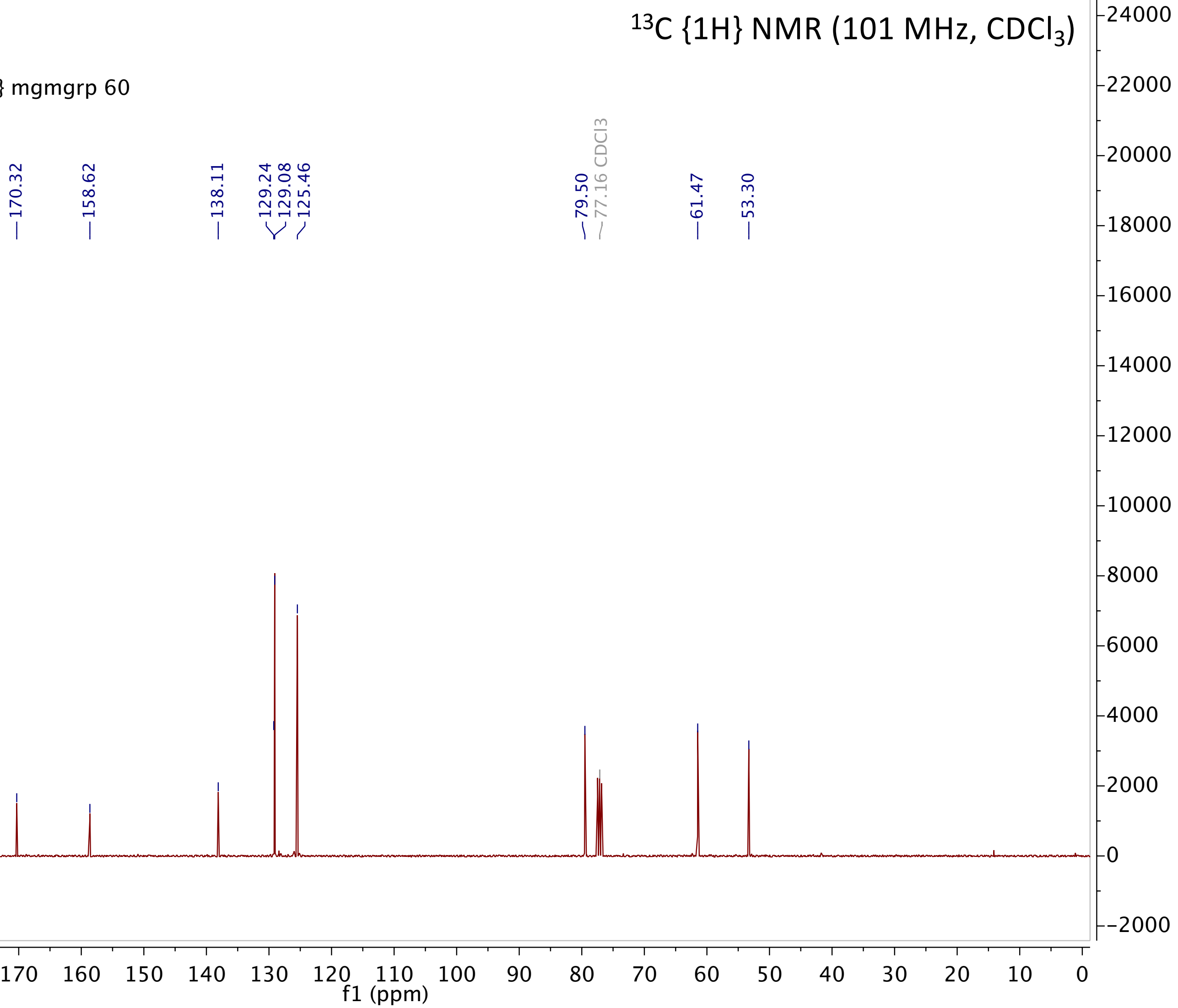

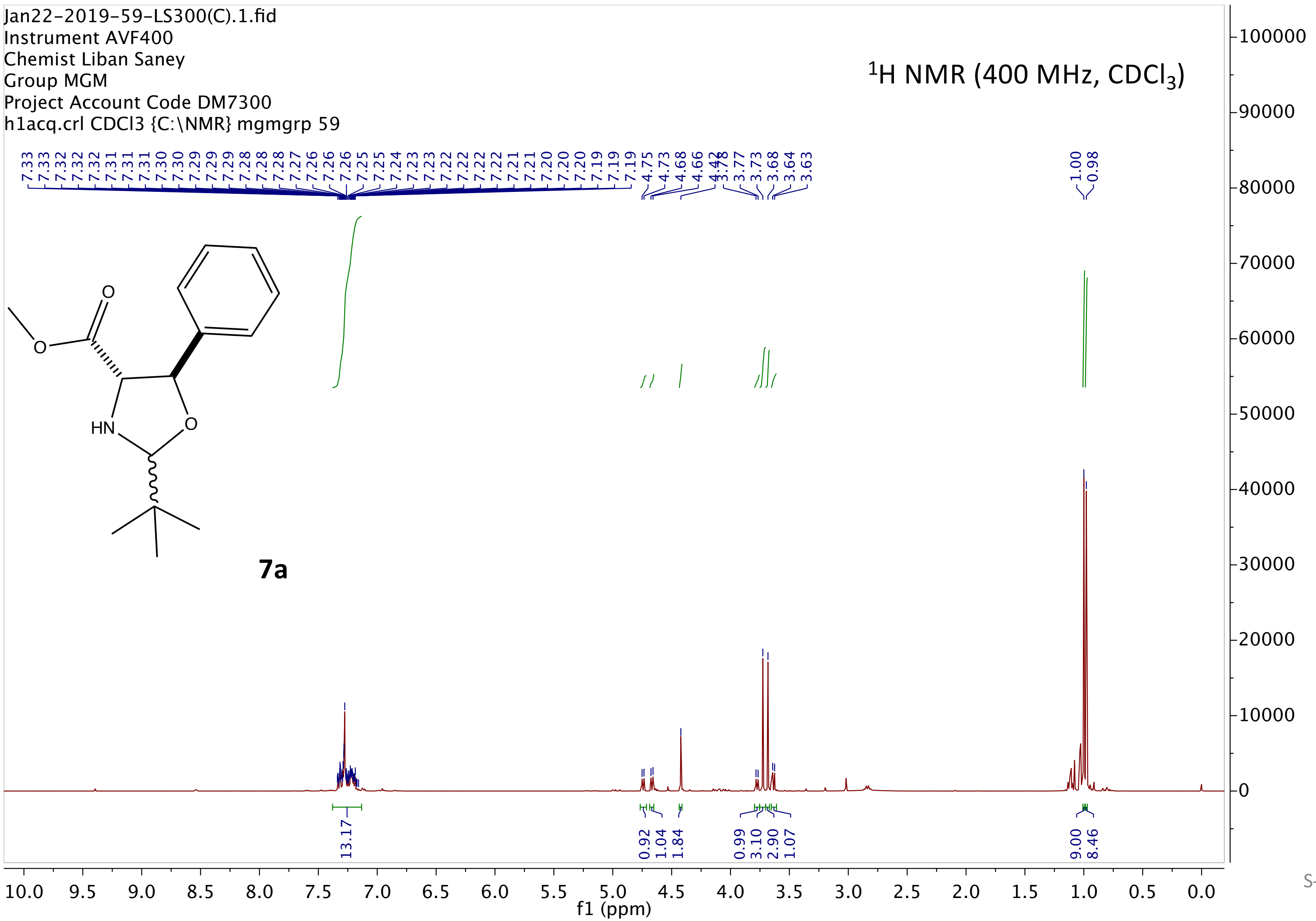
Jan22-2019-59-LS300(C).1.fid
Instrument AVF400
Chemist Liban Saney
Group MGM
Project Account Code DM7300
h1acq.crl CDCl3 {C:\NMR} mgmgrp 59
1H NMR (400 MHz, CDCl3)
7a
O
O
HN
O
f1 (ppm)

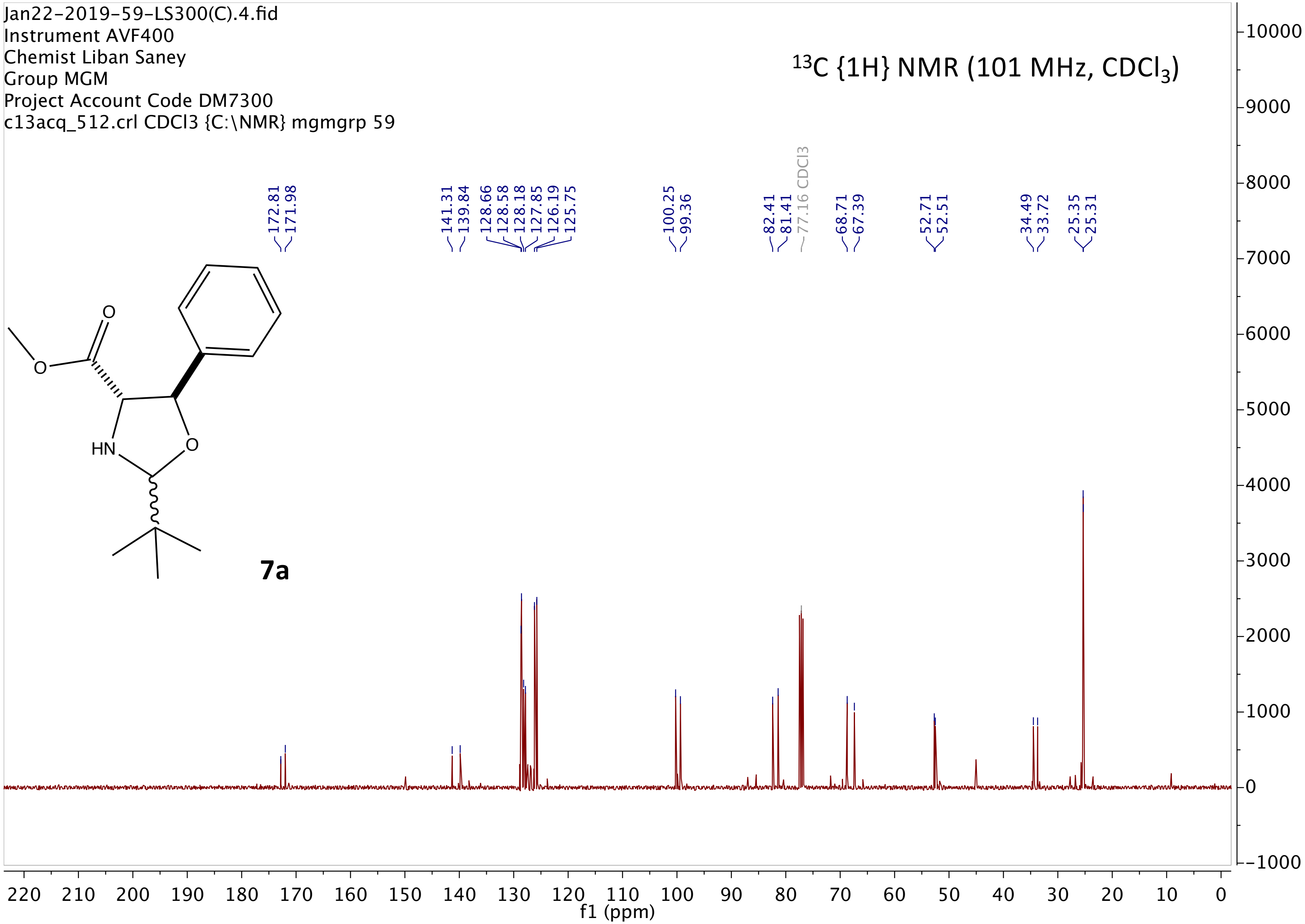
Jan22-2019-59-LS300(C).4.fid
Instrument AVF400
Chemist Liban Saney
Group MGM
Project Account Code DM7300
c13acq_512.crl CDCl3 {C:\NMR} mgmgrp 59
^{13}C {1H} NMR (101 MHz, $CDCl_3$)
172.81
171.98
141.31
139.84
128.66
128.58
128.18
127.85
126.19
125.75
100.25
99.36
82.41
81.41
77.16 CDCl3
68.71
67.39
52.71
52.51
34.49
33.72
25.35
25.31
O
O
HN
O
7a
f1 (ppm)

Apr16-2019-60-LS381(C).1.fid
Instrument AVF400
Chemist Liban Saney
Group MGM
Project Account Code DM7300
h1acq.crl CDCl3 {C:\NMR} mgmgrp 60

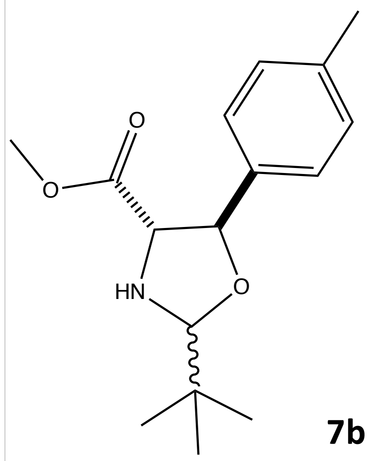

7b

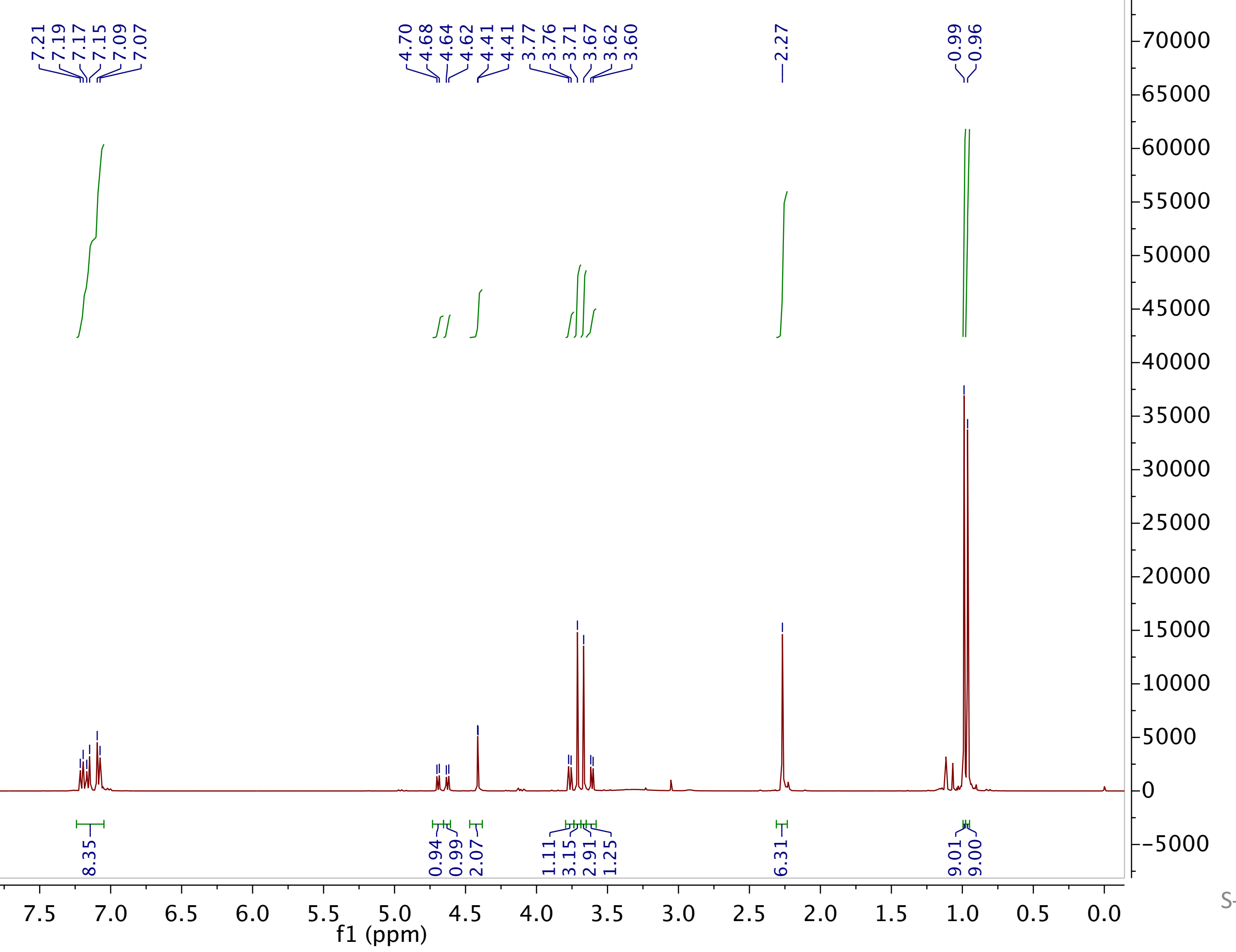

Apr16-2019-60-LS381(C).4.fid
Instrument AVF400
Chemist Liban Saney
Group MGM
Project Account Code DM7300
c13acq_512.crl CDCl3 {C:\NMR} mgmgrp 60

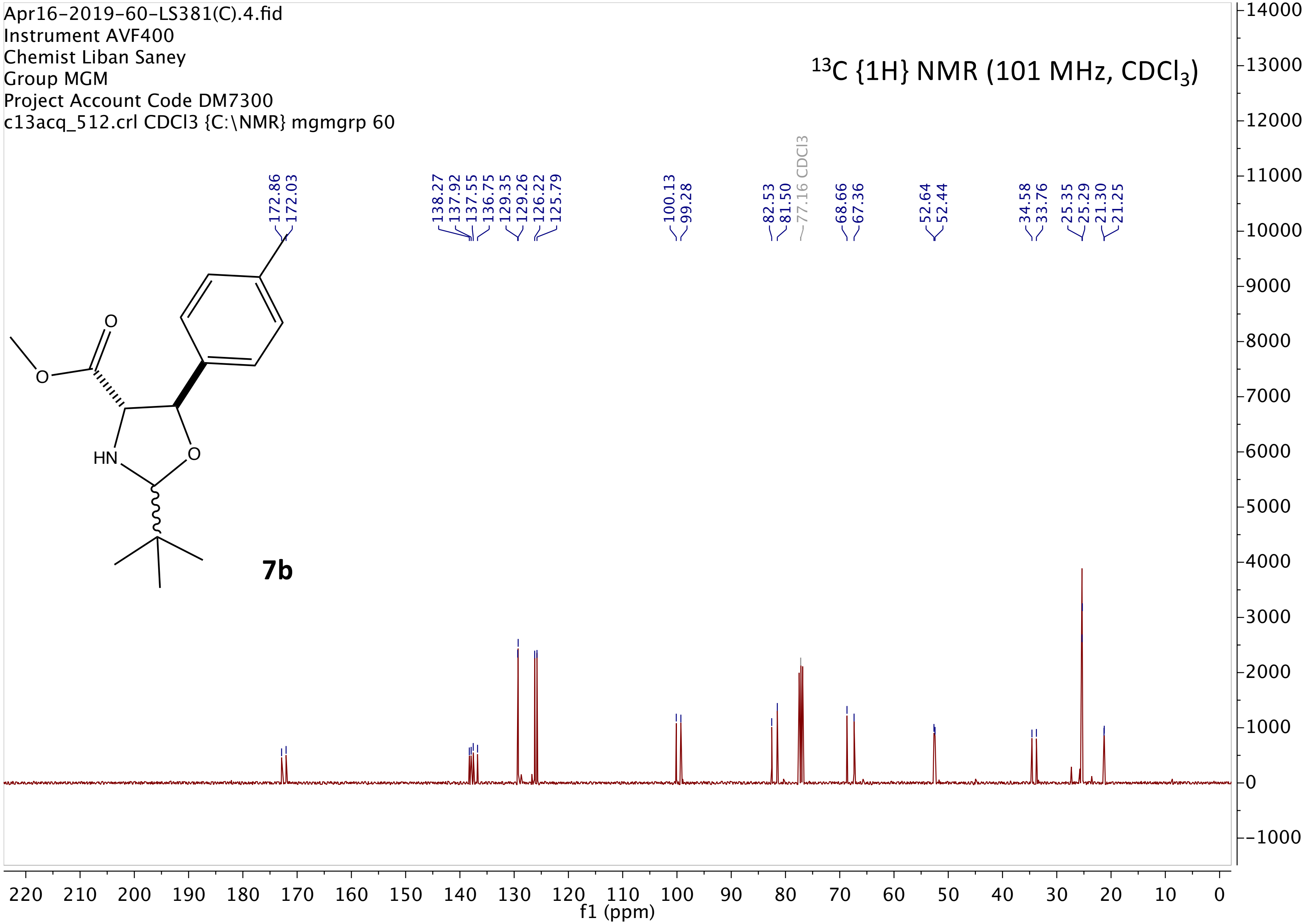

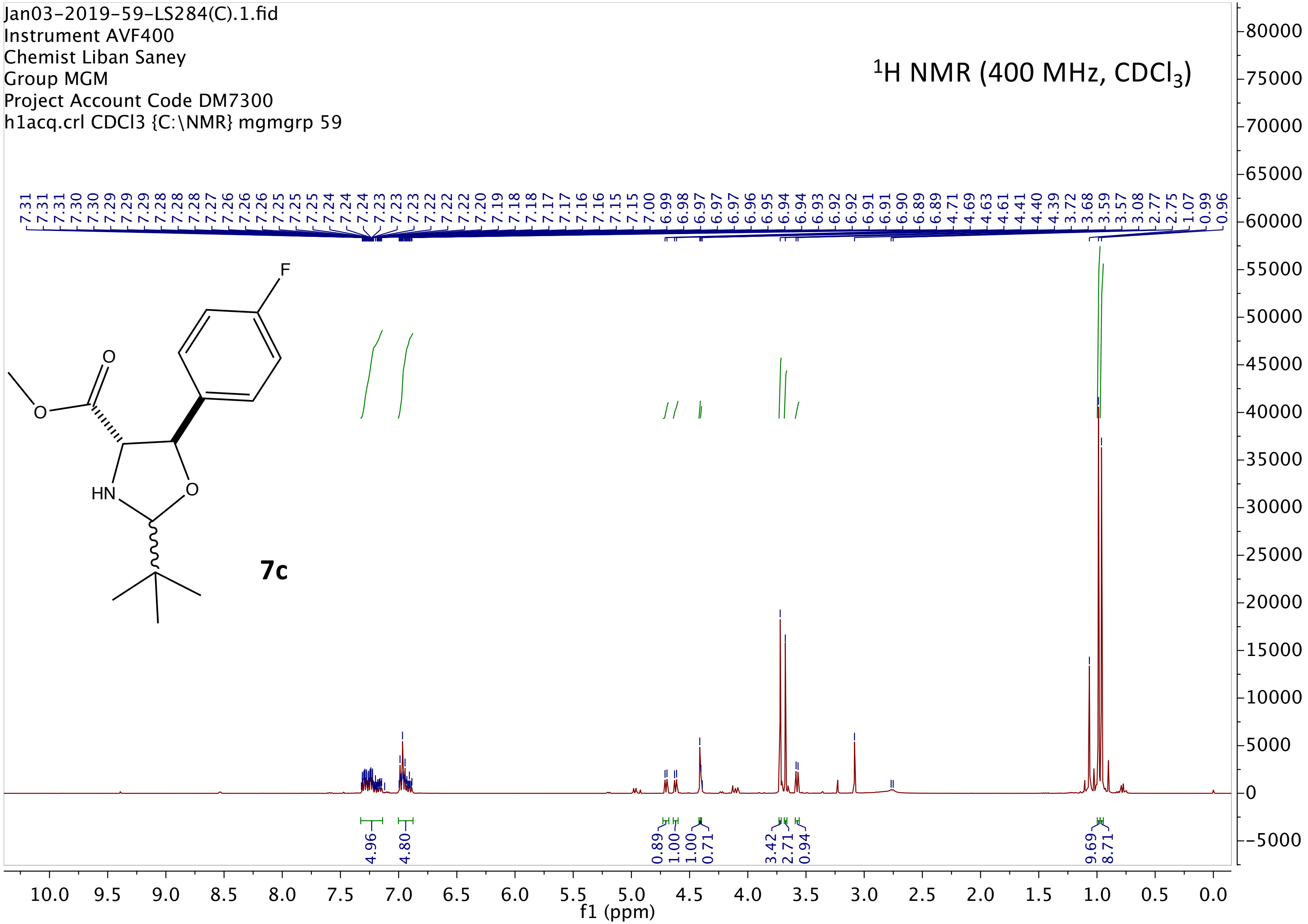
Jan03-2019-59-LS284(C).1.fid
Instrument AVF400
Chemist Liban Saney
Group MGM
Project Account Code DM7300
h1acq.crl CDCl3 {C:\NMR} mgmgrp 59
^{1}H NMR (400 MHz, CDCl$_3$)
7c
F
O
O
HN
O
4.96
4.80
0.89
1.00
1.00
0.71
3.42
2.71
0.94
9.69
8.71
f1 (ppm)

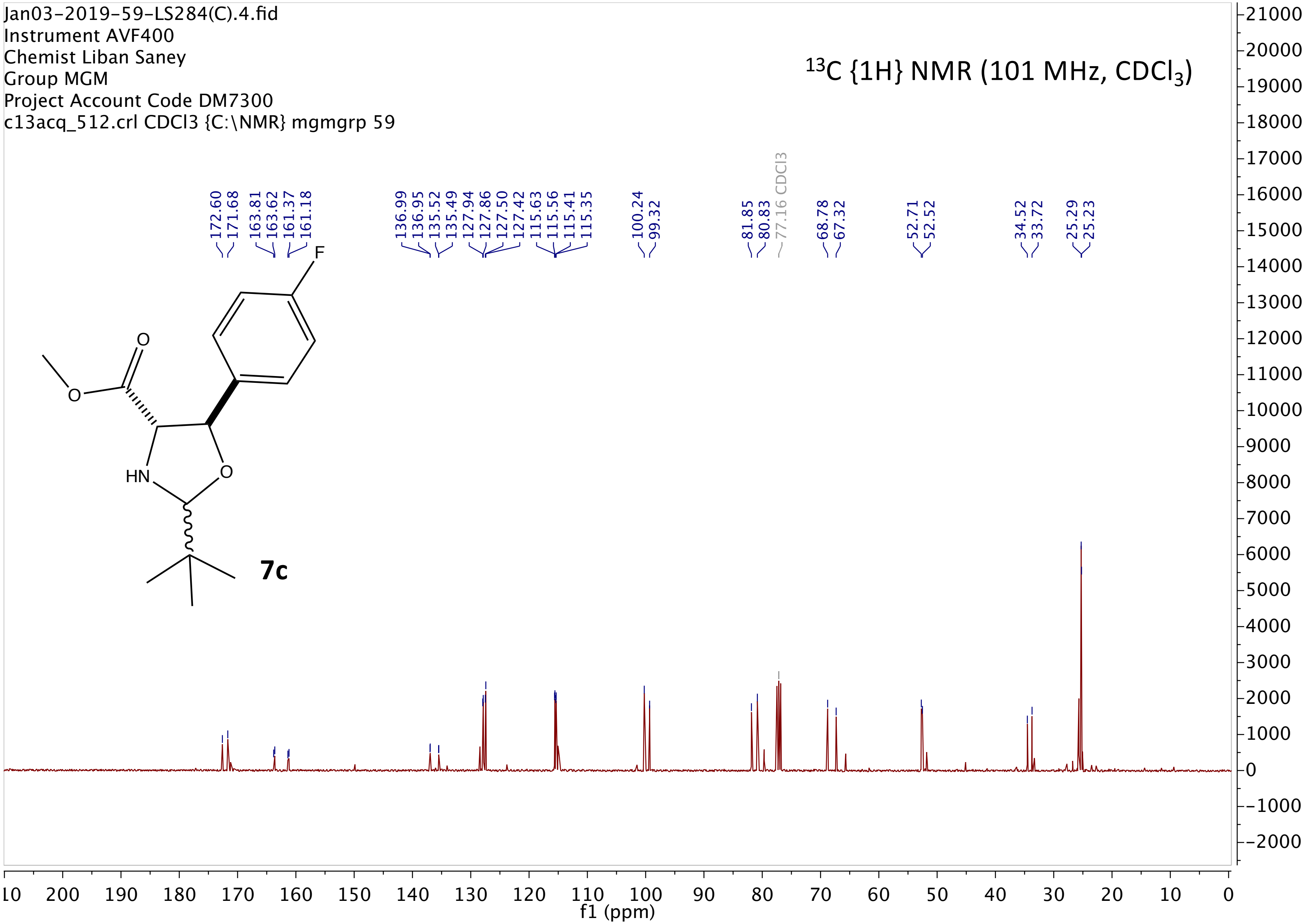

Jan03-2019-59-LS284(C).4.fid
Instrument AVF400
Chemist Liban Saney
Group MGM
Project Account Code DM7300
c13acq_512.crl CDCl3 {C:\NMR} mgmgrp 59
13C {1H} NMR (101 MHz, CDCl3)
172.60
171.68
163.81
163.62
161.37
161.18
136.99
136.95
135.52
135.49
127.94
127.86
127.50
127.42
115.63
115.56
115.41
115.35
100.24
99.32
81.85
80.83
77.16 CDCl3
68.78
67.32
52.71
52.52
34.52
33.72
25.29
25.23
F
O
O
HN
O
7c
f1 (ppm)

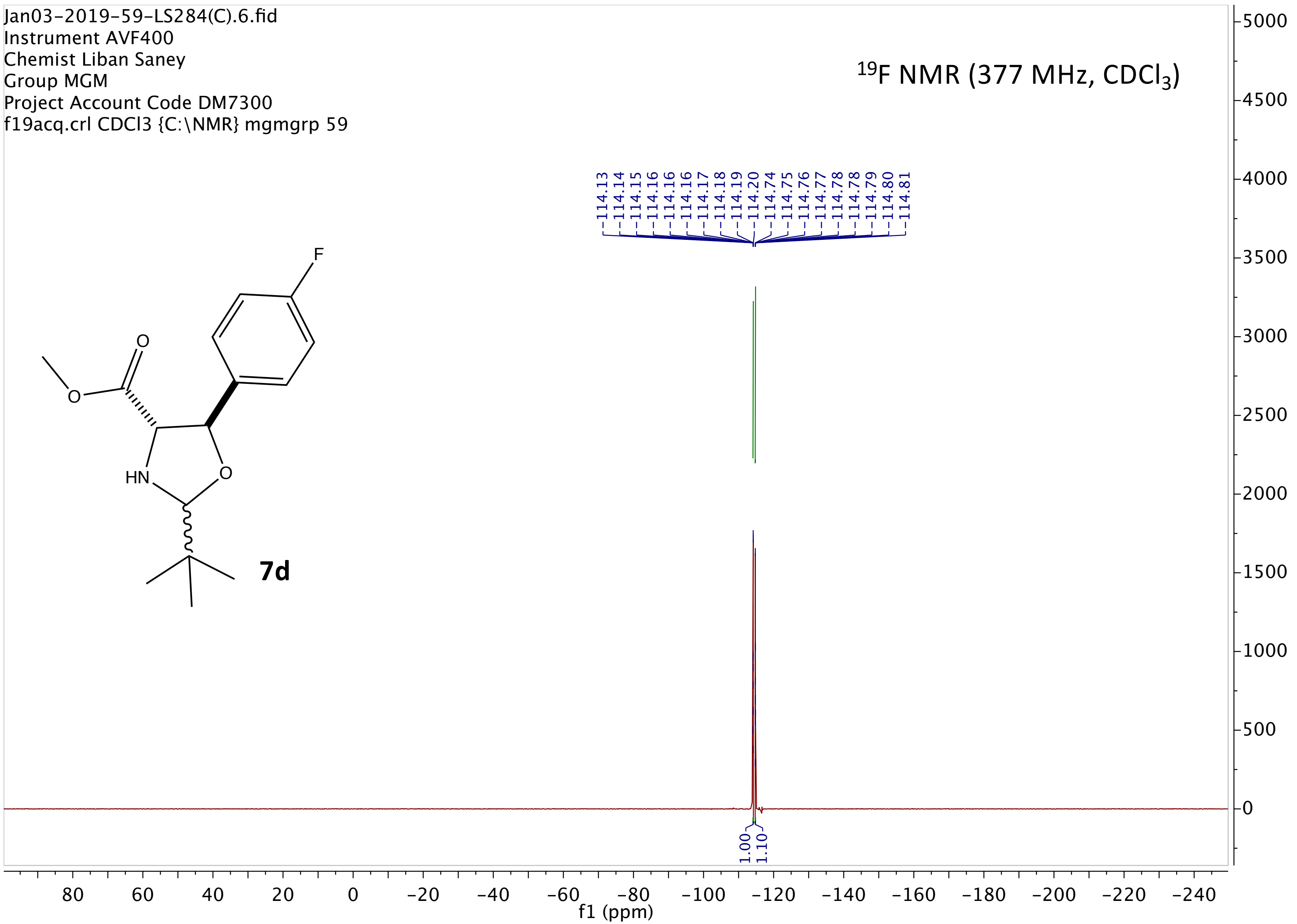
Jan03-2019-59-LS284(C).6.fid
Instrument AVF400
Chemist Liban Saney
Group MGM
Project Account Code DM7300
f19acq.crl CDCl3 {C:\NMR} mgmgrp 59
19F NMR (377 MHz, CDCl3)
-114.13
-114.14
-114.15
-114.16
-114.16
-114.16
-114.17
-114.18
-114.19
-114.20
-114.74
-114.75
-114.76
-114.77
-114.78
-114.78
-114.79
-114.80
-114.81
1.00
1.10
F
O
O
HN
O
7d
f1 (ppm)

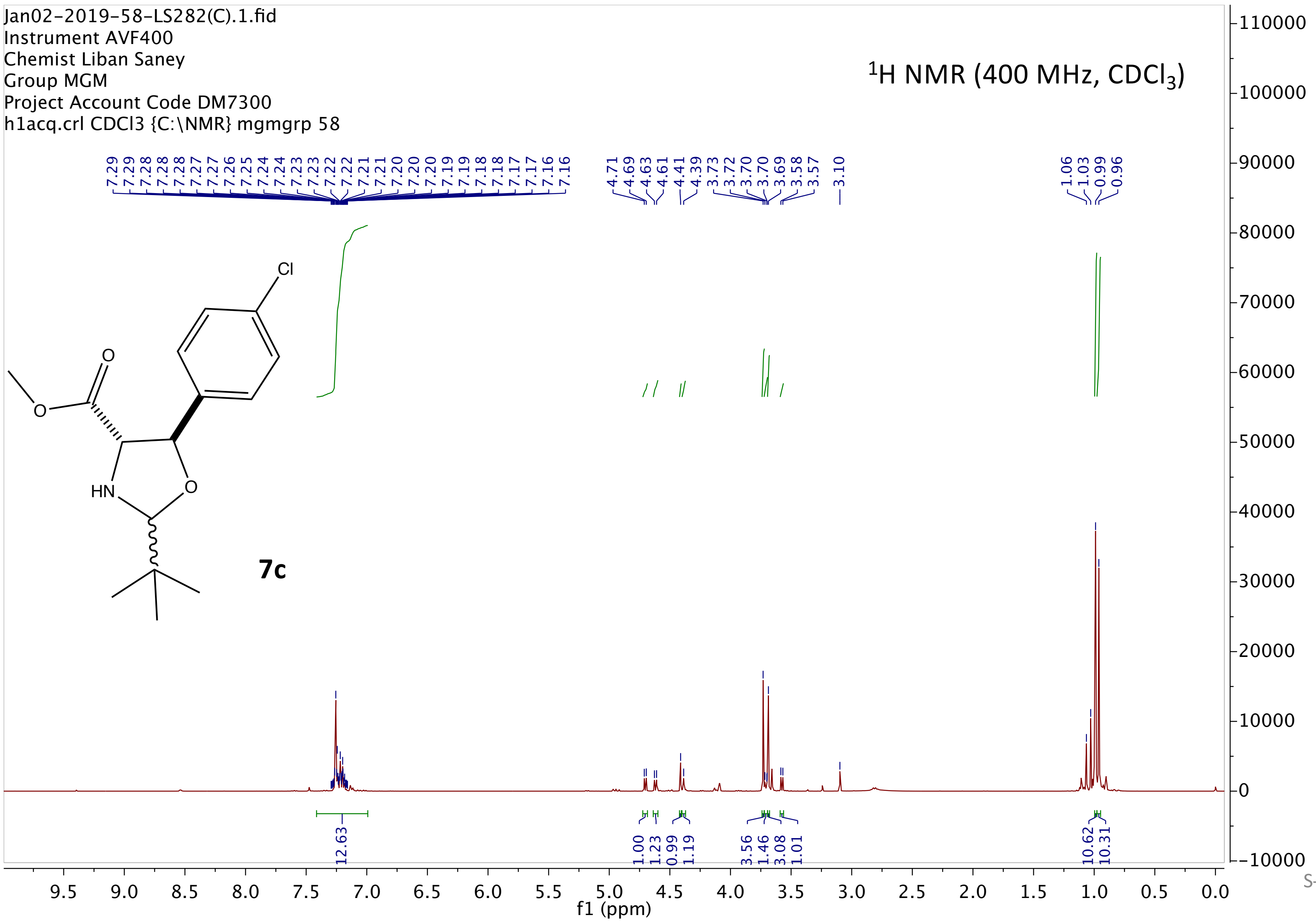
Jan02-2019-58-LS282(C).1.fid
Instrument AVF400
Chemist Liban Saney
Group MGM
Project Account Code DM7300
h1acq.crl CDCl3 {C:\NMR} mgmgrp 58
¹H NMR (400 MHz, CDCl₃)
7.29 7.29 7.28 7.28 7.28 7.27 7.27 7.26 7.25 7.24 7.24 7.23 7.23 7.22 7.22 7.21 7.21 7.20 7.20 7.19 7.19 7.18 7.17 7.17 7.16 7.16
4.71 4.69 4.63 4.61 4.41 4.39 3.73 3.72 3.70 3.69 3.58 3.57 3.10
1.06 1.03 0.99 0.96
Cl
O
O
HN
O
7c
12.63
1.00 1.23 0.99 1.19 3.56 1.46 3.08 1.01
10.62 10.31
f1 (ppm)

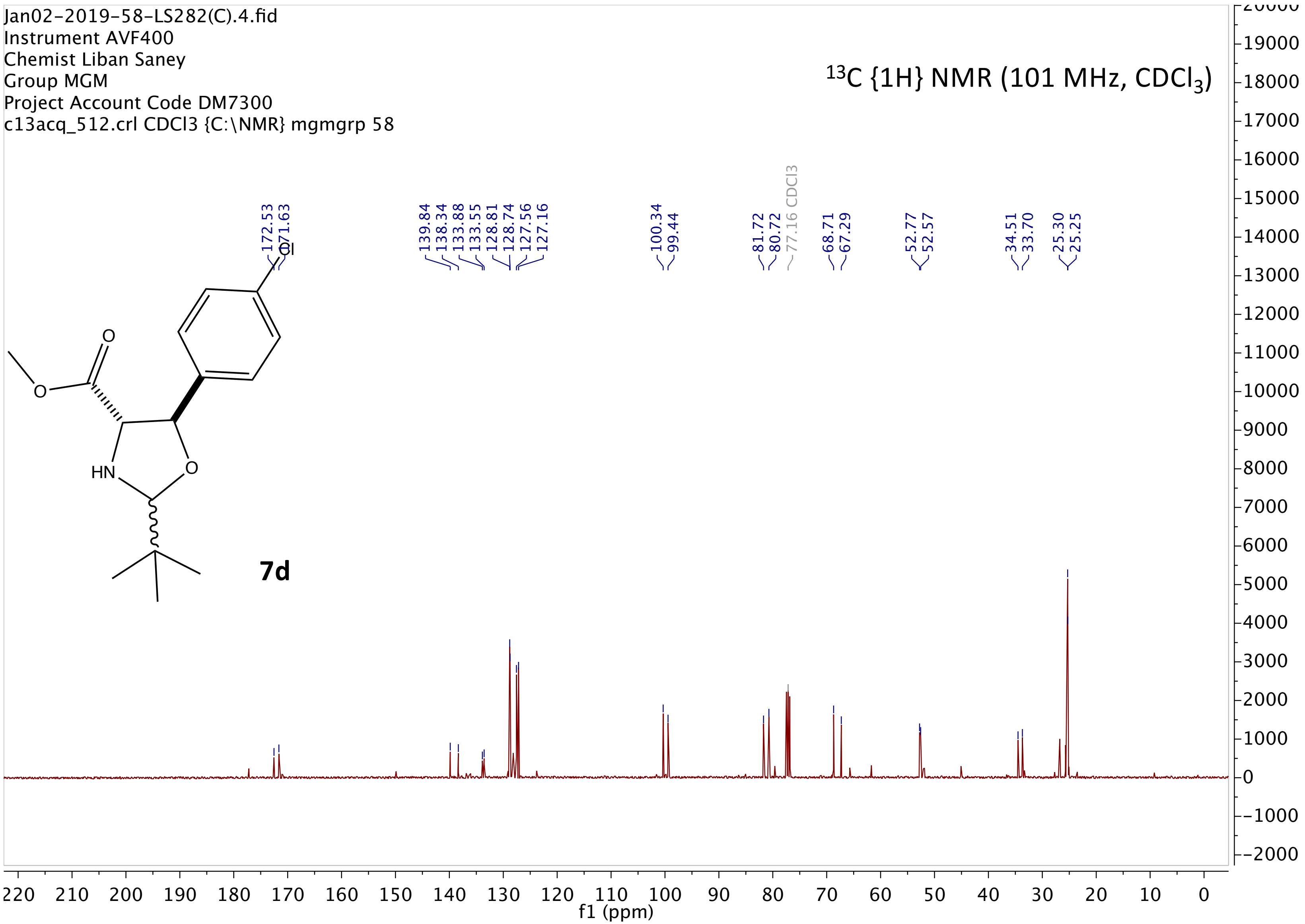
Jan02-2019-58-LS282(C).4.fid
Instrument AVF400
Chemist Liban Saney
Group MGM
Project Account Code DM7300
c13acq_512.crl CDCl3 {C:\NMR} mgmgrp 58
^{13}C {1H} NMR (101 MHz, $CDCl_3$)
172.53
171.63
139.84
138.34
133.88
133.55
128.81
128.74
127.56
127.16
100.34
99.44
81.72
80.72
77.16 CDCl3
68.71
67.29
52.77
52.57
34.51
33.70
25.30
25.25
O
O
HN
O
Cl
7d
f1 (ppm)

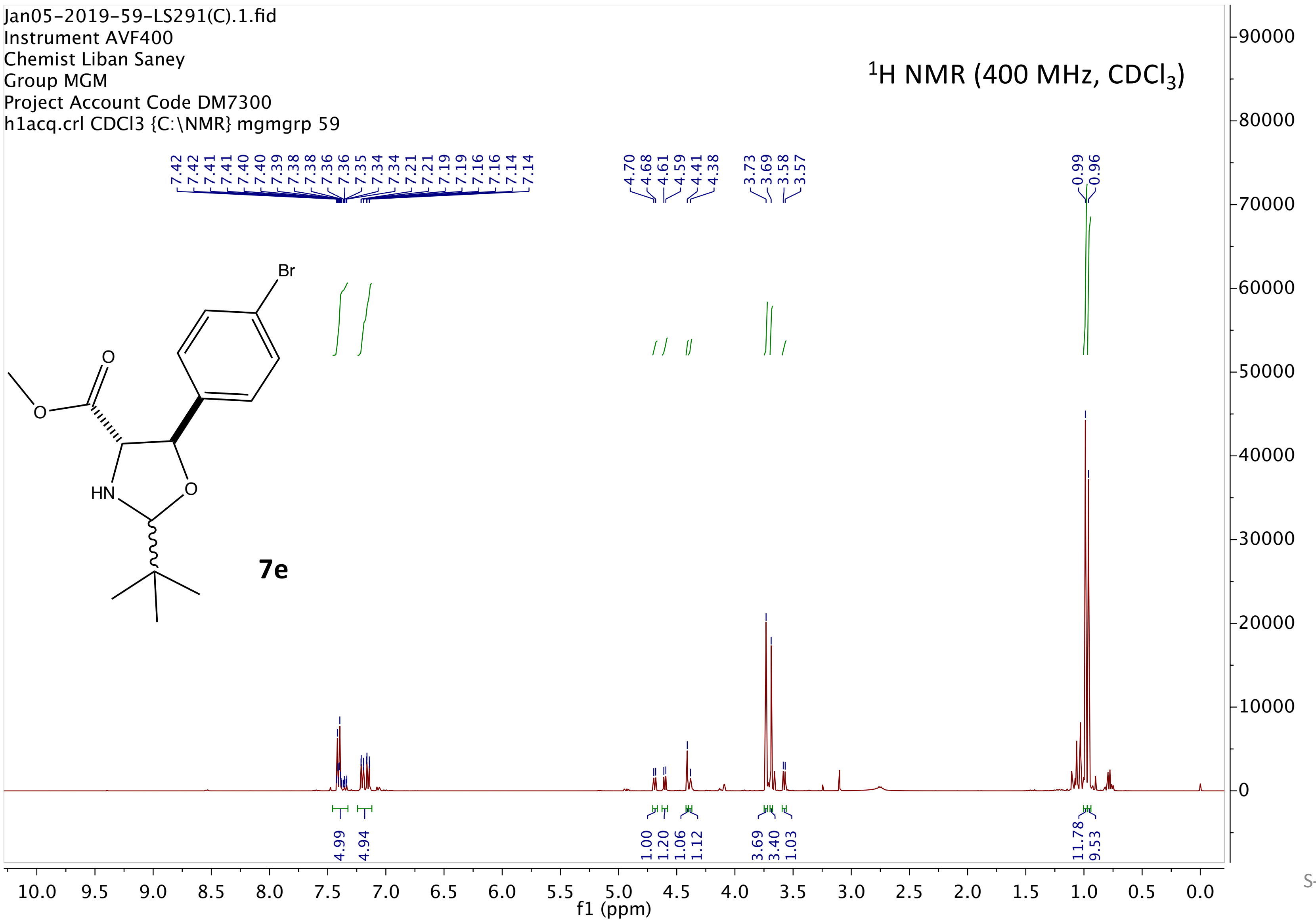
Jan05-2019-59-LS291(C).1.fid
Instrument AVF400
Chemist Liban Saney
Group MGM
Project Account Code DM7300
h1acq.crl CDCl3 {C:\NMR} mgmgrp 59
1H NMR (400 MHz, CDCl3)
7.42 7.42 7.41 7.41 7.40 7.40 7.39 7.38 7.38 7.36 7.36 7.35 7.34 7.34 7.21 7.21 7.19 7.19 7.16 7.16 7.14 7.14
4.70 4.68 4.61 4.59 4.41 4.38
3.73 3.69 3.58 3.57
0.99 0.96
Br
O
O
HN
O
7e
4.99 4.94 1.00 1.20 1.06 1.12 3.69 3.40 1.03 11.78 9.53
f1 (ppm)

Jan05-2019-59-LS291(C).4.fid
Instrument AVF400
Chemist Liban Saney
Group MGM
Project Account Code DM7300
c13acq_512.crl CDCl3 {C:\NMR} mgmgrp 59

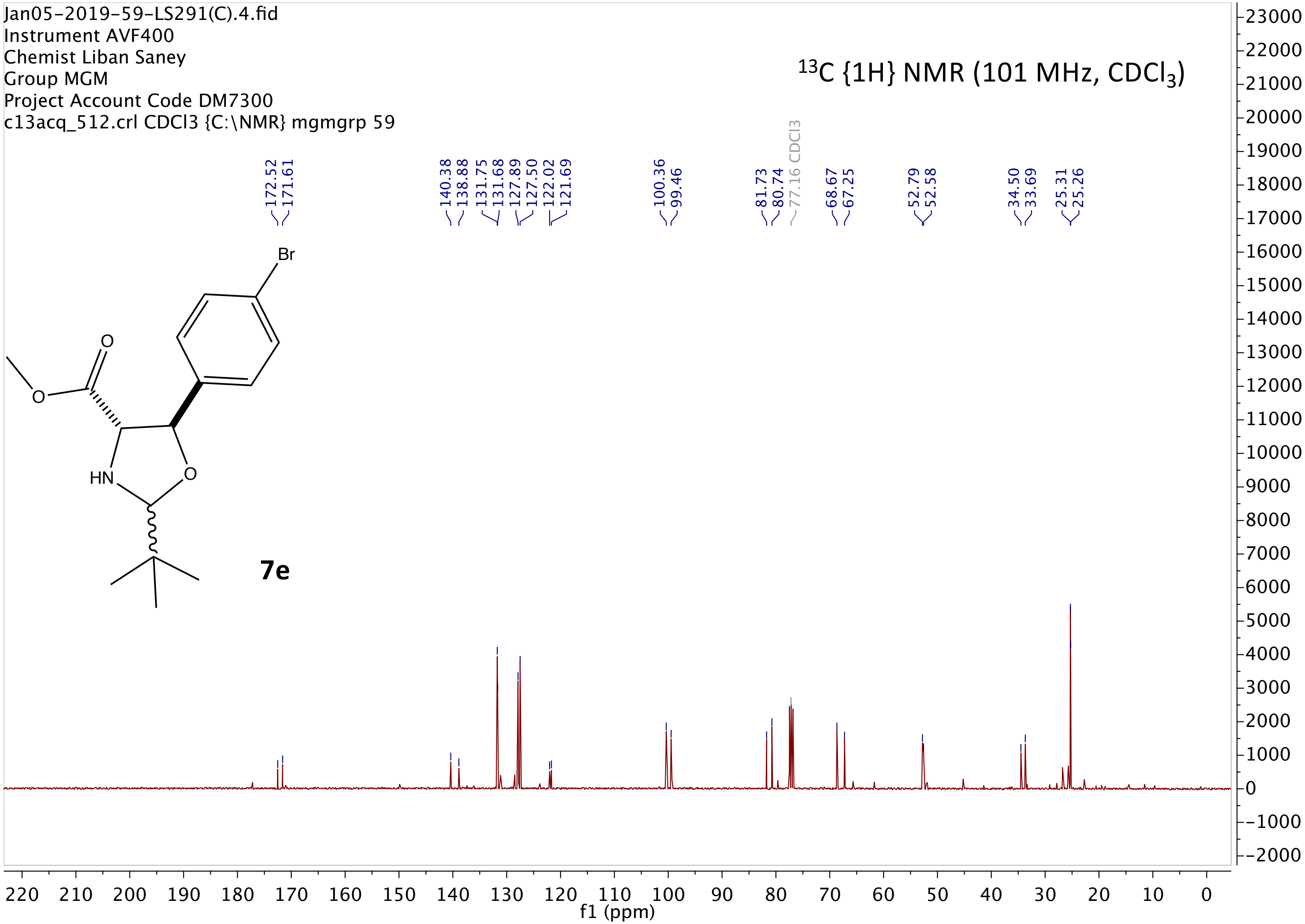

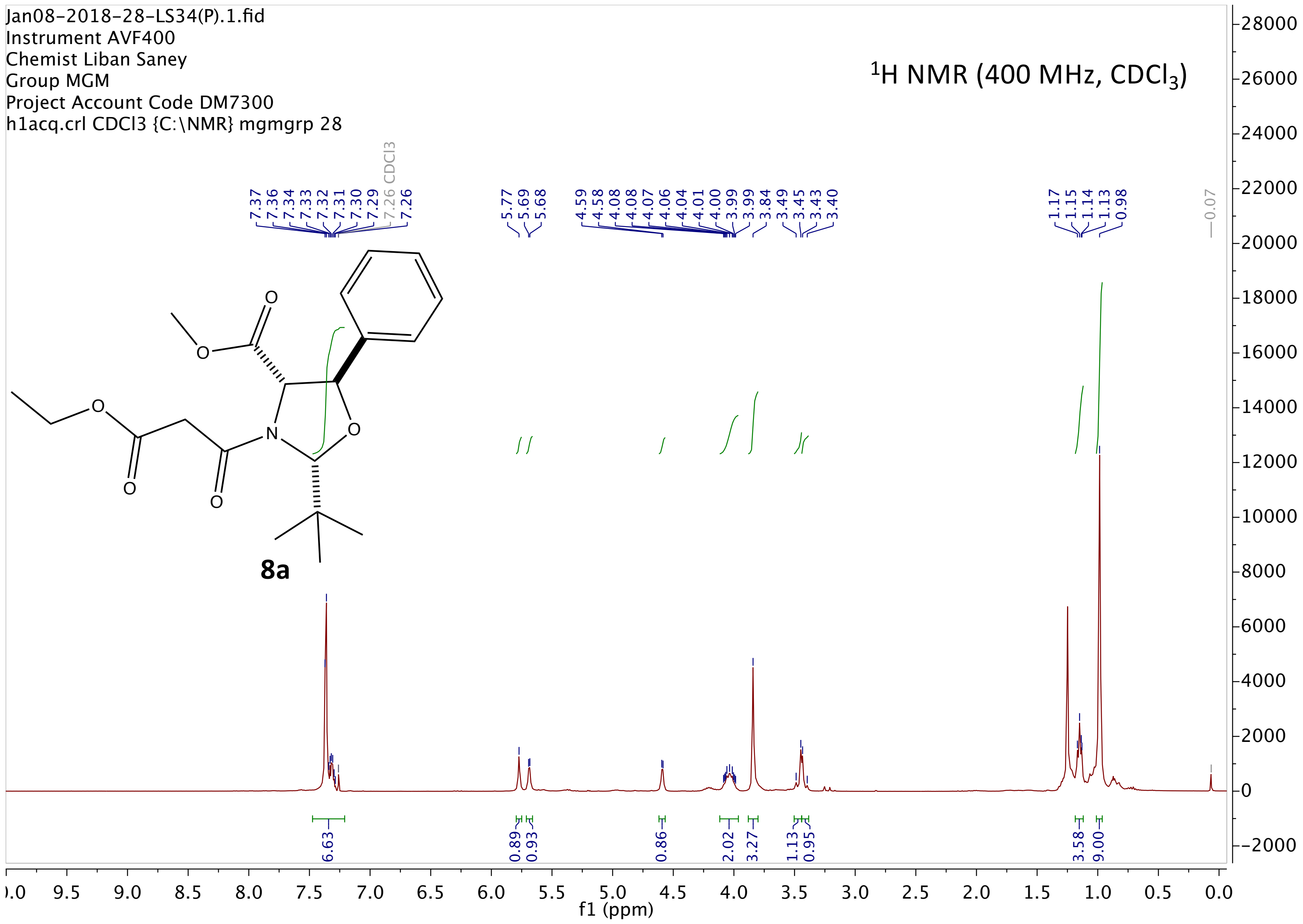
Jan08-2018-28-LS34(P).1.fid
Instrument AVF400
Chemist Liban Saney
Group MGM
Project Account Code DM7300
h1acq.crl CDCl3 {C:\NMR} mgmgrp 28
1H NMR (400 MHz, CDCl3)
7.37 7.36 7.34 7.33 7.32 7.31 7.30 7.29 7.26 CDCl3 7.26
5.77 5.69 5.68
4.59 4.58 4.08 4.08 4.07 4.06 4.04 4.01 4.00 3.99 3.99 3.84 3.49 3.45 3.43 3.40
1.17 1.15 1.14 1.13 0.98
-0.07
8a
6.63 0.89 0.93 0.86 2.02 3.27 1.13 0.95 3.58 9.00
f1 (ppm)

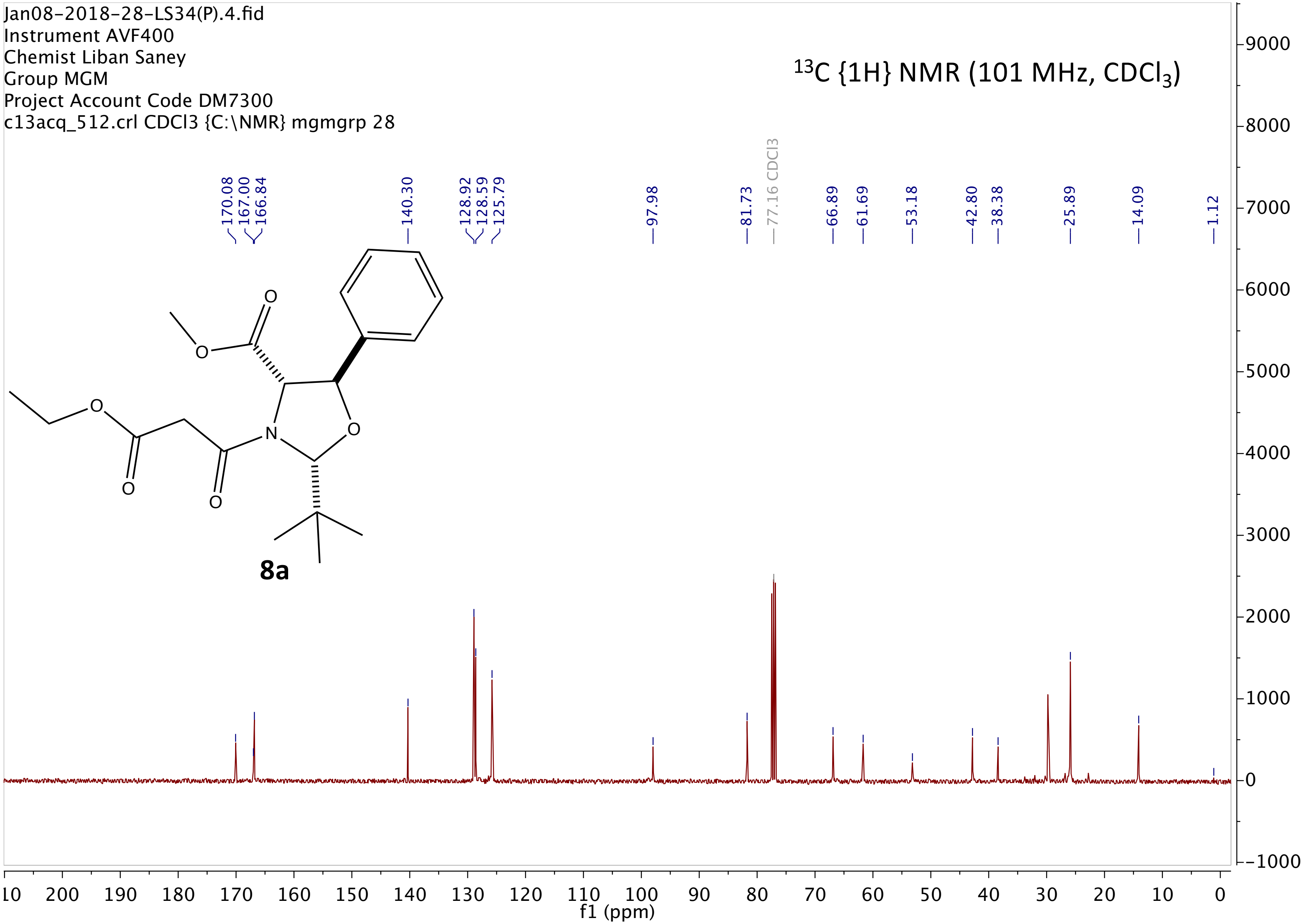
Jan08-2018-28-LS34(P).4.fid
Instrument AVF400
Chemist Liban Saney
Group MGM
Project Account Code DM7300
c13acq_512.crl CDCl3 {C:\NMR} mgmgrp 28
13C {1H} NMR (101 MHz, CDCl3)
170.08
167.00
166.84
140.30
128.92
128.59
125.79
97.98
81.73
77.16 CDCl3
66.89
61.69
53.18
42.80
38.38
25.89
14.09
1.12
8a
f1 (ppm)

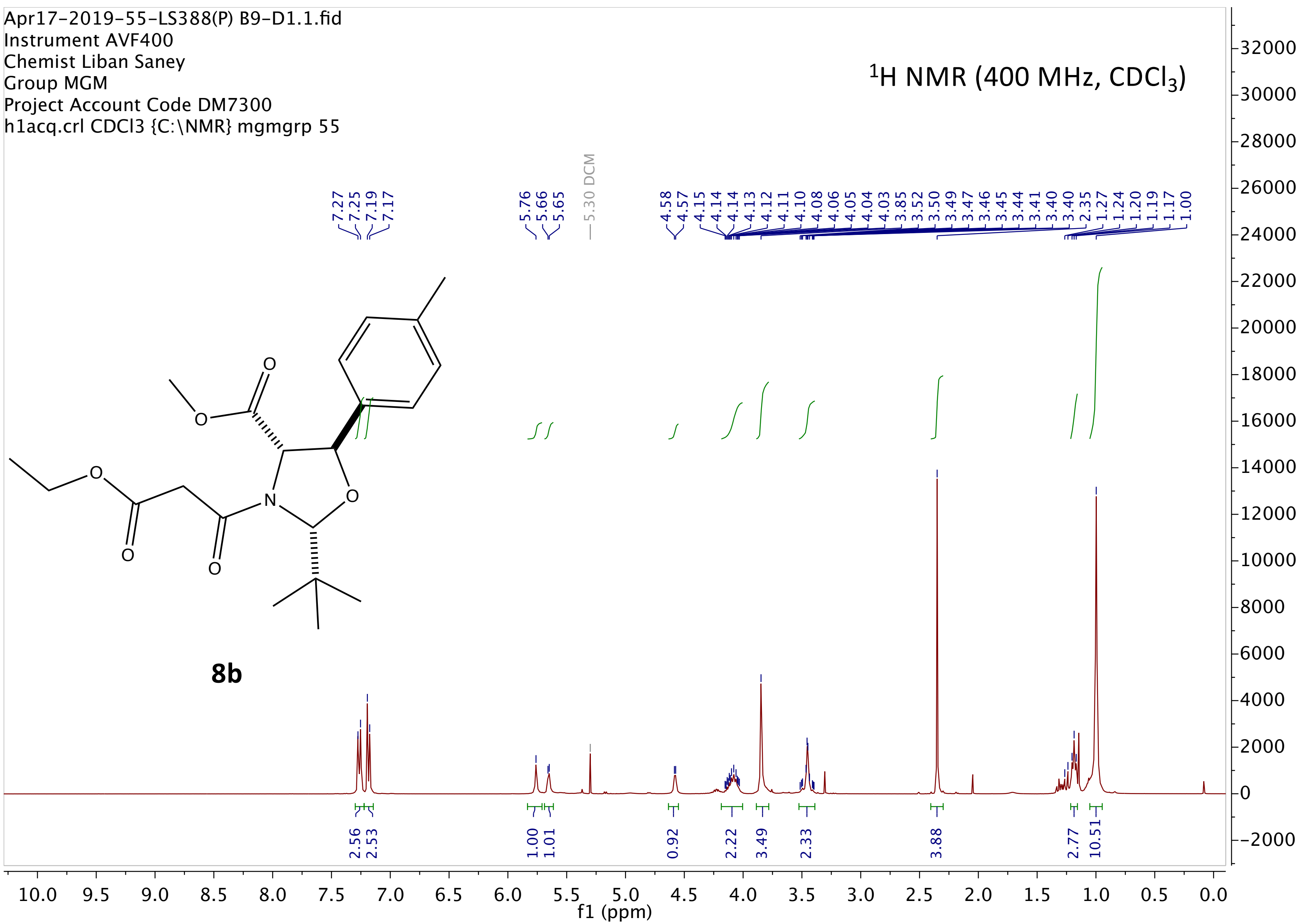
Apr17-2019-55-LS388(P) B9-D1.1.fid
Instrument AVF400
Chemist Liban Saney
Group MGM
Project Account Code DM7300
h1acq.crl CDCl3 {C:\NMR} mgmgrp 55
^{1}H NMR (400 MHz, $CDCl_3$)
7.27 7.25 7.19 7.17
5.76 5.66 5.65
5.30 DCM
4.58 4.57 4.15 4.14 4.14 4.13 4.12 4.11 4.10 4.08 4.06 4.05 4.04 4.03 3.85 3.52 3.50 3.49 3.47 3.46 3.45 3.44 3.41 3.40 2.35 1.27 1.24 1.20 1.19 1.17 1.00
O
O
N
O
O
O
O
8b
2.56 2.53 1.00 1.01 0.92 2.22 3.49 2.33 3.88 2.77 10.51
10.0 9.5 9.0 8.5 8.0 7.5 7.0 6.5 6.0 5.5 5.0 4.5 4.0 3.5 3.0 2.5 2.0 1.5 1.0 0.5 0.0
f1 (ppm)
32000 30000 28000 26000 24000 22000 20000 18000 16000 14000 12000 10000 8000 6000 4000 2000 0 -2000

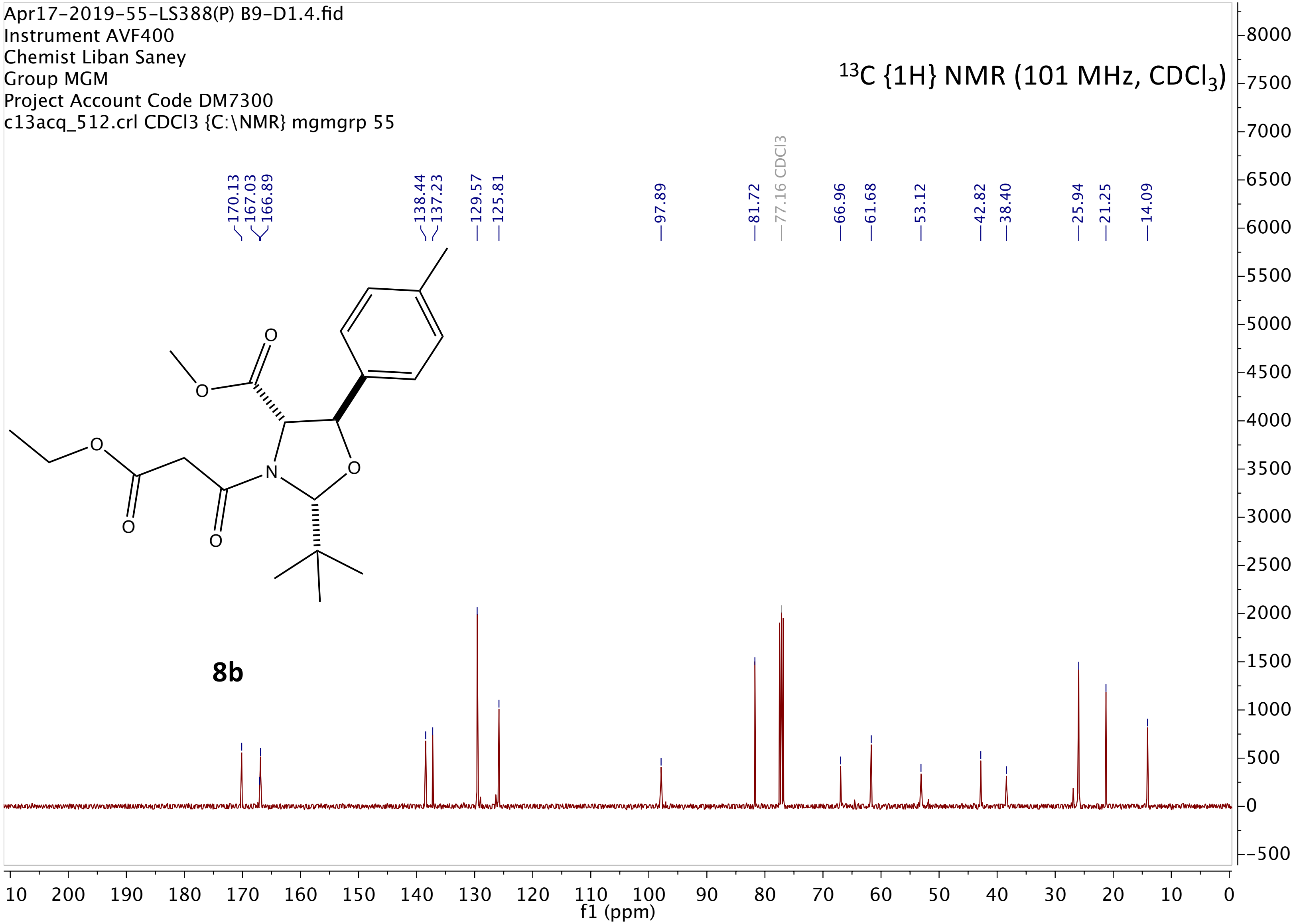
Apr17-2019-55-LS388(P) B9-D1.4.fid
Instrument AVF400
Chemist Liban Saney
Group MGM
Project Account Code DM7300
c13acq_512.crl CDCl3 {C:\NMR} mgmgrp 55
^{13}C {1H} NMR (101 MHz, CDCl$_3$)
170.13
167.03
166.89
138.44
137.23
129.57
125.81
97.89
81.72
77.16 CDCl3
66.96
61.68
53.12
42.82
38.40
25.94
21.25
14.09
8b
f1 (ppm)

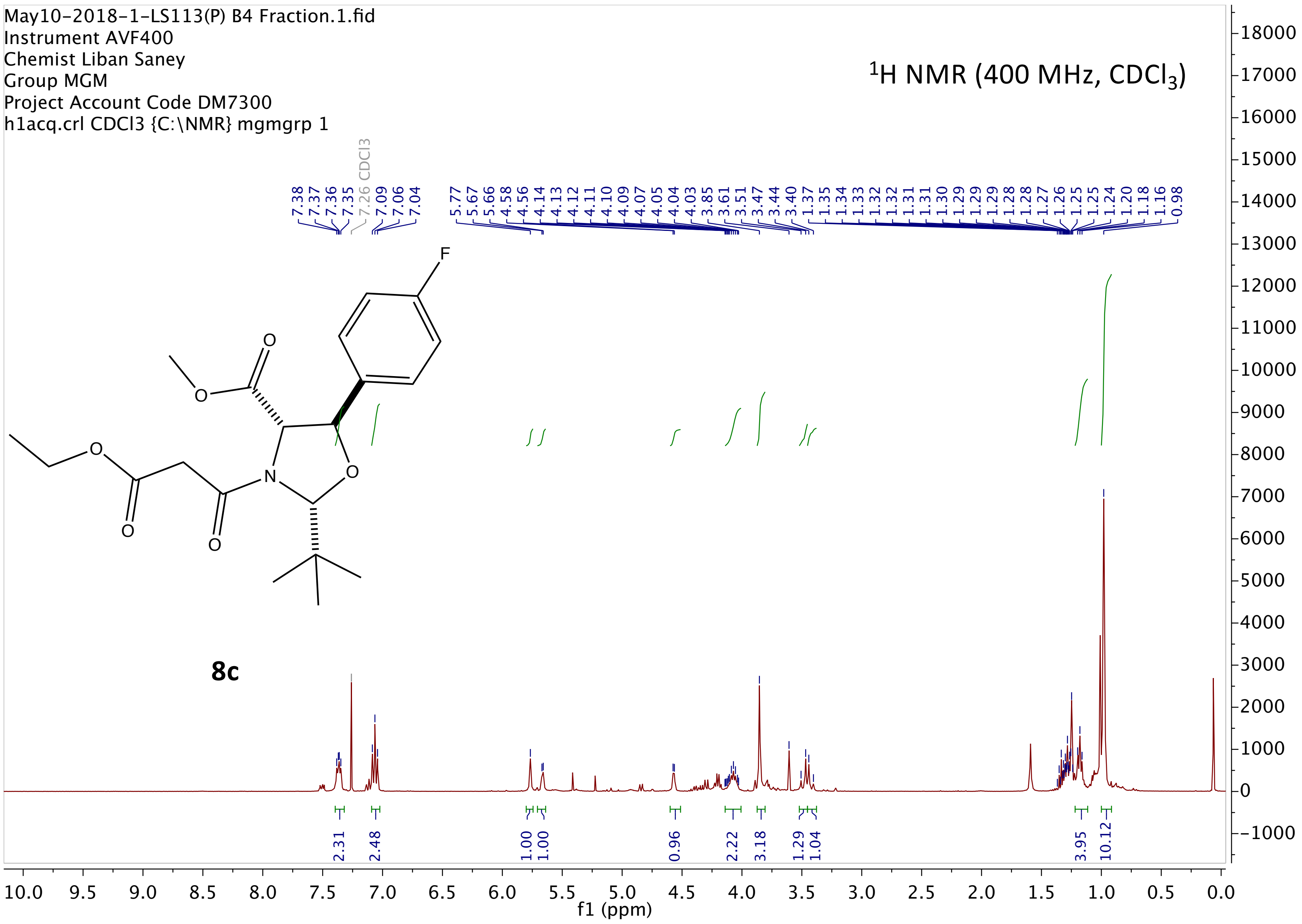
May10-2018-1-LS113(P) B4 Fraction.1.fid
Instrument AVF400
Chemist Liban Saney
Group MGM
Project Account Code DM7300
h1acq.crl CDCl3 {C:\NMR} mgmgrp 1
^{1}H NMR (400 MHz, $CDCl_3$)
8c
f1 (ppm)

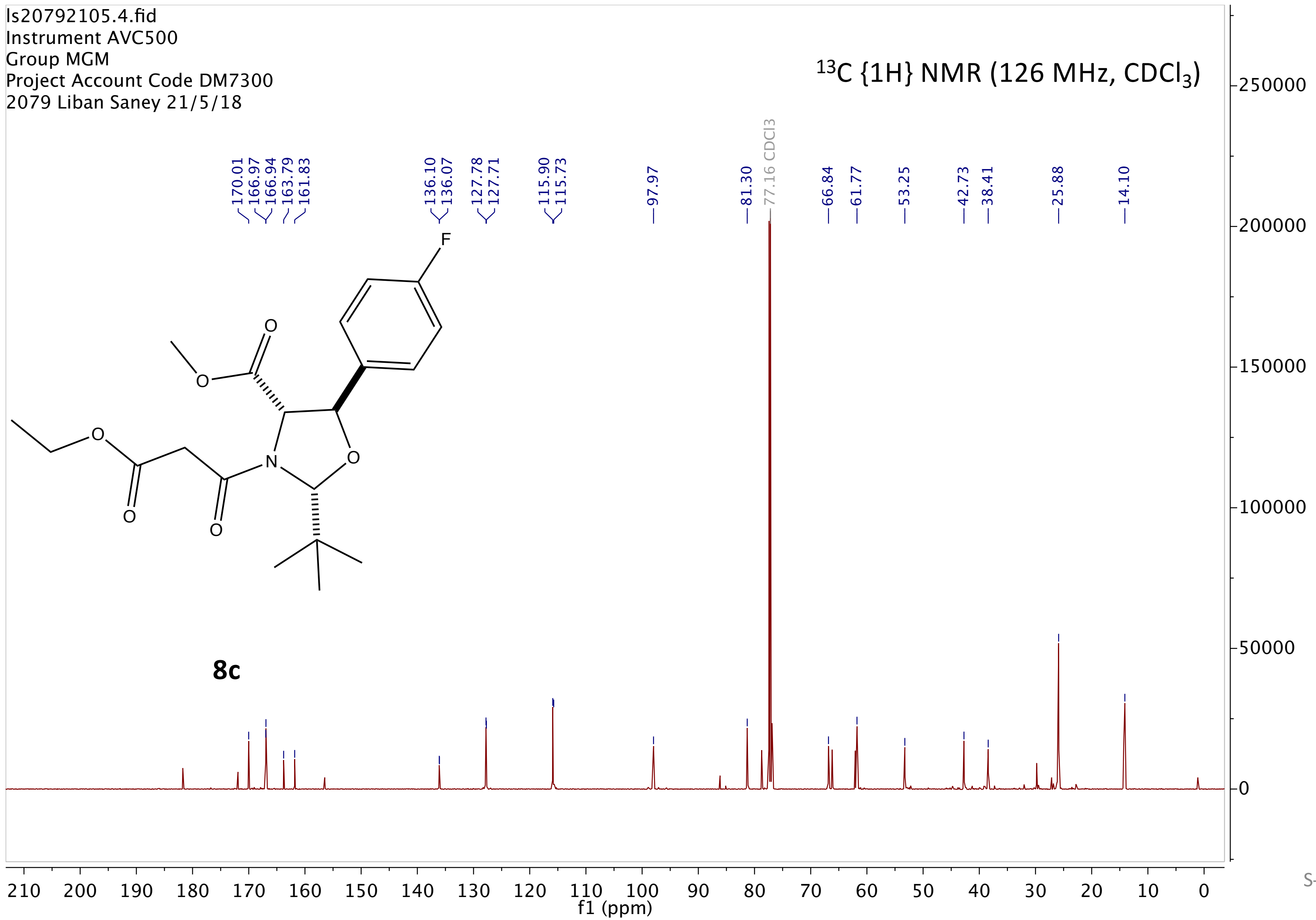
ls20792105.4.fid
Instrument AVC500
Group MGM
Project Account Code DM7300
2079 Liban Saney 21/5/18
13C {1H} NMR (126 MHz, CDCl3)
170.01
166.97
166.94
163.79
161.83
136.10
136.07
127.78
127.71
115.90
115.73
97.97
81.30
77.16 CDCl3
66.84
61.77
53.25
42.73
38.41
25.88
14.10
8c
f1 (ppm)

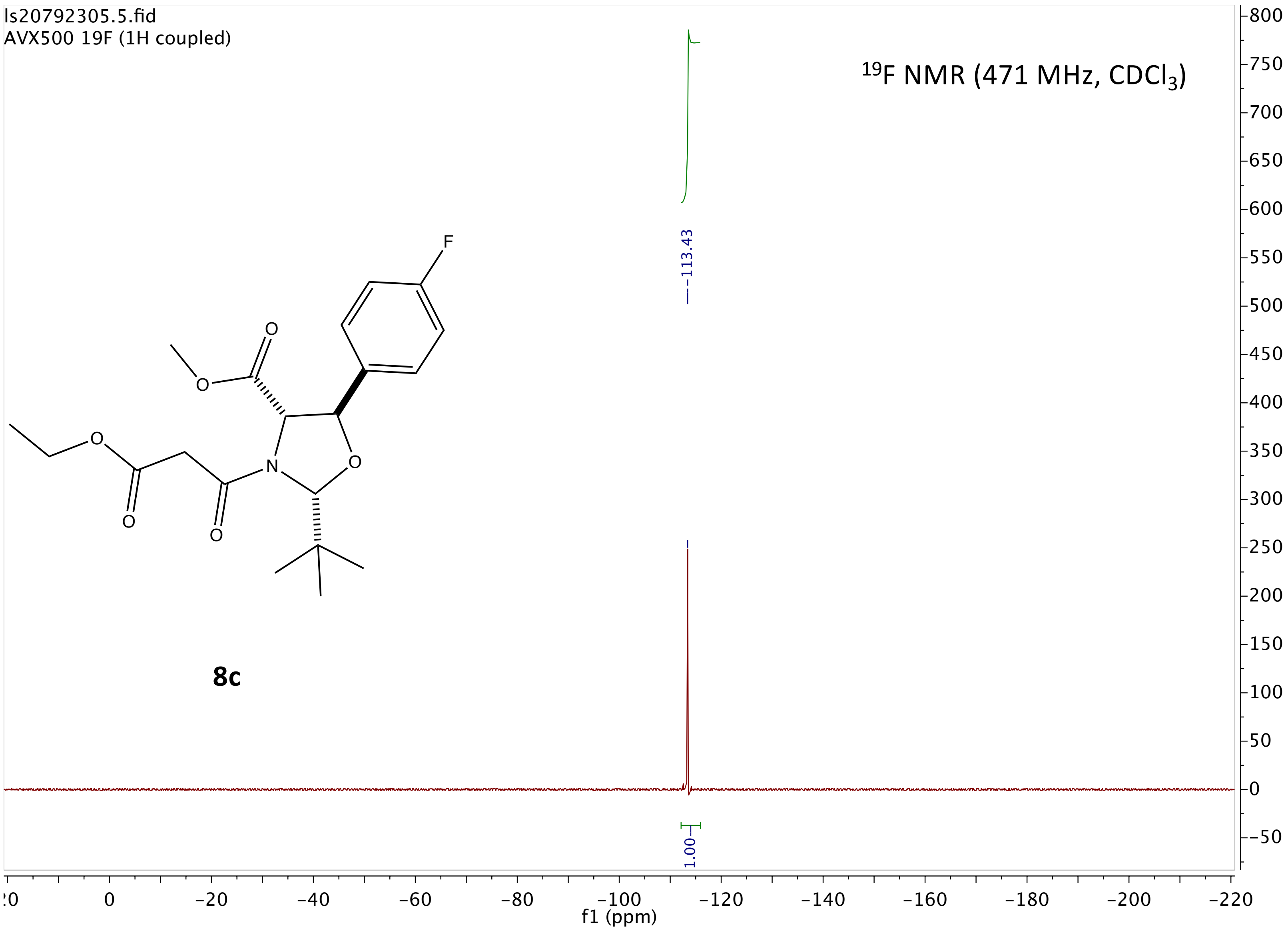
ls20792305.5.fid
AVX500 19F (1H coupled)
19F NMR (471 MHz, CDCl3)
-113.43
1.00
8c
f1 (ppm)

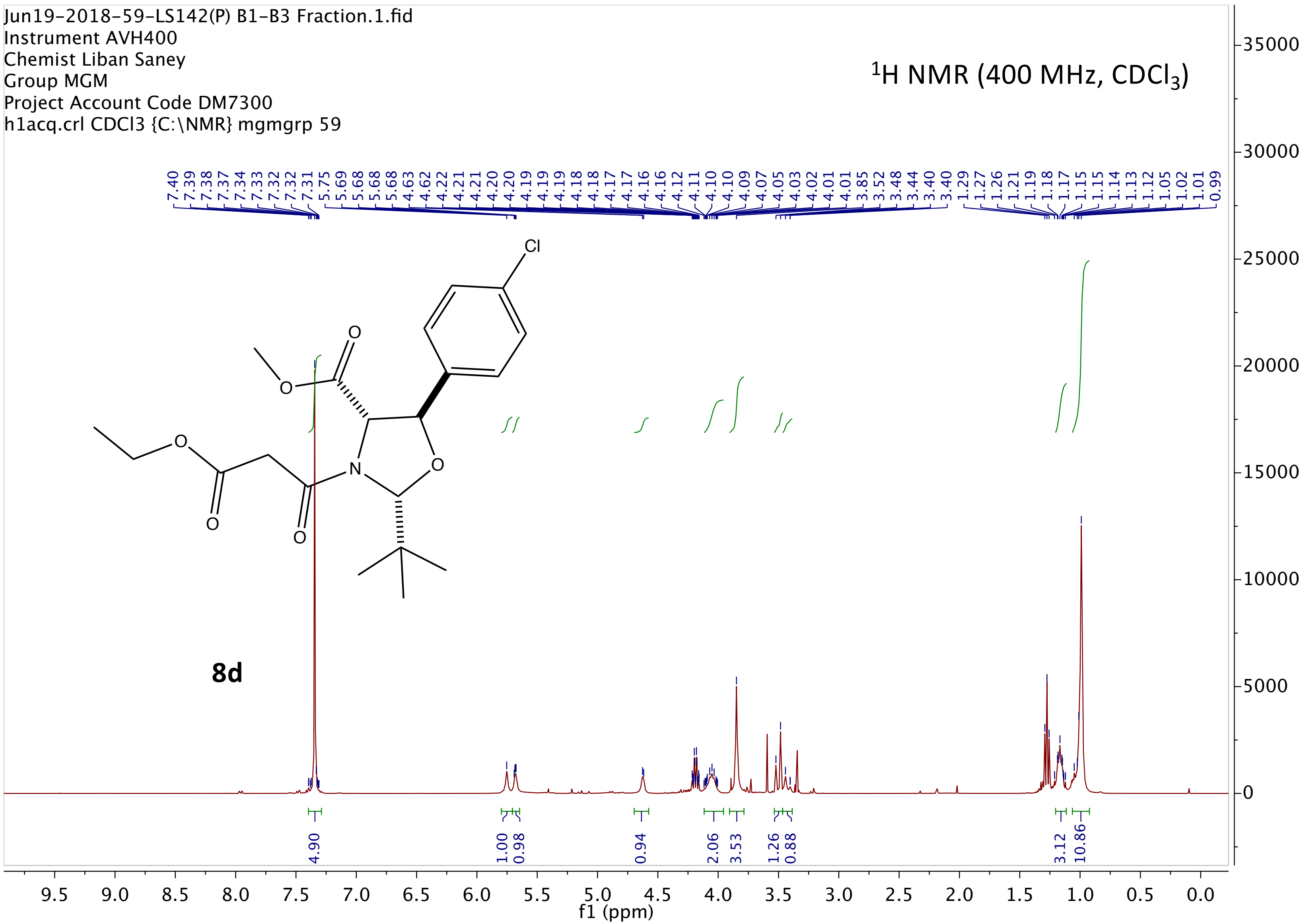

Jun19-2018-59-LS142(P) B1-B3 Fraction.1.fid
Instrument AVH400
Chemist Liban Saney
Group MGM
Project Account Code DM7300
h1acq.crl CDCl3 {C:\NMR} mgmgrp 59
1H NMR (400 MHz, CDCl3)
7.40 7.39 7.38 7.37 7.34 7.33 7.32 7.32 7.31 7.31 5.75 5.69 5.68 5.68 4.63 4.62 4.22 4.21 4.21 4.20 4.20 4.19 4.19 4.19 4.18 4.18 4.17 4.17 4.16 4.16 4.12 4.11 4.10 4.10 4.09 4.07 4.05 4.03 4.02 4.01 4.01 3.85 3.52 3.48 3.44 3.40 3.40 1.29 1.27 1.26 1.21 1.19 1.18 1.17 1.15 1.15 1.14 1.13 1.12 1.05 1.02 1.01 0.99
Cl
O
O
O
O
N
O
O
O
8d
4.90
1.00
0.98
0.94
2.06
3.53
1.26
0.88
3.12
10.86
35000
30000
25000
20000
15000
10000
5000
0
9.5 9.0 8.5 8.0 7.5 7.0 6.5 6.0 5.5 5.0 4.5 4.0 3.5 3.0 2.5 2.0 1.5 1.0 0.5 0.0
f1 (ppm)

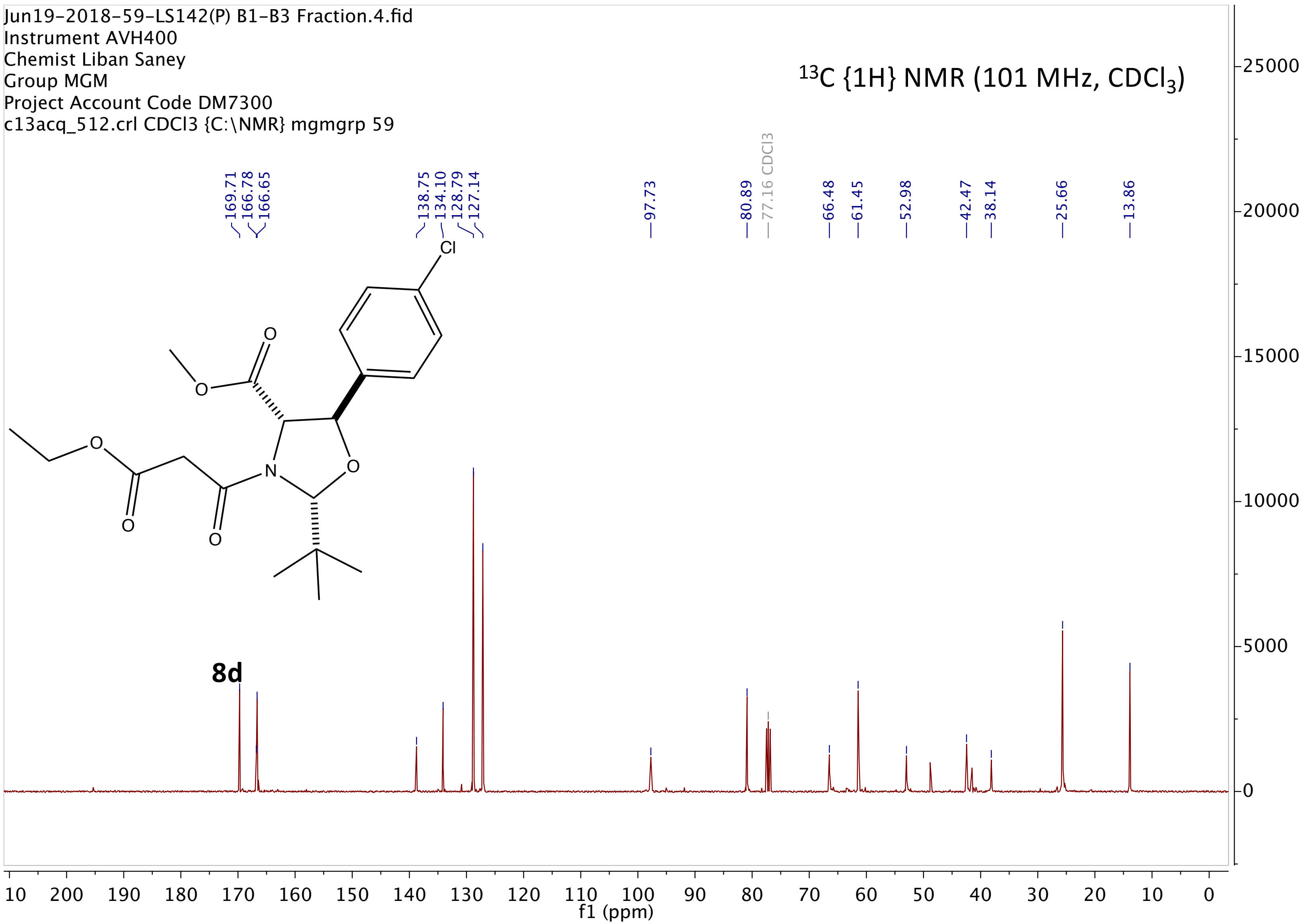
Jun19-2018-59-LS142(P) B1-B3 Fraction.4.fid
Instrument AVH400
Chemist Liban Saney
Group MGM
Project Account Code DM7300
c13acq_512.crl CDCl3 {C:\NMR} mgmgrp 59
13C {1H} NMR (101 MHz, CDCl3)
169.71
166.78
166.65
138.75
134.10
128.79
127.14
97.73
80.89
77.16 CDCl3
66.48
61.45
52.98
42.47
38.14
25.66
13.86
Cl
O
N
8d
f1 (ppm)

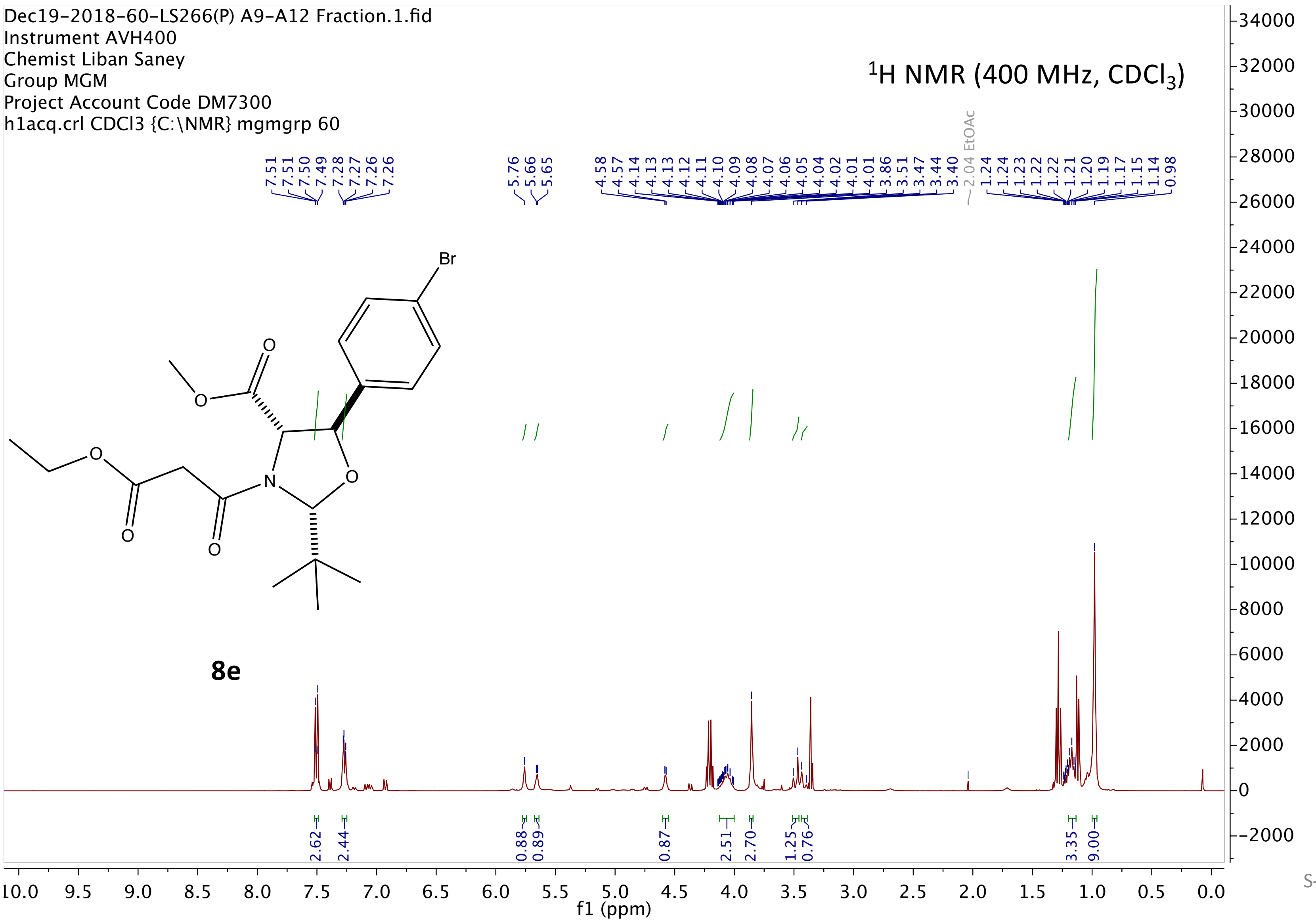

Dec19-2018-60-LS266(P) A9-A12 Fraction.1.fid
Instrument AVH400
Chemist Liban Saney
Group MGM
Project Account Code DM7300
h1acq.crl CDCl3 {C:\NMR} mgmgrp 60
1H NMR (400 MHz, CDCl3)
8e
Br
O
N
2.04 EtOAc
f1 (ppm)

Dec19-2018-60-LS266(P) A9-A12 Fraction.4.fid
Instrument AVH400
Chemist Liban Saney
Group MGM
Project Account Code DM7300
c13acq_512.crl CDCl3 {C:\NMR} mgmgrp 60

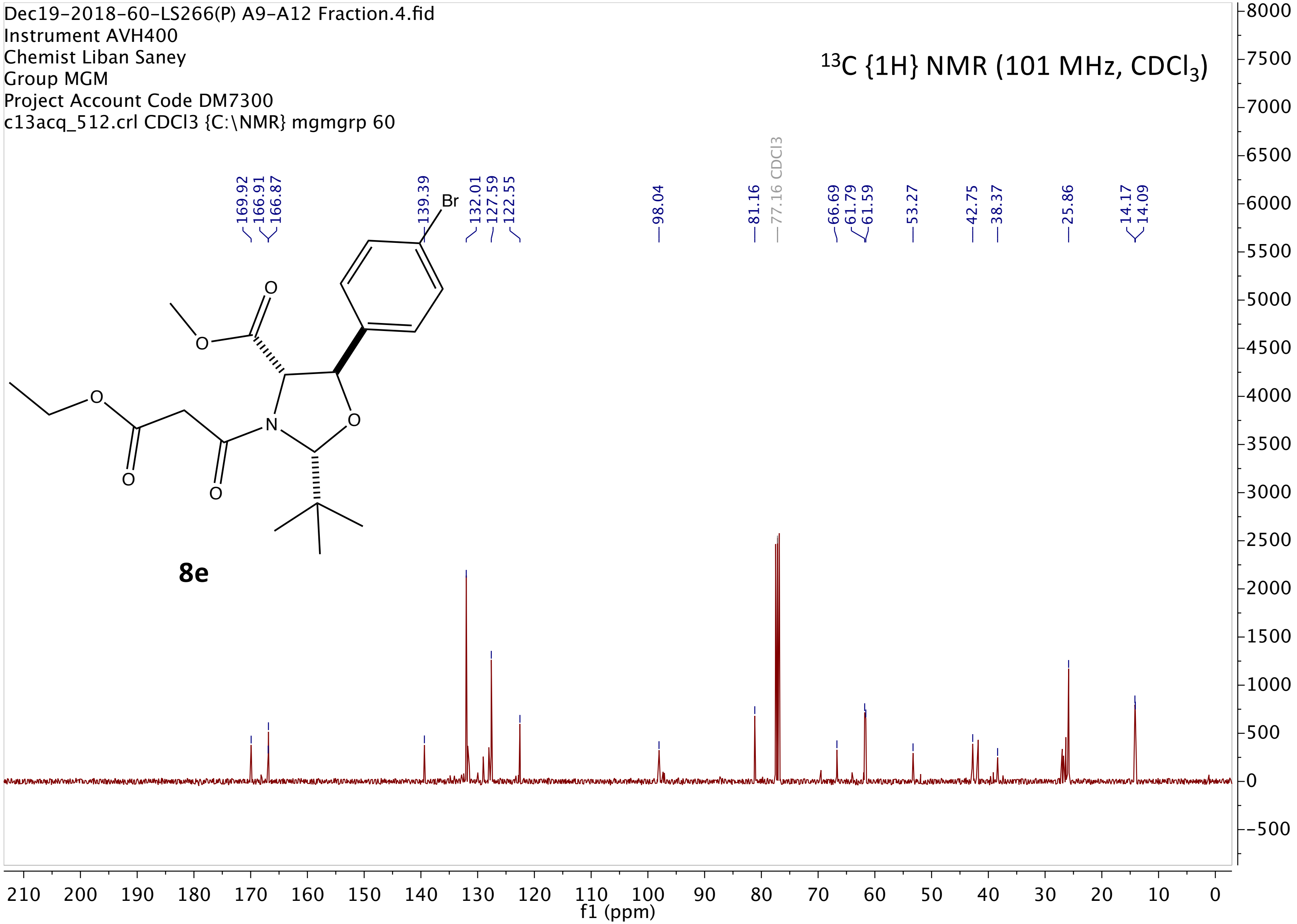

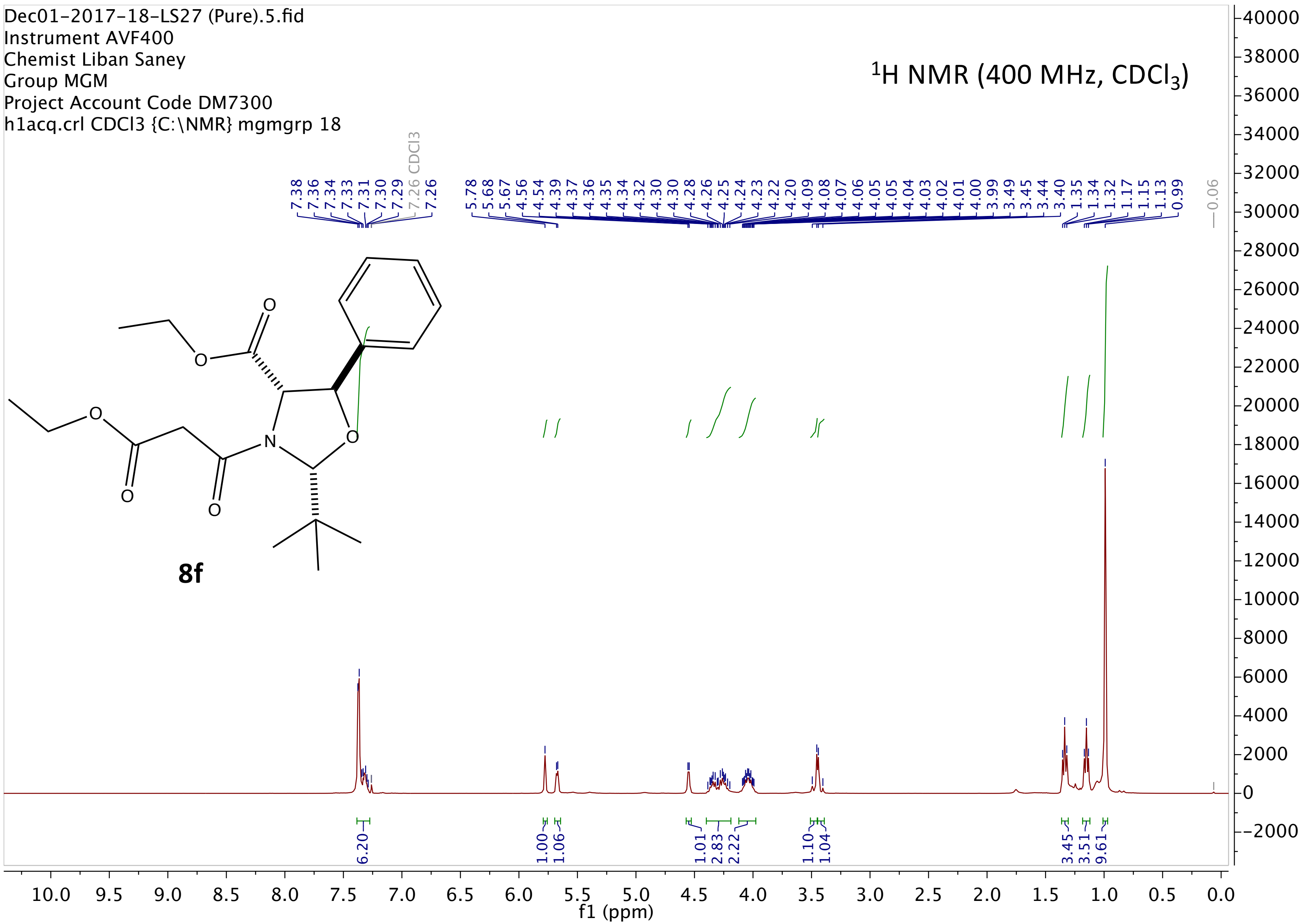
Dec01-2017-18-LS27 (Pure).5.fid
Instrument AVF400
Chemist Liban Saney
Group MGM
Project Account Code DM7300
h1acq.crl CDCl3 {C:\NMR} mgmgrp 18
1H NMR (400 MHz, CDCl3)
7.38 7.36 7.34 7.33 7.31 7.30 7.29 7.26 CDCl3 7.26
5.78 5.68 5.67 4.56 4.54 4.39 4.37 4.36 4.35 4.34 4.32 4.30 4.30 4.28 4.26 4.25 4.24 4.23 4.22 4.20 4.09 4.08 4.07 4.06 4.05 4.04 4.03 4.02 4.01 4.00 3.99 3.49 3.45 3.44 3.40 1.35 1.34 1.32 1.17 1.15 1.13 0.99
0.06
O O N O O O O
8f
6.20 1.00 1.06 1.01 2.83 2.22 1.10 1.04 3.45 3.51 9.61
f1 (ppm)

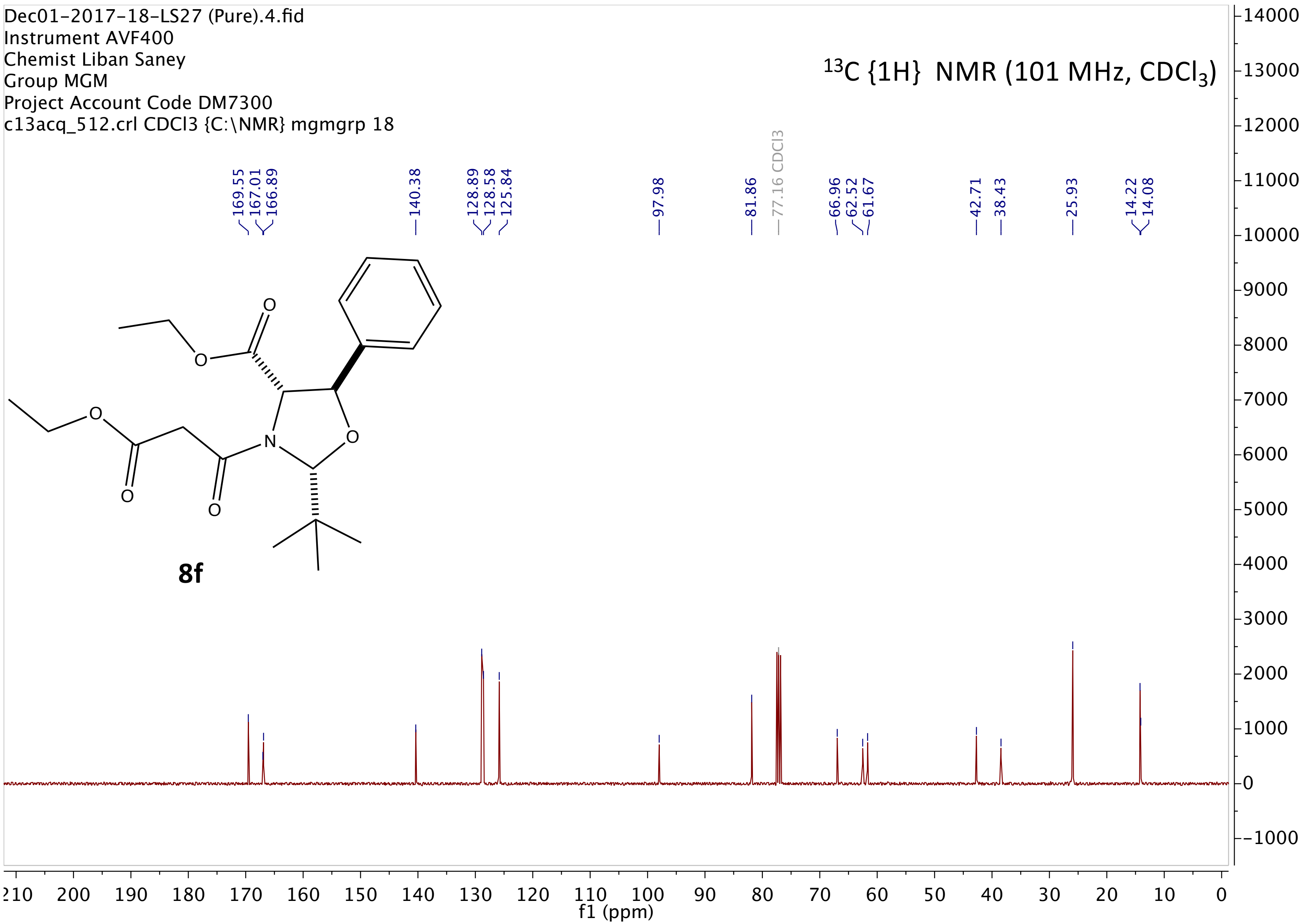
Dec01-2017-18-LS27 (Pure).4.fid
Instrument AVF400
Chemist Liban Saney
Group MGM
Project Account Code DM7300
c13acq_512.crl CDCl3 {C:\NMR} mgmgrp 18
^{13}C {1H} NMR (101 MHz, CDCl$_3$)
169.55
167.01
166.89
140.38
128.89
128.58
125.84
97.98
81.86
77.16 CDCl3
66.96
62.52
61.67
42.71
38.43
25.93
14.22
14.08
O
O
O
N
O
O
O
8f
14000
13000
12000
11000
10000
9000
8000
7000
6000
5000
4000
3000
2000
1000
0
-1000
210 200 190 180 170 160 150 140 130 120 110 100 90 80 70 60 50 40 30 20 10 0
f1 (ppm)

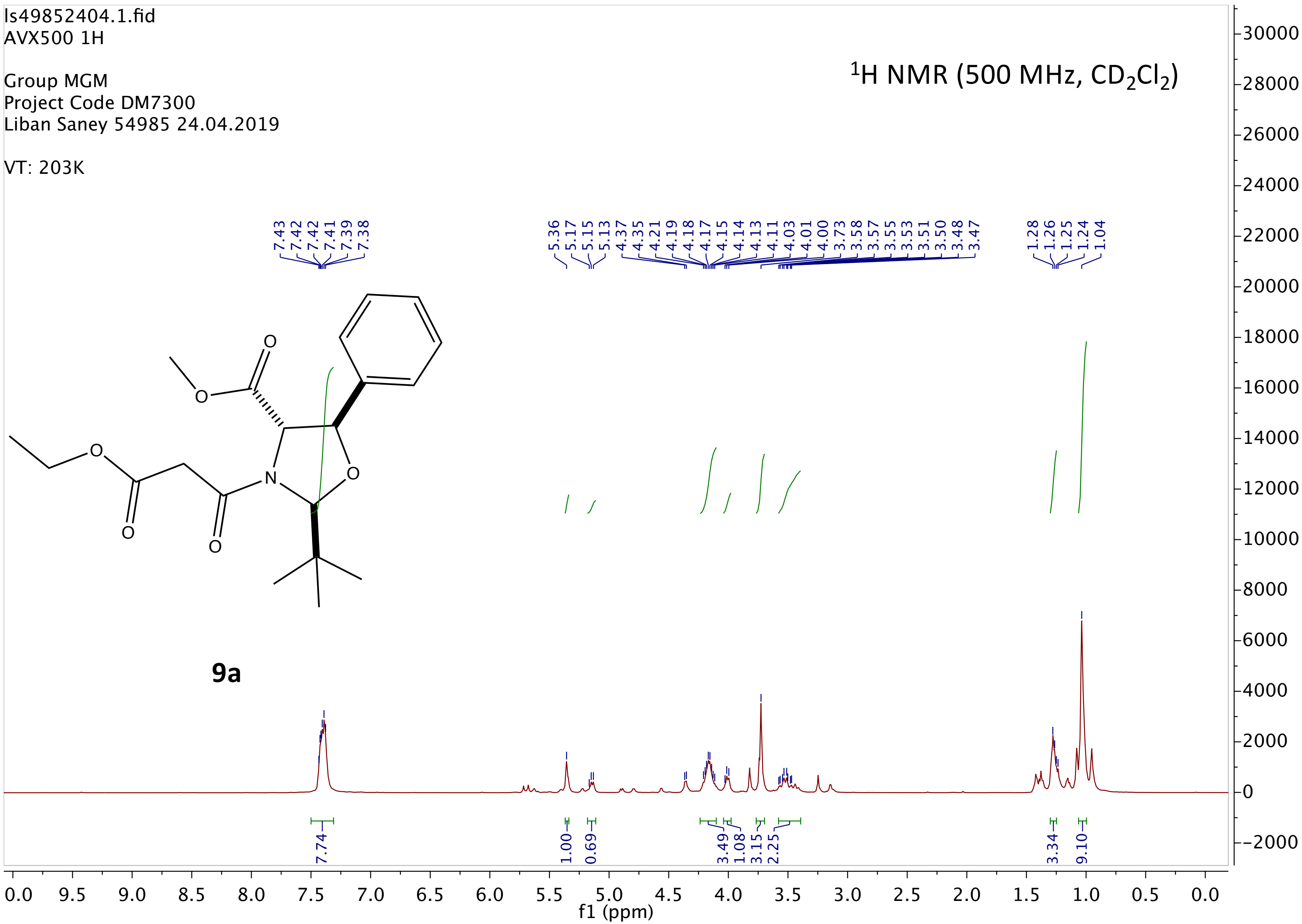
ls49852404.1.fid
AVX500 1H
Group MGM
Project Code DM7300
Liban Saney 54985 24.04.2019
VT: 203K
^{1}H NMR (500 MHz, CD_2Cl_2)
9a
7.43 7.42 7.42 7.41 7.39 7.38
5.36 5.17 5.15 5.13 4.37 4.35 4.21 4.19 4.18 4.17 4.15 4.14 4.13 4.11 4.03 4.01 4.00 3.73 3.58 3.57 3.55 3.53 3.51 3.50 3.48 3.47
1.28 1.26 1.25 1.24 1.04
7.74 1.00 0.69 3.49 1.08 3.15 2.25 3.34 9.10
f1 (ppm)

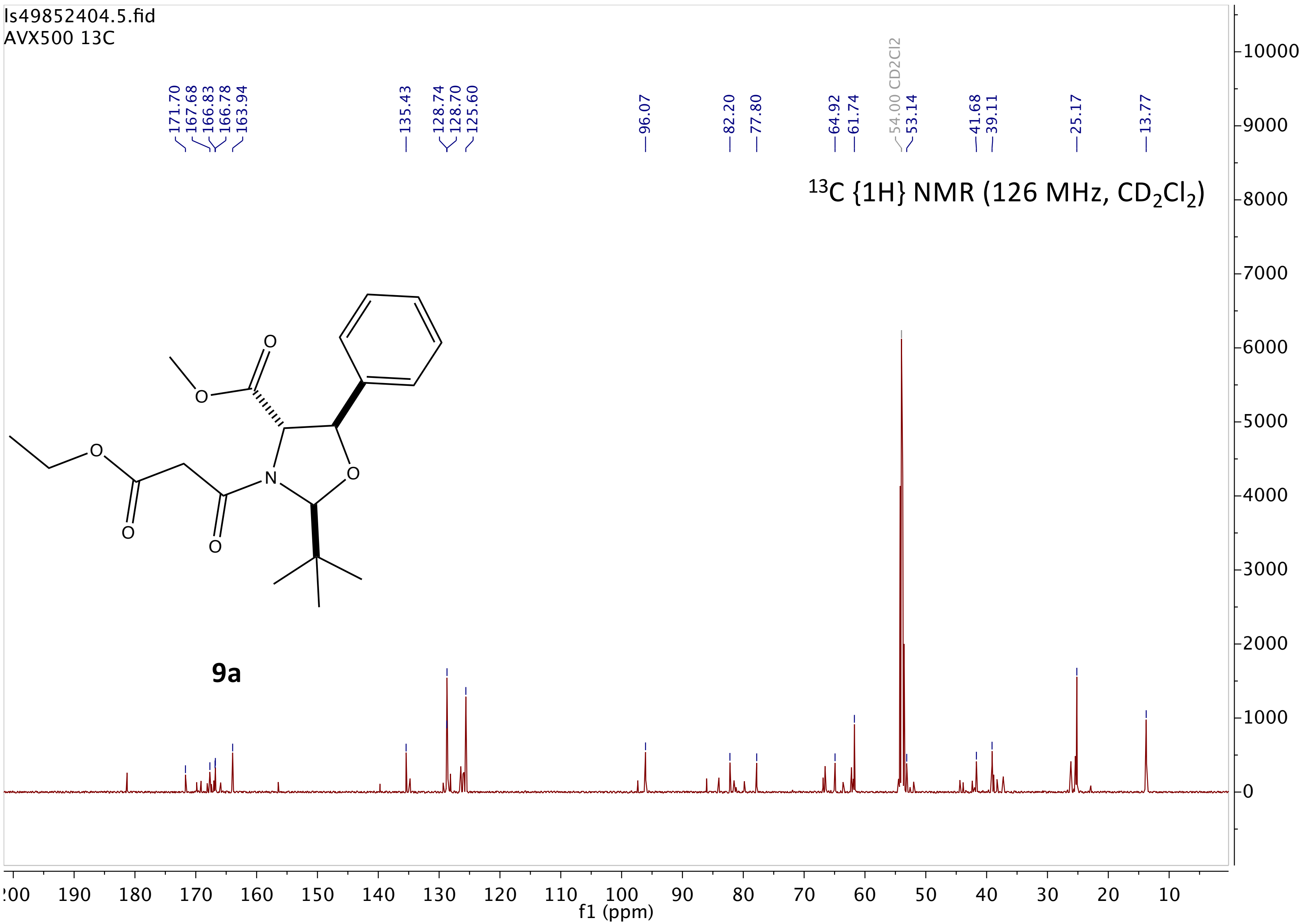
ls49852404.5.fid
AVX500 13C
171.70
167.68
166.83
166.78
163.94
135.43
128.74
128.70
125.60
96.07
82.20
77.80
64.92
61.74
54.00 CD2Cl2
53.14
41.68
39.11
25.17
13.77
^{13}C {1H} NMR (126 MHz, CD_2Cl_2)
9a
f1 (ppm)

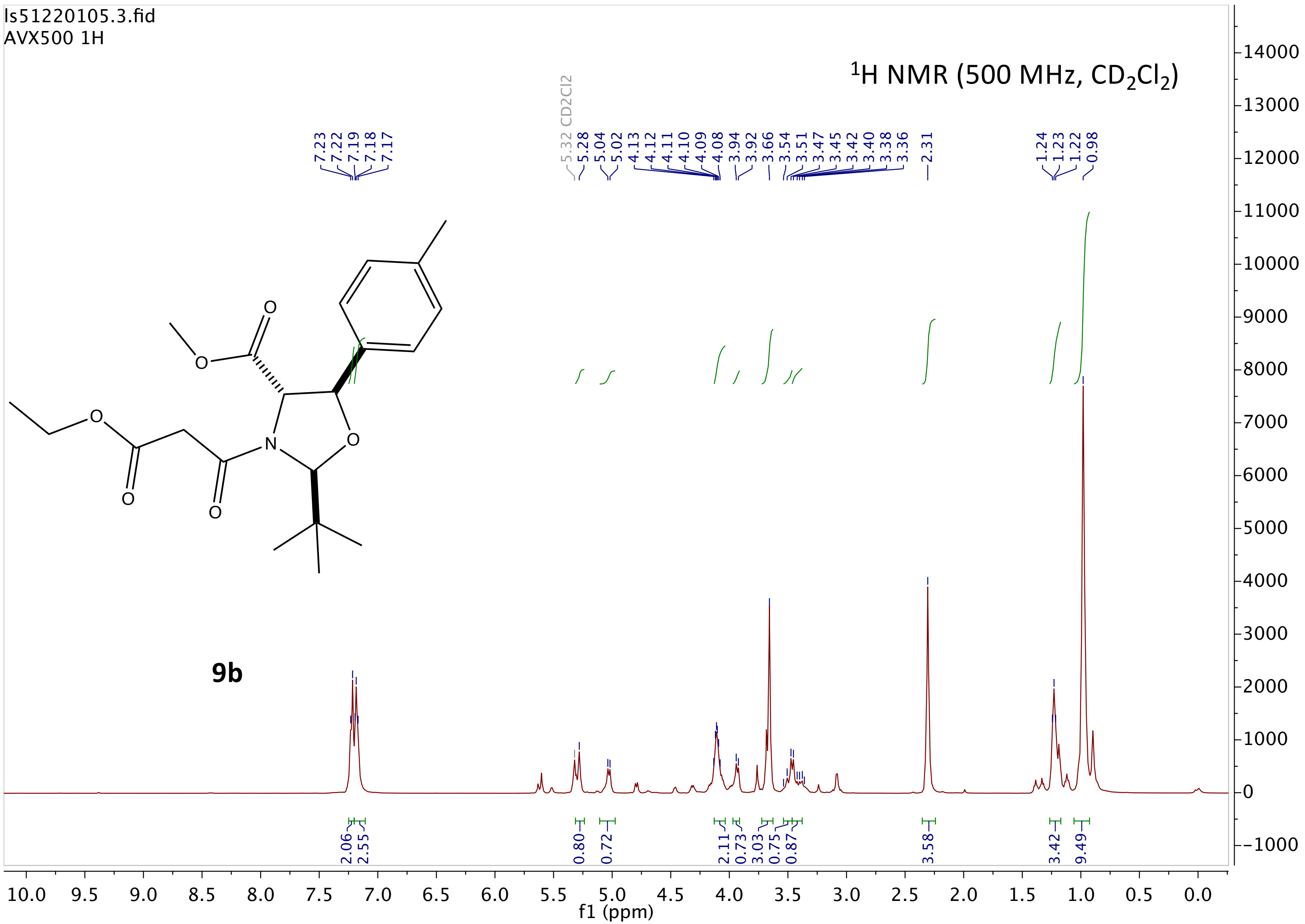
ls51220105.3.fid
AVX500 1H
1H NMR (500 MHz, CD2Cl2)
7.23 7.22 7.19 7.18 7.17
5.32 CD2Cl2
5.28 5.04 5.02 4.13 4.12 4.11 4.10 4.09 4.08 3.94 3.92 3.66 3.54 3.51 3.47 3.45 3.42 3.40 3.38 3.36 2.31
1.24 1.23 1.22 0.98
9b
2.06 2.55 0.80 0.72 2.11 0.73 3.03 0.75 0.87 3.58 3.42 9.49
f1 (ppm)

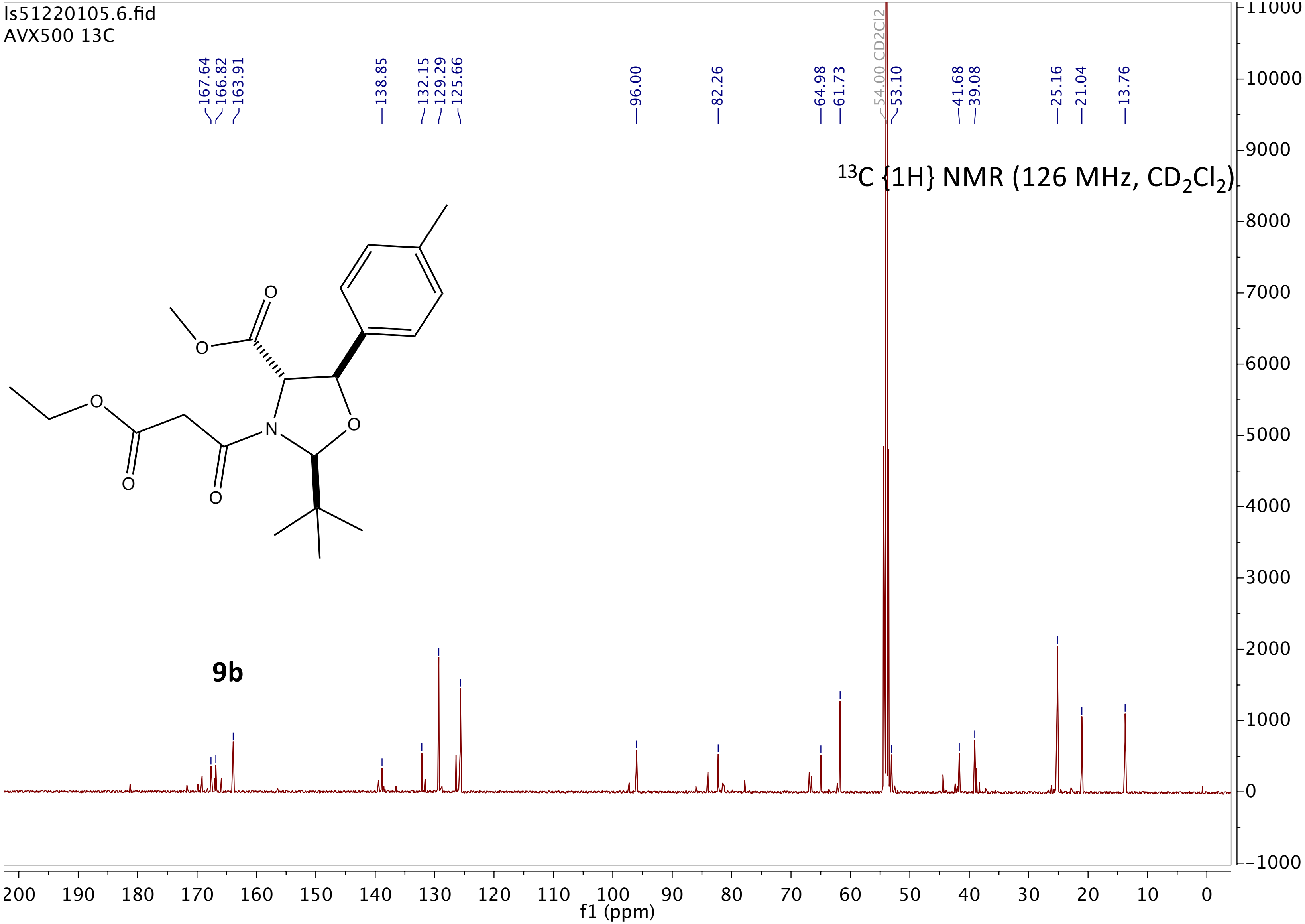
ls51220105.6.fid
AVX500 13C
167.64
166.82
163.91
138.85
132.15
129.29
125.66
96.00
82.26
64.98
61.73
54.00 CD2Cl2
53.10
41.68
39.08
25.16
21.04
13.76
^{13}C {1H} NMR (126 MHz, CD_2Cl_2)
9b
f1 (ppm)

ls45091403.4.fid
Group MGM
Project Code DM7300
Liban Saney 54509 14.03.2019

VT: 203K

^1H NMR (500 MHz, CD_2Cl_2)

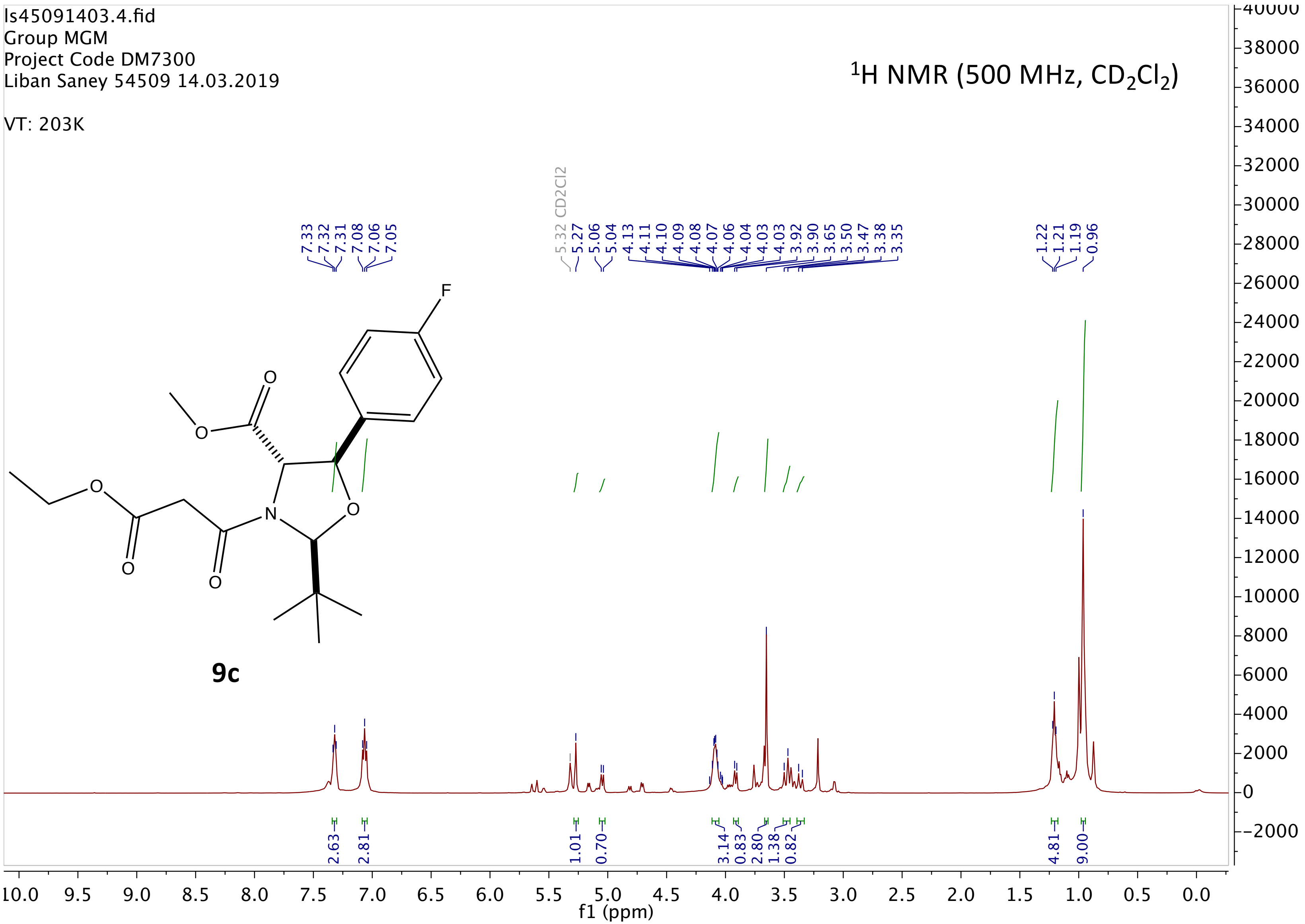

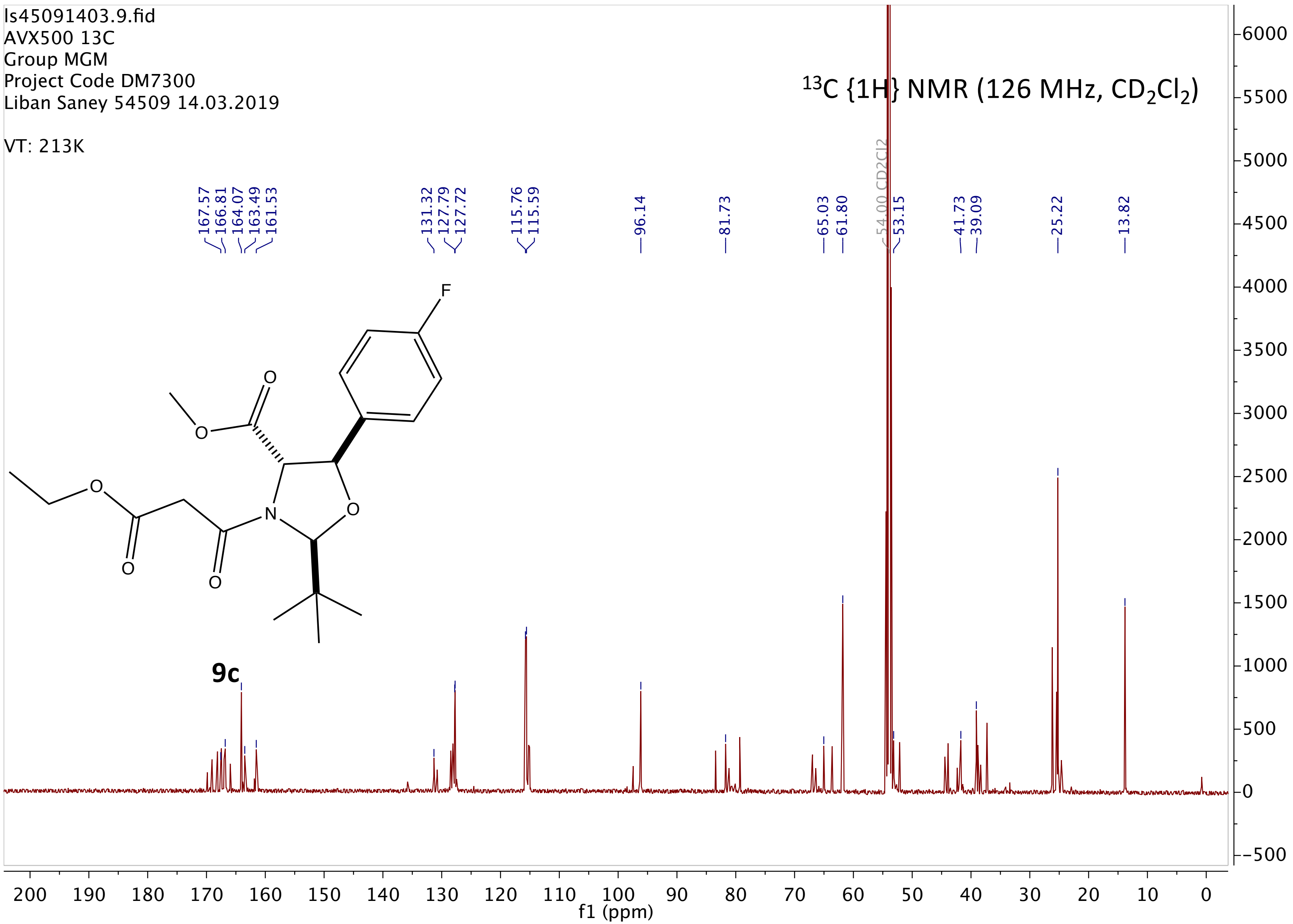
ls45091403.9.fid
AVX500 13C
Group MGM
Project Code DM7300
Liban Saney 54509 14.03.2019
VT: 213K
13C {1H} NMR (126 MHz, CD2Cl2)
167.57
166.81
164.07
163.49
161.53
131.32
127.79
127.72
115.76
115.59
96.14
81.73
65.03
61.80
54.00 CD2Cl2
53.15
41.73
39.09
25.22
13.82
F
O
N
9c
f1 (ppm)

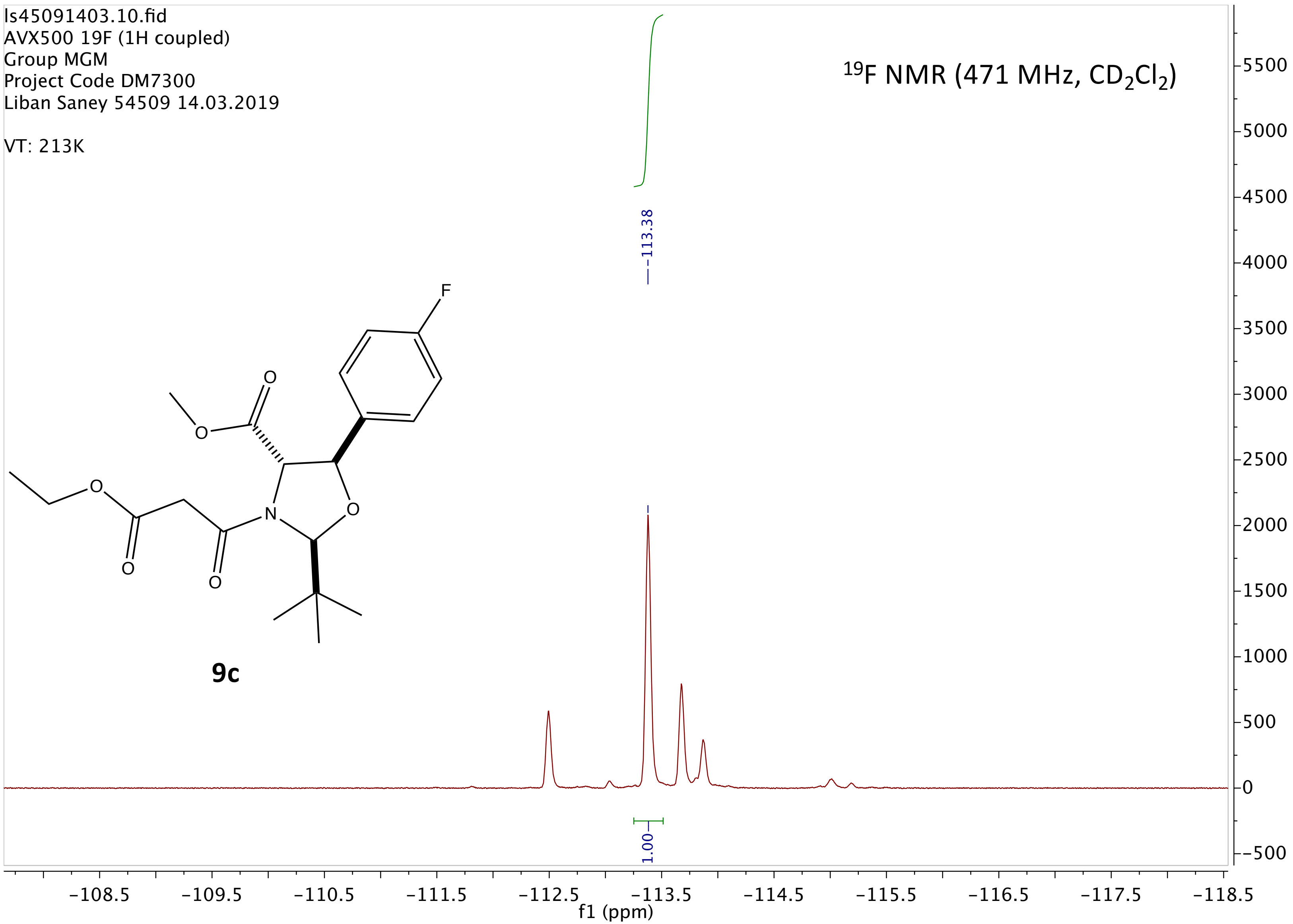
ls45091403.10.fid
AVX500 19F (1H coupled)
Group MGM
Project Code DM7300
Liban Saney 54509 14.03.2019
VT: 213K
19F NMR (471 MHz, CD2Cl2)
9c
-113.38
1.00
f1 (ppm)

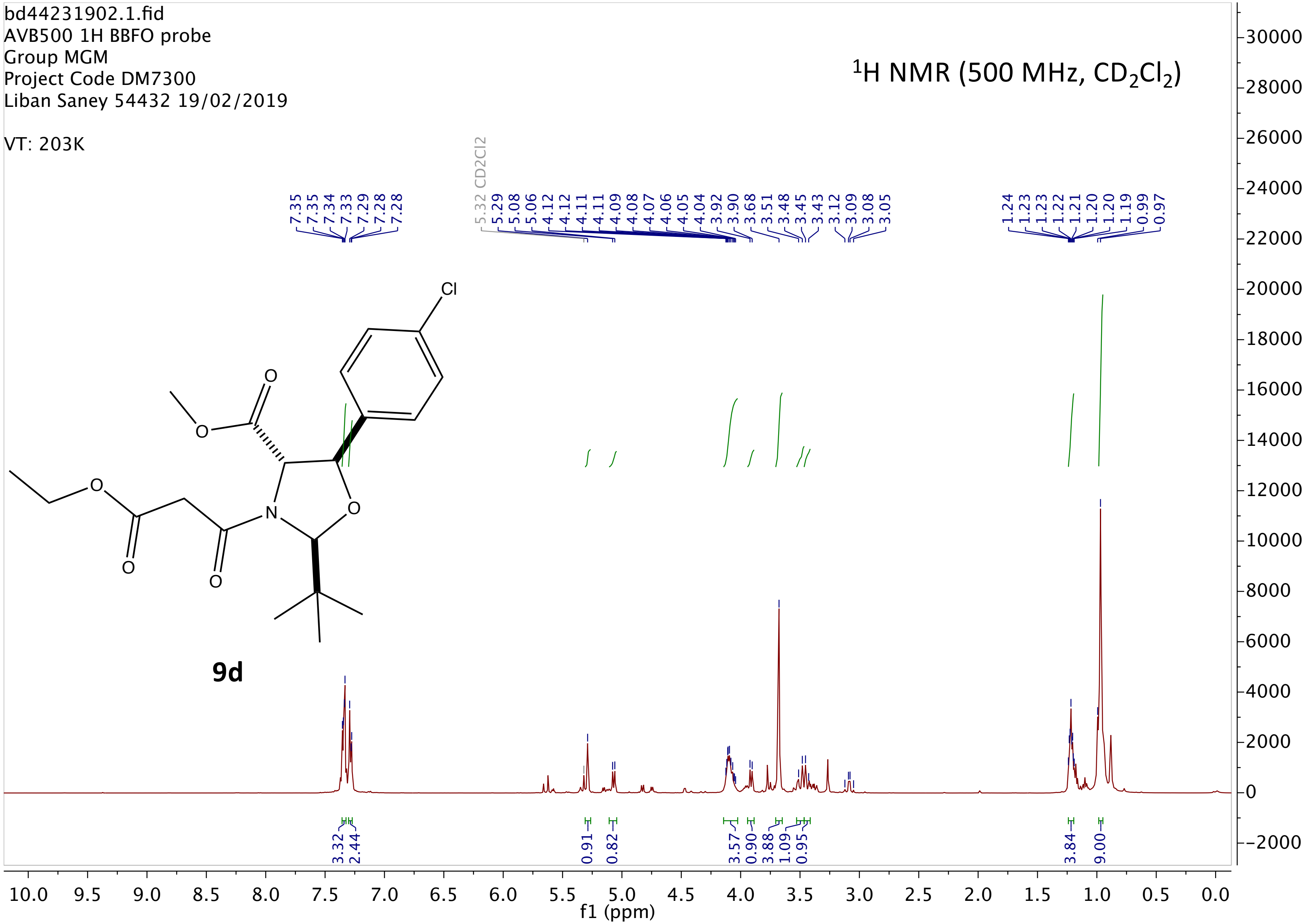

bd44231902.1.fid
AVB500 1H BBFO probe
Group MGM
Project Code DM7300
Liban Saney 54432 19/02/2019
VT: 203K
1H NMR (500 MHz, CD2Cl2)
9d
f1 (ppm)

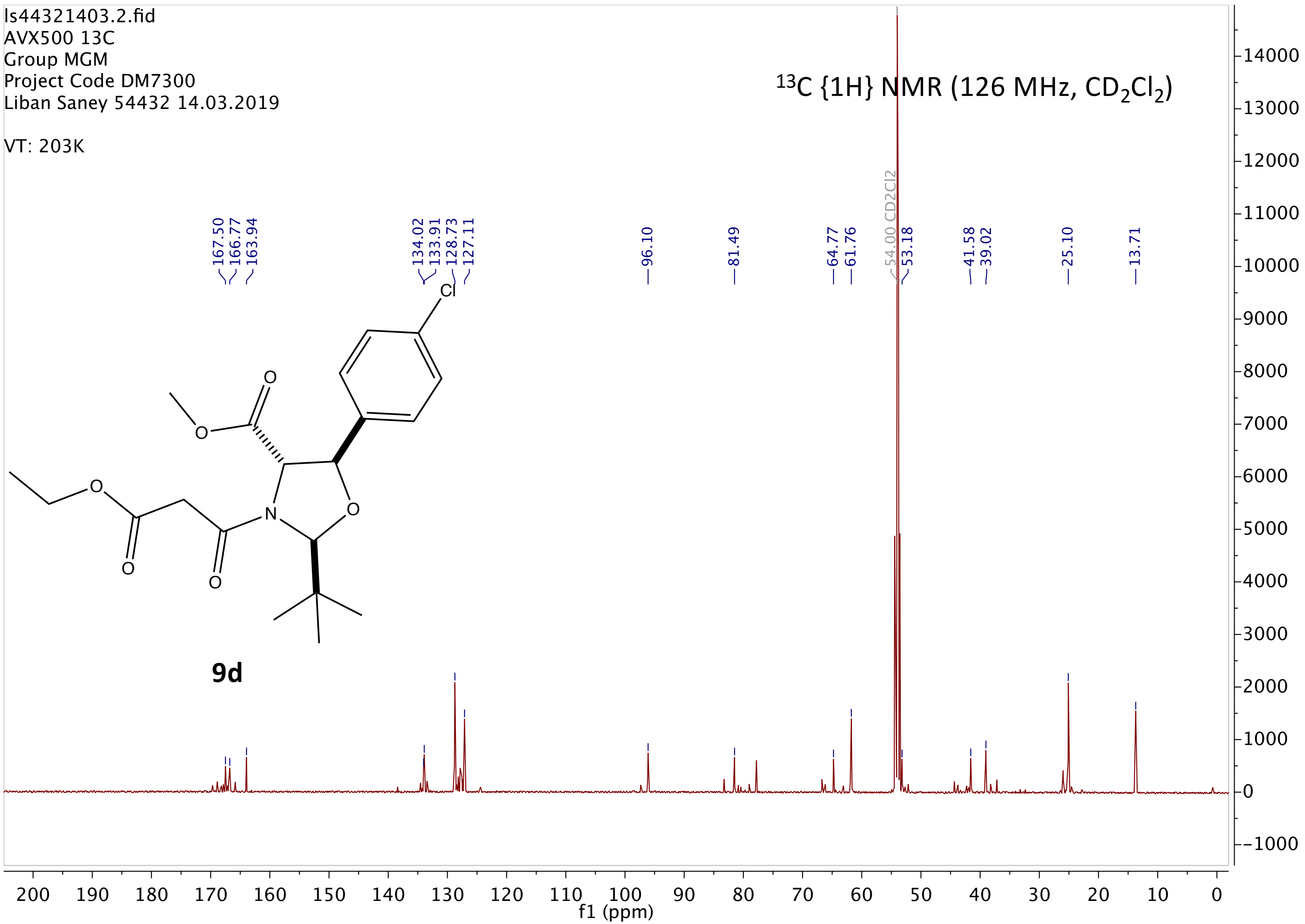

ls44321403.2.fid
AVX500 13C
Group MGM
Project Code DM7300
Liban Saney 54432 14.03.2019
VT: 203K
13C {1H} NMR (126 MHz, CD2Cl2)
167.50
166.77
163.94
134.02
133.91
128.73
127.11
96.10
81.49
64.77
61.76
54.00 CD2Cl2
53.18
41.58
39.02
25.10
13.71
Cl
O
N
9d
f1 (ppm)

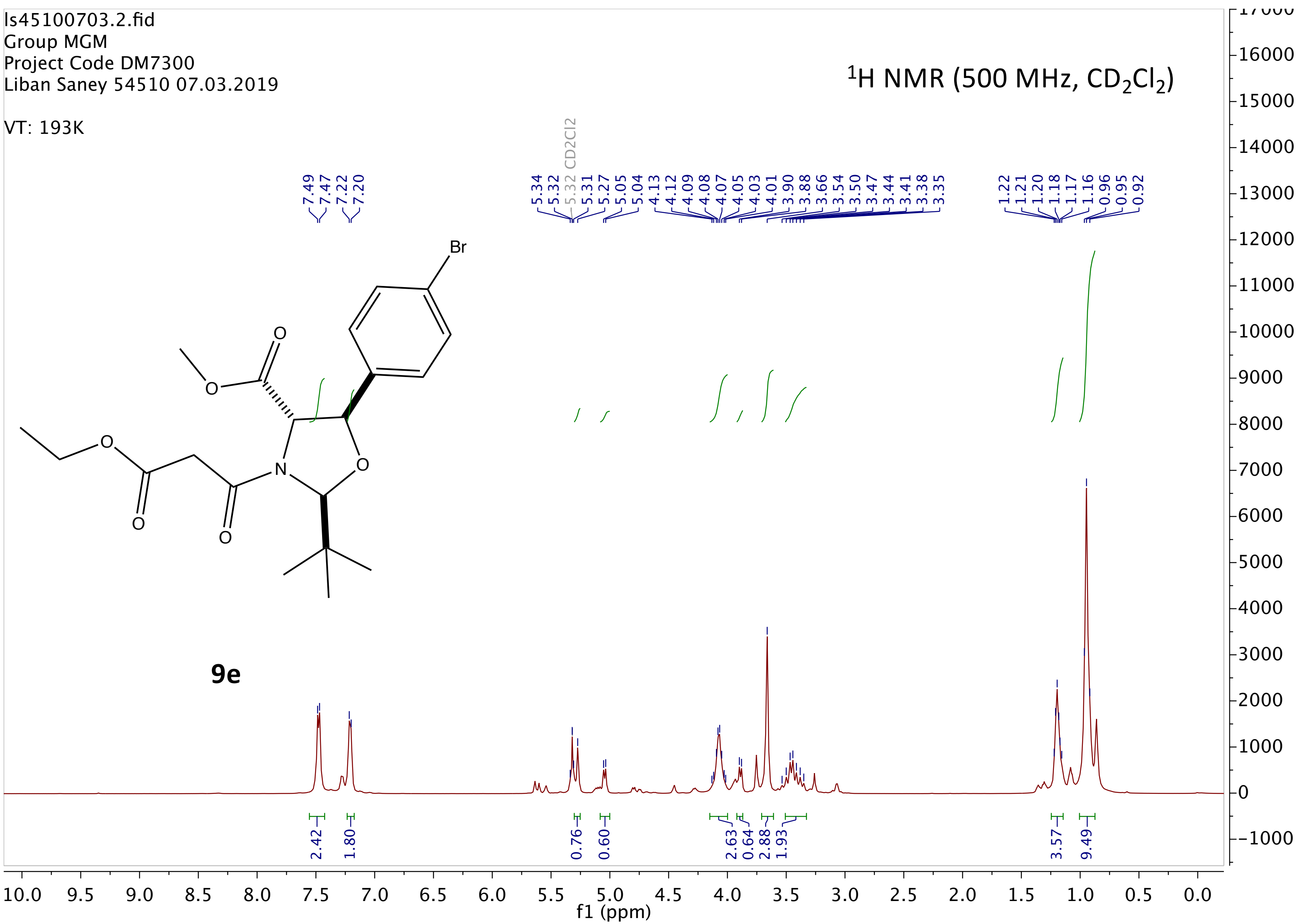
ls45100703.2.fid
Group MGM
Project Code DM7300
Liban Saney 54510 07.03.2019
VT: 193K
^{1}H NMR (500 MHz, CD_2Cl_2)
9e
f1 (ppm)

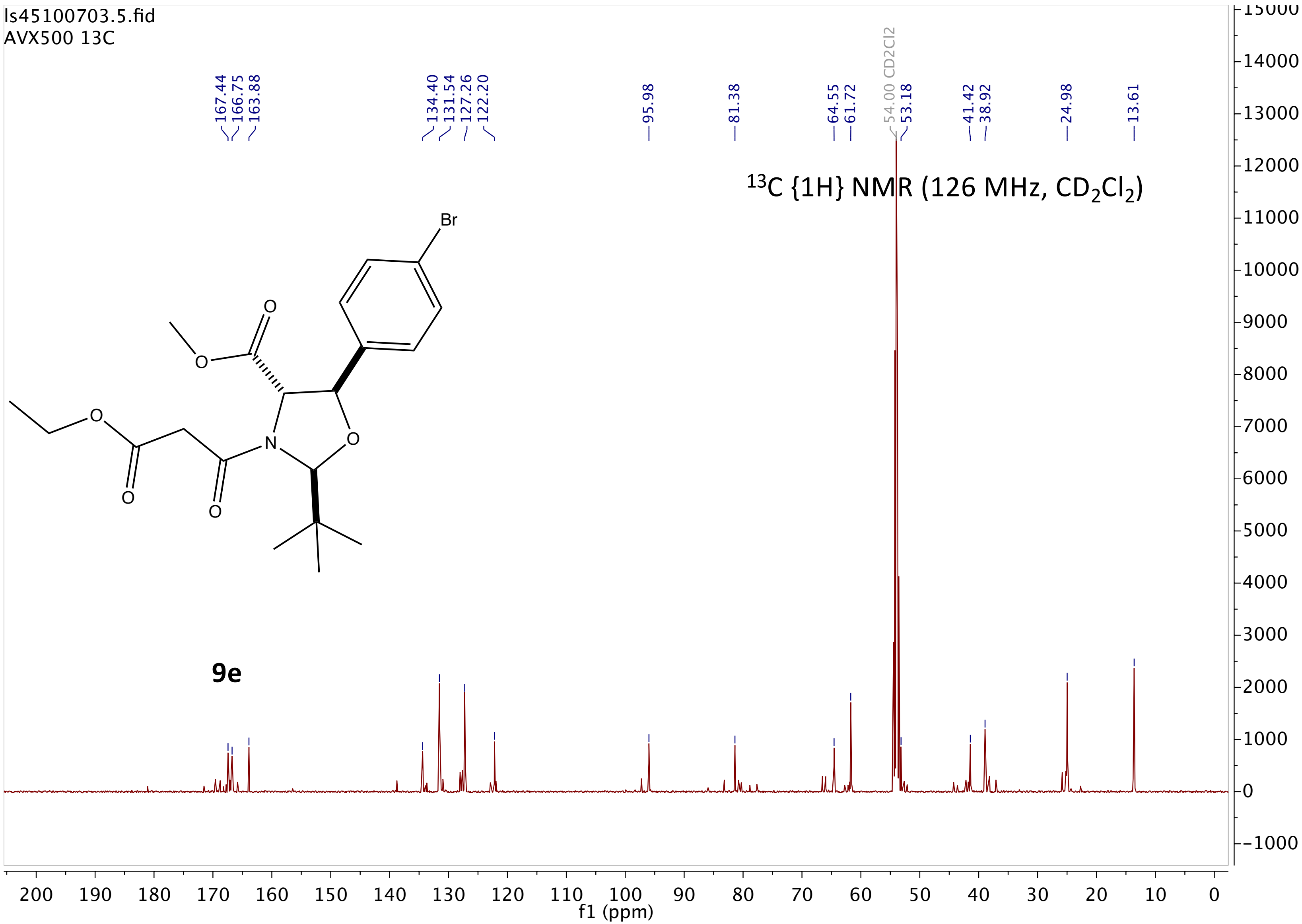

ls45100703.5.fid
AVX500 13C
167.44
166.75
163.88
134.40
131.54
127.26
122.20
95.98
81.38
64.55
61.72
54.00 CD2Cl2
53.18
41.42
38.92
24.98
13.61
^{13}C {1H} NMR (126 MHz, CD_2Cl_2)
Br
O
N
9e
f1 (ppm)

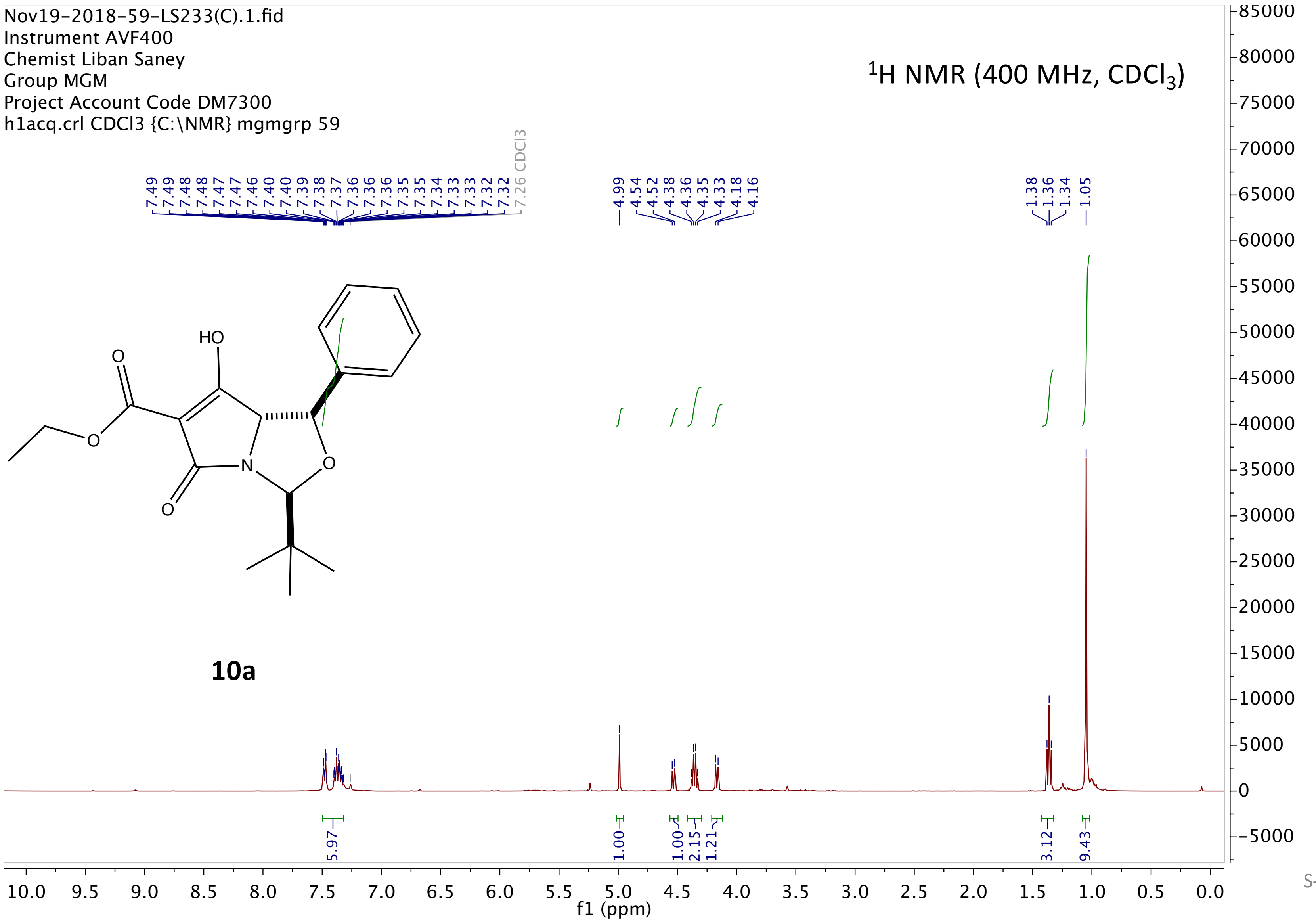
Nov19-2018-59-LS233(C).1.fid
Instrument AVF400
Chemist Liban Saney
Group MGM
Project Account Code DM7300
h1acq.crl CDCl3 {C:\NMR} mgmgrp 59
¹H NMR (400 MHz, CDCl3)
7.49 7.49 7.48 7.48 7.47 7.47 7.46 7.40 7.40 7.39 7.38 7.37 7.36 7.36 7.35 7.35 7.34 7.33 7.33 7.32 7.32
7.26 CDCl3
4.99 4.54 4.52 4.38 4.36 4.35 4.33 4.18 4.16
1.38 1.36 1.34 1.05
HO
O
O
N
O
O
10a
5.97 1.00 1.00 2.15 1.21 3.12 9.43
f1 (ppm)

Nov19-2018-59-LS233(C).4.fid
Instrument AVF400
Chemist Liban Saney
Group MGM
Project Account Code DM7300
c13acq_512.crl CDCl3 {C:\NMR} mgmgrp 59

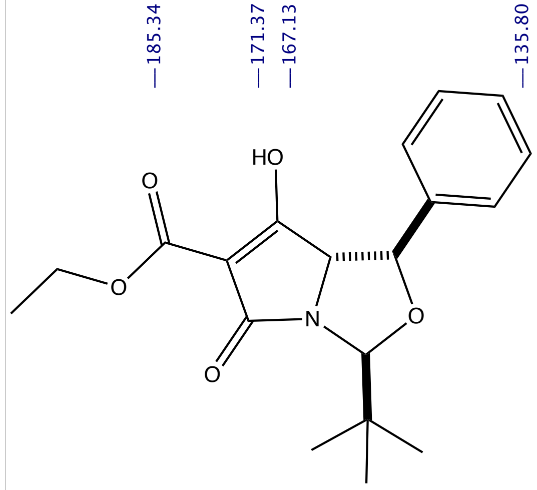

10a

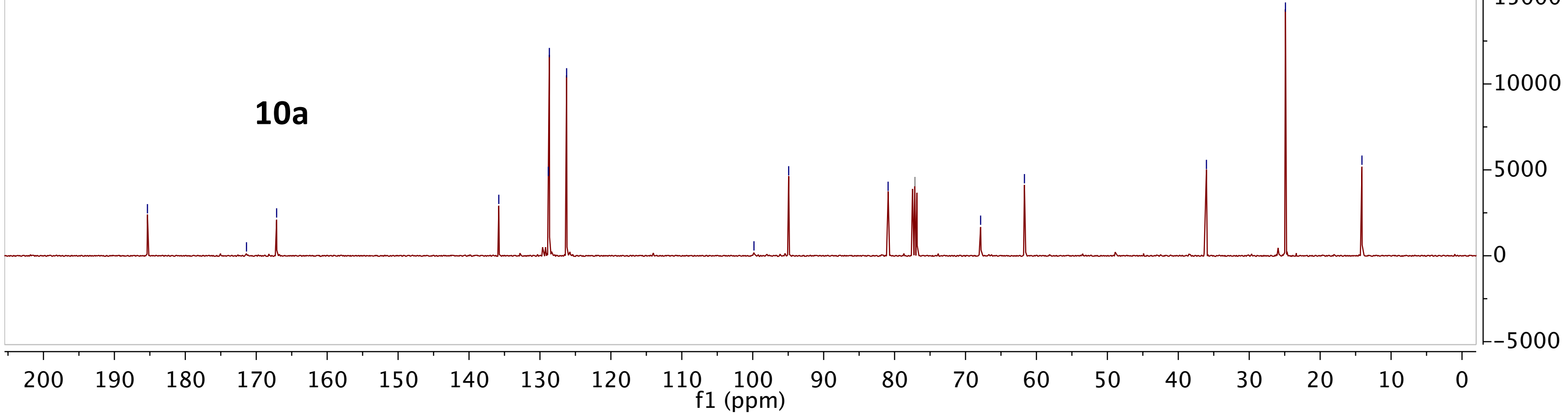

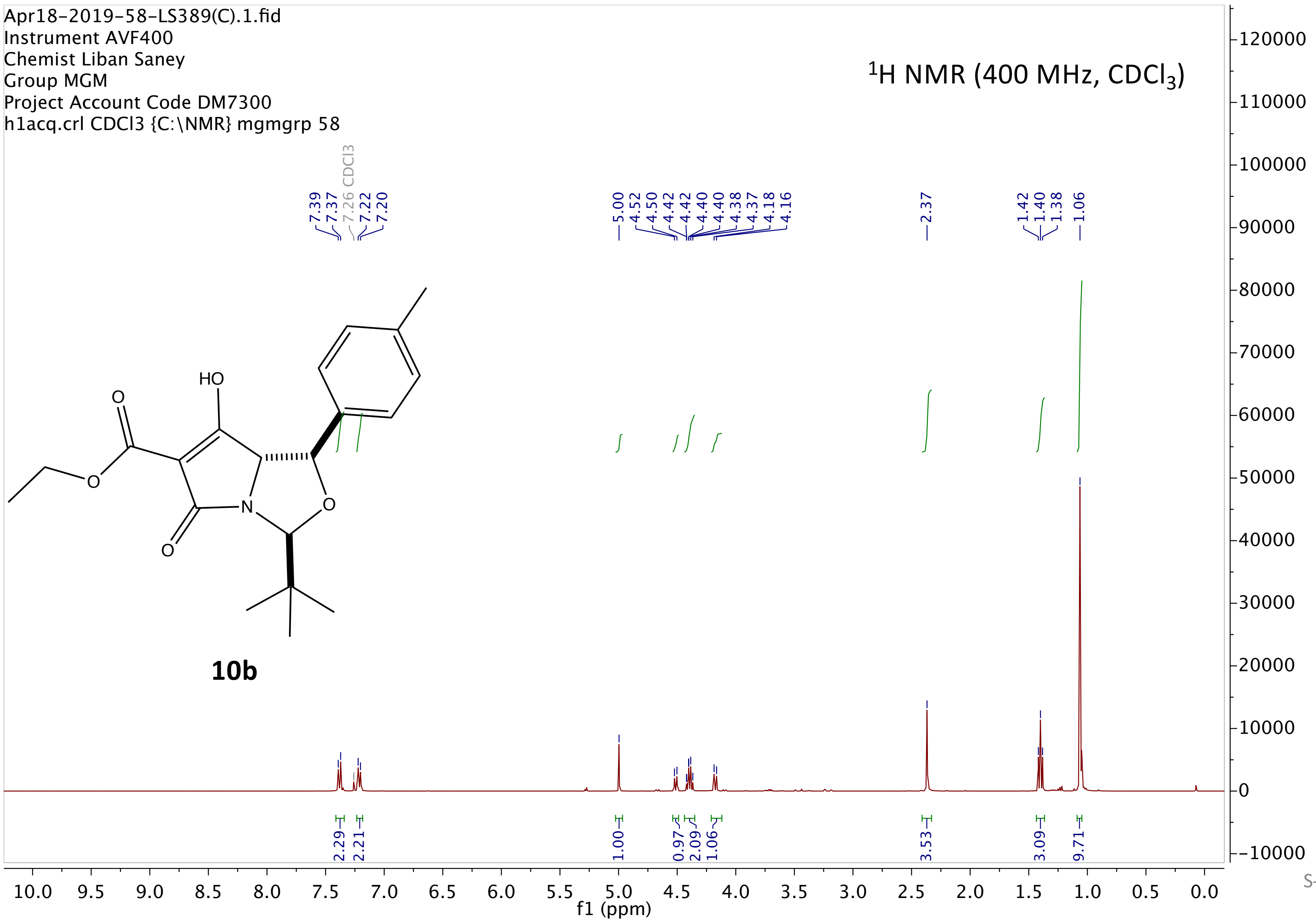
Apr18-2019-58-LS389(C).1.fid
Instrument AVF400
Chemist Liban Saney
Group MGM
Project Account Code DM7300
h1acq.crl CDCl3 {C:\NMR} mgmgrp 58
1H NMR (400 MHz, CDCl3)
7.39
7.37
7.26 CDCl3
7.22
7.20
5.00
4.52
4.50
4.42
4.42
4.40
4.40
4.38
4.37
4.18
4.16
2.37
1.42
1.40
1.38
1.06
HO
O
O
O
N
O
O
10b
2.29
2.21
1.00
0.97
2.09
1.06
3.53
3.09
9.71
f1 (ppm)

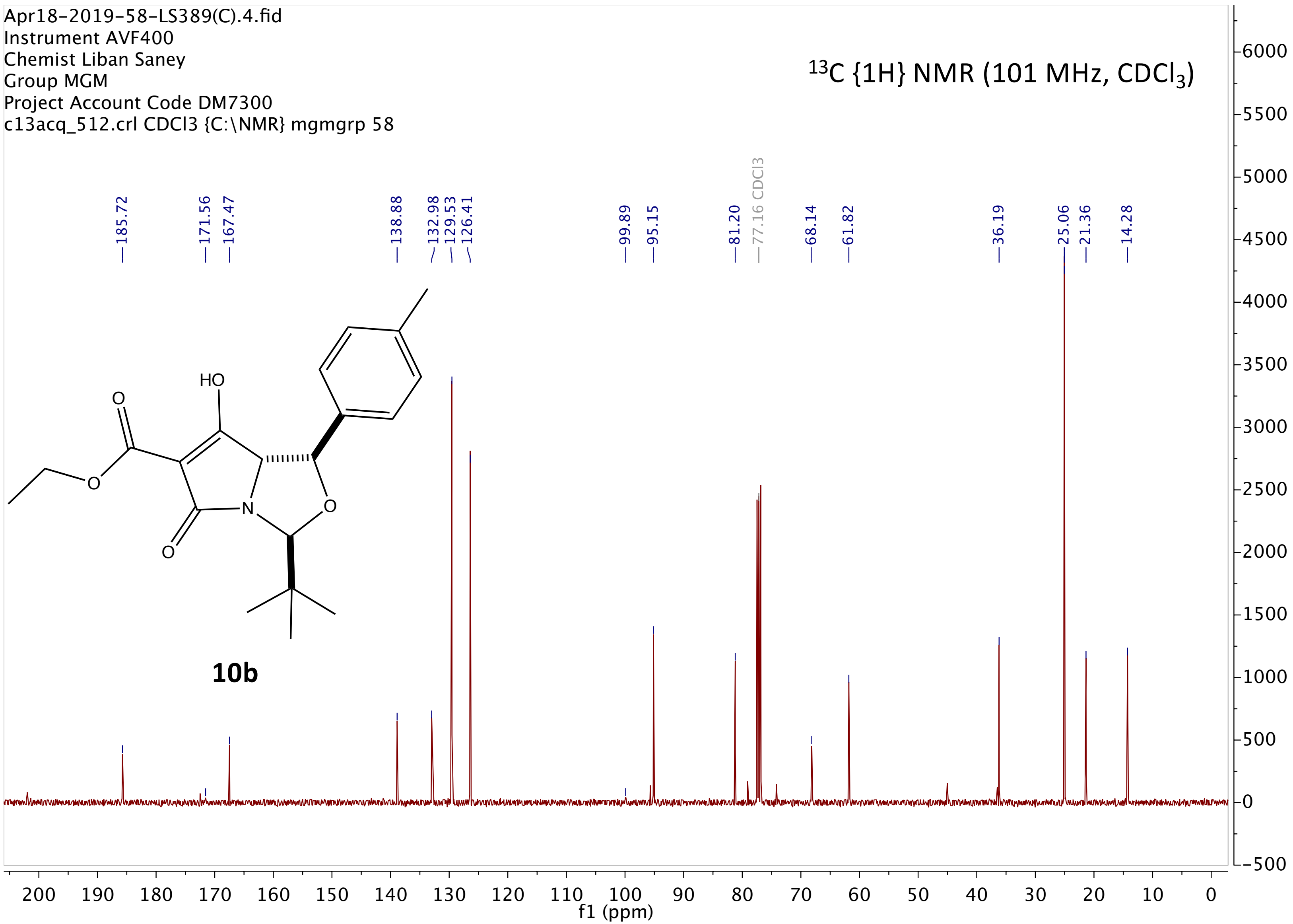
Apr18-2019-58-LS389(C).4.fid
Instrument AVF400
Chemist Liban Saney
Group MGM
Project Account Code DM7300
c13acq_512.crl CDCl3 {C:\NMR} mgmgrp 58
^{13}C {1H} NMR (101 MHz, $CDCl_3$)
185.72
171.56
167.47
138.88
132.98
129.53
126.41
99.89
95.15
81.20
77.16 CDCl3
68.14
61.82
36.19
25.06
21.36
14.28
HO
O
O
O
N
O
O
10b
f1 (ppm)

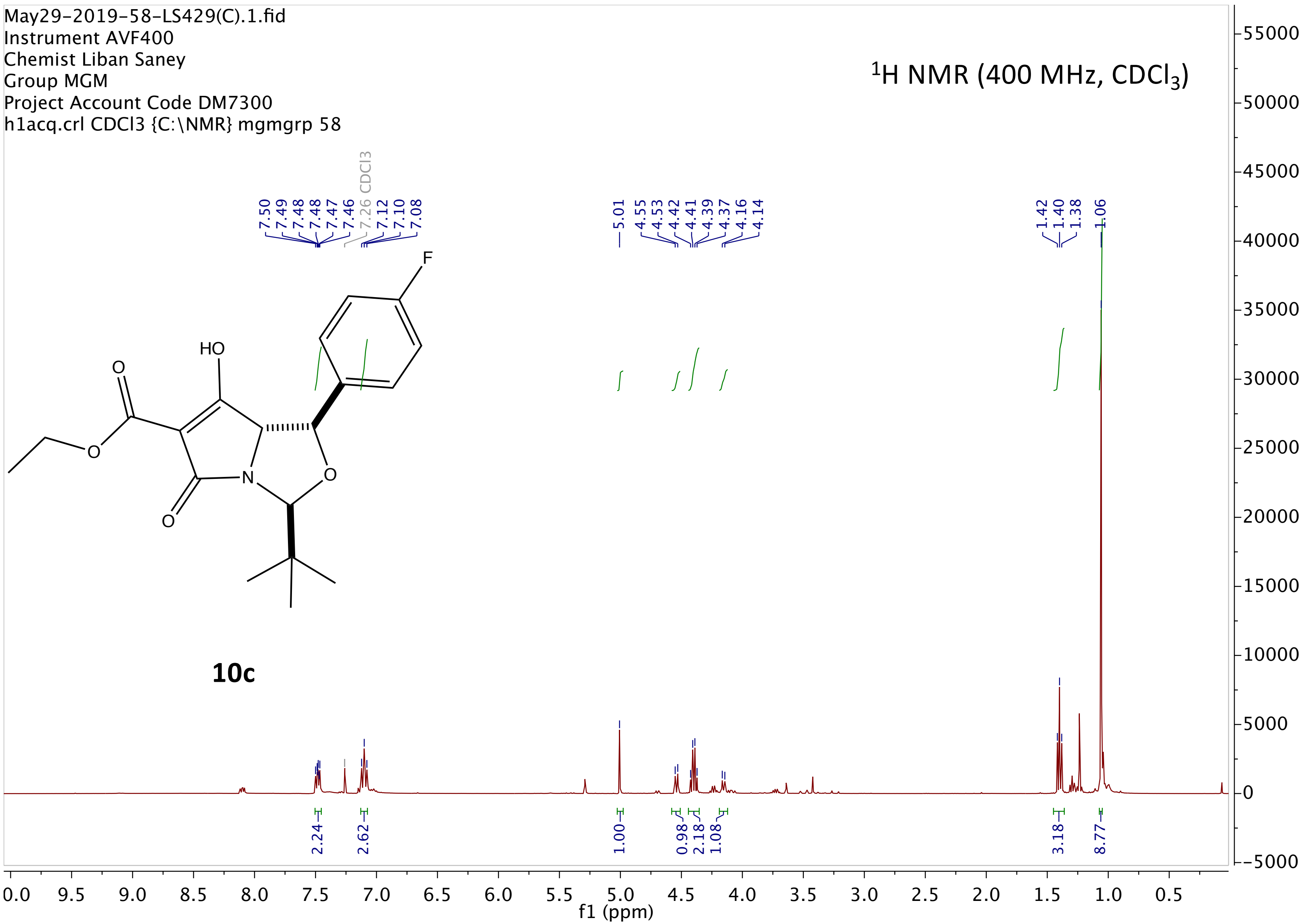
May29-2019-58-LS429(C).1.fid
Instrument AVF400
Chemist Liban Saney
Group MGM
Project Account Code DM7300
h1acq.crl CDCl3 {C:\NMR} mgmgrp 58
^{1}H NMR (400 MHz, $CDCl_3$)
7.50 7.49 7.48 7.48 7.47 7.46 7.26 CDCl3 7.12 7.10 7.08
5.01 4.55 4.53 4.42 4.41 4.39 4.37 4.16 4.14
1.42 1.40 1.38 1.06
HO
O
O
O
N
O
F
10c
2.24 2.62 1.00 0.98 2.18 1.08 3.18 8.77
f1 (ppm)

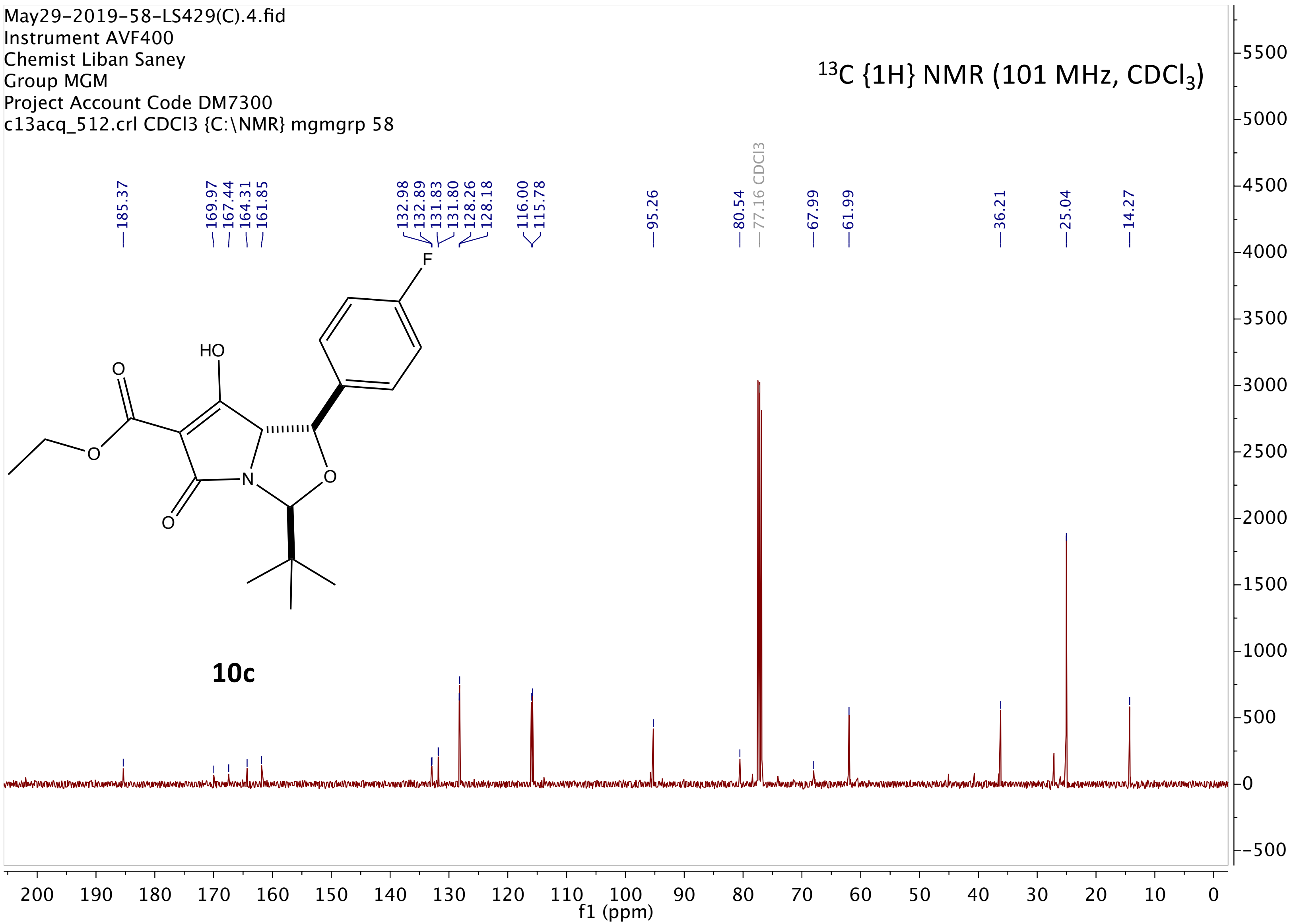
May29-2019-58-LS429(C).4.fid
Instrument AVF400
Chemist Liban Saney
Group MGM
Project Account Code DM7300
c13acq_512.crl CDCl3 {C:\NMR} mgmgrp 58
^{13}C {1H} NMR (101 MHz, $CDCl_3$)
185.37
169.97
167.44
164.31
161.85
132.98
132.89
131.83
131.80
128.26
128.18
116.00
115.78
95.26
80.54
77.16 CDCl3
67.99
61.99
36.21
25.04
14.27
F
HO
O
O
N
O
O
10c
f1 (ppm)

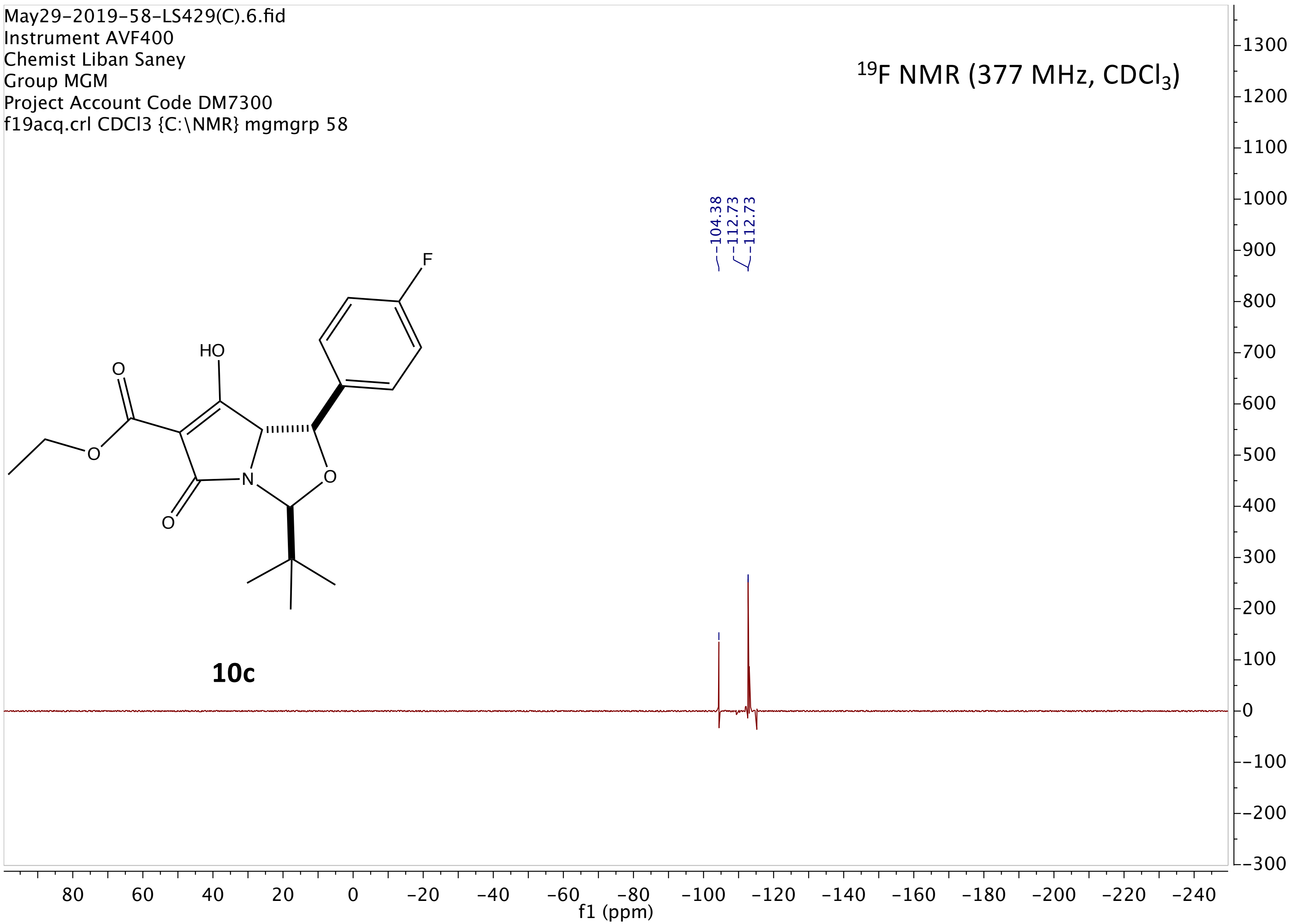
May29-2019-58-LS429(C).6.fid
Instrument AVF400
Chemist Liban Saney
Group MGM
Project Account Code DM7300
f19acq.crl CDCl3 {C:\NMR} mgmgrp 58
19F NMR (377 MHz, CDCl3)
-104.38
-112.73
-112.73
F
HO
O
O
O
N
O
10c
f1 (ppm)

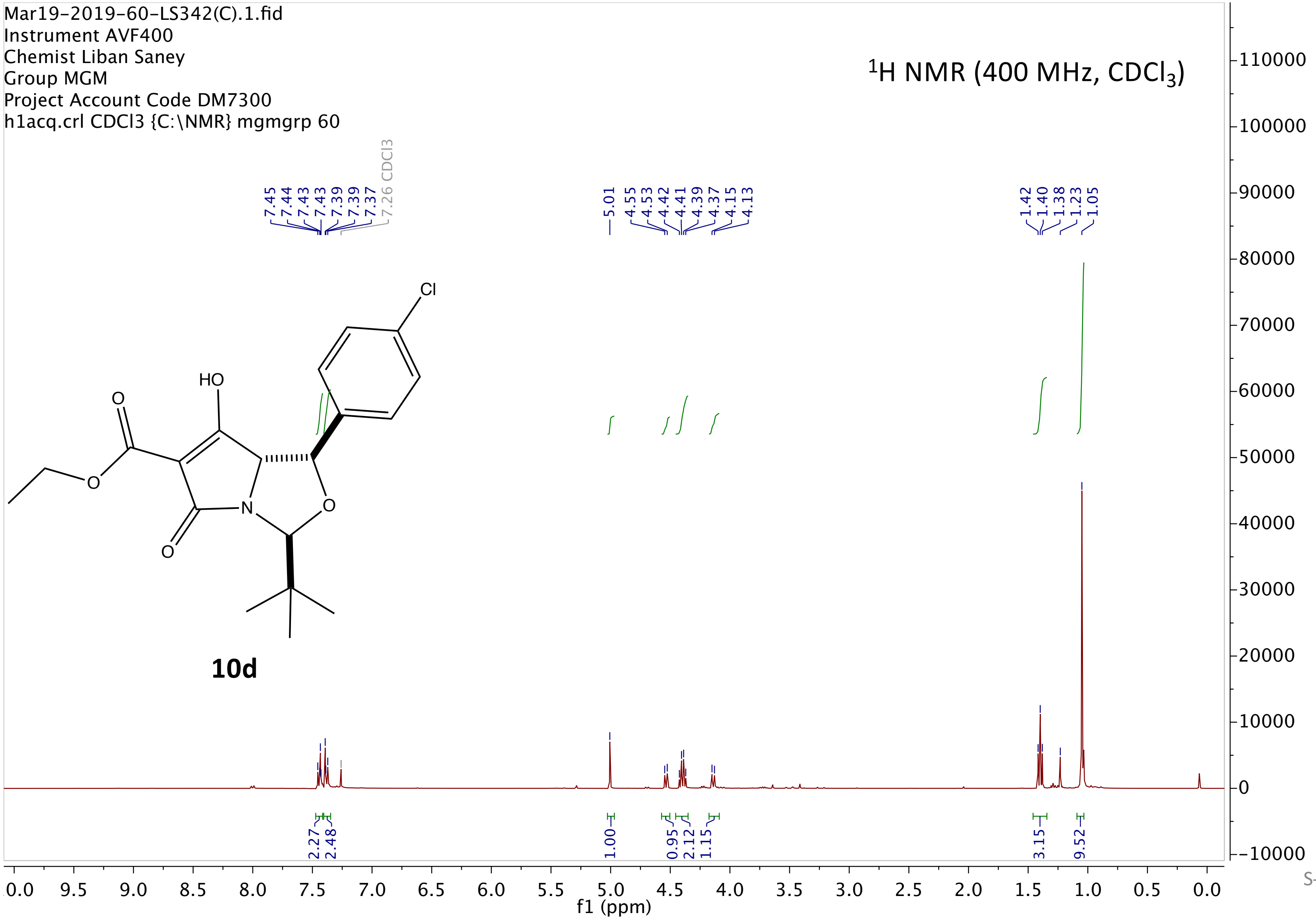
Mar19-2019-60-LS342(C).1.fid
Instrument AVF400
Chemist Liban Saney
Group MGM
Project Account Code DM7300
h1acq.crl CDCl3 {C:\NMR} mgmgrp 60
1H NMR (400 MHz, CDCl3)
7.45 7.44 7.43 7.43 7.39 7.39 7.37 7.26 CDCl3
5.01 4.55 4.53 4.42 4.41 4.39 4.37 4.15 4.13
1.42 1.40 1.38 1.23 1.05
2.27 2.48 1.00 0.95 2.12 1.15 3.15 9.52
Cl
HO
O
O
N
O
O
10d
f1 (ppm)

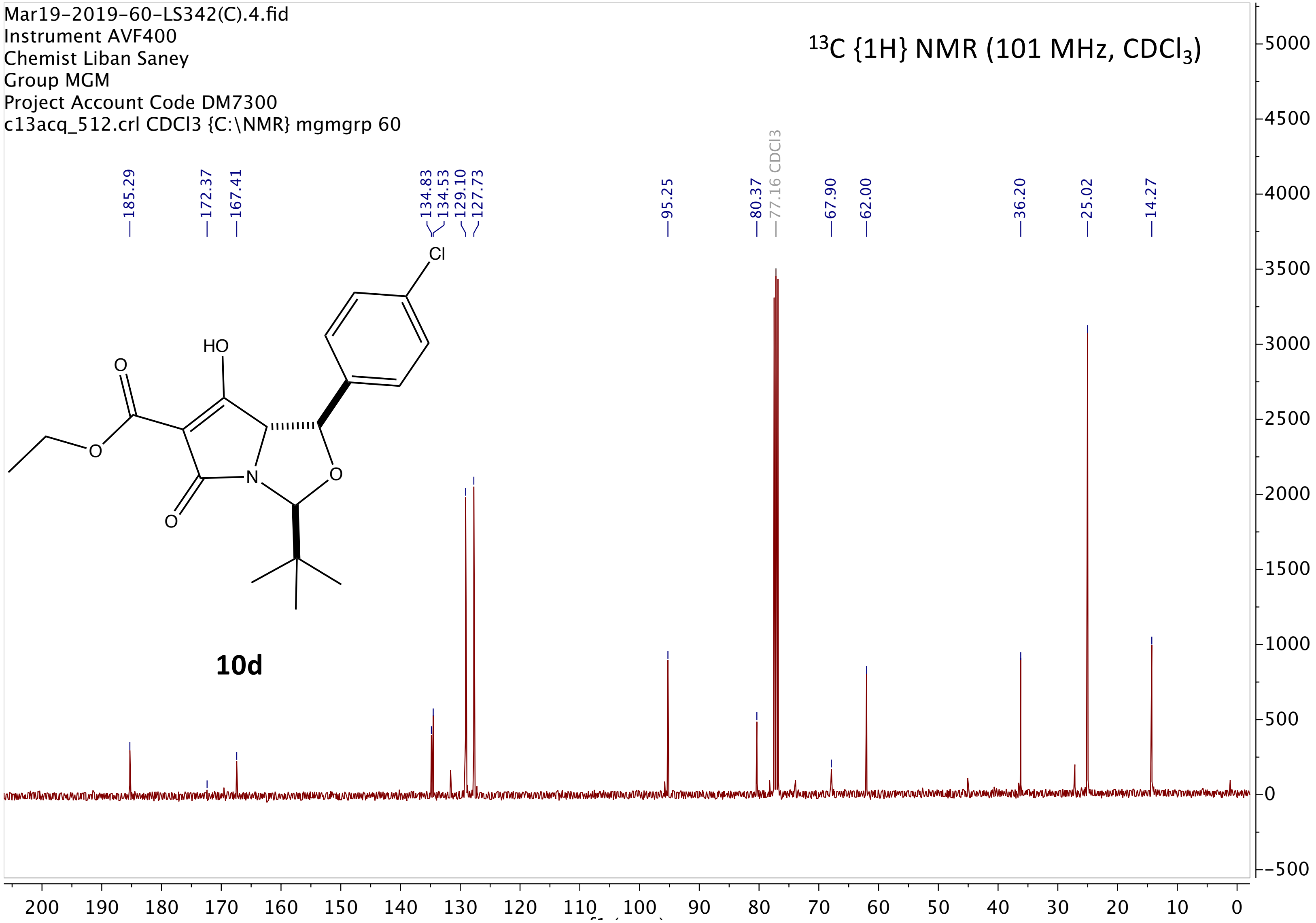
Mar19-2019-60-LS342(C).4.fid
Instrument AVF400
Chemist Liban Saney
Group MGM
Project Account Code DM7300
c13acq_512.crl CDCl3 {C:\NMR} mgmgrp 60
13C {1H} NMR (101 MHz, CDCl3)
185.29
172.37
167.41
134.83
134.53
129.10
127.73
95.25
80.37
77.16 CDCl3
67.90
62.00
36.20
25.02
14.27
Cl
HO
O
O
N
O
O
10d
5000
4500
4000
3500
3000
2500
2000
1500
1000
500
0
-500
200
190
180
170
160
150
140
130
120
110
100
90
80
70
60
50
40
30
20
10
0

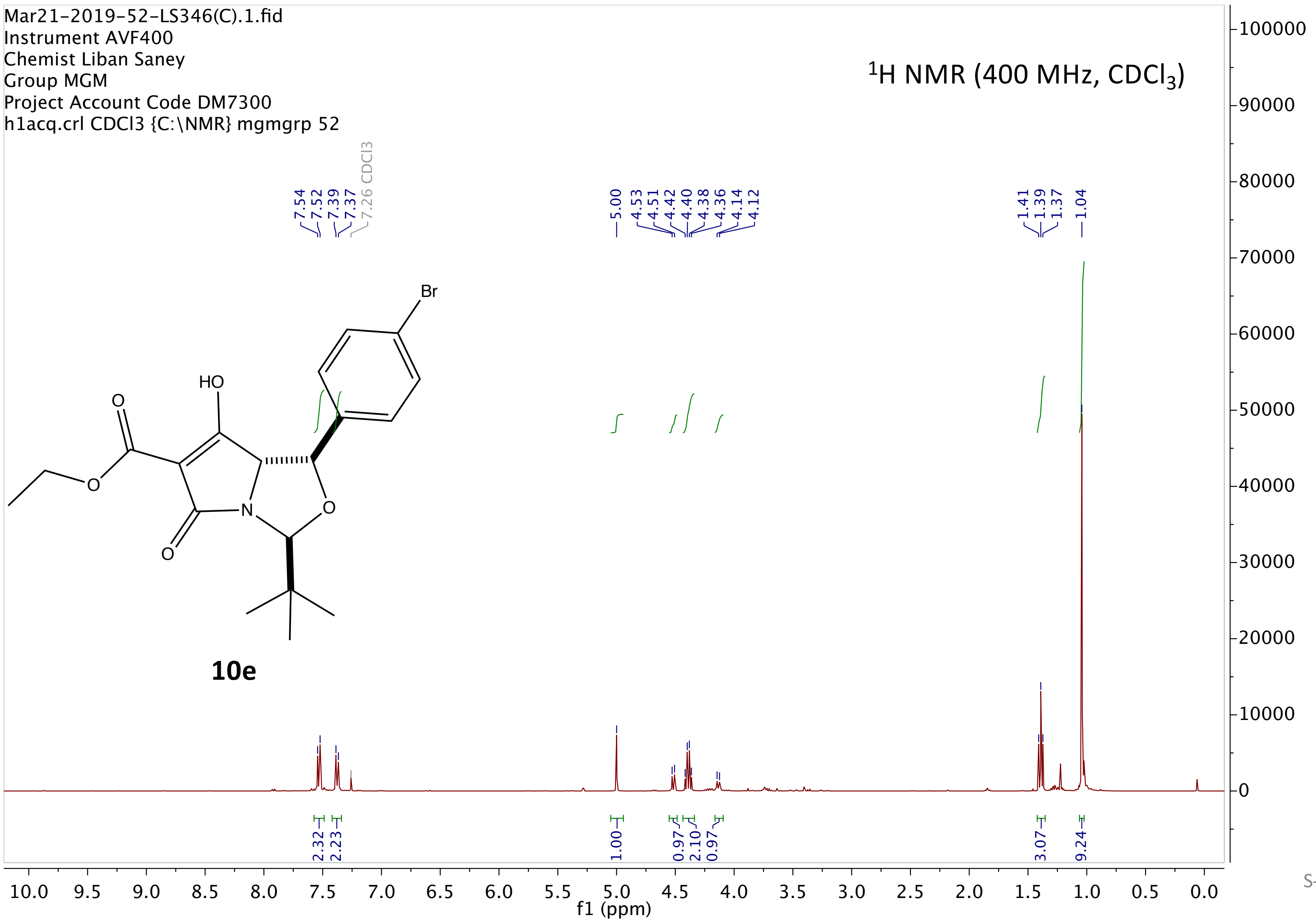

Mar21-2019-52-LS346(C).1.fid
Instrument AVF400
Chemist Liban Saney
Group MGM
Project Account Code DM7300
h1acq.crl CDCl3 {C:\NMR} mgmgrp 52
1H NMR (400 MHz, CDCl3)
7.54
7.52
7.39
7.37
7.26 CDCl3
5.00
4.53
4.51
4.42
4.40
4.38
4.36
4.14
4.12
1.41
1.39
1.37
1.04
Br
HO
O
O
N
O
O
10e
2.32
2.23
1.00
0.97
2.10
0.97
3.07
9.24
f1 (ppm)

Mar21-2019-52-LS346(C).4.fid
Instrument AVF400
Chemist Liban Saney
Group MGM
Project Account Code DM7300
c13acq_512.crl CDCl3 {C:\NMR} mgmgrp 52

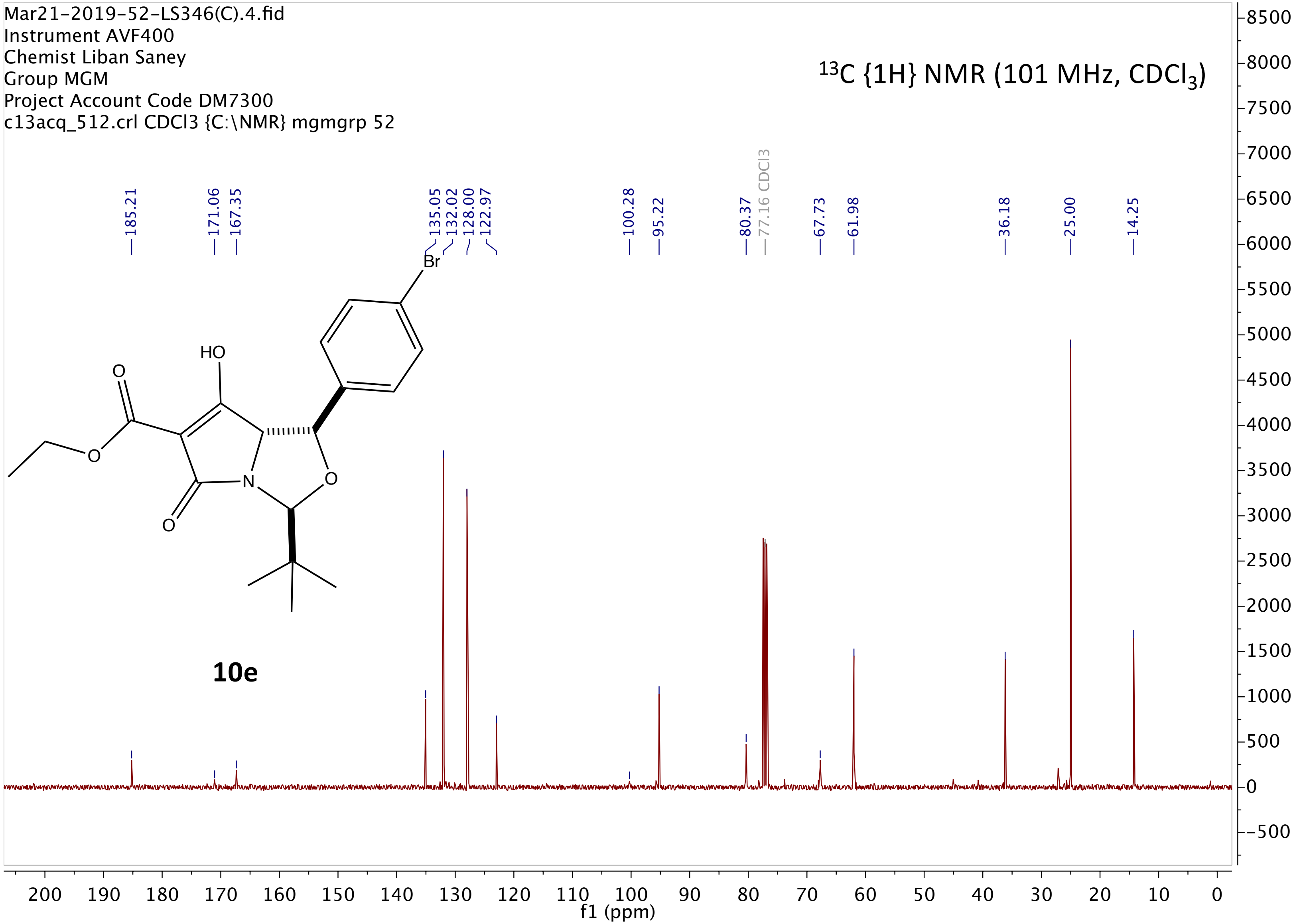

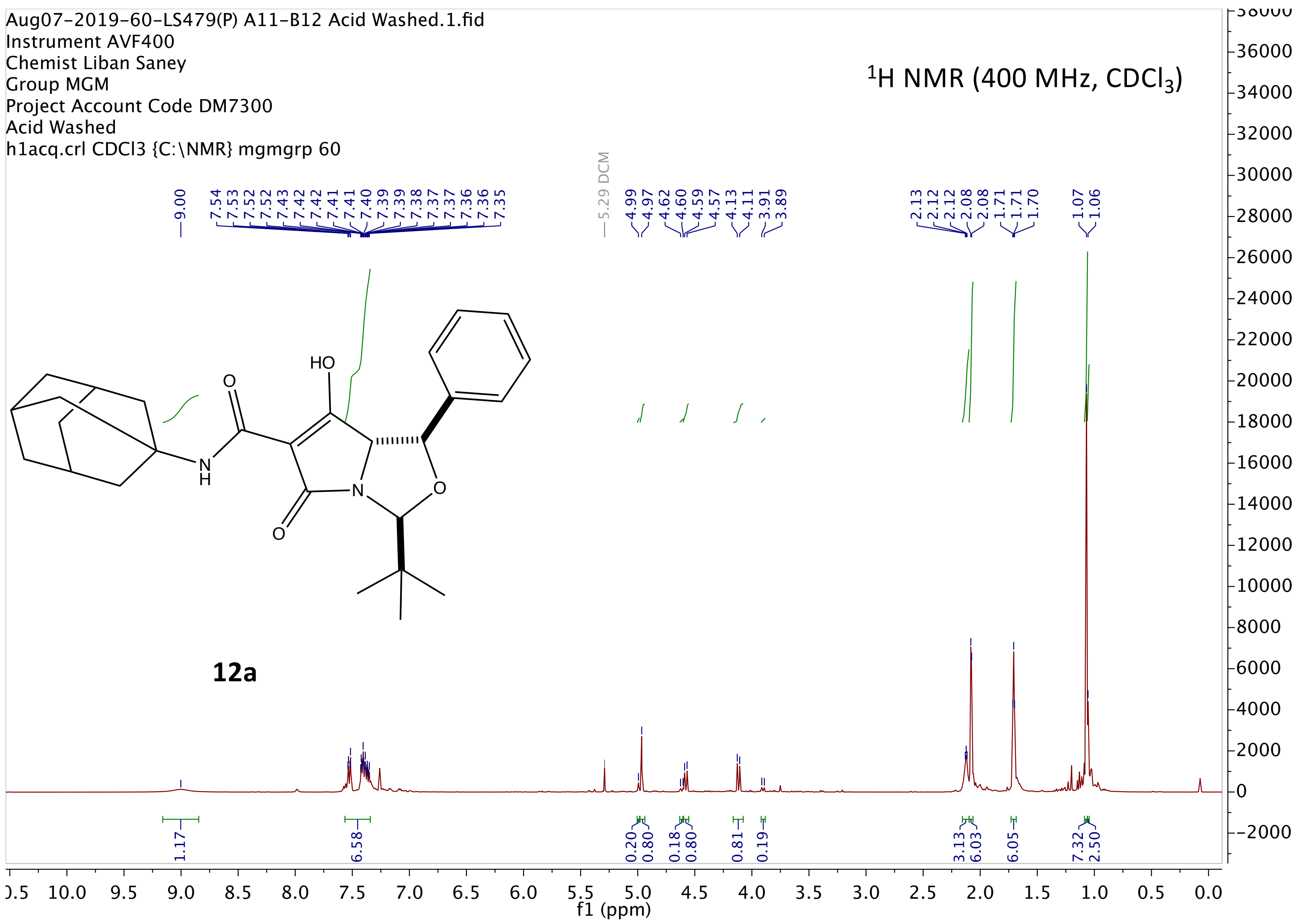

Aug07-2019-60-LS479(P) A11-B12 Acid Washed.1.fid
Instrument AVF400
Chemist Liban Saney
Group MGM
Project Account Code DM7300
Acid Washed
h1acq.crl CDCl3 {C:\NMR} mgmgrp 60
1H NMR (400 MHz, CDCl3)
12a
HO
O
N
H
O
f1 (ppm)
5.29 DCM

Aug07-2019-60-LS479(P) A11-B12 Acid Washed.4.fid
Instrument AVF400
Chemist Liban Saney
Group MGM
Project Account Code DM7300
Acid Washed
c13acq_512.crl CDCl3 {C:\NMR} mgmgrp 60

^{13}C {1H} NMR (101 MHz, $CDCl_3$)

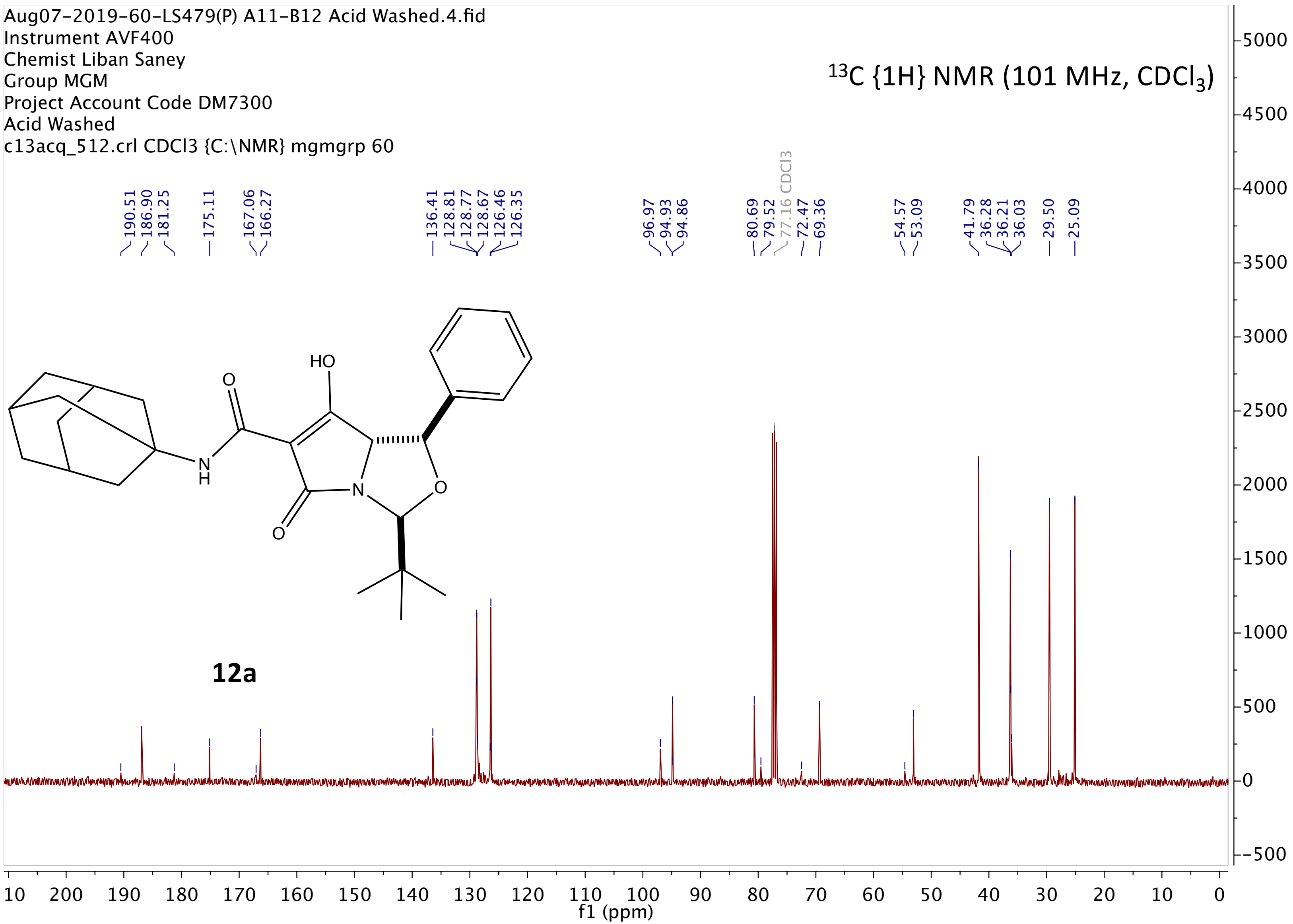

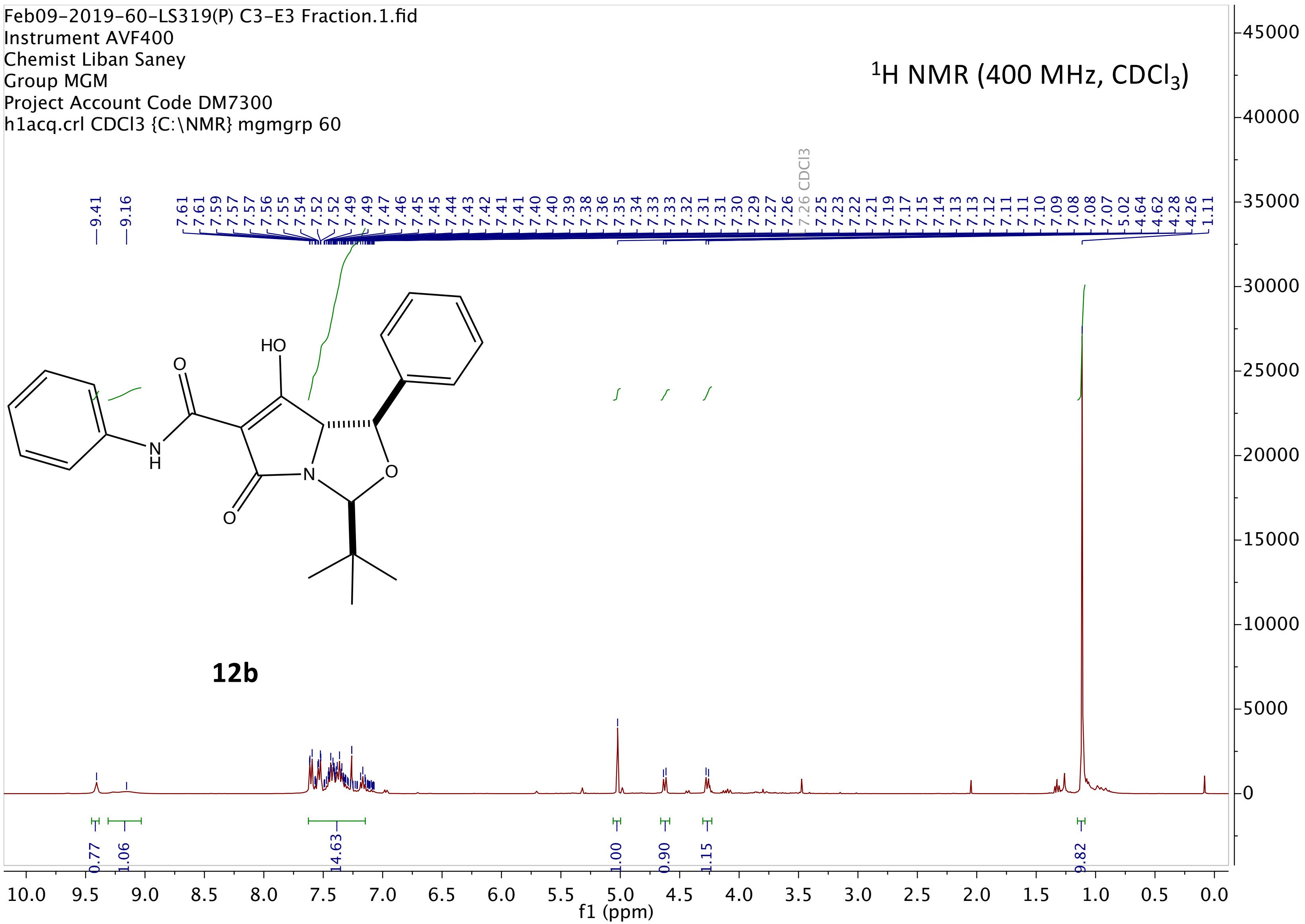
Feb09-2019-60-LS319(P) C3-E3 Fraction.1.fid
Instrument AVF400
Chemist Liban Saney
Group MGM
Project Account Code DM7300
h1acq.crl CDCl3 {C:\NMR} mgmgrp 60
1H NMR (400 MHz, CDCl3)
12b
f1 (ppm)

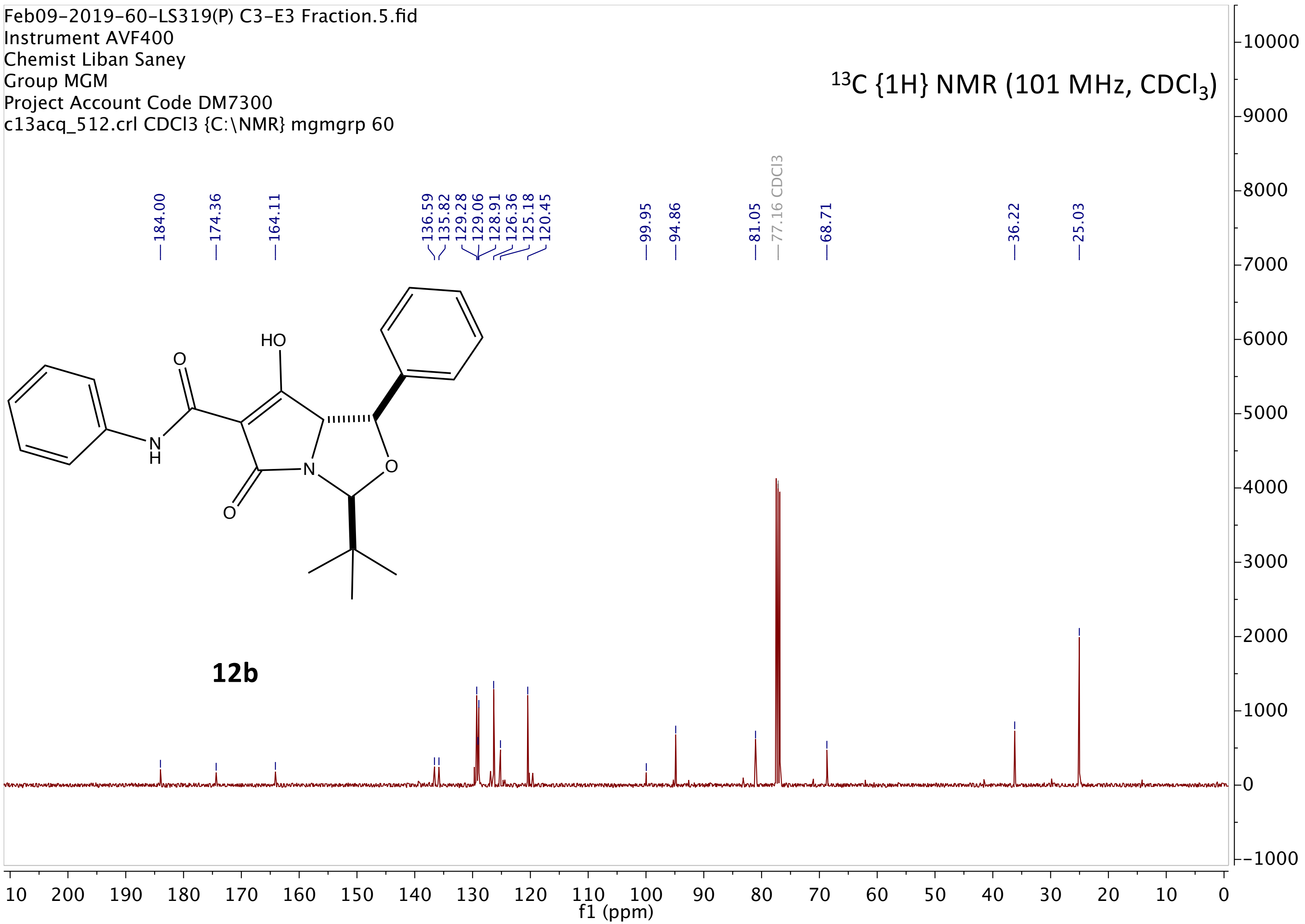

Feb09-2019-60-LS319(P) C3-E3 Fraction.5.fid
Instrument AVF400
Chemist Liban Saney
Group MGM
Project Account Code DM7300
c13acq_512.crl CDCl3 {C:\NMR} mgmgrp 60
13C {1H} NMR (101 MHz, CDCl3)
184.00
174.36
164.11
136.59
135.82
129.28
129.06
128.91
126.36
125.18
120.45
99.95
94.86
81.05
77.16 CDCl3
68.71
36.22
25.03
HO
O
N
H
N
O
O
12b
f1 (ppm)

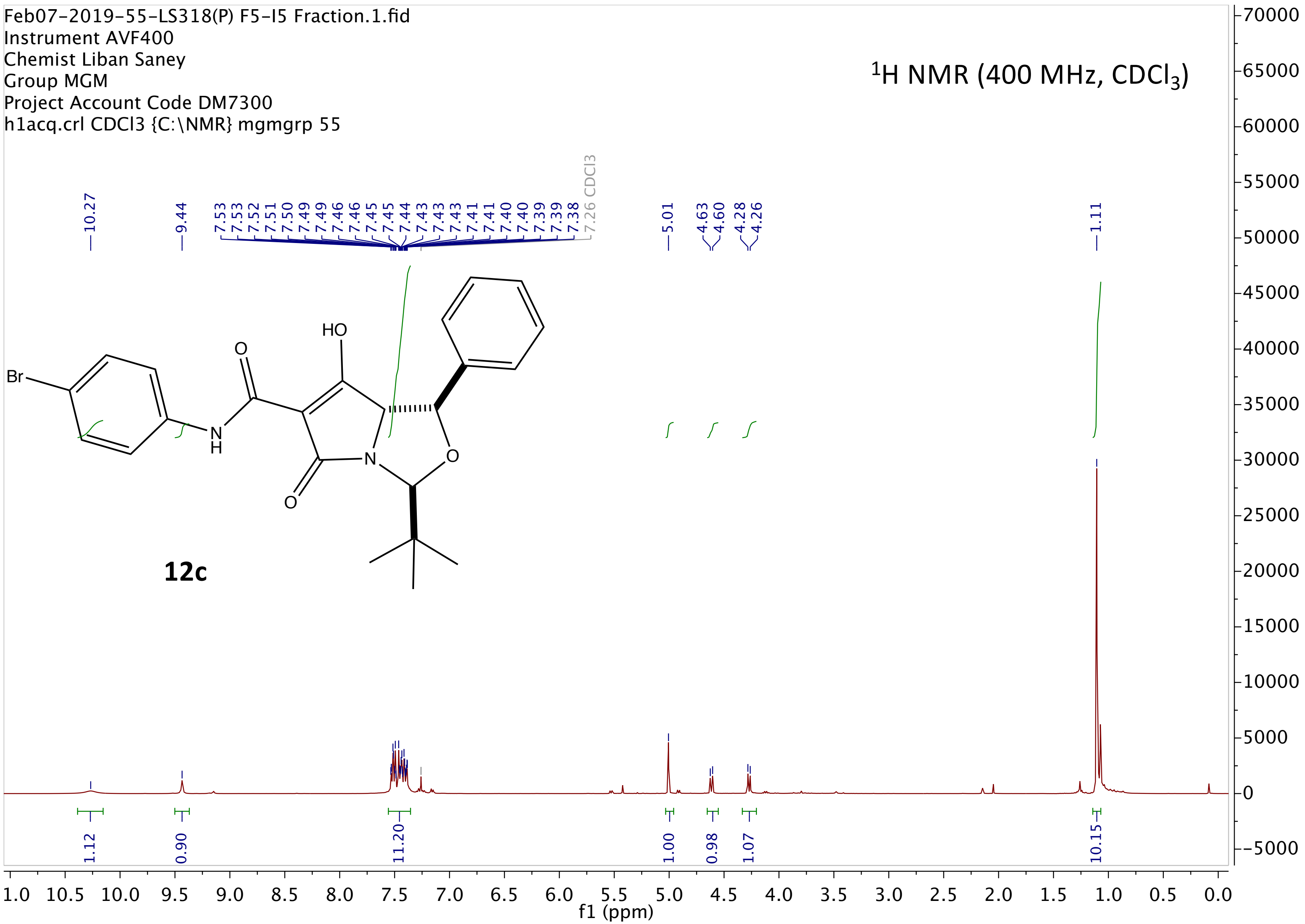
Feb07-2019-55-LS318(P) F5-I5 Fraction.1.fid
Instrument AVF400
Chemist Liban Saney
Group MGM
Project Account Code DM7300
h1acq.crl CDCl3 {C:\NMR} mgmgrp 55
1H NMR (400 MHz, CDCl3)
10.27
9.44
7.53
7.53
7.52
7.51
7.50
7.49
7.49
7.46
7.46
7.45
7.45
7.44
7.43
7.43
7.43
7.41
7.41
7.40
7.40
7.39
7.39
7.38
7.26 CDCl3
5.01
4.63
4.60
4.28
4.26
1.11
HO
O
Br
N
H
N
O
O
12c
1.12
0.90
11.20
1.00
0.98
1.07
10.15
f1 (ppm)

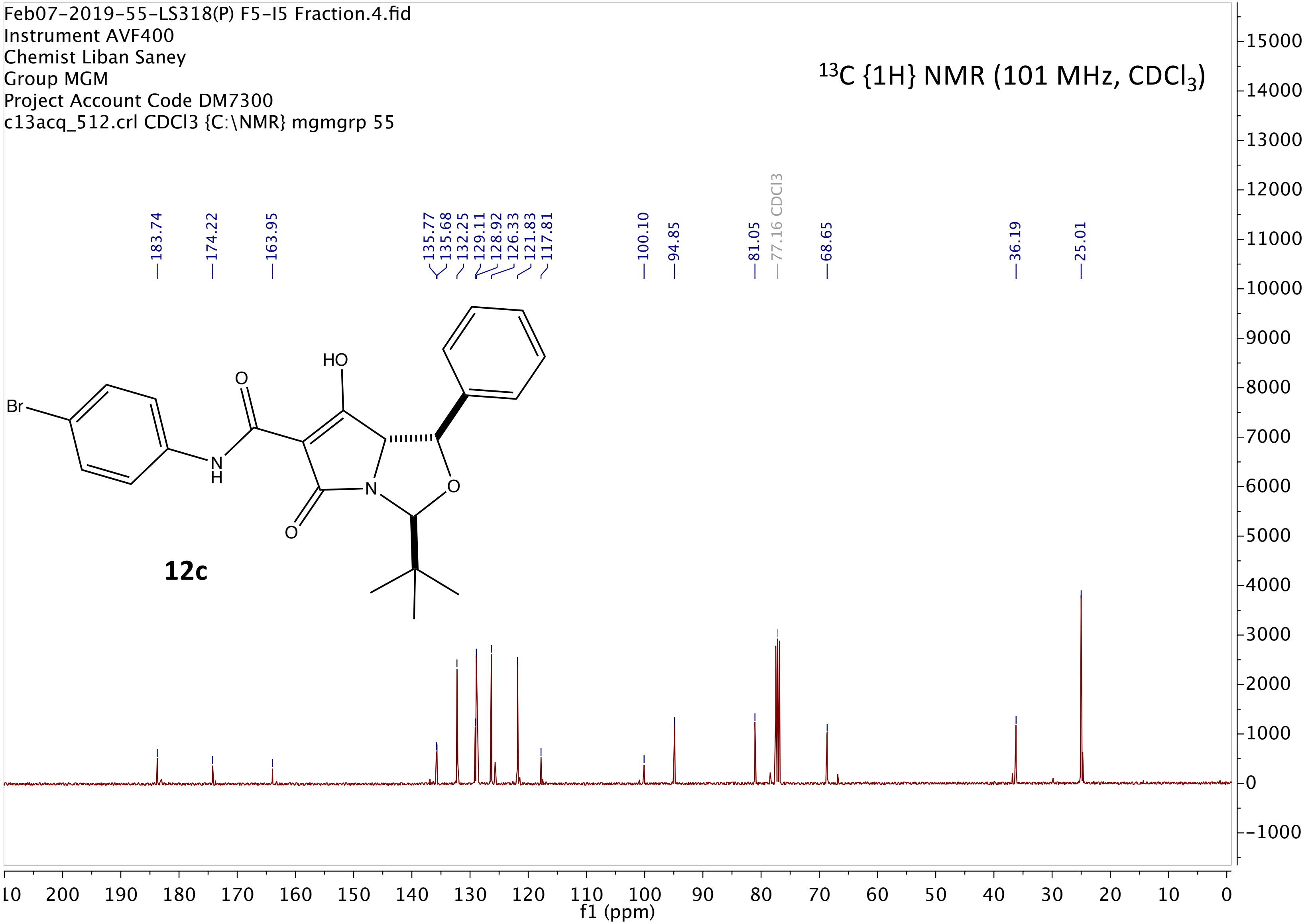
Feb07-2019-55-LS318(P) F5-I5 Fraction.4.fid
Instrument AVF400
Chemist Liban Saney
Group MGM
Project Account Code DM7300
c13acq_512.crl CDCl3 {C:\NMR} mgmgrp 55
13C {1H} NMR (101 MHz, CDCl3)
183.74
174.22
163.95
135.77
135.68
132.25
129.11
128.92
126.33
121.83
117.81
100.10
94.85
81.05
77.16 CDCl3
68.65
36.19
25.01
HO
O
Br
N
H
N
O
O
12c
f1 (ppm)

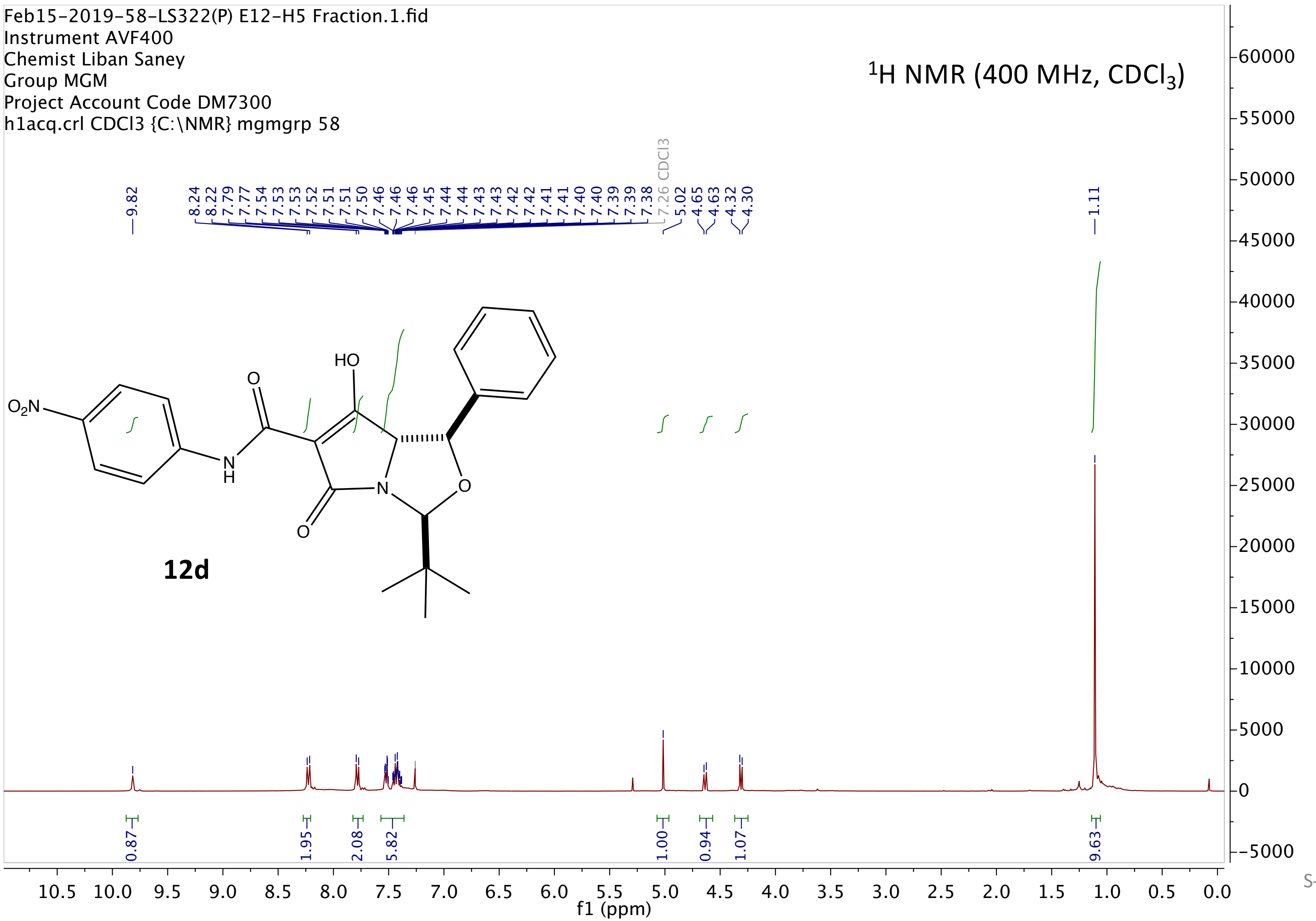
Feb15-2019-58-LS322(P) E12-H5 Fraction.1.fid
Instrument AVF400
Chemist Liban Saney
Group MGM
Project Account Code DM7300
h1acq.crl CDCl3 {C:\NMR} mgmgrp 58
1H NMR (400 MHz, CDCl3)
9.82
8.24
8.22
7.79
7.77
7.54
7.53
7.53
7.52
7.51
7.51
7.50
7.46
7.46
7.46
7.45
7.44
7.44
7.43
7.43
7.42
7.42
7.41
7.41
7.40
7.40
7.39
7.39
7.38
7.26 CDCl3
5.02
4.65
4.63
4.32
4.30
1.11
HO
O
O2N
N
H
N
O
O
12d
0.87
1.95
2.08
5.82
1.00
0.94
1.07
9.63
f1 (ppm)

Feb15-2019-58-LS322(P) E12-H5 Fraction.4.fid
Instrument AVF400
Chemist Liban Saney
Group MGM
Project Account Code DM7300
c13acq_512.crl CDCl3 {C:\NMR} mgmgrp 58

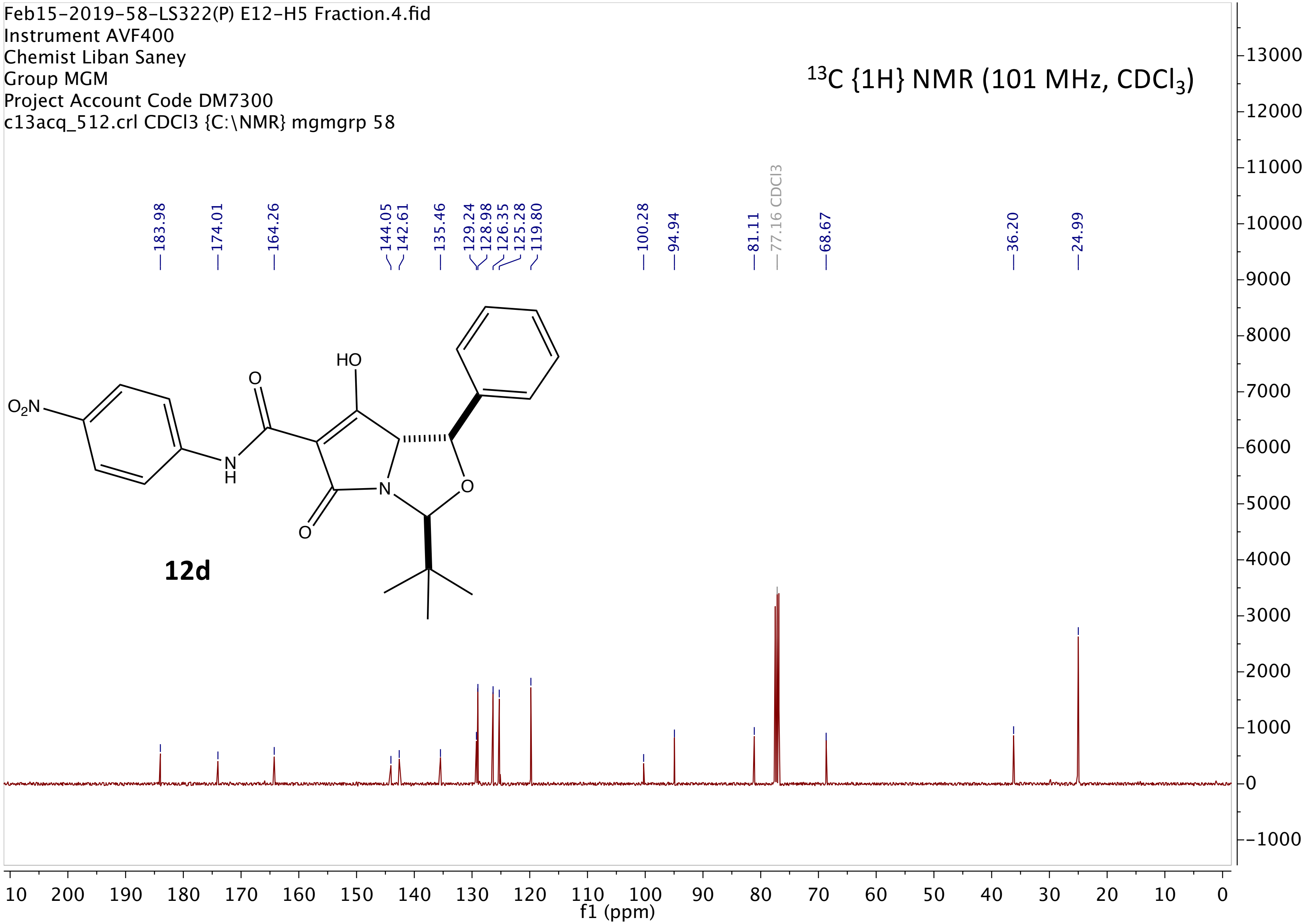

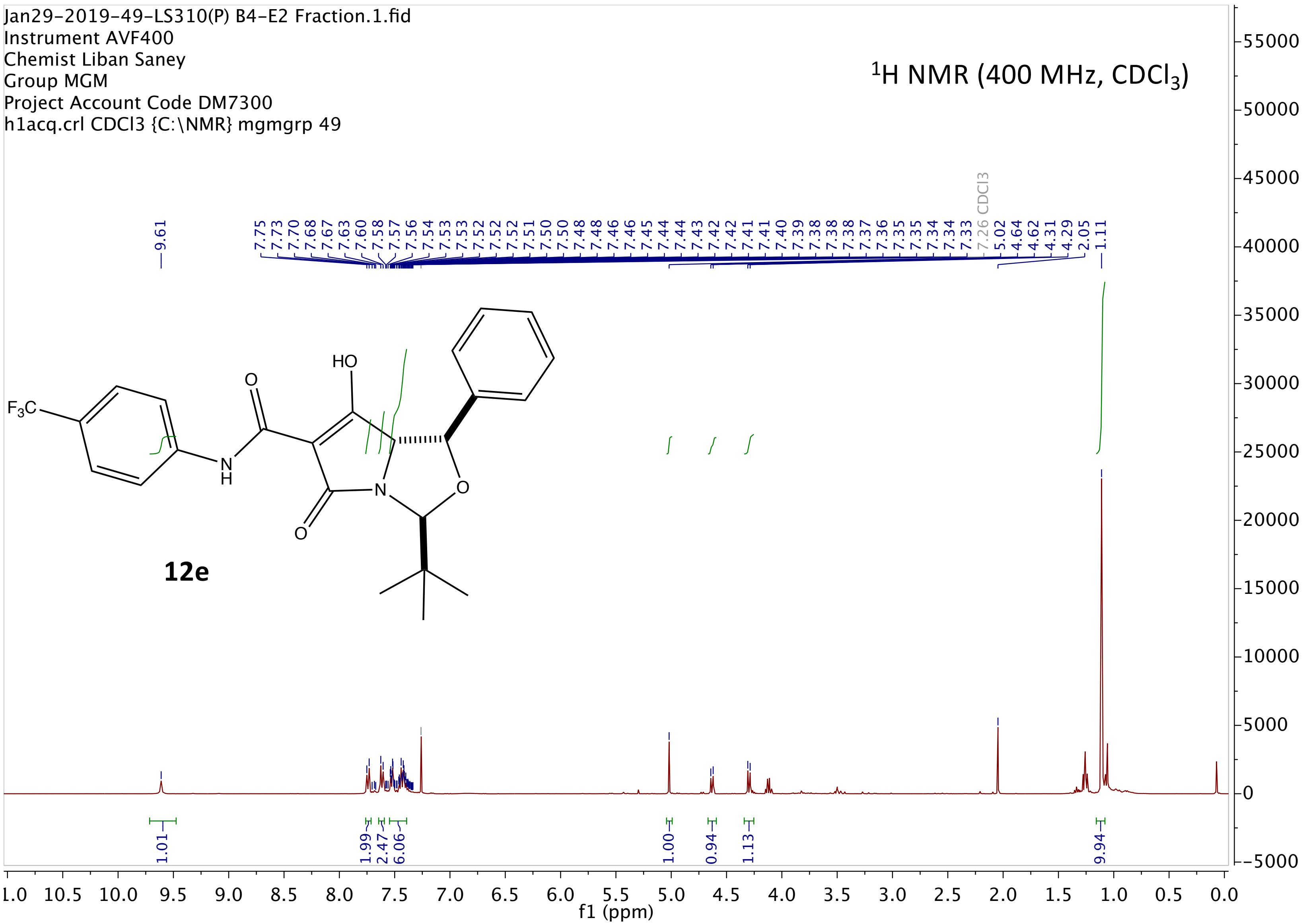
Jan29-2019-49-LS310(P) B4-E2 Fraction.1.fid
Instrument AVF400
Chemist Liban Saney
Group MGM
Project Account Code DM7300
h1acq.crl CDCl3 {C:\NMR} mgmgrp 49
1H NMR (400 MHz, CDCl3)
9.61
7.75 7.73 7.70 7.68 7.67 7.63 7.60 7.58 7.57 7.56 7.54 7.53 7.53 7.52 7.52 7.52 7.51 7.50 7.50 7.48 7.48 7.46 7.46 7.45 7.44 7.44 7.43 7.42 7.42 7.41 7.41 7.40 7.39 7.38 7.38 7.37 7.36 7.35 7.35 7.34 7.34 7.33
7.26 CDCl3
5.02 4.64 4.62 4.31 4.29 2.05 1.11
F3C
HO
O
N
H
N
O
O
12e
1.01
1.99
2.47
6.06
1.00
0.94
1.13
9.94
f1 (ppm)
11.0 10.5 10.0 9.5 9.0 8.5 8.0 7.5 7.0 6.5 6.0 5.5 5.0 4.5 4.0 3.5 3.0 2.5 2.0 1.5 1.0 0.5 0.0
55000 50000 45000 40000 35000 30000 25000 20000 15000 10000 5000 0 -5000

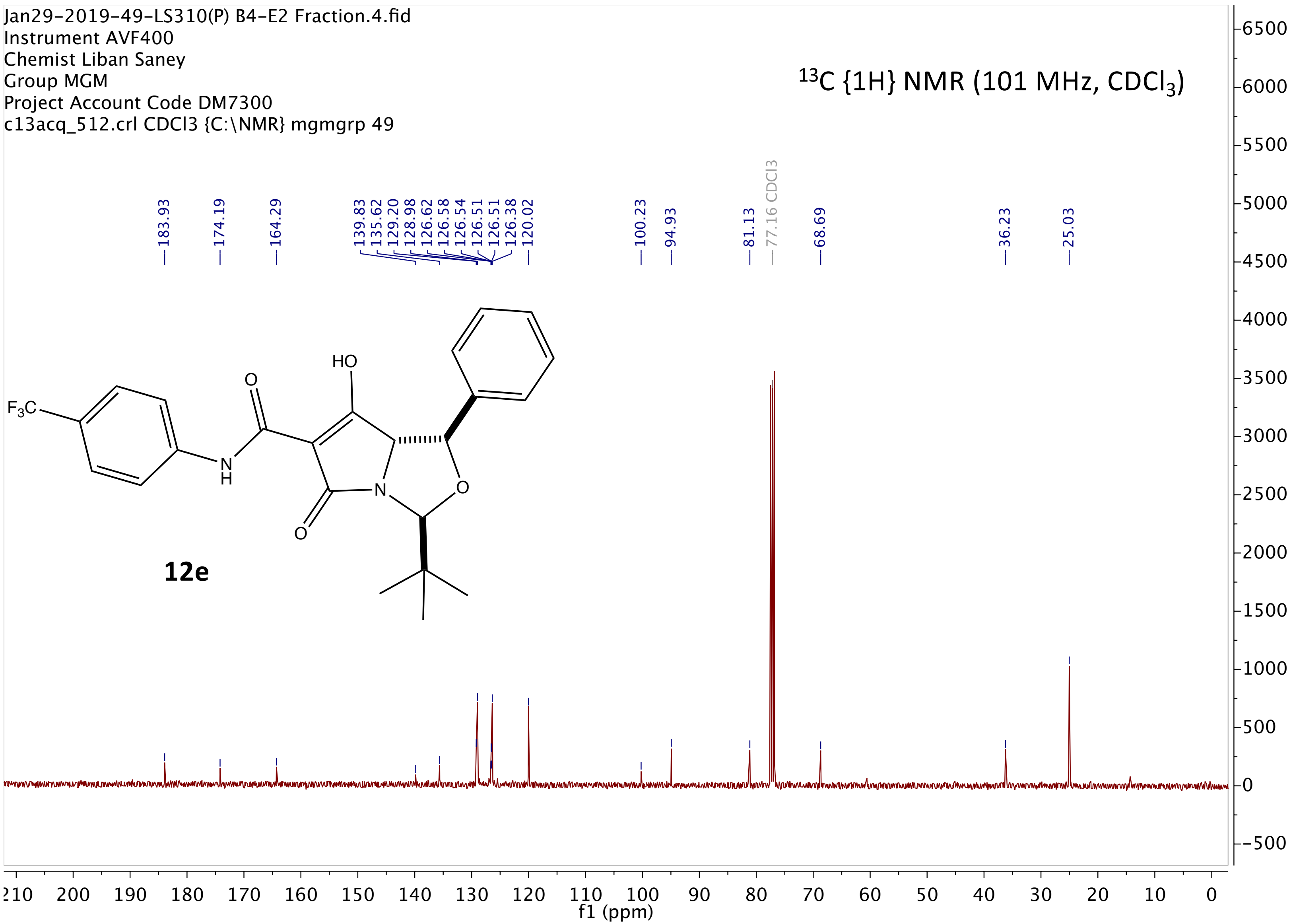
Jan29-2019-49-LS310(P) B4-E2 Fraction.4.fid
Instrument AVF400
Chemist Liban Saney
Group MGM
Project Account Code DM7300
c13acq_512.crl CDCl3 {C:\NMR} mgmgrp 49
13C {1H} NMR (101 MHz, CDCl3)
183.93
174.19
164.29
139.83
135.62
129.20
128.98
126.62
126.58
126.54
126.51
126.51
126.38
120.02
100.23
94.93
81.13
77.16 CDCl3
68.69
36.23
25.03
HO
O
F3C
N
H
N
O
O
12e
f1 (ppm)

Jan29-2019-49-LS310(P) B4-E2 Fraction.6.fid
Instrument AVF400
Chemist Liban Saney
Group MGM
Project Account Code DM7300
f19acq.crl CDCl3 {C:\NMR} mgmgrp 49

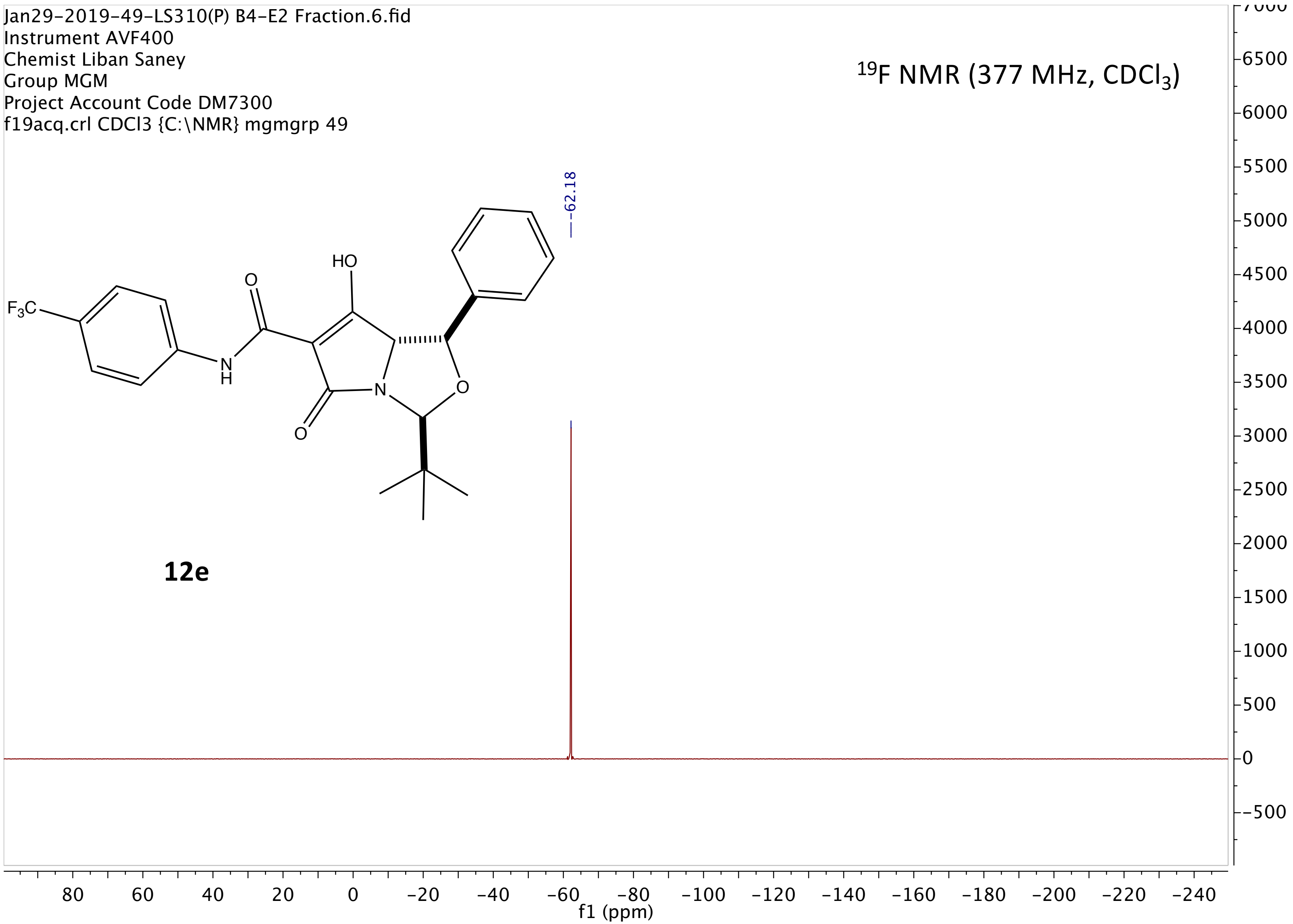

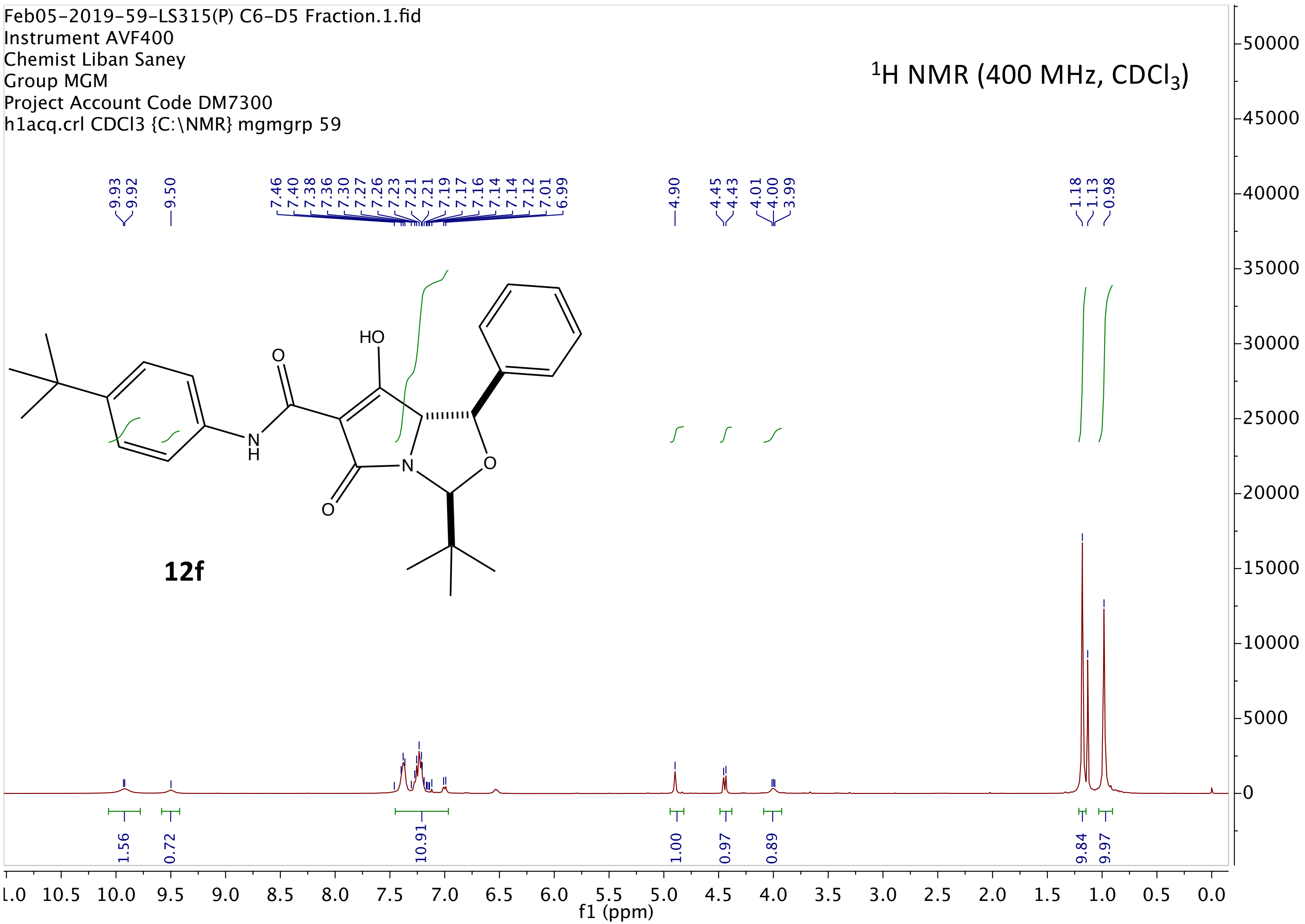
Feb05-2019-59-LS315(P) C6-D5 Fraction.1.fid
Instrument AVF400
Chemist Liban Saney
Group MGM
Project Account Code DM7300
h1acq.crl CDCl3 {C:\NMR} mgmgrp 59
1H NMR (400 MHz, CDCl3)
9.93
9.92
9.50
7.46
7.40
7.38
7.36
7.30
7.27
7.26
7.23
7.21
7.21
7.19
7.17
7.16
7.14
7.14
7.12
7.01
6.99
4.90
4.45
4.43
4.01
4.00
3.99
1.18
1.13
0.98
HO
O
N
H
N
O
O
12f
1.56
0.72
10.91
1.00
0.97
0.89
9.84
9.97
f1 (ppm)

Feb05-2019-59-LS315(P) C6-D5 Fraction.4.fid
Instrument AVF400
Chemist Liban Saney
Group MGM
Project Account Code DM7300
c13acq_512.crl CDCl3 {C:\NMR} mgmgrp 59

^{13}C {1H} NMR (101 MHz, $CDCl_3$)

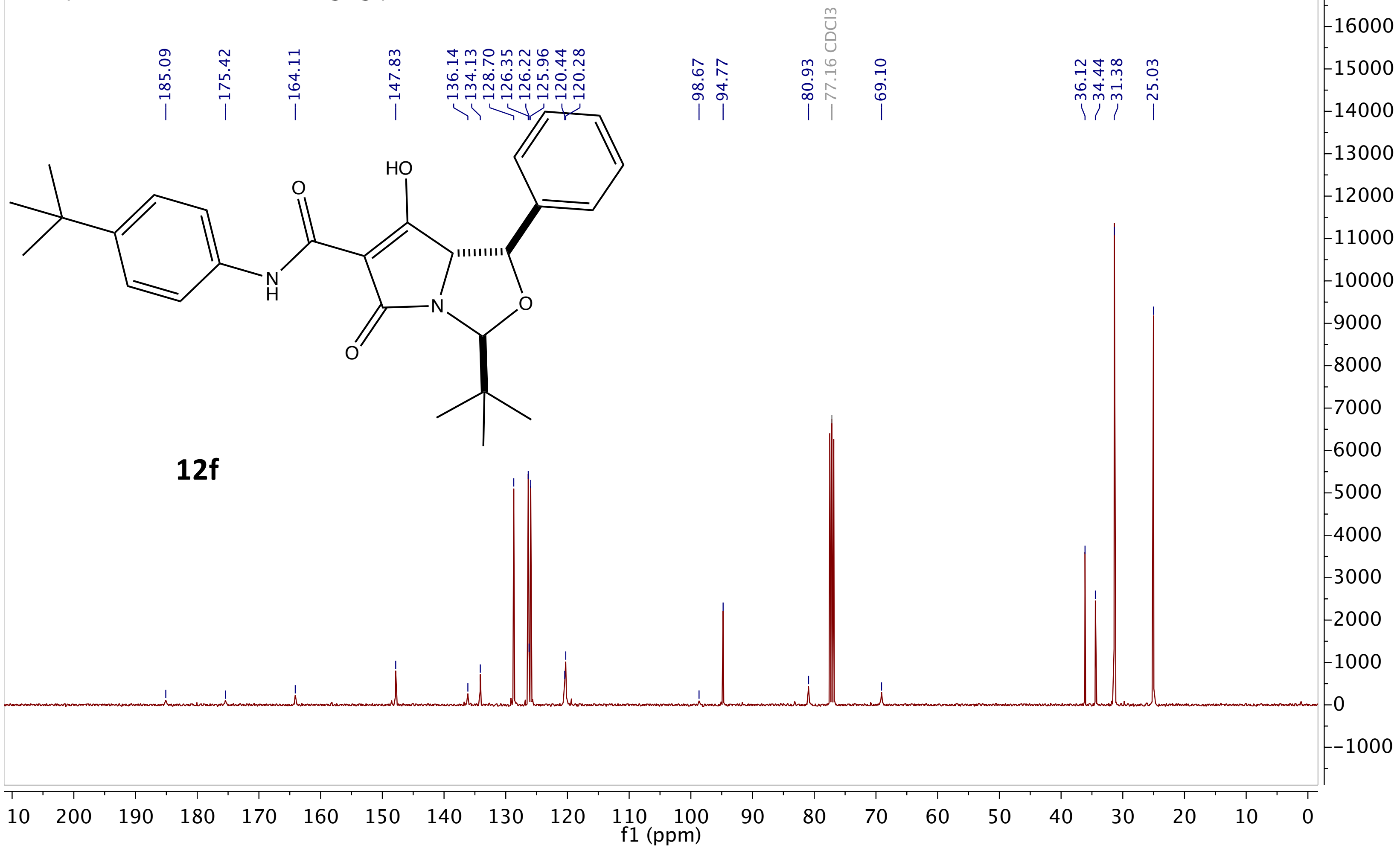

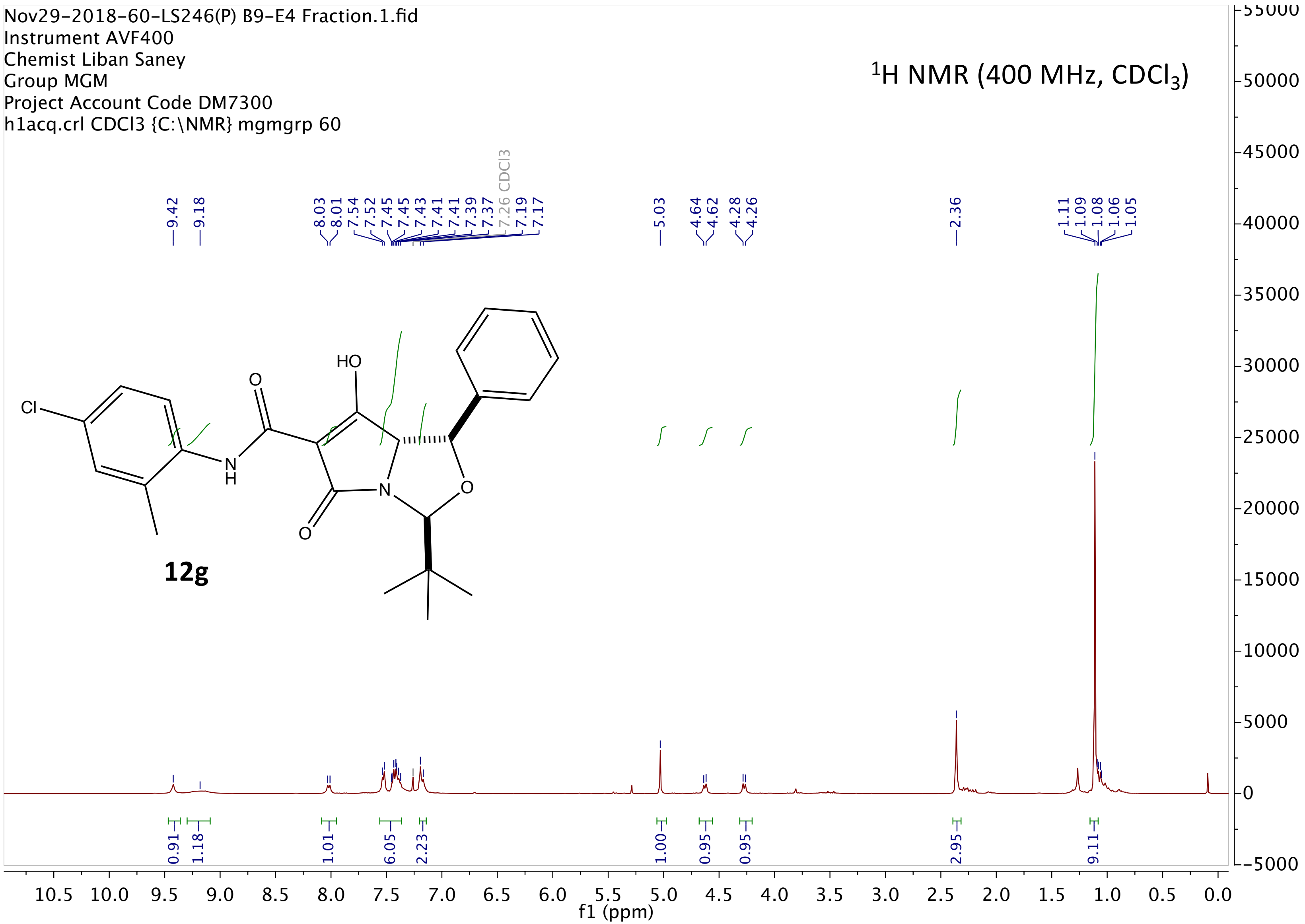
Nov29-2018-60-LS246(P) B9-E4 Fraction.1.fid
Instrument AVF400
Chemist Liban Saney
Group MGM
Project Account Code DM7300
h1acq.crl CDCl3 {C:\NMR} mgmgrp 60
1H NMR (400 MHz, CDCl3)
9.42
9.18
8.03
8.01
7.54
7.52
7.45
7.45
7.43
7.41
7.41
7.39
7.37
7.26 CDCl3
7.19
7.17
5.03
4.64
4.62
4.28
4.26
2.36
1.11
1.09
1.08
1.06
1.05
0.91
1.18
1.01
6.05
2.23
1.00
0.95
0.95
2.95
9.11
HO
O
Cl
N
H
N
O
O
12g
f1 (ppm)

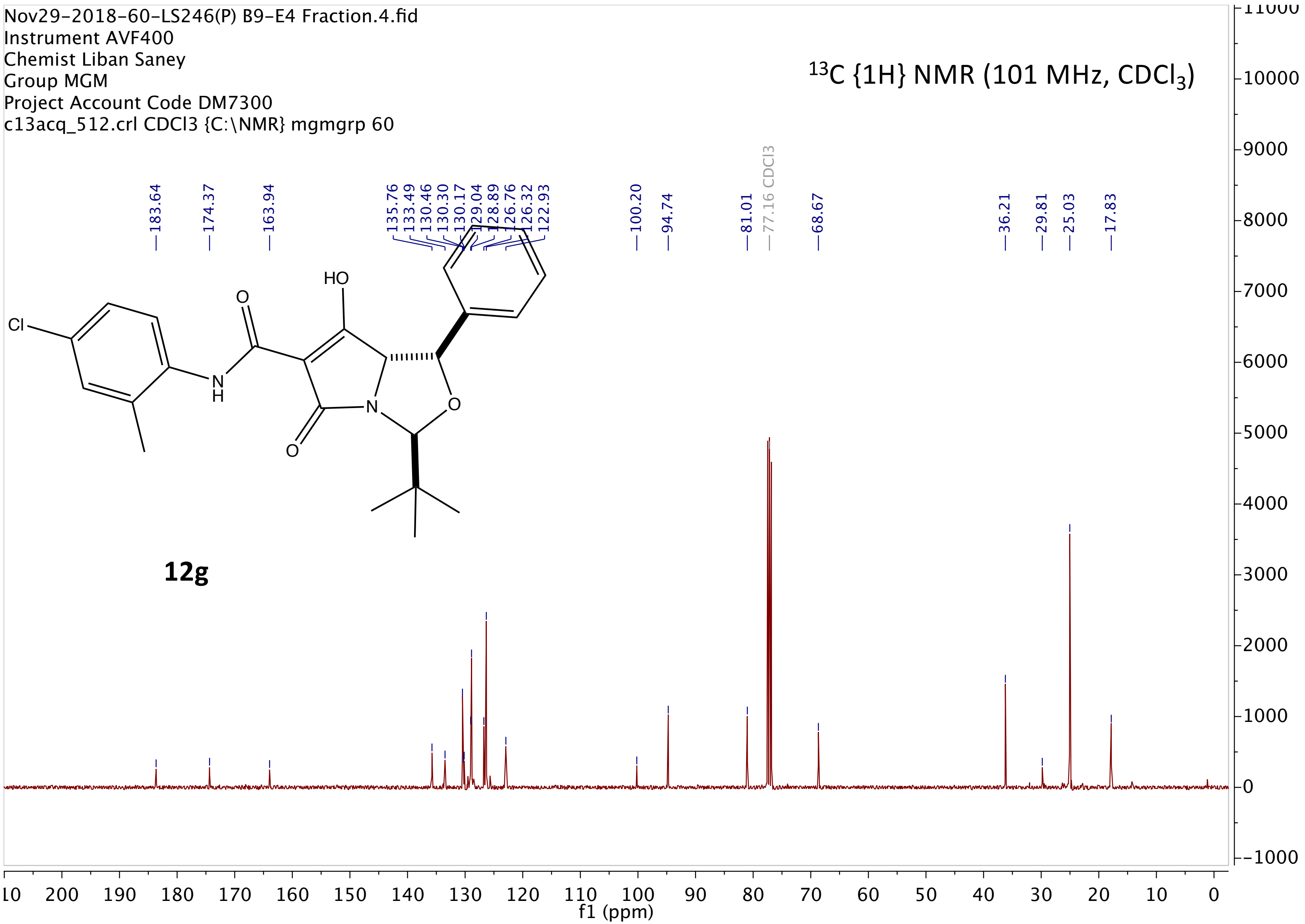
Nov29-2018-60-LS246(P) B9-E4 Fraction.4.fid
Instrument AVF400
Chemist Liban Saney
Group MGM
Project Account Code DM7300
c13acq_512.crl CDCl3 {C:\NMR} mgmgrp 60
^{13}C {1H} NMR (101 MHz, CDCl$_3$)
183.64
174.37
163.94
135.76
133.49
130.46
130.30
130.17
129.04
128.89
126.76
126.32
122.93
100.20
94.74
81.01
77.16 CDCl3
68.67
36.21
29.81
25.03
17.83
HO
O
Cl
N
H
N
O
O
12g
f1 (ppm)

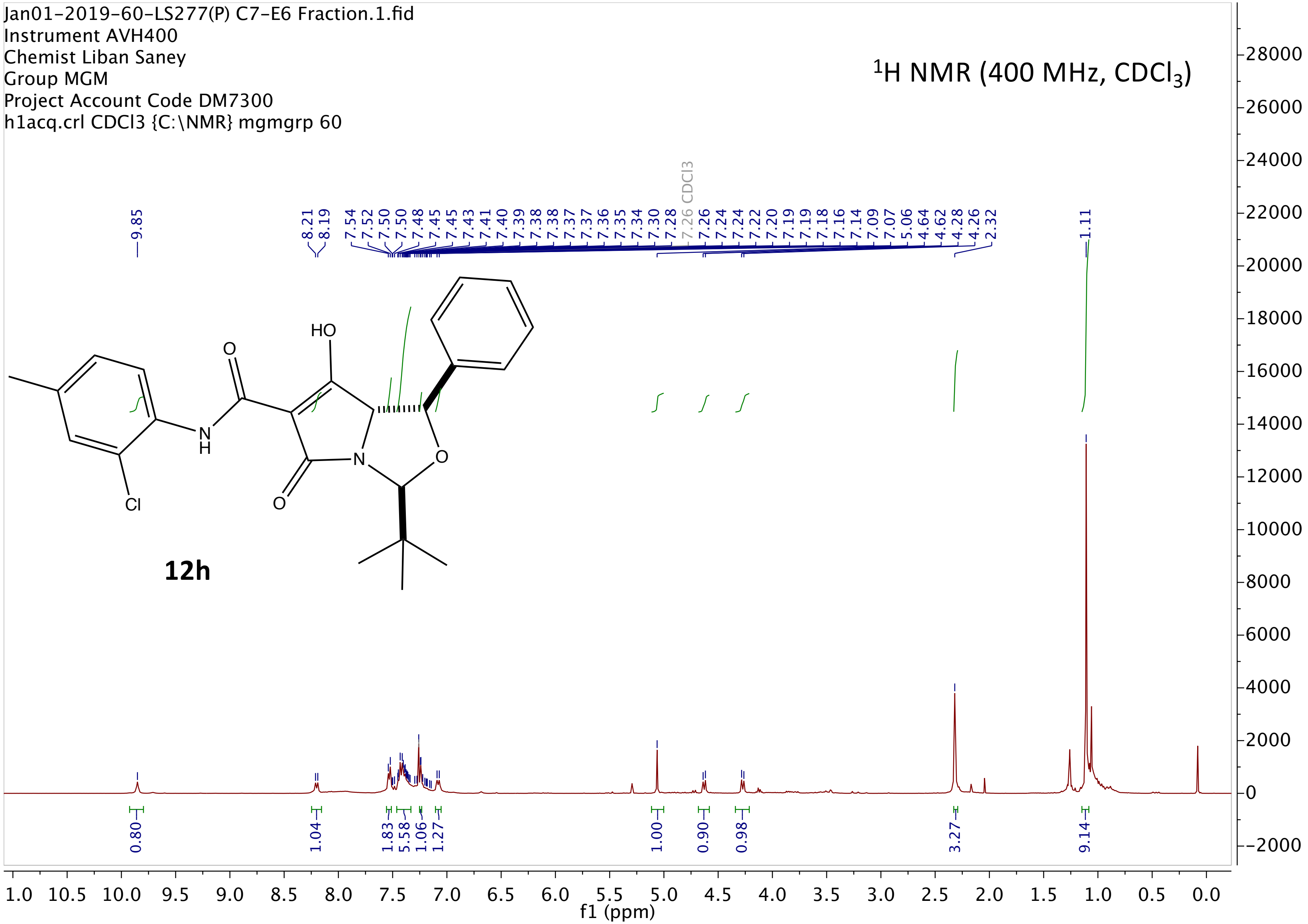
Jan01-2019-60-LS277(P) C7-E6 Fraction.1.fid
Instrument AVH400
Chemist Liban Saney
Group MGM
Project Account Code DM7300
h1acq.crl CDCl3 {C:\NMR} mgmgrp 60
¹H NMR (400 MHz, CDCl3)
9.85
8.21
8.19
7.54
7.52
7.50
7.50
7.48
7.45
7.45
7.43
7.41
7.40
7.39
7.38
7.38
7.37
7.37
7.36
7.35
7.34
7.30
7.28
7.26 CDCl3
7.26
7.24
7.24
7.22
7.20
7.19
7.19
7.18
7.16
7.14
7.09
7.07
5.06
4.64
4.62
4.28
4.26
2.32
1.11
HO
O
N
H
Cl
O
N
O
12h
0.80
1.04
1.83
5.58
1.06
1.27
1.00
0.90
0.98
3.27
9.14
f1 (ppm)

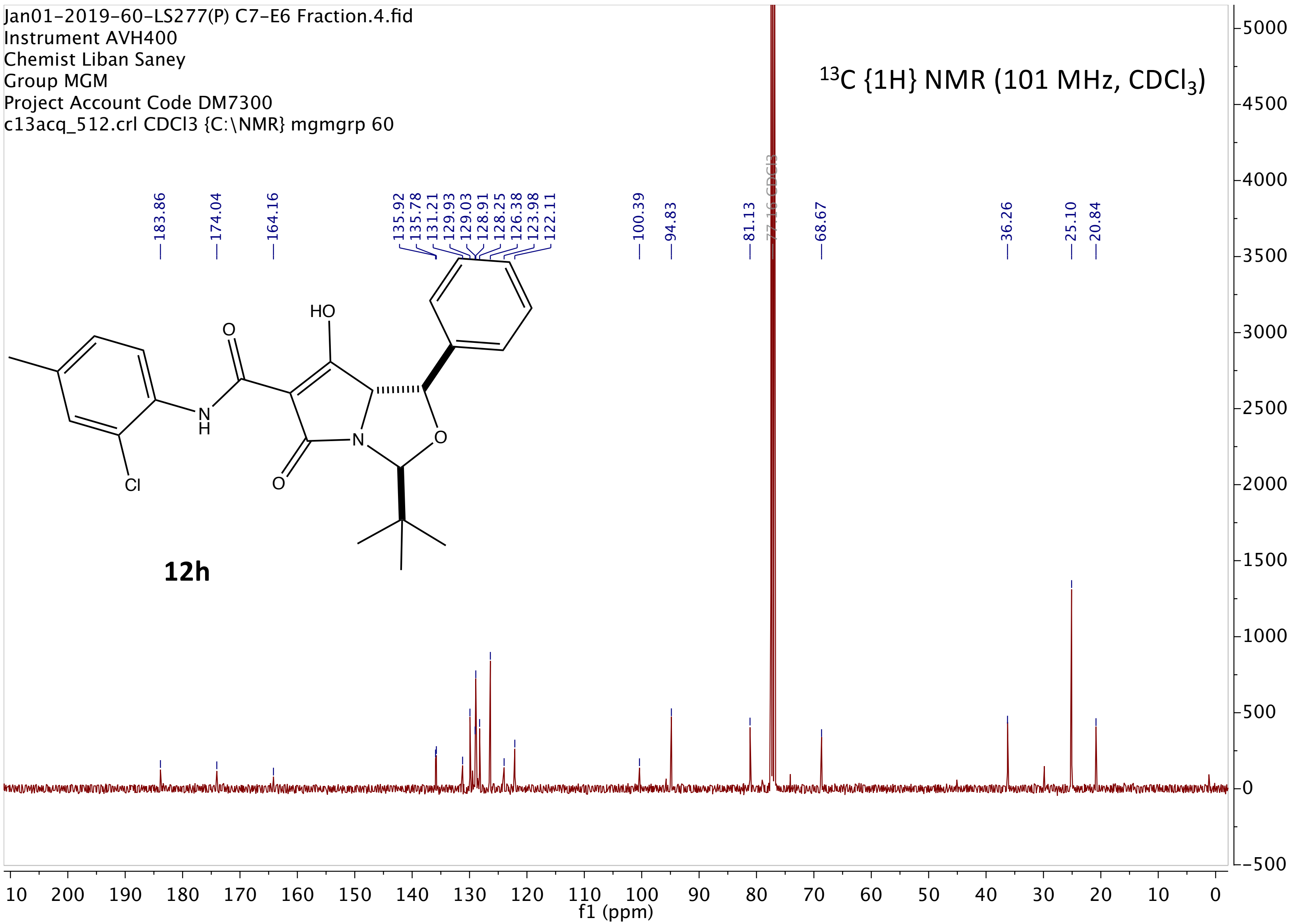
Jan01-2019-60-LS277(P) C7-E6 Fraction.4.fid
Instrument AVH400
Chemist Liban Saney
Group MGM
Project Account Code DM7300
c13acq_512.crl CDCl3 {C:\NMR} mgmgrp 60
13C {1H} NMR (101 MHz, CDCl3)
183.86
174.04
164.16
135.92
135.78
131.21
129.93
129.03
128.91
128.25
126.38
123.98
122.11
100.39
94.83
81.13
77.16 CDCl3
68.67
36.26
25.10
20.84
HO
O
N
H
Cl
O
N
O
12h
f1 (ppm)

Feb01-2019-59-LS313(P) D4-D12 Fraction.1.fid
Instrument AVF400
Chemist Liban Saney
Group MGM
Project Account Code DM7300
h1acq.crl CDCl3 {C:\NMR} mgmgrp 59

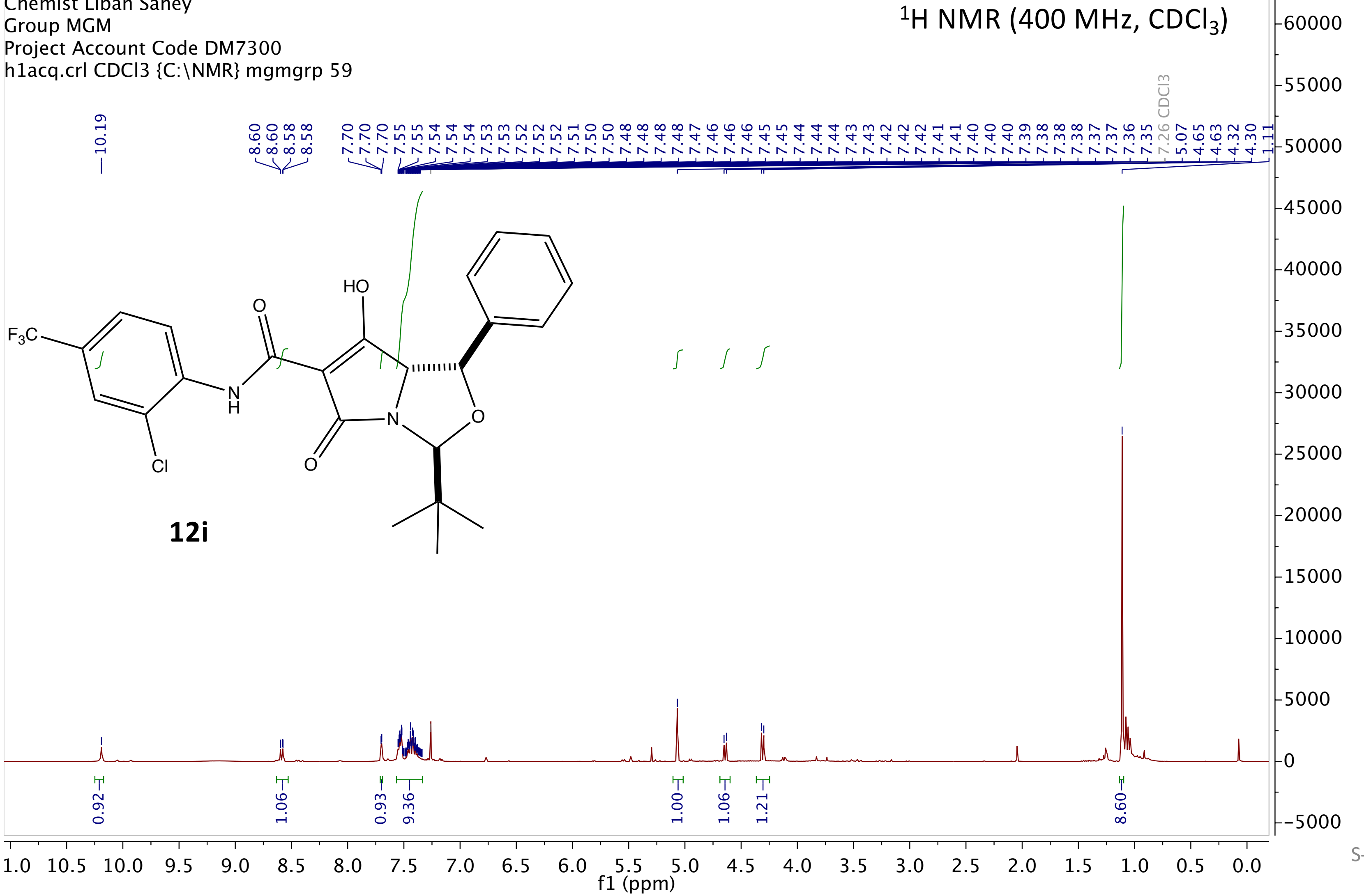

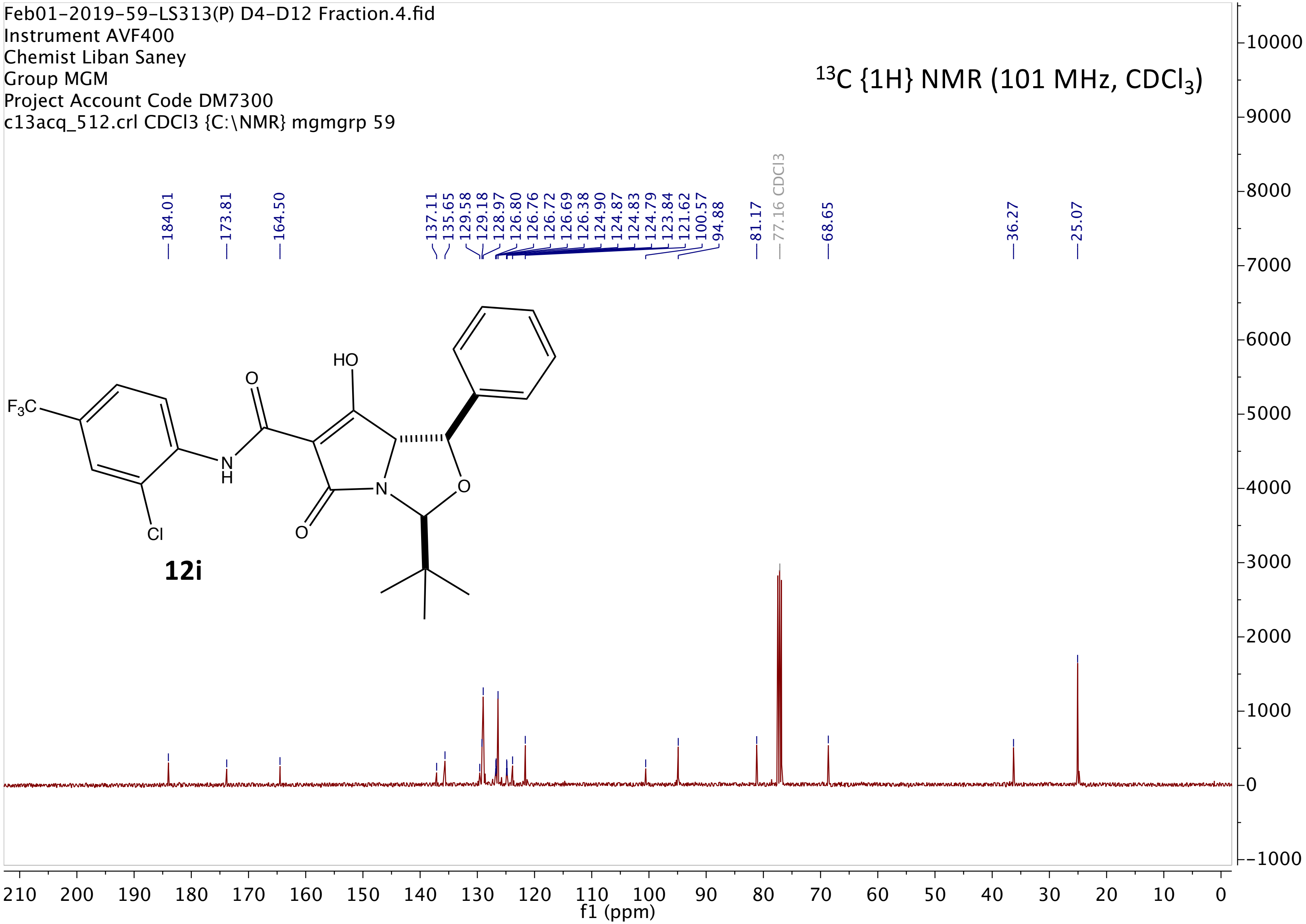
Feb01-2019-59-LS313(P) D4-D12 Fraction.4.fid
Instrument AVF400
Chemist Liban Saney
Group MGM
Project Account Code DM7300
c13acq_512.crl CDCl3 {C:\NMR} mgmgrp 59
^{13}C {1H} NMR (101 MHz, $CDCl_3$)
184.01
173.81
164.50
137.11
135.65
129.58
129.18
128.97
126.80
126.76
126.72
126.69
126.38
124.90
124.87
124.83
124.79
123.84
121.62
100.57
94.88
81.17
77.16 CDCl3
68.65
36.27
25.07
HO
O
F_3C
N
H
N
O
O
Cl
12i
10000
9000
8000
7000
6000
5000
4000
3000
2000
1000
0
-1000
210
200
190
180
170
160
150
140
130
120
110
100
90
80
70
60
50
40
30
20
10
0
f1 (ppm)

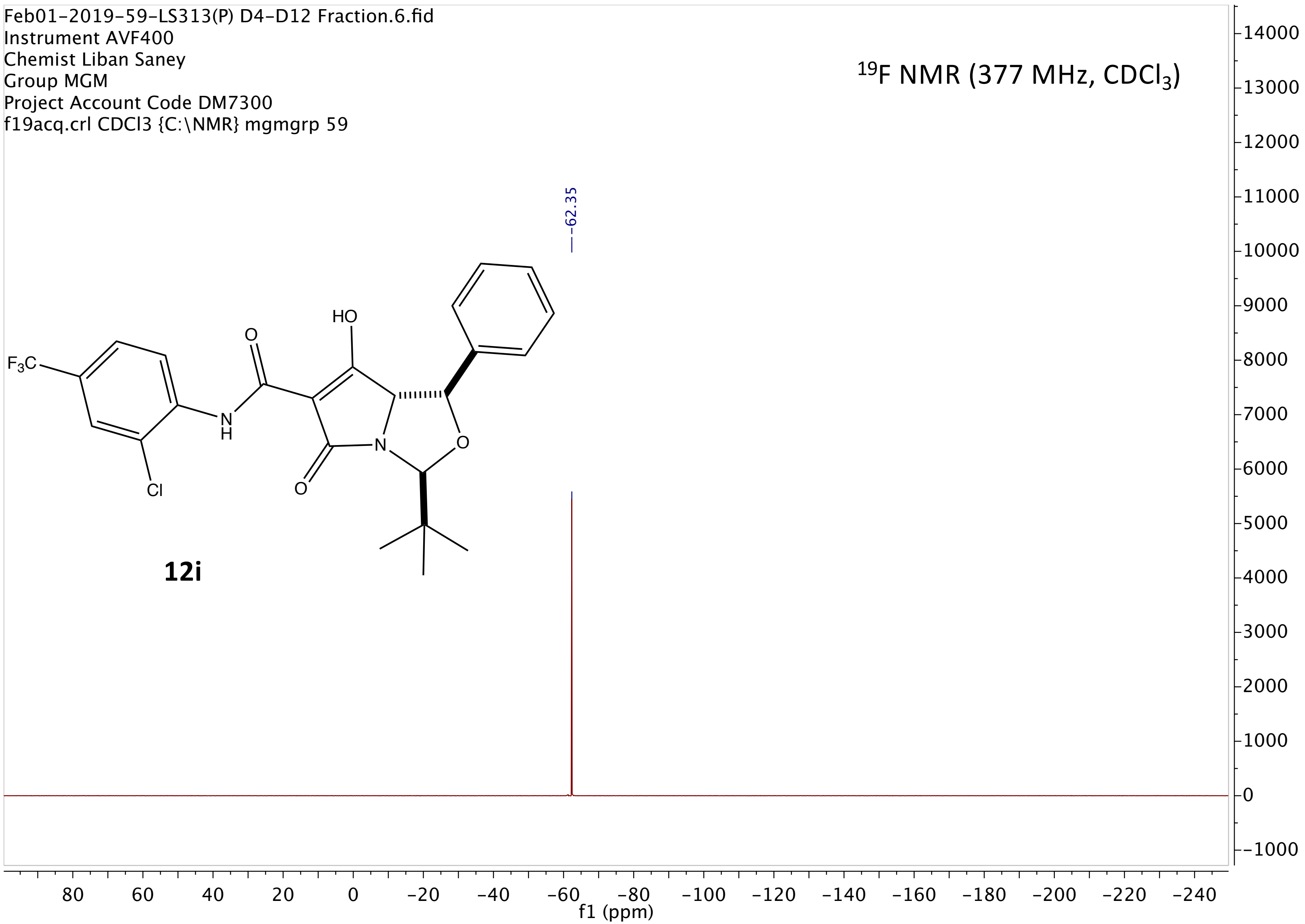
Feb01-2019-59-LS313(P) D4-D12 Fraction.6.fid
Instrument AVF400
Chemist Liban Saney
Group MGM
Project Account Code DM7300
f19acq.crl CDCl3 {C:\NMR} mgmgrp 59
^{19}F NMR (377 MHz, $CDCl_3$)
-62.35
F_3C
O
HO
N
H
Cl
O
N
O
12i
80 60 40 20 0 −20 −40 −60 −80 −100 −120 −140 −160 −180 −200 −220 −240
f1 (ppm)
14000 13000 12000 11000 10000 9000 8000 7000 6000 5000 4000 3000 2000 1000 0 −1000

Feb07-2019-59-LS316(P) G1-J1 Fraction.1.fid
Instrument AVF400
Chemist Liban Saney
Group MGM
Project Account Code DM7300
h1acq.crl CDCl3 {C:\NMR} mgmgrp 59

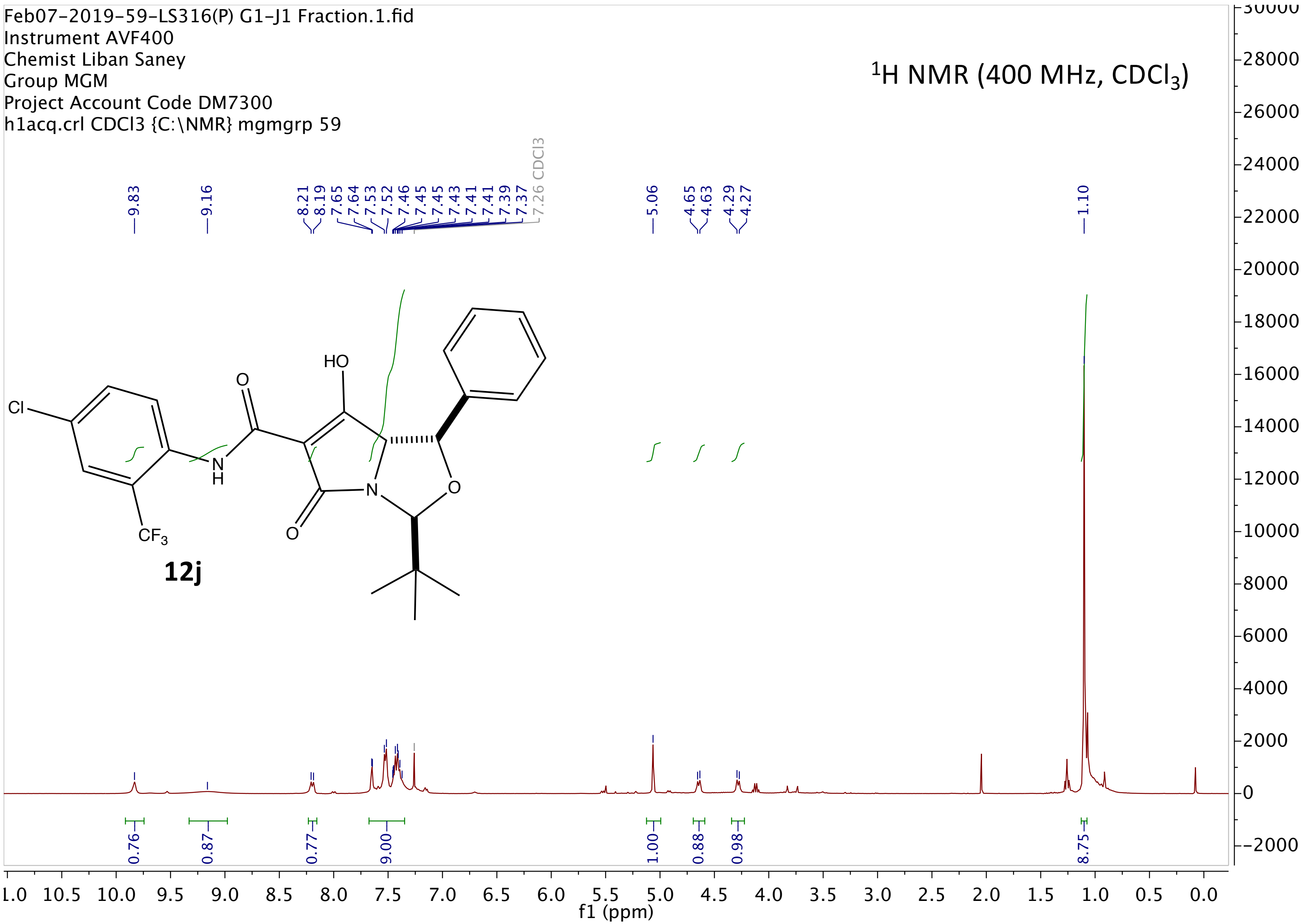

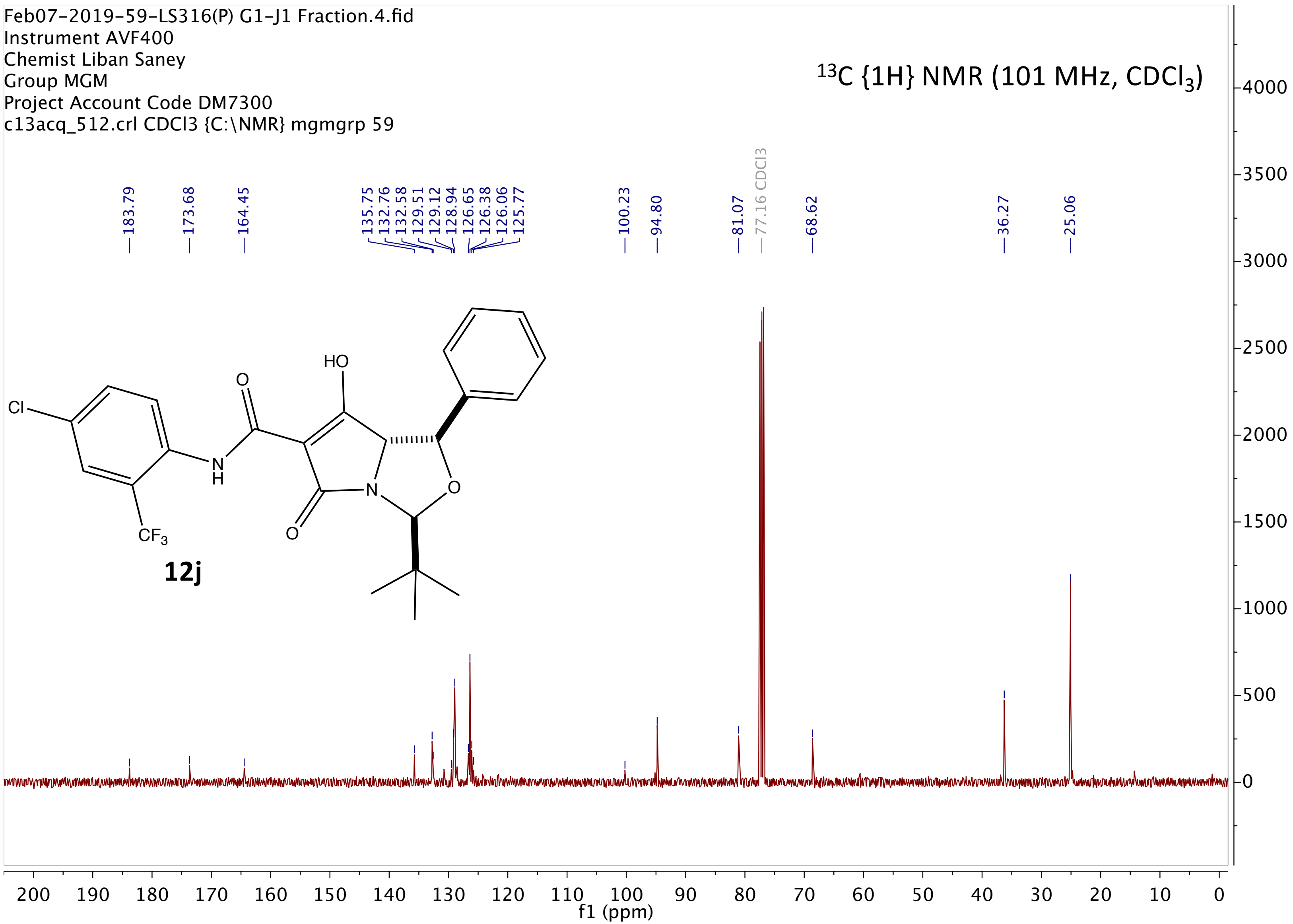
Feb07-2019-59-LS316(P) G1-J1 Fraction.4.fid
Instrument AVF400
Chemist Liban Saney
Group MGM
Project Account Code DM7300
c13acq_512.crl CDCl3 {C:\NMR} mgmgrp 59
13C {1H} NMR (101 MHz, CDCl3)
183.79
173.68
164.45
135.75
132.76
132.58
129.51
129.12
128.94
126.65
126.38
126.06
125.77
100.23
94.80
81.07
77.16 CDCl3
68.62
36.27
25.06
HO
O
Cl
N
H
N
O
O
CF3
12j
f1 (ppm)

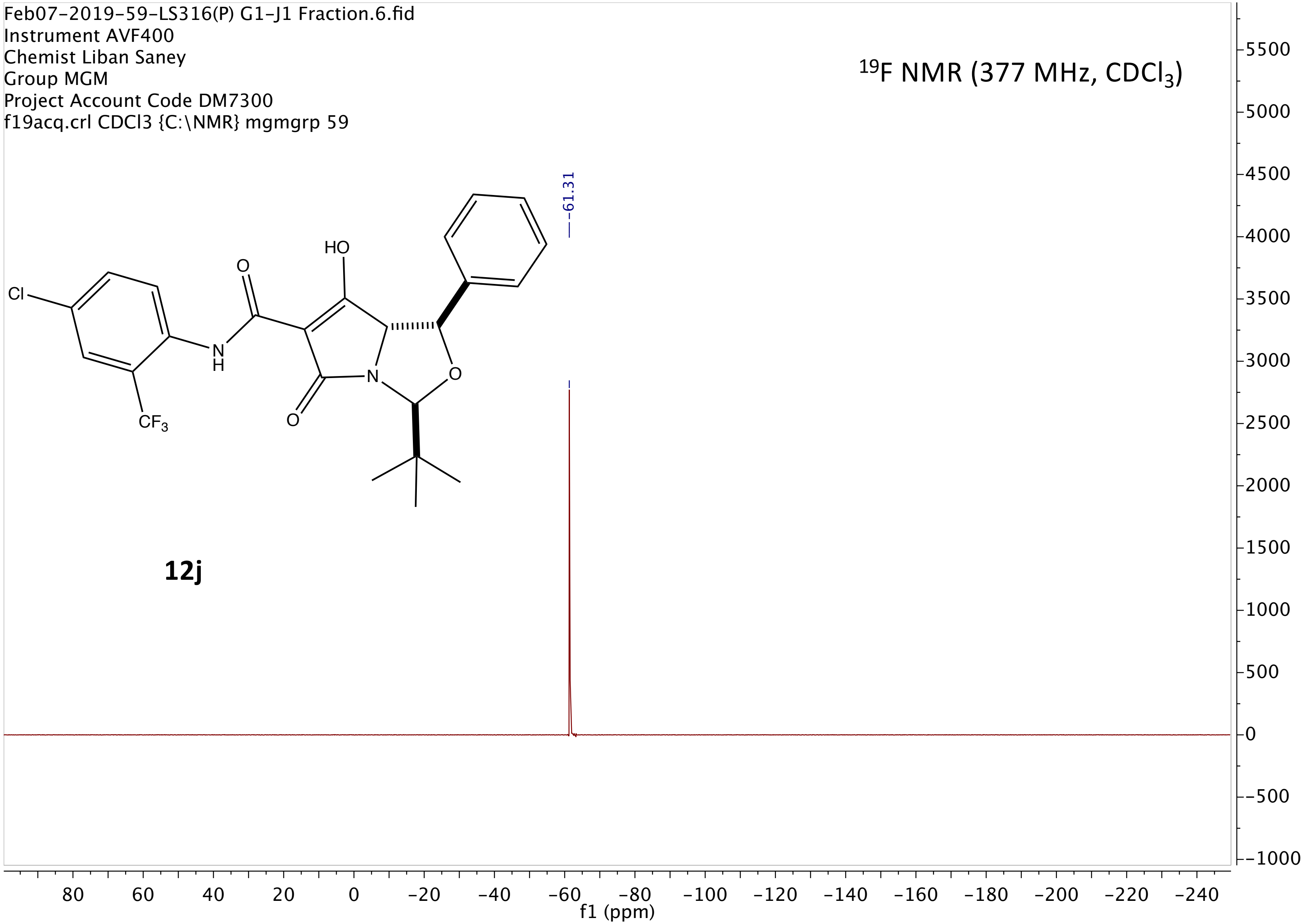
Feb07-2019-59-LS316(P) G1-J1 Fraction.6.fid
Instrument AVF400
Chemist Liban Saney
Group MGM
Project Account Code DM7300
f19acq.crl CDCl3 {C:\NMR} mgmgrp 59
19F NMR (377 MHz, CDCl3)
-61.31
HO
O
Cl
N
H
N
O
O
CF3
12j
f1 (ppm)

Feb25-2019-53-LS329(P) B1-B9 (Solid).1.fid
Instrument AVF400
Chemist Liban Saney
Group MGM
Project Account Code DM7300
h1acq.crl CDCl3 {C:\NMR} mgmgrp 53

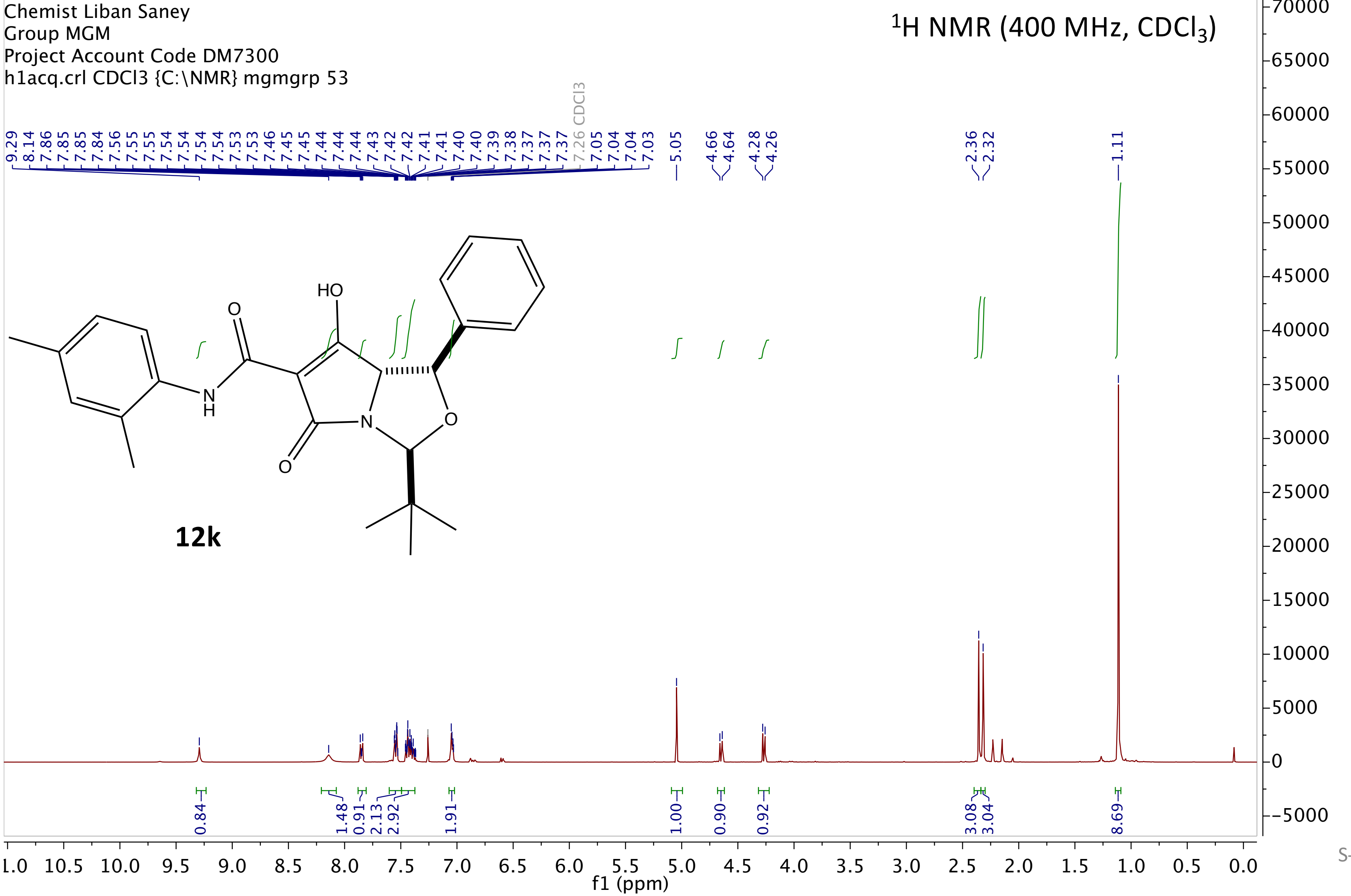

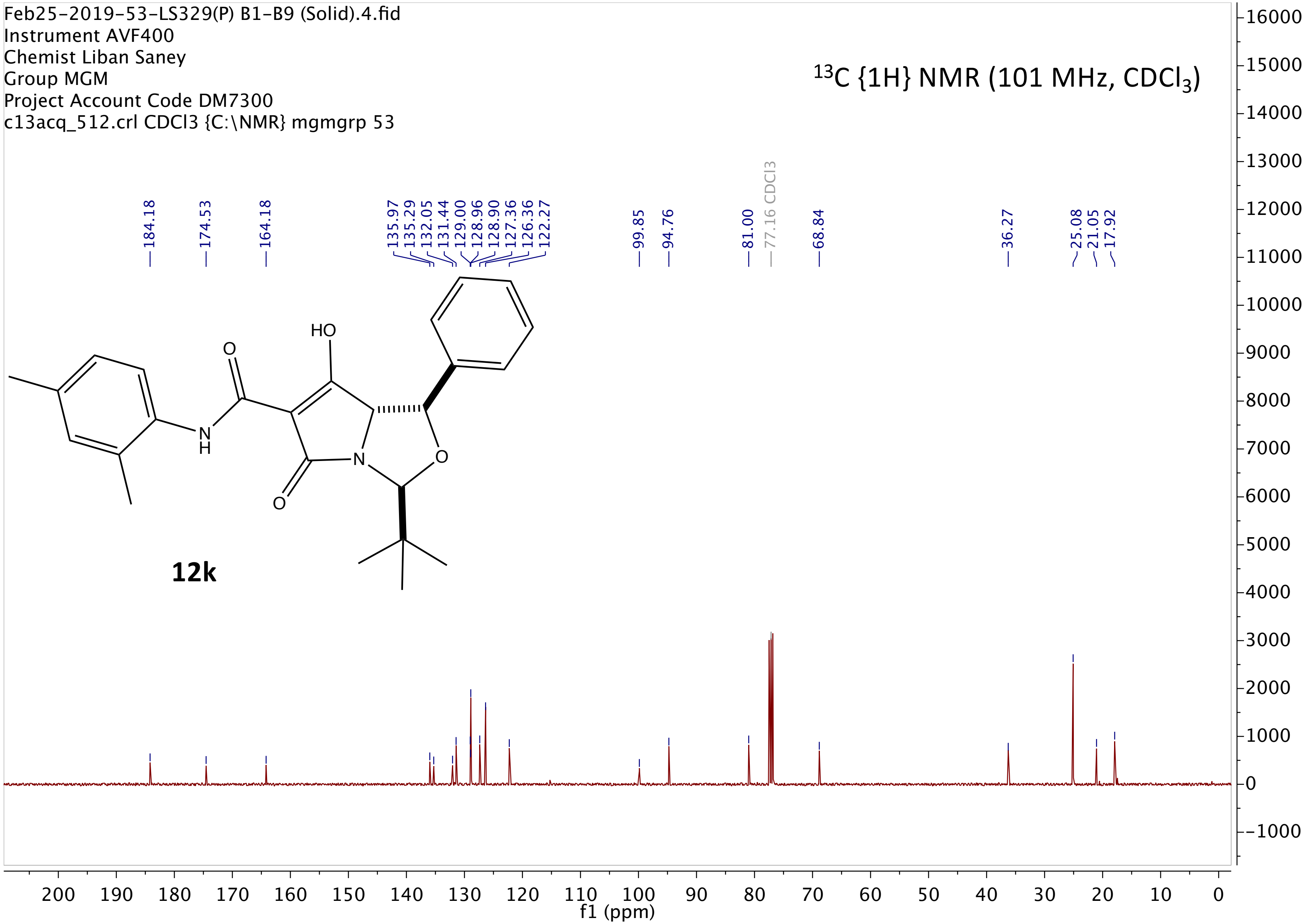

Feb25-2019-53-LS329(P) B1-B9 (Solid).4.fid
Instrument AVF400
Chemist Liban Saney
Group MGM
Project Account Code DM7300
c13acq_512.crl CDCl3 {C:\NMR} mgmgrp 53
^{13}C {1H} NMR (101 MHz, $CDCl_3$)
184.18
174.53
164.18
135.97
135.29
132.05
131.44
129.00
128.96
128.90
127.36
126.36
122.27
99.85
94.76
81.00
77.16 CDCl3
68.84
36.27
25.08
21.05
17.92
HO
O
N
H
N
O
O
12k
f1 (ppm)

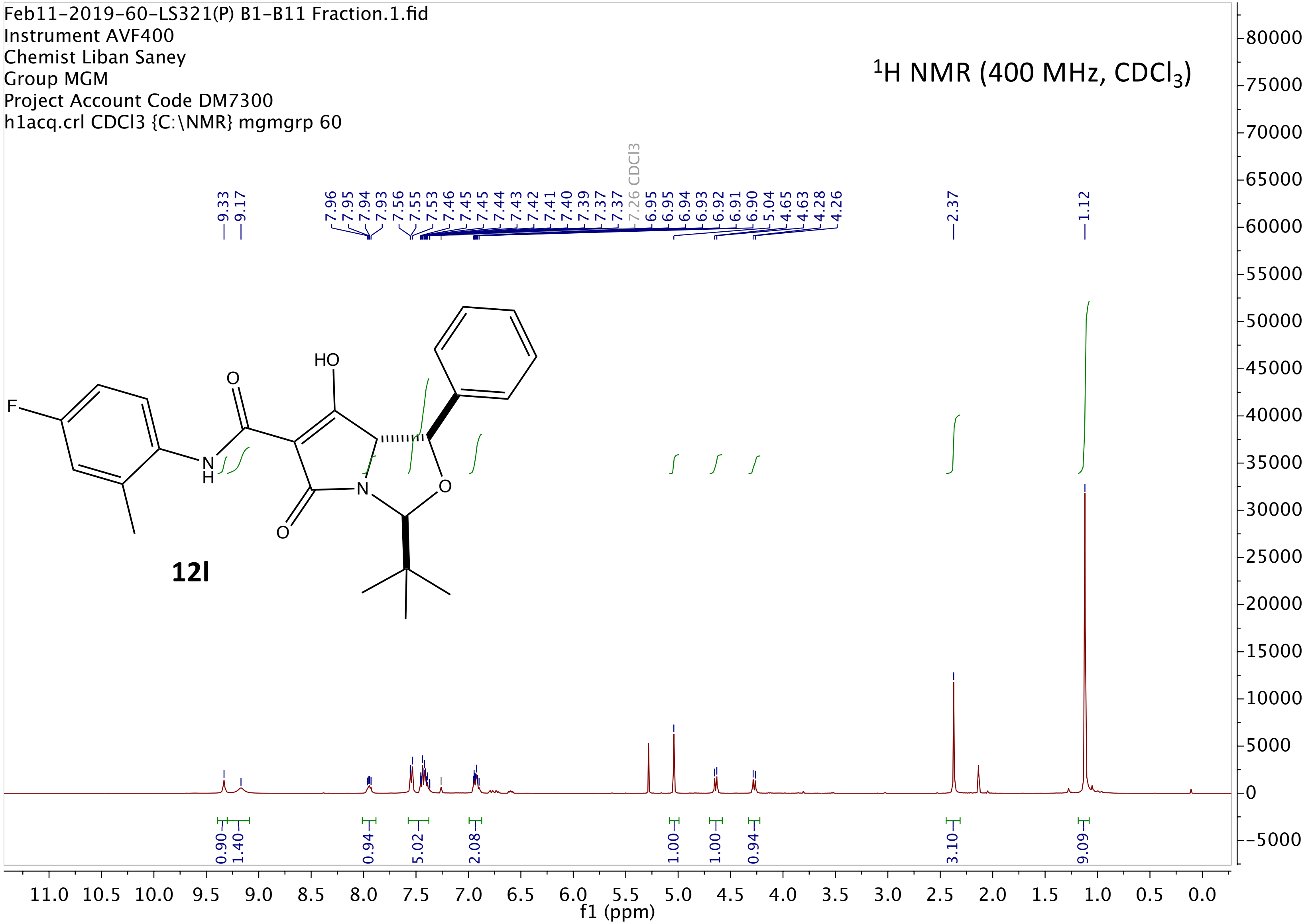
Feb11-2019-60-LS321(P) B1-B11 Fraction.1.fid
Instrument AVF400
Chemist Liban Saney
Group MGM
Project Account Code DM7300
h1acq.crl CDCl3 {C:\NMR} mgmgrp 60
1H NMR (400 MHz, CDCl3)
9.33
9.17
7.96
7.95
7.94
7.93
7.56
7.55
7.53
7.46
7.45
7.45
7.44
7.43
7.42
7.41
7.40
7.39
7.37
7.37
7.26 CDCl3
6.95
6.95
6.94
6.93
6.92
6.91
6.90
5.04
4.65
4.63
4.28
4.26
2.37
1.12
HO
O
F
N
H
N
O
O
12l
0.90
1.40
0.94
5.02
2.08
1.00
1.00
0.94
3.10
9.09
f1 (ppm)

Feb11-2019-60-LS321(P) B1-B11 Fraction.4.fid
Instrument AVF400
Chemist Liban Saney
Group MGM
Project Account Code DM7300
c13acq_512.crl CDCl3 {C:\NMR} mgmgrp 60

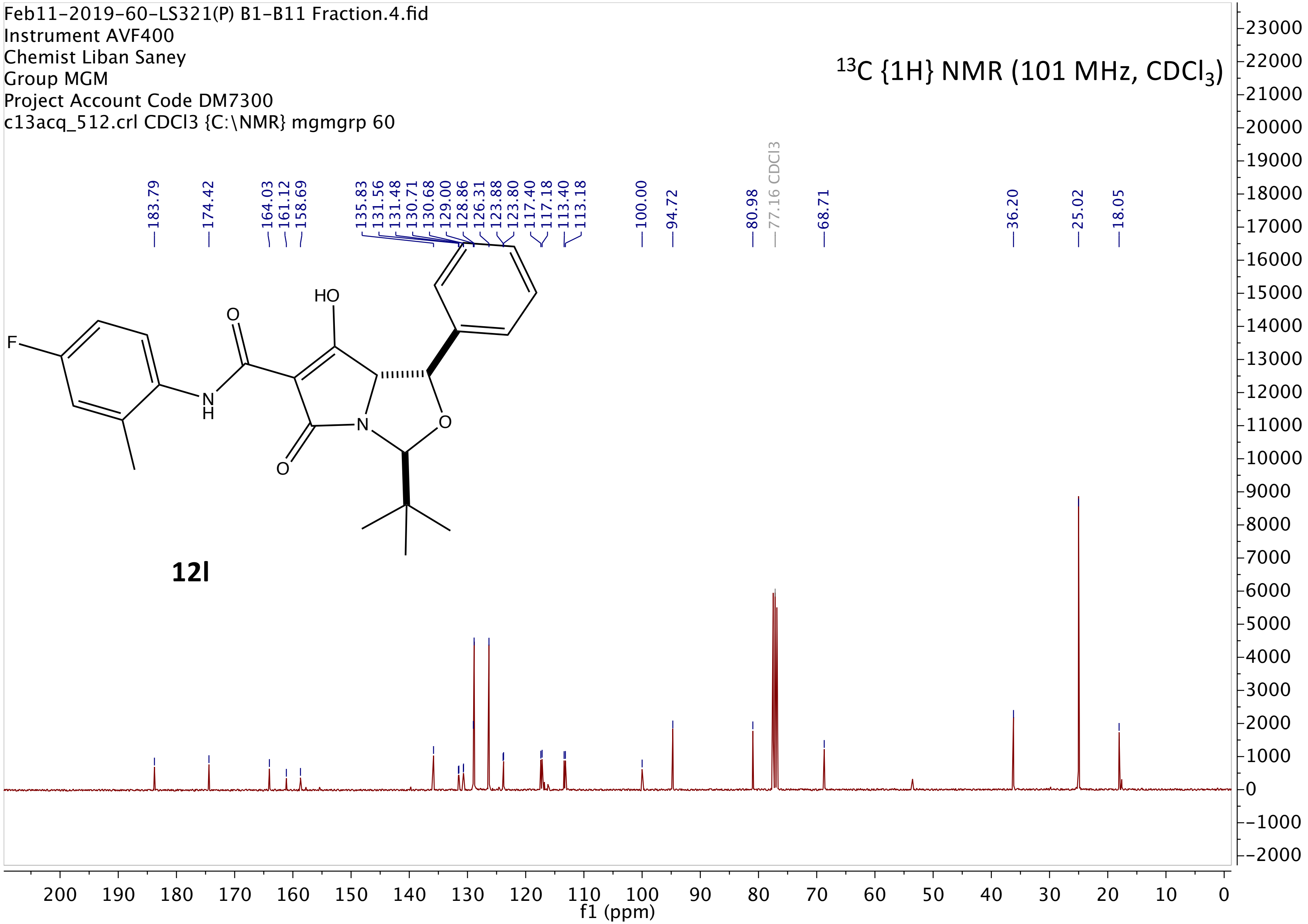

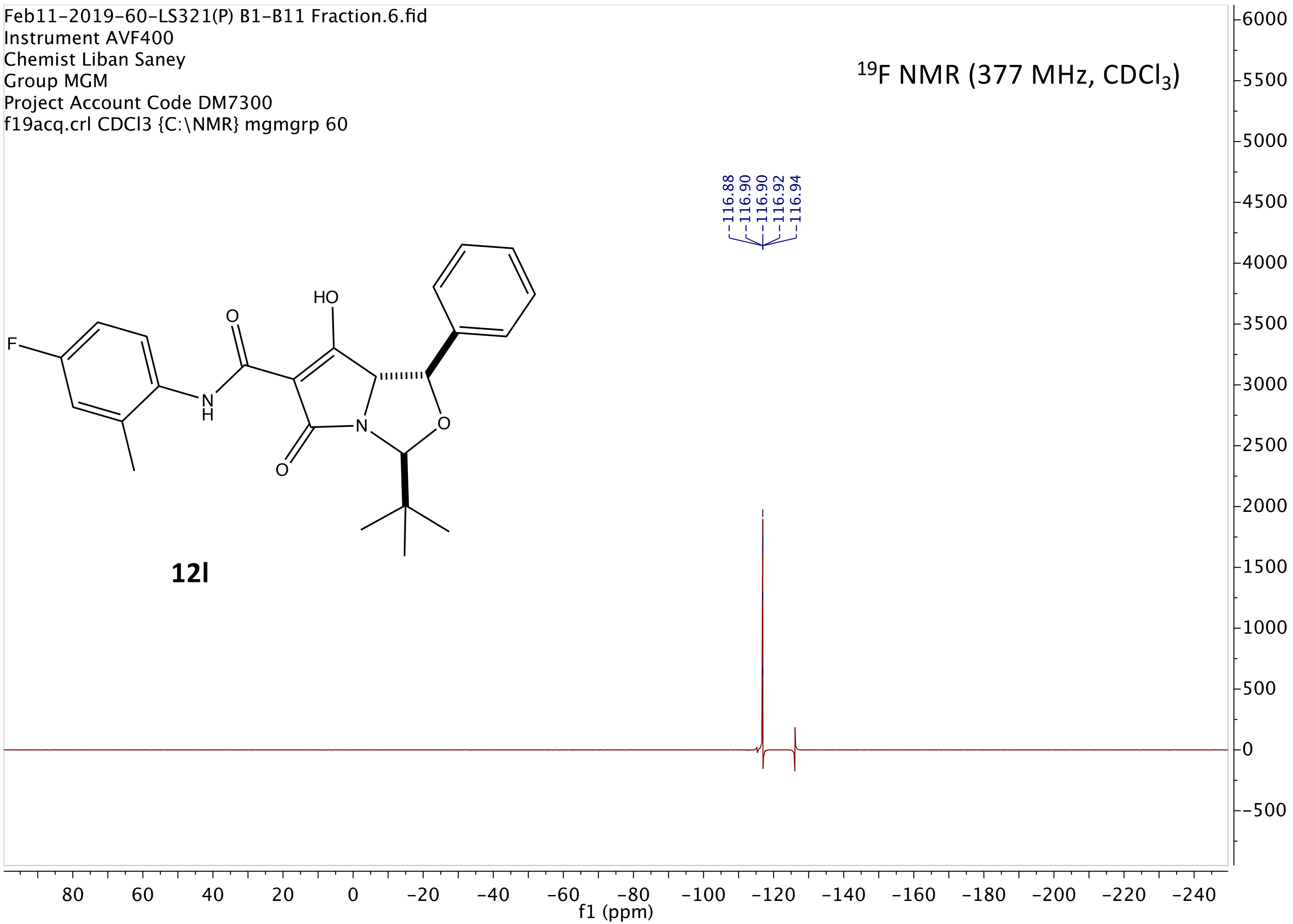
Feb11-2019-60-LS321(P) B1-B11 Fraction.6.fid
Instrument AVF400
Chemist Liban Saney
Group MGM
Project Account Code DM7300
f19acq.crl CDCl3 {C:\NMR} mgmgrp 60
19F NMR (377 MHz, CDCl3)
-116.88
-116.90
-116.90
-116.92
-116.94
F
O
HO
N
H
N
O
O
12l
f1 (ppm)

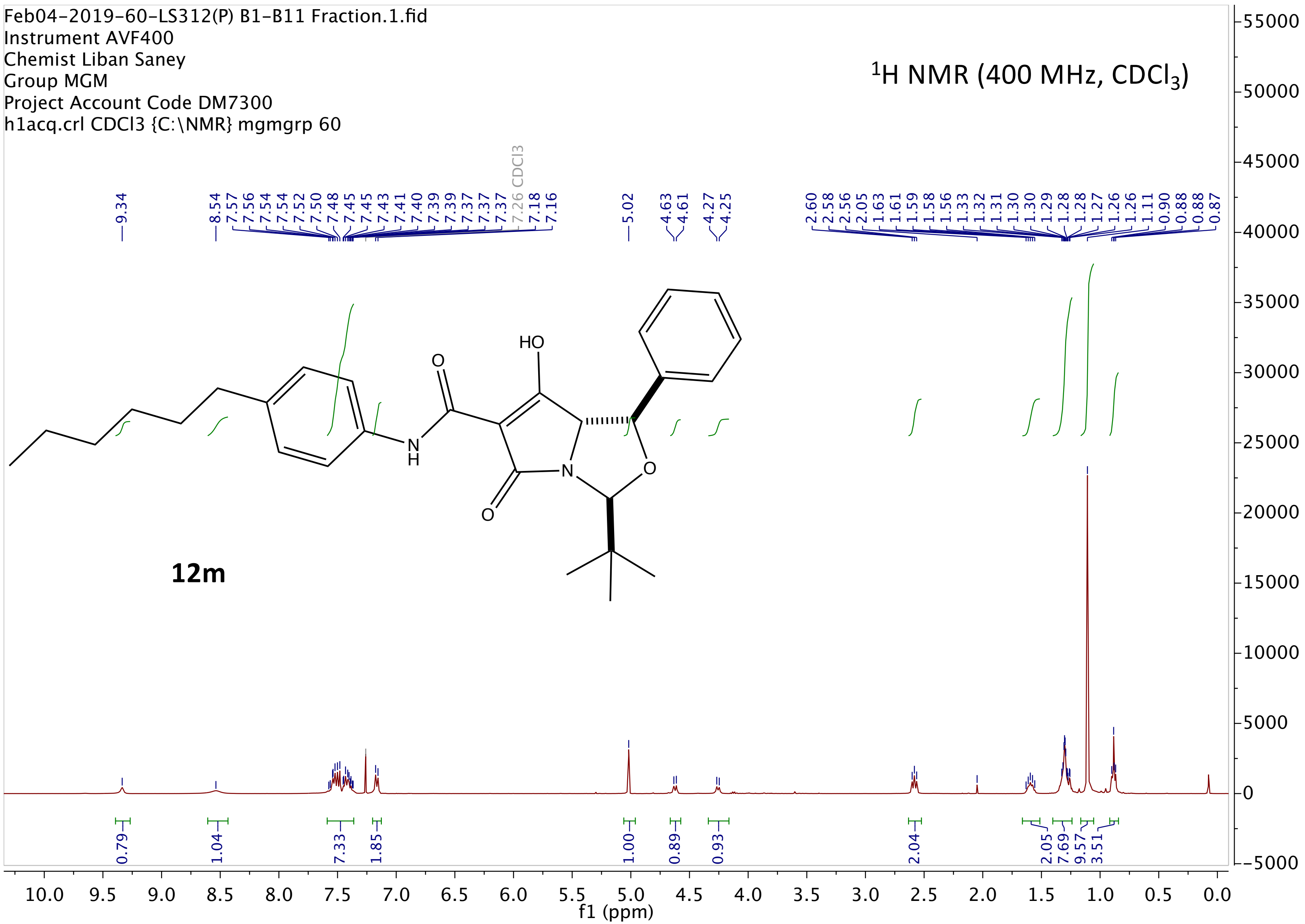

Feb04-2019-60-LS312(P) B1-B11 Fraction.1.fid
Instrument AVF400
Chemist Liban Saney
Group MGM
Project Account Code DM7300
h1acq.crl CDCl3 {C:\NMR} mgmgrp 60
^{1}H NMR (400 MHz, $CDCl_3$)
9.34
8.54
7.57 7.56 7.54 7.54 7.52 7.50 7.48 7.45 7.45 7.43 7.41 7.40 7.39 7.39 7.37 7.37 7.37
7.26 CDCl3
7.18 7.16
5.02
4.63 4.61
4.27 4.25
2.60 2.58 2.56 2.05 1.63 1.61 1.59 1.58 1.56 1.33 1.32 1.31 1.30 1.30 1.29 1.28 1.28 1.27 1.26 1.26 1.11 0.90 0.88 0.88 0.87
HO
O
O
N
H
N
O
12m
0.79
1.04
7.33
1.85
1.00
0.89
0.93
2.04
2.05
7.69
9.57
3.51
f1 (ppm)

Feb04-2019-60-LS312(P) B1-B11 Fraction.4.fid
Instrument AVF400
Chemist Liban Saney
Group MGM
Project Account Code DM7300
c13acq_512.crl CDCl3 {C:\NMR} mgmgrp 60

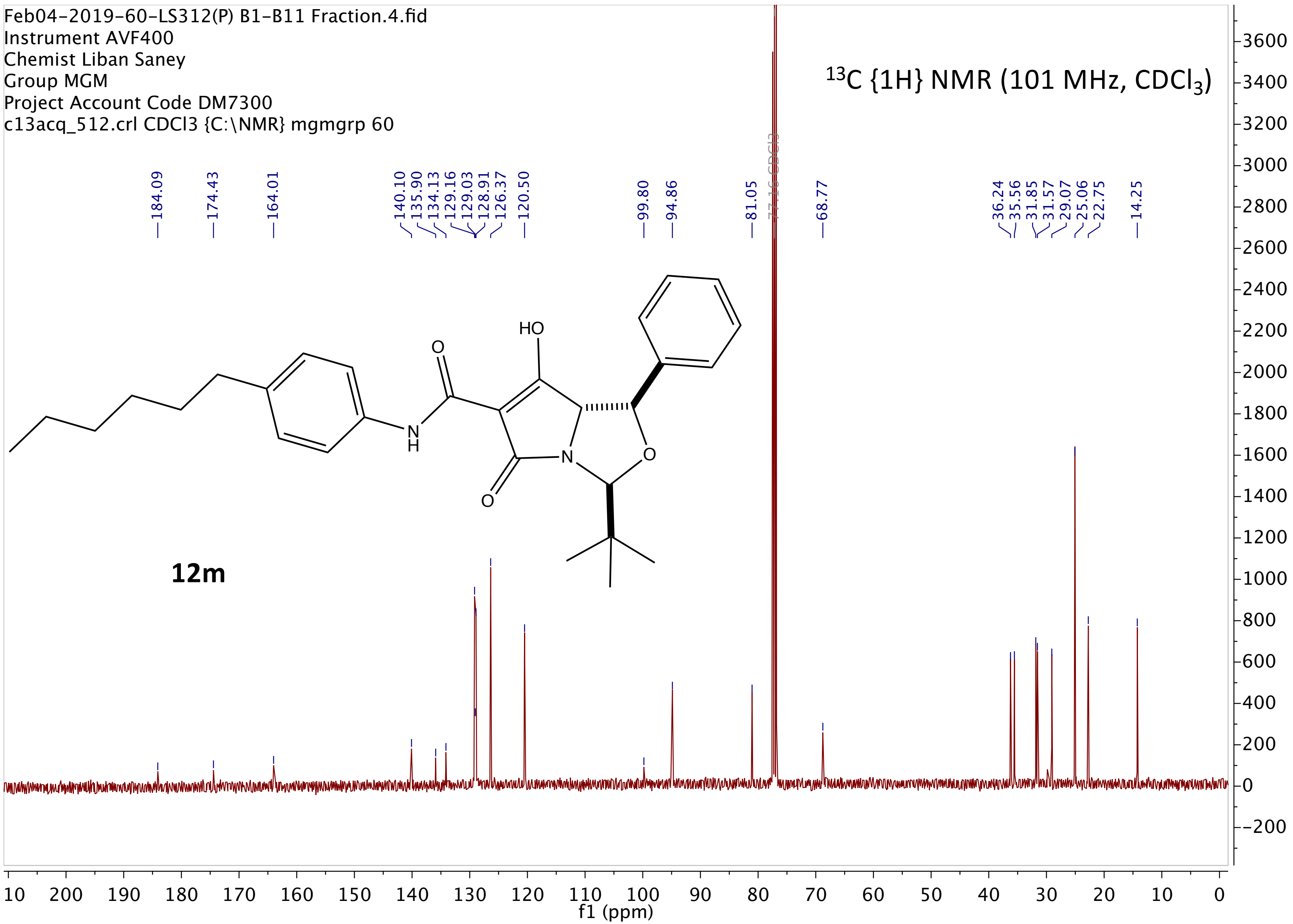

Dec12-2018-56-LS255(P) C4-C9 Fraction.1.fid
Instrument AVF400
Chemist Liban Saney
Group MGM
Project Account Code DM7300
h1acq.crl CDCl3 {C:\NMR} mgmgrp 56

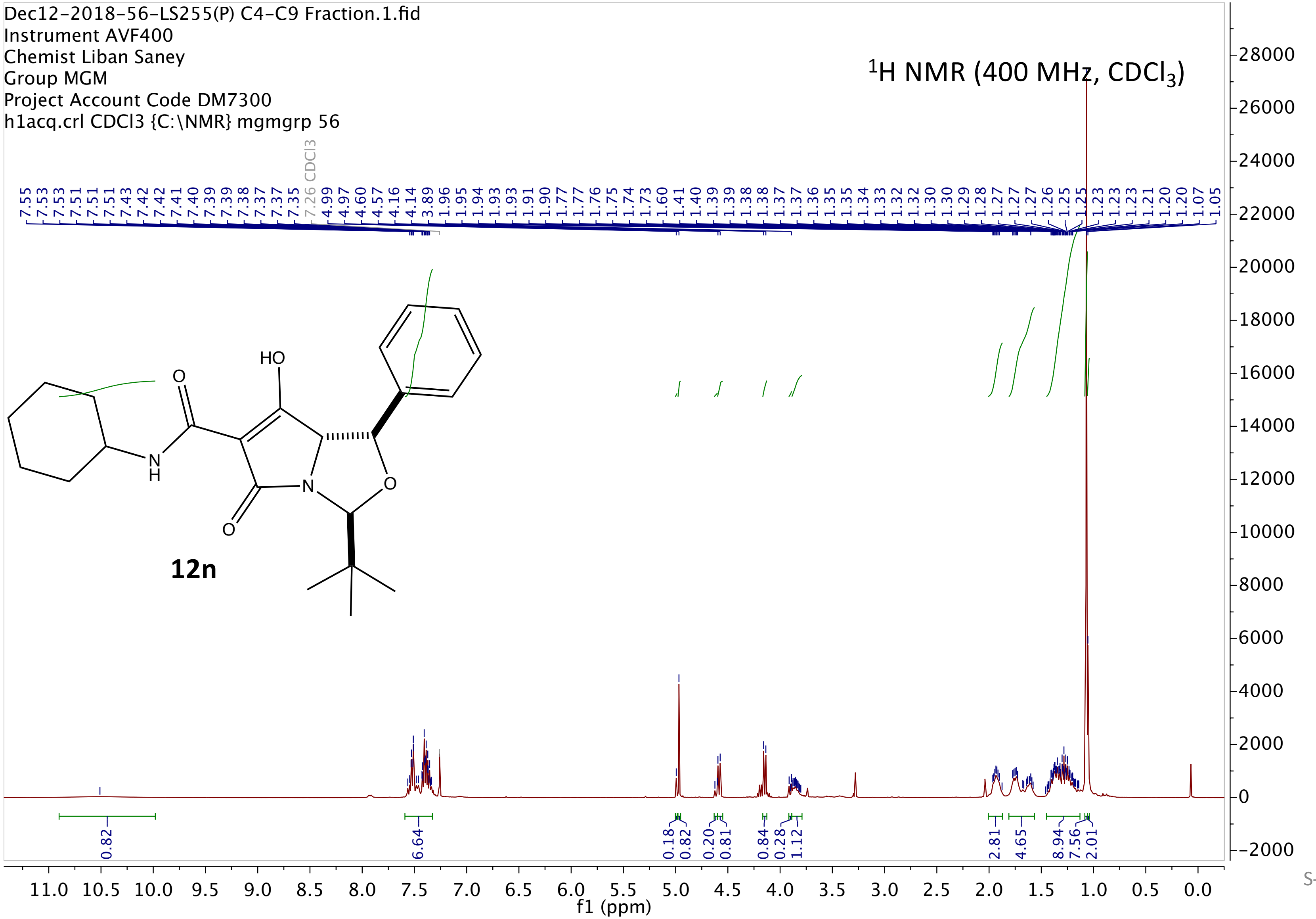

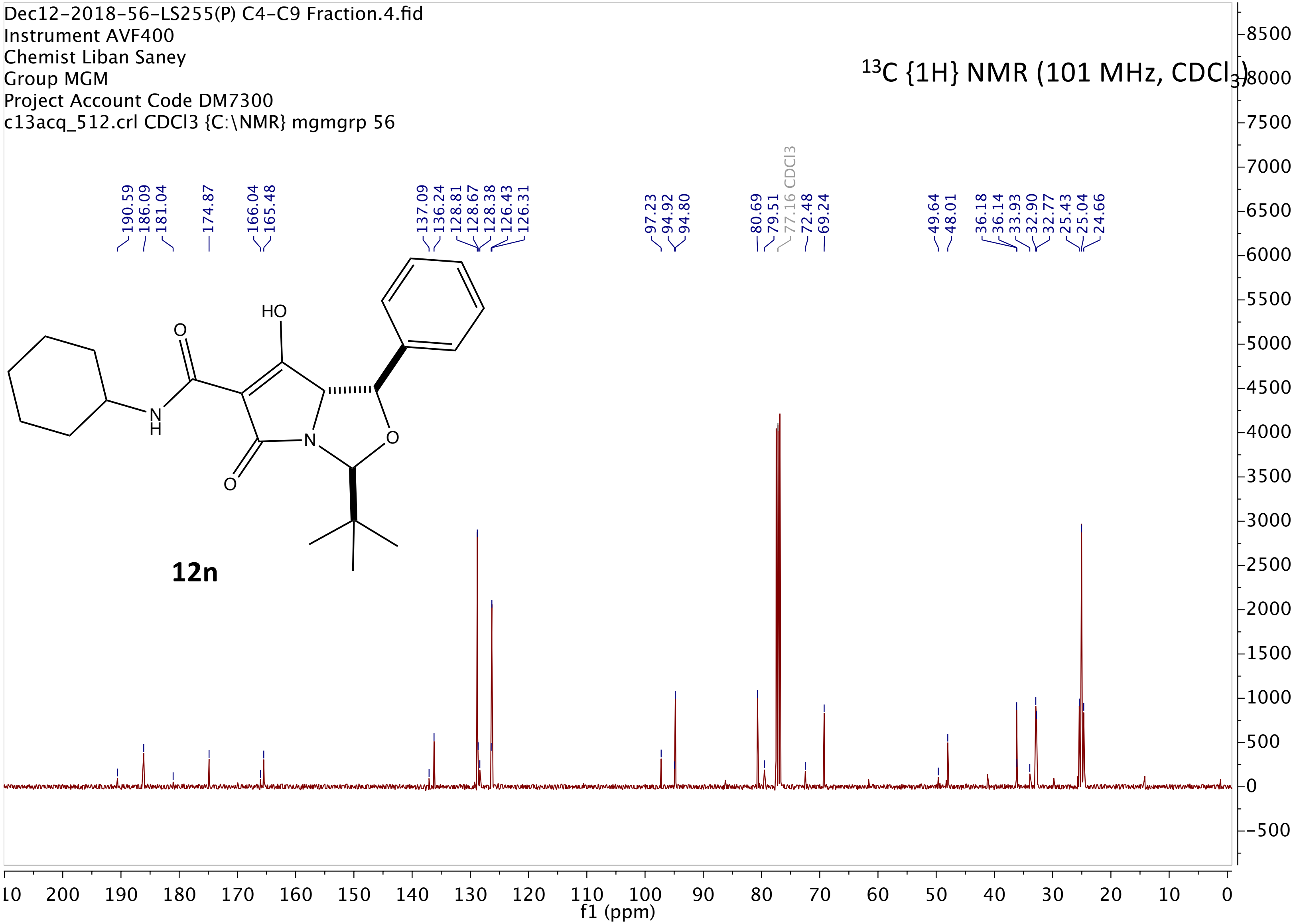
Dec12-2018-56-LS255(P) C4-C9 Fraction.4.fid
Instrument AVF400
Chemist Liban Saney
Group MGM
Project Account Code DM7300
c13acq_512.crl CDCl3 {C:\NMR} mgmgrp 56
13C {1H} NMR (101 MHz, CDCl3)
190.59
186.09
181.04
174.87
166.04
165.48
137.09
136.24
128.81
128.67
128.38
126.43
126.31
97.23
94.92
94.80
80.69
79.51
77.16 CDCl3
72.48
69.24
49.64
48.01
36.18
36.14
33.93
32.90
32.77
25.43
25.04
24.66
HO
O
N
H
N
O
O
12n
f1 (ppm)

Dec27-2018-59-LS271(P) E1-E12 Fraction.1.fid
Instrument AVF400
Chemist Liban Saney
Group MGM
Project Account Code DM7300
h1acq.crl CDCl3 {C:\NMR} mgmgrp 59

^{1}H NMR (400 MHz, $CDCl_3$)

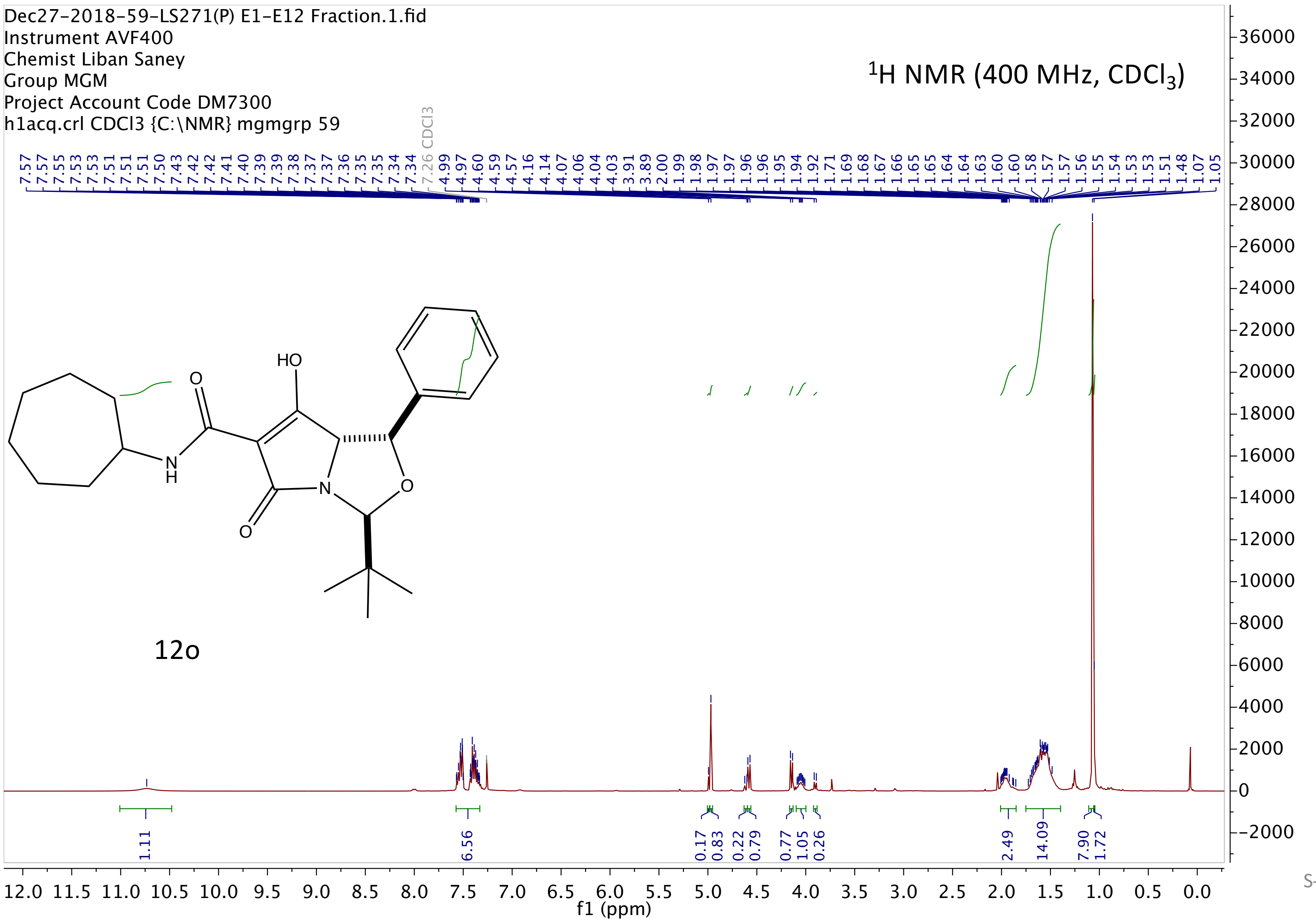

12o

Dec27-2018-59-LS271(P) E1-E12 Fraction.4.fid
Instrument AVF400
Chemist Liban Saney
Group MGM
Project Account Code DM7300
c13acq_512.crl CDCl3 {C:\NMR} mgmgrp 59

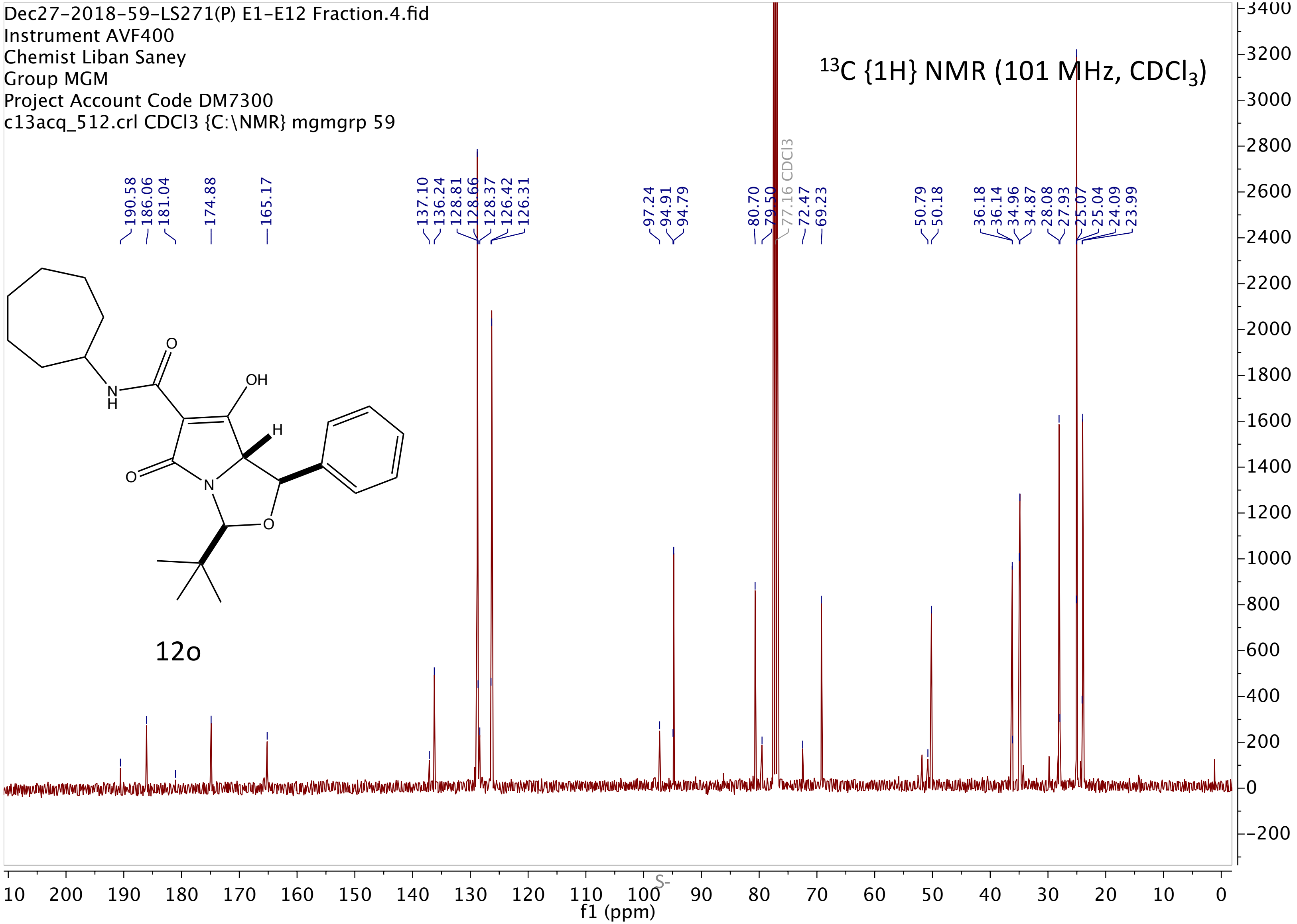

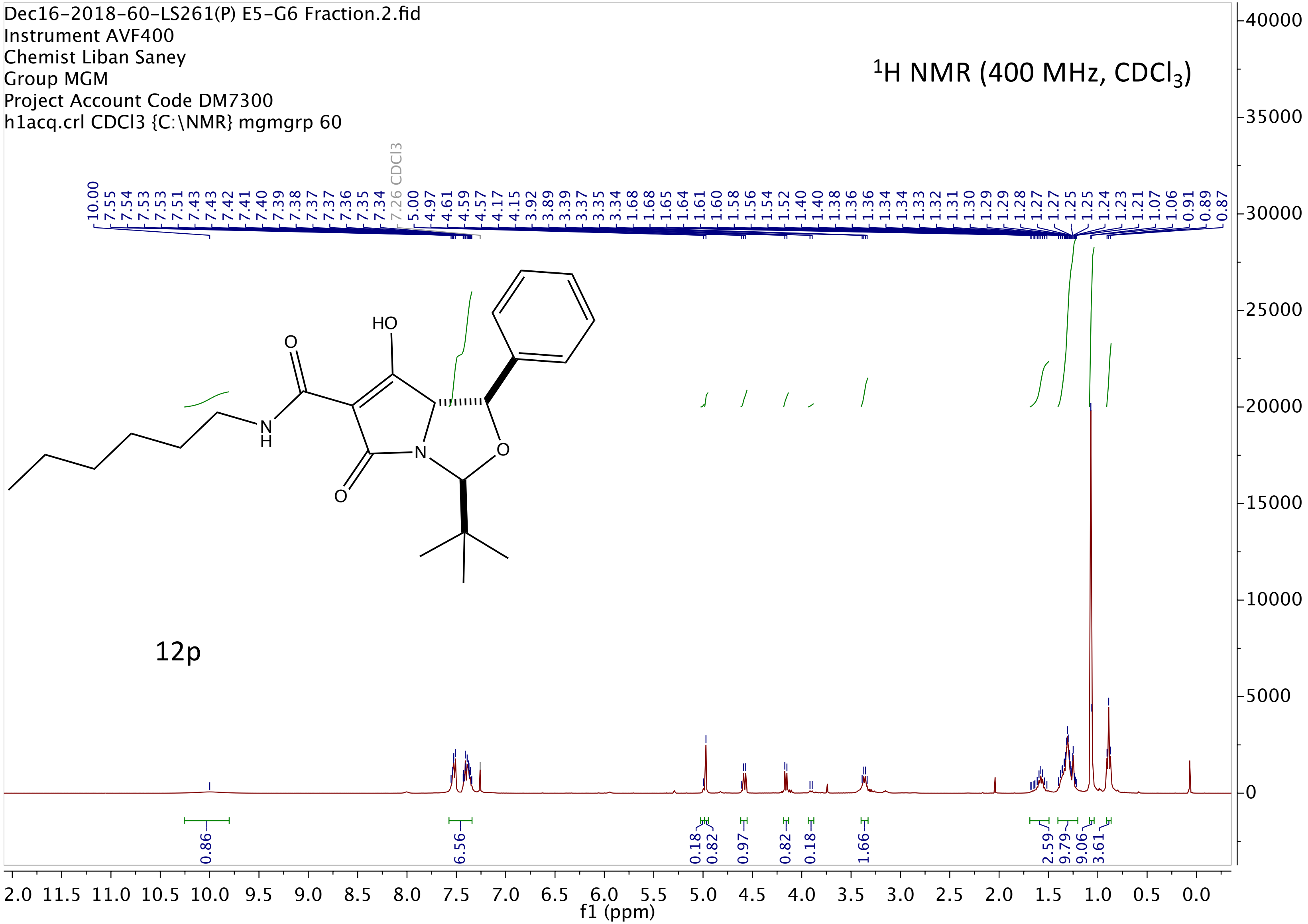

Dec16-2018-60-LS261(P) E5-G6 Fraction.2.fid
Instrument AVF400
Chemist Liban Saney
Group MGM
Project Account Code DM7300
h1acq.crl CDCl3 {C:\NMR} mgmgrp 60
^{1}H NMR (400 MHz, CDCl$_3$)
12p
f1 (ppm)

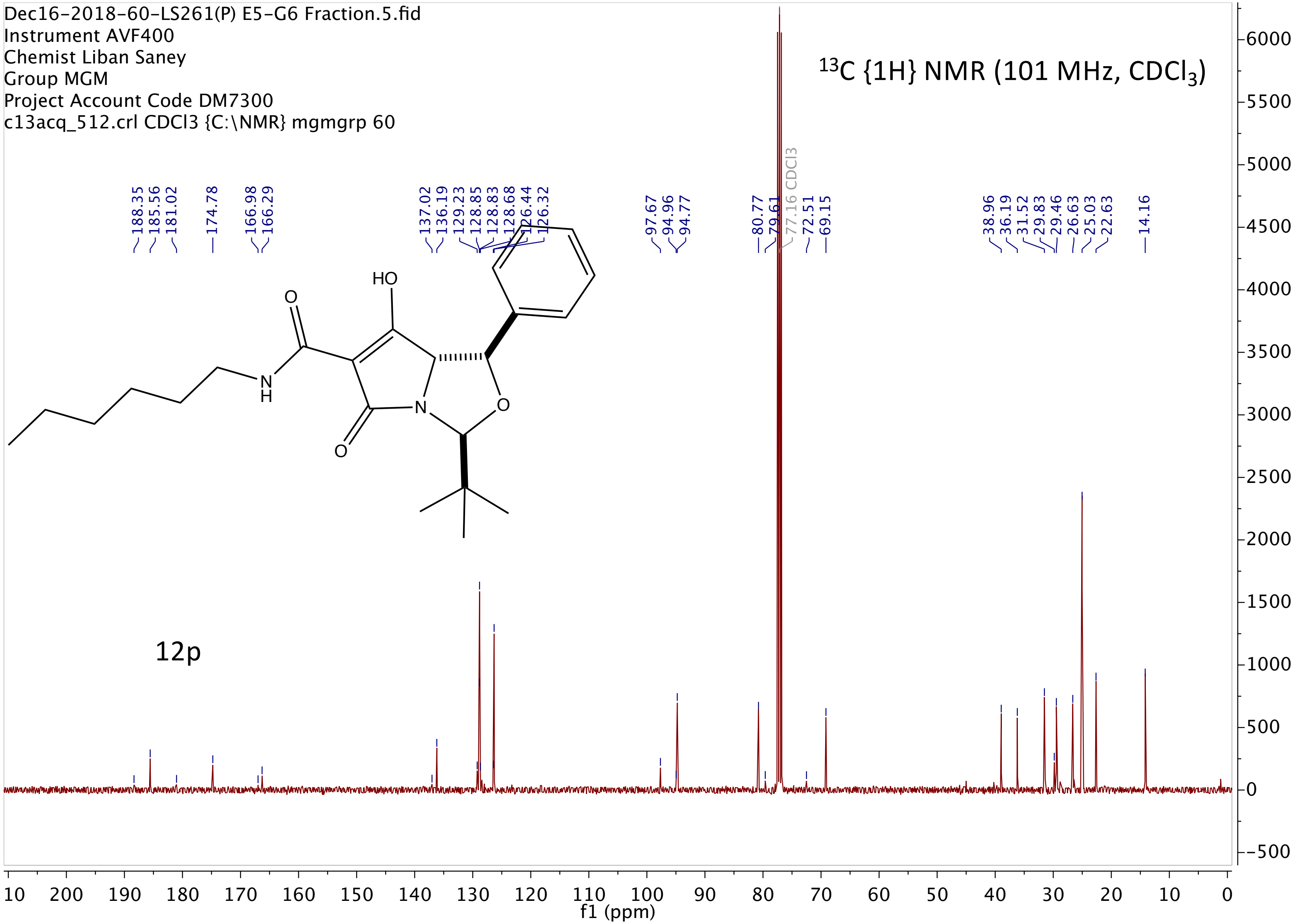

Dec16-2018-60-LS261(P) E5-G6 Fraction.5.fid
Instrument AVF400
Chemist Liban Saney
Group MGM
Project Account Code DM7300
c13acq_512.crl CDCl3 {C:\NMR} mgmgrp 60
13C {1H} NMR (101 MHz, CDCl3)
188.35
185.56
181.02
174.78
166.98
166.29
137.02
136.19
129.23
128.85
128.83
128.68
126.44
126.32
97.67
94.96
94.77
80.77
79.61
77.16 CDCl3
72.51
69.15
38.96
36.19
31.52
29.83
29.46
26.63
25.03
22.63
14.16
HO
O
N
H
N
O
O
12p
f1 (ppm)

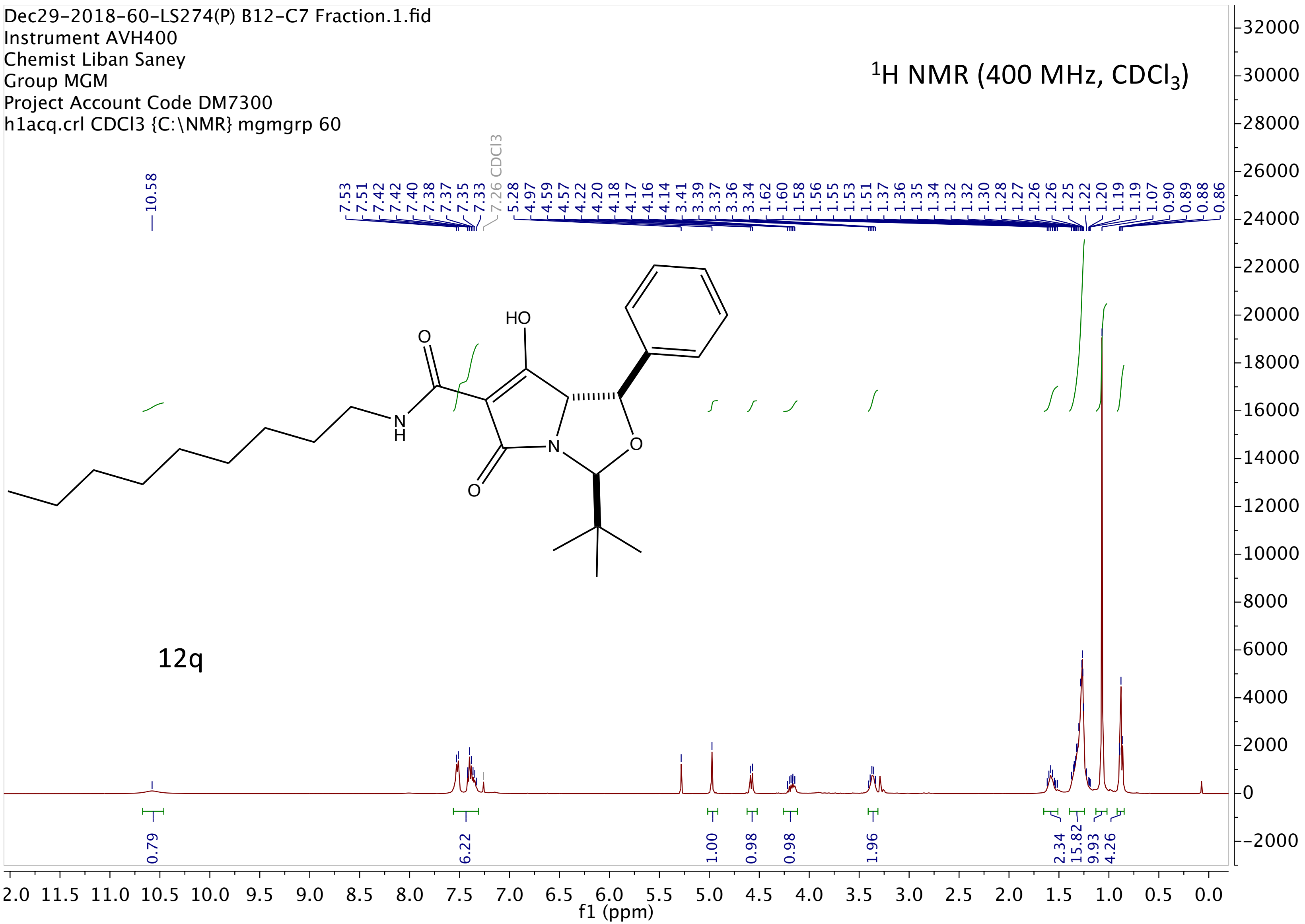
Dec29-2018-60-LS274(P) B12-C7 Fraction.1.fid
Instrument AVH400
Chemist Liban Saney
Group MGM
Project Account Code DM7300
h1acq.crl CDCl3 {C:\NMR} mgmgrp 60
^{1}H NMR (400 MHz, $CDCl_3$)
10.58
7.53 7.51 7.42 7.42 7.40 7.38 7.37 7.35 7.33 7.33
7.26 CDCl3
5.28 4.97 4.59 4.57 4.22 4.20 4.18 4.17 4.16 4.14 3.41 3.39 3.37 3.36 3.34 1.62 1.60 1.58 1.56 1.55 1.53 1.51 1.37 1.36 1.35 1.34 1.32 1.32 1.30 1.28 1.27 1.26 1.26 1.25 1.22 1.20 1.19 1.19 1.07 0.90 0.89 0.88 0.86
HO
O
N
H
N
O
O
12q
0.79
6.22
1.00
0.98
0.98
1.96
2.34
15.82
9.93
4.26
f1 (ppm)

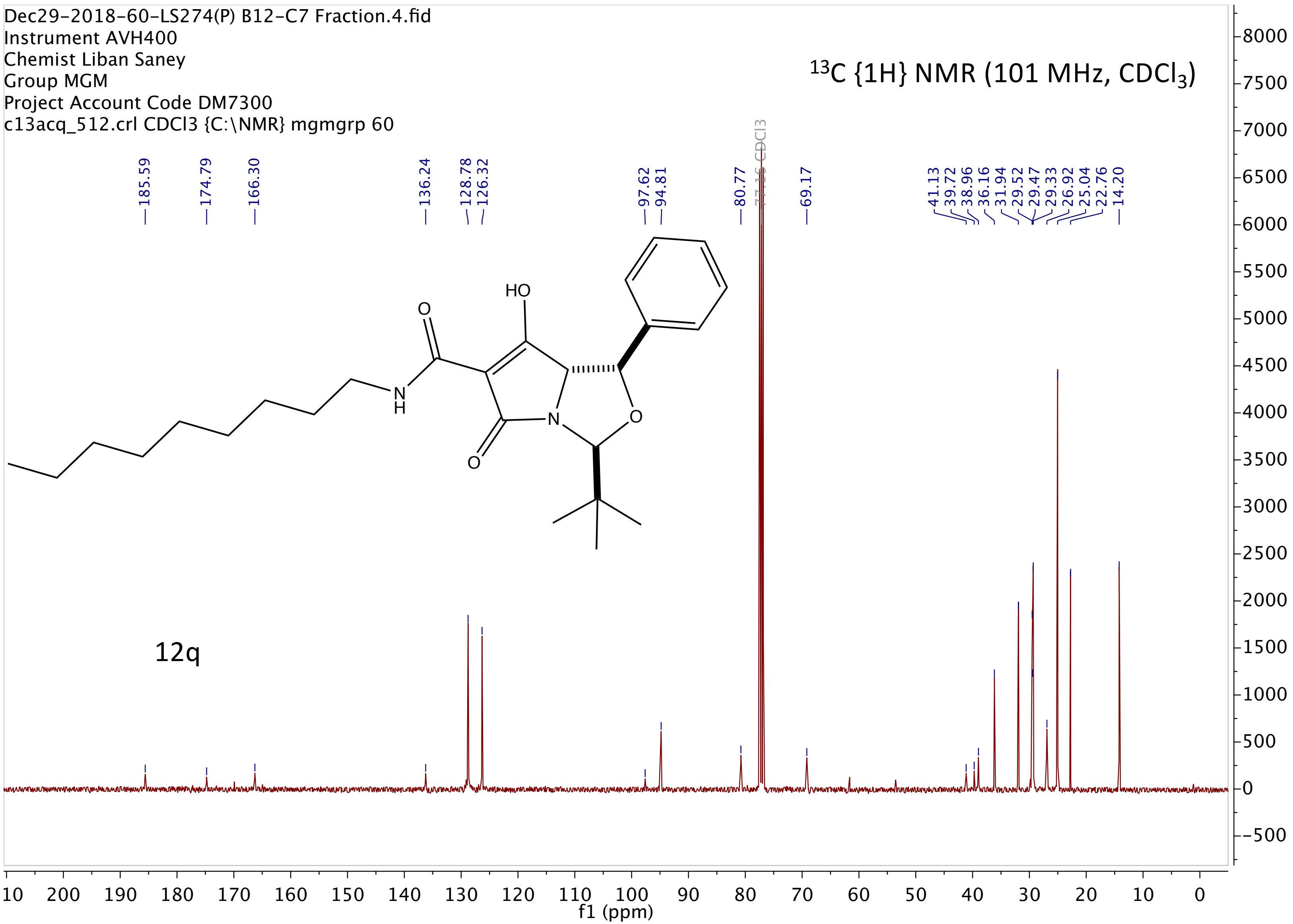
Dec29-2018-60-LS274(P) B12-C7 Fraction.4.fid
Instrument AVH400
Chemist Liban Saney
Group MGM
Project Account Code DM7300
c13acq_512.crl CDCl3 {C:\NMR} mgmgrp 60
13C {1H} NMR (101 MHz, CDCl3)
185.59
174.79
166.30
136.24
128.78
126.32
97.62
94.81
80.77
77.16 CDCl3
69.17
41.13
39.72
38.96
36.16
31.94
29.52
29.47
29.33
26.92
25.04
22.76
14.20
HO
O
N
H
O
O
12q
f1 (ppm)

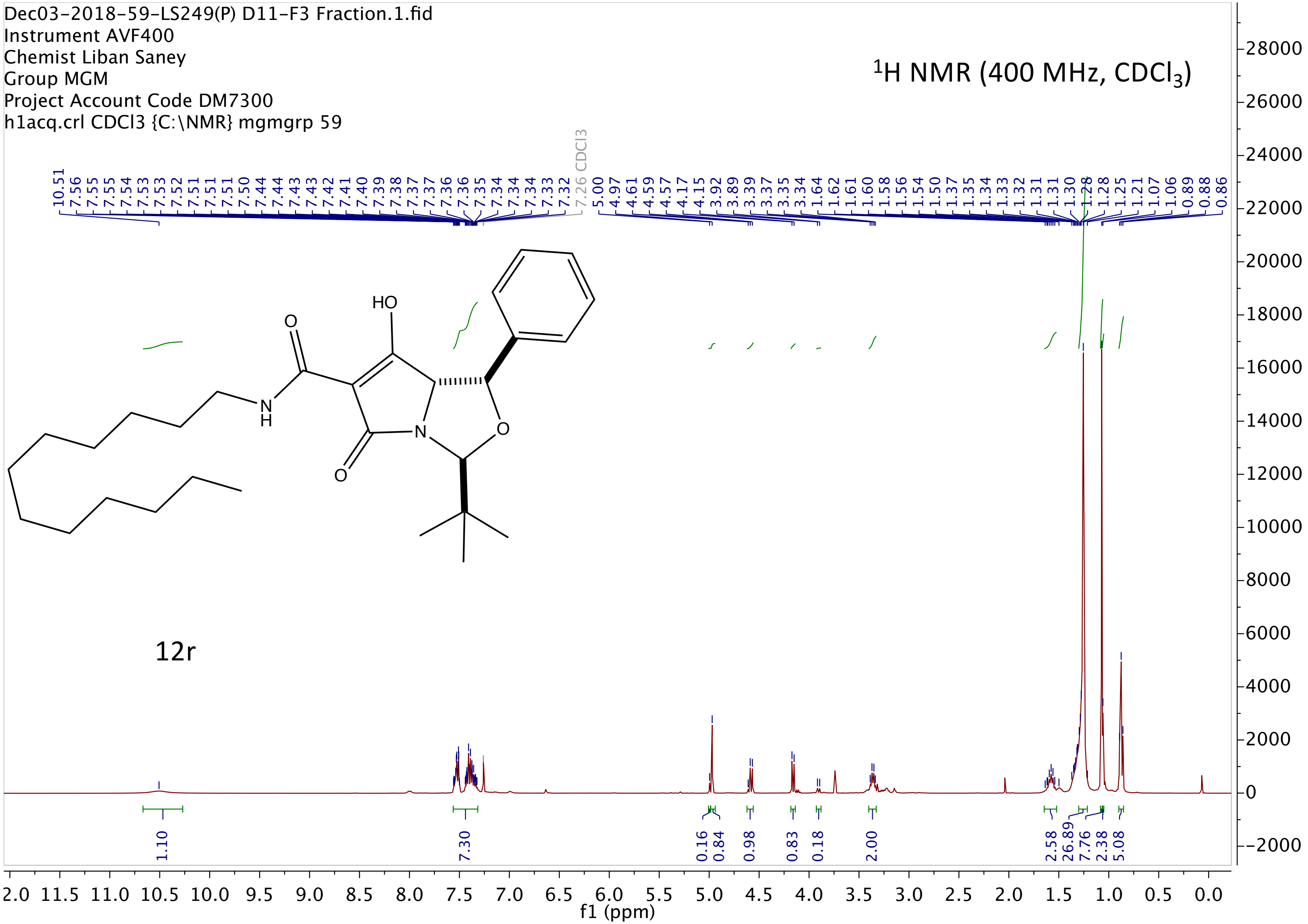
Dec03-2018-59-LS249(P) D11-F3 Fraction.1.fid
Instrument AVF400
Chemist Liban Saney
Group MGM
Project Account Code DM7300
h1acq.crl CDCl3 {C:\NMR} mgmgrp 59
1H NMR (400 MHz, CDCl3)
12r
HO
O
N
H
N
O
O
7.26 CDCl3
10.51
1.10
7.30
0.16
0.84
0.98
0.83
0.18
2.00
2.58
26.89
7.76
2.38
5.08
f1 (ppm)

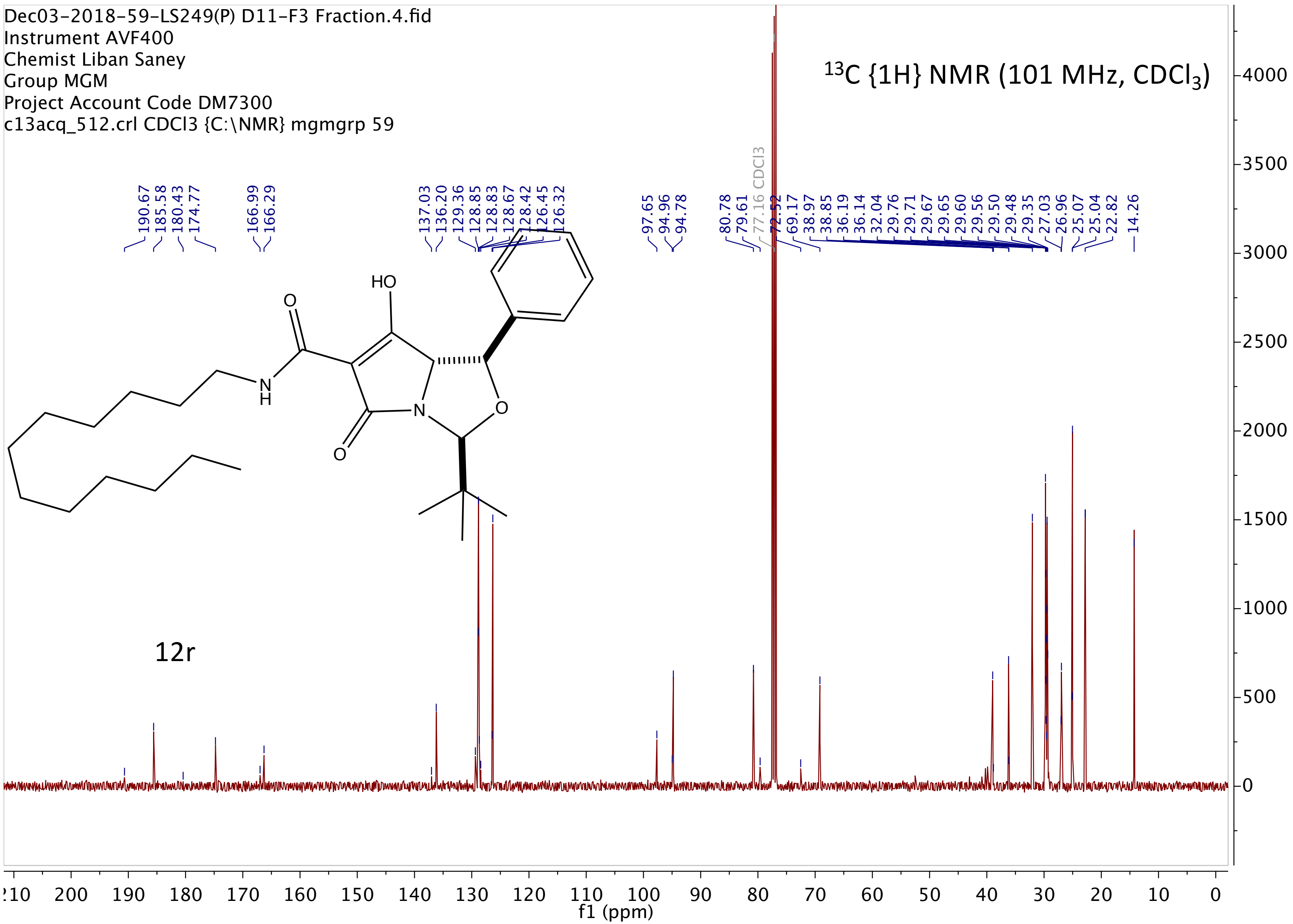
Dec03-2018-59-LS249(P) D11-F3 Fraction.4.fid
Instrument AVF400
Chemist Liban Saney
Group MGM
Project Account Code DM7300
c13acq_512.crl CDCl3 {C:\NMR} mgmgrp 59
13C {1H} NMR (101 MHz, CDCl3)
190.67
185.58
180.43
174.77
166.99
166.29
137.03
136.20
129.36
128.85
128.83
128.67
128.42
126.45
126.32
97.65
94.96
94.78
80.78
79.61
77.16 CDCl3
72.52
69.17
38.97
38.85
36.19
36.14
32.04
29.76
29.71
29.67
29.65
29.60
29.56
29.50
29.48
29.35
27.03
26.96
25.07
25.04
22.82
14.26
HO
O
N
H
N
O
O
12r
f1 (ppm)

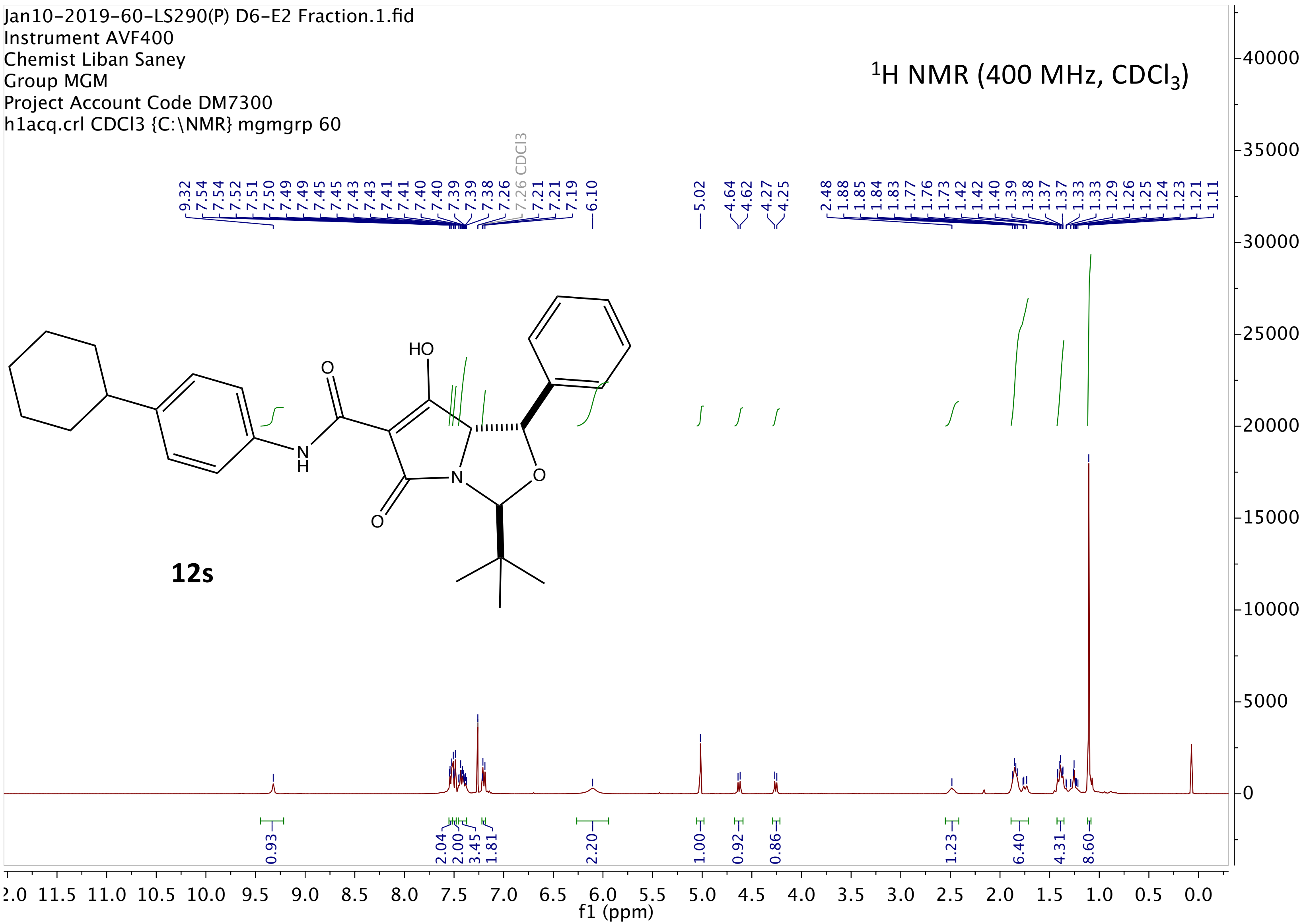
Jan10-2019-60-LS290(P) D6-E2 Fraction.1.fid
Instrument AVF400
Chemist Liban Saney
Group MGM
Project Account Code DM7300
h1acq.crl CDCl3 {C:\NMR} mgmgrp 60
1H NMR (400 MHz, CDCl3)
9.32 7.54 7.54 7.52 7.51 7.50 7.49 7.49 7.45 7.45 7.43 7.43 7.41 7.41 7.40 7.39 7.39 7.38 7.26 CDCl3 7.21 7.21 7.19 6.10
5.02 4.64 4.62 4.27 4.25
2.48 1.88 1.85 1.84 1.83 1.77 1.76 1.73 1.42 1.42 1.40 1.39 1.38 1.37 1.37 1.33 1.33 1.29 1.26 1.25 1.24 1.23 1.21 1.11
0.93 2.04 2.00 3.45 1.81 2.20 1.00 0.92 0.86 1.23 6.40 4.31 8.60
HO
O
N
H
O
O
12s
f1 (ppm)

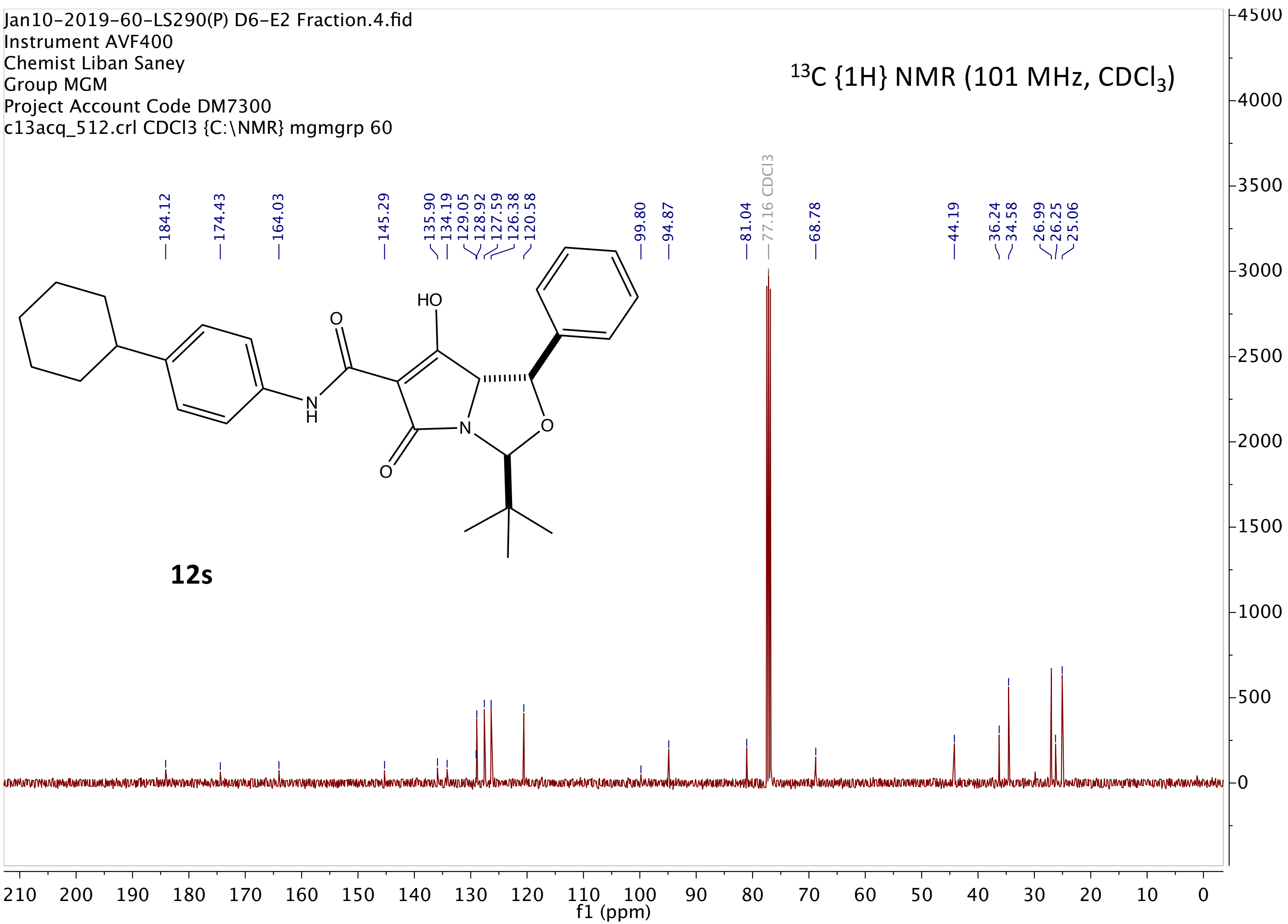
Jan10-2019-60-LS290(P) D6-E2 Fraction.4.fid
Instrument AVF400
Chemist Liban Saney
Group MGM
Project Account Code DM7300
c13acq_512.crl CDCl3 {C:\NMR} mgmgrp 60
13C {1H} NMR (101 MHz, CDCl3)
184.12
174.43
164.03
145.29
135.90
134.19
129.05
128.92
127.59
126.38
120.58
99.80
94.87
81.04
77.16 CDCl3
68.78
44.19
36.24
34.58
26.99
26.25
25.06
HO
O
N
H
O
O
12s
f1 (ppm)

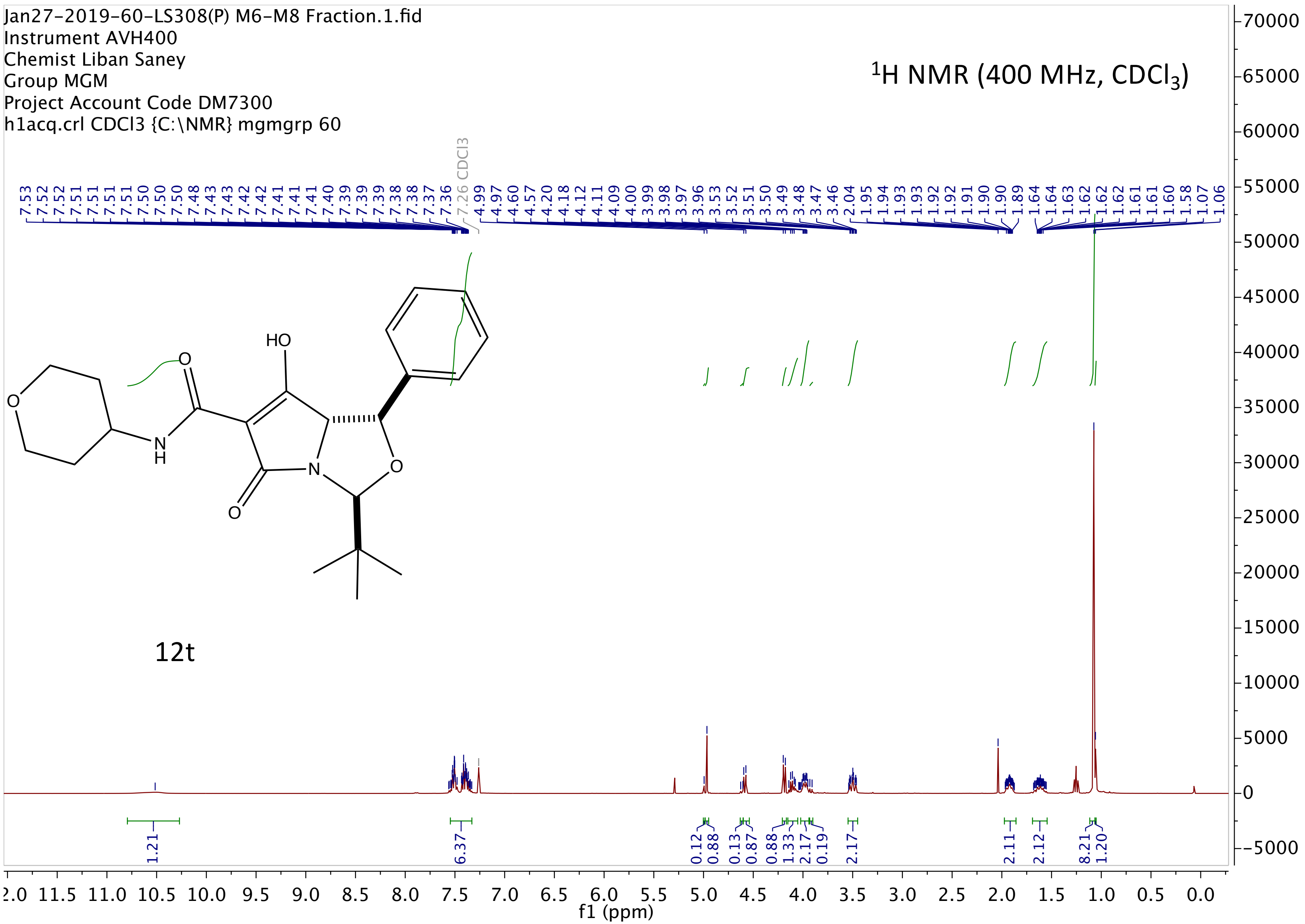
Jan27-2019-60-LS308(P) M6-M8 Fraction.1.fid
Instrument AVH400
Chemist Liban Saney
Group MGM
Project Account Code DM7300
h1acq.crl CDCl3 {C:\NMR} mgmgrp 60
1H NMR (400 MHz, CDCl3)
7.53 7.52 7.52 7.51 7.51 7.51 7.50 7.50 7.48 7.43 7.43 7.42 7.41 7.41 7.41 7.40 7.39 7.39 7.39 7.38 7.38 7.37 7.36
7.26 CDCl3
4.99 4.97 4.60 4.57 4.20 4.18 4.12 4.11 4.09 4.00 3.99 3.98 3.97 3.96 3.53 3.52 3.51 3.50 3.49 3.48 3.47 3.46 2.04 1.95 1.94 1.93 1.93 1.92 1.92 1.91 1.90 1.90 1.89 1.64 1.64 1.63 1.62 1.62 1.62 1.61 1.61 1.60 1.58 1.07 1.06
HO
O
N
H
N
O
O
O
12t
1.21 6.37 0.12 0.88 0.13 0.87 0.88 1.33 2.17 0.19 2.17 2.11 2.12 8.21 1.20
f1 (ppm)

Jan27-2019-60-LS308(P) M6-M8 Fraction.2.fid
Instrument AVH400
Chemist Liban Saney
Group MGM
Project Account Code DM7300
c13acq_512.crl CDCl3 {C:\NMR} mgmgrp 60

^{13}C {1H} NMR (101 MHz, $CDCl_3$)

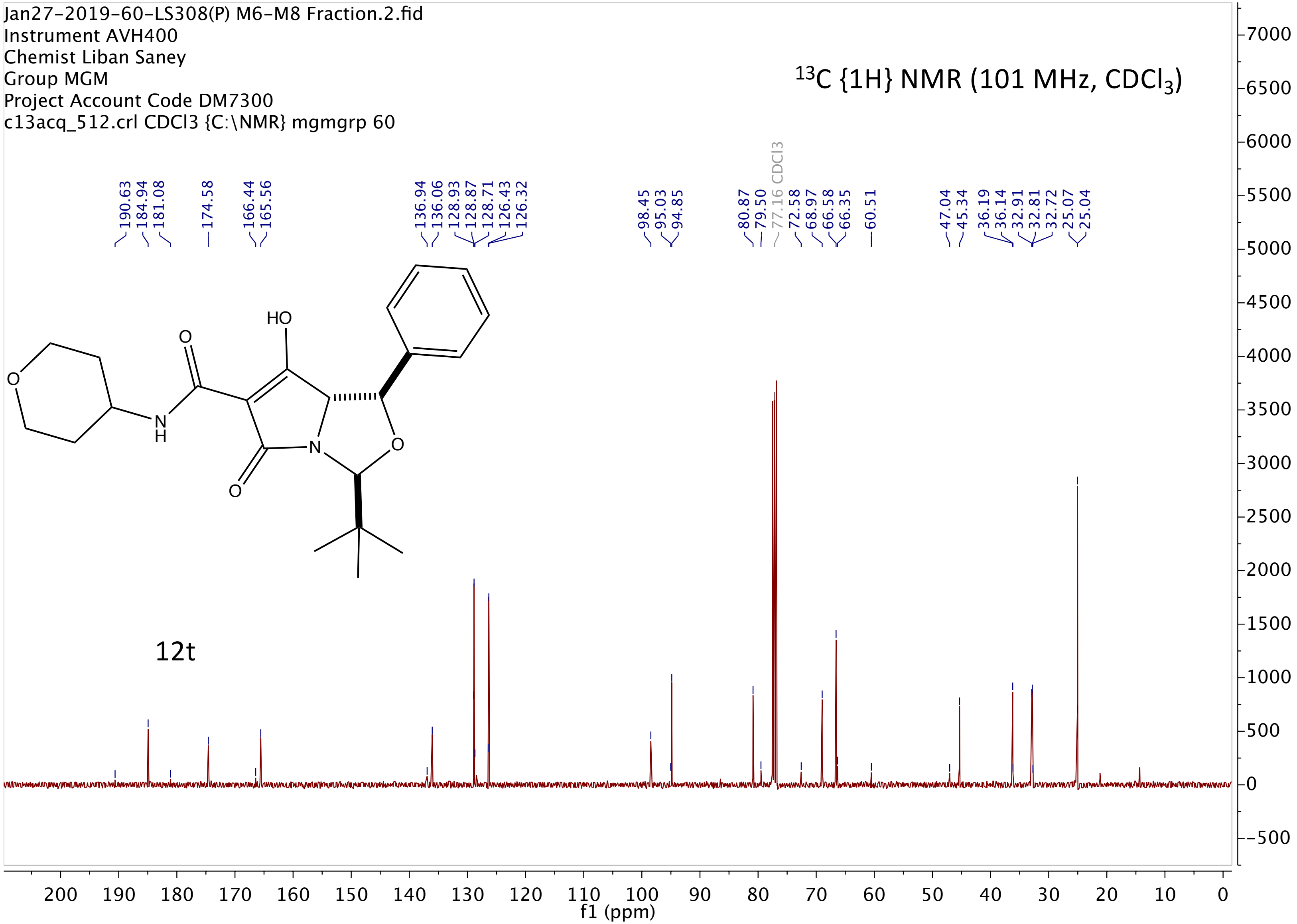

12t

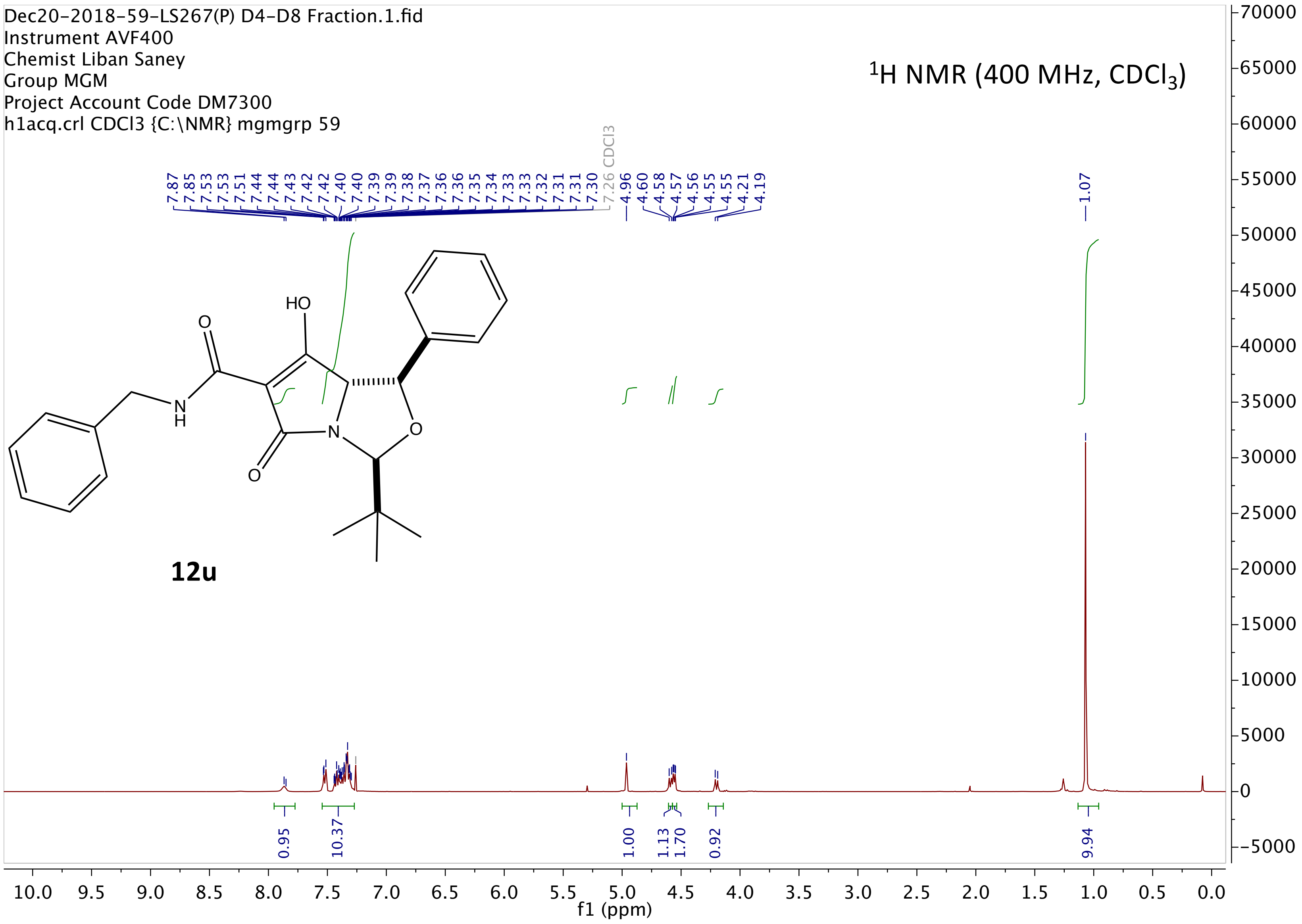
Dec20-2018-59-LS267(P) D4-D8 Fraction.1.fid
Instrument AVF400
Chemist Liban Saney
Group MGM
Project Account Code DM7300
h1acq.crl CDCl3 {C:\NMR} mgmgrp 59
1H NMR (400 MHz, CDCl3)
7.87 7.85 7.53 7.53 7.51 7.44 7.44 7.43 7.42 7.42 7.40 7.40 7.39 7.39 7.38 7.37 7.36 7.35 7.34 7.33 7.33 7.32 7.31 7.31 7.30
7.26 CDCl3
4.96 4.60 4.58 4.57 4.56 4.55 4.55 4.21 4.19
1.07
HO
O
N
H
N
O
O
12u
0.95
10.37
1.00
1.13
1.70
0.92
9.94
f1 (ppm)

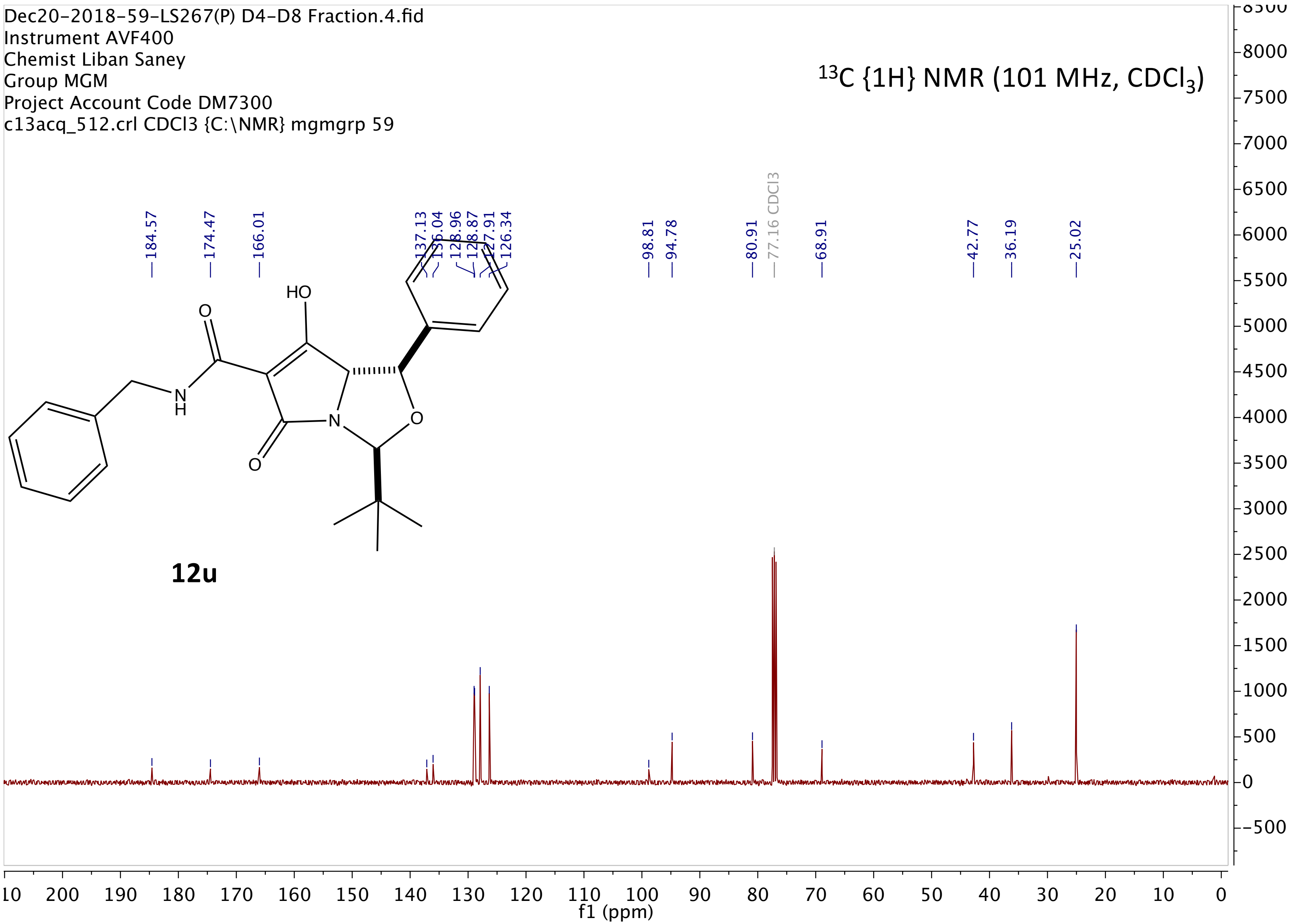
Dec20-2018-59-LS267(P) D4-D8 Fraction.4.fid
Instrument AVF400
Chemist Liban Saney
Group MGM
Project Account Code DM7300
c13acq_512.crl CDCl3 {C:\NMR} mgmgrp 59
13C {1H} NMR (101 MHz, CDCl3)
184.57
174.47
166.01
137.13
136.04
128.96
128.87
127.91
126.34
98.81
94.78
80.91
77.16 CDCl3
68.91
42.77
36.19
25.02
HO
O
N
H
N
O
O
12u
f1 (ppm)

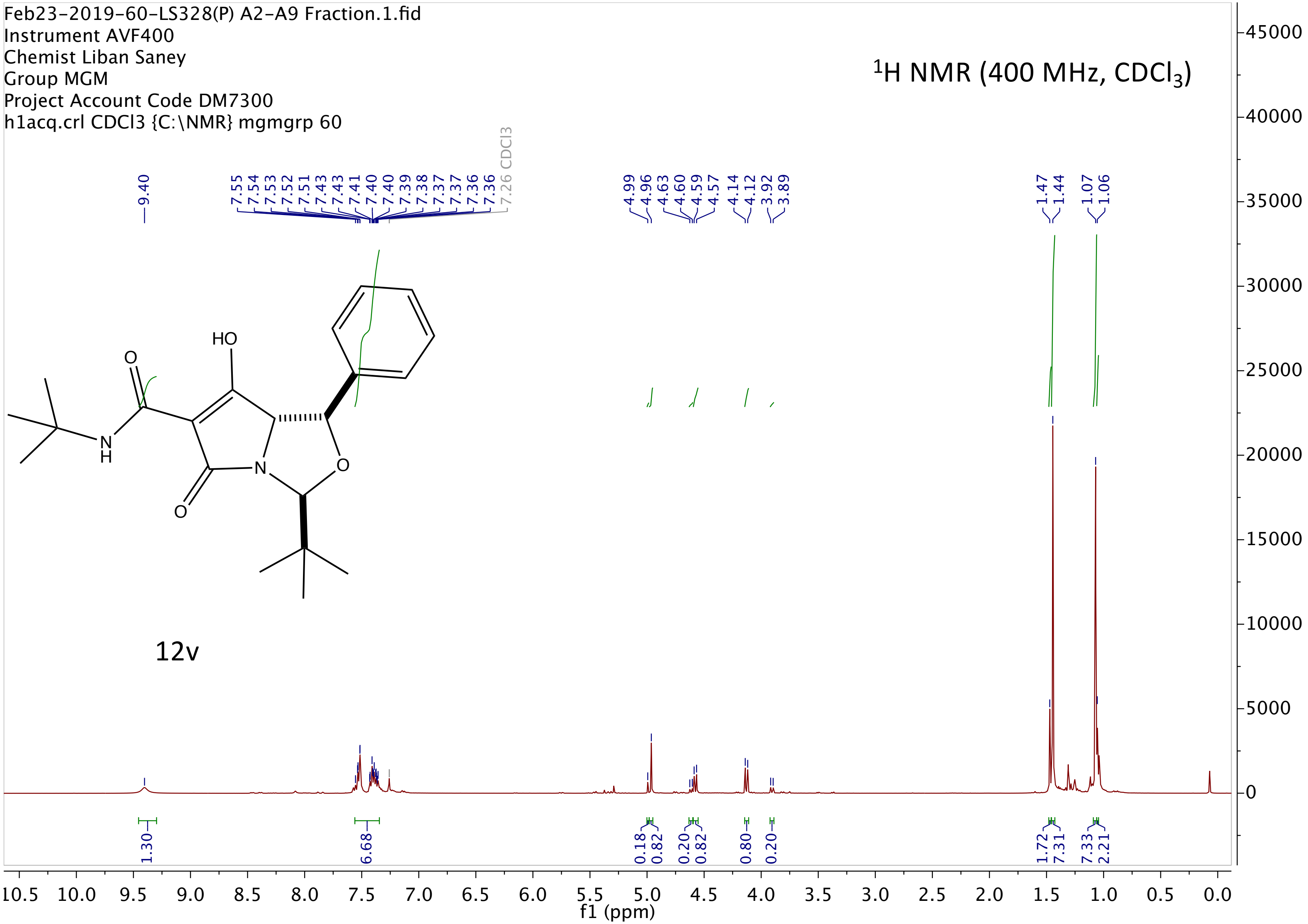
Feb23-2019-60-LS328(P) A2-A9 Fraction.1.fid
Instrument AVF400
Chemist Liban Saney
Group MGM
Project Account Code DM7300
h1acq.crl CDCl3 {C:\NMR} mgmgrp 60
1H NMR (400 MHz, CDCl3)
9.40
7.55 7.54 7.53 7.52 7.51 7.43 7.43 7.41 7.40 7.40 7.39 7.38 7.37 7.37 7.36 7.36
7.26 CDCl3
4.99 4.96 4.63 4.60 4.59 4.57 4.14 4.12 3.92 3.89
1.47 1.44 1.07 1.06
HO
O
N
H
N
O
O
12v
1.30
6.68
0.18 0.82 0.20 0.82 0.80 0.20
1.72 7.31 7.33 2.21
f1 (ppm)

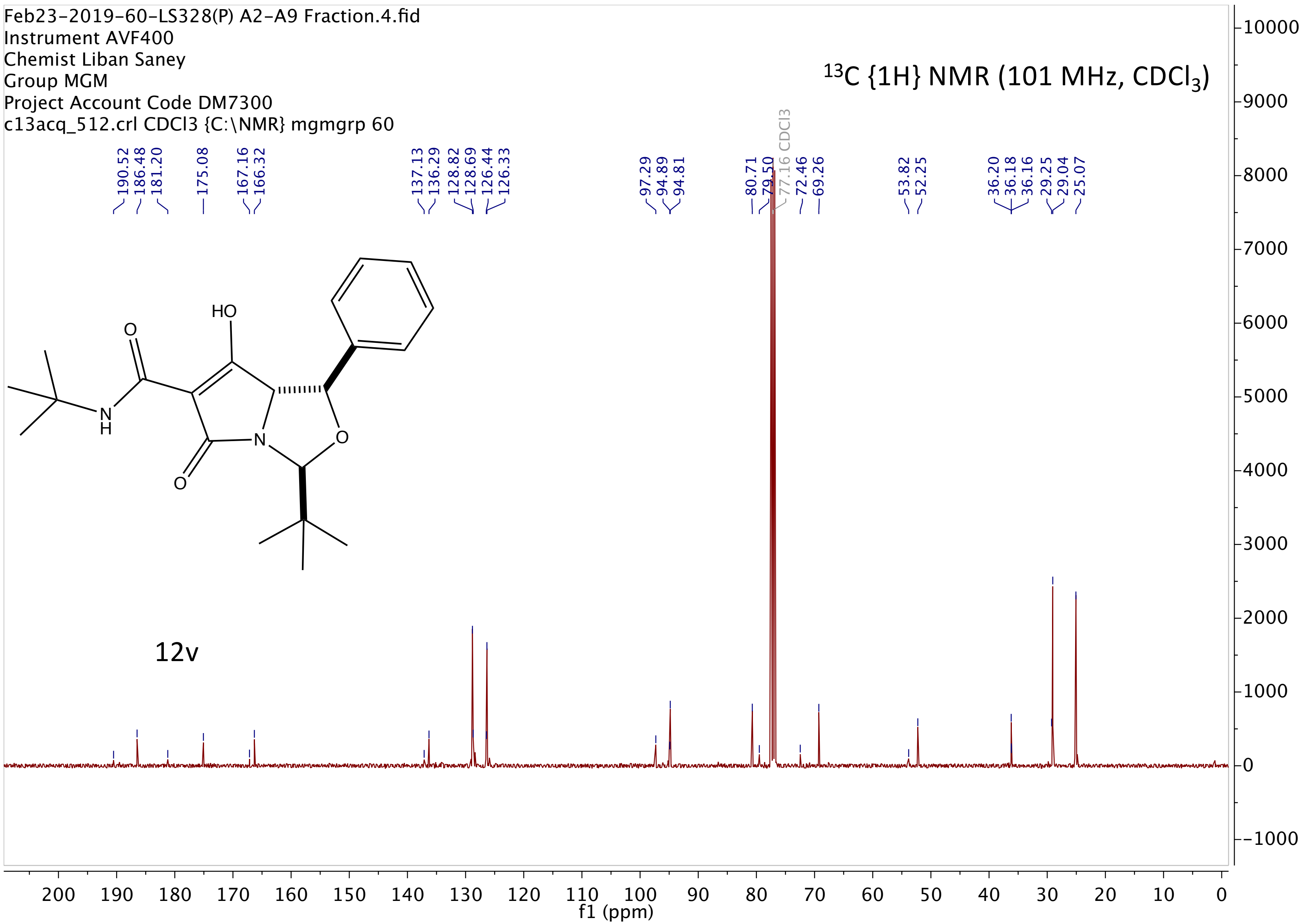
Feb23-2019-60-LS328(P) A2-A9 Fraction.4.fid
Instrument AVF400
Chemist Liban Saney
Group MGM
Project Account Code DM7300
c13acq_512.crl CDCl3 {C:\NMR} mgmgrp 60
^{13}C {1H} NMR (101 MHz, CDCl$_3$)
190.52
186.48
181.20
175.08
167.16
166.32
137.13
136.29
128.82
128.69
126.44
126.33
97.29
94.89
94.81
80.71
79.50
77.16 CDCl3
72.46
69.26
53.82
52.25
36.20
36.18
36.16
29.25
29.04
25.07
HO
O
N
H
N
O
O
12v
f1 (ppm)

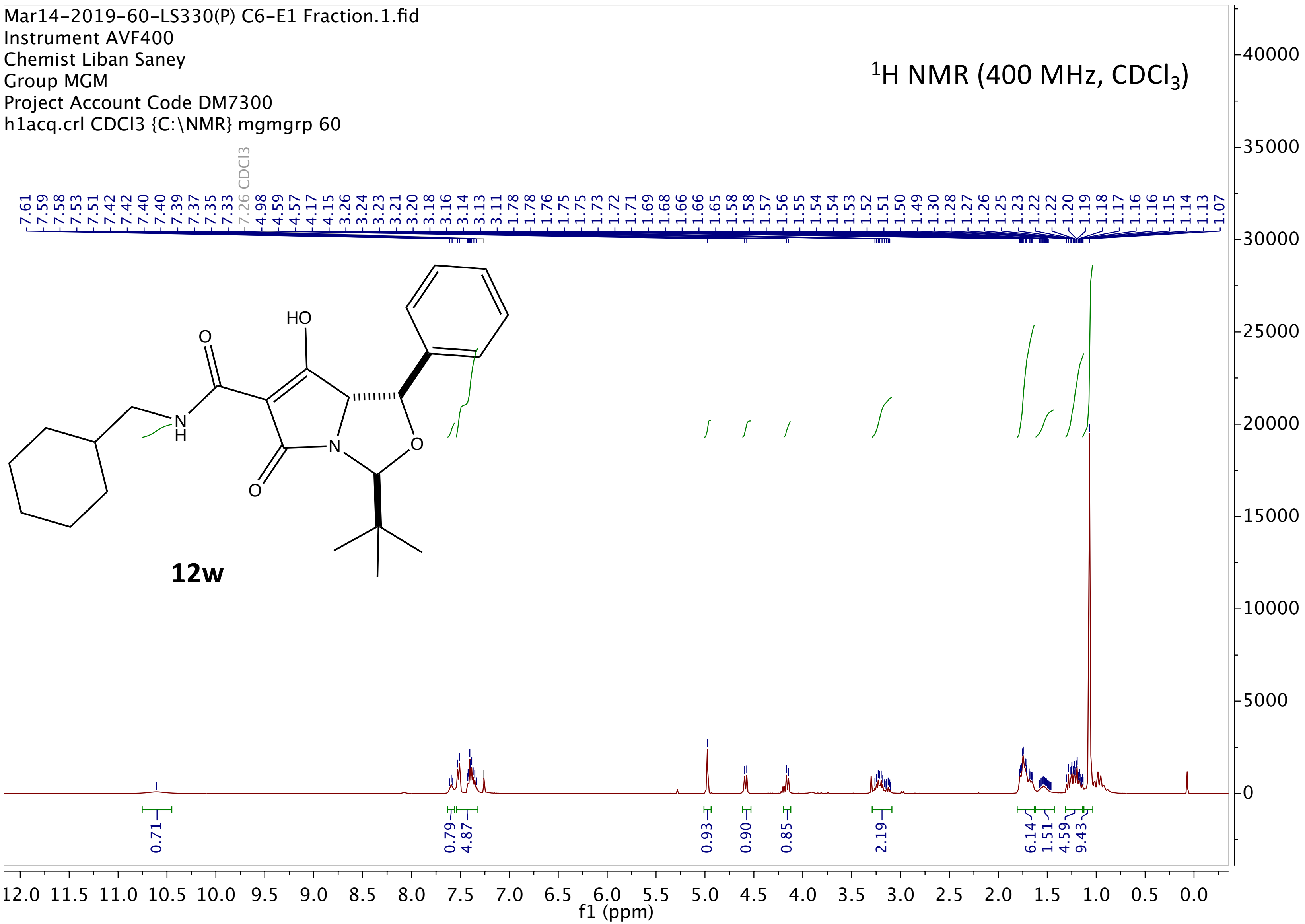
Mar14-2019-60-LS330(P) C6-E1 Fraction.1.fid
Instrument AVF400
Chemist Liban Saney
Group MGM
Project Account Code DM7300
h1acq.crl CDCl3 {C:\NMR} mgmgrp 60
¹H NMR (400 MHz, CDCl3)
7.61 7.59 7.58 7.53 7.51 7.42 7.42 7.40 7.40 7.39 7.37 7.35 7.33 7.26 CDCl3 4.98 4.59 4.57 4.17 4.15 3.26 3.24 3.23 3.21 3.20 3.18 3.16 3.14 3.13 3.11 1.78 1.78 1.76 1.75 1.75 1.73 1.72 1.71 1.69 1.68 1.66 1.65 1.58 1.58 1.57 1.56 1.55 1.54 1.53 1.52 1.51 1.50 1.49 1.30 1.28 1.27 1.26 1.25 1.23 1.22 1.22 1.20 1.19 1.18 1.17 1.16 1.16 1.15 1.14 1.13 1.07
HO
O
N
H
N
O
O
12w
0.71
0.79
4.87
0.93
0.90
0.85
2.19
6.14
1.51
4.59
9.43
f1 (ppm)

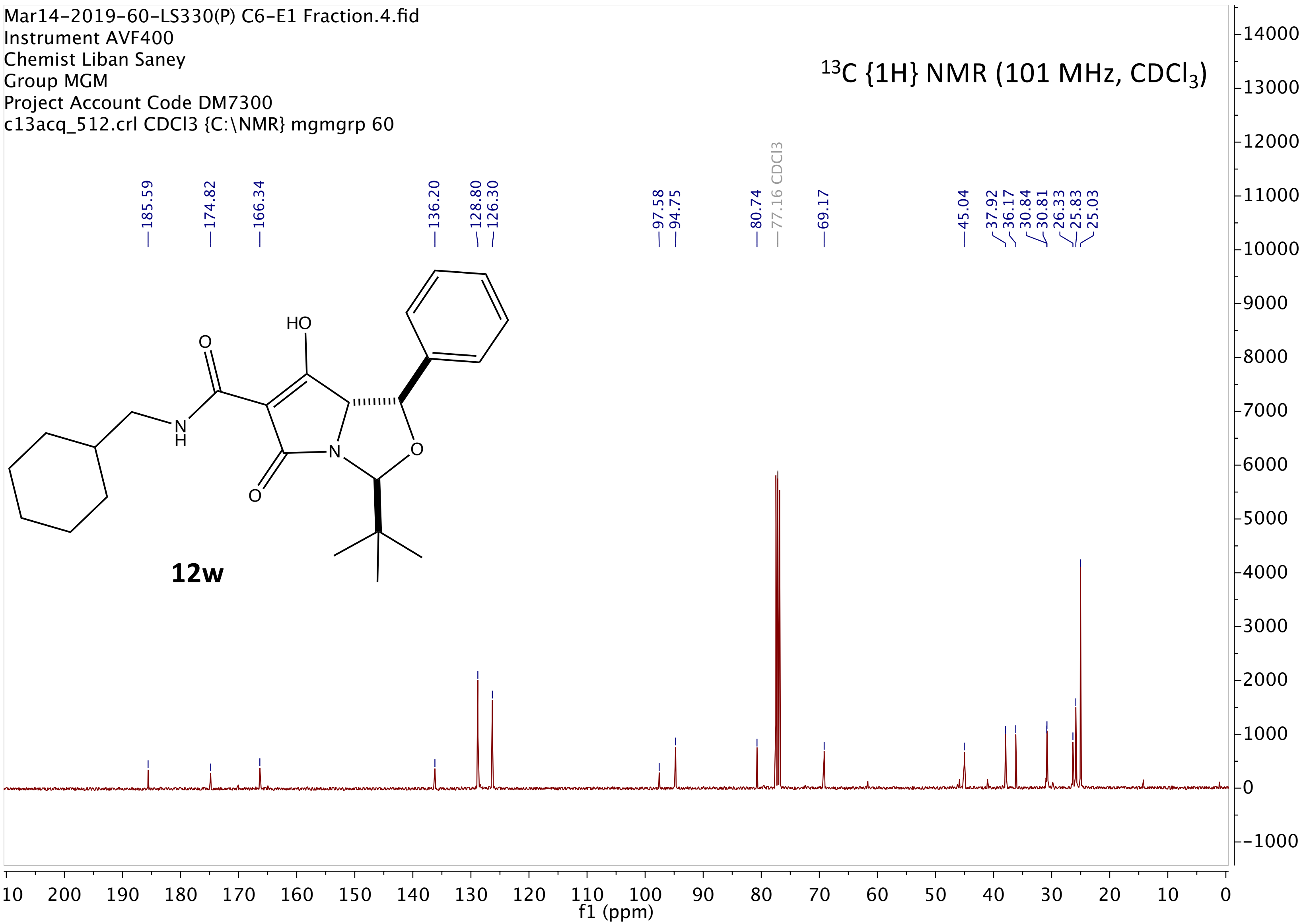
Mar14-2019-60-LS330(P) C6-E1 Fraction.4.fid
Instrument AVF400
Chemist Liban Saney
Group MGM
Project Account Code DM7300
c13acq_512.crl CDCl3 {C:\NMR} mgmgrp 60
13C {1H} NMR (101 MHz, CDCl3)
185.59
174.82
166.34
136.20
128.80
126.30
97.58
94.75
80.74
77.16 CDCl3
69.17
45.04
37.92
36.17
30.84
30.81
26.33
25.83
25.03
HO
O
N
H
N
O
O
12w
14000
13000
12000
11000
10000
9000
8000
7000
6000
5000
4000
3000
2000
1000
0
-1000
10
200
190
180
170
160
150
140
130
120
110
100
90
80
70
60
50
40
30
20
10
0
f1 (ppm)

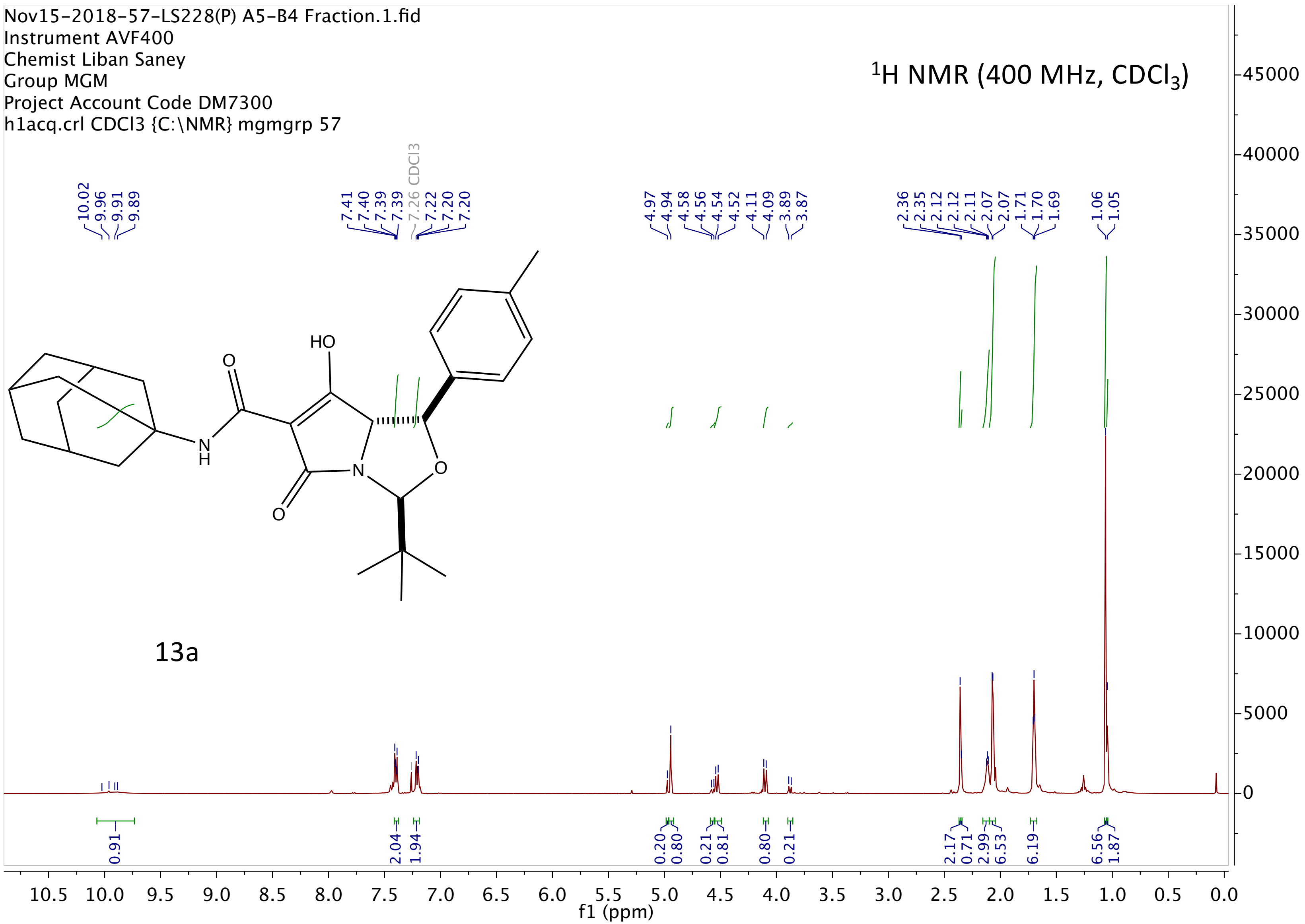
Nov15-2018-57-LS228(P) A5-B4 Fraction.1.fid
Instrument AVF400
Chemist Liban Saney
Group MGM
Project Account Code DM7300
h1acq.crl CDCl3 {C:\NMR} mgmgrp 57
1H NMR (400 MHz, CDCl3)
10.02 9.96 9.91 9.89
7.41 7.40 7.39 7.39 7.26 CDCl3 7.22 7.20 7.20
4.97 4.94 4.58 4.56 4.54 4.52 4.11 4.09 3.89 3.87
2.36 2.35 2.12 2.12 2.11 2.07 2.07 1.71 1.70 1.69
1.06 1.05
HO
O
N
H
N
O
O
13a
0.91 2.04 1.94 0.20 0.80 0.21 0.81 0.80 0.21 2.17 0.71 2.99 6.53 6.19 6.56 1.87
f1 (ppm)

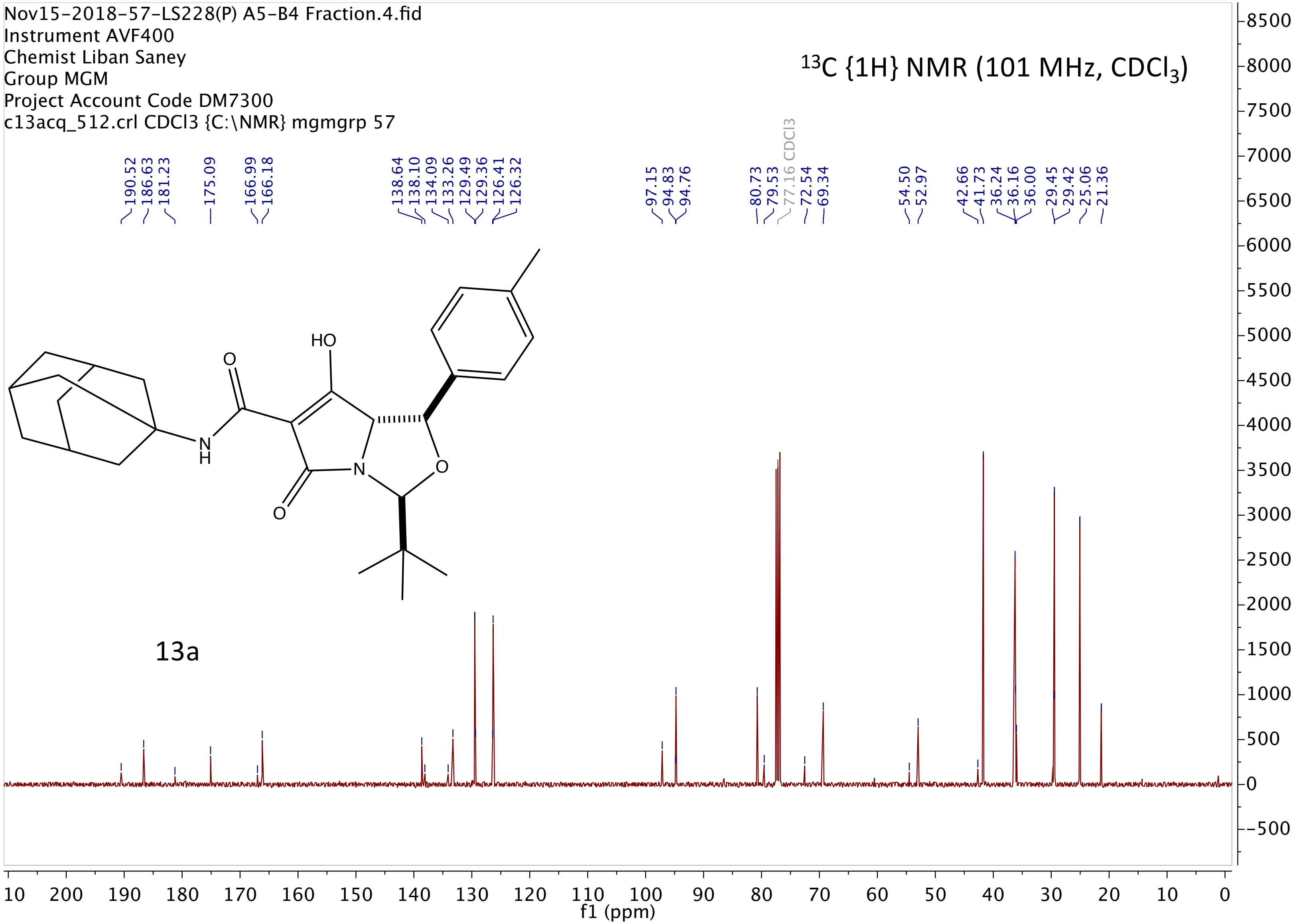
Nov15-2018-57-LS228(P) A5-B4 Fraction.4.fid
Instrument AVF400
Chemist Liban Saney
Group MGM
Project Account Code DM7300
c13acq_512.crl CDCl3 {C:\NMR} mgmgrp 57
13C {1H} NMR (101 MHz, CDCl3)
190.52
186.63
181.23
175.09
166.99
166.18
138.64
138.10
134.09
133.26
129.49
129.36
126.41
126.32
97.15
94.83
94.76
80.73
79.53
77.16 CDCl3
72.54
69.34
54.50
52.97
42.66
41.73
36.24
36.16
36.00
29.45
29.42
25.06
21.36
HO
O
N
H
N
O
O
13a
f1 (ppm)

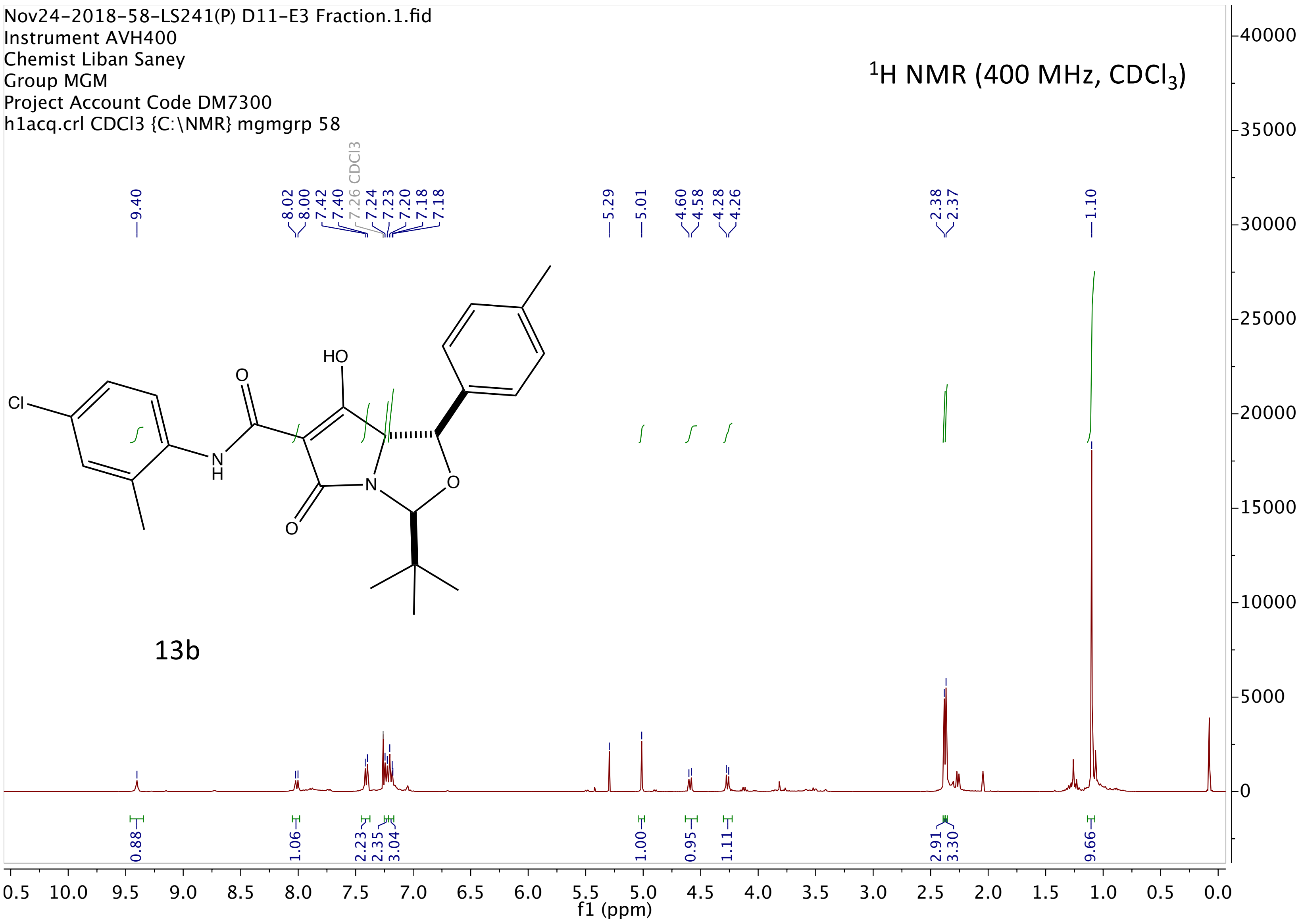
Nov24-2018-58-LS241(P) D11-E3 Fraction.1.fid
Instrument AVH400
Chemist Liban Saney
Group MGM
Project Account Code DM7300
h1acq.crl CDCl3 {C:\NMR} mgmgrp 58
1H NMR (400 MHz, CDCl3)
9.40
8.02
8.00
7.42
7.40
7.26 CDCl3
7.24
7.23
7.20
7.18
7.18
5.29
5.01
4.60
4.58
4.28
4.26
2.38
2.37
1.10
HO
O
Cl
N
H
N
O
O
13b
0.88
1.06
2.23
2.35
3.04
1.00
0.95
1.11
2.91
3.30
9.66
f1 (ppm)

Nov24-2018-58-LS241(P) D11-E3 Fraction.4.fid
Instrument AVH400
Chemist Liban Saney
Group MGM
Project Account Code DM7300
c13acq_512.crl CDCl3 {C:\NMR} mgmgrp 58

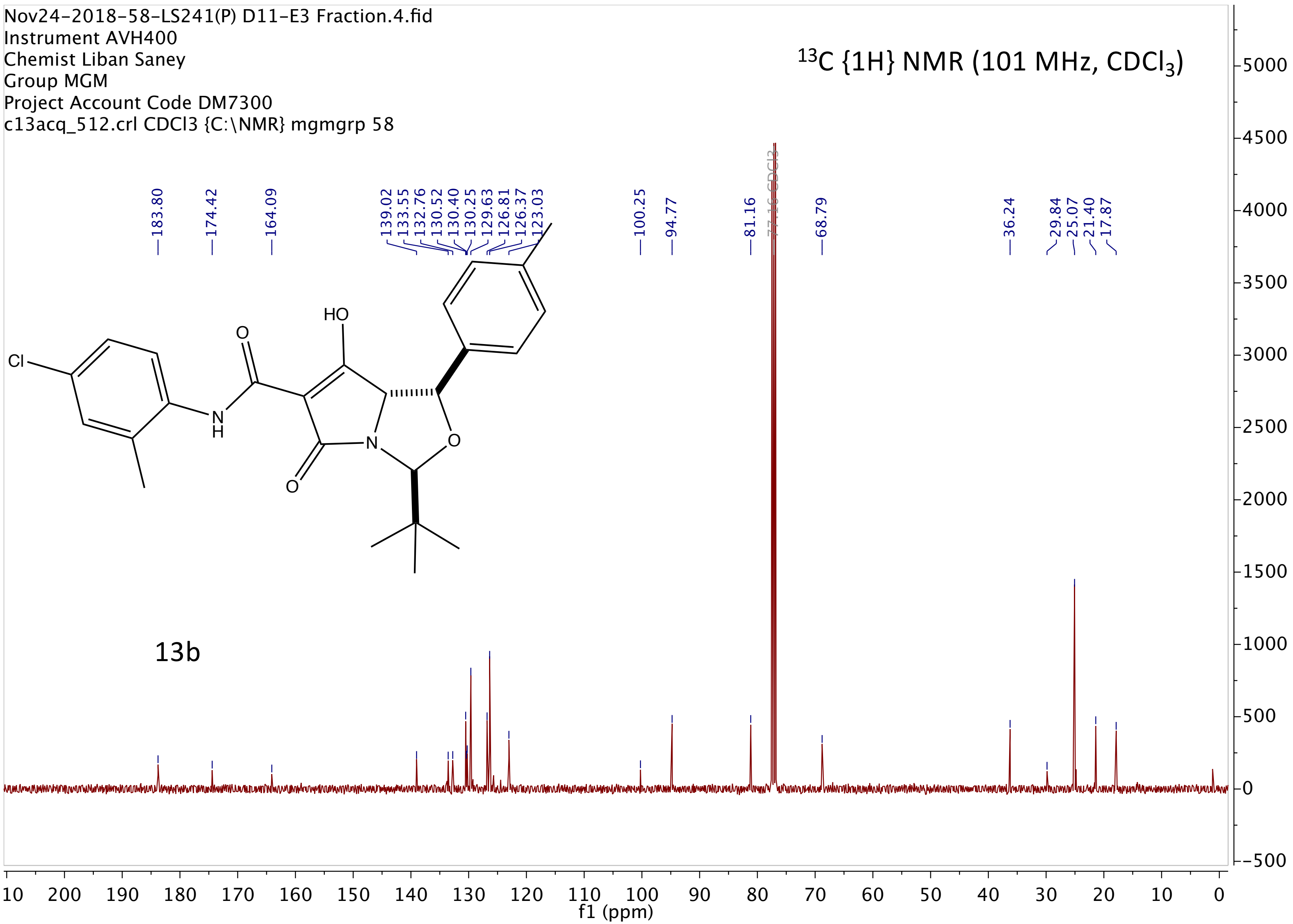

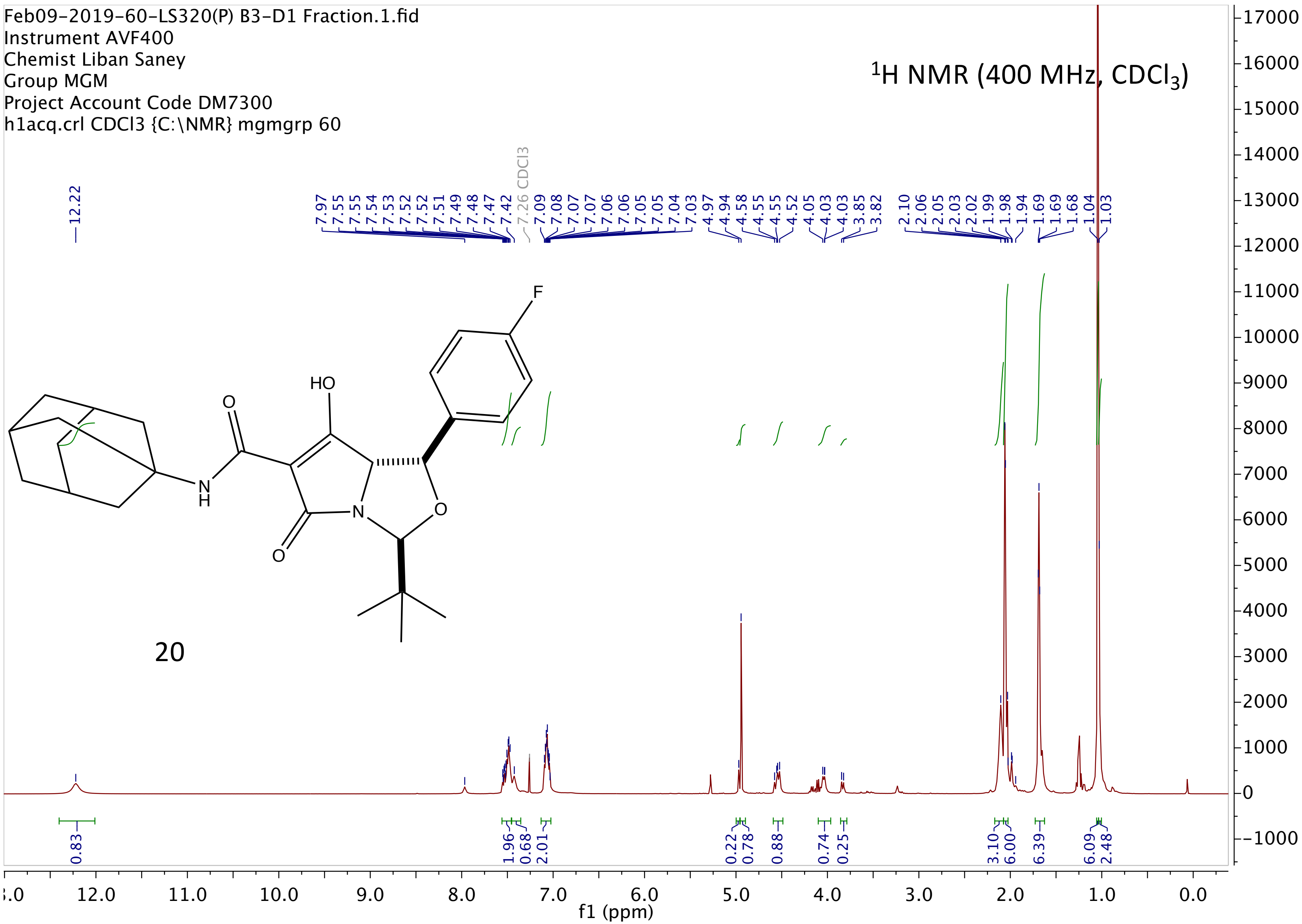
Feb09-2019-60-LS320(P) B3-D1 Fraction.1.fid
Instrument AVF400
Chemist Liban Saney
Group MGM
Project Account Code DM7300
h1acq.crl CDCl3 {C:\NMR} mgmgrp 60
1H NMR (400 MHz, CDCl3)
20
f1 (ppm)

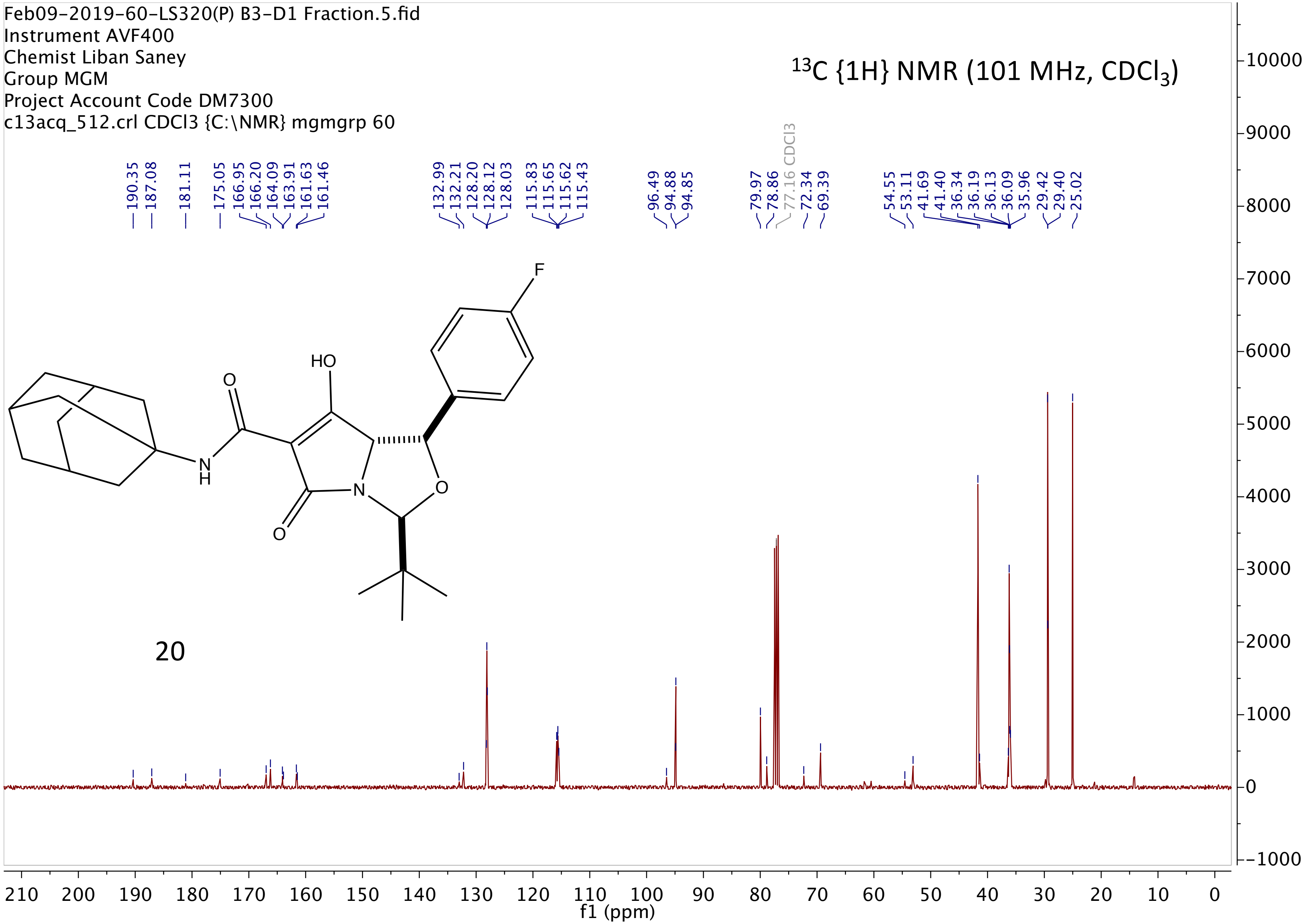
Feb09-2019-60-LS320(P) B3-D1 Fraction.5.fid
Instrument AVF400
Chemist Liban Saney
Group MGM
Project Account Code DM7300
c13acq_512.crl CDCl3 {C:\NMR} mgmgrp 60
^{13}C {1H} NMR (101 MHz, $CDCl_3$)
190.35
187.08
181.11
175.05
166.95
166.20
164.09
163.91
161.63
161.46
132.99
132.21
128.20
128.12
128.03
115.83
115.65
115.62
115.43
96.49
94.88
94.85
79.97
78.86
77.16 CDCl3
72.34
69.39
54.55
53.11
41.69
41.40
36.34
36.19
36.13
36.09
35.96
29.42
29.40
25.02
F
HO
O
N
H
N
O
O
20
f1 (ppm)

Feb09-2019-60-LS320(P) B3-D1 Fraction.7.fid
Instrument AVF400
Chemist Liban Saney
Group MGM
Project Account Code DM7300
f19acq.crl CDCl3 {C:\NMR} mgmgrp 60

^{19}F NMR (377 MHz, $CDCl_3$)

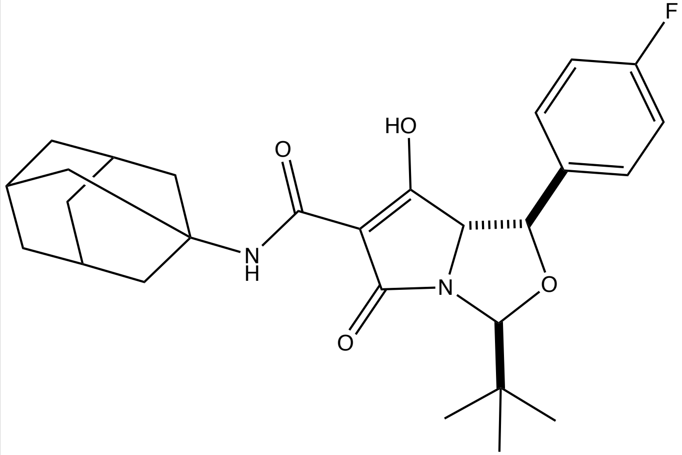

20

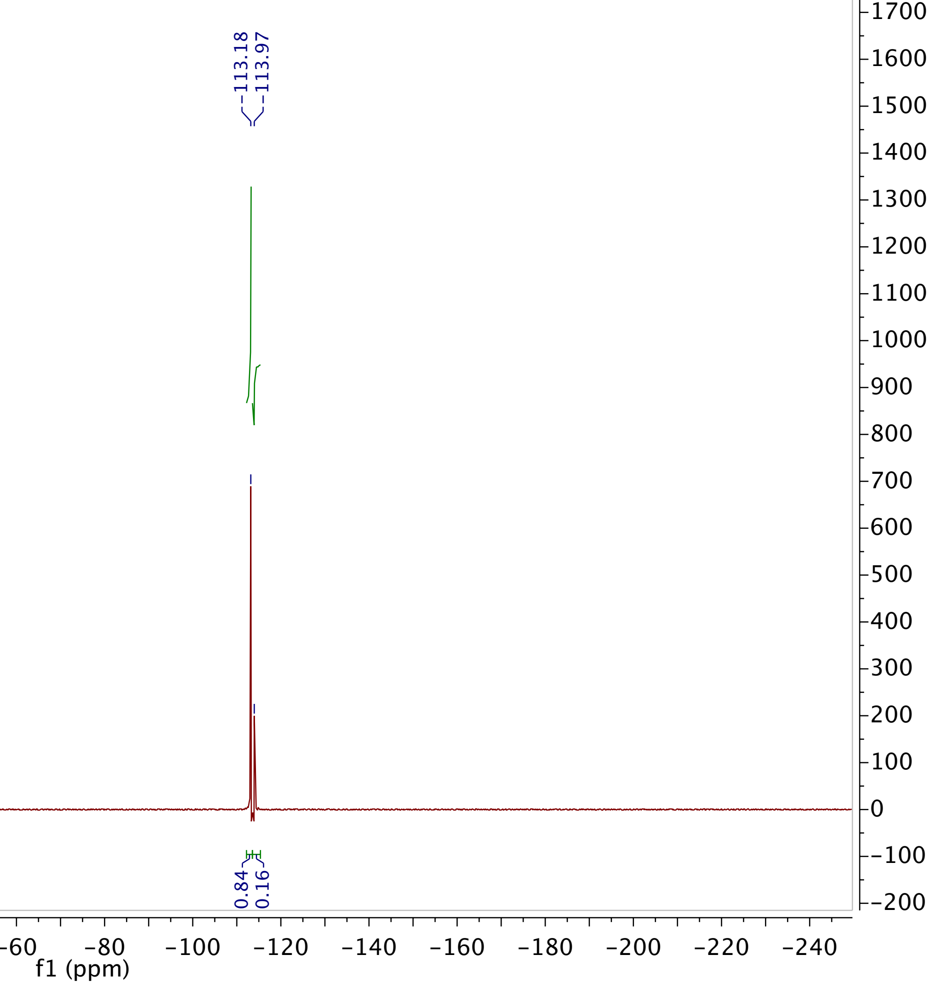

Feb14-2019-59-LS324(P) F1-G5 Fraction.1.fid
Instrument AVF400
Chemist Liban Saney
Group MGM
Project Account Code DM7300
h1acq.crl CDCl3 {C:\NMR} mgmgrp 59

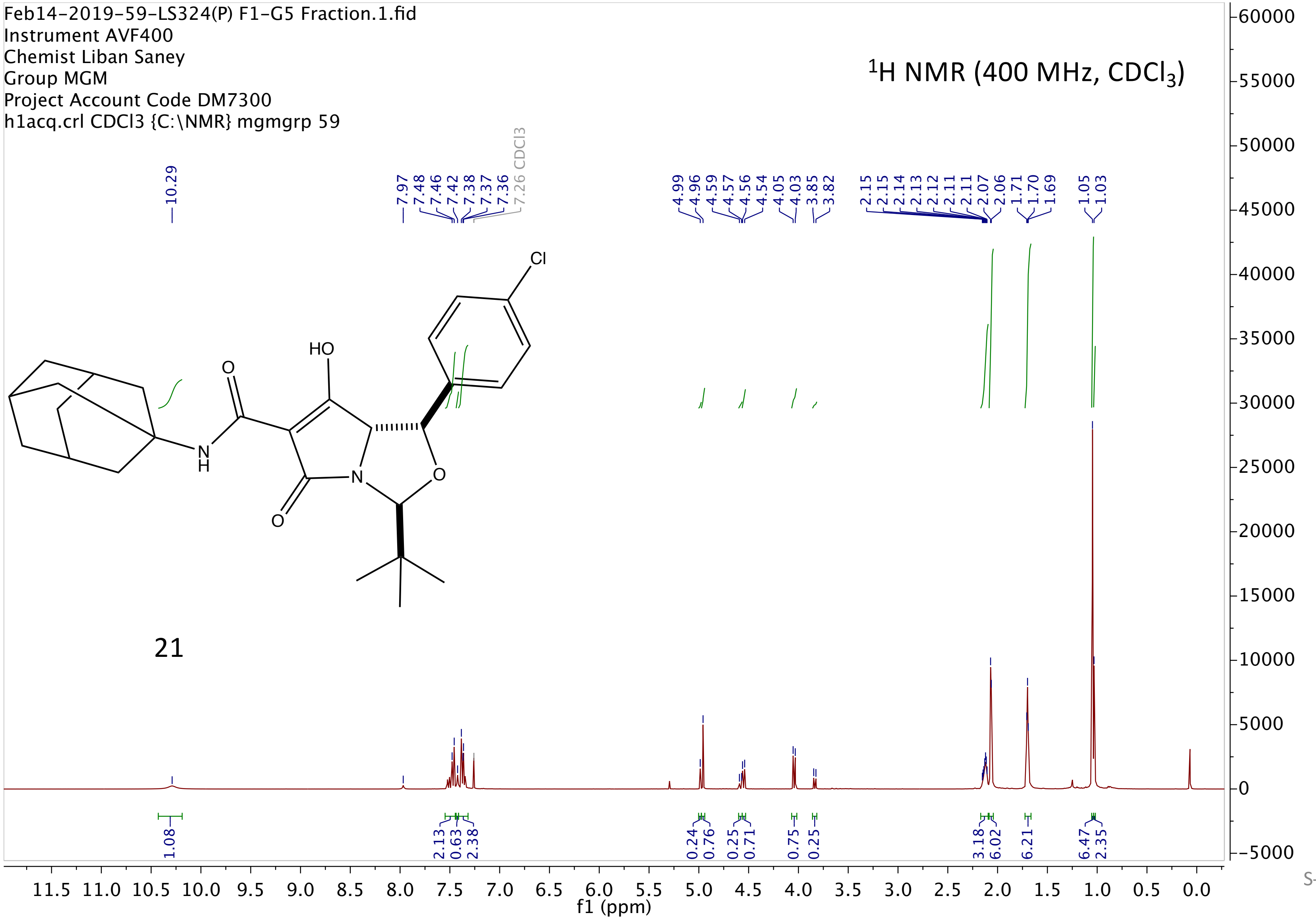

21

Feb14-2019-59-LS324(P) F1-G5 Fraction.4.fid
Instrument AVF400
Chemist Liban Saney
Group MGM
Project Account Code DM7300
c13acq_512.crl CDCl3 {C:\NMR} mgmgrp 59

^{13}C {1H} NMR (101 MHz, $CDCl_3$)

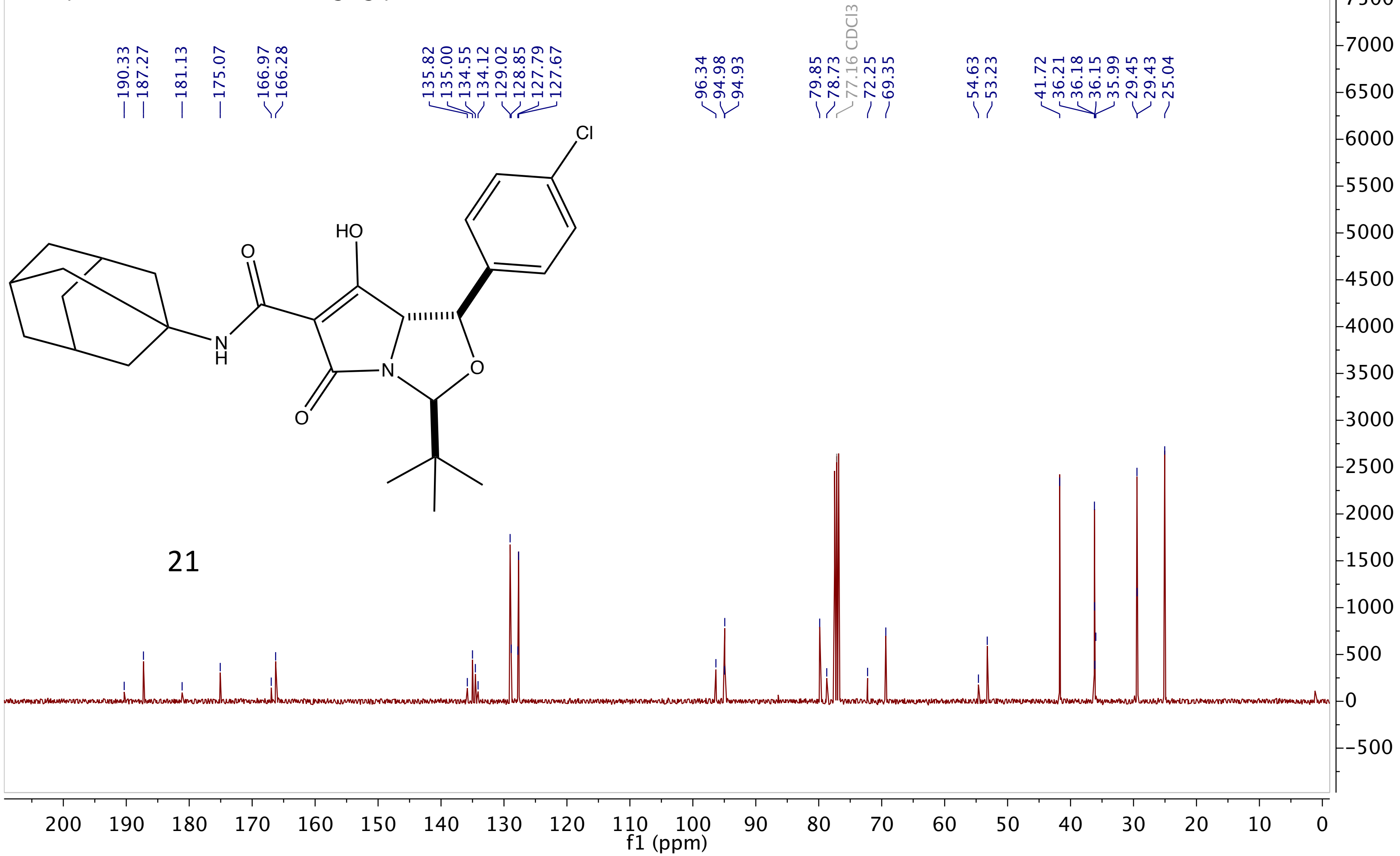

21

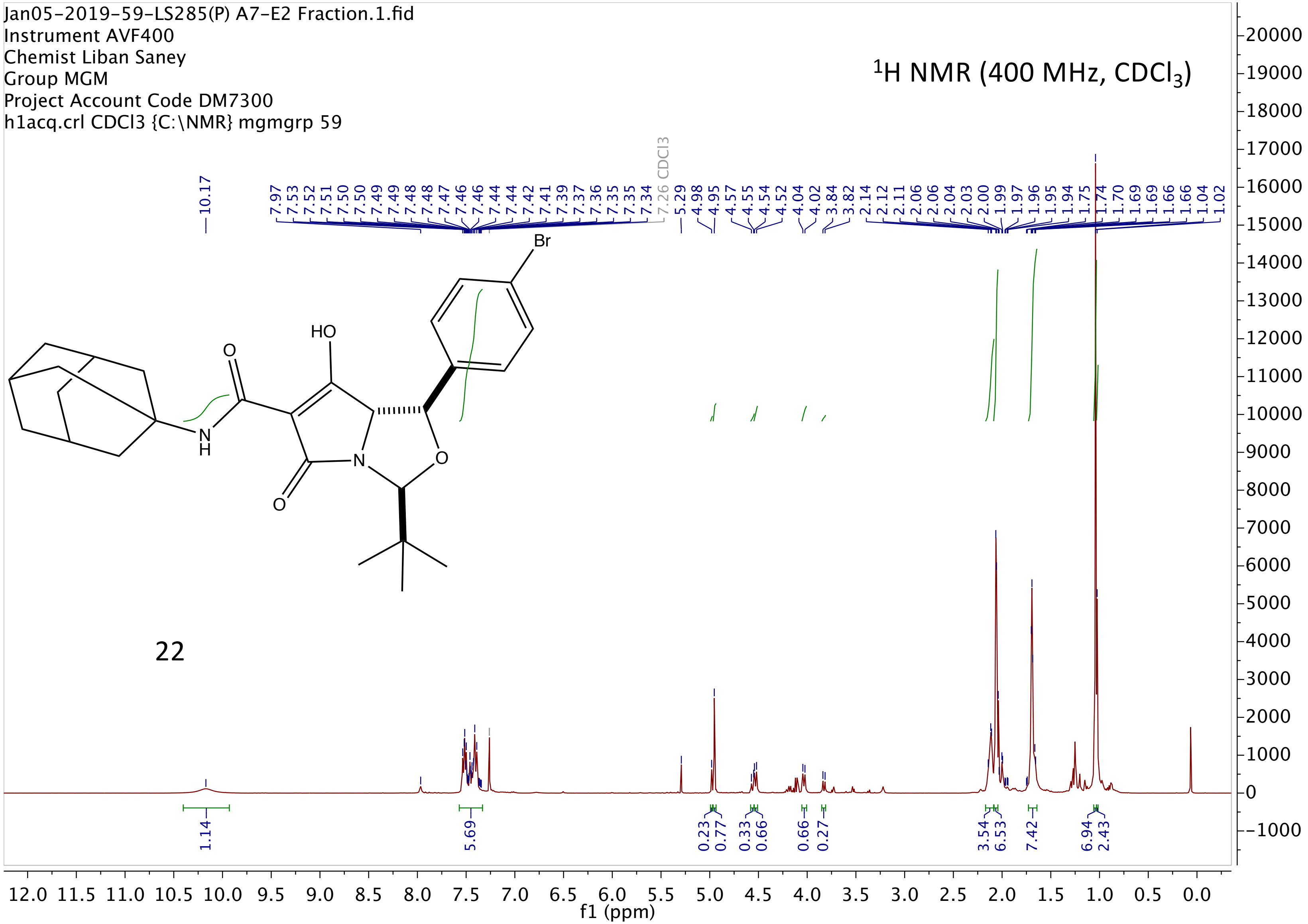
Jan05-2019-59-LS285(P) A7-E2 Fraction.1.fid
Instrument AVF400
Chemist Liban Saney
Group MGM
Project Account Code DM7300
h1acq.crl CDCl3 {C:\NMR} mgmgrp 59
¹H NMR (400 MHz, CDCl₃)
22
f1 (ppm)

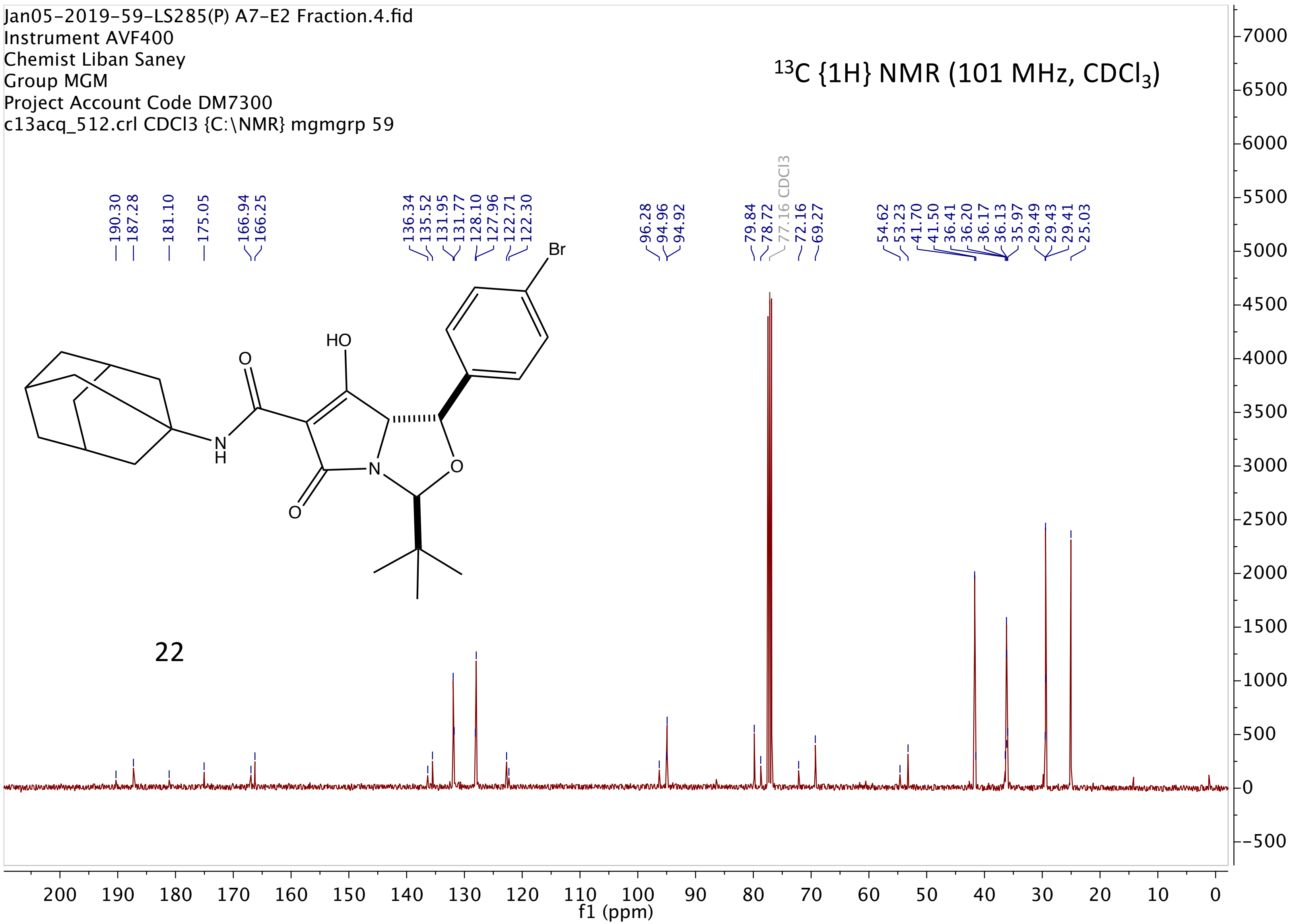

Jan05-2019-59-LS285(P) A7-E2 Fraction.4.fid
Instrument AVF400
Chemist Liban Saney
Group MGM
Project Account Code DM7300
c13acq_512.crl CDCl3 {C:\NMR} mgmgrp 59
13C {1H} NMR (101 MHz, CDCl3)
190.30
187.28
181.10
175.05
166.94
166.25
136.34
135.52
131.95
131.77
128.10
127.96
122.71
122.30
96.28
94.96
94.92
79.84
78.72
77.16 CDCl3
72.16
69.27
54.62
53.23
41.70
41.50
36.41
36.20
36.17
36.13
35.97
29.49
29.43
29.41
25.03
Br
HO
O
N
H
N
O
O
22
f1 (ppm)
